# A selenoxide for single-atom protein modification of tyrosine residues enabled by water-resistant chalcogen and hydrogen bonding

**DOI:** 10.1038/s41557-025-01842-8

**Published:** 2025-06-04

**Authors:** Songyun Lin, Marina Hirao, Philipp Hartmann, Markus Leutzsch, Marie Sophie Sterling, Alessandro Vetere, Sandra Klimmek, Heike Hinrichs, Johanna Marie Mengeler, Johannes Lehmann, Jan Samsonowicz-Gόrski, Florian Berger, Tobias Ritter

**Affiliations:** 1 Max-Planck-Institut für Kohlenforschung, Mülheim an der Ruhr, Germany; 2 Institute of Organic Chemistry, RWTH Aachen University, Aachen, Germany

**Keywords:** Synthetic chemistry methodology, Organic chemistry

## Abstract

Post-translational modifications such as phosphorylation and acetylation are often minor structural modifications that can have profound effects on protein structure and thus broaden protein functions. Nevertheless, studying these effects directly is often out of reach because no general chemistry exists to introduce small modifications selectively; either a large, stable linker structure is selectively installed on protein residues, or a small substituent is introduced at the risk of low selectivity due to the use of reactive, indiscriminate molecules. Here we report a C–H functionalization reaction of tyrosine residues to access peptides and proteins modified by small structural changes including single-atom substitutions. A rationally designed selenoxide introduces a versatile selenonium linchpin featuring a C_tyr_–Se bond that can be used for further transformations at specific tyrosine residues. Key to the advance is the interplay of water-resistant, intramolecular chalcogen and hydrogen bonding of the selenoxide reagent, which allows chemo- and site-selective electrophilic aromatic substitution of tyrosine residues in aqueous solutions.

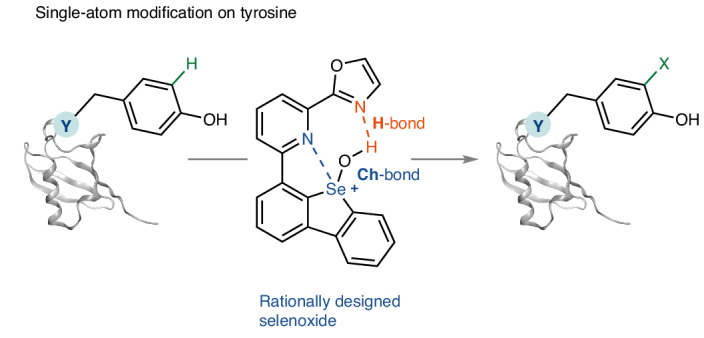

## Abstract (web summary)

Proteins with small structural modifications at specific sites are valuable, yet challenging to access by chemical methods. Now, tyrosine-selective single-atom modifications on proteins have been achieved by C–H functionalization using a rationally designed selenoxide to introduce a versatile selenonium linchpin for further transformations.

## Main

Post-translational modifications (PTMs) of proteins and peptides play multifaceted roles in numerous biochemical processes, including signal transduction^1,2^, metabolic regulation^3^ and cell proliferation^3,4^. Chemically modified peptides and proteins are also valuable in imaging, biomaterials and drug delivery^5–7^, and even single-atom modifications can have a profound impact such as increased cellular uptake^8^ and enhanced receptor binding^9^. Peptides and proteins with PTMs are mainly obtained by expression from specific cell lines or by in vitro enzymatic modification^10^. However, the range of peptidyl substrates and the types of modification are restricted by the genetic machinery and enzyme functions^11^. Chemical synthesis and semisynthesis of proteins with PTMs by native chemical ligation^12^ and by expressed protein ligation^9,13^ are complementary approaches, but are work intensive^13^. Chemical methods for the introduction of small structural perturbations to native proteins are attractive because they could expand the scope of modified products while also avoiding complex molecular biology operations and laborious protein synthesis. Due to the stringent requirements of large biomolecules, including the necessity for an aqueous environment within a small temperature window, a low (micromolar) concentration of the protein substrate, and site- and chemoselectivity^11,14,15^, chemical methods that can selectively functionalize endogenous proteins or peptides with only small structural perturbations, for example, without a linker structure, are scarce. The Davis group reported versatile post-translational protein editing by chemoselective functionalization of cysteine residues that, with the appropriate chemistry, results in synthetically useful dehydroalanine substituents^16^ or radicals^17^ for further diversification. Modification of native proteins can also be accomplished with small, reactive molecules, such as light-induced nitration^18^ or halogenation^19^. Such chemistry can be chemoselective but the energetic small molecules used in this chemistry such as nitrogen dioxide and halogen radicals can make the control of site-selectivity difficult. Chemo- and site-selectivity are already challenging for small-molecule C–H functionalization, and the challenges are exacerbated for late-stage functionalization of polypeptides and proteins. Despite the utility of current chemical^20–22^ and biochemical^11,14,15^ late-stage functionalization, the regioselective introduction of a linchpin by C–H functionalization chemistry for subsequent diversification, including single-atom modifications, is currently unknown in the field of bioconjugation, yet would afford valuable probes and analogues with small structural changes.

Tyrosine residues are an attractive target for site-selective modification due to their small surface abundance^23^. Small modifications of tyrosine residues enable fine-tuning of the p*K*_a_ of the phenolic hydroxyl group^24^, the nucleophilicity of phenolate^25^ and the redox potential of the tyrosine *π* system^24,26^, which can impact the activity of peptidyl drugs^9,27^ and protein–protein interactions^8^. However, current chemical methods are generally not suitable for functionalization of peptides and proteins with small and single-atom modifications on tyrosine, in part because the desired atoms cannot be selectively introduced directly^18,19^ and because the larger groups that can be introduced selectively form stable C–C (ref. ^28^), C–O (ref. ^29^) or C–N (ref. ^30^) bonds that cannot be readily cleaved subsequently. In general, the use of mild reagents due to the restrictions of selectivity and conditions leads to the formation of a stable linkage on the modified residues. Based on our knowledge, the chemoselective introduction of convertible substituents with a weak, cleavable linkage is only achieved on the side chains of cysteine^16,17^, selenocysteine^31,32^ or selenomethionine^33^ residues. In other words, heavy main-group elements such as sulfur and selenium must be involved in such linkages.

**Fig. 1:**
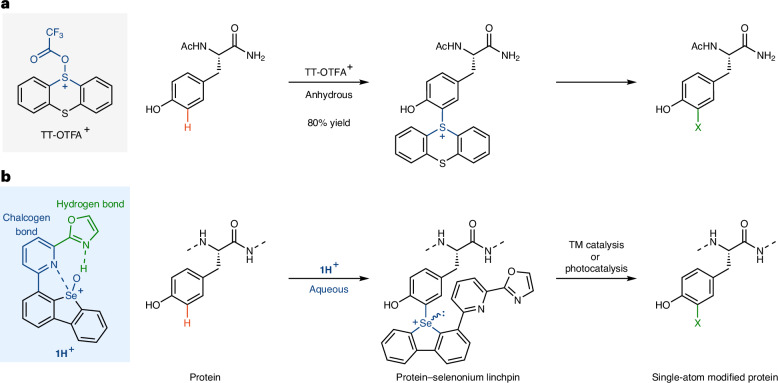
Selenoxide-based electrophilic aromatic substitution. **a**, Thianthrenation: although versatile and highly selective, functionalization with the water-sensitive TT-OTFA^+^ can only proceed under anhydrous conditions. **b**, This work: positional-selective, versatile single-atom protein modification with a selenium-based electrophile. The more basic selenoxide group, and the combination of the water-resistant, intramolecular chalcogen and hydrogen bonding, leads to the water-stable electrophile **1H**^+^, thus enabling modification in water. Ac, acetyl; TT, thianthrene; TFA, trifluoroacetyl group; TM, transition metal; X, functional group (for example, halogenyl, hydroxyl).

We had previously developed C–H thianthrenation chemistry for small molecules that is distinguished by exquisite site- and regioselectivity and by the large functional group tolerance^34^. Combined with the ability to convert the thianthrenium linchpin into a multitude of substituents^34–37^, such an approach would be ideal for small and single-atom peptide and protein modification. However, the reactive intermediate in arene thianthrenation is the aromatic, thianthrene dication, which does not persist sufficiently long in water for productive C–H functionalization in an aqueous environment^38,39^. Functionalization can be induced by acylation of thianthrene *S*-oxide (TT-OTFA^+^) or strong acid in non-aqueous medium but neither can be used for most complex biomolecules (Fig. 1a). With an analogue more basic than thianthrene *S*-oxide for protonation in water, one might be able to benefit from the high chemo- and stereoselectivity of thianthrenation which can distinguish subtle differences in the electronic structure of electron-rich arenes as indicated by a Hammett *ρ* value of −11 (ref. ^39^). However, no sulfoxide that we evaluated reached sufficiently high basicity to achieve productive C–H functionalization for potential protein modification in water (Supplementary Table 1). Therefore, we evaluated sulfur’s heavier homologue selenium due to its higher basicity^40^. What resulted ultimately is a selenoxide-based reagent **1** whose protonated form **1H**^+^ can react productively in water, yet still retain high positional selectivity, so that selective C–H functionalization of tyrosine residues of peptidyl substrates becomes feasible (Fig. 1b). The selenium-based substituent in the obtained tyrosine-based arylselenonium salts can be converted into a variety of other substituents, including single atoms to access simple, well-defined analogues that are challenging to access otherwise. Known selenium chemistry in protein science^41,42^ uses the nucleophilicity of selenols^31,32^ and selenides^33^, and selenoxides are used for disulfide bond formation^43^; this work shows the application of an electrophilic selenium reagent to C–H functionalization of biomolecules. The weak C(*sp*^2^)–Se bond can readily be engaged for further transformations and provides insights into the utilization of heavy main-group elements in the field of bioconjugation.

## Results and discussion

### Design and mechanistic study of selenoxide 1

**Fig. 2:**
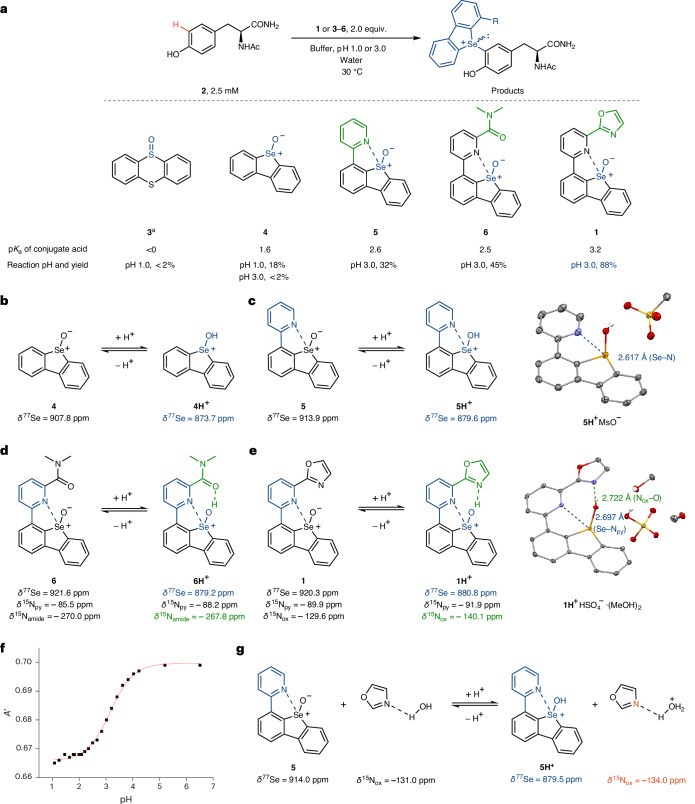
Development of selenoxide reagent. **a**, Reaction yield as a function of p*K*_a_ and pH. Aqueous H_2_SO_4_ used for pH 1.0; sodium citrate buffer used for pH 3.0. Yield determined by ^1^H NMR spectroscopy in triplicate. ^a^MeCN 20% (v/v) as cosolvent. **b**, NMR studies of **4**. **c**, NMR studies of **5** and X-ray structure of **5H**^+^MsO^‒^. **d**, NMR studies of **6**. **e**, NMR studies of **1** and X-ray structure of **1H**^+^HSO_4_^‒^·(MeOH)_2_. **f**, Titration curve of **1**. Absorption (*A*′) measured at 348 nm and corrected for the concentration of **1** during titration (Supplementary Fig. 12). **g**, Control NMR experiment of a mixture of **5** and oxazole in a molar ratio of 1/1. NMR spectroscopy performed in water/MeCN-*d*_3_ of either pH 1 or 13; hydrogen atoms in X-ray structures were found and refined. Ac, acetyl; MeCN, acetonitrile; MsO^‒^, methanesulfonate anion; MeOH, methanol; N_ox_, nitrogen nucleus of oxazolyl groups or oxazoles; N_py_, nitrogen nucleus of pyridyl groups. Source data

A productive functionalization of tyrosine analogue **2** with thianthrene *S*-oxide **3** could not be observed even at pH 1.0 in water due to the insufficient basicity of **3** (Fig. 2a). As a proof of principle, the simple selenoxide **4**, which is more basic than the sulfoxides, could functionalize **2**, although only at an inappropriately low pH of 1 that was necessary to access the selenoxonium **4H**^**+**^, which we anticipate is the reactive electrophile for aromatic functionalization (Fig. 2b and Supplementary Figs. 15 and 16). We sought to design structural modifications of **4** that would increase the p*K*_a_ value of the conjugate acid sufficiently that productive C–H functionalization could proceed efficiently in sodium citrate buffer at pH 3. A proximal Lewis base could act as a chalcogen bond acceptor to increase electron density at selenium^44^ and thereby the basicity of the selenoxide for protonation at higher pH. In addition, the chalcogen bond should induce a low-energy conformation^45^ that we suspected may preorganize the selenoxonium hydroxyl group for potential hydrogen bonding to an intramolecular hydrogen-bond acceptor. The hydrogen bond would further stabilize the selenoxonium cation to elicit electrophilic C–H functionalization at higher pH. Introduction of an appropriately positioned pyridyl substituent in **5** resulted in a higher p*K*_a_ value and is consistent with a Se–N chalcogen bond in **5H**^**+**^. NMR studies are consistent with the existence of the chalcogen bond in aqueous solution; the ^77^Se NMR resonances of **5** and **5H**^**+**^ are shifted by about 6 ppm when compared to **4** and **4H**^**+**^, respectively, which has also been observed for other compounds with chalcogen bonds^46^ (Fig. 2c). Additionally, the intramolecular chalcogen bond in **5H**^**+**^ could be observed in the solid state, where a short Se–N distance of 2.62 Å was observed; the sum of the van der Waals radii is 3.45 Å (ref. ^47^). The chalcogen bond, rather than the electron-withdrawing property of the pyridyl group, is critical for the improved reactivity of **5** at pH 3.0, as indicated by the inertness of an analogue in which the DBSe scaffold is *para* to the pyridyl nitrogen towards **2** under the same conditions (Supplementary Table 1, entry 6). Introduction of an additional substituent appropriately situated to engage in hydrogen bonding with a protonated selenoxide resulted in amide **6**. NMR studies indicate that the amide group of **6H**^**+**^ is hydrogen bonded to a donor more acidic than water, which is possibly the intramolecular selenoxonium group (Fig. 2d); the larger ^15^N NMR resonance shift of the amide group of **6H**^**+**^ compared to **6** (Δ*δ* = 2.2 ppm; Fig. 2d) matches the reported shift values of other amides with hydrogen-bond donor shift from water to more acidic trifluoroethanol (Δ*δ* = 0.8–2.9 ppm)^48^. Additionally, the shifts of ^13^C NMR and ^1^H NMR resonances of the amide group of **6H**^**+**^ compared to **6** are consistent with the existence of an intramolecular hydrogen bond (Supplementary Figs. 22 and 24–26). Nevertheless, the introduction of the hydrogen bond to **6H**^+^ only slightly increased the desired reactivity at about the same acidity, with a p*K*_a_ of **6H**^+^ similar to that of **5H**^+^. Because of the weak basicity of the amide group, the additional stabilization of **6H**^+^ by the hydrogen bond may be modest and further offset by a weaker intramolecular chalcogen bond due to the lower Lewis basicity of the amide-substituted pyridyl group. To combine both advantageous effects, that is, chalcogen and hydrogen bonding, we designed selenoxide **1** with an oxazolyl group instead of the amide in **6**. The oxazolyl group is more basic than the amide group in **6**, which resulted in improved stabilization of **1H**^+^ and thereby the higher p*K*_a_ and productive C–H functionalization in 88% yield at pH 3. Replacement of the oxazolyl group with an even more basic pyridyl group was inefficient for C–H functionalization at pH 3 (Supplementary Table 1, entry 11), perhaps due to protonation of the distant pyridyl group rather than the selenoxide. The crystal structure of **1H**^+^HSO_4_^–^·(MeOH)_2_ with a refined hydrogen atom confirmed the presence of the intramolecular hydrogen bond between the oxazolyl group and the selenoxonium group in the solid state, in addition to the intramolecular chalcogen bond. The smaller ^15^N NMR resonance of the oxazolyl group of **1H**^**+**^ compared to **1** (Δ*δ* = −10.5 ppm; Fig. 2e) in aqueous solution indicates the increased shielding at the nitrogen nucleus of **1H**^+^, explained by the change of the contribution of the *sp*^2^-hybridized nitrogen lone pair to the overall shielding tensor, which is induced by the protonation of or the hydrogen bonding to the nitrogen atom of the oxazolyl group^49^. We attribute the observed reactivity to the combination of both chalcogen and hydrogen bonds; simple activation through a second protonation event of **1H**^+^ at the oxazolyl group at pH 1, in which the protonated oxazolyl group would merely function as an intramolecular Brønsted acid, is not supported by the titration experiment shown in Fig. 2f, which indicates a single protonation event and is consistent with a hydrogen bond that is stable even in water. Similarly, a control experiment with the structural components of **1** but intermolecular, namely an equimolar mixture of oxazole and **5**, which lack the hydrogen-bonding interaction between them as judged by the nearly identical ^77^Se NMR resonances in the NMR study of selenoxide **5** (Fig. 2c) and in the control experiment (Fig. 2g), is consistent with the intramolecular hydrogen bond in **1H**^**+**^. The ^15^N NMR shift of the oxazole at pH 1 in the presence of **5H**^**+**^ decreases only by 3 ppm compared to neutral oxazole (Fig. 2g). The comparison to the change Δ*δ* = −10.5 ppm for **1** (Fig. 2e) suggests that hydrogen bonding between oxazole and hydrated protons is insufficient to explain the observed ^15^N shifts in **1H**^+^ when compared to **1**. We therefore conclude that a water-resistant intramolecular hydrogen bond between the oxazolyl nitrogen and the protonated selenoxonium group can rationalize the higher reactivity of **1H**^+^ in electrophilic substitution in tyrosine residues.

### Chemo- and site-selective bioconjugation

**Fig. 3:**
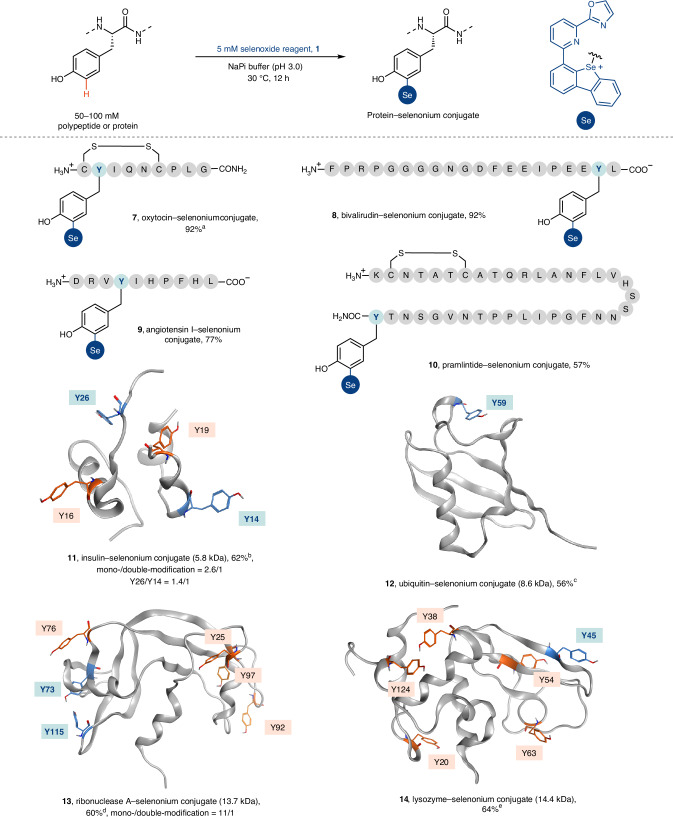
Polypeptide and protein functionalization with 1. Yield determined by UV absorption (*λ*_max_ = 328 nm); yields and ratios of **11** and **13** were determined by UV absorption at 328 nm and deconvoluted zero-charge mass spectra analysis (Supplementary Information, pages 101–102, 113). The numbers in brackets indicate the molecular weight of unmodified proteins. ^a^MgCl_2_ (0.1 M) was used in the reaction. ^b^TFA (100 mM) was used as buffer. The reaction was conducted at 25 °C for 2.5 h to prevent the aggregation of insulin. ^c^MgCl_2_ (50 mM) and urea (2.4 M) were used for denaturation. The reaction was conducted at 37 °C for 12 h. ^d^The reaction was conducted at 37 °C for 16 h; Na_2_SO_4_ (200 mM) was used to stabilize the protein. ^e^The reaction was conducted for 16 h. NaPi, sodium-phosphate buffer; TFA, trifluoroacetic acid; Y, tyrosine residue. Blue background, major modified residue as opposed to red background.

The combination of hydrogen and chalcogen bonds in the electrophilic, protonated form of **1** enabled chemoselective C–H functionalization of peptides under aqueous conditions (Fig. 3). For example, the small, cyclic nonapeptide hormone oxytocin featuring a disulfide bond was modified (**7**, 92%) at pH 3 and 30 °C. Likewise, the anticoagulent drug bivalirudin afforded **8** in 92% yield under the standard conditions. Unlike many selenoxides, which form 4-coordinated selenuranes with carboxylate groups under acidic conditions, selenoxide **1** does not engage in this undesired side reaction with both side-chain and C-terminal carboxylates, perhaps because the dibenzoselenophene scaffold creates a structurally fixed C–Se–C bond angle of 87° (Supplementary Information, page 167), which is smaller than what would be expected in selenuranes (101°)^50^. Angiotensin I and the 37-mer peptidyl drug pramlintide were modified under the standard conditions in 77% and 57% yield, respectively. The compatibility of other residues not covered by the peptidyl substrates (tryptophans, methionines and cysteines) was also studied by utilizing the corresponding amino acids as proxy at pH 3 and 30 °C (Supplementary Information, pages 80–83). Methionines were tolerated (Supplementary Fig. 30) and the electron-rich tryptophans remained untouched, but a slow conversion of **1** to the corresponding selenide was observed in 46% yield, albeit at a rate that did not preclude tyrosine functionalization. Cysteines were quantitatively oxidized to cystines, even at a temperature of 25 °C and within only 30 min. Given the low surface abundance of free cysteines in proteins (<1.9%)^23^, we do not envisage that such side reactivity would generally preclude the practical application of this modification method. Based on the favourable reaction on peptides, we examined the C–H functionalization of the 5.8-kDa peptide hormone insulin, which features four tyrosine residues. All tyrosine residues are located on the protein surface, but in different microenvironments, which results in differences large enough to elicit a site-specific functionalization: residues Y26 on the B chain and Y14 on the A chain are less structurally shielded than Y16 on the B chain and Y19 on the A chain in acidic aqueous solutions (Supplementary Information, pages 105–106) and could be functionalized selectively (Supplementary Figs. 43 and 44 and Supplementary Tables 25–27). Insulin was modified in 62% yield including mono- and double-Se-modified products in a ratio of 2.6:1. Protein recovery was 85% with unmodified insulin as the remaining mass balance (Supplementary Fig. 42c). The 8.5-kDa protein ubiquitin, with a tyrosine residue (Y59) that is not surface-exposed but half-buried by surrounding charged residues (Supplementary Fig. 48) and oxidized methionine residues (Supplementary Fig. 36), remains unreactive toward the selenoxide reagent **1** under the standard conditions. Yet, upon denaturation with urea (2.4 M) at 37 °C, the Y59 was modified in 56% yield with a protein recovery of 84% and the remaining mass balance being unmodified ubiquitin (Supplementary Fig. 47). We could further show the selectivity of the functionalization by studying the transformation of larger proteins with multiple tyrosine residues. For example, ribonuclease A (13.7 kDa, 6Y) afforded **13** in 60% yield with a selectivity for mono- versus double-Se-modified products of 11:1 and Y73 and Y115 as the major modification sites (Supplementary Table 29); four methionine residues remained untouched. Human lysozyme (14.4 kDa, 6Y), which features five tryptophan residues, can be modified in 64% yield under the standard conditions with Y45 as the major modification site (Supplementary Table 30).

### Diversification of tyrosine-selenonium linchpin

**Fig. 4:**
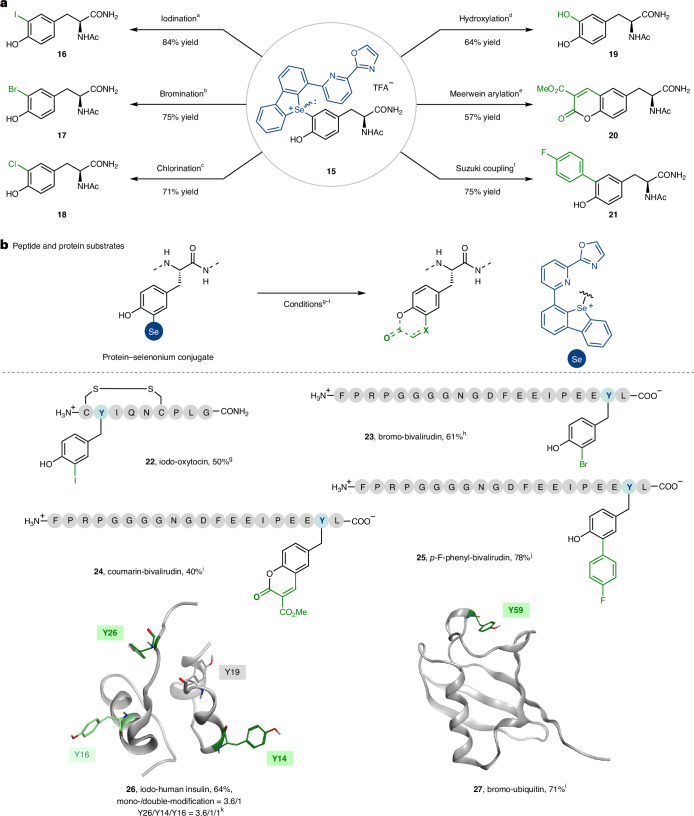
Secondary transformations of tyrosyl-selenonium salts. **a**, Transformations of **15**. Yields were determined by crude ^1^H NMR. ^a^NaI, *i*PrI. ^b^CuBr_2_, Cu(MeCN)_4_BF_4_, KBr. ^c^CuCl_2_, Cu(MeCN)_4_BF_4_, NaCl. ^d^B_2_(OH)_4_, NaF, Hantzsch ester, then H_2_O_2_. ^e^FeSO_4_·7H_2_O, dimethyl 2-(methoxymethylene)malonate, then EDTA-Na_2_ and NaHCO_3_. ^f^Pd(OAc)_2_, (4-fluorophenyl)boronic acid. **b**, Transformations of selenonium bioconjugates. Yields were determined by UV absorption at 214 nm and, if necessary, deconvoluted zero-charge mass spectra. ^g^NaI, *i*PrI. ^h^CuBr_2_, Cu(MeCN)_4_PF_6_, KBr. ^i^FeSO_4_·7H_2_O, dimethyl 2-(methoxymethylene)malonate, then Na-glycine buffer (pH 9.0). ^j^Pd(OAc)_2_, TPPTS, (4-fluorophenyl)boronic acid. ^k^NaI, *i*PrI. ^l^CuBr_2_, Cu(MeCN)_4_PF_6_, KBr. All reactions were conducted in aqueous buffer containing organic cosolvent 10–40% (v/v). Reactions, except (f) and (j), were irradiated by 390-nm LEDs for 10–20 min. *i*Pr, isopropyl; MeCN, acetonitrile; Ac, acetyl; TPPTS, 3,3′,3′′-phosphanetriyltris(benzenesulfonic acid) trisodium salt.

The lowest unoccupied molecular orbital of the tyrosine-based selenonium salt **15** has a large contribution from the antibonding C_tyr_–Se orbital (Supplementary Fig. 74). Due to the relatively small C_tyr_–Se bond dissociation energy of only about 71 kcal mol^−1^ (Supplementary Fig. 73), and the absorption of **15** in the range of 365–465 nm (Supplementary Fig. 70), we anticipated based on our experience with arylthianthrenium salts^51^ that homolytic cleavage of the C_tyr_–Se bond could occur upon irradiation with visible light without the need for a photocatalyst. The resulting synthetically useful aryl radical should be able to participate in various radical reactions, while the selenyl radical cation could be reduced to the selenide by a sacrificial reductant present in solution (Supplementary Fig. 29). Based on this design, we explored the transformations of selenonium salt **15** in buffered aqueous solution (Fig. 4a). All reactions were conducted on 0.32 μmol with a concentration of **15** of 1.6 mM, similar to the required conditions for peptide or protein transformations. Irradiation at 390 nm for only 10 min in the presence of sodium iodide resulted in iodination of **15** with isopropyl iodide to give **16** in 84% yield, determined by ^1^H NMR spectroscopy, presumably via halogen-atom transfer^52^. The selenium scaffold was recovered in a mechanistic probe experiment on a larger scale as the corresponding selenide in 90% yield and the formation of iodine was observed (Supplementary Information, page 69), consistent with the proposed formation of selenyl radicals. In the absence of sodium iodide, full conversion of the selenonium salt **15** was observed but no product was formed (Supplementary Table 15, entry 6), which could result from the oxidative decomposition of tyrosine products by the selenyl radical cation. Bromination and chlorination with Cu(MeCN)_4_BF_4_ as the reductant and CuBr_2_ or CuCl_2_ as the halogen source, respectively, afforded the other arylhalides **17** and **18** as single-atom modifications. Biologically relevant hydroxylation (**19**) can be achieved via borylation followed by the oxidation of the intermediate boronate with hydrogen peroxide. A light-mediated Meerwein arylation with a malonate Michael acceptor followed by immediate ring closure leads to the formation of a fluorescent coumarin group in 57% yield (**20**). All photoreactions proceeded within 10–20 min. Apart from photoredox reactions, the selenonium salt **15** is competent for transition-metal-catalysed cross-couplings due to the low-lying C_tyr_–Se antibonding orbital, as evidenced by the palladium-catalysed Suzuki cross-coupling, which afforded biaryl tyrosinamide **21** in 75% yield within 5 min at 37 °C. The combination of a variety of different photomediated and transition-metal-catalysed reactions demonstrates the synthetic versatility of the selenium substituent. We also conducted those reactions on a larger scale and the yields of the isolated products generally agree well with the yields measured by ^1^H NMR spectroscopy for the reactions conducted on a small scale (Supplementary Information, page 68).

Extension of the reaction chemistry to the selenium-modified tyrosine residues in complex peptides and proteins demonstrates the bio-orthogonal reactivity of the selenonium linchpin (Fig. 4b). For example, single-atom modification of oxytocin afforded the iodinated analogue **22** in 50% yield. Likewise, bromination, coumarin formation and Suzuki cross-coupling reactions were carried out on bivalirudin–selenonium conjugate **8**, and the yields were determined by measuring the absorption at 214 nm using bivalirudin as a reference. Coumarin formation allows the direct post-translational mutation of a tyrosine residue into a fluorescent residue close to the peptide backbone with only a small change of the residue size. Post-translational coumarin introduction may provide the opportunity to study protein solution structure based on probing the distance between different residues via fluorescence resonance energy transfer^53^. Iodo-insulin **26** was obtained in 64% yield including mono- and double-iodinated products with a ratio of 3.6:1. The dominant modification site is the Y26 residue upon iodination, and an additional iodination site Y16 was observed, which could possibly result from the iodination of small amounts of selenium-modified Y16 originating from the previous selenium-modification step (Supplementary Figs. 42–44). The possibility that the iodinated Y16 results from the side reaction between unmodified Y16 and the iodine formed during the reaction is excluded based on the results of the control experiment with unmodified insulin and tyrosine-based selenonium salt **15**, in which iodo-insulin was not observed and unmodified insulin was recovered in 86% yield (Supplementary Fig. 64). Our method provides an alternative synthesis approach to insulin with 3-iodo-Y26 in addition to native chemical ligation^9^. Bromo-ubiquitin **27** was obtained in 71% yield and its secondary structure matches that of the native ubiquitin upon renaturation, as judged by circular dichroism spectroscopy (Supplementary Fig. 68).

## Conclusions

We report the design and utility of a selenoxide that can achieve site-selective single-atom modifications on tyrosine residues. The method represented an approach for C–H functionalization of peptides and begins to tackle C–H functionalization of proteins with small structural changes. Although the limitations imposed by the reactivity and reaction conditions, for example, pH 3, does not yet allow selective functionalization of most proteins, the method introduces a concept that allows for the introduction of a versatile linchpin by selective C–H functionalization of proteins, including single-atom modifications that are currently not readily achieved otherwise.

## Methods

### Initial study on functionalization of compound 2

#### Preparation of stock solutions of sulfoxides

Under ambient atmosphere, a scintillation vial (4 ml) equipped with a Teflon-coated magnetic stir bar was charged with sulfoxide (0.050 mmol) and the solvent MeCN (2.0 mL, 25 mM). The mixture was stirred at 25 °C until all the solids dissolved. The resulting solution was stored at 25 °C and be directly used for reactions without any further operations.

#### Preparation of stock solutions of selenoxides

Under ambient atmosphere, a scintillation vial (4 ml) was charged with selenoxide (0.050 mmol, final concentration 25 mM) and 2.0 ml of an aqueous H_3_PO_4_ solution (50 mM) in ultrahigh-quality (UHQ)-H_2_O. The suspension was heated at 90 °C for 30 min to give a yellow solution. After cooling to 25 °C, the resulting solution was stored in the dark at 25 °C and directly used for reactions without any further operations.

#### Reaction set-up

Under ambient atmosphere, a gas chromatography (GC) vial (2 ml) equipped with a Teflon-coated mini magnetic stir bar was charged with 100 µl of sodium citrate buffer (pH 3.0, 500 mM, final concentration 100 mM) or 100 µl of an H_2_SO_4_ stock solution (250 mM, final concentration 50 mM, final pH 1.0) in UHQ-H_2_O, 250 µl of UHQ-H_2_O and 100 µl of the sulfoxide stock solution (25 mM, 2.5 µmol, 2.0 equiv., final concentration 5.0 mM) in MeCN (final volume percentage 20%) or 100 µl of the selenoxide stock solution (25 mM, 2.5 µmol, 2.0 equiv., final concentration 5.0 mM) in H_3_PO_4_ solution (50 mM) in UHQ-H_2_O. The mixture was stirred at 30 °C for 5 min followed by the addition of 50 µl of a NAc-Tyr-NH_2_ **2** stock solution (25 mM, 1.25 µmol, 0.28 mg, 1.0 equiv., final concentration 2.5 mM) in UHQ-H_2_O. Then the mixture was stirred at 30 °C for 18 h, after which 25 µl of a NaOH stock solution (2.0 M, 50 µmol, 2.0 mg, 40 equiv.) in UHQ-H_2_O was introduced to the reaction in aqueous H_2_SO_4_ solution, and 56 µl of a NaOH stock solution (2.0 M, 0.11 mmol, 4.5 mg, 90 equiv.) in UHQ-H_2_O was introduced to the reaction in sodium citrate buffer. The mixture was then concentrated to dryness under reduced pressure, the residue was dissolved in CD_3_OD (∼0.5 ml) and 5.0 µl of a CH_2_Br_2_ solution (0.50 M, 0.43 mg, 2.5 µmol, 2.0 equiv) in CD_3_OD was added as internal standard. The mixture was analysed by ^1^H NMR spectroscopy and a singlet peak at 5.16 ppm (CH_2_Br_2_) was set as 4.00. The yields of the different selenonium salts were determined as follows:

Yield of the product of **4** = integration of peak (*δ* 6.46 ppm (d, 1H)) × 100%.

Yield of the product of **5** = integration of peak (δ 6.09 ppm (d, 1H)) × 100%.

Yield of the product of **6** = integration of peak (*δ* 6.15 ppm (d, 1H)) × 100%.

Yield of the product of **1** = integration of peak (*δ* 6.11 ppm (d, 1H)) × 100%.

### General procedure for NMR study

Under ambient atmosphere, a GC vial (2 ml) equipped with a Teflon-coated mini magnetic stir bar was charged with selenoxide (0.010 mmol, final concentration 20 mM), UHQ-H_2_O (225 µl) and MeCN-*d*_3_ (250 µl, final volume percentage 50%). For the control experiment in Fig. 2g, the MeCN-*d*_3_ was replaced by a solution of oxazole (40 mM, 250 µl, 0.010 mmol, 0.69 mg, final concentration 20 mM) in MeCN-*d*_3_ (final volume percentage 50%). The pH of the mixture was adjusted by the addition of a solution of H_2_SO_4_ (1.0 M, 25 µl, 25 µmol, 2.5 mg, 2.5 equiv., final concentration 50 mM, pH 1) in UHQ-H_2_O or a solution of NaOH (2.0 M, 25 µl, 50 µmol, 2.0 mg, 5.0 equiv., final concentration 0.10 M, pH 13) in UHQ-H_2_O. The mixture was stirred at 25 °C until all the solids dissolved. The mixtures were analysed by ^1^H NMR, ^13^C NMR, ^77^Se NMR and ^1^H–^15^N HMBC (heteronuclear multiple bond correlation) spectroscopy.

### General protocol for selenium modification of peptides and proteins

#### Preparation of the stock solutions of the selenoxide 1

Under ambient atmosphere, a scintillation vial (4 ml) was charged with the selenoxide **1** (20 mg, 0.050 mmol, final concentration 50 mM) and 1.0 ml of a H_3_PO_4_ solution (0.10 M) in UHQ-H_2_O. The suspension was heated at 90 °C for 30 min to give a yellow solution. After cooling to 25 °C, the resulting solution was stored in the dark at 25 °C and used directly for reactions without any further operations.

#### Preparation of the stock solutions of peptide and protein starting materials

All peptides and proteins were directly used without any further purification. The peptides and proteins were dissolved in UHQ-H_2_O and the solution concentration was determined based on the ultraviolet (UV) absorption at 280 nm measured by Nanodrop (average of three measurements). With the molecular weight (average isotopic mass) and the corresponding extinction coefficient at 280 nm, which are calculated based on the amino acid sequence (https://web.expasy.org/protparam), the concentration of the peptide or protein solution can be calculated as follows:

$$\begin{array}{l}{\rm{Concentration}}\,({\rm{mM}})\\=\displaystyle\frac{{A}_{280}\times 1{,}000}{\text{Extinction coefficient}\,{({\rm{mg}}\,{\rm{ml}}^{-1})}^{-1}\times \text{Molecular weight}\,(\text{g}\,\text{mol}^{-1})}\end{array}$$

The solution was then diluted to a certain concentration (typically, 0.5–2.0 mM) by UHQ-H_2_O and stored at 4 °C.

#### Reaction set-up

At 20–25 °C, NaPi buffer (pH 3.0) or an aqueous solution of TFA (for insulin modification) and the necessary additives depending on the starting material (Fig. 3 and Supplementary Information, pages 89–120) were added to an Eppendorf tube (1.5 ml). The resulting mixture was diluted to 70–85 µl by UHQ-H_2_O followed by the addition of 10 µl of a stock solution of the reagent **1** (50 mM, 0.50 µmol, 50 or 100 equiv., final concentration 5.0 mM) in phosphoric acid solution (0.10 M) in UHQ-H_2_O. The mixture was vortexed for 5 s, transferred into a Thermocycler preheated at 30 °C (or 37 °C) and incubated at 30 °C (or 37 °C) for 30 min at 600 rpm. Next, 5–10 µl of the stock solution of peptide starting material (1.0–2.0 mM, 10 nmol, 1.0 equiv., final concentration 0.10 mM) in UHQ-H_2_O or 10–20 µl of protein starting material (0.25–0.50 mM, 5.0 nmol, 1.0 equiv., final concentration 50 µM) in UHQ-H_2_O was added at 20–25 °C (note that the total volume of the reaction is 100 µl). The mixture was vortexed for 5 s, transferred into a Thermocycler preheated at 30 °C (or 37 °C), and incubated at 30 °C (or 37 °C) for 12–16 h at 600 rpm. Then, excess selenoxide was removed either by reduction with sodium sulfite followed by extraction with ethyl acetate (for example, Supplementary Information, page 89), or by ultrafiltration with Amicon Ultra Centrifugal Filters (Supplementary Information, page 111). The resulting mixture was subjected to liquid chromatography–mass spectrometry (LC–MS) analysis and the yield was determined by UV absorption (*λ*_max_ = 328 nm). For multisite modification (**11** and **13**), yields and ratios were determined by UV absorption at 328 nm and deconvoluted zero-charge mass spectra analysis (Supplementary Information, pages 101–102, 113).

### General protocol for conversions of the selenonium salt 15 by photomediated reactions

#### Preparation of oxygen-free buffers and stock solutions

A scintillation vial (4 ml) was charged with the buffer or the stock solution (2 ml). An argon flow was gently passed through the solution via a needle (0.80 mm diameter × 120 mm) for 3 min. Then, the vial was closed by a screw cap and stored under a nitrogen atmosphere (in a glovebox) for further transformations.

#### Reaction set-up

Under a nitrogen atmosphere (it is recommended to perform these reactions in a glovebox), a GC vial (2 ml) equipped with a Teflon-coated mini magnetic stir bar was charged with the necessary reductants and reagents depending on the transformation (Fig. 4a and Supplementary Information, pages 68–78), followed by the addition of buffer (final concentration 100 mM) in UHQ-H_2_O, a stock solution of selenonium salt **15** (16 mM, 20 µl, 0.32 µmol, 0.23 mg, 1.0 equiv., final concentration 1.6 mM) in MeCN/UHQ-H_2_O (1/1, v/v). The cosolvent MeCN was added to reach a final volume percentage of 20% or 40% (v/v), and UHQ-H_2_O was added to reach the final volume of 200 µl. The vial was closed by a screw cap, placed between two Kessil PR160 390-nm light-emitting diodes (LEDs) (2.5 cm away from each lamp) and irradiated for 10–20 min; the temperature of the reaction mixture was kept at approximately 30 °C through the use of a cooling fan. Then (note the oxidation step of the hydroxylation transformation and the ring-closure step of the coumarin formation; see Supplementary Information, pages 75 and 77, respectively), the mixture was diluted with 800 µl of MeCN/UHQ-H_2_O (1/1, v/v) and saturated with anhydrous Na_2_SO_4_ (∼0.3 g). The mixture was extracted by tetrahydrofuran/ethyl acetate (1/1, v/v, 500 µl × 3) and the organic layers were combined, concentrated under reduced pressure and dried in vacuo. The residue was dissolved in CD_3_OD (∼0.5 ml), and 2.5 µl of a 1,3,5-trimethoxybenzene solution (0.10 M, 0.25 µmol, 42 µg, 0.78 equiv.) in CD_3_OD was added as internal standard. The mixture was analysed by ^1^H NMR and a singlet peak at 6.08 ppm (1,3,5-trimethoxybenzene) was set as 3.00 for the calculation of the product yield.

### General protocol for conversions of selenium-modified peptides and proteins by photomediated reactions

Under a nitrogen atmosphere (it is recommended to perform these reactions in a glovebox) and at 20–25 °C, 10–20 µl of the selenium bioconjugate solution (that is, the product mixture of the corresponding selenium modification step) and the necessary reagents, the additives and the cosolvent MeCN, depending on the transformation (Fig. 4b and Supplementary Information, pages 124–149), were added to an Eppendorf tube (1.5 ml). The concentration of the starting material was controlled in the range of 5–40 µM, and the volume of the reaction was controlled in the range of 45–50 µl. Then, the mixture was vortexed for 5 s (for **11** and **12**, the mixture was vortexed for 5 s and transferred into a Thermocycler preheated at 25 °C and incubated at 25 °C for 5 min at 600 rpm). The Eppendorf tube was floated on an ice-water bath under one Kessil PR160 390-nm LED (2.5 cm away from the LED; Supplementary Fig. 53) and irradiated for 15–20 min. The temperature of the reaction mixture was kept at approximately 0 °C through the use of the ice-water bath. Then, the reaction was worked up according to the transformation (Supplementary Information, pages 124–149), and the resulting mixture was subjected to LC–MS analysis; the yield was determined by UV absorption (*λ*_max_ = 214 nm). For protein modifications (**26** and **27**), yields and ratios were determined by UV absorption at 214 nm and deconvoluted zero-charge mass spectra analysis (Supplementary Information, page 122).

### Protocol for Suzuki coupling of 15 and selenium-modified bivalirudin 8

#### For selenonium salt 15

Under an ambient atmosphere, a scintillation vial (4 ml) equipped with a Teflon-coated mini magnetic stir bar was charged with the coupling partner (4-fluorophenyl)boronic acid (2.8 mg, 20 µmol, 20 equiv.) and the catalyst Pd(OAc)_2_ (1.1 mg, 5.0 µmol, 5.0 equiv.), followed by the addition of NaPi buffer (pH 8.0, 200 mM, 500 µl, final concentration 100 mM) and a stock solution of selenonium salt **15** (25 mM, 40 µl, 1.0 µmol, 0.71 mg, 1.0 equiv., final concentration 1.0 mM) in DMSO/UHQ-H_2_O (1/1, v/v), UHQ-H_2_O (380 µl). The mixture was stirred at 37 °C for 5 min followed by the addition of the cosolvent DMSO (80 µl, final volume percentage 10%). Then the mixture was stirred at 37 °C for 2 h, after which it was diluted with methanol (0.80 ml) and filtered using a 0.22-µm filter. The filtrate was concentrated under reduced pressure and dried in vacuo. The residue was dissolved in CD_3_OD, and 10 µl of a 4-fluorobenzotrifluoride solution (0.10 M, 1.0 µmol, 0.16 mg, 1.0 equiv.) in CD_3_OD was added as internal standard. The mixture was analysed by ^19^F NMR to determine the yield of the product (75%; for details of yield determination, see Supplementary Information, page 78).

#### For selenium bioconjugate 8

(For preparation of the catalyst stock solution, see Supplementary Information, pages 130 and 131.) Under a nitrogen atmosphere (in a glovebox) and at 20–25 °C, 4.0 µl of a *p*-F-phenylboronic acid stock solution (0.20 M, 0.80 µmol, 0.11 mg, 4.7 × 10^2^ equiv., final concentration 19 mM) in DMA and 8.0 µl of the dilution buffer (NaPi buffer, pH 8.0, 0.25 M, containing DMA 25% (v/v)) were added to an Eppendorf tube (1.5 ml). Then, 2.0 µl of a NaOH stock solution (1.5 M, 3.0 µmol, 0.12 mg, final concentration 0.11 M) in UHQ-H_2_O was added for the neutralization of the bivalirudin–selenonium conjugate **8** stock solution added in the next step. The mixture was vortexed for 5 s, followed by the addition of 20 µl of a bivalirudin–selenonium conjugate **8** stock solution (84 µM, 1.7 nmol, 4.3 µg, 1.0 equiv., final concentration 40 µM) in NaPi buffer (pH 3.0, 0.20 M). Next, 8.0 µl of the stock solution of palladium catalyst (Pd(OAc)_2_, 13 mM, 0.10 µmol, 22 µg, 59 equiv., final concentration 2.4 mM; TPPTS, 25 mM, 0.20 µmol, 0.11 mg, 1.2 × 10^2^ equiv., final concentration 4.8 mM) in the dilution buffer was added to finish the preparation of the reaction (the final concentration of NaPi buffer was 0.19 M, the final volume percentage of DMA was 19%, and the final solution pH was 8–9, as indicated by general pH test paper). The mixture was vortexed for 5 s again, transferred into a Thermocycler preheated at 37 °C, and incubated at 37 °C for 40 min at 600 rpm. Then, 40 µl of a sodium diethyldithiocarbamate trihydrate (DTC) stock solution (10 mM, 0.40 µmol, 90 µg, 4.0 equiv. to Pd(OAc)_2_) in UHQ-H_2_O was added and the mixture was vortexed for 5 s, transferred into a Thermocycler preheated at 25 °C, and incubated at 25 °C for 10 min at 600 rpm. After that, the mixture was centrifuged at 4 °C for 10 min at 21,694*g*. The pellets were discarded and the supernatant was stored at −20 °C, and was subjected to LC–MS analysis. The yield was determined by UV absorption (*λ*_max_ = 214 nm) (78%, average of three experiments).

### Crystallization of selenoxides

**1H**^+^HSO_4_^‒^·(MeOH)_2_ (CCDC 2291793): the crystal structure of **1H**^**+**^HSO_4_^‒^·(MeOH)_2_ was obtained as follows. First, **1** (∼3 mg) was dissolved in a solution of H_2_SO_4_ (0.2 M) in methanol (∼0.1 ml) to afford a bright-yellow solution at 25 °C. Then, the solution was slowly added to an NMR tube filled with toluene (∼0.5 mL), and left standing in the dark at 4 °C for 24 h.

**5H**^+^MsO^‒^ (CCDC 2304770): the crystal structure of **5H**^+^MsO^‒^ was obtained as follows. First, **5** (∼2 mg) was dissolved in a solution of methanesulfonic acid (0.5 M) in methanol (30 µl) at 25 °C in a GC vial (2 ml) to afford a pale yellow solution. Then, the solution was diluted with toluene (60 µl), followed by the slow addition of hexanes (∼0.4 ml) above the solution. (Note that it is important not to shake the vial during/after hexane addition.) The mixture was left standing in the dark at 25 °C for 24 h.

## Online content

Any methods, additional references, Nature Portfolio reporting summaries, source data, extended data, supplementary information, acknowledgements, peer review information; details of author contributions and competing interests; and statements of data and code availability are available at 10.1038/s41557-025-01842-8.

## Supplementary information

Supplementary Information Supplementary Figs. 1–76, Tables 1–41, experimental procedures, DFT calculation data, X-ray crystallographic analysis and product characterization.

Supplementary Data 1 Crystallographic data for compound **1H**^**+**^HSO_4_^–^·(MeOH)_2_; CCDC reference 2291793.

Supplementary Data 2 Crystallographic data for compound for **5H**^**+**^MsO^–^; CCDC reference 2304770.

Supplementary Data 3 Numerical source data for Supplementary Figs. 32, 33, 35 and 37.

Supplementary Data 4 Numerical source data for Supplementary Fig. 70.

## Source data

Source Data Fig. 2 Statistical source data for Fig. 2f.

## Data Availability

The data reported in this paper are available within the article and its Supplementary Information. Source data for the calibration curves of peptidyl substrates and the UV–visible absorption spectrum of **15** are provided with this paper. Crystallographic data for the structure reported in this article have been deposited at the Cambridge Crystallographic Data Centre (CCDC) under deposition numbers CCDC 2291793 (**1H**^+^HSO_4_^‒^·(MeOH)_2_) and 2304770 (**5H**^+^MsO^‒^). Copies of the data can be obtained free of charge via https://www.ccdc.cam.ac.uk/structures/. Source data are provided with this paper.
