# Supplementary material for: A selenoxide for single-atom protein modification of tyrosine residues enabled by water-resistant chalcogen and hydrogen bonding
Source: Nat Chem. 2025 Jun 4;17(9):1331–9. doi: 10.1038/s41557-025-01842-8 (PMC12411275; doi:10.1038/s41557-025-01842-8)
Supplement: Supplementary file 1 — Supplementary Figs. 1–76, Tables 1–41, experimental procedures, DFT calculation data, X-ray crystallographic analysis and product characterization. [file 41557_2025_1842_MOESM1_ESM.pdf]

nature chemistry

Supplementary information

https://doi.org/10.1038/s41557-025-01842-8

A selenoxide for single-atom protein modification of tyrosine residues enabled by water-resistant chalcogen and hydrogen bonding

In the format provided by the authors and unedited

TABLE OF CONTENTS

TABLE OF CONTENTS 1

MATERIALS AND METHODS..10

Starting materials and reagents 10

Solvents...11

Chromatography..12

NMR spectroscopy..12

Mass spectrometry..12

Liquid chromatography-mass spectrometry (LC-MS) 12

Miscellaneous..14

EXPERIMENTAL DATA 16

Synthesis of selenoxides 16

Preparation of DBSe (**S1**) 16

Preparation of DBSeO (**4**) 16

Preparation of DBSe-boronic acid **S2** 17

Preparation of 2-(DBSe)-Py **S3**...18

Preparation of 2-(DBSeO)-Py **5** 18

Preparation of 4-(DBSe)-Py **S4**...19

Preparation of 4-(DBSeO)-Py **S5**...20

Preparation of 6-bromo-*N*,*N*-dimethylpicolinamide (**S6**) 20

Preparation of DBSePy-amide **S7**...21

Preparation of DBSeOPy-amide **6** 22

Preparation of 2-(6-bromopyridin-2-yl)oxazole (**S8**)...22

Preparation of DBSePy-oxa **S9**...23

Preparation of DBSeOPy-oxa **1** 24

Preparation of DBSePy-py **S10**...25

Preparation of DBSeOPy-py **S11**...25

Synthesis of tyrosinamide chalcogenonium salts 27

Preparation of NAc-Tyr-NH_2 derived thianthreniumsalt **S12** 27

Preparation of NAc-Tyr-NH_2 derived DBSe-selenonium salt **S13**......................28

Preparation of NAc-Tyr-NH_2 derived 2-(DBSe)-Py-selenonium salt **S14** 29

Preparation of NAc-Tyr-NH_2 derived DBSePy-amide-selenonium salt **S15** 30

Preparation of NAc-Tyr-NH_2 derived DBSePy-oxa-selenonium salt **15**................31

Initial study on functionalization of model compound **2**.................................. 33

Results of model compound **2** functionalization by sulfoxides and selenoxides 33

Solubility of **1** in water at different pH...34

Titration study of selenoxides by UV-Vis spectrometry 35
Mathematical fundamental for pK_a calculation from titration study 35
General consideration and experiment design 36
Titration curve of 50 mM Na-citrate buffer (pH = 6.0) 37
General procedure for titration in cuvette and UV-vis spectrometry 39
Titration study of selenoxide **4** 39
First measurement: 39
Second measurement: 40
Third measurement: 41
Titration study of selenoxide **5** 44
First measurement: 44
Second measurement: 45
Third measurement: 46
Titration study of selenoxide **6** 48
First measurement: 48
Second measurement: 49
Third measurement: 50
Titration study of selenoxide **1** 52
First measurement: 52
Second measurement: 53
Third measurement: 54
NMR study of selenoxides in aqueous solutions 56
NMR study of DBSeO (**4**) and DBSeOH$^+$ (**4H$^+$**) 56
NMR study of 2-(DBSeO)-Py **5** and 2-(DBSeOH)-Py$^+$ **5H$^+$** 57
NMR study of DBSeOPy-amide **6** and DBSeOHPy-amide$^+$ **6H$^+$** 60
NMR study of DBSeOPy-oxa **1** and DBSeOHPy-oxa$^+$ **1H$^+$** 64
Study on transformations of NAc-Tyr-NH_2 derived DBSePy-oxa-selenonium salt **15** 67
Reaction design 67
General considerations on reaction scale and concentration 67
Iodination of the selenonium salt **15** 68
Optimization on a small scale 68
Preparation of NAc-3-iodo-tyrosinamide **16** 69
Bromination of the selenonium salt **15** 70
Optimization on a small scale 70
Preparation of NAc-3-bromo-tyrosinamide **17** 71
Chlorination of the selenonium salt **15** 72
Optimization on a small scale 72

Preparation of NAc-3-chloro-tyrosinamide **18** 73

Hydroxylation of the selenonium salt **15** 73

Optimization on a small scale 73

Preparation of NAc-3-hydroxyl-tyrosinamide **19** 75

Coumarin formation of the selenonium salt **15** 76

Optimization on a small scale 76

Preparation of the coumarin amino acid **20** 77

Suzuki coupling of the selenonium salt **15** 78

Suzuki coupling on a small scale 78

Preparation of the NAc-3-(*p*F-phenyl)-Tyr-NH_2 **21** 79

Protein residue tolerance study of DBSeOPy-oxa **1** 80

Summary of residue tolerance study of DBSeOPy-oxa **1** 80

Reaction between *N*-acetyl-L-cysteine-methylester and **1** 80

Reaction between *N*-acetyl-L-methionine-methylester and **1** 81

Reaction between *N*-acetyl-L-tryptophan-methylester and **1** 82

Synthesis of *Se*-modified peptides and proteins 84

Quantification of *Se*-modified peptides and proteins 84

Data on protein starting materials 84

Oxytocin 84

Bivalirudin 85

Angiotensin I 85

Pramlintide 85

Human insulin 85

Bovine ubiquitin (from erythrocytes) 86

Ribonuclease A 88

Human lysozyme 88

Preparation of oxytocin-selenonium conjugate **7** 89

Preparation in a low concentration (100 µM) 89

Sample preparation of **7** for HRMS/MS analysis 90

HRMS/MS results 91

Preparation of bivalirudin-selenonium conjugate **8** 92

Assessment of side-reactivity 93

HRMS/MS results 94

Preparation of angiotensin I-selenonium conjugate **9** 95

LC-FTMS/MS results 96

Preparation of pramlintide-selenonium conjugate **10** 97

LC-FTMS/MS results 98

Preparation of insulin-selenonium conjugate **11** 100

Yield determination 101

Site-selectivity determination 102

Analysis of the site-selectivity of the *Se*-modification of insulin 105

Preparation of ubiquitin-selenonium conjugate **12** 107

Oxidation of methionines 109

LC-FTMS/MS sample preparation 109

MS/MS results 109

Microenvironment of Y59 110

Preparation of ribonuclease A-selenonium conjugate **13** 111

Yield determination 113

Site-selectivity determination 113

MS/MS results 114

Preparation of lysozyme-selenonium conjugate **14** 117

Yield determination 117

Site-selectivity determination 119

MS/MS results 119

Transformations of *Se*-modified peptides and proteins 121

General considerations for transformations of *Se*-modified peptides and proteins 121

Photoreaction set-up 121

Quantification for transformations of *Se*-modified bivalirudin and *Se*-modified proteins 122

Preparation of iodo-oxytocin **22** 123

Preparation of bromo-bivalirudin **23** 124

LC/Q-TOF-MS/MS results of peak 4 (bromo-bivalirudin **23**) 126

Preparation of coumarin-bivalirudin **24** 127

LC/Q-TOF-MS/MS results of peak 5 (coumarin-bivalirudin **24**) 129

Preparation of *p*-F-phenyl-bivalirudin **25** 130

LC/Q-TOF-MS/MS results of peak 3 (*p*-F-phenyl-bivalirudin **25**) 131

Preparation of iodo-insulin **26** 133

Yield determination 135

Site-selectivity determination 135

Control experiment 140

Preparation of bromo-ubiquitin **27** 141

Preparation of bromo-ubiquitin **27** on a small scale 141

Preparation of bromo-ubiquitin **27** on a large scale (renatured) for LC-FTMS/MS analysis and CD spectrometry 142

Sample preparation for LC-FTMS/MS analysis 145

CD spectroscopy study 146

Control experiment 148

UV-vis spectrometry study of NAc-Tyr-NH_2 derived DBSePy-oxa-selenonium salt **15** 150

DFT calculation 151

Methods 151

Summary of calculation results 151

Dipole moment of **1** in water 151

Equilibrium between **$5H^+_{Se}$** and **$5H^+_{py}$** in aqueous solution 151

Dihedral torsions between amide and pyridyl planes in **$6H^+$** and **6** 151

Bond dissociation energy (BDE) and frontier molecular orbitals of Tyr-selenonium cation 152

Cartesian coordinates of the optimized species 152

1 152

$5H^+_{Se}$ 153

$5H^+_{py}$ 154

$6H^+$ 155

6 156

Tyr-selenonium cation 157

Tyr radical 159

DBSePy-oxa radical cation 159

X-Ray Crystallographic Data 161

Crystal data of **$5H^+$**MsO^- (CCDC 2304770) 161

Crystal data and structure refinement 161

Bond lengths [Å] and angles [°]. 163

Crystal data of **$1H^+$**HSO_4^-·$(MeOH)_2$ (CCDC 2291793) 165

Crystal data and structure refinement 165

Bond lengths [Å] and angles [°]. 167

NMR DATA 169

^{1}H NMR of **S1** 169

^{13}C NMR of **S1** 170

^{77}Se NMR of **S1** 171

^{1}H NMR of **4** 172

^{13}C NMR of **4** 173

^{77}Se NMR of **4** 174

^{1}H NMR of **S2** 175

^{13}C NMR of **S2** 176

^{77}Se NMR of **S2** 177

^{1}H NMR of **S3** 178

^{13}C NMR of **S3** 179

^{77}Se NMR of **S3** 180

^{1}H NMR of **5** 181

^{13}C NMR of **5** 182

^{77}Se NMR of **5** 183

^{1}H NMR of **S4** 184

^{13}C NMR of **S4** 185

^{77}Se NMR of **S4** 186

^{1}H NMR of **S5** 187

^{13}C NMR of **S5** 188

^{77}Se NMR of **S5** 189

^{1}H NMR of **S6** 190

^{13}C NMR of **S6** 191

^{1}H NMR of **S7** 192

^{13}C NMR of **S7** 193

^{77}Se NMR of **S7** 194

^{1}H NMR of **6** 195

^{13}C NMR of **6** 196

^{77}Se NMR of **6** 197

^{1}H NMR of **S8** 198

^{13}C NMR of **S8** 199

^{1}H NMR of **S9** 200

^{13}C NMR of **S9** 201

^{77}Se NMR of **S9** 202

^{1}H NMR of **1** 203

^{13}C NMR of **1** 204

^{77}Se NMR of **1** 205

^{1}H NMR of **S10** 206

^{13}C NMR of **S10** 207

^{77}Se NMR of **S10** 208

^{1}H NMR of **S11** 209

^{13}C NMR of **S11** 210

^{77}Se NMR of **S11** 211

^{1}H NMR of **S12** 212

^{13}C NMR of **S12** 213

^{19}F NMR of **S12** 214

^{1}H NMR of **S13** 215

^{13}C NMR of **S13** 216

^{19}F NMR of **S13** 217

^{77}Se NMR of **S13** 218

^{1}H NMR of **S14** 219

^{13}C NMR of **S14** 220

^{19}F NMR of **S14** 221

^{77}Se NMR of **S14** 222

^{1}H NMR of **S15** 223

^{13}C NMR of **S15** 224

^{19}F NMR of **S15** 225

^{77}Se NMR of **S15** 226

^{1}H NMR of **15** 227

^{13}C NMR of **15** 228

^{19}F NMR of **15** 229

^{77}Se NMR of **15** 230

^{1}H NMR of **S16** 231

^{13}C NMR of **S16** 232
^{1}H NMR of **16** 233
^{13}C NMR of **16** 234
^{1}H NMR of **17** 235
^{13}C NMR of **17** 236
^{1}H NMR of **18** 237
^{13}C NMR of **18** 238
^{1}H NMR of **19** 239
^{13}C NMR of **19** 240
^{1}H NMR of **20** 241
^{13}C NMR of **20** 242
^{1}H NMR of **21** 243
^{13}C NMR of **21** 244
^{19}F NMR of **21** 245
NMR signal assignment of **22** 246
^{1}H NMR of **22** 249
^{13}C NMR of **22** 250
^{19}F NMR of **22** 251
^{1}H-^{13}C HSQC NMR of **22** 252
^{1}H-^{13}C HMBC NMR of **22** 253
COSY NMR of **22** 254
NOESY NMR of **22** 255
ROESY NMR of **22** 256

MS AND MS/MS DATA 257
Oxytocin-selenonium conjugate **7** 257
Bivalirudin-selenonium conjugate **8** 263
Angiotensin I-selenonium conjugate **9** 268
Pramlintide-selenonium conjugate **10** 270
Insulin-selenonium conjugate **11** 274
HRMS 274
LC/Q-TOF-MS and MS/MS for **11** after trypsin digestion and reduction 276
Ubiquitin-selenonium conjugate **12** 280

LC-FTMS of **12** after trypsin digestion 280
MS/MS for the peptide 11 281

Ribonuclease A-selenonium conjugate **13** 282

HRMS 282
LC-FTMS of **13** after trypsin digestion 283
MS/MS for the peptide 14 286
MS/MS for the peptide 15 288
MS/MS for the peptide 17 290
MS/MS for the peptide 18 292
MS/MS for the peptide 20 294
MS/MS for the peptide 22 296
MS/MS for the peptide 23 298
MS/MS for the peptide 26 300
MS/MS for the peptide 27 302
MS/MS for the peptide 28 304
MS/MS for the peptide 29 306

Human lysozyme-selenonium conjugate **14** 308

HRMS 308
LC-FTMS of **14** after trypsin digestion 309
MS/MS for the peptide 13 311
MS/MS for the peptide 15 313
MS/MS for the peptide 19 315
MS/MS for the peptide 22 317
MS/MS for the peptide 27 319

Bromo-bivalirudin **23** 321

Coumarin-bivalirudin **24** 324

p-F-phenyl-bivalirudin **25** 326

Iodo-insulin **26** 328

HRMS 328
LC/Q-TOF-MS and MS/MS for **26** after trypsin digestion and reduction 330

Bromo-ubiquitin **27** 335

LC-FTMS of **27** after trypsin digestion 335
MS/MS for the peptide 16 336

REFERENCES 337

MATERIALS AND METHODS

Starting materials and reagents

All chemicals were used as received from the commercial suppliers:

Material	Vendor	Purity
1,3,5-Trimethoxybenzene	Sigma-Aldrich	≥99%
2-Biphenylboronic acid	BLD	98%
2-Bromopyridine	Alfa Aesar	99%
2,6-Dibromopyridine	Combi-blocks	99%
2-Iodopropane	Sigma-Aldrich	99%
4-Bromopyridine hydrochloride	Alfa Aesar	99%
4-Fluorophenylboronic acid	abcr	98%
6-Bromo-2,2'-bipyridine	abcr	98%
Acetic acid	J.T.Baker	99%
Ac-Tyr-NH_2 (*N*-acetyl-L-tyrosinamide)	abcr	95%
Ammonium bicarbonate	Sigma-Aldrich	≥99.5%
Angiotensin I (human acetate salt hydrate)	Sigma-Aldrich	≥90%
Ascorbic acid	Apollo Scientific	>99.5%
Bis(pinacolato)diboron	BLD	98%
Bivalirudin trifluoracetate salt	Sigma-Aldrich	>97%
Citric acid	Sigma-Aldrich	>99.5%
Copper chloride	Apollo Scientific	99.9%
Copper bromide	Sigma-Aldrich	99%
Diethyl 1,4-dihydro-2,6-dimethyl-3,5-pyridinedicarboxylate (Hantzsch ester)	Chempur	>95%
Dibenzothiophene	Alfa Aesar	98%
Dibromomethane	Sigma-Aldrich	99%
Dimethylamine (2 M in THF)	TCI	n/a
Dimethyl methoxymethylenemalonate	Thermo Fisher	>98%
DTT (Dithiothreitol)	Chempur	>99%
EDTA (Ethylenediaminetetraacetic acid)	Sigma-Aldrich	99.4–100.6%
Ethyl dibromofluoroacetate	TCI	>97%
Glycine	Sigma-Aldrich	>99%
Hexafluoroisopropanol	Fluorochem	>99.5%
Human lysozyme	Sigma-Aldrich	>90%
Hydrochloride (36–38 wt.%)	J.T.Baker	n/a
Hydrogen peroxide (35 wt.%)	Acros	n/a
Insulin (yeast recombinant)	Sigma-Aldrich	>98%
Iron(II)-sulfate-heptahydrate	Sigma-Aldrich	>99%
L-Ascorbic acid sodium salt	Alfa Aesar	99%
Magnesium chloride	Sigma-Aldrich	>98%
MES hydrate (2-(N-morpholino)ethanesulfonic acid)	Apollo Scientific	>99.5%
Methanesulfonic acid	Acros	>99%
Methyl-6-brompyridine-2-carboxylate	TCI	>98%
Methyl *N*-acetyl-L-tryptophanate	BLD	98%
N-Acetyl-L-cysteine methyl ester	Sigma-Aldrich	≥90%
N-Acetyl-L-methionine	Alfa Aesar	99%
n-Butyllithium (2.5 M in hexane)	Sigma-Aldrich	n/a

Oxazole	Apollo Scientific	95%
Oxytocin	BOC Sciences	>98%
Palladium acetate	from our institute	98%
Peracetic acid (38–40 wt.%)	Sigma-Aldrich	n/a
Phosphoric acid (aqueous solution, 85 wt.%)	Alfa Aesar	n/a
Potassium bromide	from our institute	99%
Pramlintide (acetate salt)	Sigma-Aldrich	≥95%
Ribonuclease A (from bovine pancreas, Type III-A)	Sigma-Aldrich	≥85%
SDS (Sodium dodecyl sulfate)	abcr	99%
Selenium	Thermo Fisher	>99%
Sodium acetate	Sigma-Aldrich	>99%
Sodium bicarbonate	Acros	99%
Sodium carbonate	Acros	99%
Sodium chloride	Chemsolute	99%
Sodium diethyldithiocarbamate trihydrate (30.5–32.5 wt.%, Na as Na_2SO_4)	Sigma-Aldrich	n/a
Sodium fluoride	Sigma-Aldrich	>99%
Sodium hydroxide	VWR	>98.5%
Sodium iodide	Sigma-Aldrich	>99%
Sodium phosphate monobasic monohydrate	Sigma-Aldrich	98%
Sodium sulfate (anhydrous)	VWR	98.5–101.0%
Sodium sulfite	Acros	>98%
Sodium trichloroacetate	Sigma-Aldrich	97%
Sulfuric acid (95–98 wt.%)	J.T.Baker	n/a
Tetrafluoroboric acid diethyl ether complex (51–57 wt.% HBF_4)	Sigma-Aldrich	n/a
Tetrahydroxydiboron	abcr	95%
Tetrakis-(acetonitrile)-copper(I)-hexafluorophosphate	abcr	98%
Tetrakis-(acetonitrile)-copper(I)-tetrafluoroborate	abcr	98%
Tetrakis-(triphenylphosphine)-palladium(0)	Sigma-Aldrich	99%
Thianthrene	BLD	98%
TPPTS (Triphenylphosphine-3,3′,3″-trisulfonic acid trisodium salt)	Sigma-Aldrich	≥95%
Tribromoacetic acid	TCI	>98%
Trifluoroacetic acid	abcr	99%
Trifluoroacetic anhydride	abcr	99.5%
Trimethylborate	Sigma-Aldrich	98%
Trimethylsilylcyanid	Sigma-Aldrich	98%
Tris-(hydroxymethyl)-methylamine	Alfa Aesar	>99%
Tri-tert.-butylphosphonium-tetrafluoroborat	abcr	99%
Ubiquitin (from bovine erythrocytes)	Sigma-Aldrich	≥98%
Urea	from our institute	n/a
Zinc chloride	TCI	>98%

Thianthrene *S*-oxide[1], dibenzothiophene *S*-oxide[2], and *N*-acetyl-L-methionine-methylester[3] were synthesized according to literature reports.

Solvents

Water used to prepare buffers and as solvent was of ultra-high quality (UHQ) grade (18.2 MΩ·cm^{-1}). Methanol (>99%) was purchased from Sigma-Aldrich, dichloromethane (>99%) and acetonitrile (>99%)

were purchased from Fisher Scientific, DMA was purchased from Acros, DMSO was purchased from ChemSolute and ethanol (≥99.8%) was purchased from Honeywell. All solvents were used as received. Anhydrous solvents were obtained from Phoenix Solvent Drying Systems. All deuterated solvents were purchased from Euriso-Top®.

Chromatography

Thin layer chromatography (TLC) was performed using EMD TLC plates pre-coated with 250 µm thickness silica gel 60 F254 plates and visualized by fluorescence quenching under UV light and $KMnO_4$ stain. Flash column chromatography was performed using silica gel (40–63 µm particle size) purchased from Geduran®.

NMR spectroscopy

Chemical shifts are reported in ppm with the solvent residual peak as the internal standard. For ^{1}H NMR: $CDCl_3$, δ 7.26; CD_3OD, δ 3.31; $(CD_3)_2SO$, δ 2.50; CD_3CN, δ 1.94; DMF-d_7, δ 2.75. For ^{13}C NMR: $CDCl_3$, δ 77.2; CD_3OD, δ 49.0; $(CD_3)_2SO$, δ 39.5; CD_3CN, δ 1.3; DMF-d_7, δ 34.9[4]. ^{15}N, ^{19}F, and ^{77}Se chemical shifts were referenced indirectly to the residual ^{1}H chemical shift of the solvent according to IUPAC recommendations using the xiref macro in Bruker Topspin[5].^{15}N chemical shifts are reported relative to CH_3NO_2 (δ = 0 ppm; Ξ = 10.136767%), ^{19}F chemical shifts are reported relative to CCl_3F (δ = 0 ppm; Ξ = 94.094011%), and ^{77}Se chemical shifts are reported relative to Me_2Se (δ = 0 ppm; Ξ = 19.071513%). Data are reported as follows: s = singlet, d = doublet, t = triplet, q = quartet, quint = quintet, m = multiplet, br = broad; coupling constants in Hz. ^{15}N chemical shifts were generally extracted from the indirect dimension of a ^{1}H-^{15}N HMBC spectrum optimized to a long-range coupling J_{HN} 5–7 Hz. 1D ^{77}Se NMR spectra were typically acquired at 95 MHz with a single pulse-acquire sequence or at 115 MHz with a refocused INEPT sequence with a magnetization transfer delay optimized to J_{HSe} = 8–10 Hz..

NMR spectra were recorded on the following instruments:

1. Bruker Avance III 500 spectrometer equipped with a BBFO probe head, operating at 500 MHz, 471 MHz, 95 MHz, and 126 MHz, for ^{1}H, ^{19}F, ^{77}Se, and ^{13}C acquisitions, respectively.
2. Bruker AVANCE Neo 600 MHz NMR spectrometer equipped with a BBO cryogenic probehead (Bruker GmbH, Rheinstetten) operating at 600 MHz, 61 MHz, 565 MHz, 115 MHz, and 151 MHz, for ^{1}H, ^{15}N, ^{19}F, ^{77}Se, and ^{13}C acquisitions, respectively. All experiments used standard Bruker pulse sequences of Topspin 4.0.6.

Mass spectrometry

High resolution Mass Spectrometry (HRMS) experiments for small molecules and peptides were performed on a Thermo Scientific™ Q Exactive Plus or a Thermo Scientific™ Q Exactive GC Orbitrap device.

Liquid chromatography-mass spectrometry (LC-MS)

Analytical LC-MS measurements of peptides and proteins were performed on the following instruments:

1. Shimadzu LCMS-2020
2. Shimadzu LCMS-9030 Q-TOF

The utilized methods are described for each performed analysis.

Method A: Analytical LC-MS measurements of peptides and proteins were performed on the following instruments:

Measurements were performed on a Shimadzu LCMS-2020 with SCL-40, LC-40D XS, SIL-40C XS, CTO-40C, SPD-M40, equipped with an Agilent Zorbax 300SB-C3 column, 150 mm × 4.6 mm, 5 µm. The following HPLC method was used for all protein measurements with this setup: linear gradient from 10:90 v/v (0.1% v/v formic acid in MeCN : 0.1% v/v formic acid in H_2O) to 80:20 v/v (0.1% v/v formic acid in MeCN : 0.1% v/v formic acid in H_2O) over 10 minutes, followed by linear gradient from 80:20 v/v (0.1% v/v formic acid in MeCN : 0.1% v/v formic acid in H_2O) back to 10:90 v/v (0.1% v/v formic acid in MeCN : 0.1% v/v formic acid in H_2O) over 3 minutes at a flow rate of 0.5 mL/min. The column was constantly kept at 50 °C. Absorption spectra were recorded at 214 nm and 328 nm (for selenonium conjugates) (DAD).

Analytical LC/Q-TOF-MS/MS measurements of proteins were performed on following instruments:

Measurements were performed on a Shimadzu LCMS-9030 Q-TOF with SCL-40, 2 × LC-40D XS, SIL-40C XS, CTO-40C, SPD-M40A, equipped with:

1. *Method B (for modified bivalirudin **19–21**):* Eclipse Plus C18 column, 50 mm × 3.0 mm, 1.8 µm; linear gradient from 10:90 (0.1% formic acid in MeCN : 0.1% formic acid in H_2O, v/v) to 40:60 (0.1% formic acid in MeCN : 0.1% formic acid in H_2O, v/v) over 10 minutes (15 minutes for coumarin-bivalirudin **20**), followed by isocratic run with 70:30 (0.1% formic acid in MeCN : 0.1% formic acid in H_2O, v/v) over 5 minutes at a flow-rate of 0.5 mL/min (0.3 mL/min for coumarin-bivalirudin **20**). The column was constantly kept at 40 °C.
2. *Method C (for proteins):* linear gradient from 20:80 (0.1% formic acid in MeCN : 0.1% formic acid in H_2O, v/v) to 70:30 (0.1% formic acid in MeCN : 0.1% formic acid in H_2O, v/v) over 15 minutes, followed by isocratic run with 70:30 (0.1% formic acid in MeCN : 0.1% formic acid in H_2O, v/v) over 5 minutes at a flow-rate of 0.2 mL/min. The column was constantly kept at 50 °C. The column used for different proteins are below:
   a. YMC-Triart Bio-C4 column, 100 × 2.1 mm, 1.9 µm (insulin, ubiquitin, and iodo-insulin **22**)
   b. YMC-Triart Bio-C4 column, 150 × 2.1 mm, 1.9 µm (ubiquitin-selenonium conjugate **10**)
   c. YMC Bio-C4 column, 100 × 2.1 mm, 1.9 µm (bromo-ubiquitin **23**)
3. *Method D (for MS/MS analysis of Se-insulin **9** and I-insulin **22**):* YMC-Triart Bio-C4 column, 100 × 2.1 mm, 1.9 µm; linear gradient from 20:80 (0.1% formic acid in MeCN : 0.1% formic acid in H_2O, v/v) to 50:50 (0.1% formic acid in MeCN : 0.1% formic acid in H_2O, v/v) over 15 minutes, followed by isocratic run with 50:50 (0.1% formic acid in MeCN : 0.1% formic acid in H_2O, v/v) over 5 minutes at a flow-rate of 0.2 mL/min. The column was constantly kept at 50 °C.

Semi-preparative HPLC separations of small molecules were performed on an Agilent prep system with a 1260 Infinity II prep pump (G7161A), a 1260 Infinity II prep autosampler (G7157A) with a 900 µL sample loop, a 1260 Infinity II Diode Array Detector (G7115A) with a 3 mm cell and two clustered fraction collectors 1260 Infinity II Preparative Valve-Based Fraction Collector (G7166A) and Agilent 1290 Infinity II Preparative Open-Bed Fraction Collector G7159B. No oven was used.

Preparative HPLC separations of peptides were performed on a Shimadzu system using 2x LC-20AP,

SIL-20A HT with 2 mL sample coil, CTO-20AC, SPD-20A variable cell 0.5 mm, CBM-20A, and FRC-10A modules.

LC-FTMS/MS analysis was performed by using either a Thermo Scientific™ Orbitrap Elite™ equipped with a nanospray source with 30 µm PicoTip emitters, or a Thermo Scientific™ Orbitrap Eclipse™ equipped with a Nanospray source using metal needle emitters. Mass spectra were acquired by data-dependent acquisition.

For Orbitrap Elite, MS^1 scans where acquired at mass resolution of *R* = 60,000 (FWHM at *m/z* 400) with a scan range (*m/z*) of 350–1500, a maximum injection time of 100 ms and an AGC target of 1e6. For fragmentation, only precursors with charge states 2–3 were considered. Up to 5 dependent scans were taken. For dynamic exclusion the exclusion duration was set to 30 sec.. The isolation window was set to 2.3 Da with no offset. A normalized collision energy of 35 was used in HCD mode. MS^2 scans were taken at a resolution of *R* = 15,000, with a fixed first mass of *m/z* 200. Maximum injection time was 100 ms at an AGC Target of 5e4.

For Orbitrap Eclipse, MS^1 scans where acquired at a resolution of *R* = 120,000 (FHHM at *m/z* 200) with a scan range (*m/z*) of 350–1500. Maximum injection time and an AGC target where set to automatic. For fragmentation, only precursors with charge states 2–6 were considered. Up to 20 dependent scans were taken. For dynamic exclusion the exclusion duration was set to 30 sec.. The isolation window was set to 2.3 Da with no offset. A normalized collision energy of 20, 30 and 40 (assisted CE) was used in HCD mode. MS^2 scans were taken at a resolution of *R* = 15,000 (FWHM at *m/z* 200). Maximum injection time and AGC target where set to automatic.

In all cases the spectrometer was coupled to a Thermo Scientific™ Vanquish Neo Nano-LC system with 0.1% formic acid in water (A) and 0.1% formic acid in acetonitrile (B) as solvents.

Separation of peptides for FTMS/MS was performed on a Thermo Scientific PepMap Neo C18 column with an inner diameter of 75 µm and a length of 15 cm (column A) or 50 cm (column B). Peptides were eluted with a linear gradient from 4% B to 50% B over 15 minutes and to 90% B over 4 minutes at a flow-rate of 350 nL/min (column A) or from 1.5% B to 50% B over 30 minutes and to 90% B over 5 minutes at a flow-rate of 300 nL/min (column B).

FTMS of intact proteins was performed on a Thermo Scientific™ Orbitrap Eclipse™ equipped with a Nanospray source using metal needle emitters in intact protein mode over a scan range (*m/z*) of 800-2000 at a resolution of *R* = 120,000 (FWHM at *m/z* 200). Samples were introduced by loop injection into an HPLC solvent flow of 300 nL/min using 50% B.

Deconvolution of protein mass spectra was performed with UniDec[6]. All signals in the TIC chromatogram that originate from proteins were considered for the subsequent deconvolution process. Impurities present prior to the reaction were not considered. Conversions were calculated from peak areas determined with UniDec after deconvolution by dividing the value for the product by the sum of the values for products and unmodified protein.

Miscellaneous

Lyophilization of purified products was performed using a BÜCHI Lyovapor™ L-200. Centrifugation was performed using a Thermo Scientific™ MegaFuge™ 8R. Peptide and protein reactions were conducted in

an Eppendorf ThermoMixer® C. Protein renaturation was performed with AMICON® filter units from Sigma Aldrich. Concentrations of peptide or protein stock solutions were determined by A_{280} absorption using a Thermo Scientific™ NanoDrop™ One^{C}. Extinction coefficients of the proteins used in this work were calculated based on the FASTA sequences with the assumption that all Cys residues are oxidized.

EXPERIMENTAL DATA

Synthesis of selenoxides

Preparation of DBSe (S1)

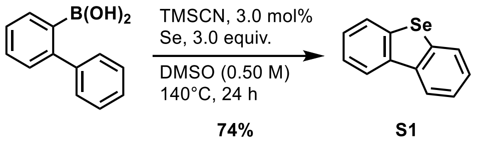

Under ambient atmosphere, an oven-dried pressure vessel (50 mL) equipped with a Teflon-coated magnetic stirring bar was charged with 2-biphenylboronic acid (2.0 g, 10 mmol, 1.0 equiv.), selenium powder (2.4 g, 30 mmol, 3.0 equiv.), TMSCN (30 mg, 38 µL, 0.30 mmol, 3.0 mol%), and DMSO (20 mL, c = 0.50 M). The reaction vessel was closed by a Teflon nozzle head and heated at 140 °C for 24 h. After cooling to 25 °C, the resulting mixture was diluted with 10 mL of diethyl ether, filtered through a pad of silica gel (ca. 15 g), eluting with additional diethyl ether (30 mL × 3). The filtrate was combined and washed with brine (100 mL × 3). The organic layer was dried over anhydrous Na_2SO_4, filtered, and the solvent evaporated under reduced pressure. The residue was purified by the chromatography on silica gel eluting with pentanes/DCM (1/0–100/1) to afford the desired compound as a colorless solid (1.7 g, 74%).

R_f = 0.30 (silica gel, hexanes).

NMR Spectroscopy:

^{1}H NMR (500 MHz, $CDCl_3$, 298 K, δ): 8.14 (dd, *J* = 8.0, 1.3 Hz, 2H), 7.89 (d, *J* = 8.0 Hz, 2H), 7.47 (ddd, *J* = 8.0, 7.2, 1.2 Hz, 2H), 7.40 (ddd, *J* = 8.4, 7.2, 1.4 Hz, 2H).

^{13}C NMR (126 MHz, $CDCl_3$, 298 K, δ): 139.4, 138.4, 127.0, 126.2, 125.0, 123.0.

^{77}Se NMR (95 MHz, $CDCl_3$, 298 K, δ): 450.6.

HRMS GC-EI (m/z) calc'd for $C_{12}H_8Se^+$ $[M]^+$, 231.9786; found, 231.9789. Deviation: –1.3 ppm.

Preparation of DBSeO (4)

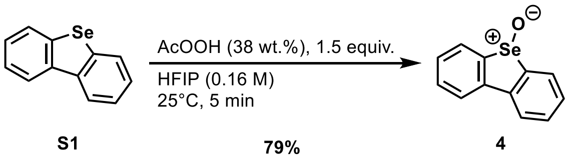

Under ambient atmosphere, a scintillation vial (20 mL) equipped with a Teflon-coated magnetic stirring bar was charged with the selenide **S1** (460 mg, 2.0 mmol, 1.0 equiv.) and the solvent HFIP (10 mL, c = 0.16 M). The mixture was stirred at 25 °C for 2 min. Then peracetic acid (38 wt.%, 0.60 g, 0.53 mL, 3.0 mmol, 1.5 equiv.) was diluted by HFIP (2.0 mL) and added to the mixture at 25 °C, resulting in the color change of the solution to deep blue, and finally to pale yellow. The mixture was stirred for another 5 min at 25 °C and subsequently transferred to a separatory funnel, washed with saturated Na_2CO_3 (20 mL) and extracted by chloroform (20 mL × 4). The organic layers were combined, dried over anhydrous Na_2SO_4, filtered, and the solvent evaporated under reduced pressure. The residue was purified by the column chromatography on silica gel eluting with DCM/MeOH (50/1–10/1) to afford the desired

compound as a colorless solid (390 mg, 79%).

R*f* = 0.30 (silica gel, DCM/MeOH = 10/1).

NMR Spectroscopy:

^{1}H NMR (500 MHz, DMSO-*d*$_6$, 298 K, δ): 8.12 (d, *J* = 7.7 Hz, 2H), 8.05 (d, *J* = 7.5 Hz, 2H), 7.67 (t, *J* = 7.1 Hz, 2H), 7.53 (t, *J* = 7.5 Hz, 2H).

^{13}C NMR (126 MHz, DMSO-*d*$_6$, 298 K, δ): 146.9, 140.4, 132.0, 129.6, 129.0, 123.4.

^{77}Se NMR (115 MHz, DMSO-*d*$_6$, 298 K, δ): 939.4.

HRMS ESI (m/z) calc'd for $C_{12}H_8OSeNa^+$ $[M+Na]^+$, 270.9633; found, 270.9631. Deviation: 0.7 ppm.

Preparation of DBSe-boronic acid S2

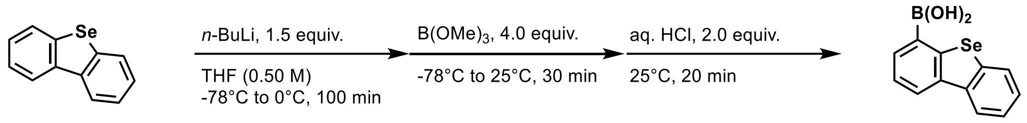

S1 **74%** in 3 stpes **S2**

An oven-dried two-neck round-bottom flask (250 mL) equipped with a Teflon-coated magnetic stirring bar was charged with the selenide **S1** (4.6 g, 20 mmol, 1.0 equiv.) and absolute THF (40 mL, c = 0.50 M) under argon atmosphere. The mixture was stirred at 25 °C until all solids dissolved. Then the flask was cooled down to –78 °C, followed by the addition of *n*-BuLi (2.5 M in pentane, 12 mL, 30 mmol, 1.5 equiv.). The resulting pale yellow solution was stirred at –78 °C for 10 min and was allowed to warm to 0 °C and stirred at 0 °C for 1.5 h, leading to the color change of the solution to bright orange. At this point, the flask was cooled down to –78 °C again, followed by the addition of $B(OMe)_3$ (8.3 g, 8.9 mL, 80 mmol, 4.0 equiv.). After the addition, the mixture was allowed to warm to 25 °C and was stirred at 25 °C for 30 min. Subsequently, 20 mL of hydrochloric acid (2.0 M, 40 mmol, 2.0 equiv.) was added to work up the reaction, and the mixture was stirred at 25 °C for 20 min. Then, the flask was kept at 20–25 °C by a cold-water bath, and 50 mL of an aqueous solution of NaOH (2 M) was slowly introduced (ca. over 30 sec.) to the flask. The mixture was stirred at 25 °C for 5 min and transferred to a separatory funnel. The aqueous layer was washed by Et_2O (120 mL) and the organic layer was extracted by NaOH (2 M, 50 mL × 2). The aqueous layers were combined and acidified to pH 1 by concentrated hydrochloric acid (37 wt.%) at 0 °C (detected by general pH test paper). The resulting suspension was filtrated and the residue was washed by cold water (20 mL × 3). The collected residue was dried in vacuo to afford the desired compound as a colorless solid (4.1 g, 74%). (Note: The compound **S2** should be stored under inert atmosphere.)

NMR Spectroscopy:

^{1}H NMR (500 MHz, DMSO-*d*$_6$, 298 K, δ): 8.36 (dd, *J* = 7.9, 1.3 Hz, 1H), 8.28 (dd, *J* = 7.9, 1.3 Hz, 1H), 8.02 (dd, *J* = 7.8, 1.2 Hz, 1H), 7.96 (dd, *J* = 7.1, 1.3 Hz, 1H), 7.50 (t, *J* = 7.5 Hz, 1H), 7.45 (td, *J* = 7.5, 1.2 Hz, 1H), 7.39 (td, *J* = 7.4, 1.3 Hz, 1H).

^{13}C NMR (126 MHz, DMSO-*d*$_6$, 298 K, δ): 145.7, 140.4, 137.6, 137.5, 133.5, 126.7, 125.8, 125.0, 124.6, 124.3, 122.8.

^{77}Se NMR (95 MHz, DMSO-*d*$_6$, 298 K, δ): 479.3.

HRMS ESI (m/z) calc'd for $C_{12}H_8BO_2Se^-$ [M–H]$^-$, 274.9788; found, 274.9791. Deviation: –1.1 ppm.

Preparation of 2-(DBSe)-Py S3

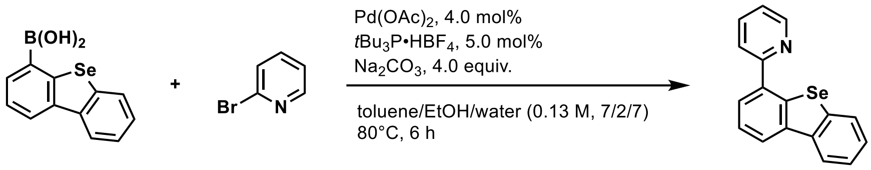

S2 **87%** **S3**

Solvent preparation: A flask (250 mL) was charged with toluene (35 mL), deionized water (35 mL), and EtOH (10 mL) under ambient atomosphere. An argon flow was gently passed through the solution via a needle (Φ 0.80 × 120 mm) for 5 min. Then, the mixture was immediately used for the reaction.

Reaction set-up: A two-neck round-bottom flask (100 mL) equipped with a reflux condenser and Teflon-coated magnetic stirring bar was charged with the boronic acid **S2** (1.5 g, 5.5 mmol, 1.1 equiv.), the coupling partner 2-bromo-pyridine (790 mg, 480 µL, 5.0 mmol, 1.0 equiv.), the catalyst $Pd(OAc)_2$ (45 mg, 0.20 mmol, 4.0 mol%), the ligand $t$$Bu_3P·HBF_4$ (73 mg, 0.25 mmol, 5.0 mol%), the base Na_2CO_3 (2.1g, 20 mmol, 4.0 equiv.), and the degassed solvent (40 mL, c = 0.125 M) under argon atmosphere. Then, the mixture was refluxed under argon atmosphere at 80 °C for 6 h. After cooling to 25 °C, the resulting mixture was transferred to a separatory funnel and washed by brine (ca. 50 mL) and the aqueous layer was extracted by EtOAc (30 mL × 3). The organic layers were combined, dried over anhydrous Na_2SO_4, filtered, and concentrated under reduced pressure. The residue was purified by chromatography on silica gel eluting with hexanes/EtOAc (50/1–20/1) to afford the desired compound as a beige solid (1.3 g, 87%).

R*f* = 0.26 (silica gel, hexanes/EtOAc = 10/1).

NMR Spectroscopy:

^{1}H NMR (600 MHz, $CDCl_3$, 298 K, δ): 8.87 (ddd, J = 4.9, 1.8, 1.0 Hz, 1H), 8.25 (dd, J = 7.8, 1.1 Hz, 1H), 8.20 (d, J = 7.6 Hz, 1H), 8.10 – 8.05 (m, 2H), 7.98 (ddd, J = 7.6, 1.2, 0.7 Hz, 1H), 7.83 (ddd, J = 8.1, 7.4, 1.8 Hz, 1H), 7.62 (t, J = 7.6 Hz, 1H), 7.47 (ddd, J = 7.8, 7.1, 1.2 Hz, 1H), 7.42 (ddd, J = 7.7, 7.1, 1.3 Hz, 1H), 7.30 (ddd, J = 7.4, 4.8, 1.0 Hz, 1H).

^{13}C NMR (151 MHz, $CDCl_3$, 298 K, δ): 155.1, 147.8, 143.8, 140.3, 137.6, 137.5, 136.8, 134.5, 126.9, 125.6, 125.2, 124.6, 124.2, 123.5, 122.6, 122.2, 120.1.

^{77}Se NMR (115 MHz, $CDCl_3$, 298 K, δ): 490.6.

HRMS ESI (m/z) calc'd for $C_{17}H_{12}NSe^+$ [M+H]$^+$, 310.0130; found, 310.0128. Deviation: 0.7 ppm.

Preparation of 2-(DBSeO)-Py 5

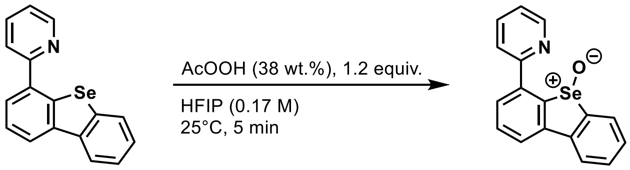

S3 **91%** **5**

Under ambient atmosphere, a scintillation vial (20 mL) equipped with a Teflon-coated magnetic stirring

bar was charged with the selenide **S3** (620 mg, 2.0 mmol, 1.0 equiv.) and the solvent HFIP (10mL, c = 0.17 M). The mixture was stirred at 25 °C for 2 min. Then peracetic acid (38 wt.%, 0.48 g, 0.42 mL, 2.4 mmol, 1.2 equiv.) was diluted by HFIP (1.6 mL) and added to the mixture at 25 °C, resulting in the color change of the solution to deep blue, and finally to bright yellow. The mixture was stirred for another 5 min at 25 °C and subsequently transferred to a separatory funnel, washed with saturated Na_2CO_3 (20 mL) and extracted by chloroform (20 mL × 4). The organic layers were combined, dried over anhydrous Na_2SO_4, filtered, and the solvent evaporated under reduced pressure. The residue was purified by the column chromatography on silica gel eluting with DCM/MeOH (50/1–9/1) to afford the desired compound as a colorless solid (590 mg, 91%).

R*f* = 0.28 (silica gel, DCM/MeOH = 10/1).

NMR Spectroscopy:

^{1}**H NMR** (500 MHz, DMSO-*d*$_6$, 298 K, δ): 8.77 (d, *J* = 4.1 Hz, 1H), 8.26 (d, *J* = 8.3 Hz, 1H), 8.19 (d, *J* = 8.0 Hz, 1H), 8.17 (d, *J* = 7.2 Hz, 1H), 8.11 (d, *J* = 7.7 Hz, 1H), 8.02 (td, *J* = 7.8, 1.8 Hz, 1H), 7.94 (d, *J* = 7.5 Hz, 1H), 7.78 (t, *J* = 7.7 Hz, 1H), 7.63 (td, *J* = 7.5, 1.1 Hz, 1H), 7.57 – 7.45 (m, 2H).

^{13}**C NMR** (126 MHz, DMSO-*d*$_6$, 298 K, δ): 151.8, 148.4, 148.0, 142.8, 142.5, 139.0, 138.2, 137.4, 132.9, 131.2, 129.6, 128.3, 126.7, 124.0, 123.9, 123.2, 120.9.

^{77}**Se NMR** (115 MHz, DMSO-*d*$_6$, 298 K, δ): 952.5.

HRMS ESI (m/z) calc'd for $C_{17}H_{12}NOSe^+$ $[M+H]^+$, 326.0079; found, 326.0080. Deviation: –0.3 ppm.

Preparation of 4-(DBSe)-Py S4

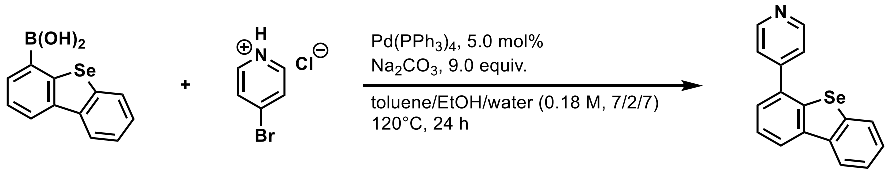

S2 **91%** **S4**

A Schlenk tube (25 mL) equipped with a Teflon-coated magnetic stirring bar was charged with the boronic acid **S2** (240 mg, 0.86 mmol, 1.0 equiv.), the coupling partner 4-bromo-pyridinium chloride (200 mg, 1.0 mmol, 1.2 equiv.), the catalyst $Pd(PPh_3)_4$ (50 mg, 0.043 mmol, 5.0 mol%), the base Na_2CO_3 (820 mg, 7.7 mmol, 9.0 equiv.), and the degassed solvent (4.8 mL, c = 0.18 M, see the section of the preparation of **S3**) under argon atmosphere. The reaction vessel was closed by a Teflon nozzle head and the reaction mixture was refluxed at 120 °C for 24 h. After cooling to 25 °C, the resulting mixture was transferred to a separatory funnel and washed by brine (ca. 20 mL) and the aqueous layer was extracted by EtOAc (20 mL × 3). The organic layers were combined, dried over anhydrous Na_2SO_4, filtered, and concentrated under reduced pressure. The residue was purified by the chromatography on silica gel eluting with hexanes/EtOAc (3/1) to afford the desired compound as a beige solid (240 mg, 91%).

R*f* = 0.17 (silica gel, hexanes/EtOAc = 3/1).

NMR Spectroscopy:

^{1}**H NMR** (500 MHz, $CDCl_3$, 298 K, δ): 8.76 (d, *J* = 6.1 Hz, 2H), 8.19 (d, *J* = 9.2 Hz, 1H), 8.17 (d, *J* = 7.1 Hz, 1H), 7.89 (d, *J* = 7.5 Hz, 1H), 7.67 (d, *J* = 6.1 Hz, 2H), 7.60 (t, *J* = 7.6 Hz, 1H), 7.50 (t, *J* = 7.6

Hz, 1H), 7.47 – 7.41 (m, 2H).

^{13}C NMR (126 MHz, $CDCl_3$, 298 K, δ): 150.3, 150.0, 139.6, 139.4, 139.0, 138.4, 137.0, 127.5, 126.9, 126.1, 125.9, 125.3, 123.3, 123.2, 122.8.

^{77}Se NMR (115 MHz, $CDCl_3$, 298 K, δ): 448.6.

HRMS ESI (m/z) calc'd for $C_{17}H_{12}NSe^+$ $[M+H]^+$, 310.0130; found, 310.0127. Deviation: 1.0 ppm.

Preparation of 4-(DBSeO)-Py S5

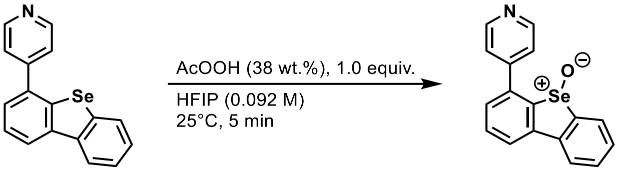

S4 **76%** **S5**

Under ambient atmosphere, a scintillation vial (4 mL) equipped with a Teflon-coated magnetic stirring bar was charged with the selenide **S4** (62 mg, 0.20 mmol, 1.0 equiv.) and the solvent HFIP (2.0 mL, c = 0.092 M). The mixture was stirred at 25 °C for 2 min to give a yellow solution. Then peracetic acid (38 wt.%, 40 mg, 35 µL, 0.20 mmol, 1.0 equiv.) was diluted by HFIP (140 µL) and added to the mixture at 25 °C, leading to the color change of the solution to colorless. The mixture was stirred for another 5 min at 25 °C and subsequently transferred to a separatory funnel, washed with saturated Na_2CO_3 (4 mL) and extracted by chloroform (4 mL × 4). The organic layers were combined, dried over anhydrous Na_2SO_4, filtered, and the solvent evaporated under reduced pressure. The residue was purified by the column chromatography on silica gel eluting with DCM/MeOH (20/1–9/1) to afford the desired compound as a colorless solid (49 mg, 76%).

R_f = 0.27 (silica gel, DCM/MeOH = 10/1).

NMR Spectroscopy:

^{1}H NMR (500 MHz, CD_3OD, 298 K, δ): 8.73 (d, *J* = 5.0 Hz, 2H), 8.17 (d, *J* = 7.7 Hz, 1H), 8.11 (d, *J* = 7.7 Hz, 1H), 7.99 (d, *J* = 7.6 Hz, 1H), 7.88 – 7.81 (m, 3H), 7.74 (t, *J* = 7.6 Hz, 1H), 7.64 (d, *J* = 8.5 Hz, 1H), 7.60 (t, *J* = 7.2 Hz, 1H).

^{13}C NMR (126 MHz, CD_3OD, 298 K, δ): 151.0, 149.2, 146.2, 145.9, 143.7, 142.3, 141.9, 134.9, 134.1, 131.7, 131.4, 129.6, 125.5, 125.14, 125.11.

^{77}Se NMR (115 MHz, CD_3OD, 298 K, δ): 921.1.

HRMS ESI (m/z) calc'd for $C_{17}H_{12}NOSe^+$ $[M+H]^+$, 326.0079; found, 326.0077. Deviation: 0.6 ppm.

Preparation of 6-bromo-*N*,*N*-dimethylpicolinamide (S6)

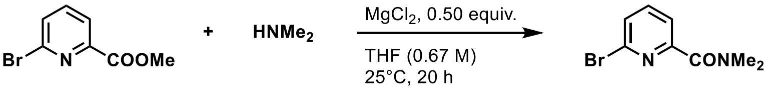

99% **S6**

Under ambient atmosphere, a scintillation vial (20 mL) equipped with a Teflon-coated magnetic stirring bar was charged with methyl 6-bromopicolinate (1.08 g, 5.00 mmol, 1.00 equiv.), $MgCl_2$ (240 mg, 2.5

mmol, 0.500 equiv.), and dimethylamine (2.0 M in THF, 7.5 mL, 15 mmol, 3.0 equiv.). Then the vial was closed by a screw cap and the mixture was stirred at 25 °C for 20 h. Subsequently, 10 mL of hydrochloric acid (2 M) was added to work up the reaction, and the mixture was stirred at 25 °C for 10 min. The resulting mixture was transferred to a separatory funnel and washed by brine (ca. 20 mL) and the aqueous layer was extracted by DCM (30 mL × 3). The organic layers were combined, dried over anhydrous Na_2SO_4, filtered, and concentrated under reduced pressure. The residue was purified by the chromatography on silica gel eluting with DCM/MeOH (1/0–100/1) to afford the desired compound as light-brown oil (1.10 g, 99%).

R*f* = 0.24 (silica gel, DCM/MeOH = 40/1).

NMR Spectroscopy:

^{1}H NMR (500 MHz, $CDCl_3$, 298 K, δ): 7.68 – 7.59 (m, 2H), 7.53 (d, *J* = 7.6 Hz, 1H), 3.12 (s, 3H), 3.10 (s, 3H).

^{13}C NMR (126 MHz, $CDCl_3$, 298 K, δ): 167.2, 155.3, 140.5, 139.5, 129.1, 122.7, 39.2, 36.0.

HRMS ESI (m/z) calc'd for $C_8H_9N_2ONaBr^+$ $[M+Na]^+$, 250.9790(6); found, 250.9791(0). Deviation: –0.2 ppm.

Preparation of DBSePy-amide S7

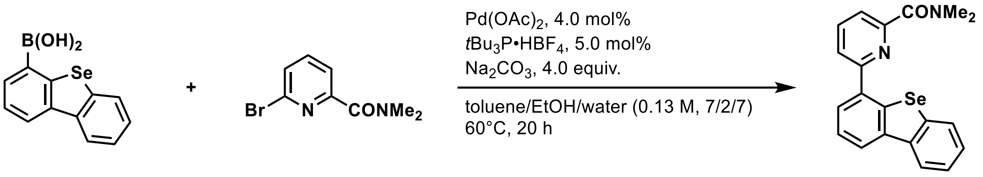

S2 95% S7

A Schlenk tube (25 mL) equipped with a Teflon-coated magnetic stirring bar was charged with the boronic acid **S2** (300 mg, 1.1 mmol, 1.1 equiv.), the coupling partner **S6** (230 mg, 170 µL, 1.0 mmol, 1.0 equiv.), the catalyst $Pd(OAc)_2$ (9.0 mg, 0.040 mmol, 4.0 mol%), the ligand $t$$Bu_3P{\cdot}HBF_4$ (15 mg, 0.050 mmol, 5.0 mol%), the base Na_2CO_3 (420 mg, 4.0 mmol, 4.0 equiv.), and the degassed solvent (8.0 mL, c = 0.125 M, see the section of the preparation of **S3**) under argon atmosphere. The reaction vessel was closed by a Teflon nozzle head and the mixture was heated at 60 °C for 20 h. After cooling to 25 °C, the resulting mixture was transferred to a separatory funnel and was washed by brine (ca. 30 mL). The aqueous layer was extracted by DCM (30 mL × 3). The organic layers were combined, dried over anhydrous Na_2SO_4, filtered, and concentrated under reduced pressure. The residue was purified by the chromatography on silica gel eluting with hexanes/DCM/EtOAc (4/0/1–4/1/5–7/3/20) to afford the desired compound as a yellow solid (360 mg, 95%).

R*f* = 0.26 (silica gel, DCM/MeOH = 40/1).

NMR Spectroscopy:

^{1}H NMR (500 MHz, $CDCl_3$, 298 K, δ): 8.26 (d, *J* = 8.0 Hz, 1H), 8.19 (d, *J* = 7.8 Hz, 1H), 8.11 (d, *J* = 8.1 Hz, 1H), 8.08 (d, *J* = 7.7 Hz, 1H), 7.97 (d, *J* = 7.6 Hz, 1H), 7.93 (td, *J* = 7.8, 1.8 Hz, 1H), 7.62 (td, *J* = 7.7, 1.7 Hz, 1H), 7.57 (d, *J* = 8.1 Hz, 1H), 7.47 (t, *J* = 7.5 Hz, 1H), 7.42 (t, *J* = 7.4 Hz, 1H), 3.30 (s, 3H), 3.17 (s, 3H).

^{13}C NMR (126 MHz, $CDCl_3$, 298 K, δ): 169.0, 154.0, 153.1, 143.4, 140.3, 137.9, 137.7, 137.5, 134.0, 127.0, 125.6, 125.2, 124.6, 124.5, 123.8, 122.7, 121.7, 120.6, 39.6, 35.8.

^{77}Se NMR (115 MHz, $CDCl_3$, 298 K, δ): 493.0.

HRMS GC-EI (m/z) calc'd for $C_{20}H_{16}N_2OSe^+$ $[M]^+$, 380.0422; found, 380.0425. Deviation: –0.8 ppm.

Preparation of DBSeOPy-amide 6

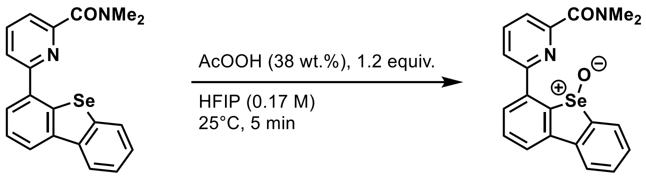

S7 **94%** **6**

Under ambient atmosphere, a scintillation vial (20 mL) equipped with a Teflon-coated magnetic stirring bar was charged with the selenide **S7** (380 mg, 1.0 mmol, 1.0 equiv.) and the solvent HFIP (5.0 mL, c = 0.17 M). The mixture was stirred at 25 °C for 2 min. Then peracetic acid (38 wt.%, 0.24 g, 0.21 mL, 1.2 mmol, 1.2 equiv.) was diluted by HFIP (0.80 mL) and added to the mixture at 25 °C, resulting in the color change of the solution to deep blue, and finally to orange. The mixture was stirred for another 5 min at 25 °C and subsequently transferred to a separatory funnel, washed with saturated Na_2CO_3 (10 mL) and extracted by chloroform (10 mL × 4). The organic layers were combined, dried over anhydrous Na_2SO_4, filtered, and the solvent evaporated under reduced pressure. The residue was purified by the column chromatography on silica gel eluting with DCM/MeOH (50/1–4/1) to afford the desired compound as a beige solid (370 mg, 94%).

R_f = 0.22 (silica gel, DCM/MeOH = 10/1).

NMR Spectroscopy:

^{1}H NMR (600 MHz, CD_3OD, 298 K, δ): 8.29 (d, J = 7.7 Hz, 1H), 8.23 (dd, J = 8.1, 1.0 Hz, 1H), 8.13 – 8.09 (m, 2H), 8.04 (d, J = 7.5 Hz, 1H), 7.95 (ddd, J = 7.5, 1.2, 0.6 Hz, 1H), 7.83 (t, J = 7.7 Hz, 1H), 7.66 (td, J = 7.6, 1.2 Hz, 1H), 7.58 (dd, J = 7.6, 0.9 Hz, 1H), 7.56 (td, J = 7.5, 1.1 Hz, 1H), 3.24 (s, 3H), 3.10 (s, 3H).

^{13}C NMR (151 MHz, CD_3OD, 298 K, δ): 170.4, 154.6, 152.8, 148.0, 144.7, 142.0, 140.8, 140.5, 138.4, 135.1, 133.3, 131.4, 129.3, 128.1, 125.7, 124.6, 123.3, 122.3, 39.7, 35.5.

^{77}Se NMR (115 MHz, CD_3OD, 298 K, δ): 923.3.

HRMS ESI (m/z) calc'd for $C_{20}H_{17}N_2O_2Se^+$ $[M+H]^+$, 397.0450; found, 397.0452. Deviation: –0.5 ppm.

Preparation of 2-(6-bromopyridin-2-yl)oxazole (S8)

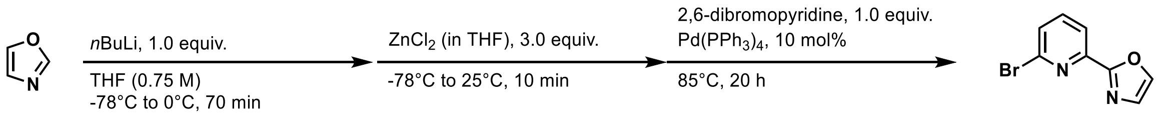

64% in 3 steps **S8**

An oven-dried two-neck round-bottom flask (100 mL) equipped with a Teflon-coated magnetic stirring bar was charged with oxazole (410 mg, 400 µL, 6.0 mmol, 1.0 equiv.) and absolute THF (8.0 mL, c = 0.75 M)

under argon atmosphere. The mixture was stirred at 25 °C for 2 min and subsequently cooled down to –78 °C, followed by the addition of *n*-BuLi (2.5 M in pentane, 2.4 mL, 6.0 mmol, 1.0 equiv.). The resulting pale yellow solution was stirred at –78 °C for 10 min and was allowed to warm to 0 °C and stirred at 0 °C for 1 h, leading to the color change of the solution to bright orange. At this point, the flask was cooled down to –78 °C again, followed by the addition of a suspension of $ZnCl_2$ (2.5 g, 18 mmol, 3.0 equiv.) in absolute THF (15 mL). After the addition, the mixture was allowed to warm to 25 °C and was stirred at 25 °C for 10 min. Next, the coupling partner 2,6-dibromopyridine (1.4 g, 6.0 mmol, 1.0 equiv.) and the catalyst $Pd(PPh_3)_4$ (690 mg, 0.60 mmol, 10 mol%) were added and the flask was equipped with a reflux condenser. The reaction mixture was refluxed at 85 °C for 20 h under argon atmosphere. After cooling to 25 °C, the mixture was transferred to a separatory funnel and was washed by an aqueous solution of EDTA-Na_2 (pH 8.5, c = 0.3 M, 80 mL, 24 mmol, 4.0 equiv.). The aqueous layer was extracted by EtOAc (40 mL × 3). The organic layers were combined, dried over anhydrous Na_2SO_4, filtered, and concentrated under reduced pressure. The residue was purified by the chromatography on silica gel eluting with hexanes/EtOAc (4/1–2/1) to afford the desired compound as a colorless needle crystal (860 mg, 64%).

R_f = 0.28 (silica gel, hexanes/EtOAc = 2/1).

NMR Spectroscopy:

^{1}H NMR (500 MHz, $CDCl_3$, 298 K, δ): 8.10 (dd, *J* = 7.7, 0.9 Hz, 1H), 7.82 (s, 1H), 7.68 (t, *J* = 7.8 Hz, 1H), 7.57 (dd, *J* = 7.9, 0.9 Hz, 1H), 7.31 (s, 1H).

^{13}C NMR (126 MHz, $CDCl_3$, 298 K, δ): 159.5, 146.9, 142.5, 140.4, 139.3, 129.4, 129.2, 120.9.

HRMS GC-EI (m/z) calc'd for $C_8H_5N_2OBr^+$ $[M]^+$, 223.9580; found, 223.9581. Deviation: –0.5 ppm.

Preparation of DBSePy-oxa S9

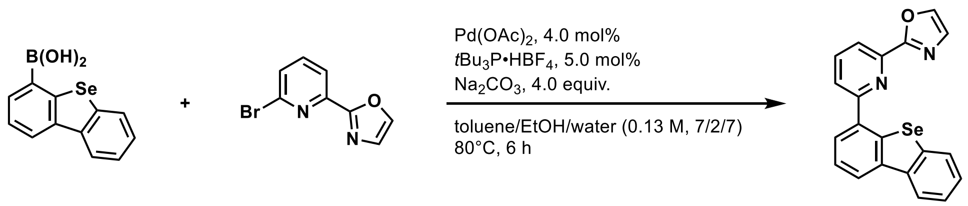

S2 **S8** **84%** **S9**

A two-neck round-bottom flask (100 mL) equipped with a reflux condenser and Teflon-coated magnetic stirring bar was charged with the boronic acid **S2** (910 mg, 3.3 mmol, 1.1 equiv.), the coupling partner **S8** (680 mg, 3.0 mmol, 1.0 equiv.), the catalyst $Pd(OAc)_2$ (27 mg, 0.12 mmol, 4.0 mol%), the ligand *t*$Bu_3P·HBF_4$ (44 mg, 0.15 mmol, 5.0 mol%), the base Na_2CO_3 (1.3 g, 12 mmol, 4.0 equiv.), and the degassed solvent (24 mL, c = 0.13 M, see the section of the preparation of **S3**) under argon atmosphere. Then the mixture was refluxed under argon atmosphere at 80 °C for 6 h. After cooling to 25 °C, the reaction mixture was transferred to a separatory funnel and was washed by brine (ca. 30 mL). The aqueous layer was extracted by EtOAc (30 mL × 3). The organic layers were combined, dried over anhydrous Na_2SO_4, filtered, and concentrated under reduced pressure. The residue was purified by the chromatography on silica gel eluting with hexanes/EtOAc (4/1–1/1) to afford the desired compound as a beige solid (950 mg, 84%).

R_f = 0.30 (silica gel, hexanes/EtOAc = 2/1).

NMR Spectroscopy:

^{1}H NMR (500 MHz, $CDCl_3$, 298 K, δ): 8.29 (dd, *J* = 7.8, 1.1 Hz, 1H), 8.21 (dd, *J* = 8.1, 1.2 Hz, 1H), 8.18 (d, *J* = 2.9 Hz, 1H), 8.16 (d, *J* = 3.6 Hz, 1H), 8.12 (dd, *J* = 7.7, 1.2 Hz, 1H), 8.05 (d, *J* = 7.5 Hz, 1H), 8.02 (d, *J* = 0.7 Hz, 1H), 7.98 (t, *J* = 7.9 Hz, 1H), 7.64 (t, *J* = 7.7 Hz, 1H), 7.49 (td, *J* = 7.5, 1.3 Hz, 1H), 7.46 – 7.41 (m, 2H).

^{13}C NMR (126 MHz, $CDCl_3$, 298 K, δ): 161.2, 155.6, 145.0, 143.8, 140.5, 140.2, 138.1, 137.8, 137.7, 133.9, 129.0, 127.0, 125.8, 125.2, 124.6(1), 124.6(0), 123.9, 122.7, 121.2, 120.8.

^{77}Se NMR (115 MHz, $CDCl_3$, 298 K, δ): 498.2.

HRMS ESI (m/z) calc'd for $C_{20}H_{13}N_2OSe^+$ $[M+H]^+$, 377.0188; found, 377.0190. Deviation: –0.5 ppm.

Preparation of DBSeOPy-oxa 1

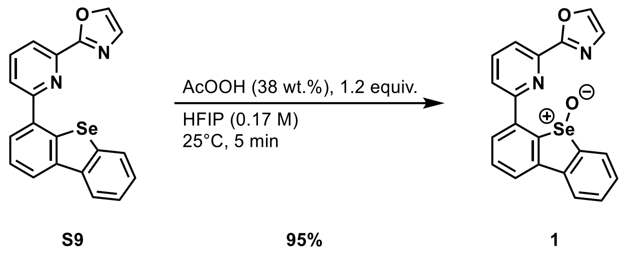

Under ambient atmosphere, a scintillation vial (20 mL) equipped with a Teflon-coated magnetic stirring bar was charged with the selenide **S9** (751 mg, 2.00 mmol, 1.00 equiv.) and the solvent HFIP (10 mL, c = 0.17 M). The mixture was stirred at 25 °C for 2 min. Then peracetic acid (38 wt.%, 0.48 g, 0.42 mL, 2.4 mmol, 1.2 equiv.) was diluted by HFIP (1.6 mL) and added to the mixture at 25 °C, resulting in the color change of the solution to deep blue, and finally to orange. The mixture was stirred for another 5 min at 25 °C and subsequently transferred to a separatory funnel, washed with saturated Na_2CO_3 (20 mL) and extracted by chloroform (20 mL × 4). The organic layers were combined, dried over anhydrous Na_2SO_4, filtered, and the solvent evaporated under reduced pressure. The residue was purified by the column chromatography on silica gel eluting with DCM/MeOH (50/1–4/1) to afford the desired compound as a colorless solid (740 mg, 95%).

R_f = 0.26 (silica gel, DCM/MeOH = 10/1).

NMR Spectroscopy:

^{1}H NMR (500 MHz, CD_3OD, 298 K, δ): 8.19 (d, *J* = 0.7 Hz, 1H), 8.17 (dd, *J* = 8.0, 1.0 Hz, 1H), 8.13 – 8.05 (m, 2H), 8.02 (t, *J* = 7.8 Hz, 1H), 7.96 (dd, *J* = 7.6, 1.0 Hz, 1H), 7.93 (d, *J* = 7.7 Hz, 1H), 7.85 (d, *J* = 7.9 Hz, 1H), 7.71 (t, *J* = 7.7 Hz, 1H), 7.62 (td, *J* = 7.5, 1.2 Hz, 1H), 7.53 (td, *J* = 7.5, 1.1 Hz, 1H), 7.44 (d, *J* = 0.7 Hz, 1H).

^{13}C NMR (126 MHz, CD_3OD, 298 K, δ): 161.5, 152.7, 147.7, 146.0, 144.7, 142.3, 141.7, 140.8, 140.5, 137.7, 135.0, 133.3, 131.4, 129.8, 129.3, 128.0, 125.8, 124.6, 123.2, 122.5.

^{77}Se NMR (115 MHz, CD_3OD, 298 K, δ): 924.8.

HRMS ESI (m/z) calc'd for $C_{20}H_{13}N_2O_2Se^+$ $[M+H]^+$, 393.0137; found, 393.0138. Deviation: –0.3 ppm.

Preparation of DBSePy-py S10

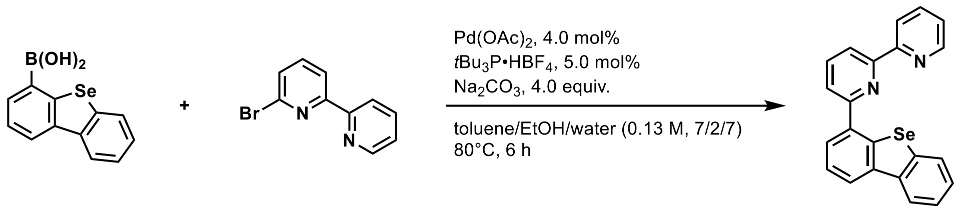

S2 **87%** **S10**

A scintillation vial (20 mL) equipped with a Teflon-coated magnetic stirring bar was charged with the boronic acid **S2** (151 mg, 0.55 mmol, 1.1 equiv.), the coupling partner 6-bromo-2,2'-bipyridine (118 mg, 0.50 mmol, 1.0 equiv.), the catalyst $Pd(OAc)_2$ (4.5 mg, 20 µmol, 4.0 mol%), the ligand $t$$Bu_3P·HBF_4$ (7.3 mg, 25 µmol, 5.0 mol%), the base Na_2CO_3 (212 mg, 2 mmol, 4.0 equiv.), and the degassed solvent (4.0 mL, c = 0.13 M, see the section of the preparation of **S3**) under argon atmosphere. Then the mixture was refluxed under argon atmosphere at 80 °C for 6 h. After cooling to 25 °C, the reaction mixture was transferred to a separatory funnel and was washed by brine (ca. 15 mL). The aqueous layer was extracted by EtOAc (10 mL × 3). The organic layers were combined, dried over anhydrous Na_2SO_4, filtered, and concentrated under reduced pressure. The residue was purified by the chromatography on silica gel eluting with pentane/EtOAc (4/1–2/1) to afford the desired compound as a colorless solid (167 mg, 87%).

R_f = 0.29 (silica gel, pentane/EtOAc = 4/1).

NMR Spectroscopy:

^{1}H NMR (500 MHz, $CDCl_3$, 298 K, δ): 8.93 (d, J = 8.0 Hz, 1H), 8.78–8.75 (m, 1H), 8.45 (dd, J = 7.5, 1.0 Hz, 1H), 8.27 (dd, J = 7.5, 1.0 Hz, 1H), 8.22 (d, J = 8.0 Hz, 1H), 8.06 (dd, J = 12, 8.0 Hz, 2H), 8.01–7.96 (m, 3H), 7.65 (t, J = 7.5 Hz, 1H), 7.49 (dd, J = 7.5, 1.0 Hz, 1H), 7.46–7.38 (m, 2H).

^{13}C NMR (151 MHz, $CDCl_3$, 298 K, δ): δ 156.1(4), 156.0(7), 155.6, 149.4, 142.7, 140.2, 137.9, 137.7, 137.2, 135.2, 127.0, 125.7, 125.4, 125.2, 124.7, 124.1, 123.5, 122.8, 122.5, 121.1, 120.2.

^{77}Se NMR (115 MHz, $CDCl_3$, 298 K, δ): 484.8.

HRMS ESI (m/z) calc'd for $C_{22}H_{14}N_2Se^+$ $[M]^+$, 386.0317; found, 386.0324. Deviation: -1.8 ppm.

Preparation of DBSeOPy-py S11

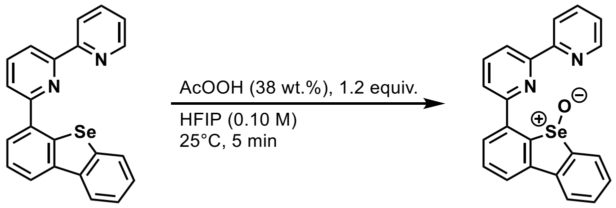

S10 **91%** **S11**

Under ambient atmosphere, a scintillation vial (4 mL) equipped with a Teflon-coated magnetic stirring bar was charged with the selenide **S10** (77 mg, 0.20 mmol, 1.0 equiv.) and the solvent HFIP (1 mL). The mixture was stirred at 25 °C for 2 min. Then peracetic acid (38 wt.%, 48 mg, 42 µL, 0.24 mmol, 1.2 equiv.) was diluted by HFIP (1 mL, final concentration c = 0.10 M) and added to the mixture at 25 °C, resulting in the color change of the solution from yellow to pale yellow. The mixture was stirred for

another 5 min at 25 °C and subsequently transferred to a separatory funnel, washed with saturated Na_2CO_3 (10 mL) and extracted by chloroform (5 mL × 3). The organic layers were combined, dried over anhydrous Na_2SO_4, filtered, and the solvent evaporated under reduced pressure. The residue was purified by the column chromatography on silica gel eluting with $CHCl_3$/MeOH (20/1–10/1) to afford the desired compound as a colorless solid (73 mg, 91%).

R*f* = 0.30 (silica gel, $CHCl_3$/MeOH = 10/1).

NMR Spectroscopy:

^{1}H NMR (600 MHz, $CDCl_3$, 298 K, δ): 9.18 (dt, *J* = 7.8, 1.2 Hz, 1H), 8.72 (ddd, *J* = 4.8, 1.8, 0.6 Hz, 1H), 8.44–8.40 (m, 1H), 8.01 (ddd, *J* = 7.8, 7.5, 1.8 Hz, 1H), 7.95–7.93 (m, 3H), 7.90 (ddd, *J* = 7.2, 1.2, 0.6 Hz, 1H), 7.85 (dd, *J* = 7.8, 1.2 Hz, 1H), 7.82 (d, *J* = 7.8 Hz, 1H), 7.65 (t, *J* = 7.2 Hz, 1H), 7.54 (td, *J* = 7.2, 1.2 Hz, 1H), 7.45 (td, *J* = 7.2, 1.2 Hz, 1H), 7.37 (ddd, *J* = 7.8, 4.8, 1.2 Hz, 1H).

^{13}C NMR (151 MHz, $CDCl_3$, 298 K, δ): 157.2, 155.9, 152.4, 149.2, 147.4, 143.4, 142.2, 139.8, 138.6, 138.5, 137.8, 133.1, 131.7, 130.0, 128.4, 127.2, 124.1, 123.6, 123.3, 123.1, 122.3, 120.9.

^{77}Se NMR (115 MHz, $CDCl_3$, 298 K, δ): 934.4.

HRMS ESI (m/z) calc'd for $C_{22}H_{15}N_2OSe^+$ $[M+H]^+$, 403.0344; found, 403.0347. Deviation: -0.7 ppm.

Synthesis of tyrosinamide chalcogenonium salts

Preparation of NAc-Tyr-NH$_2$ derived thianthreniumsalt S12

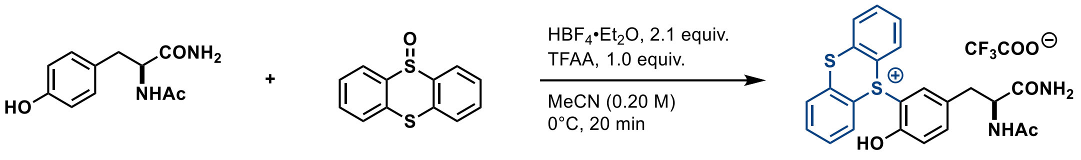

2 **3** **80%** **S12**

Under ambient atmosphere, a scintillation vial (20 mL) equipped with Teflon-coated magnetic stirring bar was charged with thianthrene *S*-oxide **3** (230 mg, 1.0 mmol, 1.0 equiv.), the tyrosinamide **2** (220 mg, 1.0 mmol, 1.0 equiv.), and the solvent MeCN (5.0 mL, c = 0.20 M). The mixture was stirred at 25 °C for 5 min and then cooled down to 0 °C. $HBF_4{\cdot}Et_2O$ (0.34 g, 0.29 mL, 2.1 mmol, 2.1 equiv.) was introduced and the mixture was stirred at 0 °C for 5 min to give a clear solution. Thereafter, trifluoroacetic anhydride (0.21 g, 0.14 mL, 1.0 mmol, 1.0 equiv.) was added dropwise over 10 min. The mixture was stirred at 0 °C for another 5 min followed by the addition of UHQ-H_2O (0.18 g, 0.18 mL, 10 mmol, 10 equiv.). The mixture was diluted by MeOH (5.0 mL) and the solvent evaporated under reduced pressure. The residue was purified by the column chromatography on silica gel. Firstly, the eluent MeCN/MeOH (9/1) was used to remove thianthrene and unreacted tyrosinamide. Then, the column chromatography was completely dried under argon flow and re-equilibriated by DCM/MeOH (9/1). Subsequently, the second eluent DCM/MeOH (9/1–4/1) + 1%(v/v) TFA was used to obtain fractions containing the thianthrenium salt. The desired fractions were combined and concentrated to ca. 10 mL under reduced pressure to give a syrup-like, colorless liquid. The syrup-like liquid was diluted by UHQ-H_2O (ca. 100 mL) and the mixture was lyophilized to afford the desired compound as a pink solid (440 mg, 80%).

R*f* = 0.55 (silica gel, DCM/MeOH = 4/1).

NMR Spectroscopy:

^{1}H NMR (600 MHz, CD_3OD, 298 K, δ): 8.31 (ddd, *J* = 8.0, 2.3, 1.3 Hz, 2H), 8.00 (ddd, *J* = 9.6, 8.0, 1.2 Hz, 2H), 7.90 – 7.83 (m, 2H), 7.77 (dddd, *J* = 8.0, 7.4, 4.6, 1.3 Hz, 2H), 7.39 (dd, *J* = 8.4, 2.1 Hz, 1H), 6.96 (d, *J* = 8.4 Hz, 1H), 6.52 (d, *J* = 2.0 Hz, 1H), 4.41 (dd, *J* = 8.7, 5.6 Hz, 1H), 2.96 (dd, *J* = 14.1, 5.6 Hz, 1H), 2.69 (dd, *J* = 14.1, 8.7 Hz, 1H), 1.87 (s, 3H).

^{13}C NMR (151 MHz, CD_3OD, 298 K, δ): 175.4, 173.0, 157.3, 138.7, 138.5, 138.0, 135.93, 135.87, 135.7, 135.6, 131.7, 131.6, 131.2, 130.7, 130.6, 130.0, 119.4, 119.2, 118.6, 108.4, 55.1, 37.6, 22.6.

^{19}F NMR (565 MHz, CD_3OD, 298 K, δ): -77.3 (br).

HRMS ESI-pos (m/z) calc'd for $C_{23}H_{21}N_2O_3S_2^+$ $[M]^+$, 437.0988; found, 437.0987. Deviation: 0.2 ppm.

HRMS ESI-neg (m/z) calc'd for $C_2O_2F_3^-$ $[M]^-$, 112.9856; found, 112.9857. Deviation: –0.9 ppm.

Preparation of NAc-Tyr-NH_2 derived DBSe-selenonium salt S13

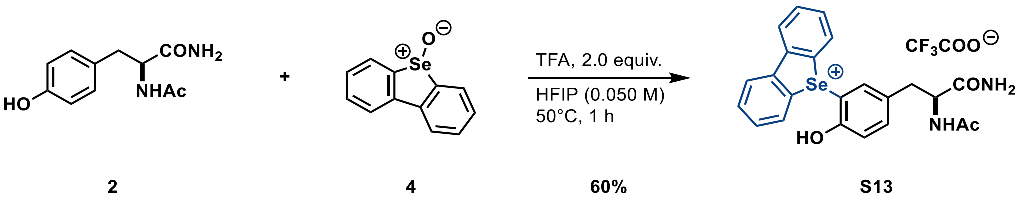

Under ambient atmosphere, a scintillation vial (20 mL) equipped with Teflon-coated magnetic stirring bar was charged with the the selenoxide **4** (49 mg, 0.20 mmol, 1.0 equiv.) and the solvent HFIP (4.0 mL, c = 50 mM). The mixture was stirred at 25 °C for 2 min to give a clear, colorless solution. Then the tyrosinamide **2** (44 mg, 0.20 mmol, 1.0 equiv.) was added as powder. The suspension was stirred at 25 °C for 5 min to give a clear, purple solution. Subsequently, trifluoroacetic acid (46 mg, 31 µL, 0.40 mmol, 2.0 equiv.) was introduced and the mixture was stirred at 50 °C for 1 h to give a dark green solution. After cooling to 25 °C, the mixture was diluted by MeOH (4.0 mL), followed by the addition of a solution of Na_2SO_3 (c = 50 mM, 1.0 mL, 0.050 mmol, 6.3 mg, 0.25 equiv.) in UHQ-H_2O. The mixture was stirred at 25 °C for 5 min and the solvent evaporated under reduced pressure. The residue was purified by the column chromatography on silica gel. Firstly, the eluent MeCN/MeOH (4/1) was used to remove selenide and unreacted tyrosinamide. Then, the column chromatography was completely dried under argon flow and the second eluent DCM/MeOH (9/1–4/1) + 1%(v/v) TFA was used to obtain fractions containing the selenonium salt. The desired fractions were combined and concentrated to ca. 3 mL under reduced pressure to give a syrup-like, colorless liquid. The syrup-like liquid was diluted by UHQ-H_2O (ca. 40 mL) and the mixture was lyophilized to afford the desired compound as a purple solid (68 mg, 60%).

R*f* = 0.14 (silica gel, DCM/MeOH = 9/1).

NMR Spectroscopy:

^{1}H NMR (600 MHz, CD_3OD, 298 K, δ): 8.32 (ddd, *J* = 10.6, 7.9, 1.2 Hz, 2H), 8.22 (t, *J* = 7.6 Hz, 2H), δ 7.90 (td, *J* = 7.7, 1.1 Hz, 1H), δ 7.87 (td, *J* = 8.0, 1.3 Hz, 1H), 7.74 (td, *J* = 7.7, 1.2 Hz, 1H), 7.71 (td, *J* = 7.7, 1.2 Hz, 1H), 7.31 (dd, *J* = 8.4, 2.0 Hz, 1H), 7.04 (d, *J* = 8.4 Hz, 1H), 6.46 (d, *J* = 2.0 Hz, 1H), 4.34 (dd, *J* = 9.0, 5.3 Hz, 1H), 2.90 (dd, *J* = 14.2, 5.2 Hz, 1H), 2.58 (dd, *J* = 14.2, 9.1 Hz, 1H), 1.70 (s, 3H).

^{13}C NMR (151 MHz, CD_3OD, 298 K, δ): 175.5, 172.8, 157.0, 143.2, 142.9, 136.6, 135.3, 134.6, 134.4, 134.3, 132.6, 132.2, 132.1, 131.3, 130.9, 127.8, 126.3, 126.2, 119.0, 117.6, 55.0, 37.8, 22.4.

^{19}F NMR (565 MHz, CD_3OD, 298 K, δ): -77.2 (br).

^{77}Se NMR (115 MHz, CD_3OD, 298 K, δ): 490.2.

HRMS ESI-pos (m/z) calc'd for $C_{23}H_{21}N_2O_3Se^+$ $[M]^+$, 453.0712; found, 453.0714. Deviation: –0.4 ppm.

HRMS ESI-neg (m/z) calc'd for $C_2O_2F_3^-$ $[M]^-$, 112.9856; found, 112.9857. Deviation: –0.9 ppm.

Preparation of NAc-Tyr-NH_2 derived 2-(DBSe)-Py-selenonium salt S14

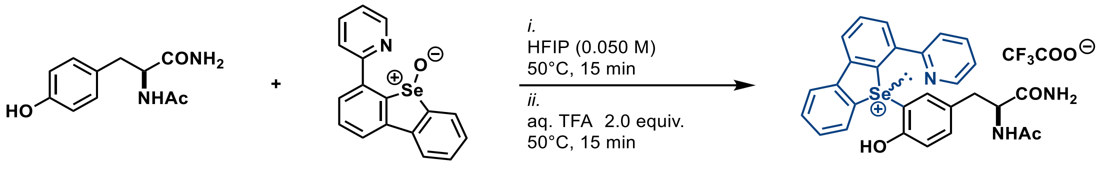

2 5 90% S14

Under ambient atmosphere, a scintillation vial (20 mL) equipped with Teflon-coated magnetic stirring bar was charged with the the selenoxide **5** (65 mg, 0.20 mmol, 1.0 equiv.), the tyrosinamide **2** (44 mg, 0.20 mmol, 1.0 equiv.), and the solvent HFIP (4.0 mL, c = 50 mM). The mixture was stirred at 25 °C for 2 min to give a clear, pale purple solution. Next, the mixture was stirred at 50 °C for 15 min, resulting in the color change of the solution to deep blue, and finally to bright yellow. Then, a solution of trifluoroacetic acid (c = 1.0 M, 0.40 mL, 0.40 mmol, 46 mg, 2.0 equiv.) in UHQ-H_2O was introduced to work up the reaction and the mixture was stirred at 50 °C for another 15 min. After cooling to 25 °C, the mixture was diluted by MeOH (4.0 mL), followed by the addition of a solution of Na_2SO_3 (c = 50 mM, 1.0 mL, 0.050 mmol, 6.3 mg, 0.25 equiv.) in UHQ-H_2O. The mixture was stirred at 25 °C for 5 min and the solvent evaporated under reduced pressure. The residue was purified by the column chromatography on silica gel. Firstly, the eluent MeCN/MeOH (9/1) was used to remove selenide and unreacted tyrosinamide. Then, the column chromatography was completely dried under argon flow and re-equibriated by DCM/MeOH (9/1). Subsequently, the second eluent DCM/MeOH (9/1–4/1) + 1%(v/v) TFA was used to obtain fractions containing the product selenonium salt. The desired fractions were combined and concentrated to ca. 3 mL under reduced pressure to give a syrup-like, pale yellow liquid. The syrup-like liquid was diluted by UHQ-H_2O (ca. 40 mL) and the mixture was lyophilized to afford the desired compound as a purple solid (120 mg, 90%).

R*f* = 0.40 (silica gel, DCM/MeOH = 4/1).

NMR Spectroscopy (mixture of a pair of epimer, ratio 1/1):

^{1}H NMR (600 MHz, CD_3OD, 298 K, δ): 8.66 (ddd, *J* = 5.0, 1.7, 0.9 Hz, 1H), 8.62 (ddd, *J* = 4.9, 1.7, 0.9 Hz, 1H), 8.57 (dd, *J* = 3.6, 1.0 Hz, 1H), 8.56 (dd, *J* = 3.6, 1.0 Hz, 1H), 8.50 (ddd, *J* = 7.8, 2.0, 1.0 Hz, 2H), 8.43 (d, *J* = 4.4 Hz, 1H), 8.42 (d, *J* = 4.4 Hz, 1H), 8.34 (d, *J* = 7.8 Hz, 2H), 8.15 (td, *J* = 7.7, 1.9 Hz, 2H), 8.08 – 7.99 (m, 4H), 7.78 (td, *J* = 7.6, 1.0 Hz, 2H), 7.61 (tdd, *J* = 7.6, 4.4, 1.2 Hz, 2H), 7.46 (dddd, *J* = 8.6, 7.4, 4.9, 0.9 Hz, 2H), 7.22 (d, *J* = 2.0 Hz, 1H), 7.20 (d, *J* = 2.0 Hz, 1H), 7.06 (d, *J* = 2.2 Hz, 1H), 7.04 (d, *J* = 2.2 Hz, 1H), 6.22 – 6.27 (m, 2H), 4.14 (ddd, *J* = 12.5, 9.5, 4.9 Hz, 2H), 2.75 (ddd, *J* = 14.6, 9.6, 4.9 Hz, 2H), 2.44 (dd, *J* = 14.5, 9.3 Hz, 1H), 2.37 (dd, *J* = 14.3, 9.8 Hz, 1H), 1.51 (s, 3H), 1.47 (s, 3H).

^{13}C NMR (151 MHz, CD_3OD, 298 K, δ): 175.54, 175.53, 172.6, 172.5, 157.0, 156.9, 150.2, 150.1, 148.1, 147.9, 145.92, 145.85, 140.8, 140.29, 140.27, 140.18, 140.16, 138.2, 138.1, 136.1, 136.04, 136.03, 133.36, 133.34, 132.6, 132.5, 132.2, 132.1, 129.0, 128.9, 128.3, 128.2, 127.39, 127.37, 127.3, 127.2, 127.04, 126.97, 126.02, 125.96, 125.78, 125.75, 121.91, 121.86, 120.3, 118.01, 117.98, 54.8, 54.7, 37.5, 37.3, 22.24, 22.21.

^{19}F NMR (565 MHz, CD_3OD, 298 K, δ): -77.2 (br).

^{77}Se NMR (115 MHz, CD_3OD, 298 K, δ): 499.2, 498.7.

HRMS ESI-pos (m/z) calc'd for $C_{28}H_{24}N_3O_3Se^+$ $[M]^+$, 530.0977; found, 530.0985. Deviation: –1.5 ppm.

HRMS ESI-neg (m/z) calc'd for $C_2O_2F_3^-$ $[M]^-$, 112.9856; found, 112.9857. Deviation: –0.9 ppm.

Preparation of NAc-Tyr-NH_2 derived DBSePy-amide-selenonium salt S15

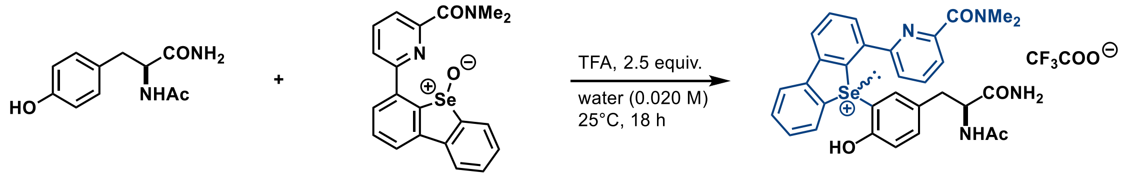

2 **6** **54%** **S15**

Under ambient atmosphere, a scintillation vial (20 mL) equipped with Teflon-coated magnetic stirring bar was charged with the selenoxide **6** (140 mg, 0.30 mmol, 1.0 equiv.) and the solvent UHQ-H_2O (15 mL, c = 20 mM). The mixture was stirred at 25 °C for 2 min to give a white suspension. Then trifluoroacetic acid (86 mg, 58 µL, 0.75 mmol, 2.5 equiv.) was added and the mixture was stirred at 25 °C for another 2 min to give a clear, yellow solution. Subsequently, the tyrosinamide **2** (80 mg, 0.36 mmol, 1.2 equiv.) was added and the mixture was stirred at 25 °C for 18 h. Subsequently, the mixture was transferred to a lyophilizing flask (250 mL), diluted by MeCN (15 mL), followed by the addition of a solution of Na_2SO_3 (c = 0.50 M, 0.30 mL, 0.15 mmol, 19 mg, 0.50 equiv.) in UHQ-H_2O. The mixture was stirred at 25 °C for 5 min and was further diluted by UHQ-H_2O (ca. 60 mL) and lyophilized. The residue was purified by the column chromatography on silica gel. Firstly, the eluent MeCN/MeOH (9/1–7/3) was used to remove selenide and unreacted tyrosinamide. Then, the column chromatography was dried under argon flow and re-equibriated by DCM/MeOH (9/1). Subsequently, the second eluent DCM/MeOH (9/1–4/1) + 1%(v/v) TFA was used to obtain fractions containing the selenonium salt. The desired fractions were combined and concentrated to ca. 5 mL under reduced pressure to give a syrup-like, pale yellow liquid. The syrup-like liquid was diluted by UHQ-H_2O (ca. 100 mL) and the mixture was lyophilized to afford the desired compound as a purple solid (120 mg, 54%).

R_f = 0.21 (silica gel, DCM/MeOH = 4/1).

NMR Spectroscopy (mixture of a pair of epimer, ratio 1/1):

^{1}H NMR (600 MHz, CD_3OD, 298 K, δ): 8.66 (d, *J* = 7.0 Hz, 2H), 8.56 – 8.53 (m, 2H), 8.51 (d, *J* = 7.7 Hz, 2H), 8.29 (d, *J* = 7.3 Hz, 2H), 8.22 – 8.14 (m, 6H), 7.75 (td, *J* = 7.6, 1.0 Hz, 2H), 7.64 – 7.57 (m, 2H), 7.50 (ddd, *J* = 7.6, 1.7, 0.7 Hz, 2H), 7.24 (dd, *J* = 8.4, 2.0 Hz, 1H), 7.21 (dd, *J* = 8.4, 2.0 Hz, 1H), 7.08 (d, *J* = 5.2 Hz, 1H), 7.07 (d, *J* = 5.2 Hz, 1H), 6.26 (d, *J* = 2.0 Hz, 1H), 6.24 (d, *J* = 2.0 Hz, 1H), 4.12 (dd, *J* = 9.5, 5.1 Hz, 1H), 4.08 (dd, *J* = 8.3, 5.4 Hz, 1H), 3.08 (s, 3H), 3.06 (s, 3H), 2.73 (dd, *J* = 14.3, 5.1 Hz, 1H), 2.69 (dd, *J* = 14.5, 5.4 Hz, 1H), 2.50 (dd, *J* = 14.9, 8.6 Hz, 1H), 2.47 (s, 3H), 2.46 (s, 3H), 2.41 (dd, *J* = 14.3, 9.5 Hz, 1H), 1.60 (s, 3H), 1.49 (s, 3H).

^{13}C NMR (151 MHz, CD_3OD, 298 K, δ): 175.5, 175.4, 172.7, 172.6, 169.10, 169.06, 156.4, 156.3, 154.1, 153.9, 150.6, 146.10, 146.08, 141.5, 140.3, 140.2, 139.70, 139.68, 138.69, 136.34, 136.30, 136.1, 136.0, 133.4, 132.6, 132.5, 132.3, 132.2, 128.93, 128.90, 128.88, 128.86, 127.54, 127.47, 126.9, 126.8, 125.71, 125.65, 125.55, 123.41, 123.39, 122.61, 122.58, 120.59, 120.4, 117.7, 117.5, 54.9, 54.8, 38.65, 38.62, 37.3, 37.0, 35.12, 35.06, 22.4, 22.2.

^{19}F NMR (565 MHz, CD_3OD, 298 K, δ): –76.9 (br).

^{77}Se NMR (115 MHz, CD_3OD, 298 K, δ): 511.7.

HRMS ESI-pos (m/z) calc'd for $C_{31}H_{29}N_4O_4Se^+$ $[M]^+$, 601.1349; found, 601.1352. Deviation: –0.5 ppm.

HRMS ESI-neg (m/z) calc'd for $C_2O_2F_3^-$ $[M]^-$, 112.9856; found, 112.9857. Deviation: –0.9 ppm.

Preparation of NAc-Tyr-NH_2 derived DBSePy-oxa-selenonium salt 15

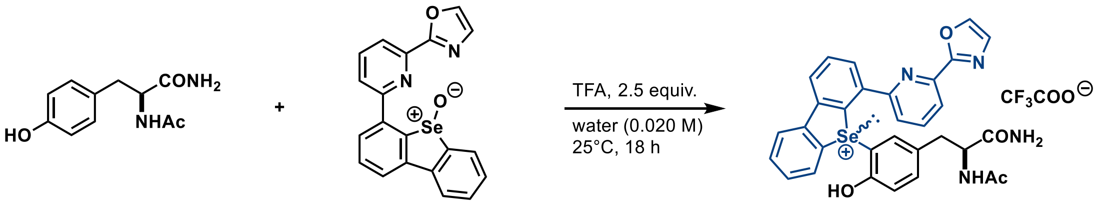

2 **1** **86%** **15**

Under ambient atmosphere, a round-bottom flask (250 mL) equipped with Teflon-coated magnetic stirring bar was charged with the the selenoxide **1** (390 mg, 1.0 mmol, 1.0 equiv.) and the solvent UHQ-H_2O (50 mL, c = 20 mM). The mixture was stirred at 25 °C for 2 min to give a white suspension. Then, trifluoroacetic acid (0.28 mg, 0.19 mL, 2.5 mmol, 2.5 equiv.) was added and the mixture was stirred for another 2 min to give a clear, yellow solution. Subsequently, the tyrosinamide **2** (270 mg, 1.2 mmol, 1.2 equiv.) was added and the mixture was stirred at 25 °C for 18 h. Subsequently, the mixture was transferred to a lyophilizing flask (500 mL), diluted by MeCN (50 mL), followed by the addition of a solution of Na_2SO_3 (c = 0.50 M, 1.0 mL, 0.50 mmol, 63 mg, 0.50 equiv.) in UHQ-H_2O. The mixture was stirred at 25 °C for 5 min and was further diluted by UHQ-H_2O (ca. 200 mL) and lyophilized. The residue was purified by the column chromatography on silica gel. Firstly, the eluent MeCN/MeOH (9/1–7/3) was used to remove selenide and unreacted tyrosinamide. Then, the column chromatography was allowed to dry under argon flow and re-equibriated by DCM/MeOH (9/1). Subsequently, the second eluent DCM/MeOH (9/1–4/1) + 1%(v/v) TFA was used to obtain fractions containing the selenonium salt. The desired fractions were combined and concentrated to ca. 10 mL under reduced pressure to give a syrup-like, pale yellow liquid. The syrup-like liquid was diluted by UHQ-H_2O (ca. 100 mL) and the mixture was lyophilized to afford the desired compound as pale pink powder (610 mg, 86%).

R_f = 0.22 (silica gel, DCM/MeOH = 4/1).

NMR Spectroscopy (mixture of a pair of epimer, ratio 1/1):

^{1}H NMR (600 MHz, CD_3OD, 298 K, δ): 8.71 (dd, *J* = 4.1, 1.0 Hz, 1H), 8.70 (dd, *J* = 4.1, 1.0 Hz, 1H), 8.61 (dd, *J* = 8.1, 0.9 Hz, 1H), 8.59 (dd, *J* = 8.1, 0.9 Hz, 1H), 8.52 (d, *J* = 1.1 Hz, 1H), 8.50 (d, *J* = 1.0 Hz, 1H), 8.37 (td, *J* = 8.0, 1.0 Hz, 2H), 8.28 (dd, *J* = 7.7, 1.2 Hz, 2H), 8.26 – 8.15 (m, 6H), 8.08 (d, *J* = 0.7 Hz, 1H), 8.07 (d, *J* = 0.7 Hz, 1H), 7.79 – 7.73 (m, 2H), 7.67 – 7.62 (m, 2H), 7.40 (d, *J* = 0.7 Hz, 1H), 7.39 (d, *J* = 0.7 Hz, 1H), 7.11 (t, *J* = 2.1 Hz, 1H), 7.10 (t, *J* = 2.1 Hz, 1H), 6.95 (d, *J* = 1.3 Hz, 1H), 6.93 (d, *J* = 1.2 Hz, 1H), 6.23 (d, *J* = 2.1 Hz, 1H), 6.19 (d, *J* = 2.0 Hz, 1H), 4.09 (dd, *J* = 8.7, 5.3 Hz, 1H), 4.02 (dd, *J* = 9.7, 5.2 Hz, 1H), 2.69 (dd, *J* = 14.6, 5.4 Hz, 1H), 2.66 (dd, *J* = 14.8, 5.5 Hz, 1H), 2.41 (dd, *J* = 14.5, 8.7 Hz, 1H), 2.32 (dd, *J* = 14.3, 9.7 Hz, 1H), 1.52 (s, 3H), 1.45 (s, 3H).

^{13}C NMR (151 MHz, CD_3OD, 298 K, δ): 175.5, 175.4, 172.5, 172.4, 160.61, 160.56, 156.58, 156.57,

151.3, 151.2, 146.07, 146.02, 145.4, 145.3, 142.2, 142.1, 141.5, 140.3, 140.2, 140.1, 140.0, 138.8, 138.7, 136.22, 136.18, 135.8, 135.7, 133.43, 133.41, 132.2, 132.10, 132.07, 131.99, 130.13, 130.09, 129.7, 129.6, 129.04, 128.97, 127.7, 127.6, 126.3, 126.0, 125.9, 125.6, 125.5, 123.9, 123.8, 123.2, 120.7, 120.6, 117.30, 117.25, 54.8, 54.5, 37.4, 37.2, 22.2, 22.1.

^{19}F NMR (565 MHz, CD_3OD, 298 K, δ): –77.1 (br).

^{77}Se NMR (115 MHz, CD_3OD, 298 K, δ): 525.0, 524.7.

HRMS ESI-pos (m/z) calc'd for $C_{31}H_{25}N_4O_4Se^+$ $[M]^+$, 597.1036; found, 597.1043. Deviation: –1.2 ppm.

HRMS ESI-neg (m/z) calc'd for $C_2O_2F_3^-$ $[M]^-$, 112.9856; found, 112.9855. Deviation: 0.9 ppm.

Initial study on functionalization of model compound 2

Results of model compound 2 functionalization by sulfoxides and selenoxides

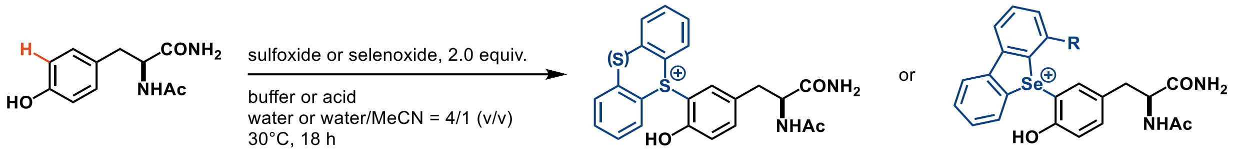

2, 2.5 mM   sulfonium product   selenonium product

sulfoxides:

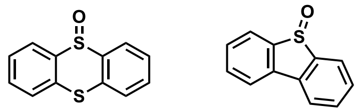

3   **DBTO**

selenoxides:

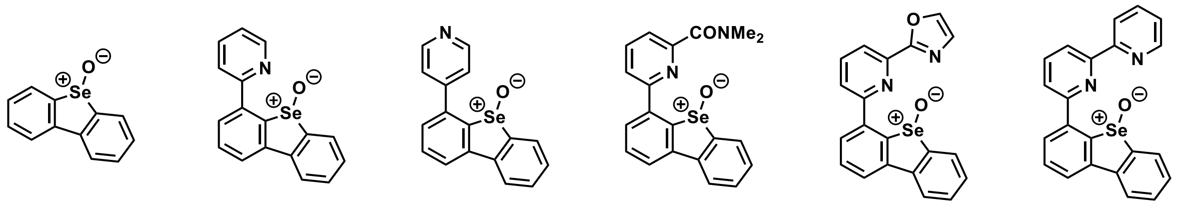

4   **5**   **S5**   **6**   **1**   **S11**

Entry	Sulfoxide or selenoxide	Buffer (pH, concentration[a])	Yield of product[b]
1[c]	**3**	H_2SO_4 (pH 1.0, 50 mM)	<2%
2[c]	**DBTO**	H_2SO_4 (pH 1.0, 50 mM)	<2%
3	**4**	H_2SO_4 (pH 1.0, 50 mM)	18%
4	**4**	Na-citrate (pH 3.0, 100 mM)	<2%
5	**5**	Na-citrate (pH 3.0, 100 mM)	32%
6	**S5**	Na-citrate (pH 3.0, 100 mM)	<2%
7	**6**	Na-citrate (pH 3.0, 100 mM)	45%
8	**1**	Na-citrate (pH 3.0, 100 mM)	88%
9	**1**	Na-acetate (pH 4.5, 100 mM)	27%
10	**1**	NaPi (pH 6.0, 100 mM)	<2%
11	**S11**	Na-citrate (pH 3.0, 100 mM)	<2%

Table 1. Functionalization of the model compound **2** by sulfoxides and selenoxides. a) Final concentration in reaction mixture. b) ^1H-NMR yield (average of 3 parallel experiments) using CH_2Br_2 as internal standard. c) Reaction mixture contains MeCN 20% (v/v) as cosolvent and the reaction time was elongated to 24 h.

Our previous study showed that the combination of thianthrene *S*-oxide **3** (1.0 equiv.) and $HBF_4{\cdot}Et_2O$ (2.0 equiv.) in MeCN leads to the functionalization of tert-butylbenzene (in 21% yield)[7], possibly by the protonated **3**. However, such functionalization of the more electron-rich substrate **2**, cannot be achieved under aqueous conditions due to the insufficient basicity of sulfoxides (pK_b of sulfoxides is typically negative in water[8]). Both sulfoxide **3** and dibenzothiophene *S*-oxide (**DBTO**) remained inert towards **2** in the acidic aqueous solution of pH 1.0, even after 24 h at 30 °C.

Solubility of 1 in water at different pH

To evaluate the solubility of selenoxide **1** in aqueous solutions of different pH, **1** (5.0 µmol, 2.0 mg, 1.0 equiv., final concentration c = 10 mM) was mixed with buffer solutions (c = 100 mM) of different pH at 37 °C for 15 min. To the resulting mixture, 50 µL of a stock solution of 3-(Trimethylsilyl)propionic-2,2,3,3-d_4 acid sodium salt (c = 0.10 M, 5.0 µmol, 0.86 mg, 1.0 equiv.) in D_2O was added as internal standard. The mixture was analyzed via ^{1}H-NMR and a singlet peak at 0.00 ppm (internal standard) was set as 9.00. The concentration of **1** (c_1(buffer)) in the solution was determined as following. The solubility of **1** at pH 3.0 is more than 10 mM. The solubility of the large hydrophobic, nonprotonated **1** in water could be attributed to its high polarity (dipole moment = 6.64 Debye, see the DFT computation Section).

c_1(Na-citrate buffer, pH 3.0) = integration of peak [δ: 7.31 (s, 1H)] × 10 mM = 10 mM.

c_1(Na-acetate buffer, pH 4.5) = integration of peak [δ: 7.43 (s, 1H)] × 10 mM = 4.5 mM.

c_1(NaPi buffer, pH 6.0) = integration of peak [δ: 7.44 (s, 1H)] × 10 mM = 3.1 mM.

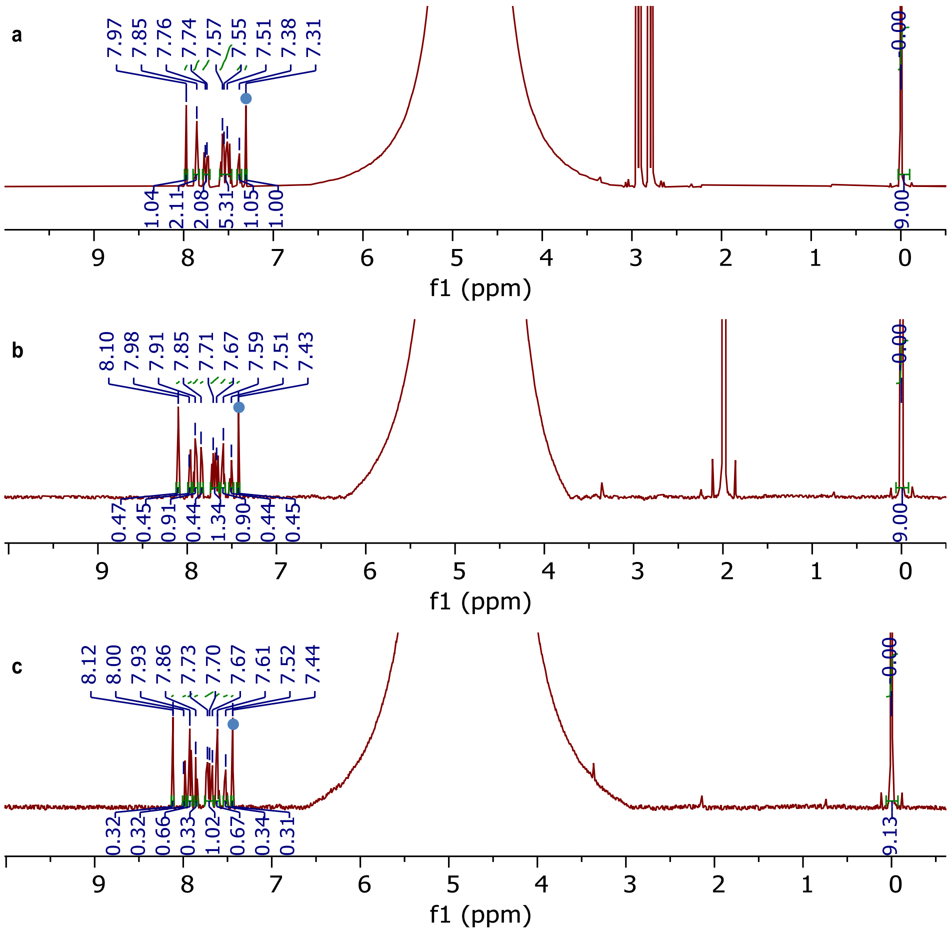

Figure 1. ^{1}H-NMR spectra of the selenoxide **1** solution in buffers with different pH: a) in Na-citrate (pH=3.0), b) in Na-acetate (pH=4.5), c) in NaPi (pH=6.0). Blue spots arethe peaks used to determine the amount of dissolved selenoxide **1**.

Titration study of selenoxides by UV-Vis spectrometry

Mathematical fundamental for pK_a calculation from titration study

Considering that selenoxides are typically weak bases, the aqueous solution of a selenoxide should consist of both selenoxide and its protonated form. Thus, the measured absorption (*A*) of the solution at a certain wavelength and certain pH is the sum of the absorption of the protonated selenoxide (A_{SeOH+}) and the absorption of its nonprotonated form (A_{SeO}). Following Lambert-Bill's law, at any step during the titration, the measured absorption can be expressed:

$$A = A_{\mathrm{SeOH+}} + A_{\mathrm{SeO}} = l \cdot (\varepsilon_{\mathrm{SeOH+}}[\mathrm{SeOH^+}] + \varepsilon_{\mathrm{SeO}}[\mathrm{SeO}]) \quad (1)$$

l, length of light pathway; ε_{SeOH+}, extinction coefficient of protonated selenoxide; ε_{SeO}, extinction coefficient of nonprotonated selenoxide; [SeOH$^+$], concentration of protonated selenoxide; [SeO], concentration of nonprotonated selenoxide

According to the distribution coefficient in aqueous solution, the concentration of nonprotonated and protonated selenoxides can be expressed:

$$[\mathrm{SeOH^+}] = \frac{[\mathrm{H^+}]}{K_a + [\mathrm{H^+}]} \cdot c' \quad (2)$$

$$[\mathrm{SeO}] = \frac{K_a}{K_a + [\mathrm{H^+}]} \cdot c' \quad (3)$$

K_a, dissociation constant of protonated selenoxide; *c′*, the total concentration of selenoxide and protonated selenoxide at a certain titration step; [H$^+$], proton concentration at the titration step

Based on the equation (3), when K_a/[H$^+$] > 1000, the [SeO]/*c′* × 100% > 99.9%, which allows the approximation that the measured absorption of the solution is only contributed by the absorption of nonprotonated selenoxide. At this point, the measured absorption is denoted as A_{basic} and A_{basic} can be expressed:

$$A_{\mathrm{basic}} \approx A_{\mathrm{SeO}} = l \cdot \varepsilon_{\mathrm{SeO}} \cdot c' \quad (4)$$

Substitute equation (4) to (1):

$$A - A_{\mathrm{basic}} = l \cdot (\varepsilon_{\mathrm{SeOH+}} - \varepsilon_{\mathrm{SeO}}) \cdot [SeOH^+] \quad (5)$$

Substitute equation (2) to (5):

$$A - A_{\mathrm{basic}} = l \cdot (\varepsilon_{\mathrm{SeOH+}} - \varepsilon_{\mathrm{SeO}}) \cdot c' \cdot \frac{[\mathrm{H^+}]}{K_a + [\mathrm{H^+}]} \quad (6)$$

Now consider that the concentration of selenoxide (protonated and nonprotonated) *c* changes during the titration because the titrant added changes the volume of the solution, so the equation (6) can be rewritten:

$$(A - A_{\mathrm{basic}}) \cdot \frac{V}{V_0} = l \cdot (\varepsilon_{\mathrm{SeOH+}} - \varepsilon_{\mathrm{SeO}}) \cdot c_0 \cdot \frac{[\mathrm{H^+}]}{K_a + [\mathrm{H^+}]} \quad (7)$$

c_0, the concentration of selenoxide (nonprotonated and protonated) before the titration starts; V_0, the volume of the solution before the titration starts; *V*, the volume of the solution at the titration step

If the solution satisfies the requirement K_a/[H$^+$] > 1000 before the titration starts, the measured absorption

of the solution before titration starts (denoted as A_0) can be used to substitute $A_{basic} \times V/V_0$, following Lambert-Bill's law:

$$A \cdot \frac{V}{V_0} - A_0 = l \cdot (\varepsilon_{\mathrm{SeOH+}} - \varepsilon_{\mathrm{SeO}}) \cdot c_0 \cdot \frac{[\mathrm{H}^+]}{K_a + [\mathrm{H}^+]} \tag{8}$$

Set $A' = A \times V/V_0$:

$$A' - A_0 = l \cdot (\varepsilon_{\mathrm{SeOH+}} - \varepsilon_{\mathrm{SeO}}) \cdot c_0 \cdot \frac{[\mathrm{H}^+]}{K_a + [\mathrm{H}^+]} \tag{9}$$

Substitute [H$^+$] by $10^{-\mathrm{pH}}$, the equation (9) can be rewritten as:

$$A' = l \cdot (\varepsilon_{\mathrm{SeOH+}} - \varepsilon_{\mathrm{SeO}}) \cdot c_0 \cdot \frac{1}{K_a \cdot 10^{\mathrm{pH}} + 1} + A_0 \tag{10}$$

Plot *A′* against pH and perform nonlinear fitting of the data points by Origin 2019b®[9] with the expression:

$$A' = a \cdot \frac{1}{b \cdot 10^{\mathrm{pH}} + 1} + c \tag{11}$$

The pK_a of the protonated selenoxide can be calculated:

$$p\mathrm{K_a} = -\log(b) \tag{12}$$

General consideration and experiment design

Buffer and titrant selection*:* Based on the discussion above, *A* (absorption of the selenoxide solution at the wavelength) and the corresponding titrand solution pH and volume at each titration step are necessary for pK_a calculation. Given that the pK_a of protonated dimethylselenoxide is 2.55[10], we decided to set the start point of the titration at pH 6.0, which could satisfy the requirement $K_a/[H^+] > 1000$. To obtain more absorption values (*A*) at different pH, we select Na-citrate buffer as the titration medium due to its high buffer capacity which allows the small pH change during each titration step over a broad range (pH 1–6). Besides, we selected aqueous solution of sulfuric acid rather than commonly used hydrochloric acid as the titrant to avoid the unwanted oxidation of chloride anion by selenoxide at low pH.

Wavelength selection*:* To minimize the deviation during the absorption measurement, the large difference between ε_{SeOH+} and ε_{SeO} was preferred. Therefore, the wavelength (above 300 nm) at which the absorption of the selenoxide solution showed largest change during titration was selected.

Concentration*:* To minimize the deviation during the absorption measurement, higher concentration of selenoxide was recommended, but the absorption at the selected wavelength should not exceed 1.000.

pH Measurement*:* Considering that the volume of a cuvette is small (3 mL) which could be difficult for direct pH measurement, we decided to perform the parallel titration with pH meter in a large scale (10 mL) and plot the pH against the amount of the added titrant. Then, we performed the titration but in a smaller scale (2.0 mL) in cuvette, and recorded the absorption spectrum of each step by UV-vis spectrophotometer. Based on the correlation between pH and titrant amount added, we could build the correlation between pH and absorption spectra, thus the correlation between pH and *A* (finally *A′*, corrected by the titrand volume *V* at each step) at a selected wavelength.

Titration curve of 50 mM Na-citrate buffer (pH = 6.0)

Under ambient atmosphere, a scintillation vial (20 mL) equipped with Teflon-coated magnetic stirring bar and a pH meter was charged with 1.0 mL of Na-citrate buffer (pH 6.0, c = 500 mM, final concentration c = 50 mM) and 8.8 mL UHQ-H_2O. Next, 200 µL of a phosphoric acid solution (c = 20 mM, 4.0 µmol, final concentration c = 0.40 mM) in UHQ-H_2O was added and the solution was stirred at 25 °C for 1 min. The value read from pH meter was recorded as start pH. Then, 20 µL of the titrant, a sulfuric acid solution (c = 2.0 M, 40 µmol) in UHQ-H_2O was added. The solution was stirred at 25 °C for 1 min and the pH value was recorded. This step was repeated for 50 times and the entire titration process was repeated for three times. The average of the obtained pH in each step was plotted against the amount of titrant in the titrand solution before next titration step to give a titration curve.

**Titrant amount (mmol)**	pH			
	1st measurement	2nd measurement	3rd measurement	**Average**
**0**	6.51	6.50	6.52	**6.51**
**0.04**	6.11	6.10	6.13	**6.11**
**0.08**	5.82	5.84	5.86	**5.84**
**0.12**	5.60	5.61	5.64	**5.62**
**0.16**	5.38	5.41	5.44	**5.41**
**0.20**	5.17	5.21	5.23	**5.20**
**0.24**	4.97	5.00	5.04	**5.00**
**0.28**	4.76	4.81	4.84	**4.80**
**0.32**	4.57	4.61	4.65	**4.61**
**0.36**	4.37	4.43	4.46	**4.42**
**0.40**	4.18	4.24	4.27	**4.23**
**0.44**	3.97	4.05	4.08	**4.03**
**0.48**	3.76	3.85	3.88	**3.83**
**0.52**	3.54	3.64	3.67	**3.62**
**0.56**	3.33	3.44	3.46	**3.41**
**0.60**	3.13	3.24	3.26	**3.21**
**0.64**	2.94	3.06	3.07	**3.02**
**0.68**	2.76	2.88	2.89	**2.84**
**0.72**	2.59	2.70	2.72	**2.67**
**0.76**	2.42	2.54	2.55	**2.50**
**0.80**	2.27	2.38	2.39	**2.35**
**0.84**	2.14	2.24	2.25	**2.21**
**0.88**	2.03	2.12	2.13	**2.09**
**0.92**	1.94	2.02	2.02	**1.99**
**0.96**	1.86	1.92	1.93	**1.90**
**1.00**	1.78	1.84	1.85	**1.82**
**1.04**	/ [a]	1.77	1.78	**1.78**[b]
**1.08**	1.66	1.71	1.72	**1.70**
**1.12**	1.61	1.66	1.66	**1.64**
**1.16**	1.54	1.62	1.62	**1.59**
**1.20**	1.48	1.56	1.57	**1.54**
**1.24**	1.44	1.53	1.53	**1.50**
**1.28**	1.41	1.49	1.49	**1.46**
**1.32**	1.38	1.46	1.46	**1.43**
**1.36**	1.35	1.42	1.42	**1.40**
**1.40**	1.32	1.39	1.39	**1.37**

**1.44**	1.30	1.37	1.36	**1.34**
**1.48**	1.27	1.34	1.34	**1.32**
**1.52**	1.25	1.32	1.31	**1.29**
**1.56**	1.23	1.29	1.29	**1.27**
**1.60**	1.21	1.27	1.27	**1.25**
**1.64**	1.19	1.25	1.25	**1.23**
**1.68**	1.17	1.23	1.23	**1.21**
**1.72**	1.15	1.21	1.21	**1.19**
**1.76**	1.14	1.19	1.19	**1.17**
**1.80**	1.12	1.18	1.18	**1.16**
**1.84**	1.10	1.16	1.16	**1.14**
**1.88**	1.08	1.15	1.14	**1.12**
**1.92**	1.07	1.13	1.13	**1.11**
**1.96**	1.05	1.12	1.12	**1.10**
**2.00**	1.04	1.10	1.10	**1.08**
**2.20**	0.98	1.05	1.05	**1.03**
**2.60**	0.89	0.95	0.95	**0.93**
**3.40**	0.76	0.82	/ [a]	**0.79** [b]
**5.00**	0.61	0.66	0.66	**0.64**
**7.00**	0.54	0.54	0.54	**0.54**
**10.0**	0.43	0.43	0.43	**0.43**

Table 2. Results of 10 mL Na-citrate (pH 6.5, 50 mM, containing H_3PO_4 0.4 mM) titrated by H_2SO_4 (2.0 M). a) Data not recorded b) Average of two measurements.

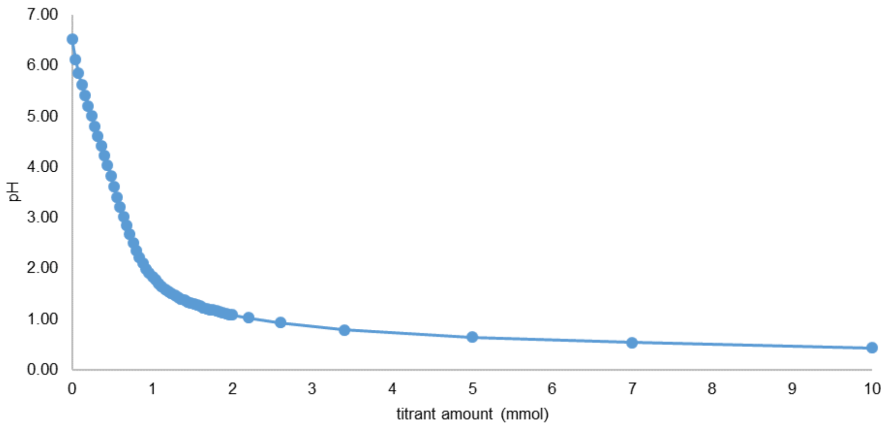

Figure 2. Titration curve of 10 mL Na-citrate (pH 6.5, 50 mM). Titrant: H_2SO_4 (2.0 M).

General procedure for titration in cuvette and UV-vis spectrometry

***Blank**:* Under ambient atmosphere, to a quartz cuvette (3.5 mL) equipped with Teflon-coated mini magnetic stirring bar 200 µL of Na-citrate buffer (pH 6.0, c = 500 mM, final concentration c = 50 mM) and 1760 µL UHQ-H_2O were added. Next, 40 µL of a phosphoric acid solution (c = 20 mM, 0.80 µmol, final concentration c = 0.40 mM) in UHQ-H_2O was added and the solution was stirred at 25 °C for 1 min. The solution was used as a blank sample on a Shimadzu UV-vis Spectrophotometer UV-2600 at 25 °C.

***Measure**:* The same quartz cuvette (3.5 mL) equipped with Teflon-coated mini magnetic stirring bar was charged with 200 µL of Na-citrate buffer (pH 6.0, c = 500 mM, final concentration c = 50 mM) and 1760 µL UHQ-H_2O. Next, 40 µL of a selenoxide stock solution (c = 10 mM, 0.40 µmol, final concentration c = 0.20 mM) in phosphoric acid solution (c = 20 mM, 0.80 µmol, final concentration c = 0.40 mM) in UHQ-H_2O was added and the solution was stirred at 25 °C for 1 min. The absorption spectrum was recorded on a Shimadzu UV-vis Spectrophotometer UV-2600 at 25 °C. Then, a certain amount of the titrant, a sulfuric acid solution (c = 2.0 M) in UHQ-H_2O was added and the solution was stirred at 25 °C for 1 min. The absorption spectrum and the titrand solution volume as well as the total amount of the titrant in the titrand solution at the titration step were recorded. This step was repeated until the solution pH reached to ca. 1.0 (0.4 for titration study of **3**). The whole titration process was repeated for three times.

***Data process**:* For each titration step, the obtained absorption at the selected wavelength (*A*) was corrected by the titrand solution volume *V* at that step to give *A'*. Then, the total amount of titrant in the titrand solution before next step was multiplied by 5 and compared to the titrant amount in Table 2. The average pH in the entry of Table 2 which matches the 5-fold titrant amount in the titrand solution was used as the solution pH at that step. By plotting *A'* against pH and performing nonlinear fitting (described in mathematical fundamental), the pK_a of the protonated selenoxide can be determined. The reported pK_a of protonated selenoxide is an average of pK_a obtained in three repetitive titrations.

Titration study of selenoxide 4

Selected wavelength: 350 nm.

First measurement:

Titrant volume (µL)	Titrant amount (mmol)	pH[a]	*A* (350 nm)	*A'* (350 nm)[b]
**0**	0	**6.51**	0.082	**0.082**
**20**	0.040	**5.20**	0.083	**0.084**
**40**	0.080	**4.23**	0.081	**0.083**
**44**	0.088	**4.03**	0.084	**0.086**
**48**	0.096	**3.83**	0.085	**0.087**
**52**	0.104	**3.62**	0.086	**0.088**
**56**	0.112	**3.41**	0.088	**0.090**
**60**	0.120	**3.21**	0.090	**0.093**
**64**	0.128	**3.02**	0.095	**0.098**
**68**	0.136	**2.84**	0.099	**0.102**
**72**	0.144	**2.67**	0.106	**0.110**
**76**	0.152	**2.50**	0.112	**0.116**
**80**	0.160	**2.35**	0.125	**0.130**
**84**	0.168	**2.21**	0.134	**0.140**
**88**	0.176	**2.09**	0.144	**0.150**

**92**	0.184	**1.99**	0.152	**0.159**
**100**	0.200	**1.82**	0.169	**0.177**
**104**	0.208	**1.78**	0.175	**0.184**
**112**	0.224	**1.64**	0.188	**0.199**
**128**	0.256	**1.46**	0.205	**0.218**
**160**	0.320	**1.25**	0.224	**0.242**
**200**	0.400	**1.08**	0.231	**0.254**
**260**	0.520	**0.93**	0.243	**0.275**
**340**	0.680	**0.79**	0.244	**0.285**
**500**	1.00	**0.64**	0.235	**0.294**
**700**	1.40	**0.54**	0.225	**0.304**
**1000**	2.00	**0.43**	0.205	**0.308**

Table 3. Titration results of the **4** solution (c = 0.20 mM) in Na-citrate buffer (pH 6.5, 50 mM, 2.0 mL, containing H_3PO_4 0.40 mM). Titrant: H_2SO_4 (2.0 M). a) From the average pH of Table 2. b) For the calculation of *A'* see previous sections.

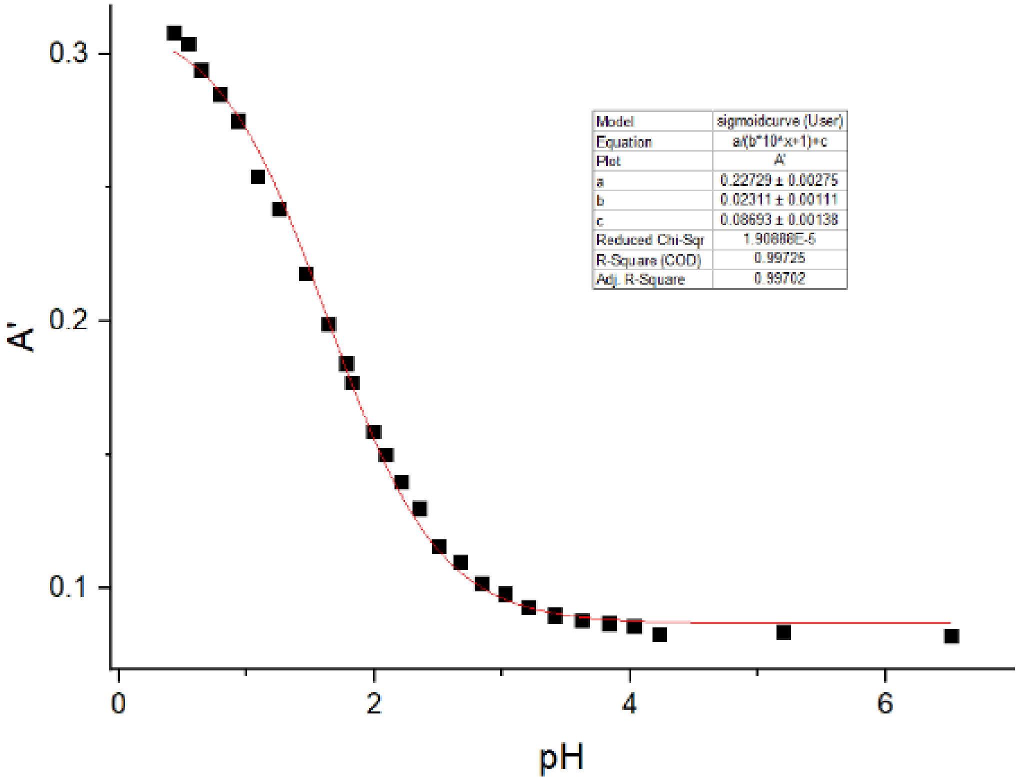

Figure 3. Absorption-pH plots (black dots) and the fitting curve (red line) of **4** titration (first measurement), meaning of parameters *a*, *b*, and *c* see previous sections.

$pK_a = -\log(b) = 1.64$

Second measurement:

Titrant volume (µL)	Titrant amount (mmol)	pH	*A* (350 nm)	*A'* (350 nm)
**0**	0	**6.51**	0.079	**0.079**
**20**	0.040	**5.20**	0.078	**0.079**
**40**	0.080	**4.23**	0.079	**0.081**
**44**	0.088	**4.03**	0.080	**0.082**
**48**	0.096	**3.83**	0.080	**0.082**
**52**	0.104	**3.62**	0.079	**0.081**
**56**	0.112	**3.41**	0.084	**0.086**

**60**	0.120	**3.21**	0.086	**0.089**
**64**	0.128	**3.02**	0.090	**0.093**
**68**	0.136	**2.84**	0.095	**0.098**
**72**	0.144	**2.67**	0.101	**0.105**
**76**	0.152	**2.50**	0.110	**0.114**
**80**	0.160	**2.35**	0.118	**0.123**
**84**	0.168	**2.21**	0.129	**0.134**
**88**	0.176	**2.09**	0.138	**0.144**
**92**	0.184	**1.99**	0.148	**0.155**
**100**	0.200	**1.82**	0.164	**0.172**
**104**	0.208	**1.78**	0.171	**0.180**
**112**	0.224	**1.64**	0.183	**0.193**
**128**	0.256	**1.46**	0.197	**0.210**
**160**	0.320	**1.25**	0.220	**0.238**
**200**	0.400	**1.08**	0.232	**0.255**
**260**	0.520	**0.93**	0.240	**0.271**
**340**	0.680	**0.79**	0.241	**0.282**
**500**	1.00	**0.64**	0.235	**0.294**
**700**	1.40	**0.54**	0.222	**0.300**
**1000**	2.00	**0.43**	0.199	**0.299**

Table 4. Second titration results of the **4** solution (c = 0.20 mM) in Na-citrate buffer (pH 6.5, 50 mM, 2.0 mL, containing H_3PO_4 0.40 mM). Titrant: H_2SO_4 (2.0 M). Conditions are same to Table 3.

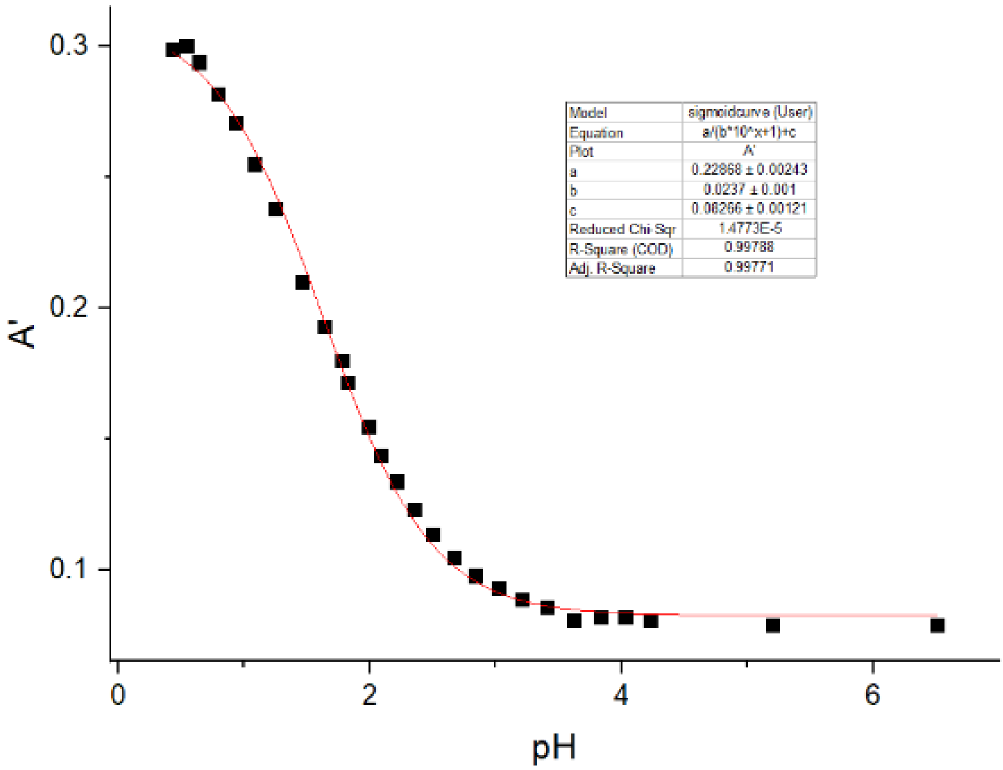

Figure 4. Absorption-pH plots (black dots) and the fitting curve (red line) of **4** titration (second measurement), meaning of parameters *a*, *b*, and *c* see previous sections.

$pK_a = -\log(b) = 1.63$

Third measurement:

**Titrant volume (μL)**	**Titrant amount (mmol)**	**pH**	***A* (350 nm)**	***A'* (350 nm)**

**0**	0	**6.51**	0.080	**0.080**
**20**	0.040	**5.20**	0.079	**0.080**
**40**	0.080	**4.23**	0.079	**0.081**
**44**	0.088	**4.03**	0.078	**0.080**
**48**	0.096	**3.83**	0.080	**0.082**
**52**	0.104	**3.62**	0.082	**0.084**
**56**	0.112	**3.41**	0.083	**0.085**
**60**	0.120	**3.21**	0.086	**0.089**
**64**	0.128	**3.02**	0.088	**0.091**
**68**	0.136	**2.84**	0.095	**0.098**
**72**	0.144	**2.67**	0.102	**0.106**
**76**	0.152	**2.50**	0.111	**0.115**
**80**	0.160	**2.35**	0.120	**0.125**
**84**	0.168	**2.21**	0.131	**0.137**
**88**	0.176	**2.09**	0.141	**0.147**
**92**	0.184	**1.99**	0.149	**0.156**
**100**	0.200	**1.82**	0.166	**0.174**
**104**	0.208	**1.78**	0.172	**0.181**
**112**	0.224	**1.64**	0.184	**0.194**
**128**	0.256	**1.46**	0.199	**0.212**
**160**	0.320	**1.25**	0.222	**0.240**
**200**	0.400	**1.08**	0.233	**0.256**
**260**	0.520	**0.93**	0.239	**0.270**
**340**	0.680	**0.79**	0.240	**0.281**
**500**	1.00	**0.64**	0.231	**0.289**
**700**	1.40	**0.54**	0.218	**0.294**
**1000**	2.00	**0.43**	0.199	**0.299**

Table 5. Third titration results of the **4** solution (c = 0.20 mM) in Na-citrate buffer (pH 6.5, 50 mM, 2.0 mL, containing H_3PO_4 0.40 mM). Titrant: H_2SO_4 (2.0 M). Conditions are same to Table 3.

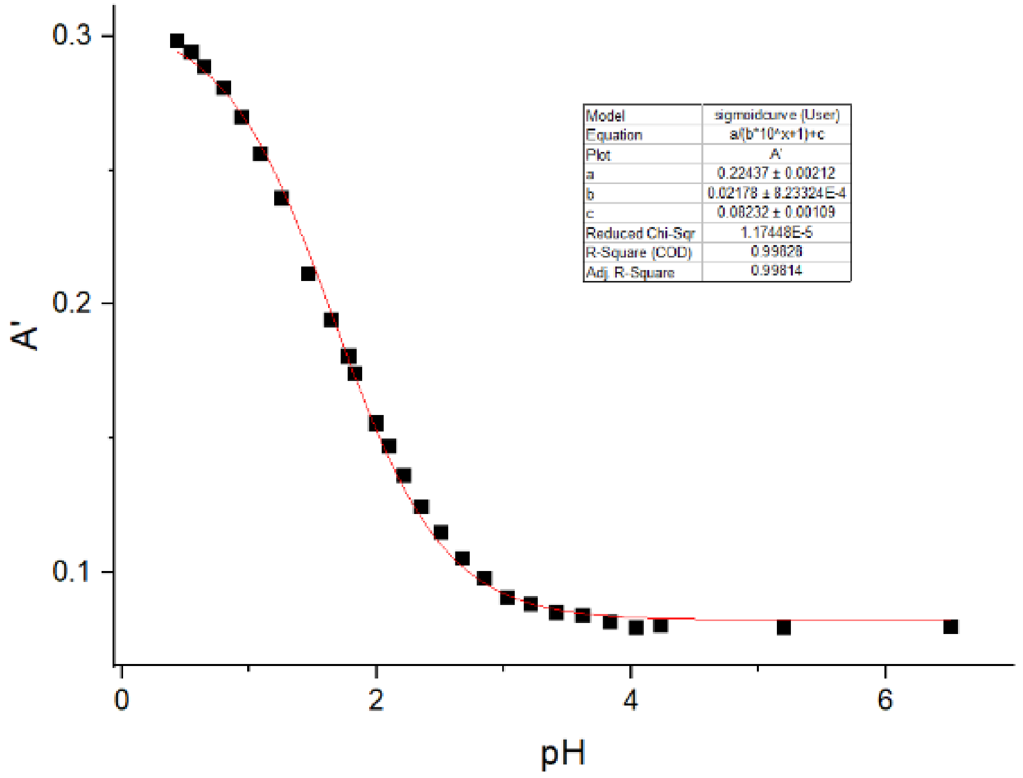

Figure 5. Absorption-pH plots (black dots) and the fitting curve (red line) of **4** titration (third measurement), meaning of parameters *a*, *b*, and *c* see previous sections.

$pK_a = -\log(b) = 1.66$.

Average pK_a = 1.64.

Titration study of selenoxide 5

Selected wavelength: 359 nm.

First measurement:

Titrant volume (µL)	Titrant amount (mmol)	pH[a]	*A* (359 nm)	*A′* (359 nm)[b]
**0**	0	**6.51**	0.198	**0.198**
**20**	0.040	**5.20**	0.196	**0.198**
**40**	0.080	**4.23**	0.200	**0.204**
**44**	0.088	**4.03**	0.201	**0.205**
**48**	0.096	**3.83**	0.205	**0.210**
**52**	0.104	**3.62**	0.211	**0.216**
**56**	0.112	**3.41**	0.219	**0.225**
**60**	0.120	**3.21**	0.231	**0.238**
**64**	0.128	**3.02**	0.246	**0.254**
**68**	0.136	**2.84**	0.262	**0.271**
**72**	0.144	**2.67**	0.279	**0.289**
**76**	0.152	**2.50**	0.297	**0.308**
**80**	0.160	**2.35**	0.312	**0.324**
**84**	0.168	**2.21**	0.325	**0.339**
**88**	0.176	**2.09**	0.333	**0.348**
**92**	0.184	**1.99**	0.339	**0.355**
**100**	0.200	**1.82**	0.347	**0.364**
**104**	0.208	**1.78**	0.350	**0.368**
**112**	0.224	**1.64**	0.354	**0.374**
**128**	0.256	**1.46**	0.358	**0.381**
**160**	0.320	**1.25**	0.359	**0.385**
**200**	0.400	**1.08**	0.355	**0.388**

Table 6. Titration results of the **5** solution (c = 0.20 mM) in Na-citrate buffer (pH 6.5, 50 mM, 2.0 mL, containing H_3PO_4 0.40 mM). Titrant: H_2SO_4 (2.0 M). a) From the average pH of Table 2. b) For the calculation of *A′* see previous sections.

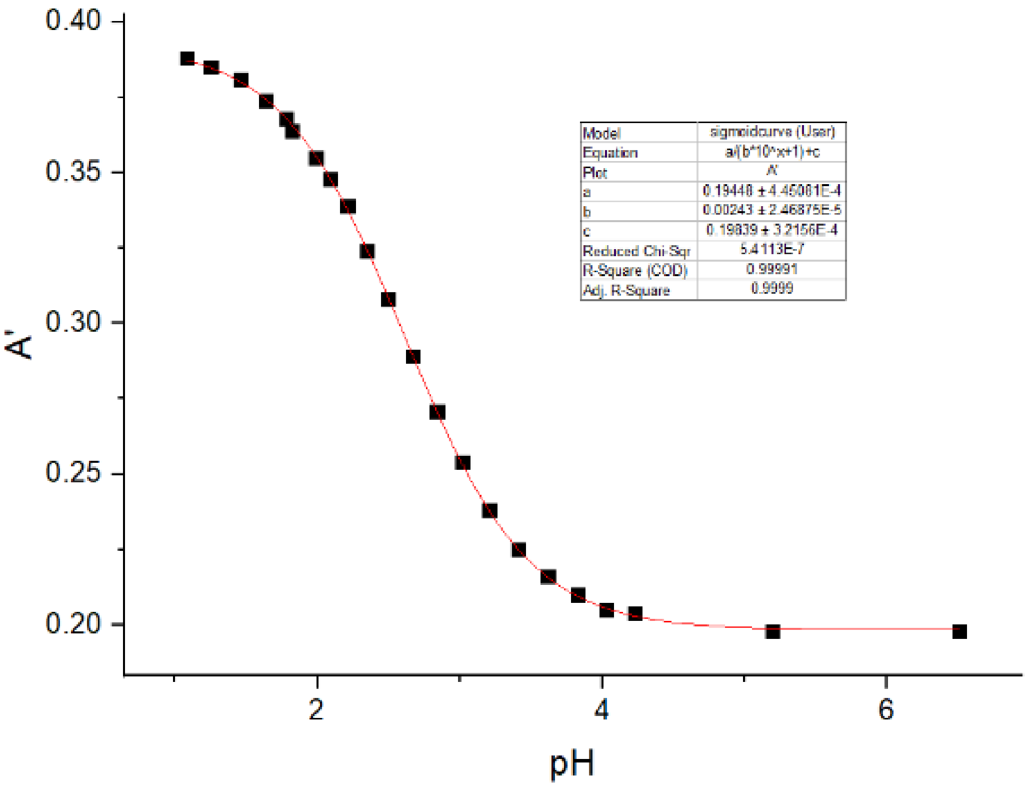

Figure 6. Absorption-pH plots (black dots) and the fitting curve (red line) of **5** titration (first measurement), meaning of parameters *a*, *b*, and *c* see previous sections.

$pK_a = -\log(b) = 2.61$.

Second measurement:

Titrant volume (μL)	Titrant amount (mmol)	pH	*A* (359 nm)	*A'* (359 nm)
**0**	0	**6.51**	0.185	**0.185**
**20**	0.040	**5.20**	0.179	**0.181**
**40**	0.080	**4.23**	0.186	**0.190**
**44**	0.088	**4.03**	0.187	**0.191**
**48**	0.096	**3.83**	0.189	**0.194**
**52**	0.104	**3.62**	0.197	**0.202**
**56**	0.112	**3.41**	0.203	**0.209**
**60**	0.120	**3.21**	0.217	**0.224**
**64**	0.128	**3.02**	0.233	**0.240**
**68**	0.136	**2.84**	0.249	**0.257**
**72**	0.144	**2.67**	0.266	**0.276**
**76**	0.152	**2.50**	0.283	**0.294**
**80**	0.160	**2.35**	0.297	**0.309**
**84**	0.168	**2.21**	0.307	**0.320**
**88**	0.176	**2.09**	0.315	**0.329**
**92**	0.184	**1.99**	0.322	**0.337**
**100**	0.200	**1.82**	0.329	**0.345**
**104**	0.208	**1.78**	0.333	**0.350**
**112**	0.224	**1.64**	0.332	**0.351**
**128**	0.256	**1.46**	0.341	**0.363**
**160**	0.320	**1.25**	0.340	**0.367**
**200**	0.400	**1.08**	0.338	**0.372**

Table 7. Second titration results of the **5** solution (c = 0.20 mM) in Na-citrate buffer (pH 6.5, 50 mM, 2.0 mL, containing H_3PO_4 0.40 mM). Titrant: H_2SO_4 (2.0 M). Conditions are same to Table 6.

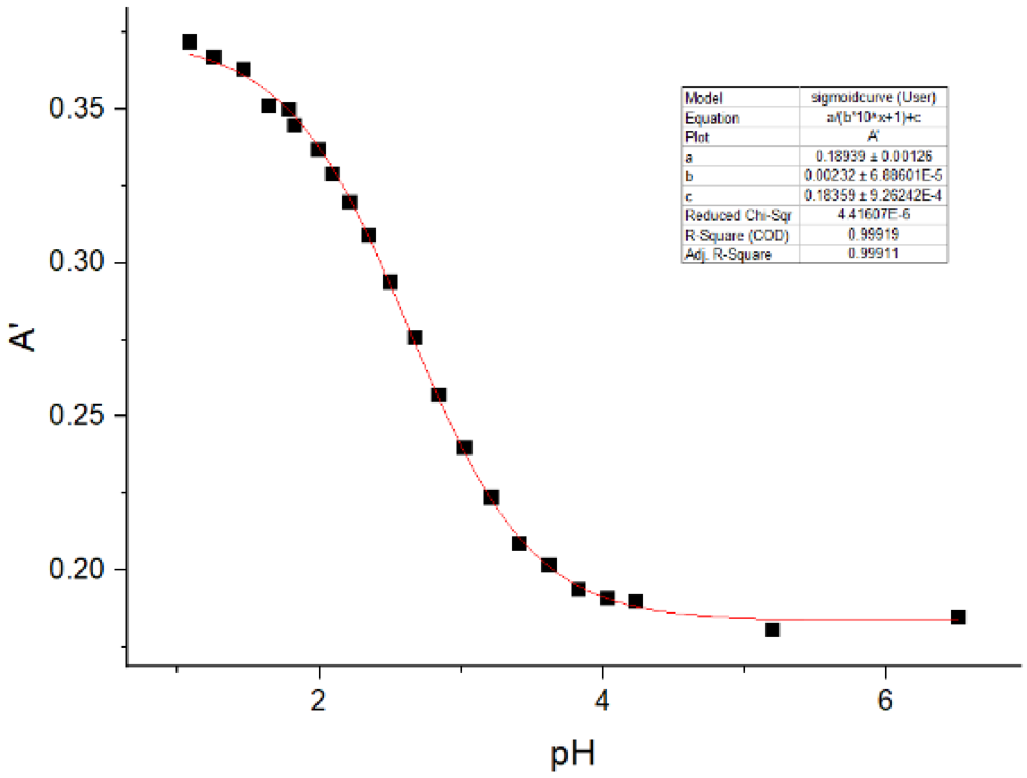

Figure 7. Absorption-pH plots (black dots) and the fitting curve (red line) of **5** titration (second measurement), meaning of parameters *a*, *b*, and *c* see previous sections.

$pK_a = -\log(b) = 2.63$

Third measurement:

**Titrant volume (μL)**	**Titrant amount (mmol)**	**pH**	***A* (359 nm)**	***A'* (359 nm)**
**0**	0	**6.51**	0.191	**0.191**
**20**	0.040	**5.20**	0.189	**0.191**
**40**	0.080	**4.23**	0.192	**0.196**
**44**	0.088	**4.03**	0.194	**0.198**
**48**	0.096	**3.83**	0.193	**0.198**
**52**	0.104	**3.62**	0.203	**0.208**
**56**	0.112	**3.41**	0.213	**0.219**
**60**	0.120	**3.21**	0.224	**0.231**
**64**	0.128	**3.02**	0.239	**0.247**
**68**	0.136	**2.84**	0.255	**0.264**
**72**	0.144	**2.67**	0.273	**0.283**
**76**	0.152	**2.50**	0.286	**0.297**
**80**	0.160	**2.35**	0.303	**0.315**
**84**	0.168	**2.21**	0.314	**0.327**
**88**	0.176	**2.09**	0.323	**0.337**
**92**	0.184	**1.99**	0.329	**0.344**
**100**	0.200	**1.82**	0.338	**0.355**
**104**	0.208	**1.78**	0.340	**0.358**
**112**	0.224	**1.64**	0.344	**0.363**

**128**	0.256	**1.46**	0.347	**0.369**
**160**	0.320	**1.25**	0.348	**0.376**
**200**	0.400	**1.08**	0.343	**0.377**

Table 8. Third titration results of the **5** solution (c = 0.20 mM) in Na-citrate buffer (pH 6.5, 50 mM, 2.0 mL, containing H_3PO_4 0.40 mM). Titrant: H_2SO_4 (2.0 M). Conditions are same to Table 6.

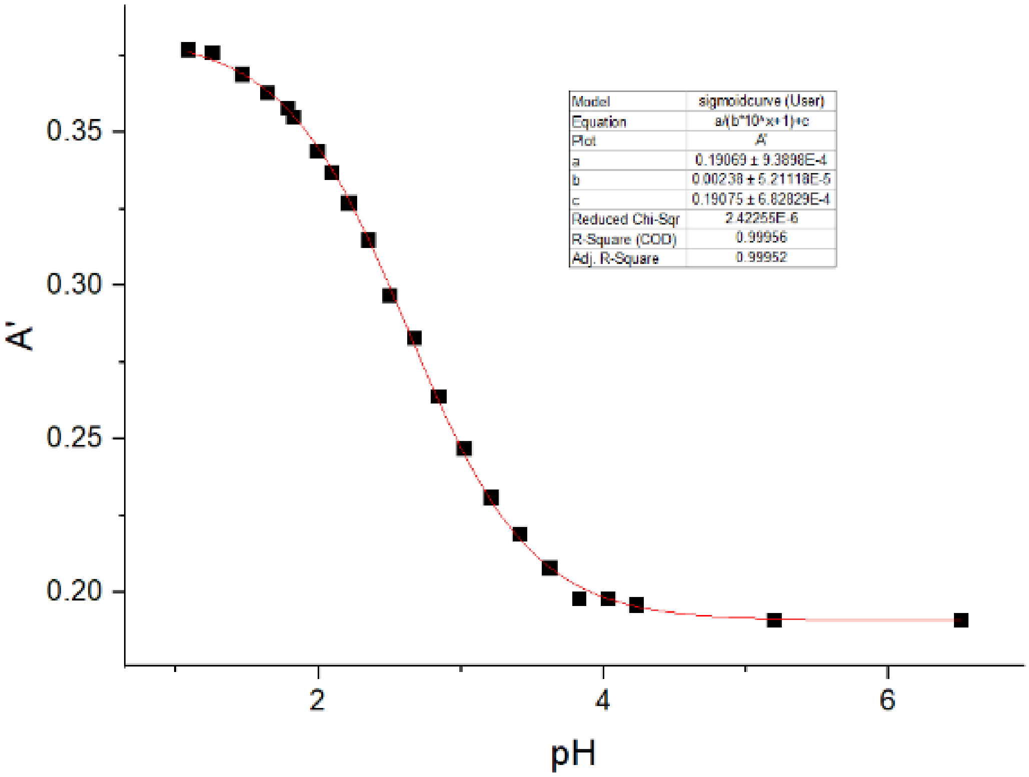

Figure 8. Absorption-pH plots (black dots) and the fitting curve (red line) of **5** titration (third measurement), meaning of parameters *a*, *b*, and *c* see previous sections.

pK_a = –log(*b*) = 2.62.

Average *p*K_a = 2.62.

Titration study of selenoxide 6

Selected wavelength: 365 nm.

First measurement:

Titrant volume (µL)	Titrant amount (mmol)	pH[a]	*A* (365 nm)	*A′* (365 nm)[b]
**0**	0	**6.51**	0.141	**0.141**
**20**	0.040	**5.20**	0.140	**0.141**
**40**	0.080	**4.23**	0.144	**0.147**
**44**	0.088	**4.03**	0.148	**0.151**
**48**	0.096	**3.83**	0.153	**0.157**
**52**	0.104	**3.62**	0.159	**0.163**
**56**	0.112	**3.41**	0.172	**0.177**
**60**	0.120	**3.21**	0.191	**0.197**
**64**	0.128	**3.02**	0.209	**0.216**
**68**	0.136	**2.84**	0.233	**0.241**
**72**	0.144	**2.67**	0.259	**0.268**
**76**	0.152	**2.50**	0.284	**0.295**
**80**	0.160	**2.35**	0.305	**0.317**
**84**	0.168	**2.21**	0.326	**0.340**
**88**	0.176	**2.09**	0.340	**0.355**
**92**	0.184	**1.99**	0.350	**0.366**
**100**	0.200	**1.82**	0.364	**0.382**
**104**	0.208	**1.78**	0.370	**0.389**
**112**	0.224	**1.64**	0.380	**0.401**
**128**	0.256	**1.46**	0.384	**0.409**
**160**	0.320	**1.25**	0.392	**0.423**
**200**	0.400	**1.08**	0.392	**0.431**

Table 9. Titration results of the **6** solution (c = 0.20 mM) in Na-citrate buffer (pH 6.5, 50 mM, 2.0 mL, containing H_3PO_4 0.40 mM). Titrant: H_2SO_4 (2.0 M). a) From the average pH of Table 2. b) For the calculation of *A′* see previous sections.

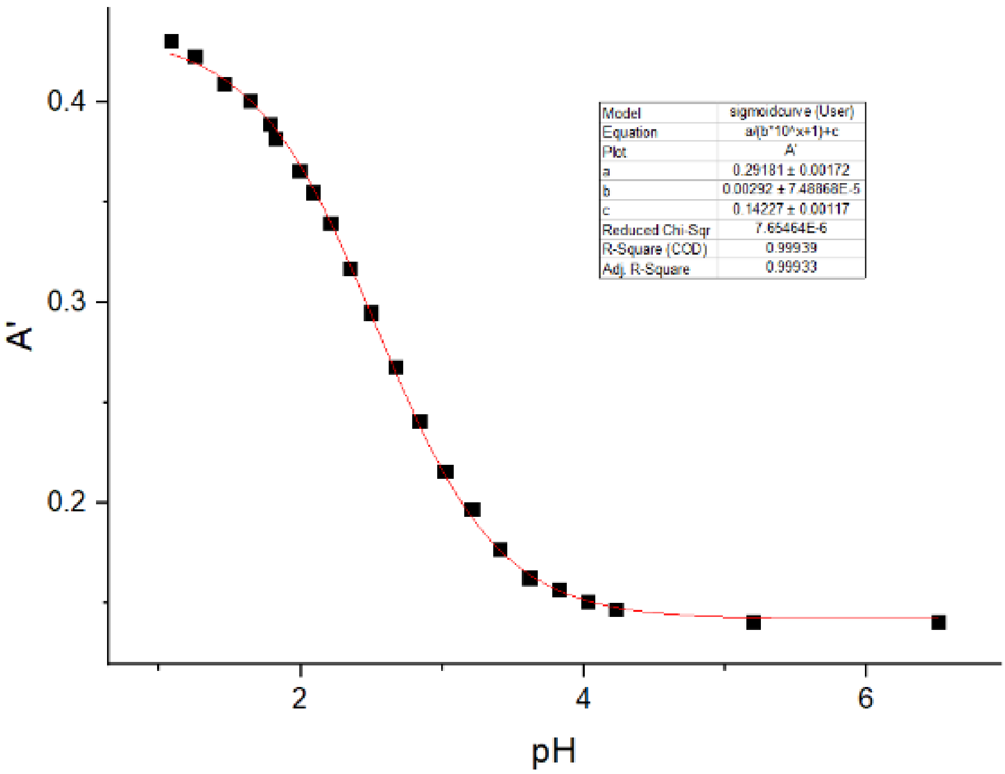

Figure 9. Absorption-pH plots (black dots) and the fitting curve (red line) of **6** titration (first measurement), meaning of parameters *a*, *b*, and *c* see previous sections.

$pK_a = -\log(b) = 2.53$.

Second measurement:

Titrant volume (µL)	Titrant amount (mmol)	pH	*A* (365 nm)	*A'* (365 nm)
**0**	0	**6.51**	0.132	**0.132**
**20**	0.040	**5.20**	0.132	**0.133**
**40**	0.080	**4.23**	0.135	**0.138**
**44**	0.088	**4.03**	0.139	**0.142**
**48**	0.096	**3.83**	0.142	**0.145**
**52**	0.104	**3.62**	0.149	**0.153**
**56**	0.112	**3.41**	0.160	**0.164**
**60**	0.120	**3.21**	0.174	**0.179**
**64**	0.128	**3.02**	0.192	**0.198**
**68**	0.136	**2.84**	0.215	**0.222**
**72**	0.144	**2.67**	0.236	**0.244**
**76**	0.152	**2.50**	0.261	**0.271**
**80**	0.160	**2.35**	0.282	**0.293**
**84**	0.168	**2.21**	0.302	**0.315**
**88**	0.176	**2.09**	0.316	**0.330**
**92**	0.184	**1.99**	0.328	**0.343**
**100**	0.200	**1.82**	0.342	**0.359**
**104**	0.208	**1.78**	0.348	**0.366**
**112**	0.224	**1.64**	0.356	**0.376**
**128**	0.256	**1.46**	0.365	**0.388**
**160**	0.320	**1.25**	0.370	**0.400**
**200**	0.400	**1.08**	0.369	**0.406**

Table 10. Second titration results of the **6** solution (c = 0.20 mM) in Na-citrate buffer (pH 6.5, 50 mM, 2.0 mL, containing H_3PO_4 0.40 mM). Titrant: H_2SO_4 (2.0 M). Conditions are same to Table 9.

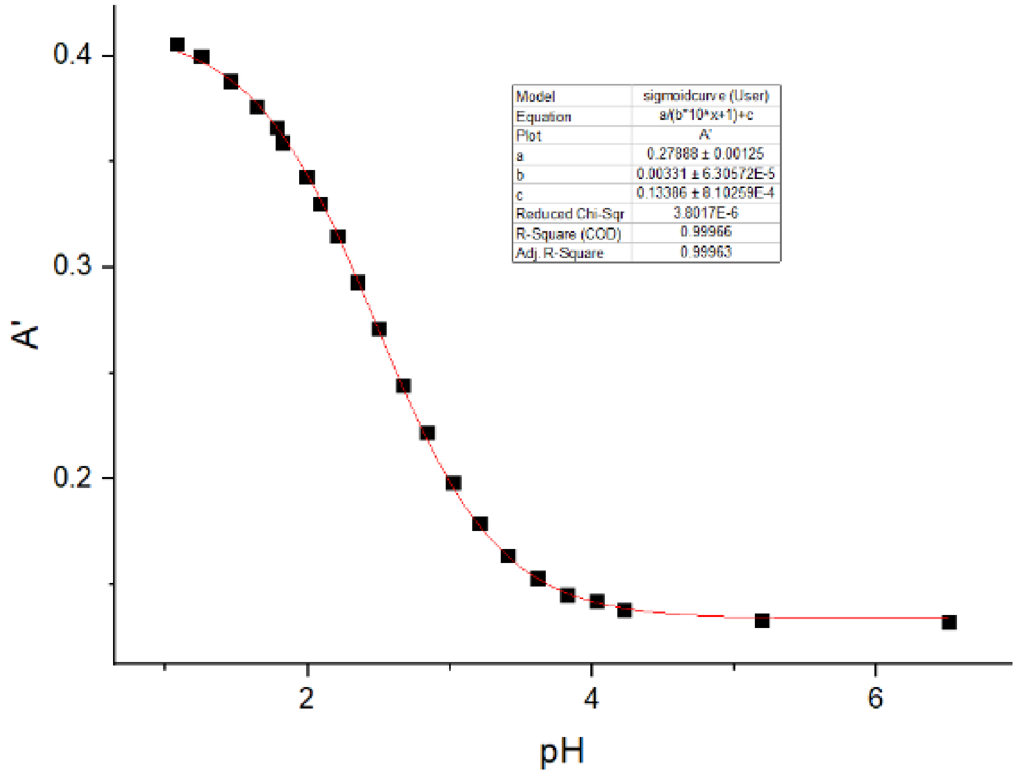

Figure 10. Absorption-pH plots (black dots) and the fitting curve (red line) of **6** titration (second measurement), meaning of parameters *a*, *b*, and *c* see previous sections.

$pK_a = -\log(b) = 2.48$

Third measurement:

Titrant volume (μL)	Titrant amount	pH	*A* (365 nm)	*A'* (365 nm)
**0**	0	**6.51**	0.128	**0.128**
**20**	0.040	**5.20**	0.128	**0.129**
**40**	0.080	**4.23**	0.134	**0.137**
**44**	0.088	**4.03**	0.136	**0.139**
**48**	0.096	**3.83**	0.142	**0.145**
**52**	0.104	**3.62**	0.151	**0.155**
**56**	0.112	**3.41**	0.162	**0.167**
**60**	0.120	**3.21**	0.178	**0.183**
**64**	0.128	**3.02**	0.197	**0.203**
**68**	0.136	**2.84**	0.216	**0.223**
**72**	0.144	**2.67**	0.242	**0.251**
**76**	0.152	**2.50**	0.267	**0.277**
**80**	0.160	**2.35**	0.286	**0.297**
**84**	0.168	**2.21**	0.302	**0.315**
**88**	0.176	**2.09**	0.315	**0.329**
**92**	0.184	**1.99**	0.324	**0.339**
**100**	0.200	**1.82**	0.337	**0.354**
**104**	0.208	**1.78**	0.342	**0.360**
**112**	0.224	**1.64**	0.348	**0.367**
**128**	0.256	**1.46**	0.354	**0.377**

**160**	0.320	**1.25**	0.359	**0.388**
**200**	0.400	**1.08**	0.359	**0.395**

Table 11. Third titration results of the **6** solution (c = 0.20 mM) in Na-citrate buffer (pH 6.5, 50 mM, 2.0 mL, containing H_3PO_4 0.40 mM). Titrant: H_2SO_4 (2.0 M). Conditions are same to Table 9.

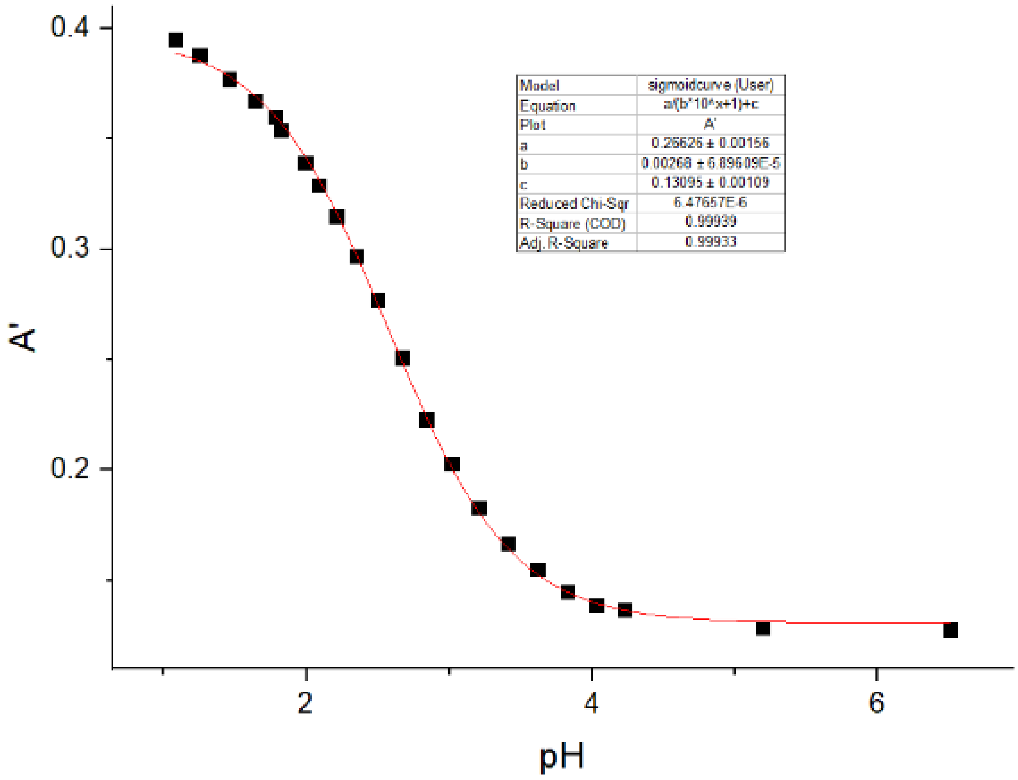

Figure 11. Absorption-pH plots (black dots) and the fitting curve (red line) of **6** titration (third measurement), meaning of parameters *a*, *b*, and *c* see previous sections.

$pK_a = –\log(b) = 2.57$.

Average pK_a = 2.53.

Titration study of selenoxide 1

Selected wavelength: 348 nm.

First measurement:

Titrant volume (µL)	Titrant amount (mmol)	pH[a]	*A* (348 nm)	*A'* (348 nm)[b]
**0**	0	**6.51**	0.699	**0.699**
**20**	0.040	**5.20**	0.692	**0.699**
**40**	0.080	**4.23**	0.683	**0.697**
**44**	0.088	**4.03**	0.681	**0.696**
**48**	0.096	**3.83**	0.678	**0.694**
**52**	0.104	**3.62**	0.674	**0.692**
**56**	0.112	**3.41**	0.669	**0.688**
**60**	0.120	**3.21**	0.664	**0.684**
**64**	0.128	**3.02**	0.659	**0.680**
**68**	0.136	**2.84**	0.654	**0.676**
**72**	0.144	**2.67**	0.650	**0.673**
**76**	0.152	**2.50**	0.647	**0.672**
**80**	0.160	**2.35**	0.644	**0.670**
**84**	0.168	**2.21**	0.642	**0.669**
**88**	0.176	**2.09**	0.640	**0.668**
**92**	0.184	**1.99**	0.639	**0.668**
**100**	0.200	**1.82**	0.636	**0.668**
**104**	0.208	**1.78**	0.635	**0.668**
**112**	0.224	**1.64**	0.632	**0.667**
**128**	0.256	**1.46**	0.630	**0.668**
**160**	0.320	**1.25**	0.626	**0.666**
**200**	0.400	**1.08**	0.616	**0.665**

Table 12. Titration results of the **1** solution (c = 0.20 mM) in Na-citrate buffer (pH 6.5, 50 mM, 2.0 mL, containing H_3PO_4 0.40 mM). Titrant: H_2SO_4 (2.0 M). a) From the average pH of Table 2. b) For the calculation of A' see previous sections.

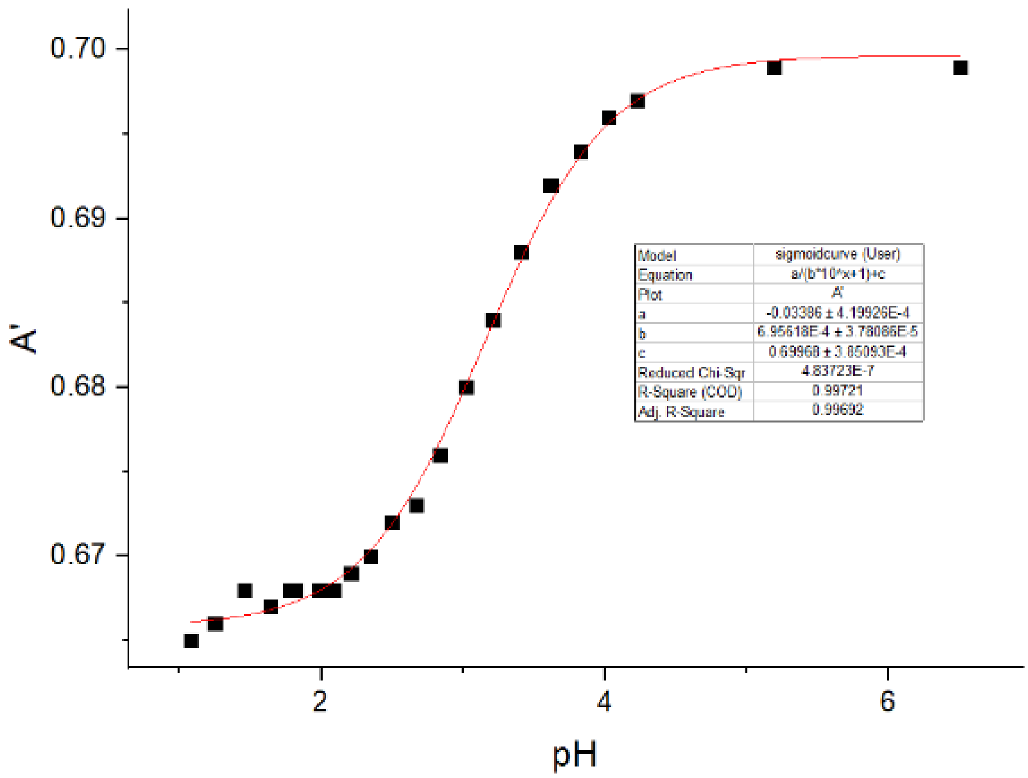

Figure 12. Absorption-pH plots (black dots) and the fitting curve (red line) of **1** titration (first measurement), meaning of parameters *a*, *b*, and *c* see previous sections.

$pK_a = -\log(b) = 3.16$.

Second measurement:

Titrant volume (µL)	Titrant amount (mmol)	pH	*A* (348 nm)	*A'* (348 nm)
**0**	0	**6.51**	0.707	**0.707**
**20**	0.040	**5.20**	0.701	**0.708**
**40**	0.080	**4.23**	0.691	**0.705**
**44**	0.088	**4.03**	0.689	**0.704**
**48**	0.096	**3.83**	0.686	**0.702**
**52**	0.104	**3.62**	0.682	**0.700**
**56**	0.112	**3.41**	0.677	**0.696**
**60**	0.120	**3.21**	0.672	**0.692**
**64**	0.128	**3.02**	0.667	**0.688**
**68**	0.136	**2.84**	0.662	**0.685**
**72**	0.144	**2.67**	0.658	**0.682**
**76**	0.152	**2.50**	0.654	**0.679**
**80**	0.160	**2.35**	0.652	**0.678**
**84**	0.168	**2.21**	0.650	**0.677**
**88**	0.176	**2.09**	0.648	**0.677**
**92**	0.184	**1.99**	0.646	**0.676**
**100**	0.200	**1.82**	0.643	**0.675**
**104**	0.208	**1.78**	0.642	**0.675**
**112**	0.224	**1.64**	0.636	**0.672**
**128**	0.256	**1.46**	0.633	**0.674**
**160**	0.320	**1.25**	0.624	**0.674**

**200**	0.400	**1.08**	0.611	**0.672**

Table 13. Second titration results of the **1** solution (c = 0.20 mM) in Na-citrate buffer (pH 6.5, 50 mM, 2.0 mL, containing H_3PO_4 0.40 mM). Titrant: H_2SO_4 (2.0 M). Conditions are same to Table 12.

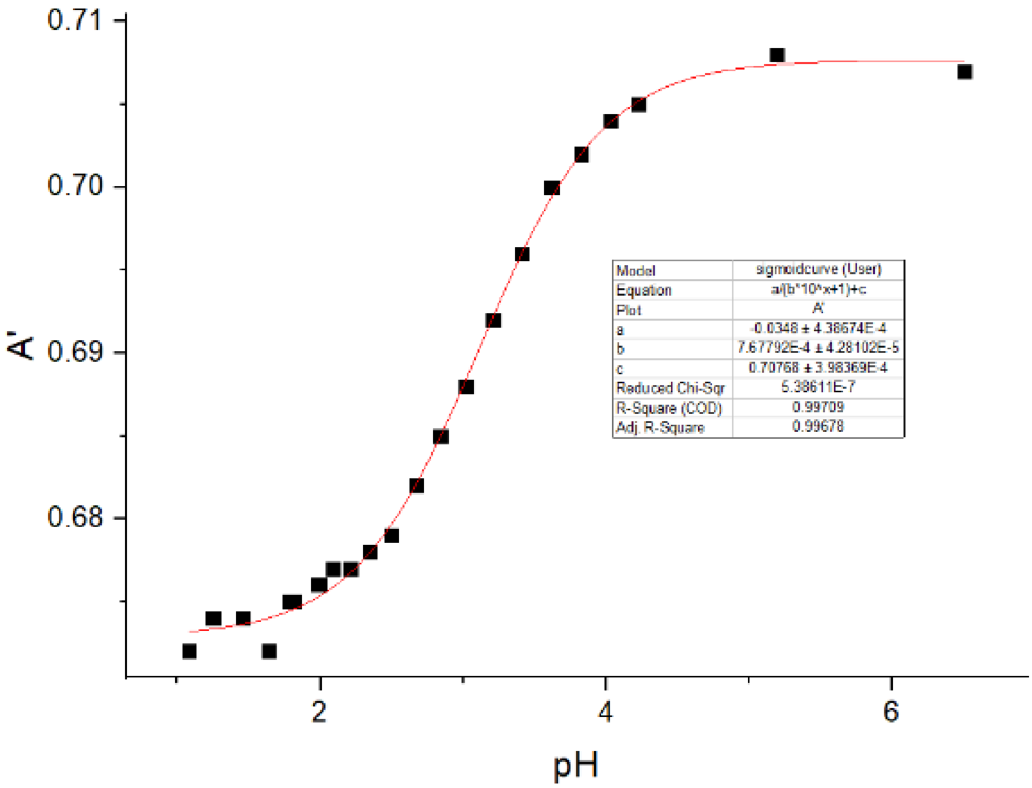

Figure 13. Absorption-pH plots (black dots) and the fitting curve (red line) of **1** titration (second measurement), meaning of parameters *a*, *b*, and *c* see previous sections.

$pK_a = -\log(b) = 3.11$.

Third measurement:

Titrant volume (μL)	Titrant amount	pH[a]	*A* (348 nm)	*A'* (348 nm)
**0**	0	**6.51**	0.700	**0.700**
**20**	0.040	**5.20**	0.692	**0.699**
**40**	0.080	**4.23**	0.684	**0.698**
**44**	0.088	**4.03**	0.682	**0.697**
**48**	0.096	**3.83**	0.678	**0.694**
**52**	0.104	**3.62**	0.674	**0.692**
**56**	0.112	**3.41**	0.669	**0.688**
**60**	0.120	**3.21**	0.664	**0.684**
**64**	0.128	**3.02**	0.658	**0.679**
**68**	0.136	**2.84**	0.655	**0.677**
**72**	0.144	**2.67**	0.650	**0.673**
**76**	0.152	**2.50**	0.647	**0.672**
**80**	0.160	**2.35**	0.643	**0.669**
**84**	0.168	**2.21**	0.642	**0.669**
**88**	0.176	**2.09**	0.640	**0.668**
**92**	0.184	**1.99**	0.639	**0.668**
**100**	0.200	**1.82**	0.637	**0.669**
**104**	0.208	**1.78**	0.636	**0.669**
**112**	0.224	**1.64**	0.633	**0.668**

**128**	0.256	**1.46**	0.628	**0.668**
**160**	0.320	**1.25**	0.616	**0.665**
**200**	0.400	**1.08**	0.604	**0.664**

Table 14. Third titration results of the **1** solution (c = 0.20 mM) in Na-citrate buffer (pH 6.5, 50 mM, 2.0 mL, containing H_3PO_4 0.40 mM). Titrant: H_2SO_4 (2.0 M). Conditions are same to Table 12.

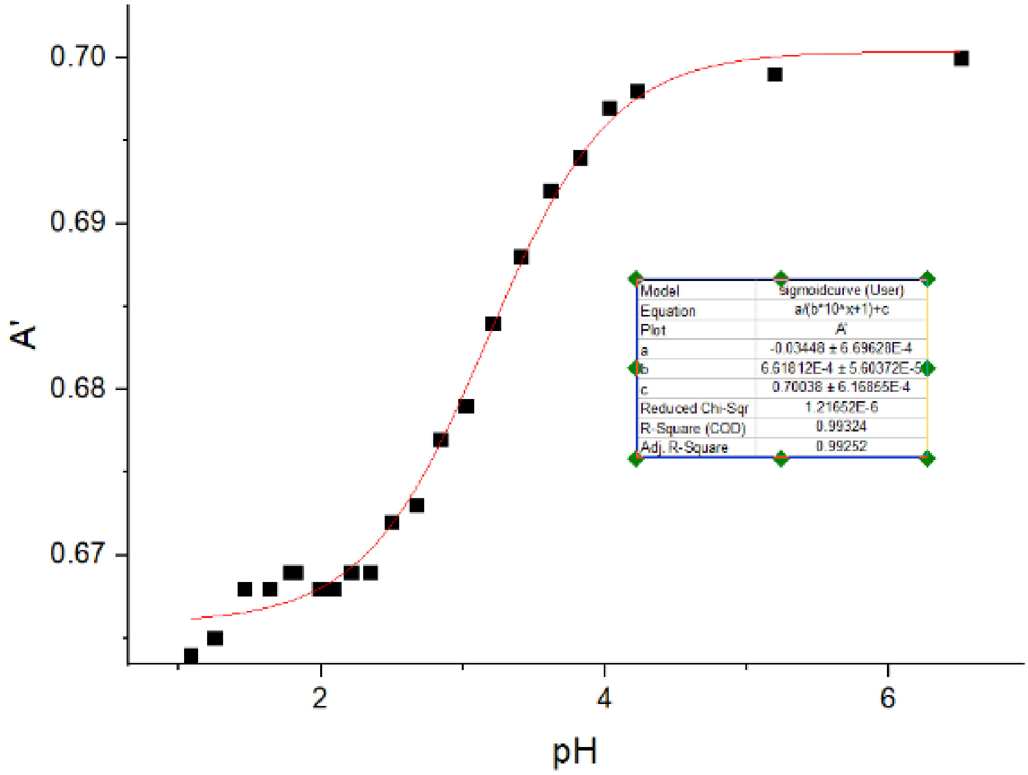

Figure 14. Absorption-pH plots (black dots) and the fitting curve (red line) of **1** titration (third measurement), meaning of parameters *a*, *b*, and *c* see previous sections.

$pK_a = -\log(b) = 3.18$

Average pK_a = 3.15.

NMR study of selenoxides in aqueous solutions

NMR study of DBSeO (4) and DBSeOH$^+$ (4H$^+$)

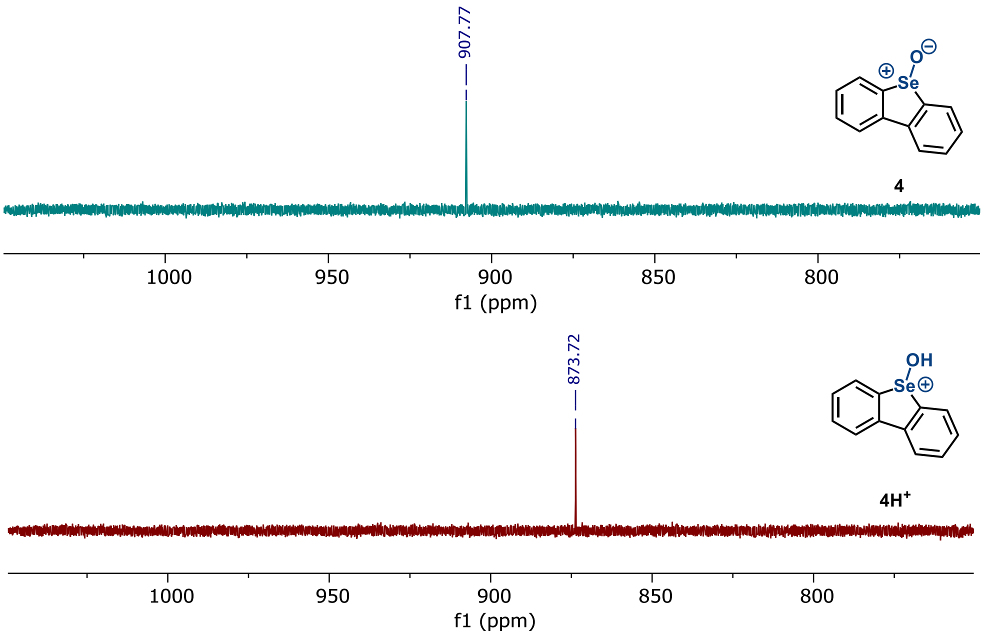

Figure 15. ^{77}Se-INEPT-NMR spectra of **4** in the solution of 0.1 M NaOH in MeCN-*d*$_3$/water (1/1) (green) and that of **4H**$^+$ in the solution of 50 mM H_2SO_4 in MeCN-*d*$_3$/water (1/1) (red).

Given that the pK_a of **4H**$^+$ is 1.6, the major species in the solution of 0.10 M NaOH in MeCN-*d*$_3$/water (1/1) (pH = 13) should be the selenoxide **4**. Based on previous study[10,11], selenoxide could exist as either acyclic selenurane or selenoxonium salt in acidic aqueous solution (Fig. 16), and the formation of selenoxonium from selenoxide leads to a small decreasing shift of ^{77}Se-NMR resonance (–27 ppm)[10] while the formation of acyclic selenurane results in a large decreasing shift (< –100 ppm)[11]. Given that the decreasing shift of ^{77}Se-NMR resonance of **4** during the pH change from 13 to 1 (–34 ppm) is more matched with the reported shift value of selenoxonium formation, we proposed that the major species in the solution of 50 mM H_2SO_4 in MeCN-*d*$_3$/water (1/1) (pH = 1) should be the diarylselenoxonium salt **4H**$^+$.

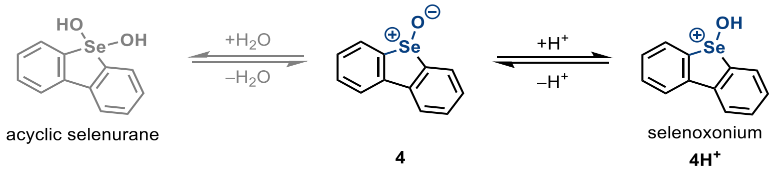

Figure 16. Two possible structures of selenoxide **4** under the acidic conditions.

NMR study of 2-(DBSeO)-Py 5 and 2-(DBSeOH)-Py⁺ 5H⁺

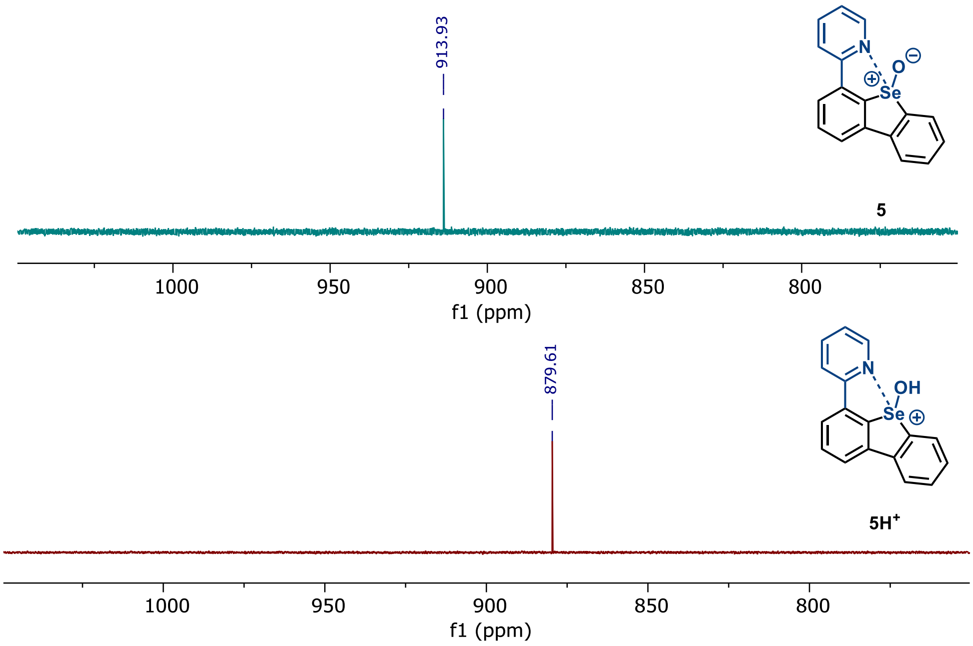

Figure 17. ^{77}Se-INEPT-NMR spectra of **5** in the solution of 0.1 M NaOH in MeCN-d_3/water (1/1) (green) and that of **5H⁺** in the solution of 50 mM H_2SO_4 in MeCN-d_3/water (1/1) (red). Both **5** and **5H⁺** could exist as racemates in the corresponding solution.

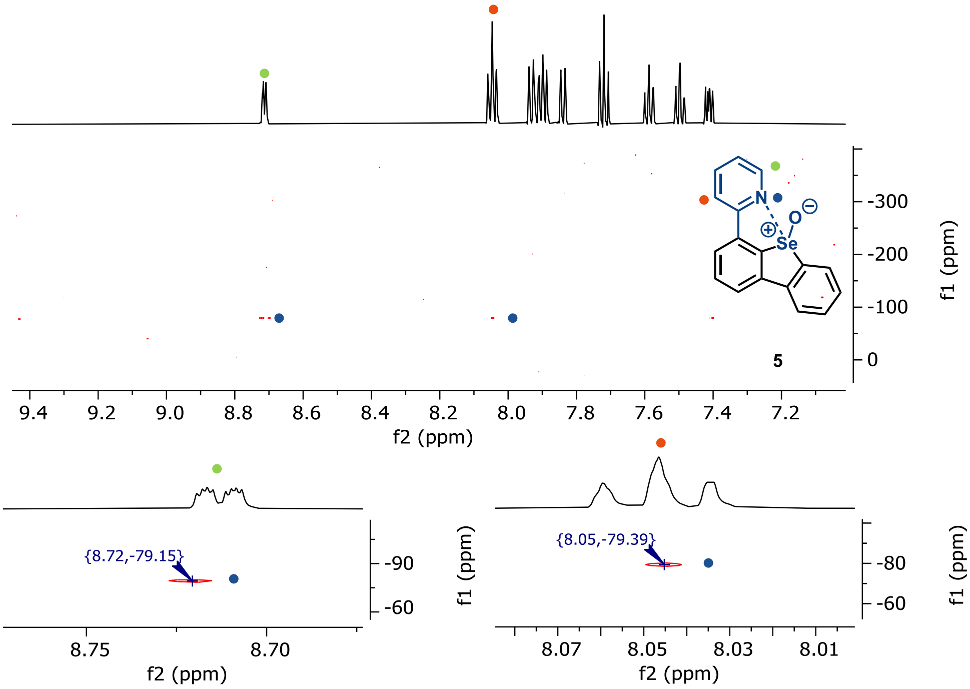

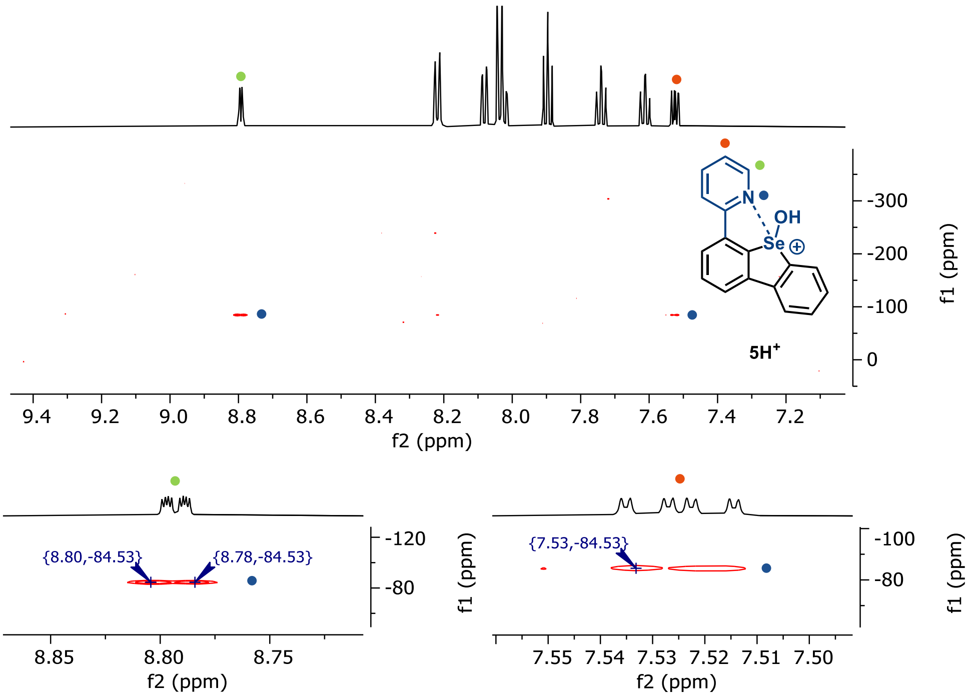

Figure 18. ^{1}H–^{15}N HMBC NMR spectra of **5** in the solution of 0.1 M NaOH in MeCN-*d*$_3$/water (1/1) and that of **5H$^+$** in the solution of 50 mM H_2SO_4 in MeCN-*d*$_3$/water (1/1). f1 (ppm), ^{15}N chemical shift; f2 (ppm), ^{1}H chemical shift; blue spots, NMR traces of pyridyl N nucleus; pale green and orange spots, NMR traces of pyridyl H nucleus coupled to pyridyl N nucleus in HMBC. Both **5** and **5H$^+$** could exist as racemates in the corresponding solution.

Given that the *p*K$_a$ of **5H$^+$** is 2.6, the major species in the solution of 0.10 M NaOH in MeCN-*d*$_3$/water (1/1) (pH = 13) should be the selenoxide **5**. The decreasing shift of ^{77}Se-NMR resonance of **5** during the pH change from 13 to 1 (–34.3 ppm) is nearly identical to the shift of **4H$^+$** compared to **4** (–34.0 ppm), which indicates that the major species in the solution of 50 mM H_2SO_4 in MeCN-*d*$_3$/water (1/1) (pH = 1) should be the diarylselenoxonium salt **5H$^+$**. Based on the reported increasing shift of ^{77}Se-NMR resonances during chalcogen bond formation (O···Se–C, 7.9 ppm[12]), the increasing shifts of ^{77}Se-NMR resonances of **5** and **5H$^+$** compared to **4** and **4H$^+$** (6.2 ppm and 5.9 ppm, respectively) provide an evidence that the intramolecular chalcogen bonds (N···Se–C) exist in both **5** and **5H$^+$** in aqueous solutions. In addition, the decreasing shift of ^{15}N-NMR resonance of **5H$^+$** compared to **5** (5.1 ppm), although much smaller than the shift of pyridinium compared to pyridine (< –100 ppm, ^{15}N-NMR resonance)[13], indicates that a small fraction of **5H$^+$** could exist as pyridinium salt. This is further supported by the computation results that ca. 3.8% of **5H$^+$** exists as **5H$^+_{py}$** based on the small Gibbs free energy difference between **5H$^+_{Se}$** and **5H$^+_{py}$** (1.92 kcal/mol, from **5H$^+_{Se}$** to **5H$^+_{py}$**).

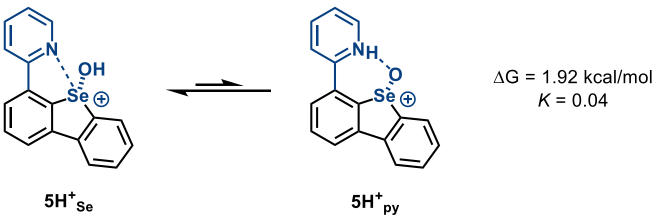

Figure 19. Equilibrium between **5H$^+_{Se}$** and **5H$^+_{py}$** in aqueous solution. Gibbs free energy ($\Delta G_{solv\ 298K}$) calculated at the D3(BJ)/def2-TZVPP level in water.

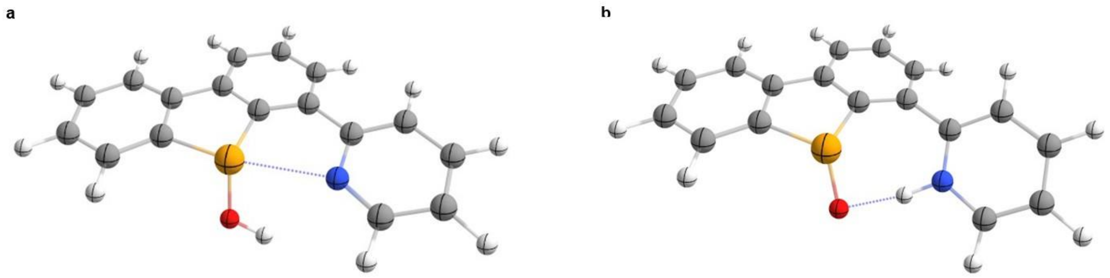

Figure 20. Geometry structure of a) **5H^{+}$_{Se}$** and b) **5H^{+}$_{py}$.** Dashed lines represent chalcogen bond or hydrogen bond.

NMR study of DBSeOPy-amide 6 and DBSeOHPy-amide$^+$ 6H$^+$

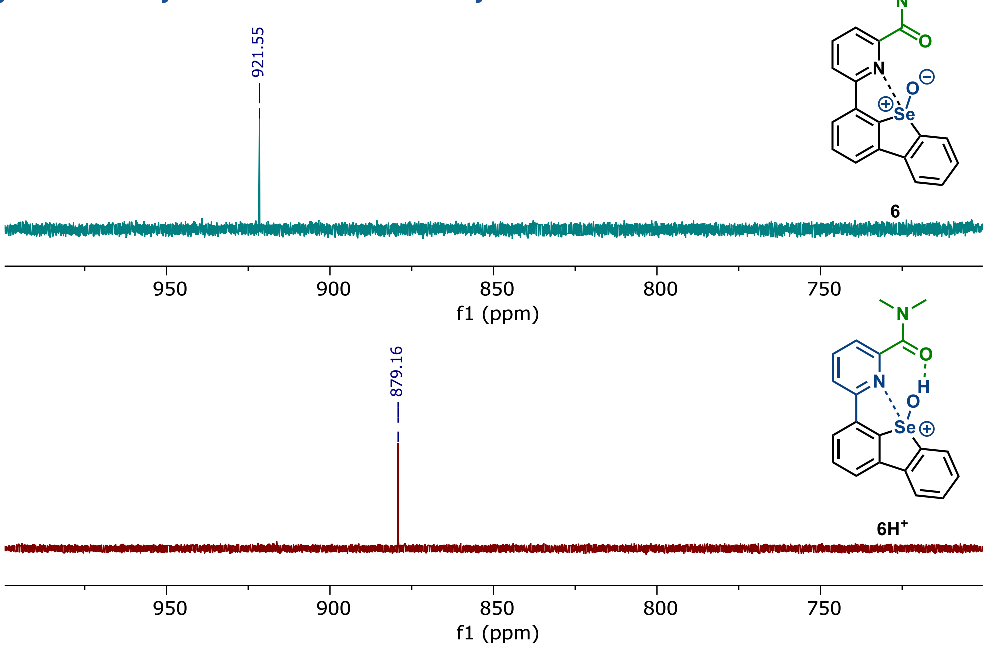

Figure 21. ^{77}Se-INEPT NMR spectra of **6** in the solution of 0.1 M NaOH in MeCN-d_3/water (1/1) (green) and that of **6H**$^+$ in the solution of 50 mM H_2SO_4 in MeCN-d_3/water (1/1) (red). Both **6** and **6H**$^+$ could exist as racemates in the corresponding solution.

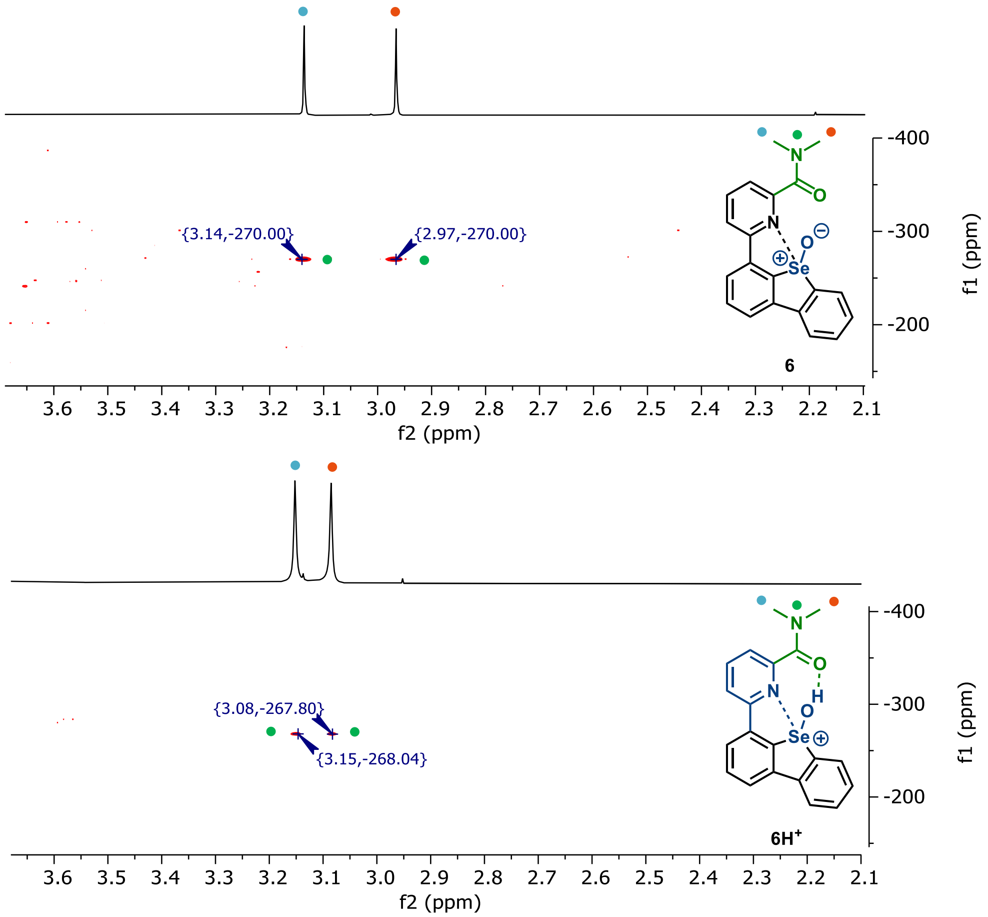

Figure 22. ^{1}H–^{15}N HMBC NMR spectra of **6** in the solution of 0.1 M NaOH in MeCN-d_3/water (1/1) and that of **6H**$^+$ in the solution of 50 mM H_2SO_4 in MeCN-d_3/water (1/1). f1 (ppm), ^{15}N chemical shift; f2 (ppm), ^{1}H chemical shift; green spots, NMR traces of amide N nucleus; cyan and orange spots, NMR traces of methyl H nucleus coupled to amide N nucleus in HMBC. Both **6** and **6H**$^+$ could exist as racemates in the corresponding solution.

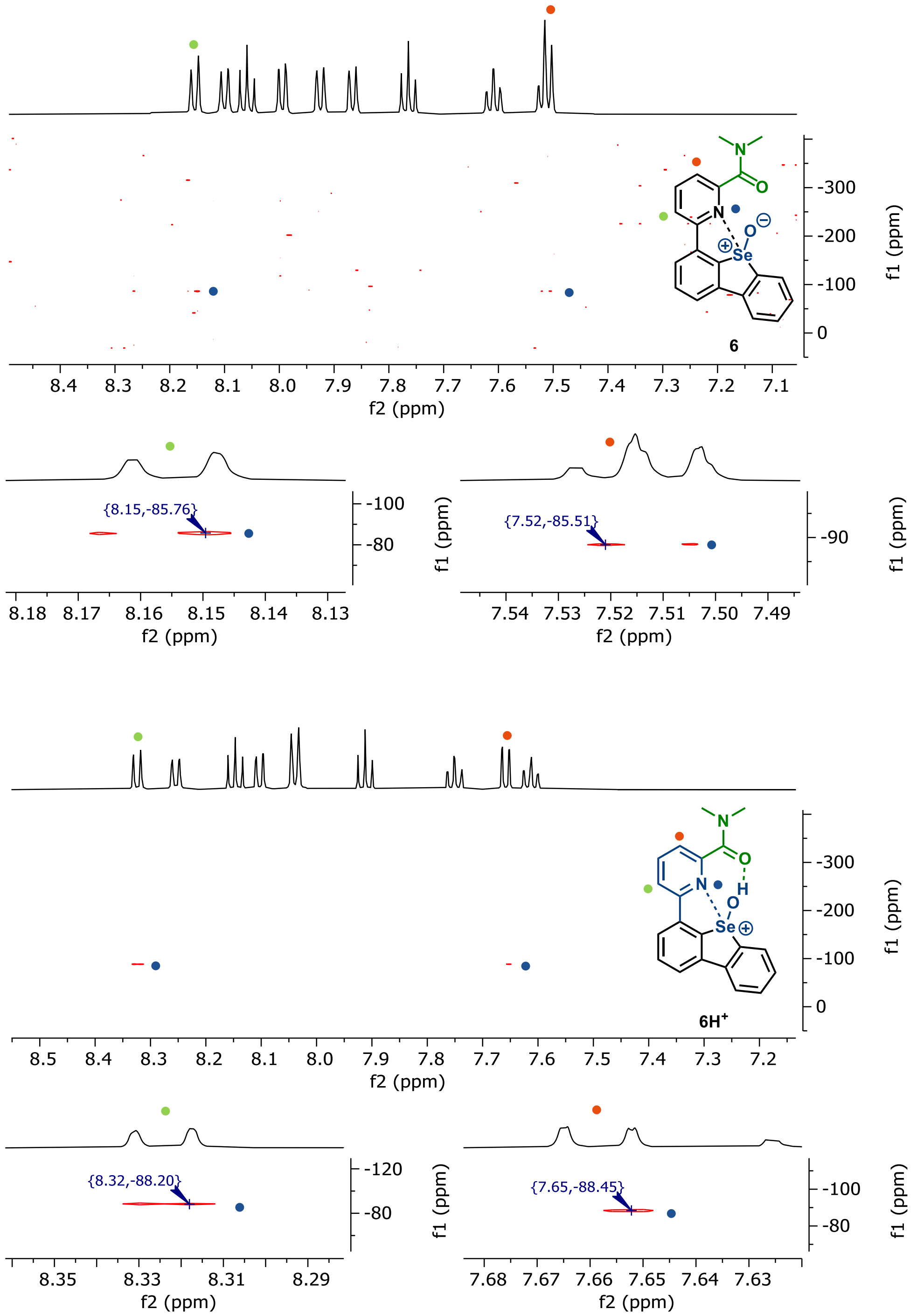

Figure 23. ^{1}H–^{15}N HMBC NMR spectra of **6** in the solution of 0.1 M NaOH in MeCN-d_3/water (1/1) and that of **6H**$^{+}$ in the solution of 50 mM H_2SO_4 in MeCN-d_3/water (1/1). f1 (ppm), ^{15}N chemical shift; f2 (ppm), ^{1}H chemical shift; blue spots, NMR traces of pyridyl N nucleus; pale green and orange spots, NMR traces of pyridyl H nucleus coupled to pyridyl N nucleus in HMBC. Both **6** and **6H**$^{+}$ could exist as racemates in the corresponding solution.

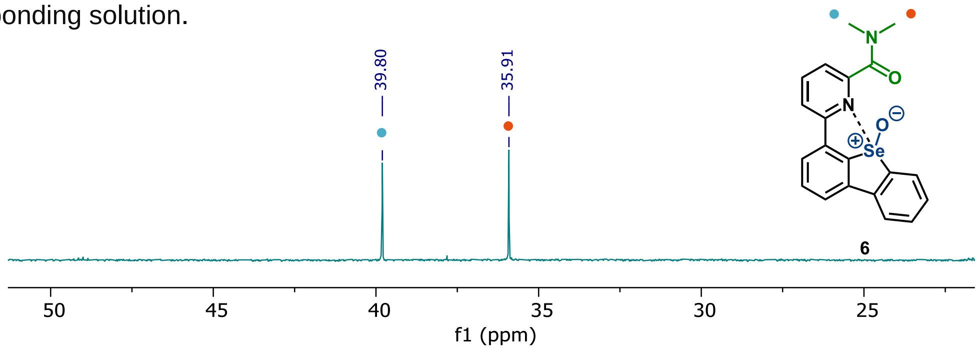

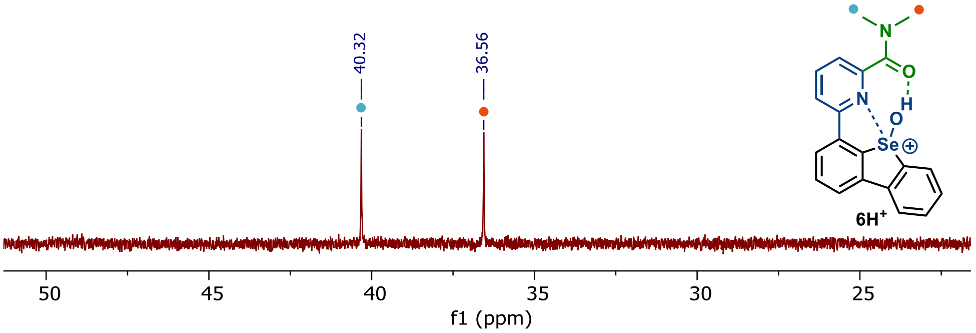

Figure 24. ^{13}C-chemical shift of the methyl carbons of **6** in the solution of 0.1 M NaOH in MeCN-d_3/water (1/1) (green) and that of **6H**$^+$ in the solution of 50 mM H_2SO_4 in MeCN-d_3/water (1/1) (red). Cyan and orange spots, NMR traces of methyl C nucleus. Both **6** and **6H**$^+$ could exist as racemates in the corresponding solution.

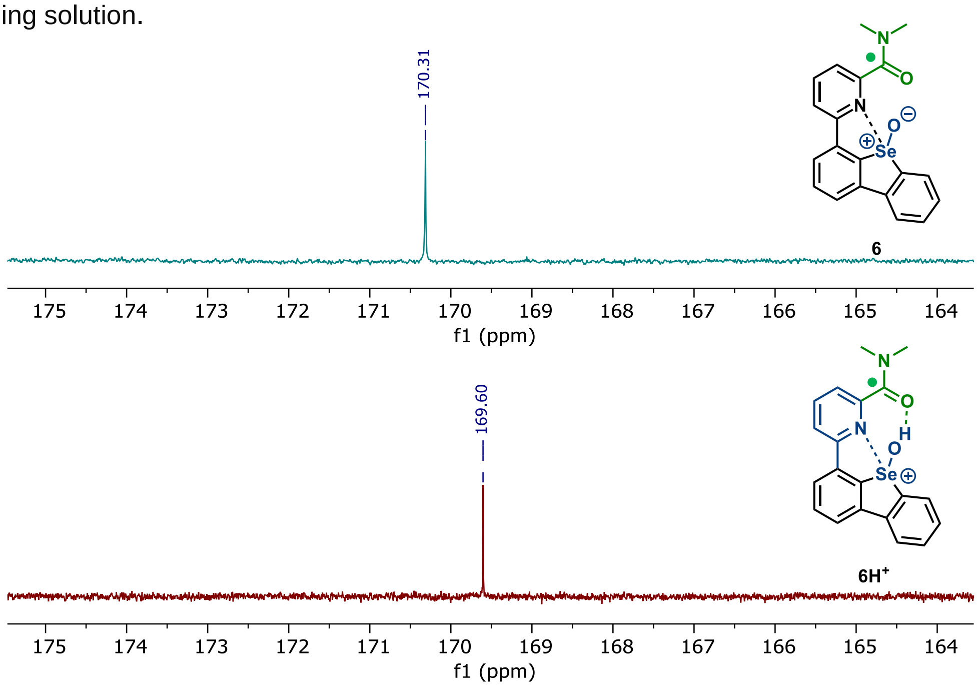

Figure 25. ^{13}C-chemical shift of the carbonyl carbons of **6** in the solution of 0.1 M NaOH in MeCN-d_3/water (1/1) (green) and that of **6H**$^+$ in the solution of 50 mM H_2SO_4 in MeCN-d_3/water (1/1) (red). Green spots, carbonyl C nucleus. Both **6** and **6H**$^+$ could exist as racemates in the corresponding solution.

Given that the pK_a of **6H**$^+$ is 2.5, the major species in the solution of 0.10 M NaOH in MeCN-d_3/water (1/1) (pH = 13) should be the selenoxide **6**. Similarly, the decreasing shift of ^{77}Se-NMR resonance (–42.4 ppm) of **6** during the pH change from 13 to 1 indicates that the major species in the solution of 50 mM H_2SO_4 in MeCN-d_3/water (1/1) (pH = 1) should be the selenoxonium salt **6H**$^+$. Although the increasing shift of ^{77}Se-NMR resonances of **6** compared to **4** (13.8 ppm) was larger than that of **5** compared to **4** (6.2 ppm), we cannot deduce that the chalcogen bond in **6** is stronger because the shift values do not correlate with the strength of chalcogen bonds[14]. It should be noticed that the ^{15}N-NMR resonance of amide group is unsensitive to pH change in aqueous solution (shifts < 1 ppm in the pH range of 0.5–13[15]). Therefore, the increasing shift of ^{15}N-NMR resonance indicates that the amide **6H**$^+$ is hydrogen-bonded to the intramolecular selenoxonium group rather than the hydrated protons in the acidic solution. Interestingly, a decreasing shift of ^{13}C-NMR resonance (C of carbonyl group) of **6H**$^+$ compared to **6** was observed (–0.7 ppm), which is different from the typical increasing shift values of amides hydrogen bonding to more acidic hydrogen-bonding-donors via carbonyl groups (0.9 ppm, solvent changed from water to trifluoroethanol[16]). Indeed, the decreasing shift of the ^{13}C-NMR resonance (C of carbonyl group) here is a summed result of both the deshielding effect (from hydrogen bond) and the shielding effect (from

conjugation). Based on the computation study, the conjugation between the pyridyl group and the amide group of **6H**$^+$ is found to be better compared to that of **6** (Fig. 26). The better conjugation, which increases the π electron density of the carbonyl group in **6H**$^+$ and hence the shielding on the carbonyl carbon nuclei[17], could explain the decreasing shift of ^{13}C-NMR resonance of carbonyl carbon of **6H**$^+$. Noticeably, the smaller decreasing shift of ^{15}N-NMR resonance (N of pyridyl group, –2.7 ppm) of **6H**$^+$ relative to **6** compared to the shift of **5H**$^+$ relative to **5** indicates that the pyridinium form of **6H**$^+$ is more unfavorable in the equilibrium with its selenoxonium form, which could result from both the lower basicity of amide-susbstituted pyridyl group and the synergistic stabilization of the diarylselenoxonium form by both the intramolecular chalcogen bond and hydrogen bond of **6H**$^+$.

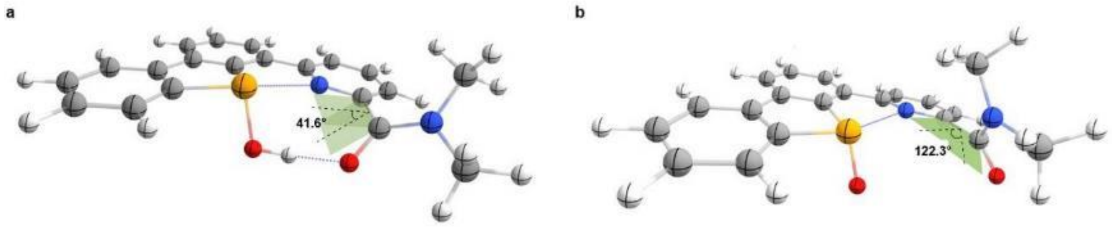

Figure 26. Geometry structure and dihedral torsions between amide and pyridyl planes (grass green planes) of a) **6H**$^+$ and b) **6**. Geometry optimization was conducted at the D3(BJ)/def2-TZVPP level in water. Blue dashed lines, chalcogen bonds or hydrogen bonds; black dashed lines, auxiliary lines for dihedral torsion measurements.

NMR study of DBSeOPy-oxa 1 and DBSeOHPy-oxa$^+$ 1H$^+$

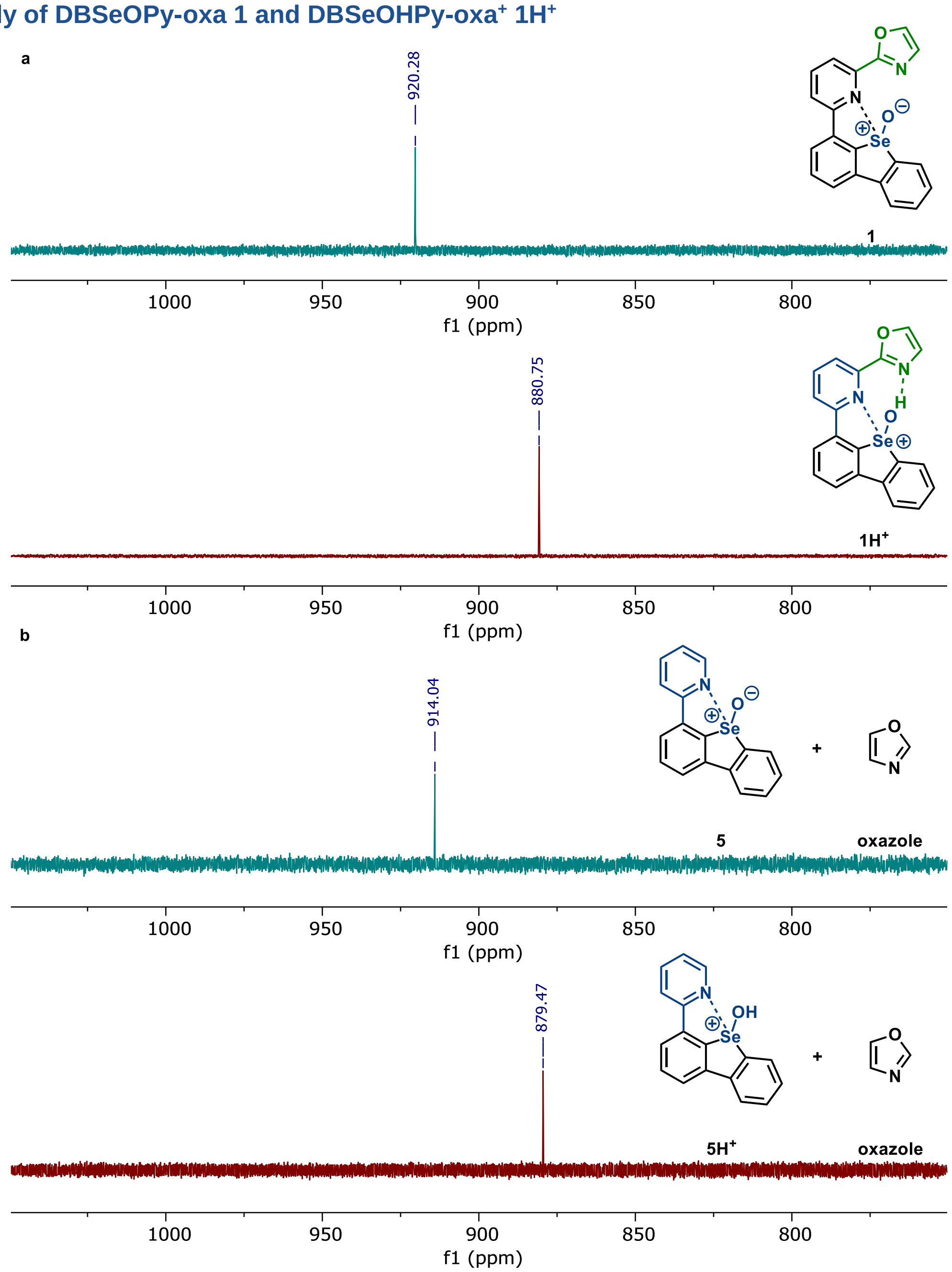

Figure 27. ^{77}Se-INEPT NMR spectra of compounds in the solution of 0.1 M NaOH in MeCN-*d*$_3$/water (1/1) (green) and in the solution of 50 mM H_2SO_4 in MeCN-*d*$_3$/water (1/1) (red). a) **1** and **1H**$^+$ b) Equimolar mixture of **5** + **oxazole** and **5H**$^+$ + **oxazole**. **1**, **5**, **1H**$^+$, and **5H**$^+$ could exist as racemates in the corresponding solution.

a

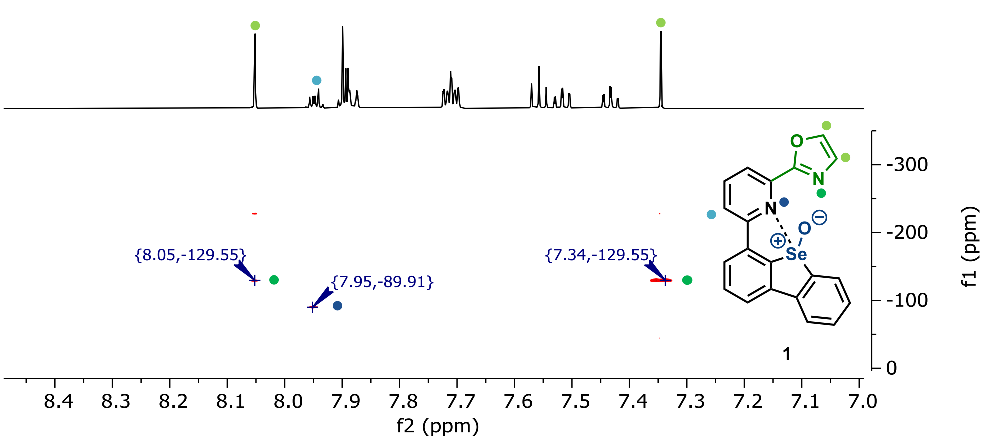
{8.05,-129.55}
{7.95,-89.91}
{7.34,-129.55}
1
f2 (ppm)
f1 (ppm)
{8.13,-140.07}
{8.23,-91.87}
{7.44,-139.83}
1H+

b

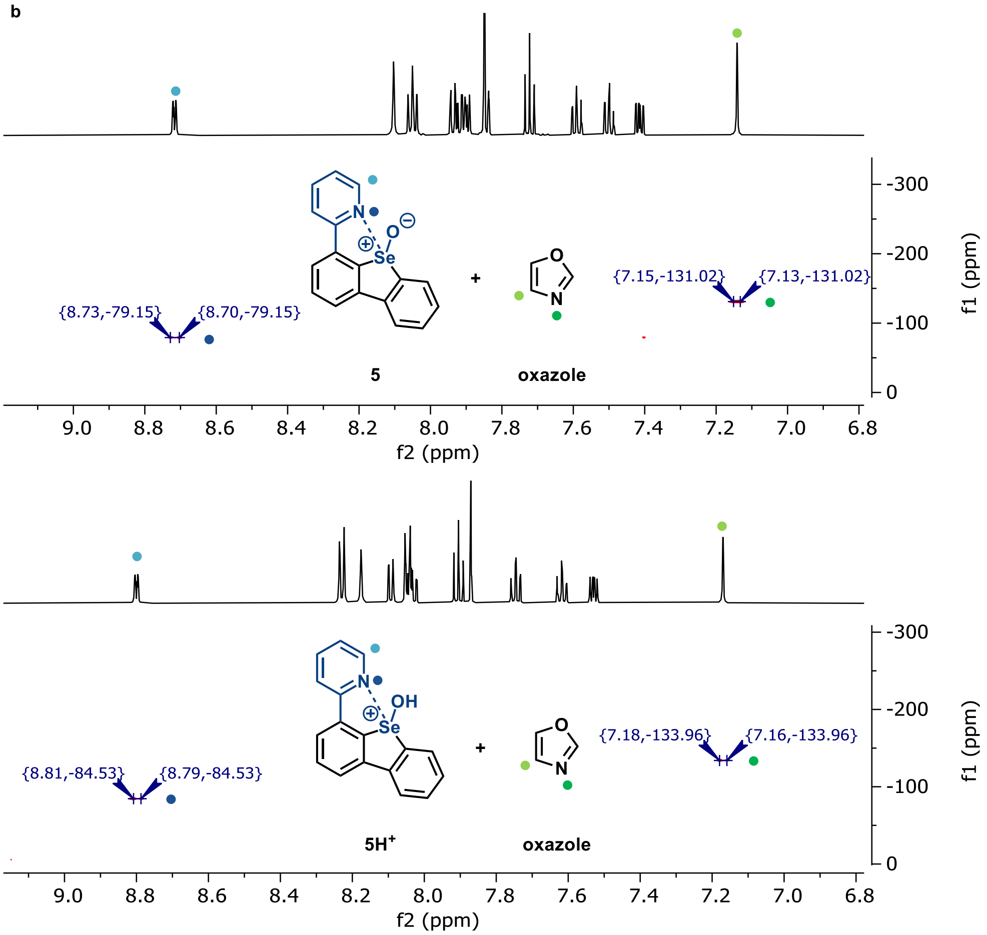
{8.73,-79.15}
{8.70,-79.15}
{7.15,-131.02}
{7.13,-131.02}
5
oxazole
f2 (ppm)
f1 (ppm)
{8.81,-84.53}
{8.79,-84.53}
{7.18,-133.96}
{7.16,-133.96}
5H+
oxazole

Figure 28. ^{1}H–^{15}N HMBC NMR spectra of compounds in the solution of 0.1 M NaOH in MeCN-d_3/water (1/1) and in the solution of 50 mM H_2SO_4 in MeCN-d_3/water (1/1). f1 (ppm), ^{15}N chemical shift; f2 (ppm), ^{1}H chemical shift; blue spots, NMR traces of pyridyl N nucleus; cyan spots, NMR traces of pyridyl H nucleus coupled to pyridyl N nucleus in HMBC; green spots, NMR traces of oxazolyl (or oxazole) N nucleus; pale green spots, NMR traces of oxazolyl (or oxazole) H nucleus coupled to oxazolyl (or oxazole) N nucleus in HMBC.a) **1H**$^+$ and **1** b) Equimolar mixture of **5H**$^+$ + **oxazole** and **5** + **oxazole**. **1**, **5**, **1H**$^+$, and **5H**$^+$ could exist as racemates in the corresponding solution.

Given that the pK_a of **1H**$^+$ is 3.2, the major species in the solution of 0.10 M NaOH in MeCN-d_3/water (1/1) (pH = 13) should be the selenoxide **1**. Similarly, the major species in the solution of 50 mM H_2SO_4 in MeCN-d_3/water (1/1) (pH = 1) should be the diarylselenoxonium salt **1H**$^+$ based on the decreasing shift of ^{77}Se-NMR resonance of **1H**$^+$ compared to **1** (–39.5 ppm) that is similar to the shift value of **6H**$^+$ compared to **6** (–42.4 ppm). The ^{77}Se-NMR and ^{15}N-NMR resonances of **5** and **5H**$^+$ in the control experiments are nearly identical to the resonances obtained in the NMR study of **5** and **5H**$^+$ ($\Delta\delta_{Se}$ = 0.11 and –0.14 for **5** and **5H**$^+$, respectively; $\Delta\delta_{N(pyridyl)}$ = 0 for both **5** and **5H**$^+$), which indicates that oxazole has almost no interactions with **5** or **5H**$^+$ under the measurement conditions. According to the analysis, the small decreasing shift of ^{15}N-NMR resonance of oxazole (–2.9 ppm) in the control experiments during the solution pH change from 13 to 1 should result from the hydrogen bonding between oxazoles and hydrated protons, or from a small amount of protonated oxazoles at pH 1. Therefore, the larger decreasing shift of ^{15}N-NMR resonance (N of oxazolyl group, –10.4 ppm) of **1H**$^+$ compared to **1** cannot simply be explained by the interactions between oxazolyl groups and hydrated protons caused by the solution pH change or, by a second protonation of **1H**$^+$ at oxazolyl nitrogen under the measurement conditions (50 mM H_2SO_4 in MeCN-d_3/water (1/1), pH 1) which is not supported by the titration experiment (Figs. 12–14). Indeed, the phenomenon indicates that the oxazolyl nitrogen of **1H**$^+$ may intramolecularly hydrogen bond to the selenoxonium group. Because of the higher acidity of the selenoxonium group compared to bulk water, the hydrogen bond of **1H**$^+$ ($N_{oxazolyl}\cdots$HO–Se) induces the change of the contribution of the sp^2-hybridized nitrogen lone pair to the overall shielding tensor, thus leading to the observed decreasing shift of ^{15}N-NMR resonance of $N_{oxazolyl}$ of **1H**$^+$ compared to **1**[18]. The existence of the water-resistant intramolecular hydrogen bond in **1H**$^+$ could be further supported by the increased pK_a of **1H**$^+$ compared to that of **5H**$^+$ (ΔpK_a = 0.6, Figs. 12–14), which indicates an extra stabilization of **1H**$^+$ in addition to the intramolecular chalcogen bond ($N_{pyridyl}\cdots$Se–C).

Study on transformations of NAc-Tyr-NH2 derived DBSePy-oxa-selenonium salt 15

Reaction design

The tyrosine-based selenonium salt **15** was used as a model compound to study the transformations of phenolic selenonium group. Because of the large contribution of C_{Tyr}–Se antibonding orbital to the LUMO of **15** (Fig. 74) and the absorption of **15** in the range of visible light (365–465 nm, Fig. 70), we hypothesized that **15** could be excited and lysed to Tyr radical and selenyl radical cation under visible light irradiation without photocatalyst. To achieve the homolytic cleavage of the C_{Tyr}–Se bond, the energy of the photon absorbed by **15** should be higher than the bond dissociation energy of the C_{Tyr}–Se bond (70.6 kcal/mol, Fig. 73). Therefore, we chose the 390 nm LED as the light source (photon energy: 73.2 kcal/mol) for the photoreaction of **15**. After the photolysis of **15**, the Tyr radical could participate in various radical reactions to give the desired functionalized products and importantly, a sacrificial reductant is necessary to quench the oxidizing selenyl radical cation and hence protect the products from unwanted oxidative decomposition by the selenyl radical cation. Apart from the photoreaction, the selenonium salt **15** was akin to aryl thianthrenium salt and could be a suitable substrate in transition-metal catalyzed cross-coupling reactions such as Suzuki coupling.

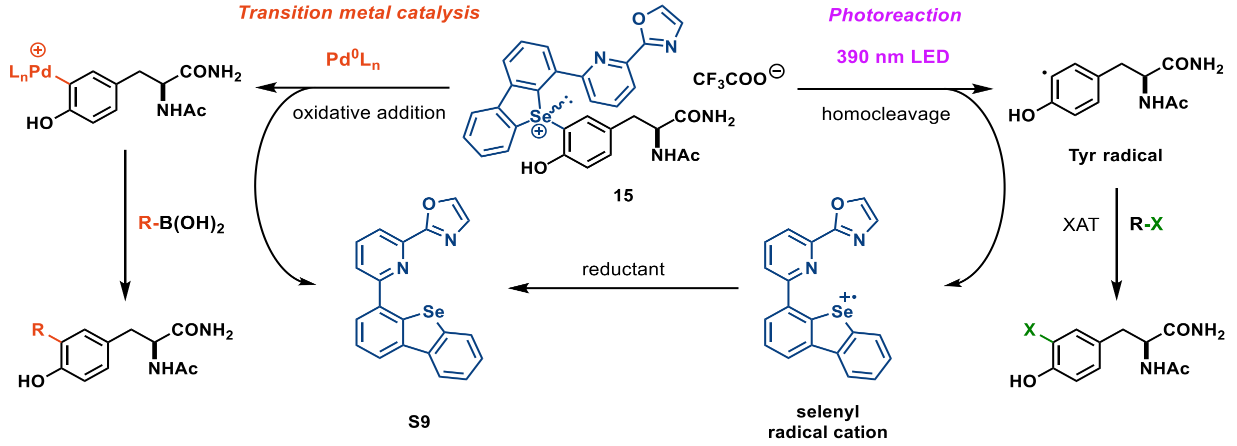

Figure 29. Proposed reaction mechanism for photoreaction and transition-metal catalysis of **15**. L, ligand; Ac, acetyl; X, halogen; R, aryl or alkyl group; XAT, halogen atom transfer.

General considerations on reaction scale and concentration

The goal of studying transformations on the model compound **15** is to develop the transformations for peptide- or protein-selenonium conjugates, which typically proceed with low micromolar peptide/protein concentrations (10–50 µM) and small reaction volumes (50–200 µL). Considering the sensitivity of photoreactions to reaction scales and reactant/reagent concentrations, we decided to study the transformations of the model compound **15** with relatively low selenonium salt concentration (2.0 mM) as well as small reaction volume (200 µL), which are similar to the conditions of peptide or protein transformations. However, the small scale of the reaction (0.32 µmol, 0.23 mg of **15**) is an obstacle to the full characterization of the product and the determination of the purification yield. Therefore, we used ^{1}H-NMR to determine all small-scale-reaction yields with internal standards. For the purification and subsequent full characterization of the product, a scale-up reaction (40 µmol, 28 mg of **15**) was conducted based on the best conditions (with some adaptive modifications due to the higher concentration of **15** in the scale-up reactions) of the corresponding small-scale transformation of **15**. To

our delight, scaling-up resulted in only a small drop of the yield (5–12%) for all transformations except the coumarin formation of **15**.

Iodination of the selenonium salt 15

Optimization on a small scale

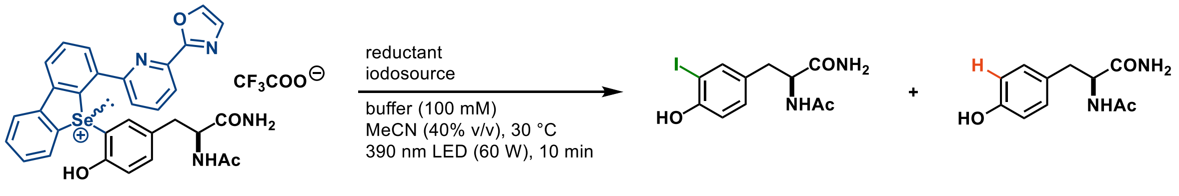

15, 1.6 mM **16** **2**

Under nitrogen atmosphere, a GC-vial (2 mL) equipped with a Teflon-coated mini magnetic stirring bar was charged with the reductant and the iodosource, followed by the addition of buffer (c = 0.50 M, 40 µL, final concentration c = 100 mM) or the addition of a sulfuric acid solution (c = 0.25 M, 40 µL, final concentration c = 50 mM) in UHQ-H_2O, a stock solution of selenonium salt **15** (c = 16 mM, 20 µL, 0.32 µmol, 0.23 mg, 1.0 equiv., final concentration c = 1.6 mM) in MeCN/UHQ-H_2O (1/1, v/v), the cosolvent MeCN (70 µL, final volume percentage 40%), and UHQ-H_2O (70 µL, to reach the final volume of 200 µL). The vial was closed by a screw cap, placed between 2 Kessil PR160-390 nm LEDs (2.5 cm away from each lamp) and irradiated for 10 min. The temperature of the reaction was kept at approximately 30 °C through the use of a cooling fan. Subsequently, the mixture was diluted by 800 µL of MeCN/UHQ-H_2O (1/1, v/v) and was saturated by anhydrous Na_2SO_4 (ca. 0.3 g). The mixture was extracted by THF/EtOAc (1/1, v/v, 500 µL × 3) and the organic layers were combined, concentrated under reduced pressure and dried *in vacuo*. The residue was dissolved in CD_3OD and 2.5 µL of a 1,3,5-trimethoxybenzene solution (c = 0.10 M, 0.25 µmol, 42 µg, 0.78 equiv.) in CD_3OD was added as internal standard. The mixture was analyzed via ^{1}H-NMR and a singlet peak at 6.08 ppm (1,3,5-trimethoxybenzene) was set as 3.00. Yield of different products was determined as following:

Yield of the product of **16** = integration of peak [δ: 6.77 (d, 1H)] / 1.28 × 100%.

Yield of the product of **2** = integration of peak [δ: 6.72 (d, 2H)] / 2.56 × 100%.

Entry	Reductant (mM[a])	Iodo-source (mM[a])	Buffer	Yield of 16 (2)[b]
1	–	NaI, 100 mM	NaPi (pH 6.0)	34% (45%)
2	–	NaI, 100 mM	Na-acetate (pH 4.5)	41% (42%)
3	–	NaI, 100 mM	H_2SO_4 (pH 1.0, 50 mM[a])	4% (0%)
**4**	**NaI, 200 mM**	***i*Pr-I, 60 mM**	**NaPi (pH 6.0)**	**84% (4%)**
5	Hantzsch ester, 50 mM	*i*Pr-I, 60 mM	NaPi (pH 6.0)	76% (10%)
**6**	**–**	***i*Pr-I, 60 mM**	**NaPi (pH 6.0)**	**0% (0%)**
7[c]	NaI, 200 mM	*i*Pr-I, 60 mM	NaPi (pH 6.0)	N.R.

Table 15. Optimization of the iodination of the selenonium salt **15**. a) Final concentration in the reaction mixture. b) ^{1}H-NMR yield using 1,3,5-trimethoxybenzene as internal standard. c) Reaction conducted in the dark.

Preparation of NAc-3-iodo-tyrosinamide 16

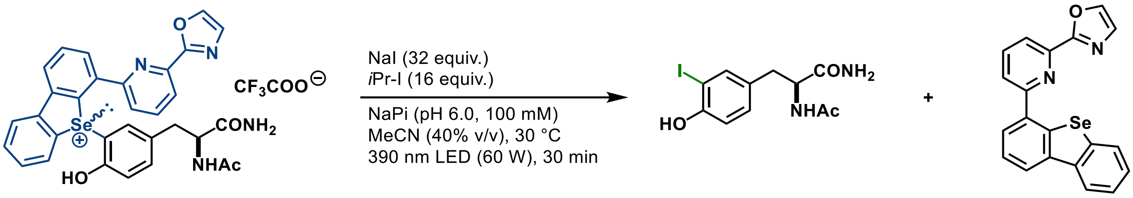

15, 6.3 mM **16** **72%** yield **S9** **90%** yield

Under ambient atmosphere, a scintillation vial (20 mL) equipped with Teflon-coated magnetic stirring bar was charged with the selenonium salt **15** (28 mg, 0.040 mmol, 1.0 equiv.) and the reductant NaI (190 mg, 1.3 mmol, 32 equiv.). Next, 3200 µL of NaPi buffer (pH 6.0, c = 200 mM, final concentration c = 100 mM), 640 µL of UHQ-H_2O, and 2560 µL of the cosolvent MeCN (final volume percentage 40%) were added and the mixture was stirred at 25 °C until all solids dissolved. Then the mixture was degassed by gently purging argon through the solution via a needle (Φ 0.80 × 120 mm) for 5 min. Thereafter, the iodosource *i*Pr-I (110 mg, 64 µL, 0.64 mmol, 16 equiv.) was added and the mixture was degassed for an additional 1 min. Then the vial was closed by a screw cap, placed between 2 Kessil PR160-390 nm LEDs (2.5 cm away from each lamp) and irradiated for 30 min. The temperature of the reaction mixture was kept at approximately 30 °C through the use of a cooling fan. The reaction was diluted by UHQ-H_2O (2.5 mL) and the iodine formed during the reaction (detected by a solution of starch (0.1 wt.%) in UHQ-H_2O) was quenched by the addition of $Na_2S_2O_3{\cdot}5H_2O$ (160 mg, 0.64 mmol, 16 equiv.). Subsequently, anhydrous Na_2SO_4 (ca. 3 g) was added to saturate the solution and the mixture was extracted by THF/EtOAc (1/1, v/v, 5.0 mL × 4). The organic layers were combined and concentrated to dryness under high vacuum. The residue was purified by the column chromatography on silica gel eluting firstly with DCM to afford the selenide **S9** as a colorless solid (15 mg, 90%), and subsequently with DCM/MeOH (30/1–20/1) to afford the desired compound as a yellow solid. Further purification by semi-preparative reverse phase HPLC (YMC-Triart C18, 150 × 20.0 mm, 5 µm) with an eluent mixture of water/MeOH (55/45) at a flow rate of 15.0 mL/min to afford the desired compound as a colorless solid (10 mg, 72%).

R_f = 0.23 (silica gel, DCM/MeOH = 9/1).

NMR Spectroscopy:

^{1}H NMR (500 MHz, CD_3OD, 298 K, δ): 7.59 (d, *J* = 2.2 Hz, 1H), 7.07 (dd, *J* = 8.3, 2.2 Hz, 1H), 6.74 (d, *J* = 8.2 Hz, 1H), 4.51 (dd, *J* = 9.1, 5.6 Hz, 1H), 3.01 (dd, *J* = 14.0, 5.6 Hz, 1H), 2.73 (dd, *J* = 13.9, 9.1 Hz, 1H), 1.92 (s, 3H)

^{13}C NMR (126 MHz, CD_3OD, 298 K, δ): 176.3, 173.1, 158.2, 140.8, 131.3, 130.5, 116.0, 85.5, 56.0, 37.6, 22.4.

HRMS GC-EI (m/z) calc'd for $C_{11}H_{13}N_2O_3I^+$ $[M]^+$, 347.9965; found, 347.9966. Deviation: –0.3 ppm.

Bromination of the selenonium salt 15

Optimization on a small scale

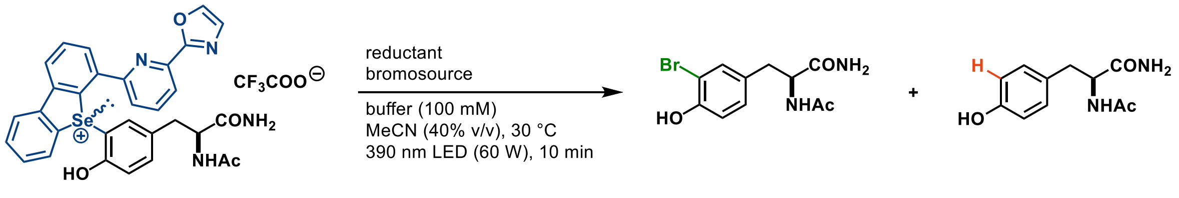

15, 1.6 mM **17** **2**

Under nitrogen atmosphere, a GC-vial (2 mL) equipped with a Teflon-coated mini magnetic stirring bar was charged with the reductant and the bromosource, followed by the addition of buffer (c = 200 mM, 100 µL, final concentration c = 100 mM), a stock solution of selenonium salt **15** (c = 16 mM, 20 µL, 0.32 µmol, 0.23 mg, 1.0 equiv., final concentration c = 1.6 mM) in MeCN/UHQ-H_2O (1/1, v/v), the cosolvent MeCN (70 µL, final volume percentage 40%), and UHQ-H_2O (10 µL, to reach the final volume of 200 µL). The vial was closed by a screw cap, placed between 2 Kessil PR160-390 nm LEDs (2.5 cm away from each lamp) and irradiated for 10 min. The temperature of the reaction mixture was kept at approximately 30 °C through the use of a cooling fan. Subsequently, the mixture was diluted by 800 µL of MeCN/UHQ-H_2O (1/1, v/v) and was saturated by anhydrous Na_2SO_4 (ca. 0.3 g). The mixture was extracted by THF/EtOAc (1/1, v/v, 500 µL × 3) and the organic layers were combined, concentrated under reduced pressure and dried *in vacuo*. The residue was dissolved in CD_3OD and 2.5 µL of a 1,3,5-trimethoxybenzene solution (c = 0.10 M, 0.25 µmol, 42 µg, 0.78 equiv.) in CD_3OD was added as internal standard. The mixture was analyzed via ^{1}H-NMR and a singlet peak at 6.08 ppm (1,3,5-trimethoxybenzene) was set as 3.00. Yield of different products was determined as following:

Yield of the product of **17** = integration of peak [δ: 6.82 (d, 1H)] / 1.28 × 100%.

Yield of the product of **2** = integration of peak [δ: 6.72 (d, 2H)] / 2.56 × 100%.

Entry	Reductant (mM[a])	Bromo-source (mM[a])	Buffer	Yield of 17 (2)[b]
1[c]	Na_2SO_3, 200 mM	CBr_3CO_2Na, 100 mM	NaPi (pH 8.0)	48% (15%)
2	Na_2SO_3, 100 mM	CBr_3CO_2Na, 100 mM	NaPi (pH 8.0)	60% (21%)
3	Na_2SO_3, 50 mM	CBr_3CO_2Na, 50 mM	NaPi (pH 8.0)	56% (9%)
4	Na_2SO_3, 50 mM	CBr_3CO_2Na, 25 mM	NaPi (pH 8.0)	45% (13%)
5	Na_2SO_3, 25 mM	CBr_3CO_2Na, 50 mM	NaPi (pH 8.0)	53% (11%)
6[c]	Hantzsch ester, 60 mM	CBr_3CO_2Na, 50 mM	Na-acetate (pH 5.0)	52% (4%)
7[c]	Hantzsch ester, 60 mM	$CFBr_2CO_2Et$, 50 mM	Na-acetate (pH 5.0)	10% (10%)
8[c]	Hantzsch ester, 60 mM	CBr_3CO_2Na, 50 mM	Na-citrate (pH 5.0)	45% (7%)
9[c]	Hantzsch ester, 60 mM	CBr_3CO_2Na, 50 mM	NaPi (pH 6.0)	41% (5%)
**10**	**$Cu(MeCN)_4BF_4$, 66 mM**	**$CuBr_2$, 66 mM + KBr, 330 mM**	**Na-acetate (pH 5.0)**	**75% (3%)**
11[d]	$Cu(MeCN)_4BF_4$, 66 mM	$CuBr_2$, 66 mM + KBr, 330 mM	Na-acetate (pH 5.0)	N.R.

Table 16. Optimization of the bromination of the selenonium salt **15**. a) Final concentration in the reaction mixture. b) ^{1}H-NMR yield using1,3,5-trimethoxybenzene as internal standard. c) Reaction mixture contains MeCN 20%(v/v) as cosolvent. d) Reaction conducted in the dark.

Preparation of NAc-3-bromo-tyrosinamide 17

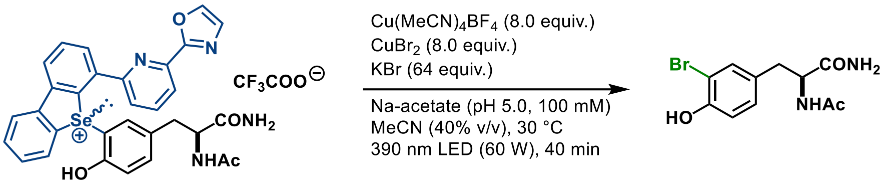

15, 6.3 mM **61%** **17**

Under ambient atmosphere, a scintillation vial (20 mL) equipped with Teflon-coated magnetic stirring bar was charged with the selenonium salt **15** (28 mg, 0.040 mmol, 1.0 equiv.), KBr (310 mg, 2.6 mmol, 64 equiv.), and $CuBr_2$ (72 mg, 0.32 mmol, 8.0 equiv.). Next, 3200 µL of Na-acetate buffer (pH 5.0, c = 200 mM, final concentration c = 100 mM), 640 µL of UHQ-H_2O, and 2560 µL of the cosolvent MeCN (final volume percentage 40%) were added and the mixture was stirred at 25 °C until all solids dissolved. Then the vial was degassed by gently purging argon through the solution via a needle (Φ 0.80 × 120 mm) for 5 min. Thereafter, the reductant $Cu(MeCN)_4BF_4$ (100 mg, 0.32 mmol, 8.0 equiv.) was added and the mixture was degassed for an additional 1 min. Then the vial was closed by a screw cap and the solution was stirred at 25 °C for 5 min. The vial was placed between 2 Kessil PR160-390 nm LEDs (2.5 cm away from each lamp) and irradiated for 40 min. The temperature of the reaction mixture was kept at approximately 30 °C through the use of a cooling fan. Then, to the vial an aqueous solution of EDTA-Na_2 (pH 8.5, c = 0.30 M, 2.5 mL, 0.75 mmol, 0.25 g, 19 equiv.) was added. The solution was saturated by anhydrous Na_2SO_4 (ca. 3 g) and the mixture was extracted by THF/EtOAc (1/1, v/v, 5.0 mL × 4). The organic layers were combined and concentrated to dryness under high vacuum. The residue was purified by the column chromatography on silica gel eluting with DCM/MeOH (30/1–20/1) to afford the desired compound as a yellow solid. Further purification by semi-preparative reverse phase HPLC (YMC-Triart C18, 150 × 20.0 mm, 5 µm) with an eluent mixture of water/MeOH (70/30) at a flow rate of 15.0 mL/min to afford the desired compound as a colorless solid (7.3 mg, 61%).

R*f* = 0.23 (silica gel, DCM/MeOH = 9/1).

NMR Spectroscopy:

^{1}**H NMR** (500 MHz, CD_3OD, 298 K, δ): 7.36 (d, *J* = 2.1 Hz, 1H), 7.04 (dd, *J* = 8.3, 2.2 Hz, 1H), 6.80 (d, *J* = 8.3 Hz, 1H), 4.53 (dd, *J* = 9.1, 5.6 Hz, 1H), 3.03 (dd, *J* = 14.0, 5.6 Hz, 1H), 2.75 (dd, *J* = 13.9, 9.1 Hz, 1H), 1.91 (s, 3H).

^{13}**C NMR** (126 MHz, CD_3OD, 298 K, δ): 176.2, 173.1, 154.3, 134.7, 131.0, 130.4, 117.2, 110.6, 55.9, 37.8, 22.4.

HRMS GC-EI (m/z) calc'd for $C_{11}H_{13}N_2O_3Br^+$ $[M]^+$, 300.0104; found, 300.0106. Deviation: –0.7 ppm.

Chlorination of the selenonium salt 15

Optimization on a small scale

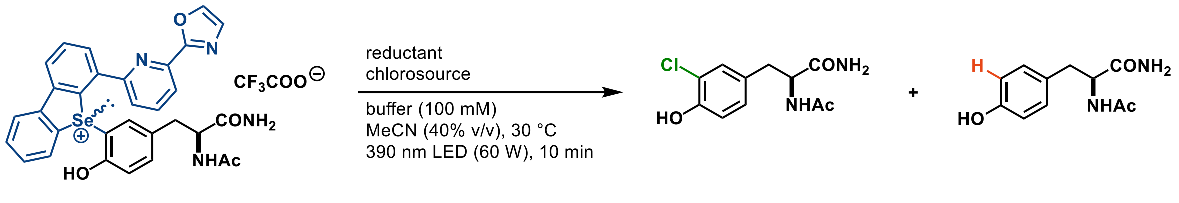

15, 1.6 mM **18** **2**

Under nitrogen atmosphere, a GC-vial (2 mL) equipped with a Teflon-coated mini magnetic stirring bar was charged with the reductant and the chlorosource, followed by the addition of buffer (c = 200 mM, 100 µL, final concentration c = 100 mM), a stock solution of selenonium salt **15** (c = 16 mM, 20 µL, 0.32 µmol, 0.23 mg, 1.0 equiv., final concentration c = 1.6 mM) in MeCN/UHQ-H_2O (1/1, v/v), the cosolvent MeCN (70 µL, final volume percentage 40%), and UHQ-H_2O (10 µL, to reach the final volume of 200 µL). The vial was closed by a screw cap, placed between 2 Kessil PR160-390 nm LEDs (2.5 cm away from each lamp) and irradiated for 10 min. The temperature of the reaction mixture was kept at approximately 30 °C through the use of a cooling fan. Subsequently, the mixture was diluted by 800 µL of MeCN/UHQ-H_2O (1/1, v/v) and was saturated by anhydrous Na_2SO_4 (ca. 0.3 g). The mixture was extracted by THF/EtOAc (1/1, v/v, 0.5 mL × 3) and the organic layers were combined, concentrated under reduced pressure and dried *in vacuo*. The residue was dissolved in CD_3OD and 2.5 µL of a 1,3,5-trimethoxybenzene solution (c = 0.10 M, 0.25 µmol, 42 µg, 0.78 equiv.) in CD_3OD was added as internal standard. The mixture was analyzed via ^{1}H-NMR and a singlet peak at 6.08 ppm (1,3,5-trimethoxybenzene) was set as 3.00. Yield of different products was determined as following:

Yield of the product of **18** = integration of peak [δ: 6.83 (d, 1H)] / 1.28 × 100%.

Yield of the product of **2** = integration of peak [δ: 6.72 (d, 2H)] / 2.56 × 100%.

Entry	Reductant (mM[a])	Chloro-source (mM[a])	Buffer	Yield of 18 (2)[b]
1[c]	Hantzsch ester, 60 mM	CCl_3CO_2Na, 100 mM	Na-acetate (pH 5.0)	0% (48%)
2[c]	$FeSO_4·7H_2O$, 100 mM	CCl_3CO_2Na, 100 mM	Na-acetate (pH 5.0)	21% (42%)
3	–	$CuCl_2$, 50 mM + NaCl, 200 mM	Na-acetate (pH 5.0)	0% (10%)
4[c]	$FeSO_4·7H_2O$, 25 mM	$CuCl_2$, 50 mM + NaCl, 200 mM	Na-acetate (pH 5.0)	20% (8%)
5[c]	$FeSO_4·7H_2O$, 50 mM	$CuCl_2$, 50 mM + NaCl, 200 mM	Na-acetate (pH 5.0)	6% (34%)
**6**	**$Cu(MeCN)_4BF_4$, 33 mM**	**$CuCl_2$, 33 mM + NaCl, 330 mM**	**Na-acetate (pH 5.0)**	**71% (8%)**
7	$Cu(MeCN)_4BF_4$, 33 mM	NaCl, 330 mM	Na-acetate (pH 5.0)	0% (61%)
8[d]	$Cu(MeCN)_4BF_4$, 33 mM	$CuCl_2$, 33 mM + NaCl, 330 mM	Na-acetate (pH 5.0)	N.R.

Table 17. Optimization of the chlorination of the selenonium salt **15**. a) Final concentration in the reaction mixture. b) ^{1}H-NMR yield using1,3,5-trimethoxybenzene as internal standard. c) Reaction mixture contains MeCN 20%(v/v) as cosolvent. d) Reaction conducted in the dark.

Preparation of NAc-3-chloro-tyrosinamide 18

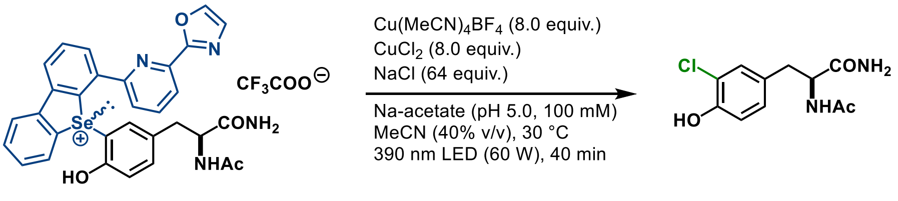

15, 6.3 mM **60%** **18**

Under ambient atmosphere, a scintillation vial (20 mL) equipped with Teflon-coated magnetic stirring bar was charged with the selenonium salt **15** (28 mg, 0.040 mmol, 1.0 equiv.), NaCl (150 mg, 2.6 mmol, 64 equiv.), and $CuCl_2$ (43 mg, 0.32 mmol, 8.0 equiv.). Next, 3200 µL of Na-acetate buffer (pH 5.0, c = 200 mM, final concentration c = 100 mM), 640 µL of UHQ-H_2O, and 2560 µL of the cosolvent MeCN (final volume percentage 40%) were added and the mixture was stirred at 25 °C until all solids dissolved. Then the vial was degassed by gently purging argon through the solution via a needle (Φ 0.80 × 120 mm) for 5 min. Thereafter, the reductant $Cu(MeCN)_4BF_4$ (100 mg, 0.32 mmol, 8.0 equiv.) was added and the mixture was degassed for additional 1 min. The vial was closed by a screw cap and the solution was stirred at 25 °C for 5 min. Then the vial was placed between 2 Kessil PR160-390 nm LEDs (2.5 cm away from each lamp) and irradiated for 40 min. The temperature of the reaction mixture was kept at approximately 30 °C through the use of a cooling fan. Then, to the vial an aqueous solution of EDTA-Na_2 (pH 8.5, 0.30 M, 2.5 mL, 0.75 mmol, 0.25 g, 19 equiv.) was added. The solution was saturated by anhydrous Na_2SO_4 (ca. 3 g) and the mixture was extracted by THF/EtOAc (1/1, 5 mL × 4). The organic layers were combined and concentrated to dryness under high vacuum. The residue was purified by the column chromatography on silica gel eluting with DCM/MeOH (30/1–10/1) to afford the desired compound as a yellow solid. Further purification by semi-preparative reverse phase HPLC (YMC-Triart C18, 150 × 20.0 mm, 5 µm) with an eluent mixture of water/MeOH (55/45) at a flow rate of 15.0 mL/min to afford the desired compound as a colorless solid (6.2 mg, 60%).

R*f* = 0.22 (silica gel, DCM/MeOH = 9/1).

NMR Spectroscopy:

^{1}H NMR (500 MHz, CD_3OD, 298 K, δ): 7.20 (d, *J* = 2.1 Hz, 1H), 7.01 (dd, *J* = 8.3, 2.2 Hz, 1H), 6.82 (d, *J* = 8.3 Hz, 1H), 4.53 (dd, *J* = 9.3, 5.9 Hz, 1H), 3.03 (dd, *J* = 14.0, 5.7 Hz, 1H), 2.76 (dd, *J* = 14.0, 9.0 Hz, 1H), 1.91 (s, 3H).).

^{13}C NMR (126 MHz, CD_3OD, 298 K, δ): 176.2, 173.1, 153.1, 131.6, 130.8, 129.7, 121.4, 117.5, 55.8, 37.9, 22.4.

HRMS ESI (m/z) calc'd for $C_{11}H_{13}N_2O_3ClNa^+$ $[M+Na]^+$, 279.0506(9); found, 279.0507(1). Deviation: –0.1 ppm.

Hydroxylation of the selenonium salt 15

Optimization on a small scale

Direct hydroxylation (failed):

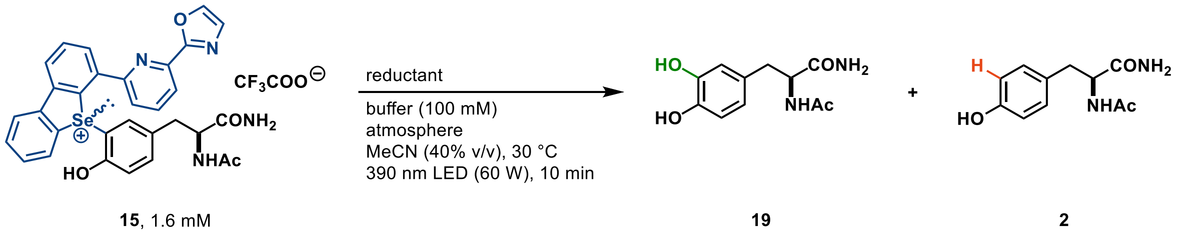

Under ambient or nitrogen atmosphere, a GC-vial (2 mL) equipped with a Teflon-coated mini magnetic stirring bar was charged with the corresponding reductants, followed by the addition of buffer (c = 500 mM, 40 µL, final concentration c = 100 mM), a stock solution of selenonium salt **15** (c = 16 mM, 20 µL, 0.32 µmol, 0.23 mg, 1.0 equiv., final concentration c = 1.6 mM) in MeCN/UHQ-H_2O (1/1, v/v), the cosolvent MeCN (70 µL, final volume percentage 40%), and UHQ-water (70 µL, to reach the final volume of 200 µL). The vial was closed by a screw cap, placed between 2 Kessil PR160-390 nm LEDs (2.5 cm away from each lamp) and irradiated for 10 min. The temperature of the reaction mixture was kept at approximately 30 °C through the use of a cooling fan. Subsequently, the mixture was diluted by 800 µL of MeCN/UHQ-water (1/1, v/v) and was saturated by anhydrous Na_2SO_4 (ca. 0.3 g). The mixture was extracted by THF/EtOAc (1/1, v/v, 500 µL × 3) and the organic layers were combined, concentrated under reduced pressure and dried *in vacuo*. The residue was dissolved in CD_3OD and 2.5 µL of a 1,3,5-trimethoxybenzene solution (c = 0.10 M, 0.25 µmol, 42 µg, 0.78 equiv.) in CD_3OD was added as internal standard. The mixture was analyzed via ^{1}H-NMR and a singlet peak at 6.08 ppm (1,3,5-trimethoxybenzene) was set as 3.00. Yield of the products was determined as following:

Yield of the product of **19** = integration of peak [δ: 6.55 (d, 1H)] / 1.28× 100%.

Yield of the product of **2** = integration of peak [δ: 6.72 (d, 2H)] / 2.56 × 100%.

Entry	Reductant (mM[a])	Atmosphere	Buffer	Yield of 19 (2)[b]
1	ascorbate-Na, 50 mM	nitrogen	Na-citrate (pH 5.0)	0% (0%)
2	ascorbate-Na, 50 mM	air	Na-citrate (pH 5.0)	0% (0%)
3	Hantzsch ester, 50 mM	air	Na-citrate (pH 5.0)	0% (11%)
4	ascorbate-Na, 50 mM	air	Na-citrate (pH 6.0)	0% (0%)
5	ascorbate-Na, 50 mM	air	Na-acetate (pH 5.0)	0% (0%)
6[c]	ascorbate-Na, 50 mM	air	Na-citrate (pH 5.0)	0% (0%)

Table 18. Trials of direct hydroxylation of the selenonium salt **15**. a) Final concentration in the reaction mixture. b) ^{1}H-NMR yield with 1,3,5-trimethoxybenzene as internal standard. c) The reaction was conducted at 0 °C (device see Fig. 53).

Borylation followed by oxidation:

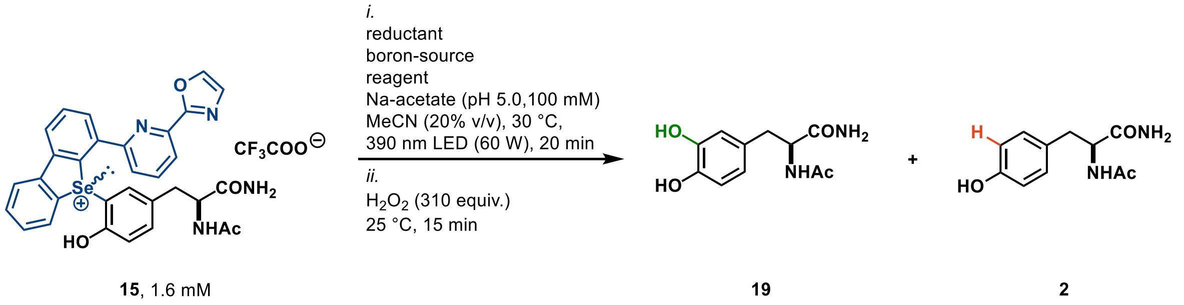

Under nitrogen atmosphere, a GC-vial (2 mL) equipped with a Teflon-coated mini magnetic stirring bar was charged with the corresponding reductants, boronsources, and reagents, followed by the addition of Na-acetate buffer (c = 500 mM, 40 µL, final concentration c = 100 mM), a stock solution of selenonium salt **15** (c = 16 mM, 20 µL, 0.32 µmol, 0.23 mg, 1.0 equiv., final concentration c = 1.6 mM) in MeCN/UHQ-H_2O (1/1, v/v), the cosolvent MeCN (30 µL, final volume percentage 20%), and UHQ-H_2O (110 µL, to reach the final volume of 200 µL). The vial was closed by a screw cap, placed between 2 Kessil PR160-390 nm LEDs (2.5 cm away from the LED) and irradiated for 20 min. The temperature of the reaction mixture was kept at approximately 30 °C through the use of a cooling fan. Subsequently, a stock solution of H_2O_2 (10 wt.%, 34 mg, 34 µL, 0.10 mmol, 3.1 × 10^2 equiv.) in UHQ-H_2O was introduced to convert the borylation product to hydroxylated tyrosine **19**. The mixture was stirred at 25 °C for 10 min, followed by the addition of $Na_2S_2O_4$ (17 mg, 0.10 mmol, 3.1 × 10^2 equiv.). The mixture was stirred at 25 °C for an additional 5 min and then acidified by concentrated hydrochloric acid (37 wt.%, ca. 100 µL) to pH 1 (detected by general pH test paper). Subsequently, the mixture was diluted by 800 µL of MeCN/UHQ-H_2O (1/1, v/v) and saturated by anhydrous Na_2SO_4 (ca. 0.30 g). The mixture was extracted by THF/EtOAc (2/1, 1.0 mL × 4) and the organic layers were combined, concentrated under reduced pressure and dried *in vacuo*. The residue was dissolved in CD_3OD and 2.5 µL of a 1,3,5-trimethoxybenzene solution (c = 0.10 M, 0.25 µmol, 42 µg, 0.78 equiv.) in CD_3OD was added as internal standard. The mixture was analyzed via ^{1}H-NMR and a singlet peak at 6.08 ppm (1,3,5-trimethoxybenzene) was set as 3.00. Yield of the products was determined as following:

Yield of the product of **19** = integration of peak [δ: 6.55 (d, 1H)] / 1.28× 100%.

Yield of the product of **2** = integration of peak [δ: 6.72 (d, 2H)] / 2.56 × 100%.

Entry	Reductant (mM[a])	Boron-source (mM[a])	Reagent (mM[a])	Yield of 19 (2)[b]
1	Hantzsch ester, 40 mM	B_2pin_2, 60 mM	–	0% (45%)
2	Hantzsch ester, 40 mM	B_2pin_2, 60 mM	NaF, 120 mM	40% (36%)
3	Hantzsch ester, 40 mM	B_2pin_2, 200 mM	NaF, 400 mM	56% (16%)
4[c]	Hantzsch ester, 40 mM	B_2pin_2, 200 mM	NaF, 400 mM	48%(11%)
**5**	**Hantzsch ester, 40 mM**	**$B_2(OH)_4$, 400 mM**	**NaF, 400 mM**	**64%(16%)**
6[d]	Hantzsch ester, 40 mM	$B_2(OH)_4$, 400 mM	NaF, 400 mM	N.R.

Table 19. Optimization of the hydroxylation of the selenonium salt **15**. a) Final concentration in the reaction mixture. b) ^{1}H-NMR yieldusing 1,3,5-trimethoxybenzene as internal standard. c) NaPi (pH 8.0, 100 mM) used as buffer. d) Reaction conducted in the dark.

Preparation of NAc-3-hydroxyl-tyrosinamide 19

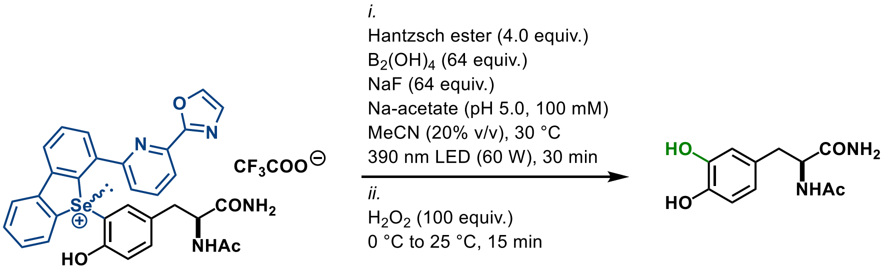

15, 6.3 mM **48%** **19**

Under ambient atmosphere, a scintillation vial (20 mL) equipped with Teflon-coated magnetic stirring bar was charged with the selenonium salt **15** (28 mg, 0.040 mmol, 1.0 equiv.), $B_2(OH)_4$ (230 mg, 2.6 mmol, 64 equiv.), NaF (110 mg, 2.6 mmol, 64 equiv.), and the reductant Hantzsch ester (41 mg, 0.16 mmol, 4.0 equiv.). Next, 3200 µL of Na-acetate buffer (pH 5.0, c = 200 mM, final concentration c = 100 mM), 1920 µL of UHQ-H_2O, and 1280 µL of the cosolvent MeCN (final volume percentage 20%) were added and the mixture was stirred at 25 °C for 5 min. Then the mixture was degassed by gently purging argon through the solution via a needle (Φ 0.80 × 120 mm) for 5 min. Thereafter, the vial was closed by a screw cap, placed between 2 Kessil PR160-390 nm LEDs (2.5 cm away from each lamp) and irradiated for 30 min. The temperature of the reaction mixture was kept at approximately 30 °C through the use of a cooling fan. The reaction mixture was diluted by UHQ-H_2O (4.9 mL) and washed by EtOAc (5.0 mL × 2) to remove the resulting selenide and excessive Hantzsch ester. Then, the mixture was stirred at 0 °C for 5 min and a stock solution of H_2O_2 (10 wt.%, 1.4 g, 1.4 mL, 4.1 mmol, 1.0 × 10^2 equiv.) in UHQ-H_2O was introduced dropwise. The mixture was allowed to warm to 25 °C and stirred at 25 °C for 10 min. Next, $Na_2S_2O_4$ (350 mg, 2.0 mmol, 50 equiv.) was added and the mixture was stirred at 25 °C for 10 min, and then acidified by concentrated hydrochloric acid (37 wt.%) to pH 1 (detected by general pH test paper). Subsequently, the mixture was saturated by anhydrous Na_2SO_4 (ca. 5.0 g) and was extracted by THF/EtOAc (2/1, 5.0 mL × 4). The organic layers were combined, dried over anhydrous Na_2SO_4, filtered, and the solvent evaporated under reduced pressure at 30 °C (**Important:** higher temperature during evaporation can cause unwanted oxidation of the product). The residue was purified by semi-preparative reverse phase HPLC (YMC-Triart C18, 150 × 20.0 mm, 5 µm) with an eluent mixture of water/MeOH (90/10) at a flow rate of 15.0 mL/min to afford the desired compound as a colorless solid (4.6 mg, 48%).

NMR Spectroscopy:

^{1}H NMR (500 MHz, CD_3OD, 298 K, δ): 6.69 – 6.65 (m, 2H), 6.56 (dd, J = 8.1, 2.0 Hz, 1H), 4.51 (dd, J = 8.7, 5.8 Hz, 1H), 2.98 (dd, J = 13.9, 5.8 Hz, 1H), 2.73 (dd, J = 13.9, 8.8 Hz, 1H), 1.92 (s, 3H).

^{13}C NMR (126 MHz, CD_3OD, 298 K, δ): 176.6, 173.1, 146.2, 145.2, 129.9, 121.6, 117.3, 116.2, 56.1, 38.4, 22.5.

HRMS ESI (m/z) calc'd for $C_{11}H_{14}N_2O_4Na^+$ $[M+Na]^+$, 261.0846; found, 261.0848. Deviation: –0.8 ppm.

Coumarin formation of the selenonium salt 15

Optimization on a small scale

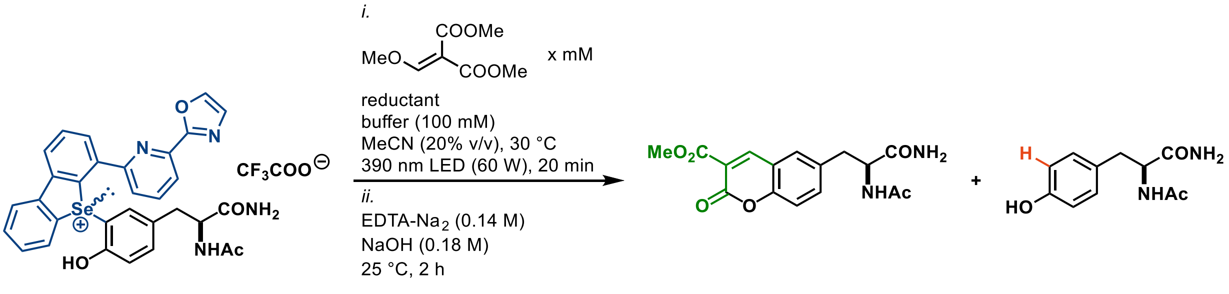

15, 1.6 mM **20** **2**

Under nitrogen atmosphere, a GC-vial (2 mL) equipped with a Teflon-coated mini magnetic stirring bar was charged with the reductant and the Michael acceptor dimethyl 2-(methoxymethylene)malonate,

followed by the addition of buffer (c = 200 mM, 100 µL, final concentration c = 100 mM), a stock solution of selenonium salt **15** (c = 16 mM, 20 µL, 0.32 µmol, 0.23 mg, 1.0 equiv., final concentration c = 1.6 mM) in MeCN/UHQ-H_2O (1/1, v/v), the cosolvent MeCN (30 µL, final volume percentage 20%), and UHQ-H_2O (50 µL, to reach the final volume of 200 µL). The vial was closed by a screw cap and was placed between 2 Kessil PR160-390 nm LEDs (2.5 cm away from each lamp) and irradiated for 20 min. The temperature of the reaction mixture was kept at approximately 30 °C through the use of a cooling fan. Subsequently, 200 µL of an aqueous solution of EDTA-Na_2 (pH 8.5, c = 0.30 M, 60 µmol, 20 mg, 1.9 × 10^2 equiv., final concentration c = 0.14 M) and 40 µL of an aqueous solution of NaOH (c = 2.0 M, 80 µmol, 3.2 mg, 2.5 × 10^2 equiv., final concentration c = 0.18 M) were added to start the coumarin formation. The mixture was stirred at 25 °C for another 2 h and was diluted by 400 µL of MeCN and 160 µL of UHQ-H_2O. To the mixture anhydrous Na_2SO_4 (ca. 0.30 g) was added and the mixture was extracted by THF/EtOAc (1/1, 500 µL × 3). The organic layers were combined, concentrated under reduced pressure and dried *in vacuo*. The residue was dissolved in CD_3OD and 2.5 µL of a 1,3,5-trimethoxybenzene solution (c = 0.10 M, 0.25 µmol, 42 µg, 0.78 equiv.) in CD_3OD was added as internal standard. The mixture was analyzed via ^{1}H-NMR and a singlet peak at 6.08 ppm (1,3,5-trimethoxybenzene) was set as 3.00. Yield of the products was determined as following:

Yield of the product of **20** = integration of peak [δ: 7.34 (d, 1H)] / 1.28× 100%.

Yield of the product of **2** = integration of peak [δ: 6.72 (d, 2H)] / 2.56 × 100%.

Entry	Reductant (mM[a])	x mM[a]	Buffer	Yield of 20 (2)[b]
1	$FeSO_4 \cdot 7H_2O$, 200 mM	400	Na-citrate (pH 5.0)	39% (16%)
2	$FeSO_4 \cdot 7H_2O$, 100 mM	400	Na-citrate (pH 5.0)	55% (16%)
**3**	**$FeSO_4 \cdot 7H_2O$, 50 mM**	**400**	**Na-citrate (pH 5.0)**	**57% (16%)**
4	$FeSO_4 \cdot 7H_2O$, 30 mM	400	Na-citrate (pH 5.0)	48% (16%)
5	$FeSO_4 \cdot 7H_2O$, 10 mM	400	Na-citrate (pH 5.0)	45% (13%)
6	$FeSO_4 \cdot 7H_2O$, 50 mM	600	Na-citrate (pH 5.0)	55% (11%)
7	$FeSO_4 \cdot 7H_2O$, 50 mM	400	Na-citrate (pH 6.0)	25% (19%)
8	$FeSO_4 \cdot 7H_2O$, 50 mM	400	Na-Mes (pH 6.0)	50% (20%)
9	$FeSO_4 \cdot 7H_2O$, 50 mM	400	Tris-HCl (pH 8.0)	51% (16%)
10	ascorbic acid, 50 mM	400	Na-citrate (pH 5.0)	44% (23%)

Table 20. Optimization of the coumarin formation of the selenonium salt **15**. a) Final concentration in the reaction mixture. b) ^{1}H-NMR yield using1,3,5-trimethoxybenzene as internal standard.

Preparation of the coumarin amino acid 20

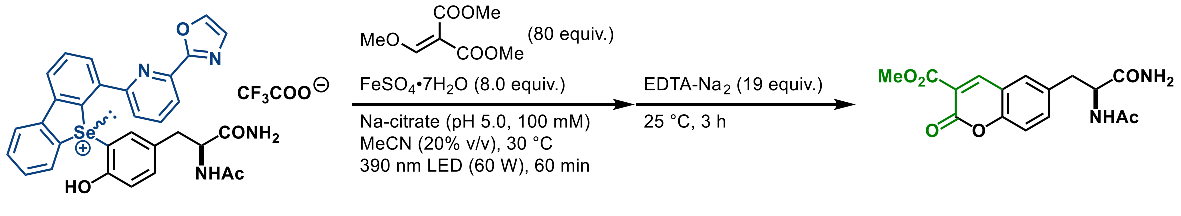

15, 6.3 mM **25%** in 2 steps **20**

Under ambient atmosphere, a scintillation vial (20 mL) equipped with Teflon-coated magnetic stirring bar

was charged with the selenonium salt **15** (28 mg, 0.040 mmol, 1.0 equiv.) and the Michael acceptor dimethyl 2-(methoxymethylene)malonate (560 mg, 3.2 mmol, 80 equiv.). Next, 1280 µL of Na-citrate buffer (pH 5.0, c = 500 mM, final concentration c = 100 mM), 3840 µL of UHQ-H_2O, and 1280 µL of the cosolvent MeCN (final volume percentage 20%) were added and the mixture was stirred at 25 °C until all solids dissolved. Then the mixture was degassed by gently purging argon through the solution via a needle (Φ 0.80 × 120 mm) for 5 min. Thereafter, the reductant $FeSO_4·7H_2O$ (89 mg, 0.32 mmol, 8.0 equiv.) was added and the mixture was degassed for an additional 1 min. The vial was closed by a screw cap and the solution was stirred at 25 °C for 5 min. Then the vial was placed between 2 Kessil PR160-390 nm LEDs (2.5 cm away from each lamp) and irradiated for 60 min. The temperature of the reaction mixture was kept at approximately 30 °C through the use of a cooling fan. Then, to the vial an aqueous solution of EDTA-Na_2 (pH 8.5, c = 0.30 M, 2.5 mL, 0.75 mmol, 0.25 g, 19 equiv.) was added and the pH of the solution was adjusted to approximately 9 by the addition of an aqueous solution of $NaHCO_3$ (c = 1.0 M, ca. 3 mL). The mixture was stirred at 25 °C for 3 h. Subsequently, anhydrous Na_2SO_4 (ca. 3.0 g) was added to the mixture and the aqueous layer was extracted by THF/EtOAc (1/1, v/v, 5.0 mL × 4). The organic layers were combined and concentrated to dryness under high vacuum. The residue was purified by the column chromatography on silica gel eluting with DCM/MeOH (30/1–20/1) to afford the desired compound as a yellow solid. Further purification by semi-preparative reverse phase HPLC (YMC-Triart C18, 150 × 4.6 mm, 5 µm) with an eluent mixture of water/MeOH (65/35) at a flow rate of 15.0 mL/min to afford the desired compound as a colorless solid (3.3 mg, 25%).

R_f = 0.35 (silica gel, DCM/MeOH = 9/1).

NMR Spectroscopy:

^{1}H NMR (600 MHz, DMSO-*d*$_6$, 298 K, δ): 8.68 (s, 1H), 8.07 (d, *J* = 8.5 Hz, 1H), 7.70 (d, *J* = 2.1 Hz, 1H), 7.60 (dd, *J* = 8.5, 2.1 Hz, 1H), 7.45 (s, 1H), 7.36 (d, *J* = 8.5 Hz, 1H), 7.07 (s, 1H), 4.46 (td, *J* = 9.0, 5.1 Hz, 1H), 3.83 (s, 3H), 3.06 (dd, *J* = 13.8, 5.0 Hz, 1H), 2.82 (dd, *J* = 13.8, 9.4 Hz, 1H), 1.77 (s, 3H).

^{13}C NMR (151 MHz, DMSO-*d*$_6$, 298 K, δ): 172.8, 169.1, 163.2, 156.0, 153.3, 148.8, 135.6, 135.0, 130.3, 117.4, 117.3, 115.9, 53.5, 52.5, 36.8, 22.5.

HRMS ESI (m/z) calc'd for $C_{16}H_{16}N_2O_6Na^+$ $[M+Na]^+$, 355.0901; found, 355.0902. Deviation: –0.3 ppm.

Suzuki coupling of the selenonium salt 15

Suzuki coupling on a small scale

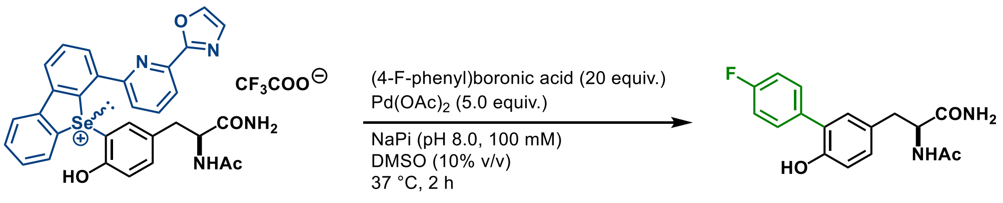

15, 1.0 mM **75%** **21**

Yield determination: A singlet peak at –108.0 ppm (4-fluorobenzotrifluoride) was set as 1.00. Yield of the product was determined to be 75% as following:

Yield of the product of **21** = integration of peak [δ: –116.93 – –117.04 (m, 1F)] / 1.00 × 100%.

Preparation of the NAc-3-(*p*F-phenyl)-Tyr-NH_2 21

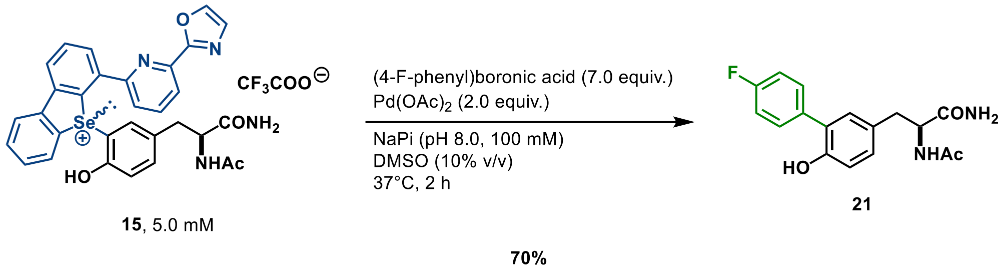

Under ambient atmosphere, a scintillation vial (20 mL) equipped with Teflon-coated magnetic stirring bar was charged with the selenonium salt **15** (35 mg, 0.050 mmol, 1.0 equiv.), the coupling partner (4-fluorophenyl)boronic acid (49 mg, 0.35 mmol, 7.0 equiv.), and the catalyst $Pd(OAc)_2$ (23 mg, 0.10 mmol, 2.0 equiv.). Next, 5.0 mL of NaPi buffer (pH 8.0, c = 200 mM, final concentration c = 100 mM) and 4.0 mL of UHQ-H_2O were added. The suspension was stirred at 37 °C for 5 min. Thereafter, 1.0 mL of the cosolvent DMSO (final volume percentage 10%) was introduced to the mixture to start the reaction and the mixture was stirred at 37 °C for 2 h. Next, 2 mL of an aqueous solution of HCl (2 M) was introduced. The resulting black suspension was diluted by MeOH (5 mL) and filtered through a pad of silica gel (ca. 2 g). The residue was rinsed by MeOH (10 mL × 3) and the filtrate was combined and concentrated to dryness under high vacuum. The residue was purified by the chromatography on silica gel eluting eluting with DCM/MeOH (1/0–20/1) to afford the desired compound as a colorless solid (11 mg, 70%).

R*f* = 0.30 (silica gel, DCM/MeOH = 10/1).

NMR Spectroscopy:

^{1}H NMR (500 MHz, CD_3OD, 298 K, δ): 7.60 – 7.52 (m, 2H), 7.15 (d, *J* = 2.3 Hz, 1H), 7.09 (t, *J* = 8.9 Hz, 2H), 7.04 (dd, *J* = 8.2, 2.3 Hz, 1H), 6.81 (d, *J* = 8.2 Hz, 1H), 4.58 (dd, *J* = 8.8, 5.7 Hz, 1H), 3.08 (dd, *J* = 13.9, 5.8 Hz, 1H), 2.82 (dd, *J* = 13.9, 8.9 Hz, 1H), 1.91 (s, 3H).

^{13}C NMR (126 MHz, CD_3OD, 298 K, δ): 176.5, 173.1, 164.2, 162.3, 154.2, 136.38, 136.35, 132.4, 132.2, 132.1, 130.3, 129.7, 128.7, 117.0, 115.6, 115.5, 56.0, 38.3, 22.5.

^{19}F NMR (471 MHz, CD_3OD, 298 K, δ): –118.6.

HRMS ESI (m/z) calc'd for $C_{17}H_{17}N_2O_3FNa^+$ $[M+Na]^+$, 339.1115; found, 339.1117. Deviation: –0.6 ppm.

Protein residue tolerance study of DBSeOPy-oxa 1

Summary of residue tolerance study of DBSeOPy-oxa 1

Considering that selenoxide is a medium-strong oxidant, we examined the compatibility of the selenoxide **1** to oxidation-vulnerable amino acids (cysteines, methionines, and tryptophans) under the standard conditions (pH 3.0, 30 °C, 18 h) used for tyrosinamide **2** modification but with 1.0 equivalent **1**. Cysteine was converted to cystine **S16** in 98% yield even at lower temperature (25 °C) and in a shorter reaction time (30 min). The oxidation of methionine was not observed under the standard conditions. Tryptophan also remained untouched (92% recovery) under the standard conditions but **1** was decomposed to the selenide **S9** in 46% yield.

Reaction between *N*-acetyl-L-cysteine-methylester and 1

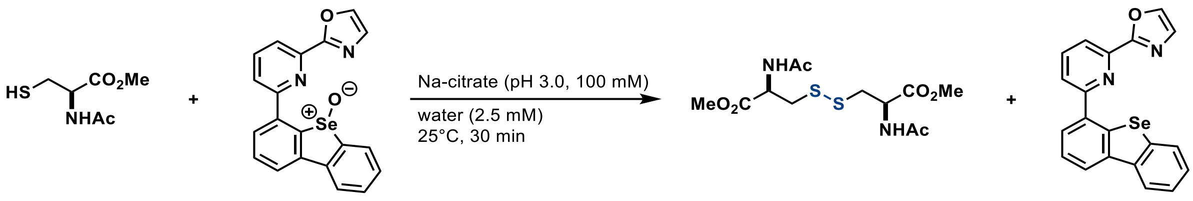

1 **S16** 98% yield **S9** 94% yield

Under ambient atmosphere, a round-bottom flask (50 mL) equipped with Teflon-coated magnetic stirring bar was charged with the selenoxide **1** (20 mg, 0.050 mmol, 1.0 equiv., final concentration c = 2.5 mM) and 10 mL of Na-citrate buffer (pH 3.0, c = 200 mM, final concentration c = 100 mM). The mixture was stirred at 25 °C for 10 min. Then, a solution of *N*-acetyl-L-cystein-methylester (c = 5.0 mM, 10 mL, 0.050 mmol, 8.9 mg, 1.0 equiv., final concentration c = 2.5 mM) in UHQ-H_2O was added and the mixture was stirred at 25 °C for another 30 min. Subsequently, the mixture was transferred to a separatory funnel and extracted by chloroform (10 mL × 3). The organic layers were combined, dried over anhydrous Na_2SO_4, filtered, and the solvent evaporated under reduced pressure. The residue was purified by the column chromatography on silica gel eluting with hexanes/EtOAc (1/1) to afford both the cystine derivative **S16** as a colorless solid (8.6 mg, 98%) and the selenide **S9** as a (colorless solid(18 mg, 94%).

R_f (S16) = 0.25 (silica gel, DCM/MeOH = 20/1).

NMR Spectroscopy:

^{1}H NMR (500 MHz, $CDCl_3$, 298 K, δ): 6.53 (d, *J* = 7.6 Hz, 2H), 4.87 (dt, *J* = 7.4, 5.1 Hz, 2H), 3.77 (s, 6H), 3.26 – 3.14 (m, 4H), 2.06 (s, 6H).

^{13}C NMR (126 MHz, $CDCl_3$, 298 K, δ): 171.0, 170.2, 52.9, 51.9, 40.9, 23.3.

HRMS GC-EI (m/z) calc'd for $C_{12}H_{20}N_2O_6S_2Na^+$ $[M+Na]^+$, 375.0655; found, 375.0658. Deviation: –0.8 ppm.

Reaction between *N*-acetyl-L-methionine-methylester and 1

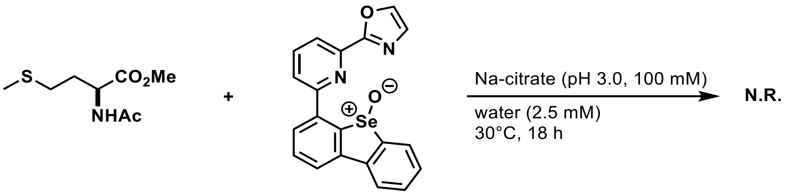

1

*Preparation of the stock solutions of the selenoxide **1**:* Under ambient atmosphere, a scintillation vial (4 mL) was charged with the selenoxide **1** (20 mg, 0.050 mmol, final concentration c = 50 mM) and 1.0 mL of a H_3PO_4 solution (c = 0.10 M) in UHQ-water. The suspension was heated at 90 °C for 30 min to give a yellow solution. After cooling to 25 °C, the resulting solution was stored in the dark at 25 °C and directly used for reactions without any further operations.

Reaction set-up: Under ambient atmosphere, a round-bottom flask (50 mL) equipped with Teflon-coated magnetic stirring bar was charged with 4.0 mL of Na-citrate buffer (pH 3.0, c = 500 mM, final concentration c = 100 mM), 13 mL of UHQ-water, and 1.0 mL of the selenoxide stock solution (c = 50 mM, 0.050 mmol, 20 mg, 1.0 equiv., final concentration c = 2.5 mM) in H_3PO_4 solution (c = 0.10 M, final concentration c = 5.0 mM) in UHQ-water. The mixture was stirred at 30 °C for 30 min followed by the addition of 2.0 mL of an *N*-acetyl-L-methionine-methylester stock solution (c = 25 mM, 0.050 mmol, 10 mg, 1.0 equiv., final concentration c = 2.5 mM) in UHQ-water. Then, the mixture was stirred at 30 °C for 18 h. Subsequently, the mixture was extracted with chloroform (10 mL × 4). The organic layers were combined, dried over anhydrous Na_2SO_4, filtered, and the solvent evaporated under reduced pressure. The residue was dissolved in $CDCl_3$ (ca. 0.5 mL) and 7.0 µL of CH_2Br_2 (0.10 mmol, 18 mg, 2.0 equiv) was added as internal standard. The mixture was analyzed via ^{1}H-NMR and a singlet peak at 5.16 ppm (CH_2Br_2) was set as 4.00. *N*-Acetyl-L-methionine-methylester was recovered in95% yield. (Recovery = integration of peak [δ: 3.74 (s, 3H)]/3 × 100%)

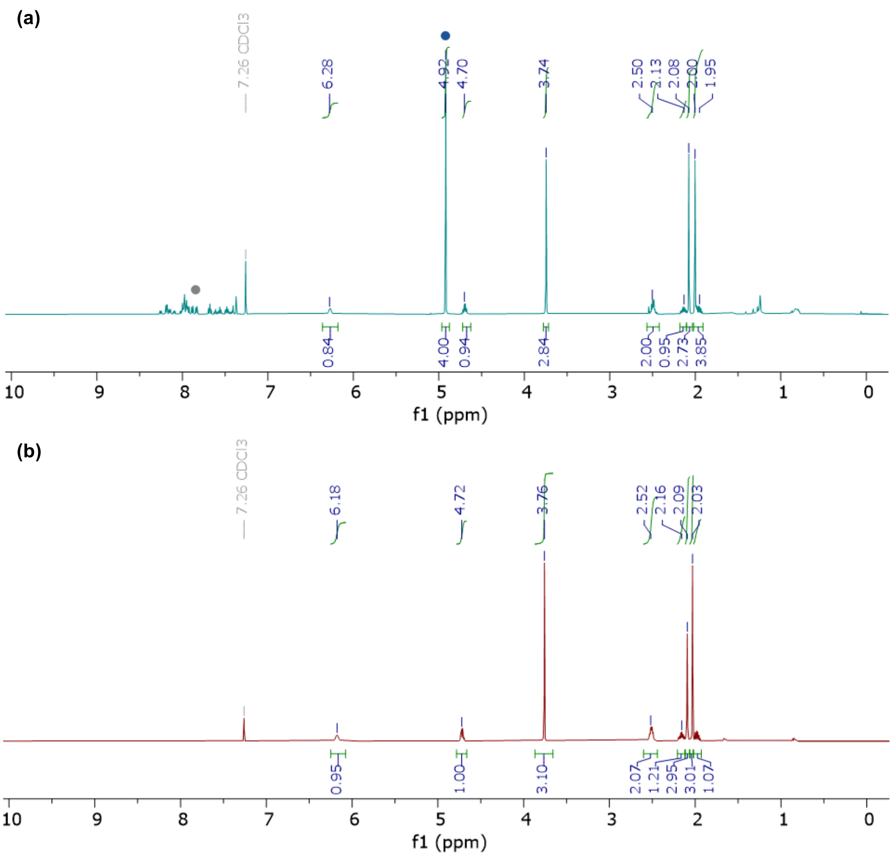

Figure 30. ^{1}H-NMR of a) the reaction crude mixture and b) the methionine starting material in $CDCl_3$. Blue spot, CH_2Br_2; grey spot, selenoxide **1** (^{1}H-NMR resonance range: 7.30–8.50 ppm).

Reaction between *N*-acetyl-L-tryptophan-methylester and 1

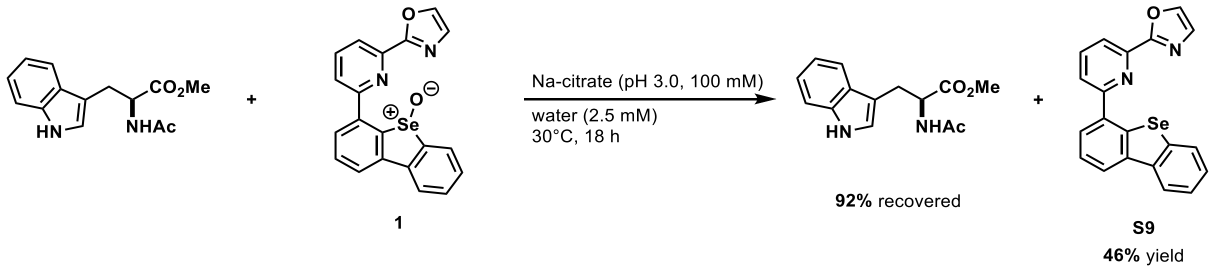

*Preparation of the stock solutions of the selenoxide **1**:* Under ambient atmosphere, a scintillation vial (4 mL) was charged with the selenoxide **1** (20 mg, 0.050 mmol, final concentration c = 50 mM) and 1.0 mL of a H_3PO_4 solution (c = 0.10 M) in UHQ-water. The suspension was heated at 90 °C for 30 min to give a yellow solution. After cooling to 25 °C, the resulting solution was stored in the dark at 25 °C and directly used for reactions without any further operations.

Reaction set-up: Under ambient atmosphere, a round-bottom flask (50 mL) equipped with Teflon-coated magnetic stirring bar was charged with 4.0 mL of Na-citrate buffer (pH 3.0, c = 500 mM, final concentration c = 100 mM), 13 mL of UHQ-water, and 1.0 mL of the selenoxide stock solution (c = 50 mM, 0.050 mmol, 20 mg, 1.0 equiv., final concentration c = 2.5 mM) in H_3PO_4 solution (c = 0.10 M, final concentration c = 5.0 mM) in UHQ-water. The mixture was stirred at 30 °C for 30 min followed by the addition of 2.0 mL of an *N*-acetyl-L-tryptophan-methylester suspension (0.050 mmol, 13 mg, 1.0 equiv., final concentration c = 2.5 mM) in UHQ-water. Then, the mixture was stirred at 30 °C for 18 h.

Subsequently, the mixture was transferred to a separatory funnel and extracted with chloroform (10 mL × 3). The organic layers were combined, dried over anhydrous Na_2SO_4, filtered, and the solvent evaporated under reduced pressure. The residue was purified by the column chromatography on silica gel eluting with hexanes/EtOAc (1/1) followed by DCM/MeOH (10/1–4/1) to afford the selenide **S9** as a colorless solid (8.6 mg, 46%) and to recover the starting material *N*-acetyl-L-tryptophan-methylester as a beige solid (12 mg, 92%) as well as the unreacted selenoxide **1** as a colorless solid (9.0 mg, 46%).

Synthesis of *Se*-modified peptides and proteins

Quantification of *Se*-modified peptides and proteins

Due to the specific absorption at 328 nm of the tyrosine-selenonium chromophores in the bioconjugation products, the compound **15** was used as a proxy to prepare the LC calibration curve for the quantification of *Se*-modified peptides and proteins. The LC injection volume was 5 µL and the absorption peak of the proxy **15** was integrated at 328 nm by PDA analysis.

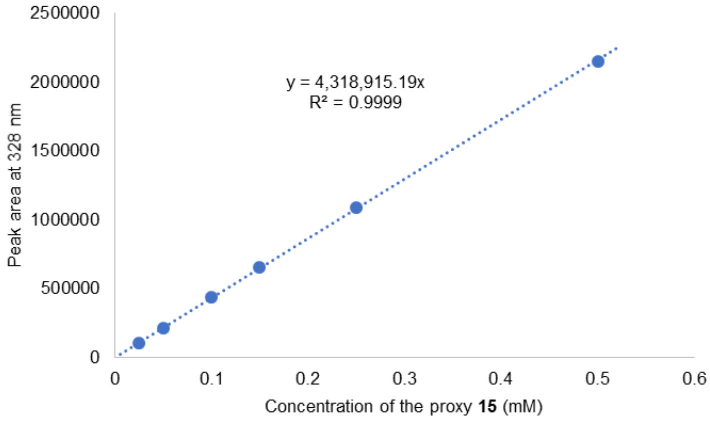

Figure 31. LC calibration curve of the proxy **15** (LC-MS Method A).

Determination of the concentration of the tyrosine-selenonium chromophores in bioconjuagtion products was performed via peak integration after PDA analysis at 328 nm. With the standard curve, the concentration of the chromophores in the samples can be calculated as follows:

$$\text{chromophore concentration (mM)} = \frac{\text{peak area of the desired product/LC injection volume (µL)}}{\text{the slope of the standard curve (mM}^{-1})/5\ \text{(µL)}}$$

For the product with only one selenonium modification, the yield was calculated as follows:

$$\text{yield} = \frac{\text{chromophore concentration (mM)} \times \text{sample volume (µL)}}{\text{total peptide or protein amount used in reaction (nmol)}} \times 100\%$$

However, due to the unknown extinction coefficients of newly formed species, the determined yields should be considered semi-quantitative.

Data on protein starting materials

The LC calibration curves of bivalirudin, insulin, ubiquitin, and ribonuclease A were prepared for quantification aims. Data in the standard curves was obtained as an average of three or four runs (technical repetition) with error bars representing standard error of the mean (SEM). The LC injection volume in all experiments was 5 µL except the ones for ribonuclease A (injection volume was 1 µL). The absorption peak of each peptide or protein was integrated at 214 nm by PDA analysis.

The mark of "*" in the amino acid sequences indicates the end of the sequence.

Oxytocin

Amino acid sequence: CYIQNCPLG-NH_2*, MW: 1007.19 (one disulfide bond)

Bivalirudin

Amino acid sequence: FPRPGGGGNGDFEEIPEEYL*, MW: 2180.32

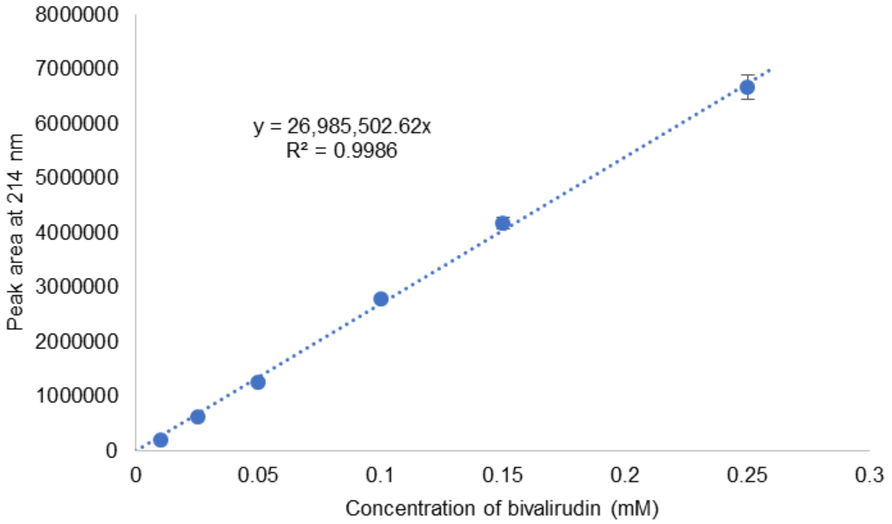

Figure 32. LC calibration curve of bivalirudin (LC-MS method A). Technical repetition, 3 times; error bar represents standard error of the mean (SEM).

Angiotensin I

Amino acid sequence: DRVYIHPFHL*, MW: 1296.48 (anhydrous free base basis)

Pramlintide

Amino acid sequence: KCNTATCATQRLANFLVHSSNNFGPILPPTNVGSNTY-NH_2*, MW: 3949.39

Human insulin

Amino acid sequence of human insulin (all cysteines form disulfide bonds, three disulfide bonds):

A-chain: GIVEQCCTSICSLYQLENYCN*

B-chain: FVNQHLCGSHLVEALYLVCGERGFFYTPKT*

Calculated MW: 5807.63; found: 5807.64

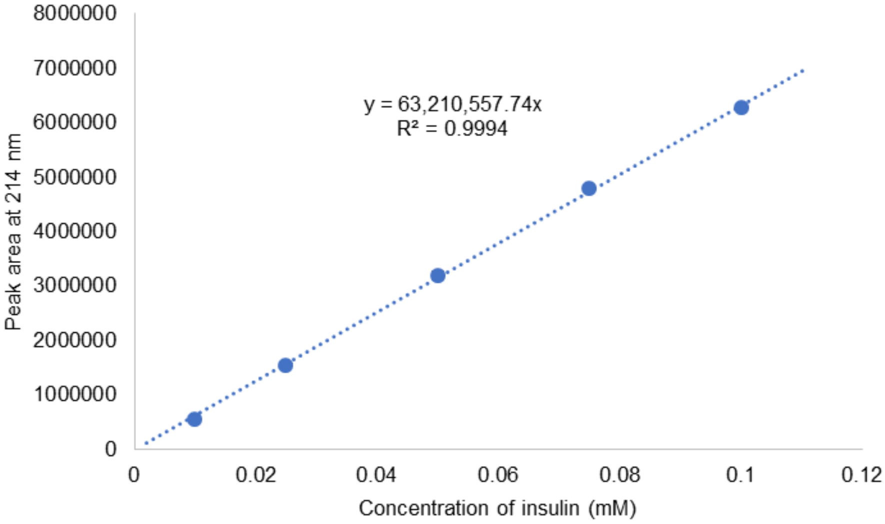

Figure 33. LC calibration curve of insulin (LC-MS method A). Technical repetition, 3 times; error bar represents standard error of the mean (SEM).

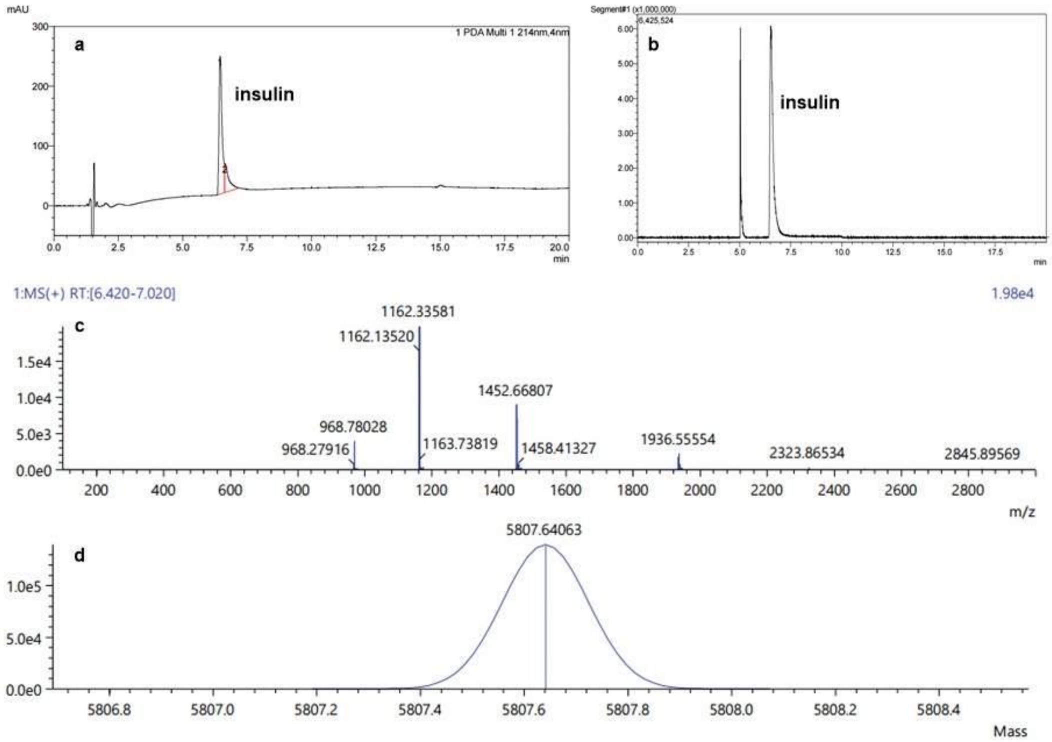

Figure 34. LC-MS analysis (Method C) of insulin: a) PDA trace at 214 nm b) Total ion chromatogram c) Insulin peak ion series d) Deconvoluted zero-charged spectrum.

Bovine ubiquitin (from erythrocytes)

Amino acid sequence of bovine ubiquitin (with oxidized methionine):

MQIFVKTLTGKTITLEVEPSDTIENVKAKIQDKEGIPPDQQRLIFAGKQLEDGRTLSD**Y**NIQKESTLHLVLRLRGG*

Calculated MW: 8564.63; found: 8564.69 (ubiquitin)

Calculated MW: 8580.63; found: 8580.60 (ubiquitin with S-methionine oxide)

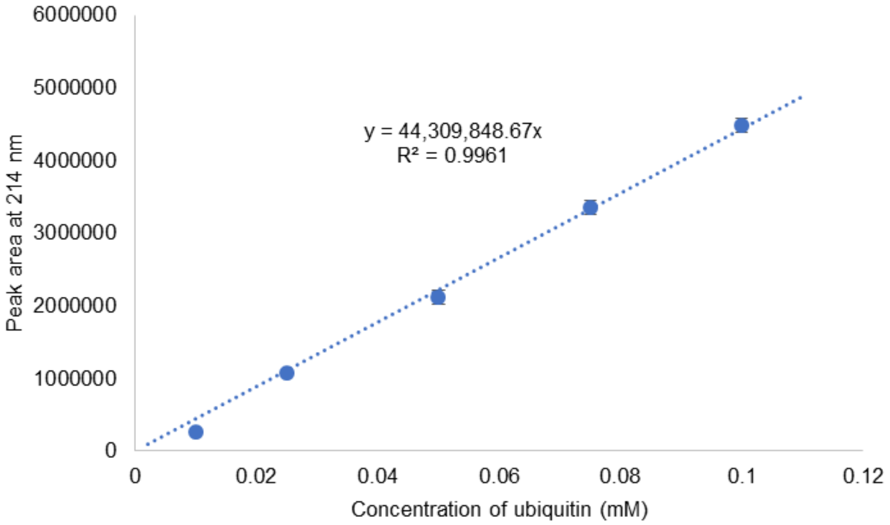

Figure 35. LC calibration curve of ubiquitin (LC-MS Method A). Technical repetition, 4 times; error bar represents standard error of the mean (SEM).

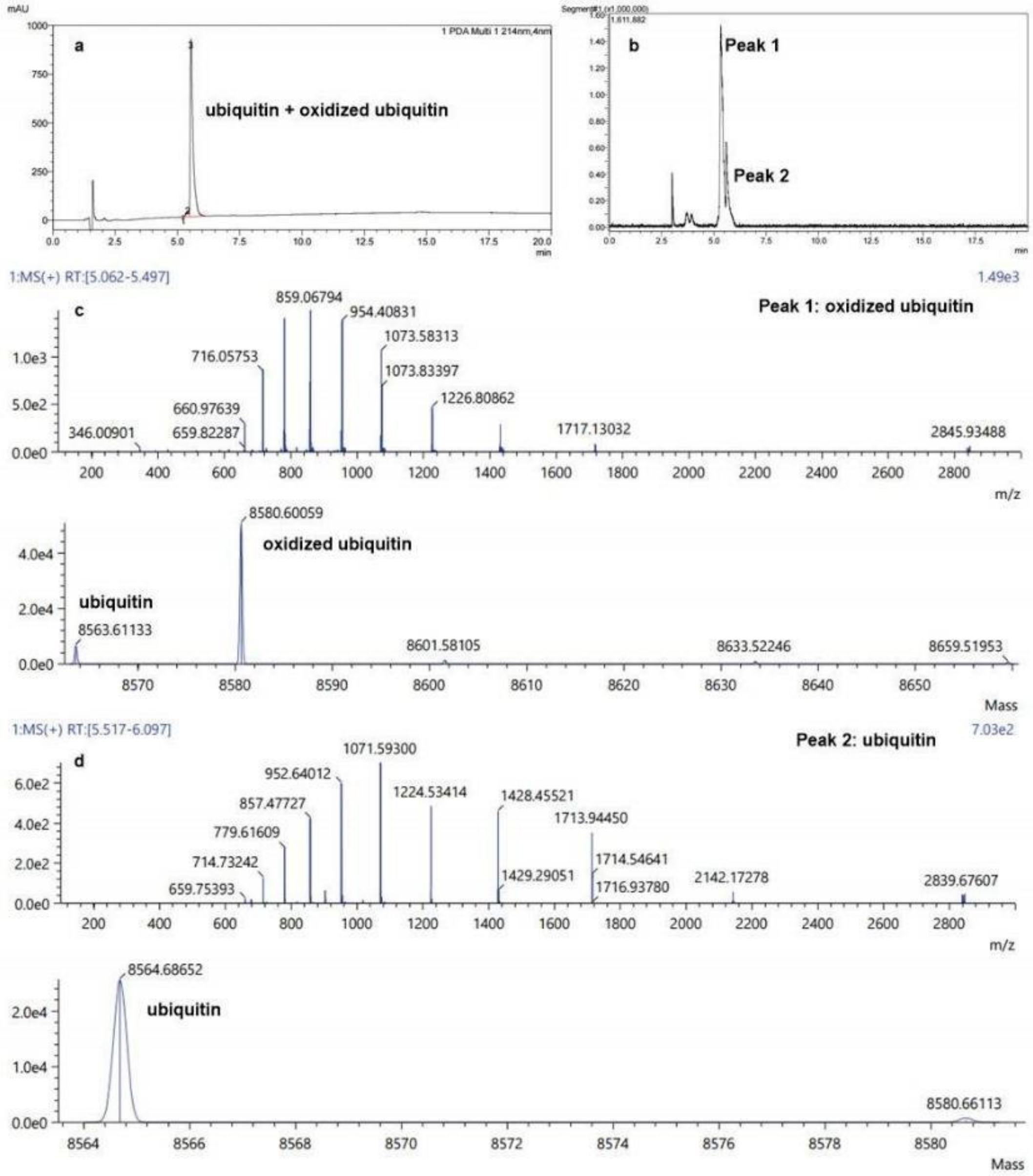

Figure 36. LC-MS analysis (Method C) of ubiquitin: a) PDA trace at 214 nm b) Total ion chromatogram c) Peak 1 (oxidized ubiquitin) ion series and deconvoluted zero-charged spectrum d) Peak 2 (ubiquitin) ion series and deconvoluted zero-charged spectrum.

Ribonuclease A

Amino acid sequence of ribonuclease A from bovine pancreas:

KETAAAKFERQHMDSSTSAASSSN**Y**CNQMMKSRNLTKDRCKPVNTFVHESLADVQAVCSQKNVACKN

GQTNC**Y**QS**Y**STMSITDCRETGSSK**Y**PNCA**Y**KTTQANKHIIVACEGNP**Y**VPVHFDASV*

Calculated MW (averaged): 13682.28.

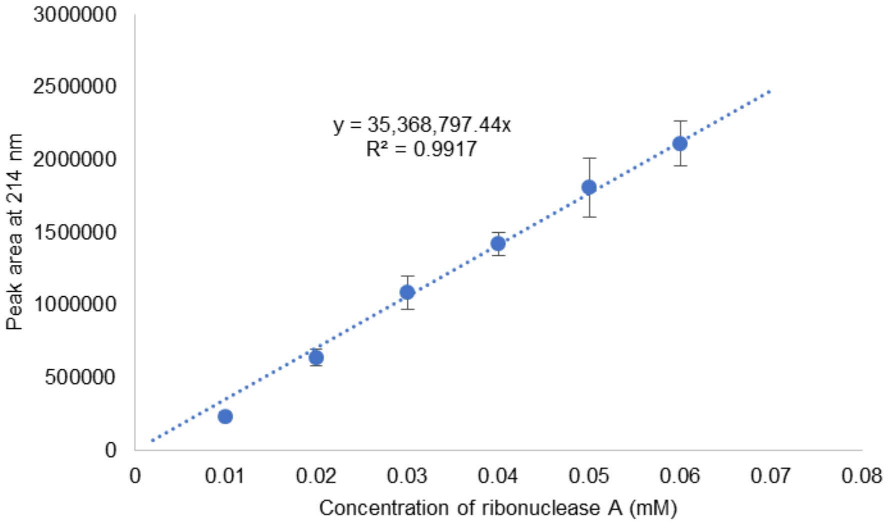

Figure 37. LC calibration curve of ribonuclease A (LC-MS Method A). Technical repetition, 3 times; error bar represents standard error of the mean (SEM).

Human lysozyme

Amino acid sequence of human lysozyme (recombinant, expressed in rice):

KVFERCELARTLKRLGMDG**Y**RGISLANWMCLAKWESG**Y**NTRATN**Y**NAGDRSTD**Y**GIFQINSR**Y**WCNDG

KTPGAVNACHLSCSALLQDNIADAVACAKRVVRDPQGIRAWVAWRNRCQNRDVRQ**Y**VQGCGV*

Calculated MW (averaged): 14692.51.

Preparation of oxytocin-selenonium conjugate 7

Preparation in a low concentration (100 μM)

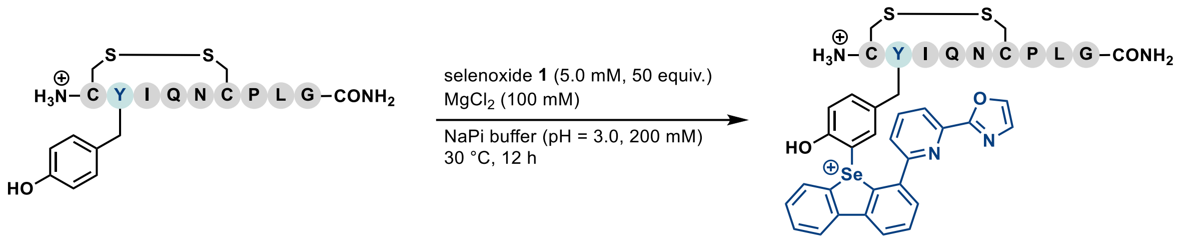

oxytocin, 100 μM **92%** **7, oxytocin-selenonium conjugate**

Under ambient atmosphere and at 20–25 °C, 40 μL of NaPi buffer (pH 3.0, c = 500 mM, final concentration c = 200 mM), 5.0 μL of an $MgCl_2$ stock solution (c = 2.0 M, 10 μmol, 0.95 mg, 1.0 × 10^3 equiv., final concentration c = 0.10 M) in UHQ-H_2O, and 35 μL of UHQ-H_2O were added to an Eppendorf tube (1.5 mL). Then, 10 μL of a selenoxide **1** stock solution (c = 50 mM, 0.50 μmol, 0.20 mg, 50 equiv., final concentration c = 5.0 mM) in phosphoric acid solution (c = 0.10 M) in UHQ-H_2O was added. The mixture was vortexed for 5 sec., transferred into a Thermocycler pre-heated at 30 °C and incubated at 30 °C for 30 min at 600 rpm. Next, 10 μL of an oxytocin stock solution (c = 1.0 mM, 10 nmol, 10 μg, 1.0 equiv., final concentration c = 0.10 mM) in UHQ-H_2O was introduced to start the reaction. The mixture was vortexed for 5 sec., transferred into a Thermocycler pre-heated at 30 °C, and incubated at 30 °C for 12 h at 600 rpm. Subsequently, 100 μL of MeCN (final volume percentage 50%) was added and the mixture was vortexed for 5 sec., transferred into a Thermocycler pre-heated at 25 °C, and incubated at 25 °C for 10 min at 600 rpm. Subsequently, 15 μL of a freshly prepared Na_2SO_3 stock solution (c = 50 mM, 0.75 μmol, 95 μg, 75 equiv.) in UHQ-H_2O was added to reduce excess selenoxides. Again, the mixture was vortexed for 5 sec., transferred into a Thermocycler pre-heated at 25 °C, and incubated at 25 °C for 10 min at 600 rpm. The mixture was washed with ethyl acetate (100 μL × 2) to remove the selenide. Theaqueous layer was gently purged by argon 2–4 mm above the solution via a needle (Φ 0.80 × 40 mm) for 90 sec. to remove residual organic solvent. The solution volume was adjusted to 100 μL by the addition of UHQ-H_2O (15 μL). Then, 100 μL of MeCN (final volume percentage 50%) was added and the resulting mixture was directly used for LC-MS analysis for yield determination. The mixture is stable at 4 °C for at least 6 months.

Yield (in triplicate) was determined by LC-MS post-run PDA analysis at 328 nm:

$$\frac{196585\ (A328\ area)/5\ (LC\ injection\ volume, \mu\mathrm{L})}{4318915.2\ (the\ slope\ of\ the\ std.curve, \mathrm{mM}^{-1})/5\ (\mu\mathrm{L})} = 0.046\ (selenonium\ motif\ concentration, \mathrm{mM})$$

$$\frac{0.046\ (\mathrm{mM}) \times 200\ (sample\ volume, \mu\mathrm{L})}{10\ (oxytocin\ amount, \mathrm{nmol})} \times 100\% = 92\%\ (yield)$$

HRMS ESI-pos (m/z) calc'd for $C_{63}H_{77}N_{14}O_{13}S_2Se^+$ $[M]^+$, 1381.4396; found, 1381.4392. Deviation: 0.3 ppm.

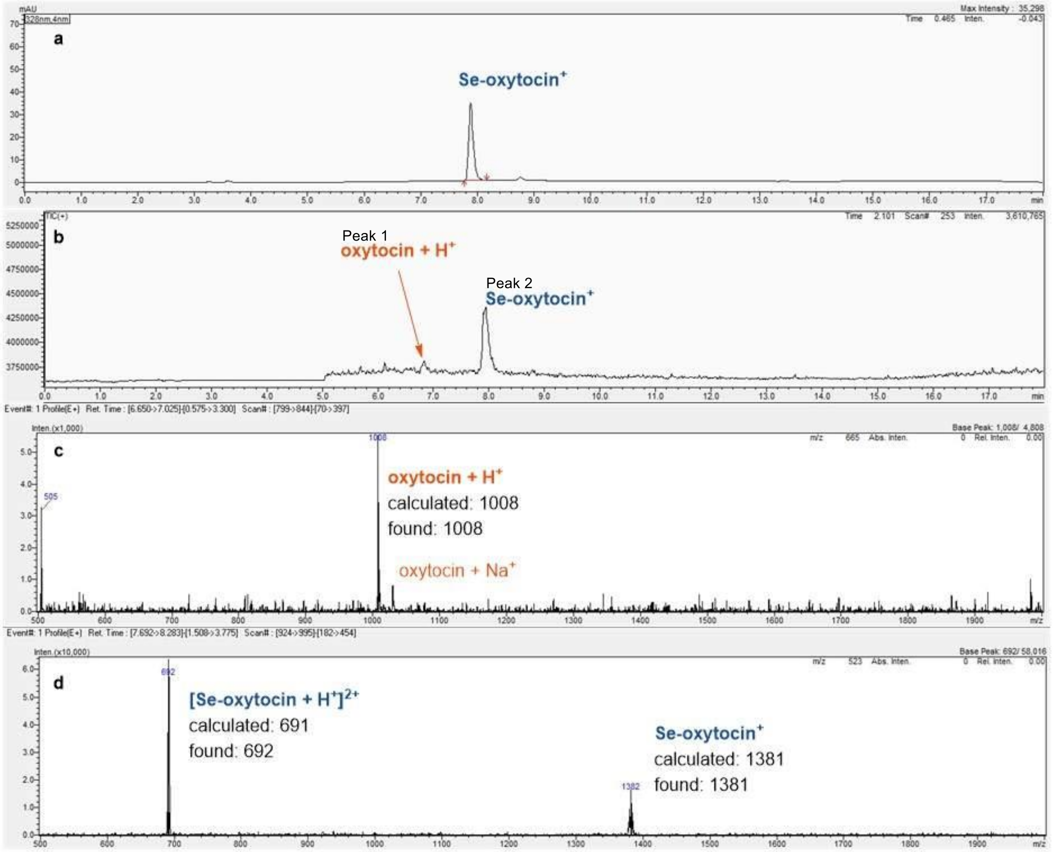

Figure 38. LC-MS analysis (Method A) of oxytocin-selenonium conjugate **7**. a) LC chromatogram (PDA at 328 nm) b) Total ion chromatogram c) Peak 1 (starting material) ion series d) Peak 2 (product) ion series.

Sample preparation of 7 for HRMS/MS analysis

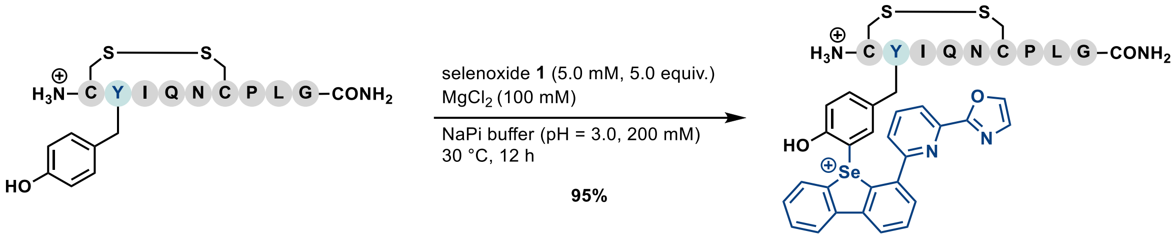

oxytocin, 1.0 mM

7, oxytocin-selenonium conjugate

Under ambient atmosphere and at 20–25 °C, 40 µL of NaPi buffer (pH 3.0, c = 500 mM, final concentration c = 200 mM), 5.0 µL of an $MgCl_2$ stock solution (c = 2.0 M, 10 µmol, 0.95 mg, 1.0 × 10^2 equiv., final concentration c = 0.10 M) in UHQ-H_2O, and 25 µL of UHQ-H_2O were added to an Eppendorf tube (1.5 mL). Then, 10 µL of a selenoxide **1** stock solution (c = 50 mM, 0.50 µmol, 0.20 mg, 5.0 equiv., final concentration c = 5.0 mM) in phosphoric acid solution (c = 100 mM) in UHQ-H_2O was added. The mixture was vortexed for 5 sec., transferred into a Thermocycler pre-heated at 30 °C and incubated at 30 °C for 30 min at 600 rpm. Next, 20 µL of an oxytocin stock solution (c = 5.0 mM, 0.10 µmol, 0.10 mg, 1.0 equiv., final concentration c = 1.0 mM) in UHQ-H_2O was introduced to start the reaction. The mixture was vortexed for 5 sec., transferred into a Thermocycler pre-heated at 30 °C, and incubated at 30 °C for 12 h at 600 rpm. Subsequently, 100 µL of MeCN (final volume percentage 50%) was added and the mixture was vortexed for 5 sec., transferred into a Thermocycler pre-heated at 25 °C, and incubated at 25 °C for 10 min at 600 rpm. Subsequently, 15 µL of a freshly prepared Na_2SO_3 stock solution (c = 50

mM, 0.75 µmol, 95 µg, 75 equiv.) in UHQ-H_2O was added to reduce excess selenoxides. Again, the mixture was vortexed for 5 sec., transferred into a Thermocycler pre-heated at 25 °C, and incubated at 25 °C for 10 min at 600 rpm. The mixture was washed with ethyl acetate (100 µL × 2) to remove the selenide. The aqueous layer was gently purged by argon 2–4 mm above the solution via a needle (Φ 0.80 × 40 mm) for 90 sec. to remove residual organic solvent. The solution volume was adjusted to 100 µL by the addition of UHQ-H_2O (15 µL). Then, 100 µL of MeCN (final volume percentage 50%) was added and the resulting mixture was used for HRMS/MS sample preparation without any further purification. Yield was determined by LC-MS post-run PDA analysis at 328 nm to be 95% (average of three experiments).

HRMS/MS sample preparation:

Under ambient atmosphere and at 20–25 °C, 50 µL of the oxytocin-selenonium conjugate **7** stock solution (c = 0.48 mM, 24 nmol, 33 µg, 1.0 equiv.) in the 1/1 (v/v) mixture of NaPi buffer (pH 3.0, c = 100 mM) containing $MgCl_2$ (c = 50 mM) and MeCN (50% v/v) was added to an Eppendorf tube (1.5 mL). Then, 2 µL of a NaOH stock solution (c = 2.0 M) in UHQ-H_2O was introduced to adjust the solution pH to ca. 7–8. Next, 4.8 µL of a DTT stock solution (c = 0.20 M, 0.96 µmol, 0.15 mg, 40 equiv.) in UHQ-H_2O was added. The mixture was vortexed for 5 sec., transferred into a Thermocycler pre-heated at 37 °C, and incubated at 37 °C for 1 h at 600 rpm. The resulting mixture was directly used for HRMS/MS analysis.

HRMS/MS results

b^+	b^{2+}		Sequence		y^+	y^{2+}
104.0165	52.5119	1	C	9	1383.4553	692.2313<br>**692.2310 (0.4)**
641.0757<br>**641.0764 (–1.1)**	321.0415	2	**Y + Se**	8	1280.4461	640.7267
754.1597<br>**754.1601 (–0.5)**	377.5835	3	I	7	743.3869<br>**743.3875 (–0.8)**	372.1971
882.2183<br>**882.2188 (–0.6)**	441.6128	4	Q	6	630.3029<br>**630.3033 (–0.6)**	315.6551
996.2612<br>**996.2625 (–1.3)**	498.6343	5	N	5	502.2443<br>**502.2445 (–0.4)**	251.6258
1099.2704<br>**1099.2721 (–1.5)**	550.1389<br>**550.1391 (–0.4)**	6	C	4	388.2013<br>**388.2013 (0)**	194.6043
1196.3232	598.6653<br>**598.6657 (–0.7)**	7	P	3	285.1922<br>**285.1921 (0.4)**	143.0997
1309.4073	655.2073<br>**655.2081 (–1.2)**	8	L	2	188.1394<br>**188.1394 (0)**	94.5734
1365.4447	683.2260	9	G-$CONH_2$	1	75.0553	38.0313

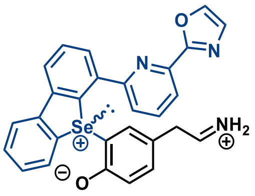

Immonium ion

m/z calculated: 510.0715

m/z found: 510.0721

Table 21. HRMS/MS analysis of reduced oxytocin-selenonium conjugate **7**. Sample measured after reduction with 40 equiv. DTT for 1 h at 37 °C. Peptide concentration for analytical sample: 0.58 mg/mL. b^{n+}, peptide fragment starting from the N-terminus of the analyte with n positive charges; y^{n+}, peptide fragment starting from the C-terminus of the analyte with n positive charges; the integer, residue numbers in the corresponding fragments, integer in blue or orange indicates the corresponding b or y fragment

was found, respectively; the number with 4 decimals in black, theoretical m/z numbers for the corresponding fragment; the bold numbers with 4 decimals in blue or orange, found m/z numbers for the corresponding b or y fragment, respectively; the number in bracket, the deviation of the theoretical m/z number from the found m/z number in ppm.

Preparation of bivalirudin-selenonium conjugate 8

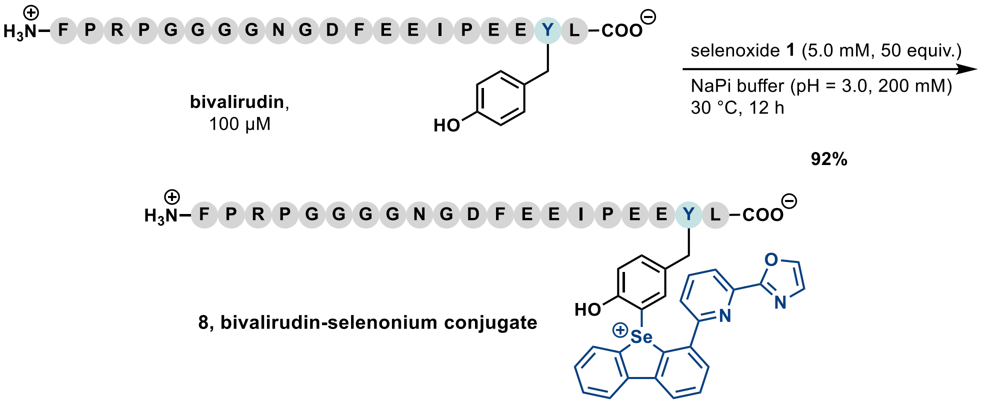

Under ambient atmosphere and at 20–25 °C, 120 µL of NaPi buffer (pH 3.0, c = 500 mM, final concentration c = 200 mM) and 120 µL of UHQ-H_2O were added to an Eppendorf tube (1.5 mL). Then, 30 µL of a selenoxide **1** stock solution (c = 50 mM, 1.5 µmol, 0.60 mg, 50 equiv., final concentration c = 5.0 mM) in phosphoric acid solution (c = 100 mM) in UHQ-H_2O was added. The mixture was vortexed for 5 sec., transferred into a Thermocycler pre-heated at 30 °C and incubated at 30 °C for 30 min at 600 rpm. Next, 30 µL of a bivalirudin stock solution (c = 1.0 mM, 30 nmol, 66 µg, 1.0 equiv., final concentration c = 0.10 mM) in UHQ-H_2O was introduced to start the reaction. The mixture was vortexed for 5 sec., transferred into a Thermocycler pre-heated at 30 °C, and incubated at 30 °C for 12 h at 600 rpm. Subsequently, 300 µL of MeCN (final volume percentage 50%) was added and the mixture was vortexed for 5 sec., transferred into a Thermocycler pre-heated at 25 °C, and incubated at 25 °C for 20 min at 600 rpm. Subsequently, 45 µL of a freshly prepared Na_2SO_3 stock solution (c = 50 mM, 2.3 µmol, 0.29 mg, 75 equiv.) in UHQ-H_2O was added to reduce excess selenoxides. Again, the mixture was vortexed for 5 sec., transferred into a Thermocycler pre-heated at 25 °C, and incubated at 25 °C for 10 min at 600 rpm. The mixture was washed with ethyl acetate (300 µL × 2) to remove selenide. The aqueous layer was gently purged by argon 2–4 mm above the solution via a needle (Φ 0.80 × 40 mm) for 2 min to remove residual organic solvent. The solution volume was adjusted to 300 µL by the addition of UHQ-H_2O (ca. 50 µL). The resulting mixture was directly used for LC-MS analysis for yield determination as well as HRMS and HRMS/MS analysis for product characterization and site-selectivity determination. The mixture was also used for subsequent transformations without any further purification, and was stable at 4 °C for at least 1 month.

Yield was determined by LC-MS post-run PDA analysis at 328 nm to be 92% (average of three experiments).

HRMS ESI-pos (m/z) calc'd for $C_{118}H_{151}N_{26}O_{34}Se^{3+}$ [M+2H]$^{3+}$, 852.0012; found, 852.0017. Deviation: –0.6 ppm.

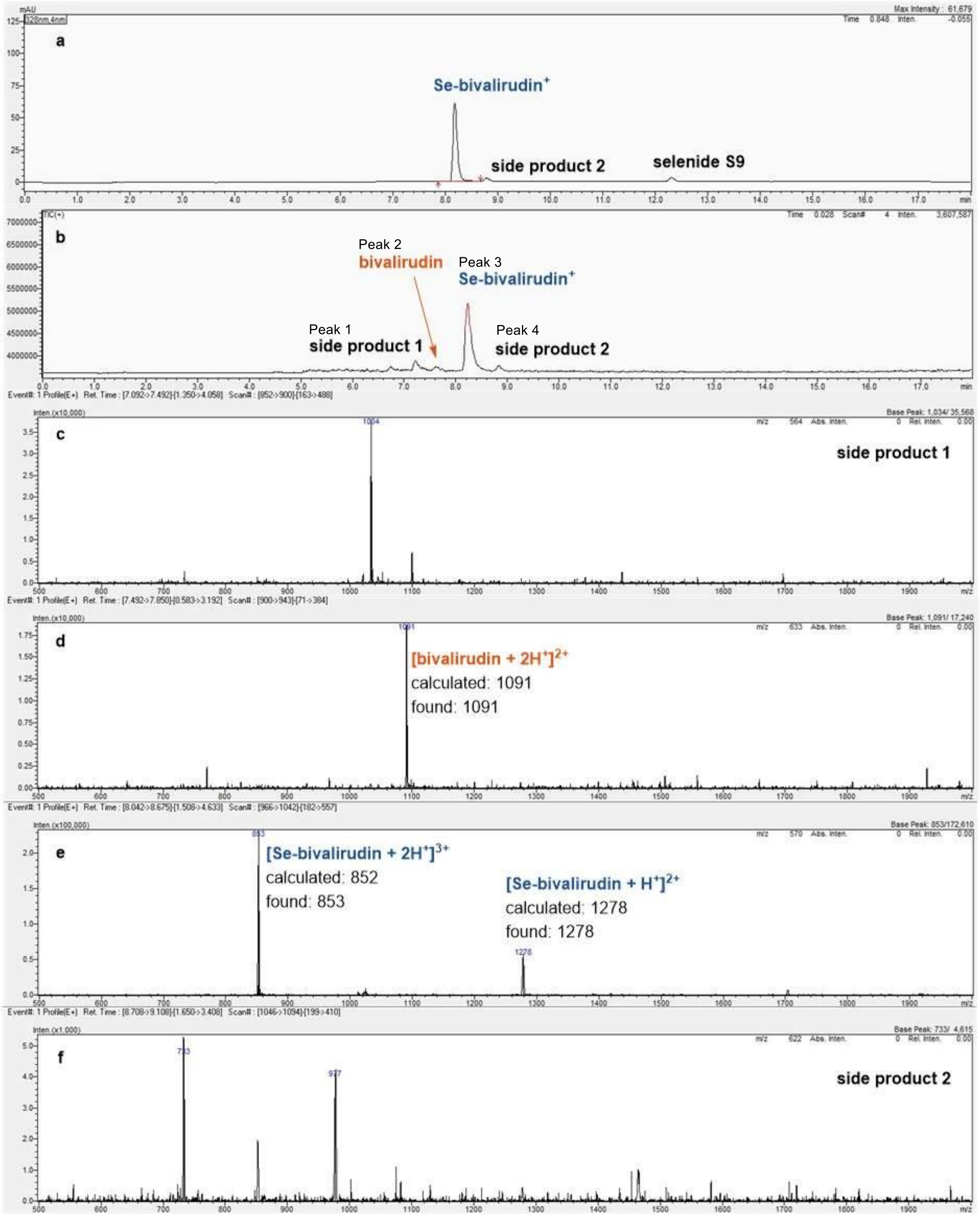

Figure 39. LC-MS analysis (Method A) of bivalirudin-selenonium conjugate **8**. a) LC chromatogram (PDA 328 nm) b) Total ion chromatogram c) Peak 1 (side product 1) ion series d) Peak 2 (starting material) ion series e) Peak 3 (product) ion series f) Peak 4 (side product 2) ion series.

Assessment of side-reactivity

In order to reveal side reactivities of the reaction, we looked for two reaction pathways in addition to the desired reaction with the selenoxide reagent:

1. Cleavage of peptide bond: the product is one amino acid less than the starting material: FPRPGGGGNGDFEEIPEEY, MW: 2067.15.
   Calculated for [side product 1 + 2H$^+$]$^{2+}$: 1034, found: 1034.
2. Modification by a second selenoxide: the product is one selenonium motif more than the product: [FPRPGGGGNGDFEEIPEE(Y + 2Se)L]$^{2+}$, MW: 2928.90.
   Calculated for [side product 2 + H$^+$]$^{3+}$: 977, found: 977.

Calculated for [side product 2 + $2H^+$]$^{4+}$: 733, found: 733.

HRMS/MS results

b^+	b^{2+}	b^{3+}		Sequence		y^+	y^{2+}
148.0757	74.5415	50.0301	**1**	F	**20**	2553.9890	1277.4982
245.1285<br>**245.1282 (1.2)**	123.0679	82.3811	**2**	P	**19**	2406.9206	1203.9639
401.2296<br>**401.2293 (0.7)**	201.1185	134.4148	**3**	R	**18**	2309.8678	1155.4376
498.2824	249.6449	166.7657	**4**	P	**17**	2153.7667	1077.3870<br>**1077.3870 (0)**
555.3038<br>**555.3040 (–0.4)**	278.1556	185.7728	**5**	G	**16**	2056.7139	1028.8606
612.3253<br>**612.3256 (–0.5)**	306.6663	204.7800	**6**	G	**15**	1999.6925	1000.3499
669.3468<br>**669.3476 (–1.2)**	335.1770	223.7871	**7**	G	**14**	1942.6710	971.8392
726.3682<br>**726.3688 (–0.8)**	363.6878	242.7943	**8**	G	**13**	1885.6495	943.3284
840.4112<br>**840.4113 (–0.1)**	420.7092	280.8086	**9**	N	**12**	1828.6281	914.8177
897.4326<br>**897.4337 (–1.2)**	449.2200	299.8158	**10**	G	**11**	1714.5851	857.7962
1012.4596<br>**1012.4613 (–1.7)**	506.7334<br>**506.7336 (–0.4)**	338.1581	**11**	D	**10**	1657.5637	829.2855
1159.5280<br>**1159.5292 (–1.0)**	580.2677<br>**580.2674 (0.5)**	387.1809	**12**	F	**9**	1542.5367	771.7720<br>**771.7709 (1.4)**
1288.5706<br>**1288.5696 (0.8)**	644.7889<br>**644.7891 (–0.3)**	430.1951	**13**	E	**8**	1395.4683<br>**1395.4677 (0.4)**	698.2378
1417.6132<br>**1417.6135 (–0.2)**	709.3102<br>**709.3104 (–0.3)**	473.2093	**14**	E	**7**	1266.4257<br>**1266.4254 (0.2)**	633.7165
1530.6972<br>**1530.6968 (0.3)**	765.8523<br>**765.8521 (0.3)**	510.9040	**15**	I	**6**	1137.3831<br>**1137.3842 (–1.0)**	569.1952<br>**569.1948 (0.7)**
1627.7500	814.3787<br>**814.3782 (0.6)**	543.2549	**16**	P	**5**	1024.2991<br>**1024.3002 (–1.1)**	512.6532<br>**512.6530 (0.4)**
1756.7926	878.9000<br>**878.8990 (1.1)**	586.2691	**17**	E	**4**	927.2463<br>**927.2458 (0.5)**	464.1268
1885.8352	943.4213<br>**943.4210 (0.3)**	629.2833	**18**	E	**3**	798.2037<br>**798.2031 (0.8)**	399.6055
2422.8943	1211.9508	808.6377<br>**808.6371 (0.7)**	**19**	**Y + Se**	**2**	669.1611<br>**669.1609 (0.3)**	335.0842
2535.9784	1268.4929	845.9977	**20**	L	**1**	132.1020	66.5546

Table 22. HRMS/MS analysis of bivalirudin-selenonium conjugate **8**. Sample was directly measured without any reduction. Peptide concentration for analytical sample: 0.21 mg/mL. b^{n+}, peptide fragment starting from the N-terminus of the analyte with n positive charges; y^{n+}, peptide fragment starting from the C-terminus of the analyte with n positive charges; the integer, residue numbers in the corresponding fragments, integer in blue or orange indicates the corresponding b or y fragment was found, respectively; the number with 4 decimals in black, theoretical m/z numbers for the corresponding fragment; the bold numbers with 4 decimals in blue or orange, found m/z numbers for the corresponding b or y fragment, respectively; the number in bracket, the deviation of the theoretical m/z number from the found m/z number in ppm.

Preparation of angiotensin I-selenonium conjugate 9

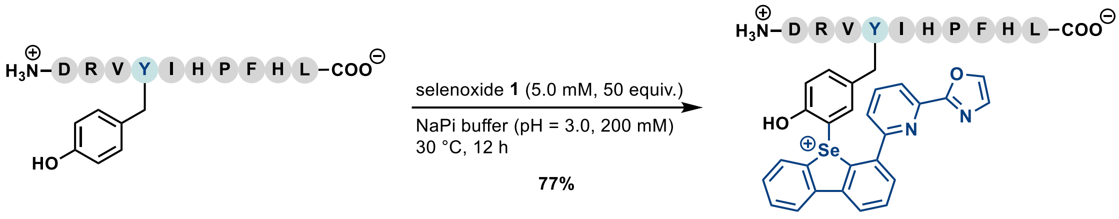

Under ambient atmosphere and at 20–25 °C, 40 µL of NaPi buffer (pH 3.0, c = 500 mM, final concentration c = 200 mM), 45 µL of UHQ-H_2O, and 10 µL of a selenoxide **1** stock solution (c = 50 mM, 0.50 µmol, 0.20 mg, 50 equiv., final concentration c = 5.0 mM) in phosphoric acid solution (c = 0.10 M) in UHQ-H_2O were added to an Eppendorf tube (1.5 mL). The mixture was vortexed for 5 sec., transferred into a Thermocycler pre-heated at 30 °C and incubated at 30 °C for 30 min at 600 rpm. Next, 5.0 µL of an angiotensin I stock solution (c = 2.0 mM, 10 nmol, 13 µg, 1.0 equiv., final concentration c = 0.10 mM) in UHQ-H_2O was added. The mixture was vortexed for 5 sec., transferred into a Thermocycler pre-heated at 30 °C, and incubated at 30 °C for 12 h at 600 rpm. Subsequently, 100 µL of MeCN (final volume percentage 50%) was added and the mixture was vortexed for 5 sec., transferred into a Thermocycler pre-heated at 25 °C, and incubated at 25 °C for 20 min at 600 rpm. Subsequently, 15 µL of a freshly prepared Na_2SO_3 stock solution (c = 50 mM, 0.75 µmol, 95 µg, 75 equiv.) in UHQ-H_2O was added to reduce excess selenoxides. Again, the mixture was vortexed for 5 sec., transferred into a Thermocycler pre-heated at 25 °C, and incubated at 25 °C for 10 min at 600 rpm. The mixture was washed with ethyl acetate (100 µL × 2) to remove the selenide. The aqueous layer was gently purged by argon 2–4 mm above the solution via a needle (Φ 0.80 × 40 mm) for 2 min to remove residual organic solvent. The solution volume was adjusted to 100 µL by the addition of UHQ-H_2O (ca. 15 µL). Then, 100 µL of MeCN (final volume percentage 50%) was added and the resulting mixture was directly used for LC-MS analysis for yield determination as well as HRMS and LC-FTMS/MS analysis for product characterization and site-selectivity determination.

Yield was determined by LC-MS post-run PDA analysis at 328 nm to be 77% (average of three experiments).

HRMS ESI-pos (m/z) calc'd for $C_{82}H_{102}N_{19}O_{15}Se^{3+}$ $[M+2H]^{3+}$, 557.5655; found, 557.5656. Deviation: –0.2 ppm.

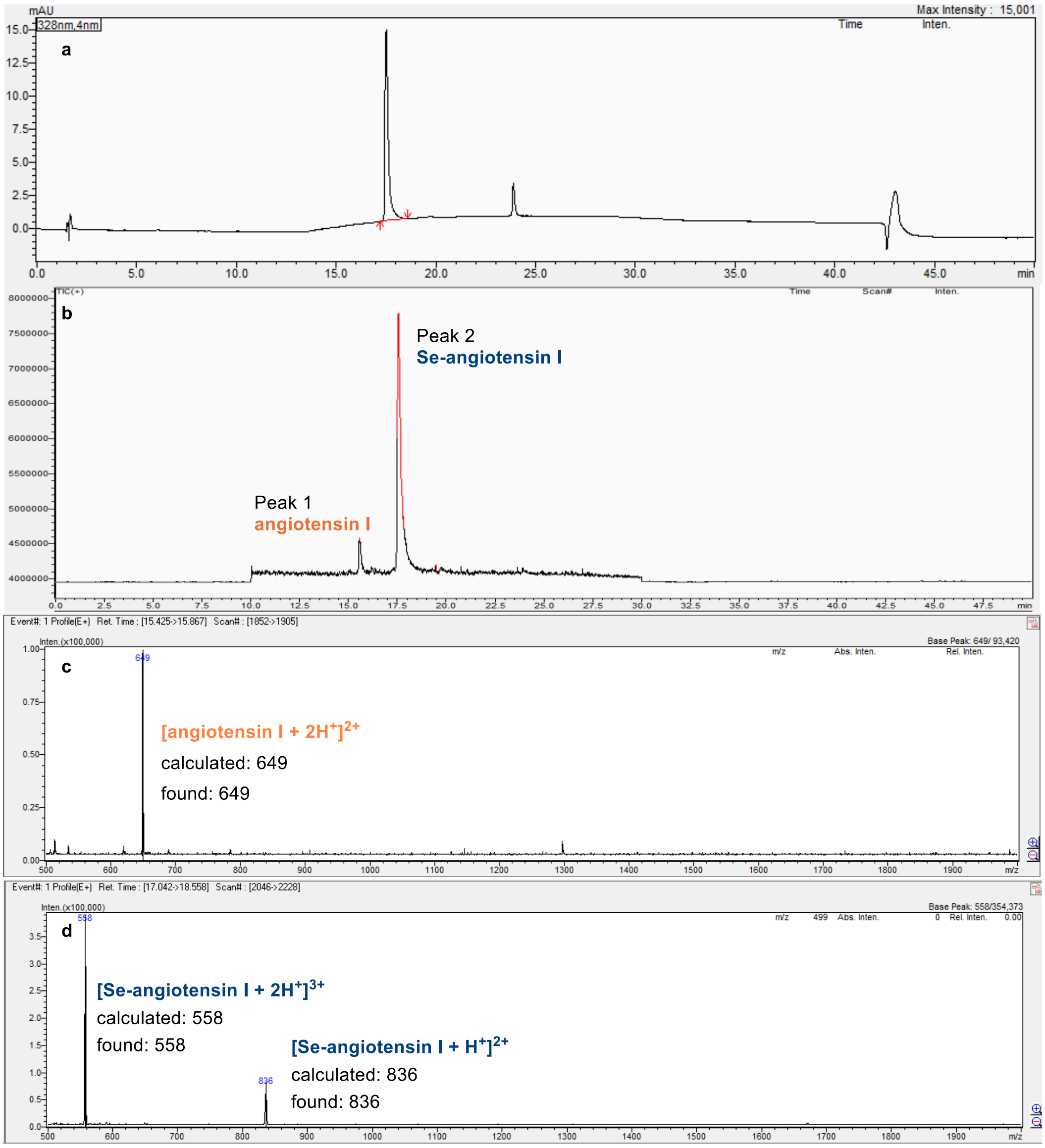

Figure 40. LC-MS analysis (Method A) of angiotensin I-selenonium conjugate **9**. a) LC chromatogram (PDA 328 nm) b) Total ion chromatogram c) Peak 1 (starting material) ion series d) Peak 2 (product) ion series.

LC-FTMS/MS results

Based on the LC-FTMS/MS analysis, the modification site Y4 was confirmed by the three detected ions; $(a3)^+$ Ion without modification from D1 to V3 and $(a5)^+$ ion with a possible modification from D1 to I5 indicated the modification site would be Y4 or I5. In addition to this, the presence of $(x6)^{4+}$ or $(z6)^{4+}$ indicated no modification from I5 to L10, resulting Y4 is the only modification site.

a	b		Sequence		x	y	z
		**1**	D	**10**			
	**b$^+$**: 272.1353<br>**272.1358 (–1.6)**	**2**	R	**9**			
**a$^+$**: 343.2088<br>**343.2090 (–0.5)**		**3**	V	**8**			
		**4**	**Y-Se**	**7**			
**a^{2+}**: 497.1797<br>**497.1813 (–3.2)**	**b^{2+}**: 511.1771<br>**511.1786 (–2.9)**	**5**	I	**6**	**x^{2+}**: 395.2058<br>**395.2059 (–0.4)**		**z^{2+}**: 374.2068<br>**374.2069 (–0.3)**

**a**$^{2+}$: 565.7901<br>**565.7091 (+0.1)**	**b**$^{2+}$: 579.7066<br>**579.7072 (–1.1)**	**6**	H	**5**	**x**$^{2+}$: 338.6637<br>**338.6641 (–1.2)**	**y**$^{2+}$: 325.6741<br>**325.6743 (–0.7)**	**z**$^{2+}$: 317.6647<br>**317.6650 (–0.8)**
		**7**	P	**4**		**y**$^{+}$: 513.2820<br>**513.2819 (+0.3)**	
**a**$^{2+}$: 687.7697<br>**687.7703 (–0.9)**	**b**$^{2+}$: 701.7672<br>**701.7679 (–1.1)**	**8**	F	**3**		**y**$^{+}$: 416.2292<br>**416.2294 (–0.3)**	
**a**$^{3+}$: 504.5352<br>**504.5362 (–2.0)**		**9**	H	**2**		**y**$^{+}$: 269.1608<br>**269.1610 (–0.7)**	
		**10**	L	**1**		**y**$^{+}$: 132.1019<br>**132.1021 (–1.7)**	

Table 23. MS/MS analysis of angiotensin I-selenonium conjugate **9**. Peptide concentration for analytical sample: 0.13 mg/mL. a^{n+} and b^{n+}, peptide fragment starting from the N-terminus of the analyte with n positive charges; x^{n+}, y^{n+}, and z^{n+}, peptide fragment starting from the C-terminus of the analyte with n positive charges; the integer, residue numbers in the corresponding fragments, integer in blue or orange indicates the corresponding b or y fragment was found, respectively; the number with 4 decimals in black, theoretical m/z numbers for the corresponding fragment; the bold numbers with 4 decimals in blue or orange, found m/z numbers for the corresponding b or y fragment, respectively; the number in bracket, the deviation of the theoretical m/z number from the found m/z number in ppm.

Preparation of pramlintide-selenonium conjugate 10

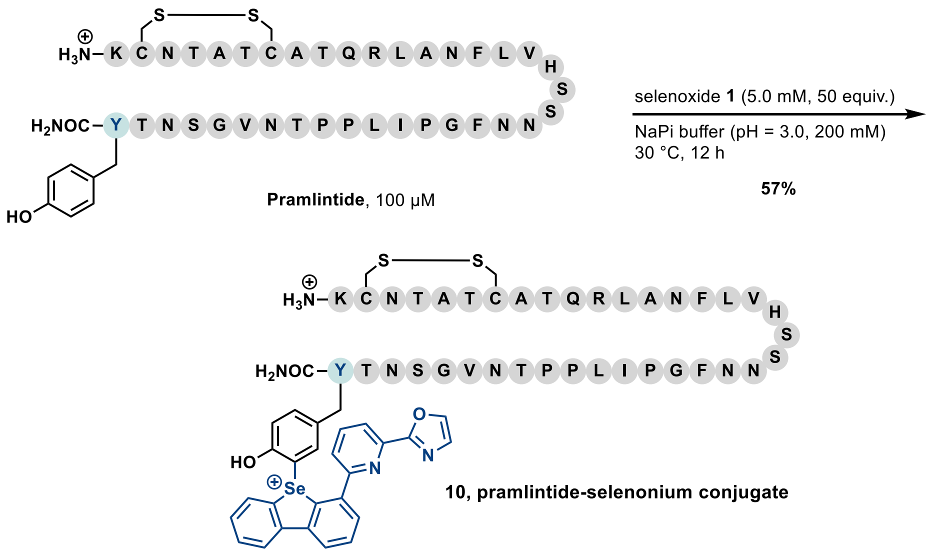

Under ambient atmosphere and at 20–25 °C, 40 µL of NaPi buffer (pH 3.0, c = 500 mM, final concentration c = 200 mM), 45 µL of UHQ-H_2O, and 10 µL of a selenoxide **1** stock solution (c = 50 mM, 0.50 µmol, 0.20 mg, 50 equiv., final concentration c = 5.0 mM) in phosphoric acid solution (c = 0.10 M) in UHQ-H_2O were added to an Eppendorf tube (1.5 mL). The mixture was vortexed for 5 sec., transferred into a Thermocycler pre-heated at 30 °C and incubated at 30 °C for 30 min at 600 rpm. Next, 5.0 µL of a pramlintide stock solution (c = 2.0 mM, 10 nmol, 39 µg, 1.0 equiv., final concentration c = 0.10 mM) in UHQ-H_2O was added. The mixture was vortexed for 5 sec., transferred into a Thermocycler pre-heated at 30 °C, and incubated at 30 °C for 12 h at 600 rpm. Subsequently, 100 µL of MeCN (final volume percentage 50%) was added and the mixture was vortexed for 5 sec., transferred into a Thermocycler pre-heated at 25 °C, and incubated at 25 °C for 20 min at 600 rpm. Subsequently, 15 µL of a freshly prepared Na_2SO_3 stock solution (c = 50 mM, 0.75 µmol, 95 µg, 75 equiv.) in UHQ-H_2O was added to reduce excess selenoxides. Again, the mixture was vortexed for 5 sec., transferred into a Thermocycler pre-heated at 25 °C, and incubated at 25 °C for 10 min at 600 rpm. The mixture was washed with ethyl acetate (100 µL × 2) to remove the selenide. The aqueous layer was gently purged by argon 2–4 mm

above the solution via a needle (Φ 0.80 × 40 mm) for 2 min to remove residual organic solvent. The solution volume was adjusted to 100 µL by the addition of UHQ-H_2O (ca. 15 µL). The resulting mixture was directly used for LC-MS analysis for yield determination as well as HRMS and LC-FTMS/MS analysis for product characterization and site-selectivity determination.

Yield was determined by LC-MS post-run PDA analysis at 328 nm to be 57% (average of three experiments).

HRMS ESI-pos (m/z) calc'd for $C_{191}H_{282}N_{53}O_{54}S_2Se^{5+}$ $[M+4H]^{5+}$, 865.1906; found, 865.1919. Deviation: –0.3 ppm.

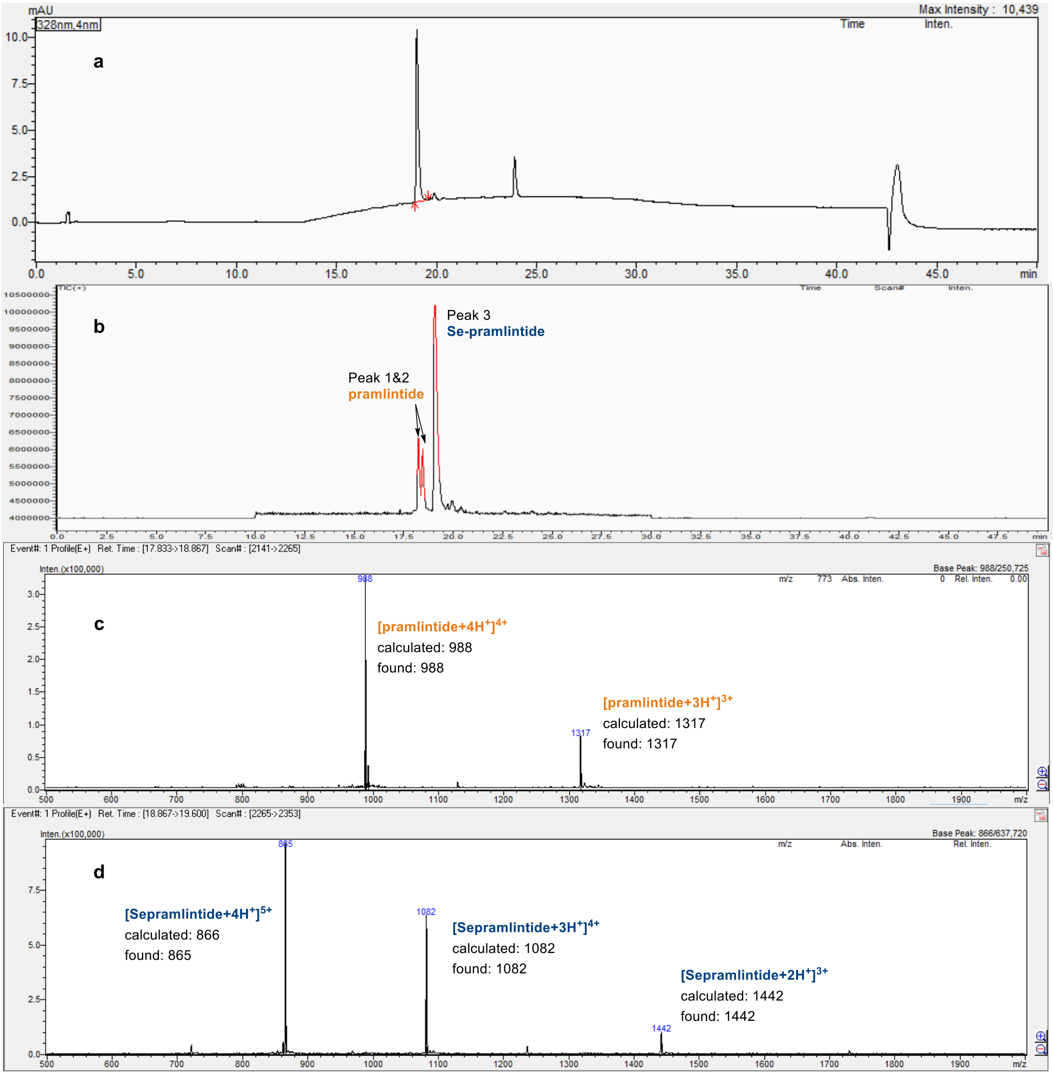

Figure 41. LC-MS analysis (Method A) of pramlintide-selenonium conjugate **10**. a) LC chromatogram (PDA 328 nm) b) Total ion chromatogram c) Peak 1 and 2 (starting material) ion series d) Peak 3 (product) ion series.

LC-FTMS/MS results

Based on LC-FTMS/MS analysis, the modification site Y37 was confirmed by the two directed ions: $(y3)^+$ ion with a possible modification on N35, T36, or Y37, and $(b36)^{4+}$ without modification from K1 to T36.

b	Sequence	y	b	Sequence	y
1	K	37	20	S	18

	**2**	C	**36**	
	**3**	N	**35**	
	**4**	T	**34**	
	**5**	A	**33**	
	**6**	T	**32**	
	**7**	C	**31**	
	**8**	A	**30**	
	**9**	T	**29**	
	**10**	Q	**28**	
	**11**	R	**27**	
	**12**	L	**26**	
	**13**	A	**25**	
	**14**	N	**24**	
	**15**	F	**23**	
	**16**	L	**22**	
**b^{2+}**:917.4586<br>**917.4576 (+1.0)**	17	V	**21**	
**b^{2+}**:985.9880<br>**985.9885 (–0.5)**	18	H	**20**	
	**19**	S	**19**	
	**21**	N	**17**	
**b^{3+}**:791.7111<br>**791.7093 (+2.3)**	22	N	**16**	
**b^{3+}**:840.7339<br>**840.7327 (+1.4)**	23	F	**15**	
**b^{4+}**:645.0576<br>**645.0572 (+0.6)**	24	G	**14**	
**b^{4+}**:669.3208<br>**669.3190 (+2.7)**	25	P	**13**	
**b^{3+}**:929.7867<br>**929.7861 (+0.6)**	26	I	**12**	
**b^{3+}**:967.4813<br>**967.4808 (+0.6)**	27	L	**11**	
**b^{4+}**:750.1260<br>**750.1263 (–0.5)**	28	P	10	**y^{2+}**:711.7545<br>**711.7546 (–0.3)**
	29	P	**9**	
**b^{3+}**:1065.8657<br>**1065.8632 (+2.4)**	30	T	**8**	
**b^{3+}**:1103.8800<br>**1103.8789 (+1.0)**	31	N	**7**	
**b^{4+}**:852.9290<br>**852.9287 (+0.4)**	32	V	**6**	
	**33**	G	5	**y^{+}**:914.2371<br>**914.2364 (+0.8)**
	**34**	S	**4**	
	**35**	N	3	**y^{+}**:770.1836<br>**770.1835 (+0.2)**
**b^{4+}**:942.7150<br>**942.7144 (+0.6)**	36	T	2	**y^{+}**:656.1407<br>**656.1403 (+0.4)**
	**37**	**Y-Se**	**1**	

Table 24. MS/MS analysis of pramlintide-selenonium conjugate **10**. Peptide concentration for analytical sample: 0.39 mg/mL. b^{n+}, peptide fragment starting from the N-terminus of the analyte with n positive charges; y^{n+}, peptide fragment starting from the C-terminus of the analyte with n positive charges; the integer, residue numbers in the corresponding fragments, integer in blue or orange indicates the corresponding b or y fragment was found, respectively; the number with 4 decimals in black, theoretical m/z numbers for the corresponding fragment; the bold numbers with 4 decimals in blue or orange, found m/z numbers for the corresponding b or y fragment, respectively; the number in bracket, the deviation of the theoretical m/z number from the found m/z number in ppm.

Preparation of insulin-selenonium conjugate 11

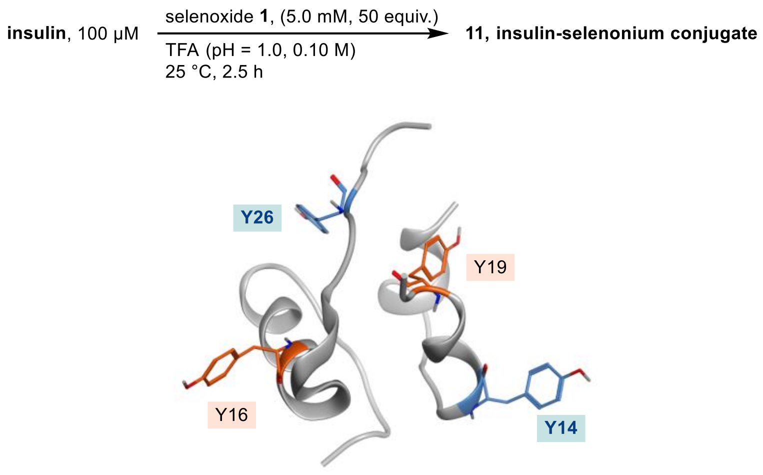

62%
mono-/double-modification = **2.6/1**
Y26/Y14 = **1.4/1**

Under ambient atmosphere and at 20–25 °C, 10 µL of a TFA stock solution (c = 1.0 M, final concentration c = 0.10 M, final pH 1.0) in UHQ-H_2O and 60 µL of UHQ-H_2O were added to an Eppendorf tube (1.5 mL). Then, 10 µL of a selenoxide **1** stock solution (c = 50 mM, 0.50 µmol, 0.20 mg, 50 equiv., final concentration c = 5.0 mM) in phosphoric acid solution (c = 100 mM) in UHQ-H_2O was added. The mixture was vortexed for 5 sec., transferred into a Thermocycler pre-heated at 25 °C, and incubated at 25 °C for 30 min at 600 rpm. Next, 20 µL of a freshly prepared insulin stock solution (c = 0.50 mM, 10 nmol, 58 µg, 1.0 equiv., final concentration c = 0.10 mM) in UHQ-H_2O was introduced to start the reaction. The mixture was vortexed for 5 sec., transferred into a Thermocycler pre-heated at 25 °C, and incubated at 25 °C for 2.5 h at 600 rpm. Subsequently, 100 µL of MeCN (final volume percentage 50%) was added and the mixture was vortexed for 5 sec., transferred into a Thermocycler pre-heated at 25 °C, and incubated at 25 °C for 10 min at 600 rpm. Subsequently, 15 µL of a freshly prepared Na_2SO_3 stock solution (c = 50 mM, 0.75 µmol, 95 µg, 75 equiv.) in UHQ-H_2O was added to reduce excess selenoxides. Again, the mixture was vortexed for 5 sec., transferred into a Thermocycler pre-heated at 25 °C, and incubated at 25 °C for 10 min at 600 rpm. The mixture was washed by ethyl acetate (100 µL × 2) to remove the selenide. The aqueous layer was gently purged by argon 2–4 mm above the solution via a needle (Φ 0.80 × 40 mm) for 90 sec. to remove residual organic solvent. The solution volume was adjusted to 100 µL by the addition of UHQ-H_2O (15 µL). The resulting mixture was used for LC-MS analysis for yield determination as well as HRMS and LC/Q-TOF-MS/MS analysis for product characterization and site-selectivity determination. The mixture was also used for the formation of iodo-insulin **26** without any further purification, and was stable at 4 °C for at least 1 week.

HRMS ESI-pos (m/z, most abundant mass)

Insulin: calc'd for $C_{257}H_{383}N_{65}O_{77}S_6$ [M], 5806.64; deconvoluted, 5806.63. Deviation: 1.7 ppm.

Insulin + Se, mono-modification: calc'd for $C_{277}H_{393}N_{67}O_{78}S_6Se$ [M], 6180.64; deconvoluted, 6180.63. Deviation: 1.6 ppm

Insulin + Se, di-modification: calc'd for $C_{297}H_{403}N_{69}O_{79}S_6Se_2$ [M], 6554.64; deconvoluted, 6554.63. Deviation: 1.5 ppm

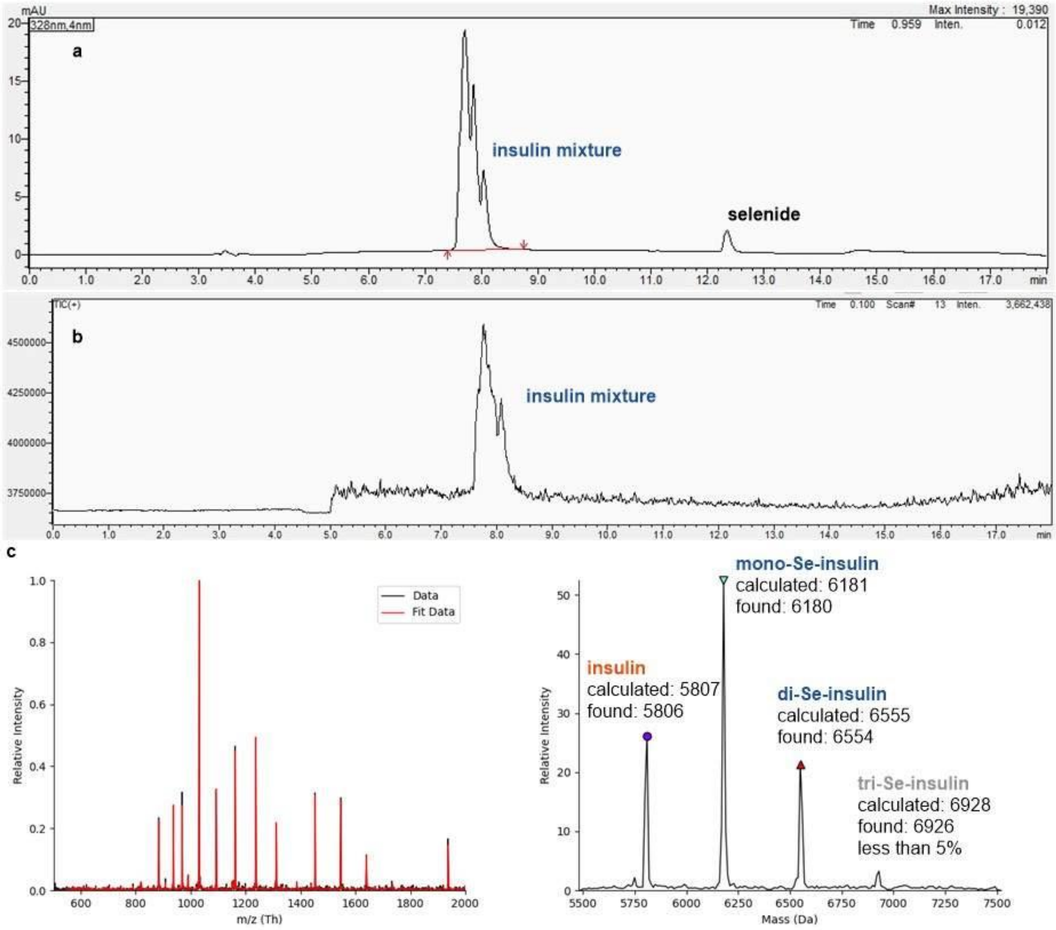

Figure 42. LC-MS analysis (Method A) of insulin-selenonium conjugate **11**. a) LC chromatogram (PDA 328 nm) b) Total ion chromatogram c) Ion series (with Unidec fitting) and deconvoluted spectrum.

Yield determination

Due to the difficulties in the separation of insulin with mono-Se modification and di-Se modifications, the yield was determined via both PDA analysis at 328 nm and peak areas obtained from the zero-charge spectra after deconvolution.

Firstly, the total yield of the selenonium chromophore in the sample was calculated as previous (LC injection volume was 5 μL):

$$\text{total yield} = \frac{\text{peak area/LC injection volume (μL)} \times \text{sample volume (μL)}}{\text{the slope of the standard curve } (\text{mM}^{-1})/5\ (\text{μL}) \times \text{total insulin amount (nmol)}} \times 100\%$$

Then, based on the percentage of the product peak area in the total peak area of all insulins in zero-charge spectra, the yield of unmodified insulin, insulin with mono-Se modification, and insulin with di-Se modifications can be calculated:

$$\text{insulin recovery} = \frac{\text{total yield} \times \text{pct. of unmodified insulin}}{\text{pct. of monomodification} + 2 \times \text{pct. of dimodification}}$$

$$\text{mono-Se insulin yield} = \frac{\text{total yield} \times \text{pct. of mono-Se insulin}}{\text{pct. of monomodification} + 2 \times \text{pct. of dimodification}}$$

$$\text{di-Se insulin yield} = \frac{\text{total yield} \times \text{pct. of di-Se insulin}}{\text{pct. of monomodification} + 2 \times \text{pct. of dimodification}}$$

(**Note:** tri-*Se*-modified insulin was not involved in the calculation because its area percentage in zero-charge spectrum is lower than the detection threshold of UniDec.)

Finally, the total recovery of protein can be calculated:

$$\text{total recovery} = \text{insulin recovery} + \text{mono-Se insulin yield} + \text{di-Se insulin yield}$$

In this reaction, the yield of mono-Se insulin and di-Se insulin was 45% and 17%, respectively (ratio 2.6/1). Combined with the recovery of unmodified insulin (23%), the total recovery of the protein was 85% (each yield was an average of three experiments).

Site-selectivity determination

Sample preparation for LC/Q-TOF-MS/MS analysis

Under ambient atmosphere and at 20–25 °C, 12.5 µL of the insulin-selenonium conjugate **11** product mixture (for total protein: c = 85 µM, 1.1 nmol, 6.2 µg (weight of modification group not included), 1.0 equiv.) in the aqueous solution of TFA (c = 0.10 M), 16 µL of UHQ-H_2O, and 7.5 µL of NH_4HCO_3 buffer (pH 9.0, c = 500 mM, final concentration c = 100 mM) were added to an Eppendorf tube (1.5 mL). The mixture was vortexed for 5 sec. and 1.5 µL of the trypsin (0.2 mg/mL, 0.3 µg) was added to fragment the protein (blue dashed line in Fig. 43). Then the mixture was transferred into a Thermocycler pre-heated at 37 °C and incubated at 37 °C for 18 h at 600 rpm. Next, 2.0 µL of a DTT stock solution (c = 0.20 M, 0.40 µmol, 62 µg, 3.6 × 10^2 equiv.) in UHQ-H_2O was added to reduce all disulfide bonds (black dashed line in Fig. 43). The mixture was vortexed for 5 sec., transferred into a Thermocycler pre-heated at 37 °C, and incubated at 37 °C for an additional 30 min at 600 rpm. The resulting mixture was analyzed by LC/Q-TOF-MS/MS to determine the site of modification.

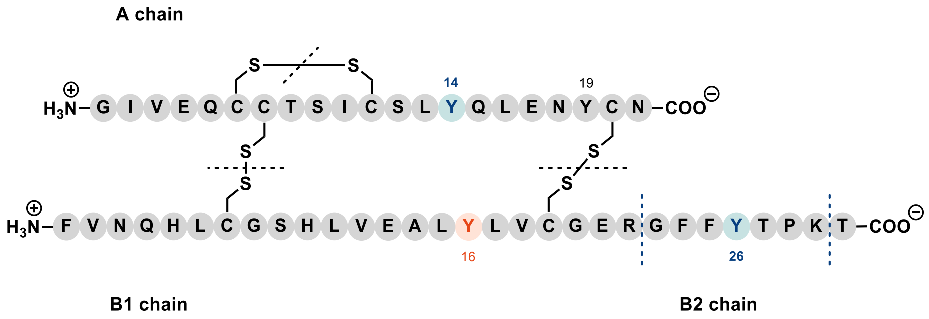

Figure 43. Fragmentation of insulin-selenonium conjugate **11**. Black dashed lines, disulfide bond broke by DTT reduction; blue dashed line, peptide bonds cleaved by trypsin; blue Y, modified tyrosine; orange Y, modification occurs at trace amount (<2%).

The LC/Q-TOF-MS/MS results suggest significant single modification only on A chain and B2 chain (Fig. 44a, b). The modification sites were located at Y14 of A chain and Y4 of B2 chain (Y26 of B chain) confirmed by MS/MS analysis (Tables 25–27). Because the Se is a chiral center, the modified products A-Se and B2-Se chains both contain a pair of epimers, which explains the doublet peaks of the modified peptide chains in the PDA analysis at 214 nm and 328 nm (Fig. 44a). Given that each modified fragment contained only one modification, the ratio of modification on different tyrosine residues was determined based on the post-run PDA analysis at 328 nm, by dividing the integral value for the B2-Se peak by the value for A-Se peak. The ratio of B2-Se (Y4) to A-Se (Y14) was 1.4/1.

The side reaction on B1 chain was also detected by mass spectrometry, which could explain the

observation of a small amount of triple *Se*-modified product in the functionalization of insulin (Fig. 42c). However, the amount of *Se*-modifed B1 was very small and even cannot be detected in the post-run PDA analysis at 328 nm (integral area was less than 5% of the whole integration area).

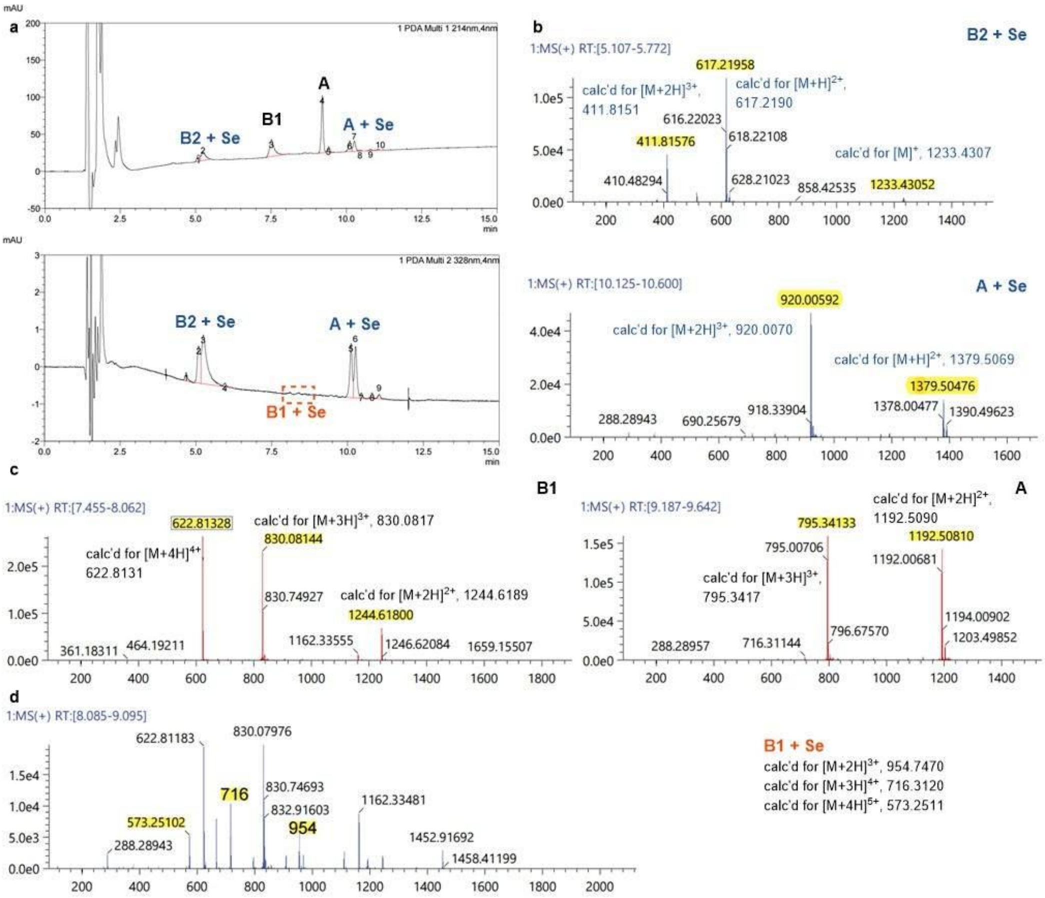

Figure 44. LC/Q-TOF-MS analysis (Method D) of digested insulin-selenonium conjugate **11** product mixture. a) LC chromatogram (PDA 214 nm and 328 nm), doublet peaks of A-Se and B2-Se chains indicated a pair of epimers of A-Se and B2-Se chains, respectiviely. b) Ion series of *Se*-modified B2 and *Se*-modified-A chain c) Ion series of unmodified B1 and A chain d) Ion series of modified B1 chain (mixed with unmodified B1 chain). Total peptide concentration for analytical sample: 0.16 mg/mL.

LC/Q-TOF-MS/MS result of B2-Se chain

b^+	b^{2+}		Sequence		y^+	y^{2+}
58.0288	29.5181	**1**	G	**7**	1233.4307	617.2190<br>**617.2195 (–0.8)**
205.0972<br>**205.0969 (1.5)**	103.0523	**2**	F	**6**	1176.4093	588.7083<br>**588.7099 (–2.7)**
352.1656<br>**352.1652 (1.1)**	176.5865	**3**	F	**5**	1029.3408	515.1741<br>**515.1744 (–0.6)**
889.2247	445.1160<br>**445.1163 (–0.7)**	**4**	**Y + Se**	**4**	882.2724	441.6399<br>**441.6402 (–0.7)**
990.2724	495.6399<br>**495.6408 (–1.8)**	**5**	T	**3**	345.2133<br>**345.2131 (0.6)**	173.1103
1087.3252	544.1663<br>**544.1665 (0.4)**	**6**	P	**2**	244.1656<br>**244.1654 (0.8)**	122.5865
1215.4201	608.2137<br>**608.2142 (–0.8)**	**7**	K	**1**	147.1129<br>**147.1126 (2.0)**	74.0601

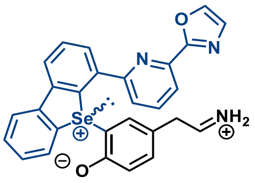

Immonium ion

m/z calculated: 510.0715

m/z found: 510.0713

Table 25. LC/Q-TOF-MS/MS analysis of B2-Se peptide. Total peptide concentration for analytical sample: 0.16 mg/mL. b^{n+}, peptide fragment starting from the N-terminus of the analyte with n positive charges; y^{n+}, peptide fragment starting from the C-terminus of the analyte with n positive charges; the integer, residue numbers in the corresponding fragments, integer in blue or orange indicates the corresponding b or y fragment was found, respectively; the number with 4 decimals in black, theoretical m/z numbers for the corresponding fragment; the bold numbers with 4 decimals in blue or orange, found m/z numbers for the corresponding b or y fragment, respectively; the number in bracket, the deviation of the theoretical m/z number from the found m/z number in ppm.

LC/Q-TOF-MS/MS result of A-Se chain

Se modification on Y14 (matched with MS/MS data):

b^+	b^{2+}	b^{3+}		Sequence		y^+	y^{2+}
58.0288	29.5181	20.0145	**1**	G	**21**	2757.0032	1379.0053
171.1129<br>**171.1128 (0.6)**	86.0601	57.7092	**2**	I	**20**	2699.9817	1350.4945
270.1813<br>**270.1812 (0.4)**	135.5943	90.7320	**3**	V	**19**	2586.8977	1293.9525
399.2239<br>**399.2235 (1.0)**	200.1156	133.7462	**4**	E	**18**	2487.8292	1244.4183<br>**1244.4156 (2.2)**
527.2824<br>**527.2817 (1.3)**	264.1449	176.4324	**5**	Q	**17**	2358.7867	1179.8970<br>**1179.8991 (–1.8)**
630.2916<br>**630.2901 (2.4)**	315.6495	210.7688	**6**	C	**16**	2230.7281	1115.8677<br>**1115.8654 (2.1)**
733.3008<br>**733.2995 (1.8)**	367.1541	245.1052	**7**	C	**15**	2127.7189	1064.3631<br>**1064.3683 (–4.9)**
834.3485<br>**834.3477 (1.0)**	417.6779	278.7877	**8**	T	**14**	2024.7097	1012.8585<br>**1012.8621 (–3.6)**
921.3805<br>**921.3763 (4.6)**	461.1939	307.7984	**9**	S	**13**	1923.6620	962.3347<br>**962.3323 (2.5)**
1034.4646<br>**1034.4630 (1.5)**	517.7360	345.4931	**10**	I	**12**	1836.6300	918.8187<br>**918.8184 (0.3)**
1137.4738<br>**1137.4746 (–0.7)**	569.2405	379.8295	**11**	C	**11**	1723.5459	862.2766<br>**862.2777 (–1.3)**
1224.5058	612.7566	408.8401	**12**	S	**10**	1620.5368	810.7720<br>**810.7716 (0.5)**
1319.5792[a]<br>**1319.5823 (–2.3)**	660.2933[a]	440.5313[a]	**13**[a]	L	**9**	1533.5047	767.2560
1874.6490	937.8282<br>**937.8267 (1.6)**	625.5546	**14**	**Y + Se**	**8**	1420.4207	710.7140
2002.7076	1001.8575<br>**1001.8576 (–0.1)**	668.2408	**15**	Q	**7**	883.3615<br>**883.3604 (1.2)**	442.1844
2115.7917	1058.3995<br>**1058.3983 (1.1)**	705.9354	**16**	L	**6**	755.3029<br>**755.3019 (1.3)**	378.1551
2244.8343	1122.9208<br>**1122.9218 (–0.9)**	748.9496	**17**	E	**5**	642.2189<br>**642.2186 (0.5)**	321.6131
2358.8772	1179.9423<br>**1179.9401 (1.9)**	786.9639	**18**	N	**4**	513.1763<br>**513.1772 (–1.8)**	257.0918

2521.9405	1261.4739<br>**1261.4737 (0.2)**	841.3184<br>**841.3201 (–2.0)**	**19**	Y	**3**	399.1333<br>**399.1331 (0.5)**	200.0703
2624.9497	1312.9785<br>**1312.9797 (0.9)**	875.6548<br>**875.6551 (–0.3)**	**20**	C	**2**	236.0700<br>**236.0698 (0.8)**	118.5387
2738.9926	1370.0000	913.6691<br>**913.6638 (5.8)**	**21**	N	**1**	133.0608<br>**133.0608 (0)**	67.0341

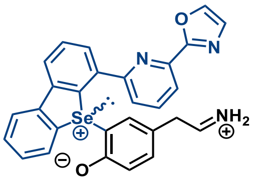

Immonium ion

m/z calculated: 510.0715

m/z found: 510.0711

Table 26. LC/Q-TOF-MS/MS analysis of A-Se peptide. Table presents the MS signals calculated based on Y14 modification. Total peptide concentration for analytical sample: 0.16 mg/mL. b^{n+}, peptide fragment starting from the N-terminus of the analyte with n positive charges; y^{n+}, peptide fragment starting from the C-terminus of the analyte with n positive charges; the integer, residue numbers in the corresponding fragments, integer in blue or orange indicates the corresponding b or y fragment was found, respectively; the number with 4 decimals in black, theoretical m/z numbers for the corresponding fragment; the bold numbers with 4 decimals in blue or orange, found m/z numbers for the corresponding b or y fragment, respectively; the number in bracket, the deviation of the theoretical m/z number from the found m/z number in ppm. a) $[b13–H_2O]^+$ observed.

Se modification on Y19 (not supported by experimental data):

b^+	b^{2+}	b^{3+}		Sequence		y^+	y^{2+}
1500.65318	750.83025	500.88927	**14**	Y			
1628.71176	814.85954	543.57547	**15**	Q	**7**	1257.35733	629.18232
1741.79582	871.40157	581.27015	**16**	L	**6**	1129.29875	565.15304
1870.83841	935.92287	624.28435	**17**	E	**5**	1016.21469	508.61100
1984.88134	992.94433	662.29866	**18**	N	**4**	887.17209	444.08971
				**Y + Se**	**3**	773.12917	387.06824

Table 27. LC/QTOF-MS/MS analysis of A-Se peptide. Table presents the different MS/MS signals of Y19 modification compared to those of Y14 modification. b^{n+}, peptide fragment starting from the N-terminus of the analyte with n positive charges; y^{n+}, peptide fragment starting from the C-terminus of the analyte with n positive charges; the integer, residue numbers in the corresponding fragments, integer in blue or orange indicates the corresponding b or y fragment was found, respectively; the number with 4 decimals in black, theoretical m/z numbers for the corresponding fragment; the bold numbers with 4 decimals in blue or orange, found m/z numbers for the corresponding b or y fragment, respectively; the number in bracket, the deviation of the theoretical m/z number from the found m/z number in ppm.

Analysis of the site-selectivity of the *Se*-modification of insulin

Among all tyrosine residues in insulin, the Y14 on A chain is completely exposed to solvent while the Y19 on A chain is half-buried by neighbouring residues, which could explain the reactivity of Y14 and the inertness of Y19 toward **1** under the reaction conditions (Fig. 45). However, the site selectivity of reagent **1** cannot be predicted simply from the crystal structure, because the structure of protein in solution could differ from that in the solid state. Although the Y26 on B chain is more shielded than the Y16 on B chain according to the crystal structure (Fig. 46), the Y26 is the other major modification of **1** due to the strong solvent exposure of Y26 in acidic aqueous solution (pH < 3), which is suggested by the faster proton-deuterium exchange of the Y26 peptidyl group compared to that of the Y19 (A chain) peptidyl group and that of the Y16 (B chain) peptidyl group in both simulation[19] and NMR experiments[20]. In acidic solution, insulin could exsit as monomer and the C-terminal part of B chain (B20–30) can be more flexible and detach from the hydrophobic core[19], which leads to the larger solvent exposure of Y26 in the acidic

solution compared to that in crystal structure, thus further explaining the reactivity of Y26 toward **1** under the reaction conditions.

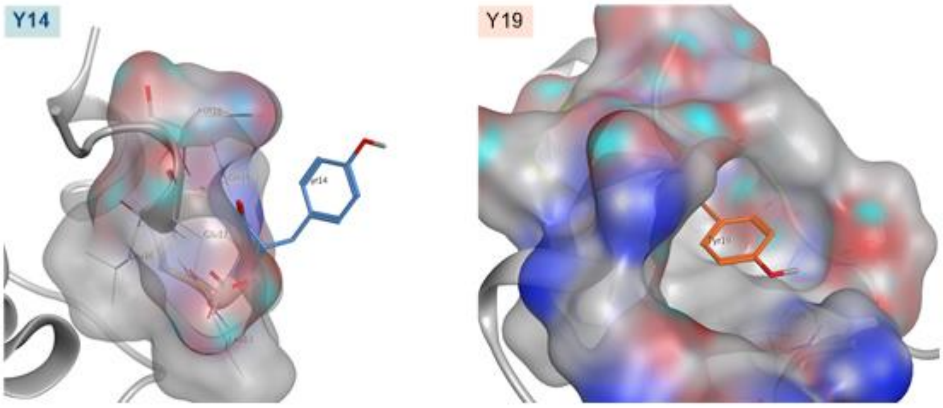

Figure 45. Surface of surrounding residues (distance in 4.5 Å) of Y14 and Y19 on A chain. Blue part of surface, nitrogen atom; red part of surface, oxygen atom. Water molecules are omitted for clarity. PDB ID of the insulin structure: 3I40.

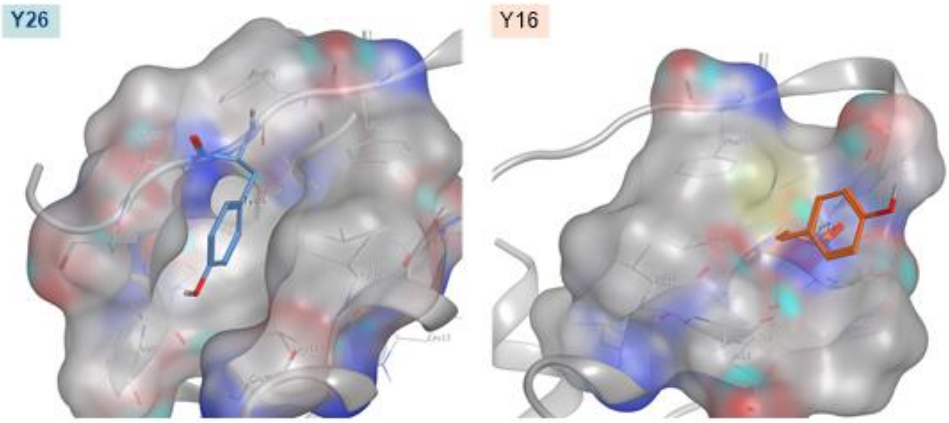

Figure 46. Surface of surrounding residues (distance in 4.5 Å) of Y16 and Y26 on B chain. Blue part of surface, nitrogen atom; red part of surface, oxygen atom. Water molecules are omitted for clarity. PDB ID of the insulin structure: 3I40.

Preparation of ubiquitin-selenonium conjugate 12

selenoxide **1** (5.0 mM, 100 equiv.)
$MgCl_2$ (50 mM)
urea (2.4 M)
ubiquitin, 50 µM ⟶ **12, ubiquitin-selenonium conjugate**
NaPi buffer (pH = 3.0, 50 mM)
37 °C, 12 h

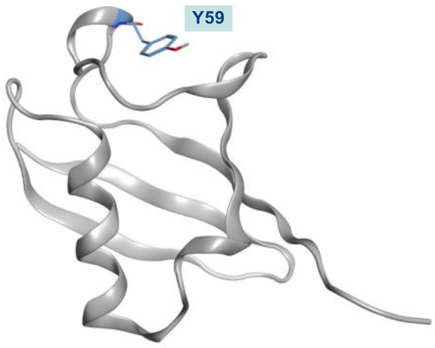

56%, **Y59**

Under ambient atmosphere and at 20–25 °C, 10 µL of NaPi buffer (pH 3.0, c = 500 mM, final concentration c = 50 mM), 2.5 µL of an $MgCl_2$ stock solution (c = 2.0 M, 5.0 µmol, 0.48 mg, 1.0 × 10^3 equiv., final concentration c = 0.10 M) in UHQ-H_2O, 30 µL of a freshly prepared urea stock solution (c = 8.0 M, 0.24 mmol, 14 mg, 4.8 × 10^4 equiv., final concentration c = 2.4 M, pH adjusted to 3.0 by TFA) in UHQ-H_2O, and 37.5 µL of UHQ-H_2O were added to an Eppendorf tube (1.5 mL). (*Note:* The urea stock solution must be prepared freshly because urea can slowly hydrolyze and release ammonia under acidic conditions, and the ammonia is detrimental to the subsequent bromination by complexation with Cu^{2+}.) Then, 10 µL of a selenoxide **1** stock solution (c = 50 mM, 0.50 µmol, 0.20 mg, 1.0 × 10^2 equiv., final concentration c = 5.0 mM) in phosphoric acid solution (c = 100 mM) in UHQ-H_2O was added. The mixture was vortexed for 5 sec., transferred into a Thermocycler pre-heated at 37 °C and incubated at 37 °C for 30 min at 600 rpm. Next, 10 µL of an ubiquitin stock solution (c = 0.50 mM, 5.0 nmol, 43 µg, 1.0 equiv., final concentration c = 50 µM) in UHQ-H_2O was introduced to start the reaction. The mixture was vortexed for 5 sec., transferred into a Thermocycler pre-heated at 37 °C, and incubated at 37 °C for 12 h at 600 rpm. Subsequently, 100 µL of MeCN (final volume percentage 50%) was added and the mixture was vortexed for 5 sec., transferred into a Thermocycler pre-heated at 25 °C, and incubated at 25 °C for 10 min at 600 rpm. Subsequently, 15 µL of a freshly prepared Na_2SO_3 stock solution (c = 50 mM, 0.75 µmol, 95 µg, 75 equiv.) in UHQ-H_2O was added to reduce excess selenoxides. Again, the mixture was vortexed for 5 sec., transferred into a Thermocycler pre-heated at 25 °C, and incubated at 25 °C for 10 min at 600 rpm. The mixture was washed by ethyl acetate (200 µL × 2) to remove the selenide. The aqueous layer was gently purged by argon 2–4 mm above the solution via a needle (Φ 0.80 × 40 mm) for 90 sec. to remove residual organic solvent. The solution volume was adjusted to 100 µL by the addition of UHQ-H_2O (ca. 20 µL). The resulting mixture was directly used for LC-MS analysis for yield and recovery determination, for LC/Q-TOF-MS and LC-FTMS/MS analysis for product characterization, and for the subsequent bromination without any further purification, and can be stable at 4 °C for at least 1 week. Yield of the product was determined by LC-MS post-run PDA analysis at 328 nm to be 56% and recovery of the ubiquitin was determined by PDA analysis at 214 nm to be 28% (in triplicate) using the equation below (LC injection volume was 5 µL):

$$\text{recovery} = \frac{\text{ubiquitin peak area/LC injection volume } (\mu\text{L}) \times \text{sample volume } (\mu\text{L})}{\text{the slope of ubiquitin std. curve } (\text{mM}^{-1})/5\ (\mu\text{L}) \times \text{ubiquitin used in reaction (nmol)}} \times 100\%$$

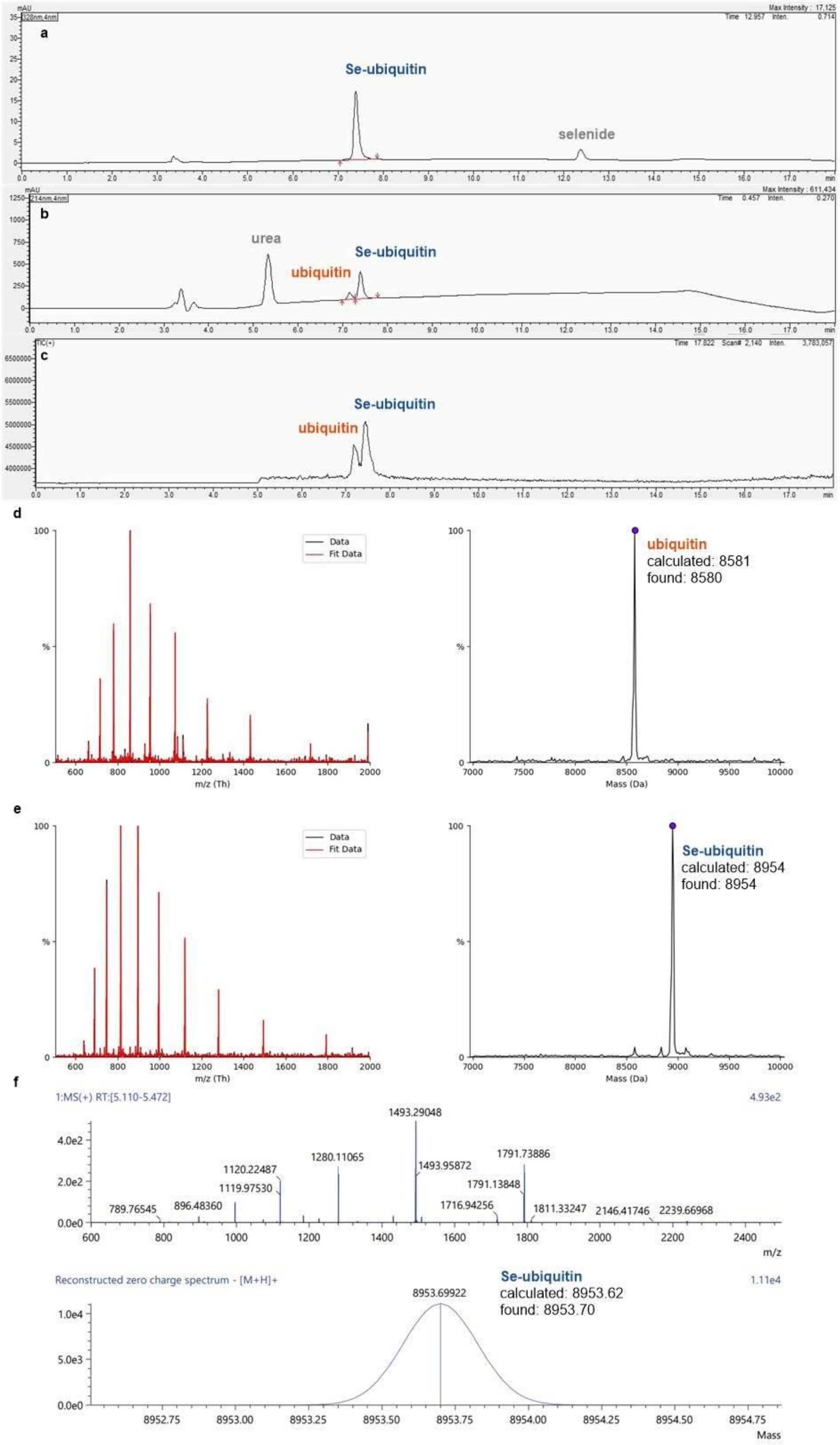

Figure 47. LC-MS analysis (Method A) and LC/Q-TOF-MS (Method C) of ubiquitin-selenonium conjugate **10** for yield determination. a) LC chromatogram (PDA 328 nm) b) LC chromatogram (PDA 214 nm) c) Total ion chromatogram d) Peak 1 (starting material) ion series and deconvoluted spectrum e) Peak 2 (product) ion series and deconvoluted spectrum f) Product peak ion series and deconvoluted spectrum obtained by LC/Q-TOF-MS. All ubiquitins and derivatives contain oxidized methionine residues.

Oxidation of methionines

Although only methionine-oxidized ubiquitin was observed upon the *Se*-modification, considering that most methionine residues in the ubiquitin substrate were already oxidized before the reaction (Fig. 36), the oxidation of the methionine residues upon *Se*-modification should be of small extent. Combined with the result that methionine remains inert to the selenoxide **1** under the standard conditions (pH 3.0, 30 °C, Fig. 30), the side reactivity toward methionine residue here could result from the elevated reaction temperature (37 °C).

LC-FTMS/MS sample preparation

Under ambient atmosphere and at 20–25 °C, 43 µL of the ubiquitin-selenonium conjugate **12** reaction mixture (c = 28 µM, 1.2 nmol, 11 µg; for total protein, c = 42 µM, 1.8 nmol, 16 µg) in NaPi buffer (pH = 3.0, c = 50 mM, final concentration c = 22 mM) containing $MgCl_2$ (c = 50 mM, final concentration c = 22 mM) and urea (c = 2.4 M, final concentration c = 1.0 M), 2.0 µL of NaOH (c = 2.0 M, to adjust the solution pH to 6–7), and 5.0 µL of SDS stock solution (10 wt.%, final conc. 1.0 wt.%) were added to an Eppendorf tube (1.5 mL). The mixture was vortexed for 5 sec., transferred into a Thermocycler pre-heated at 37 °C and incubated at 37 °C for 15 min at 600 rpm. Then, 46 µL of UHQ-H_2O and 4 µL of SP3 magneitc beads were added and the subsequent operations were following the established standard SP3 protocol[21]. No reductant or alkylating reagent was used because there is no cysteine residue in ubiquitin.

MS/MS results

b^+		Sequence		y^+
102.0550	**1**	T	**9**	1455.5483
215.1391<br>**215.1390 (0.5)**	**2**	L	**8**	1354.5006
302.1711<br>**302.1707 (1.3)**	**3**	S	**7**	1241.4166<br>**1241.4155 (0.9)**
417.1980<br>**417.1976 (1.0)**	**4**	D	**6**	1154.3845<br>**1154.3887 (–3.6)**
954.2572	**5**	**Y + Se**	**5**	1039.3576
1068.3001<br>**1068.3041 (–3.7)**	**6**	N	**4**	502.2984<br>**502.2981 (0.6)**
1181.3842	**7**	I	**3**	388.2555<br>**388.2556 (–0.3)**
1309.4428	**8**	Q	**2**	275.1714<br>**275.1712 (0.7)**
1437.5377	**9**	K	**1**	147.11285

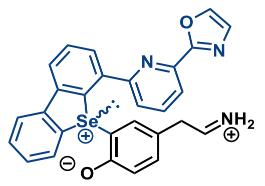

Immonium ion

m/z calculated: 510.0715

m/z found: 510.0515

Table 28. MS/MS analysis of peptide fragment TLSDYNIQK of ubiquitin-selenonium conjugate **10** after digestion. Total peptide concentration for analytical sample: 0.16 mg/mL. b^{n+}, peptide fragment starting from the N-terminus of the analyte with n positive charges; y^{n+}, peptide fragment starting from the C-terminus of the analyte with n positive charges; the integer, residue numbers in the corresponding fragments, integer in blue or orange indicates the corresponding b or y fragment was found, respectively; the number with 4 decimals in black, theoretical m/z numbers for the corresponding fragment; the bold numbers with 4 decimals in blue or orange, found m/z numbers for the corresponding b or y fragment, respectively; the number in bracket, the deviation of the theoretical m/z number from the found m/z number in ppm.

Microenvironment of Y59

The Y59 in ubiquitin is half buried and mainly surrounded by the salt bridge between Arg54 and Asp58, and the alkyl side chain of Lys48. The steric hindrance of the surrounding residues prevents the selenoxide **1** from accessing Y59, resulting in the inertness of the protein substrate toward **1** under the reaction conditions without urea.

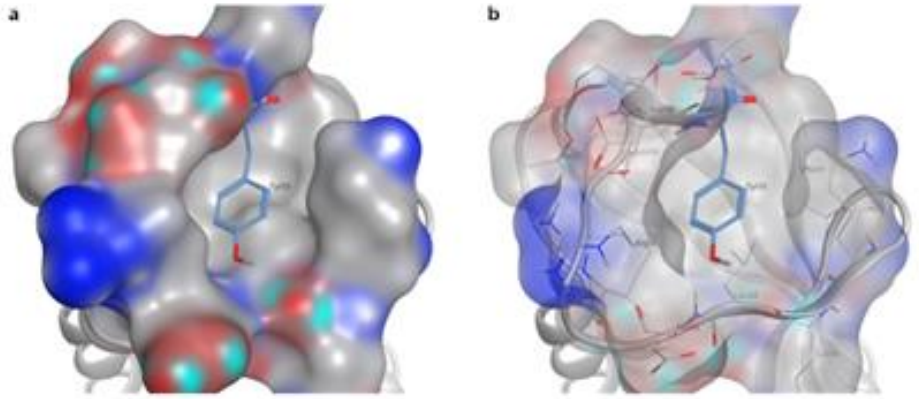

Figure 48. Surface of surrounding residues (distance in 4.5 Å) of Y59. Blue part of surface, nitrogen atom; red part of surface, oxygen atom. Water molecules are omitted for clarity. PDB ID of the ubiquitin structure: 1UBQ. a) Nontransparent surface, b) Transparent surface and residues in the proximity of Y59.

Preparation of ribonuclease A-selenonium conjugate 13

ribonuclease A, 50 µM —[selenoxide **1**, (5.0 mM, 100 equiv.), Na_2SO_4 (200 mM); NaPi buffer (pH = 3.0, 200 mM), 37 °C, 16 h]→ **13, ribonuclease A-selenonium conjugate**

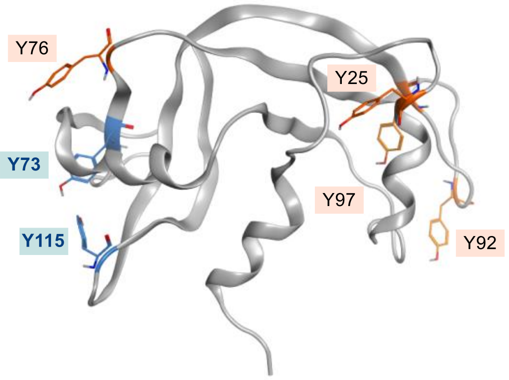

60%
mono-/double-modification = **11/1**

Under ambient atmosphere and at 20–25 °C, 40 µL of NaPi buffer (pH 3.0, c = 500 mM, final concentration c = 50 mM), 10 µL of an Na_2SO_4 stock solution (c = 2.0 M, 20 µmol, 2.8 mg, 4.0 × 10^3 equiv., final concentration c = 0.20 M) in UHQ-H_2O, and 20 µL of UHQ-H_2O were added to an Eppendorf tube (1.5 mL). Then, 10 µL of a selenoxide **1** stock solution (c = 50 mM, 0.50 µmol, 0.20 mg, 1.0 × 10^2 equiv., final concentration c = 5.0 mM) in phosphoric acid solution (c = 100 mM) in UHQ-H_2O was added. The mixture was vortexed for 5 sec., transferred into a Thermocycler pre-heated at 37 °C and incubated at 37 °C for 30 min at 600 rpm. Next, 20 µL of a ribonuclease A stock solution (c = 0.25 mM, 5.0 nmol, 68 µg, 1.0 equiv., final concentration c = 50 µM) in UHQ-H_2O was introduced to start the reaction. The mixture was vortexed for 5 sec., transferred into a Thermocycler pre-heated at 37 °C, and incubated at 37 °C for 18 h at 600 rpm. Subsequently, the mixture was added to 300 µL of HEPES buffer (pH 8.0, c = 100 mM) dropwise in an Eppendorf tube (1.5 mL) at 20–25 °C. The resulting solution was transferred to Amicon® Ultra Centrifugal Filters (10 kDa cutoff, 0.5 mL) and centrifuged at 14,000 ×g and at 4 °C for 15 min. Subsequently, 300 µL of HEPES buffer (pH 8.0, c = 100 mM) was added and the mixture was centrifuged at 14,000 ×g and at 4 °C for 15 min again, and this step was repeated for 3 times to remove excess amount of the selenoxide. The volume of the resulting mixture was adjusted to 100 µL by ca. 50 µL of HEPES buffer (pH 8.0, c = 100 mM). The mixture was used for LC-MS analysis and for the sample preparation of LC-FTMS/MS analysis.

HRMS ESI-pos (m/z, averaged mass)

Due to low abundance of the monoisotopic signal, HRMS is performed on the most intense signal of the isotopic distribution. The theoretical value is calculated from the simulated isotopic pattern at instrument mass resolution.

Ribonuclease A: calc'd for $C_{567}{}^{13}C_8H_{910}N_{171}O_{193}S_{12}{}^{9+}$ $[M+9H]^{9+}$, 1521.15(0); found, 1521.15(1). Deviation: –0.7 ppm.

Ribonuclease A + Se, mono-modification:

Calc'd for $C_{588}{}^{13}C_7H_{921}N_{173}O_{194}S_{12}Se^{10+}$ $[M + 7 +9H]^{10+}$, 1406.43(4); found, 1406.43(5). Deviation: –0.7

ppm.

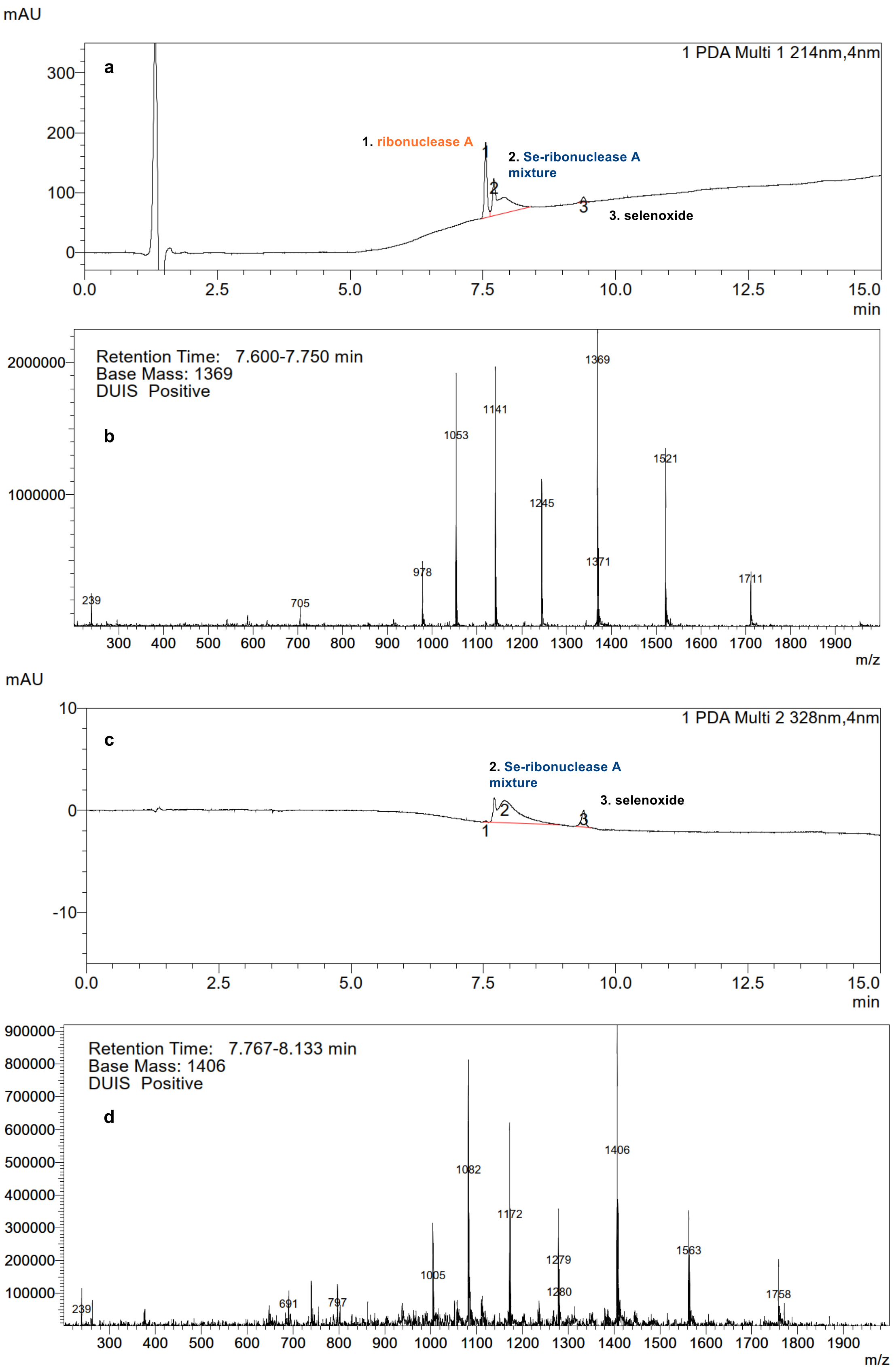
mAU
1 PDA Multi 1 214nm,4nm
a
1. ribonuclease A
2. Se-ribonuclease A mixture
3. selenoxide
min
Retention Time: 7.600-7.750 min
Base Mass: 1369
DUIS Positive
b
m/z
mAU
1 PDA Multi 2 328nm,4nm
c
2. Se-ribonuclease A mixture
3. selenoxide
min
Retention Time: 7.767-8.133 min
Base Mass: 1406
DUIS Positive
d
m/z

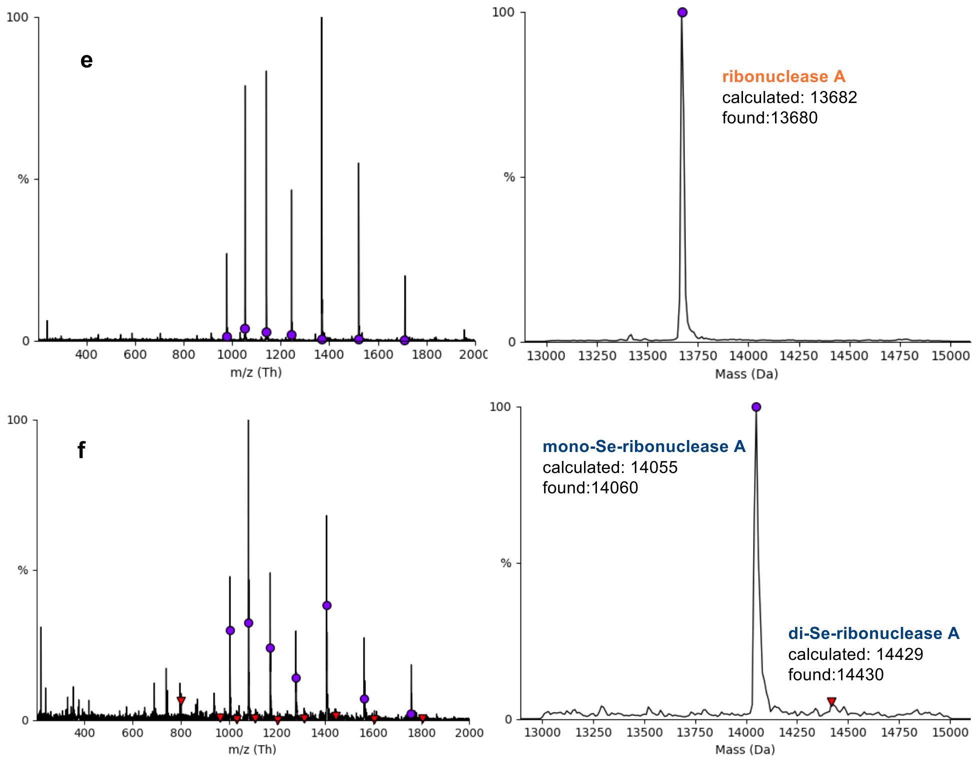

Figure 49. LC-MS analysis (Method A) of ribonuclease A-selenonium conjugate **13**. a) LC chromatogram (PDA 214 nm), b) MS spectrum of peak 1 (ribonuclease A), c) LC chromatogram (PDA 328 nm), d) MS spectrum of peak 2 (*Se*-ribonuclease A mixture), e) Ion series (with Unidec fitting) and deconvoluted spectrum of peak 1 (ribonuclease A) f) Ion series (with Unidec fitting) and deconvoluted spectrum of peak 2 (*Se*-ribonuclease A mixture). The deconvoluted MS numbers correspond to zero-charge mass (averaged).

Yield determination

The yield was determined in the same way as insulin. The yield of mono-Se ribonuclease A and di-Se ribonuclease A was 55% and 5%, respectively (ratio 11/1). Combined with the recovery of unmodified insulin (22%), the total recovery of the protein was 82% (each yield was an average of three experiments).

Site-selectivity determination

Sample preparation for LC-FTMS/MS analysis

The sample was prepared following the literature[21], using DTT as a reductant, 2-iodoacetamide as an alkylating reagent, and trypsin as a digestive enzyme. Under ambient atmosphere and at 20–25 °C, 80 µL of the ribonuclease A-selenonium conjugate **13** product mixture (for total protein: c = 41 µM, 3.3 nmol, 45 µg (weight of modification group not included), 1.0 equiv.) in HEPES buffer (pH 8.0, c = 100 mM), 75 µL of HEPES buffer (pH 8.0, c = 100 mM), 20 µL of SDS (10 wt%) in UHQ-H_2O, and 5 µL of a freshly prepared DTT solution (c = 0.20 M, 1.0 µmol, 1.5× 10^2 µg, 2.6 × 10^2 equiv.) in UHQ-H_2O were added to an Eppendorf tube (1.5 mL). The mixture was vortexed for 5 sec. and transferred into a Thermocycler pre-heated at 60 °C and incubated at 60 °C for 30 min at 1000 rpm. Next, 20 µL of a freshly prepared 2-iodoacetamide solution (c = 0.20 M, 1.0 µmol, 1.9× 10^2 µg, 2.6 × 10^2 equiv.) in HEPES buffer (pH 8.0, c = 100 mM) was added. The mixture was vortexed for 5 sec., transferred into a Thermocycler pre-heated at 25 °C, and incubated at 25 °C for 30 min at 1000 rpm. To the incubation, 7.0 µL of a SP3 beads solution (50 µg/µL, 3.6× 10^2 µg, protein:beads=1:8 (wt./wt.)) was added and vortexed for 5s. Subsequestly, 207

μL of absolute EtOH (final volume percentage 50%) was added and the mixture was transferred into a Thermocycler pre-heated at 25 °C and incubated at 25 °C for 5 min at 1000 rpm. The tube was put in a magnetic stand and the supernatant was removed. The tube containing beads was taken out from the magnetic stand. To the tube, 400 μL of 80% EtOH was added and pipetted to homogenize the solution. The tube was replaced on the magnetic stand and the supernatant was removed. After this washing step was repeated three times, 97 μL of NH_4HCO_3 buffer (pH 9.0, c = 100 mM) and 3.3 uL trypsin (0.2 mg/mL, 0.66 μg, protein: trypsin=68:1 (wt./wt.)) was added to fragment the protein (blue dashed line in Fig. 50). Then the mixture was sonicated for 30 sec. and transferred into a Thermocycler pre-heated at 37 °C and incubated at 37 °C for 18 h at 1000 rpm. After the digestion, the mixture was centrifuged at 15,000 ×g and at 25 °C for 5 min, and the tube was placed on the magnetic stand. The supernatant was taken and analyzed by MS/MS to determine the site of modification.

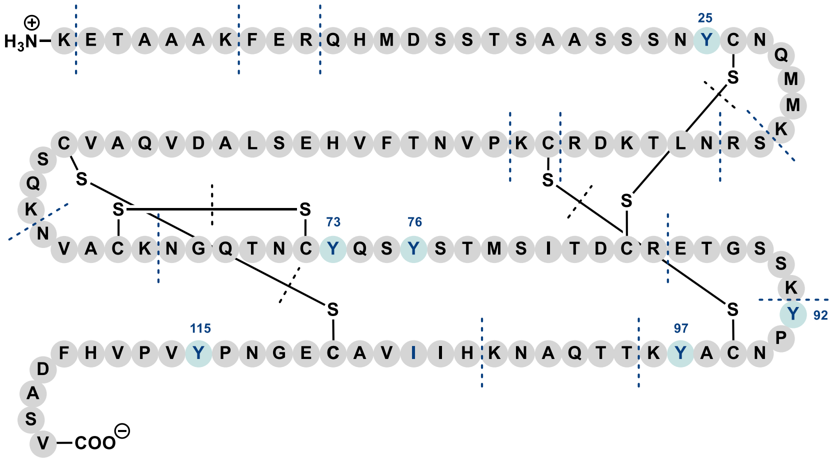

Figure 50. Fragmentation of ribonuclease A-selenonium conjugate **13**. Black dashed lines, disulfide bond broke by DTT reduction; blue dashed line, peptide bonds cleaved by trypsin.

MS/MS results

Based on the results of MS/MS analysis, five tyrosine residues are the only modification sites with the following MS signal abundance: Y25 (10%), Y73 (24%), Y76 (3%), Y92 (7%), and Y115 (57%). The MS/MS data of the peptide fragment with modified Y73 (Table 29a) and that with modified Y115 (Table 29b) are as follows:

(a)

b^+	I^+		Sequence		y^+	y^{2+}
		**1**	N	**19**		
172.0716(7)<br>**172.0716(9) (–0.1)**		**2**	G	**18**		
300.1302(5)<br>**300.1303(1) (–0.2)**		**3**	Q	**17**		
401.1779<br>**401.1773 (1.5)**		**4**	T	**16**		1180.9011<br>**1180.9021 (–0.8)**
515.2209<br>**515.2212 (–0.6)**		**5**	N	**15**		1130.3772<br>**1130.3796 (–2.1)**
675.2515<br>**675.2523 (–1.2)**	133.0430<br>**133.0427 (2.3)**	**6**	C-Carb.	**14**		1073.3558<br>**1073.3525 (3.1)**
		**7**	**Y-Se**	**13**		993.3404

b	I		Sequence		y
					993.3409 (–0.5)
		8	Q	12	1448.6144<br>1448.6127 (1.2)
		9	S	11	1320.5559<br>1320.5488 (5.4)
	136.0756(9)<br>136.0756(5) (0.3)	10	Y	10	1233.5238<br>1233.5158 (6.5)
		11	S	9	1070.4605<br>1070.4608 (–0.3)
		12	T	8	983.4285<br>983.4291 (–0.6)
		13	M	7	882.3808<br>882.3809 (–0.1)
		14	S	6	751.3403<br>751.3405 (–0.3)
		15	I	5	664.3083<br>664.3082 (0.2)
		16	T	4	551.2242<br>551.2244 (–0.4)
		17	D	3	450.1765<br>450.1764 (0.2)
	133.0430<br>133.0427 (2.3)	18	C-Carb.	2	335.1496(0)<br>335.1496(0) (0.0)
		19	R	1	175.1190<br>175.1187 (1.7)

(b)

b	I		Sequence		y
	I^{+}: 110.0713<br>110.0710 (2.7)	1	H	20	y^{4+}: 650.2758<br>650.2766 (–1.2)
b^{+}: 251.1503<br>251.1493 (4.0)		2	I	19	
b^{+}: 364.2343<br>364.2342 (0.3)		3	I	18	
b^{+}: 463.3027<br>463.3026 (0.2)		4	V	17	
b^{+}: 534.3398<br>534.3399 (–0.2)		5	A	16	y^{2+}: 1068.3966<br>1068.3965 (0.1)
b^{2+}: 347.68888<br>347.68887 (0.03)	I^{+}: 133.04301<br>133.04300 (0.1)	6	C-Carb.	15	y^{2+}: 1032.8780<br>1032.8789 (–0.9)
b^{2+}: 412.21018<br>412.21021 (–0.1)		7	E	14	y^{2+}: 952.8627<br>952.8642 (–1.6)
b^{2+}: 440.7209<br>440.7210 (–0.2)		8	G	13	y^{2+}: 888.3414<br>888.3428 (–1.6)
b^{+}: 994.4775<br>440.4761 (3.2)		9	N	12	y^{2+}: 859.8307<br>859.8323 (–1.9)
		10	P	11	y^{2+}: 802.8092<br>802.8099 (–0.9)
		11	Y + Se	10	
		12	V	9	y^{+}: 970.4993<br>970.4986 (0.7)
		13	P	8	y^{+}: 871.4308<br>871.4307 (0.1)

		**14**	V	**7**	
	**I⁺**: 110.0713<br>**110.0710 (2.7)**	**15**	H	**6**	**y⁺**: 675.3097<br>**675.3088 (1.3)**
		**16**	F	**5**	**y⁺**: 538.2508<br>**538.2524 (–3.0)**
**b³⁺**: 774.9826<br>**774.9841 (–1.9)**		**17**	D	**4**	
**b³⁺**: 798.6616<br>**798.6633 (–2.1)**		**18**	A	**3**	**y⁺**: 276.1554<br>**276.1571 (–6.2)**
**b³⁺**: 827.6723<br>**827.6741 (–2.2)**		**19**	S	**2**	**y⁺**: 205.1183<br>**205.1182 (0.5)**
		**20**	V	**1**	**y⁺**: 118.08626<br>**118.08627 (–0.1)**

Table 29. MS/MS analysis of peptide fragment (a) NGQTNC**Y**^{73}QSYSTMSITDCR and (b) HIIVACEGNP**Y**^{115}VPVHFDASV of ribonuclease A-selenonium conjugate **13** after digestion. b^{n+}, peptide fragment starting from the N-terminus of the analyte with n positive charges; I^{+}, residue iminium ions; y^{n+}, peptide fragment starting from the C-terminus of the analyte with n positive charges; the integer, residue numbers in the corresponding fragments, integer in blue or orange indicates the corresponding b or y fragment was found, respectively; the number with 4 decimals in black, theoretical m/z numbers for the corresponding fragment; the bold numbers with 4 decimals in blue or orange, found m/z numbers for the corresponding b or y fragment, respectively; the number in bracket, the deviation of the theoretical m/z number from the found m/z number in ppm.

Preparation of lysozyme-selenonium conjugate 14

lysozyme, 50 µM → (selenoxide **1**, (5.0 mM, 100 equiv.); NaPi buffer (pH = 3.0, 200 mM), 30 °C, 16 h) → **14, lysozyme-selenonium conjugate**

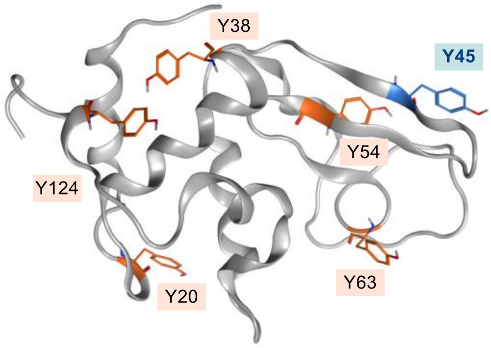

64%
only mono-modification

Under ambient atmosphere and at 20–25 °C, to an Eppendorf tube (1.5 mL), 32 µL of NaPi buffer (pH 3.0, c = 500 mM), 38 µL of UHQ-H_2O, and 10 µL of a selenoxide **1** stock solution (c = 50 mM, 0.50 µmol, 0.20 mg, 1.0 × 10^2 equiv., final concentration c = 5.0 mM) in phosphoric acid solution (c = 100 mM) in UHQ-H_2O were added. The mixture was vortexed for 5 sec., transferred into a Thermocycler pre-heated at 30°C and incubated at 30 °C for 30 min at 600 rpm. Next, 20 µL of a human lysozyme stock solution (c = 0.25 mM, 5.0 nmol, 74 µg, 1.0 equiv., final concentration c = 50 µM) in UHQ-H_2O was introduced to start the reaction. The mixture was vortexed for 5 sec., transferred into a Thermocycler pre-heated at 30 °C, and incubated at 30 °C for 18 h at 600 rpm. Subsequently, 100 µL of MeCN (final volume percentage 50%) and 15 µL of a freshly prepared Na_2SO_3 stock solution (c = 50 mM, 0.75 µmol, 95 µg, 75 equiv.) in UHQ-H_2O were added and the mixture was vortexed for 5 sec., transferred into a Thermocycler pre-heated at 25 °C, and incubated at 25 °C for 10 min at 600 rpm. The mixture was washed by ethyl acetate (100 µL × 3) to remove the selenide. The aqueous layer was gently purged by argon 2–4 mm above the solution via a needle (Φ 0.80 × 40 mm) for 90 sec. to remove residual organic solvent. The solution volume was adjusted to 100 µL by the addition of UHQ-H_2O (20 µL). The mixture was used for LC-MS analysis and for the sample preparation of LC-FTMS/MS analysis.

HRMS ESI-pos (m/z, averaged mass)

Due to low abundance of the monoisotopic signal, HRMS is performed on the most intense signal of the isotopic distribution. The theoretical value is calculated from the simulated isotopic pattern at instrument mass resolution.

Human lysozyme: calc'd for $C_{625}{}^{13}C_{8}H_{994}N_{200}O_{186}S_{10}{}^{10+}$ $[M+10H]^{10+}$, 1470.11(89); found, 1470.11(91). Deviation: –0.1 ppm.

Human lysozyme + Se, mono-modification: Calc'd for $C_{645}{}^{13}C_{8}H_{1004}N_{202}O_{187}S_{10}Se^{10+}$ $[M + 7 +9H]^{10+}$, 1507.51(849); found, 1507.51(842). Deviation: –0.05 ppm

Yield determination

The substrate human lysozyme contains two lysozyme-derived protein impurities, based on the results of LC-MS analysis (human lysozymes 2 and 3, Fig. 51d). Mono-Se modification was observed in both human lysozyme and impurities (Fig. 51e). Due to the difficulty in the separation of the different modified

proteins, the yield was determined based on the integration of all the peaks assigned to the modified proteins after PDA analysis at 328 nm. The yield of *Se*-modified human lysozyme was 64% (an average of three experiments).

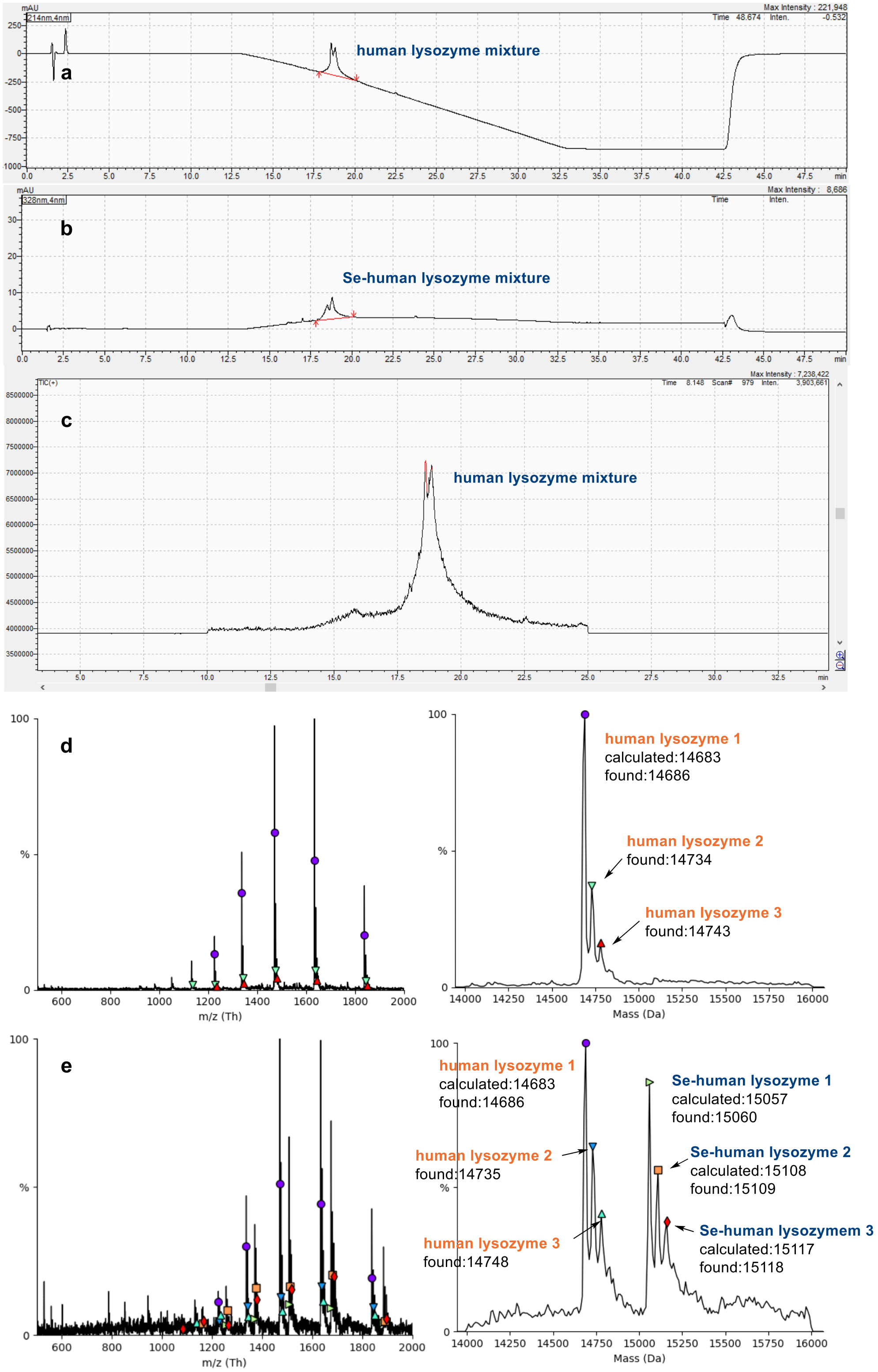

Figure 51. LC-MS analysis (Method A) of human lysozyme-selenonium conjugate 14. a) LC chromatogram (PDA 214 nm), b) LC chromatogram (PDA 328 nm) c) Total ion chromatogram, c) LC chromatogram (PDA 328 nm), d) Ion series (with Unidec fitting) and deconvoluted spectrum of the starting material, e) Ion series (with Unidec fitting) and deconvoluted spectrum of the reaction mixture. The deconvoluted MS numbers correspond to zero-charge mass (monoisotopic).

Site-selectivity determination

Sample preparation for LC-FTMS/MS analysis

The sample was prepared following the literature[21], using DTT as a reductant, 2-iodoacetamide as an alkylating reagent, and trypsin as a digestive enzyme. Under ambient atmosphere and at 20–25 °C, 80 µL of the lysozyme-selenonium conjugate **14** product mixture (for total protein: c = 50 µM, 4.0 nmol, 46 µg (weight of modification group not included), 1.0 equiv.) in HEPES buffer (pH 8.0, c = 100 mM), 75 µL of HEPES buffer (pH 8.0, c = 100 mM), 20 µL of SDS (10 wt%) in UHQ-H_2O, and 5 µL of a freshly prepared DTT solution (c = 0.20 M, 1.0 µmol, 1.5× 10^2 µg, 2.6 × 10^2 equiv.) in UHQ-H_2O were added to an Eppendorf tube (1.5 mL). The mixture was vortexed for 5 sec. and transferred into a Thermocycler pre-heated at 60 °C and incubated at 60 °C for 30 min at 1000 rpm. Next, 20 µL of a freshly prepared 2-iodoacetamide solution (c = 0.20 M, 1.0 µmol, 1.9× 10^2 µg, 2.6 × 10^2 equiv.) in HEPES buffer (pH 8.0, c = 100 mM) was added. The mixture was vortexed for 5 sec., transferred into a Thermocycler pre-heated at 25 °C, and incubated at 25 °C for 30 min at 1000 rpm. To the incubation, 7.0 µL of a SP3 beads solution (50 µg/µL, 3.6× 10^2 µg, protein:beads=1:8 (wt./wt.)) was added and vortexed for 5s. Subsequestly, 207 µL of absolute EtOH (final volume percentage 50%) was added and the mixture was transferred into a Thermocycler pre-heated at 25 °C and incubated at 25 °C for 5 min at 1000 rpm. The tube was put in a magnetic stand and the supernatant was removed. The tube containing beads was taken out from the magnetic stand. To the tube, 400 µL of 80% EtOH was added and pipetted to homogenize the solution. The tube was replaced on the magnetic stand and the supernatant was removed. After this washing step was repeated three times, 97 µL of NH_4HCO_3 buffer (pH 9.0, c = 100 mM) and 3.3 uL trypsin (0.2 mg/mL, 0.66 µg, protein: trypsin=70:1 (wt./wt.)) was added to fragment the protein (blue dashed line in Fig. 52). Then the mixture was sonicated for 30 sec. and transferred into a Thermocycler pre-heated at 37 °C and incubated at 37 °C for 18 h at 1000 rpm. After the digestion, the mixture was centrifuged at 15,000 ×g and at 25 °C for 5 min, and the tube was placed on the magnetic stand. The supernatant was taken and analyzed by MS/MS to determine the site of modification.

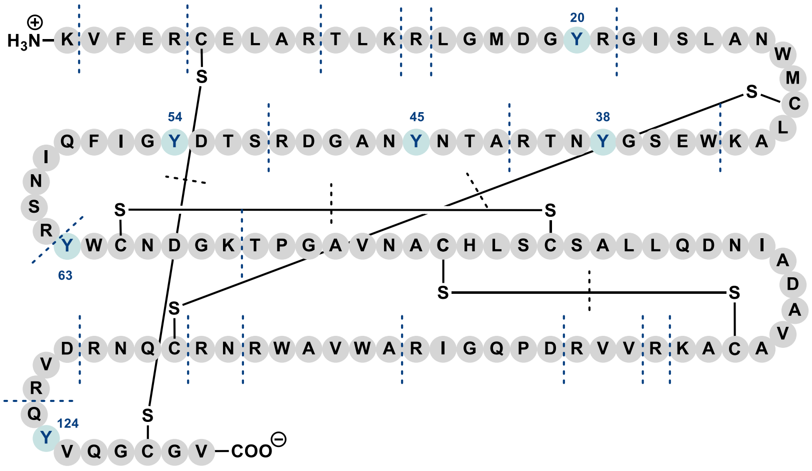

Figure 52. Fragmentation of human lysozyme-selenonium conjugate **14**. Black dashed lines, disulfide bond broke by DTT reduction; blue dashed line, peptide bonds cleaved by trypsin.

MS/MS results

Based on the results of MS/MS analysis, four tyrosine residues are the only modification sites with the following MS signal abundance: Y45 (75%), Y54 (5%), Y63 (13%), and Y124 (6%). The MS/MS data of

the peptide fragment with Y45 is as follows:

b^+	b^{2+}		Sequence		y^+	y^{2+}	y^{3+}
		**1**	A	**9**			452.4829<br>**452.4833 (–0.9)**
173.0921<br>**173.0920 (0.6)**		**2**	T	**8**		642.7022<br>**642.7020 (0.3)**	428.8039<br>**428.8037 (0.5)**
287.1350<br>**287.1345 (1.7)**		**3**	N	**7**		592.1784<br>**592.1780 (0.7)**	
824.1942<br>**824.1918 (2.9)**	412.6007<br>**412.6008 (–0.2)**	**4**	**Y + Se**	**6**		535.1569<br>**535.1569 (0)**	
	469.6222<br>**469.6227 (–1.1)**	**5**	N	**5**	532.2474<br>**532.2469 (0.9)**	266.6273<br>**266.6271 (0.8)**	
	505.1407<br>**505.1385 (4.4)**	**6**	A	**4**	418.2045<br>**418.2041 (1.0)**	209.6059<br>**209.6055 (1.9)**	
	533.6515<br>**533.6565 (–9.4)**	**7**	G	**3**	347.1674<br>**347.1670 (1.2)**		
		**8**	D	**2**	290.1459<br>**290.1457 (0.7)**		
		**9**	R	**1**	175.1190<br>**175.1188 (1.1)**		

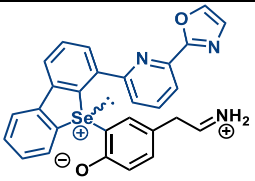

Immonium ion

m/z calculated: 510.0715

m/z found: 510.0703

Table 30. MS/MS analysis of peptide fragment ATNYNAGDR of human lysozyme-selenonium conjugate **14** after digestion. b^{n+}, peptide fragment starting from the N-terminus of the analyte with n positive charges; y^{n+}, peptide fragment starting from the C-terminus of the analyte with n positive charges; the integer, residue numbers in the corresponding fragments, integer in blue or orange indicates the corresponding b or y fragment was found, respectively; the number with 4 decimals in black, theoretical m/z numbers for the corresponding fragment; the bold numbers with 4 decimals in blue or orange, found m/z numbers for the corresponding b or y fragment, respectively; the number in bracket, the deviation of the theoretical m/z number from the found m/z number in ppm.

Transformations of *Se*-modified peptides and proteins

General considerations for transformations of *Se*-modified peptides and proteins

To avoid tedious purification of *Se*-modified peptide or protein linchpin, the product mixture of *Se*-modification step was directly used for selenonium transformations. However, the pH of the product mixture may not match the required pH for the subsequent transformations. Stock solutions of NaOH and TFA in UHQ-H_2O were employed for adjusting the pH of the product mixture and the amount was only dependent on the amount of buffer species in the product mixture used in the transformation instead of the amount of *Se*-modified peptide or protein used in the transformation. Similarly, the amount of the chelator used to complex the metal ions in work-up step was only dependent on the amount of the metal ions in the reaction mixture. Additionally, because the amount of the reagents used in the transformation step is in large excess to the *Se*-modified peptides and proteins, we recommend to report the reaction conditions with the final concentration of the reagents in the transformation mixture rather than the equivalence of the reagents to *Se*-peptide or *Se*-proteins. Based on our experience, at the same final concentration of the reagents in the transformation mixture, variation of the final concentration of the substrate *Se*-peptide or *Se*-protein (as well as the equivalance of the reagents to the substrate) in the range of 5–50 µM did not affect the yield of the transformation.

Photoreaction set-up

For the photoreaction, because the long peptides and proteins can be denatured at elevated temperature, the apparatus below was used for the photoreactions of *Se*-bivalirudin **8**, *Se*-insulin **11** and *Se*-ubiquitin **12**, to keep the reaction temperature at approximately 0 °C during light irradiation of less than 30 min. The Eppendorf tube containing the reaction mixture was floated on the ice-water bath in a small Dewar equipped with Teflon-coated magnetic stirring bar. Then, the Dewar was placed under 1 Kessil PR160-390 nm LED (2.5 cm away from the lamp) and irradiated for desired time. (**Note:** after 30 minutes of irradiation, all the ice in the Dewar will have melted, so it is necessary to add more ice before continuing the reaction if a longer irradiation time is required).

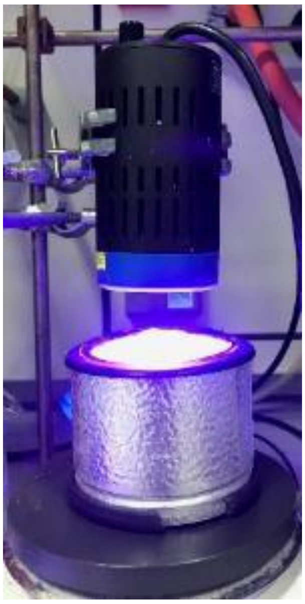

Figure 53. Apparatus used for the photoreactions of *Se*-modified peptides and *Se*-modified proteins.

Quantification for transformations of *Se*-modified bivalirudin and *Se*-modified proteins

Considering that only a small even single-atom modification was introduced in the final products, it can be assumed that the extinction coefficient for the absorption of peptidyl groups at 214 nm should be nearly identical for the final product and the corresponding unmodified peptide or protein. Therefore, the unmodified peptides or proteins can be used as proxys to prepare the LC calibration curves (Figs. 32–33, 35) for the quantification of the corresponding products. If the final product can be separated from other peptide or protein components in LC, the concentration of the final product in the transformation mixture was determined via the peak integration of the product after PDA analysis at 214 nm. With the standard curve, the concentration of the product in the mixture can be calculated as follows:

$$\text{product concentration (mM)} = \frac{\text{peak area of the desired product/LC injection volume (µL)}}{\text{the slope of the standard curve } (\text{mM}^{-1})/5\ (\text{µL})}$$

And the yield of the reaction was calculated as follows:

$$\text{yield of product} = \frac{\text{product concentration in reaction mixture (mM)} \times \text{sample volume (µL)}}{\text{selenonium conjugate amount used in reaction (nmol)}} \times 100\%$$

If no separation or only bad separation was achieved (commonly for protein substrates), the yield was determined based on both the peak integration of all protein species after PDA analysis at 214 nm and the conversion obtained from the zero-charge spectra after deconvolution. The total concentration of proteins in the mixture after the transformation can be calcuclated as follows:

$$\text{total protein concentration (mM)} = \frac{\text{summed peak areas of proteins/LC injection volume (µL)}}{\text{the slope of the standard curve } (\text{mM}^{-1})/5\ (\text{µL})}$$

Because the product mixture of *Se*-modification step was directly used for selenonium transformations (which contained both *Se*-modified proteins and unreacted proteins), the total protein recovery was calculated as follows:

$$\text{recovery} = \frac{\text{total protein concentration (mM)} \times \text{sample volume (µL)}}{\text{total protein amount used in reaction (nmol)}} \times 100\%$$

Then, all signals that originate from proteins in total ion chromatogram (TIC) were deconvoluted and, based on the areas of different proteins in the deconvoluted zero-charge spectrum, the conversion of the desired product can be calculated as follows:

$$\text{conversion of product} = \frac{\text{zero-charge spectrum peak area of desired product}}{\text{zero-charge spectrum peak areas of all proteins}} \times 100\%$$

With the percentage of the *Se*-protein in the product mixture of the *Se*-modification step used in the transformation,

$$\text{percentage of Se-protein} = \frac{\text{selenonium conjugate amount used in reaction (nmol)}}{\text{total protein amount used in reaction (nmol)}} \times 100\%$$

The yield of the transformation can be calculated as follows:

$$\text{yield of final product} = \frac{\text{protein recovery (\%)} \times \text{conversion(\%)}}{\text{percentage of Se-protein in total protein used in the reaction(\%)}}$$

However, due to the unknown extinction coefficients of the final products and unkown response factors

for MS analysis of unmodified proteins and their modification products, the determined yields should be considered semi-quantitative.

Preparation of iodo-oxytocin 22

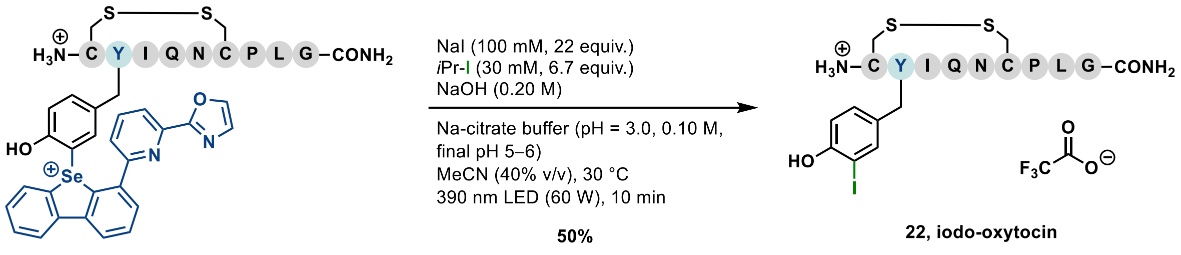

oxytocin-selenonium conjugate 7, 0.90 mM

Under ambient atmosphere and at 20–25 °C, a scintillation vial (20 mL) equipped with Teflon-coated magnetic stirring bar was charged with the reductant NaI (150 mg, 1.0 mmol, 22 equiv., final concentration c = 100 mM). Then, 5.0 mL of the oxytocin-selenonium conjugate **7** stock solution (c = 0.90 mM, 4.5 µmol, 6.2 mg, 1.0 equiv. final concentration c = 0.45 mM) in Na-citrate buffer (pH 3.0, c = 0.20 M, 1.0 mmol, final concentration c = 0.10 M) containing $MgCl_2$ (c = 0.20 M, final concentration c = 0.10 M) and 4.0 mL of the cosolvent MeCN (final volume percentage 40%) were added and the mixture was stirred at 25 °C until all solids dissolved (ca. 3 min). Next, 1.0 mL of NaOH stock solution (c = 2.0 M, 2.0 mmol, 80 mg, 2.0 equiv. to citrate buffer species, final concentration c = 0.20 M) was introduced dropwise over 1 min to elevate the solution pH to 5–6 (indicated by general pH test paper). Then the mixture was degassed by gently purging argon through the solution via a needle (Φ 0.80 × 120 mm) for 5 min. Thereafter, the iodo-source *i*Pr-I (51 mg, 30 µL, 0.30 mmol, 6.7 equiv.) was added and the mixture was degassed for an additional 1 min. Then, the vial was closed by a screw cap, placed between 2 Kessil PR160-390 nm LEDs (2.5 cm away from each lamp), and irradiated for 10 min. The temperature of the reaction mixture was kept at approximately 30°C through the use of a cooling fan. The mixture was washed with ethyl acetate (5 mL × 2) to remove iodine and selenide, and the aqueous layer was further diluted with UHQ-H_2O (20 mL) and subjected to lyophilization. The residue was purified by preparative reverse phase HPLC (YMC Pro C18, 150 × 30 mm, 5 µm) with an eluent mixture of TFA 0.1% in water/MeOH (60/40 to 55/45 in 9 min, then to 50/50 in 4 min) at a flow rate of 42 mL/min at 308 K. The fractions containing the desired product were combined and evaporated under reduced pressure at 30 °C to remove MeOH, and were further lyophilized to afford the desired compound as a colorless solid (2.8 mg, 50%).

NMR Spectroscopy:

^{1}**H NMR** (600 MHz, DMF-d_7, 298 K, δ): 10.59 (s, 1H), 8.63 (s, 2H), 8.45 (d, *J* = 7.8 Hz, 1H), 8.20 (d, *J* = 7.8 Hz, 1H), 7.94 (t, *J* = 5.8 Hz, 2H), 7.70 (d, *J* = 2.0 Hz, 1H), 7.67 (s, 1H), 7.56 (s, 1H), 7.30 (s, 1H), 7.25 (d, *J* = 7.3 Hz, 1H), 7.11 (s, 1H), 7.07 (s, 1H), 6.92 (s, 1H), 6.90 (d, *J* = 8.2 Hz, 1H), 5.05 (d, *J* = 5.8 Hz, 1H), 4.80 (s, 1H), 4.73 (q, *J* = 7.0 Hz, 1H), 4.47 – 4.30 (m, 2H), 4.10 (dt, *J* = 9.7, 5.0 Hz, 1H), 4.06 (d, *J* = 6.9 Hz, 1H), 3.88 (d, *J* = 6.3 Hz, 1H), 3.85 (d, *J* = 6.3 Hz, 1H), 3.73 (d, *J* = 5.8 Hz, 1H), 3.70 (d, *J* = 5.9 Hz, 2H), 3.37 (d, *J* = 14.3 Hz, 1H), 3.30 (dd, *J* = 13.8, 6.3 Hz, 1H), 3.18 (dd, *J* = 13.6, 7.2 Hz, 1H), 2.99 – 2.95 (m, 1H), 2.88 (d, *J* = 7.0 Hz, 1H), 2.79 (d, *J* = 6.2 Hz, 1H), 2.43 – 2.33 (m, 2H), 2.19 (dt, *J* = 9.2, 5.0 Hz, 1H), 2.10 – 1.90 (m, 6H), 1.76 – 1.52 (m, 4H), 1.28 – 1.20 (m,

1H), 0.99 (d, *J* = 6.8 Hz, 3H), 0.91 (t, *J* = 7.3 Hz, 3H), 0.90 (d, *J* = 6.2 Hz, 3H), 0.86 (d, *J* = 6.2 Hz, 3H).

^{13}C NMR (151 MHz, DMF-d_7, 298 K, δ): 175.4, 172.9(2), 172.8(9), 172.7, 172.2, 171.9, 171.9, 171.7, 169.4, 156.1, 139.7, 130.9, 114.9, 84.1, 61.7, 60.6, 55.5, 55.3, 52.4, 52.3, 51.2, 47.6, 42.7, 42.1, 40.0, 36.9, 36.6, 36.5, 31.9, 29.4, 26.7, 25.8, 25.1, 24.9, 23.1, 21.1, 15.6, 11.3.

^{19}F NMR (565 MHz, DMF-d_7, 298 K, δ): –74.7 (br).

HRMS ESI-pos (m/z) calc'd for $C_{43}H_{66}N_{12}O_{12}S_2I^+$ [M+H]$^+$, 1133.3404; found, 1133.3406. Deviation: –0.2 ppm.

Preparation of bromo-bivalirudin 23

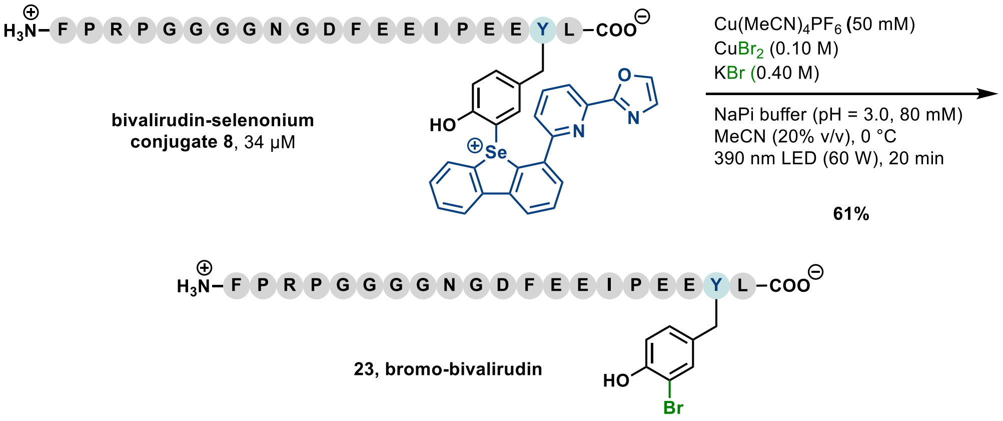

Preparation of stock solutions

Stock solution A: Under ambient atmosphere, a scintillation vial (4 mL) equipped with a Teflon-coated magnetic stirring bar was charged with $CuBr_2$ (56 mg, 0.25 mmol, 1.0 equiv.), KBr (120 mg, 1.0 mmol, 4.0 equiv.), and the solvent UHQ-H_2O (1.0 mL, $CuBr_2$: c = 0.25 M; KBr: c = 1.0 M). The mixture was stirred at 25 °C until all solids dissolved. The vial was degassed by gently purging argon through the solution via a needle (Φ 0.80 × 120 mm) for 5 min and stored under nitrogen atmosphere for further usage.

Stock solution B: Under nitrogen atmosphere, a scintillation vial (4 mL) equipped with a Teflon-coated magnetic stirring bar was charged with $Cu(MeCN)_4PF_6$ (93 mg, 0.25 mmol) and the solvent MeCN (1.0 mL, c = 0.25 M). The mixture was stirred at 25 °C for 10 min and stored under nitrogen atmosphere.

Photoreaction set-up

Under argon atmosphere and at 20–25 °C, 20 µL of the stock solution A ($CuBr_2$: c = 0.25 M, 5.0 µmol, 1.1 mg, 2.9 × 10^3 equiv., final concentration c = 0.10 M; KBr: c = 1.0 M, 20 µmol, 2.4 mg, 1.2 × 10^4 equiv., final concentration c = 0.40 M) in UHQ-H_2O and 10 µL of the stock solution B ($Cu(MeCN)_4PF_6$: c = 0.25 M, 2.5 µmol, 0.93 mg, 1.5 × 10^3 equiv., final concentration c = 50 mM) in MeCN (final volume percentage 20%) were added to an Eppendorf tube (1.5 mL) under argon atmosphere. Then 20 µL of a bivalirudin-selenonium salt **8** stock solution (84 µM, 1.7 nmol, 4.3 µg, 1.0 equiv. final concentration c = 34 µM) in NaPi buffer (pH 3.0, c = 0.20 M, final concentration c = 80 mM) was added and the mixture was vortexed for 5 sec.. The Eppendorf tube was floated on ice-water bath and the ice-water bath was placed under 1 Kessil PR160-390 nm LED (2.5 cm away from the LED) and irradiated for 20 min (see the "Photoreaction set-up" section). The temperature of the reaction mixture was kept at approximately 0°C through the use

of the ice-water bath. Subsequently, 30 µL of an EDTA-Na_2 stock soluition (pH 8.5, 0.3 M, 9.0 µmol, 3.4 mg, 1.2 equiv. to copper ions) in UHQ-H_2O was added to complex copper ion. The mixture was vortexed for 5 sec., transferred into a Thermocycler pre-heated at 25 °C, and incubated at 25 °C for 5 min at 600 rpm. The resulting solution was stored at –20 °C and directly used for LC-MS analysis for yield determination by LC-MS post-run PDA analysis at 214 nm (61%, average of three experiments) as well as for LC/Q-TOF-MS/MS analysis for product characterization without any further purification.

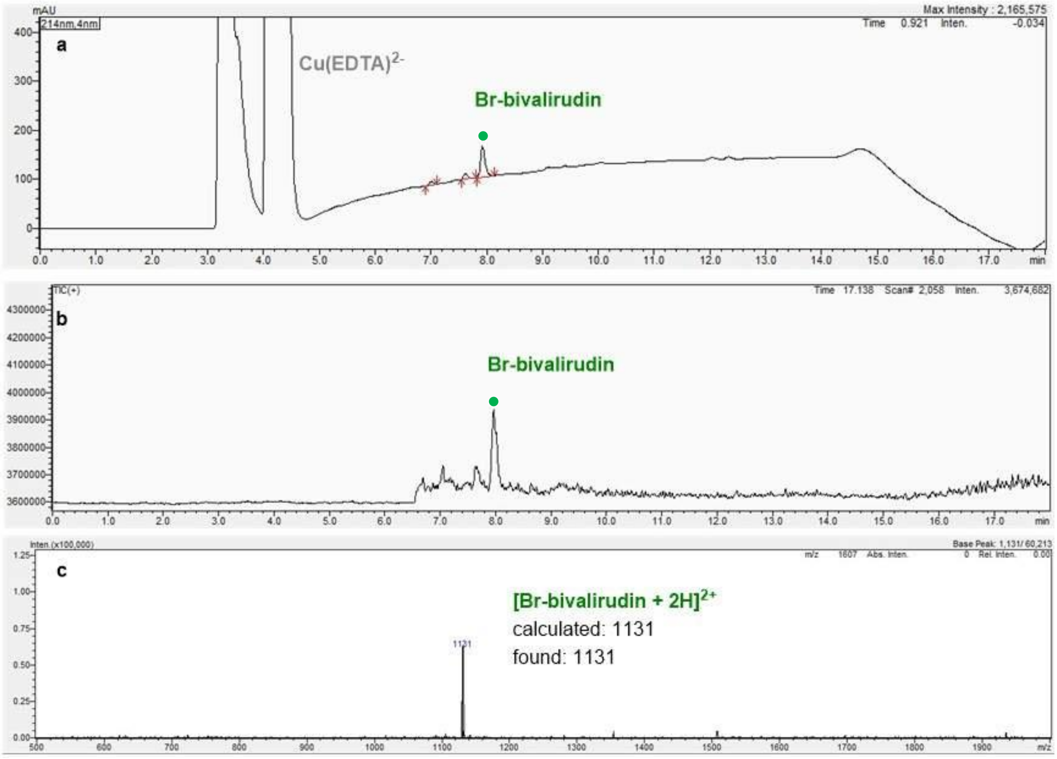

Figure 54. LC-MS analysis (Method A) of bromo-bivalirudin **23** for yield determination. a) LC chromatogram (PDA 214 nm) and the peak used for integration b) total ion chromatogram c) Product peak ion series.

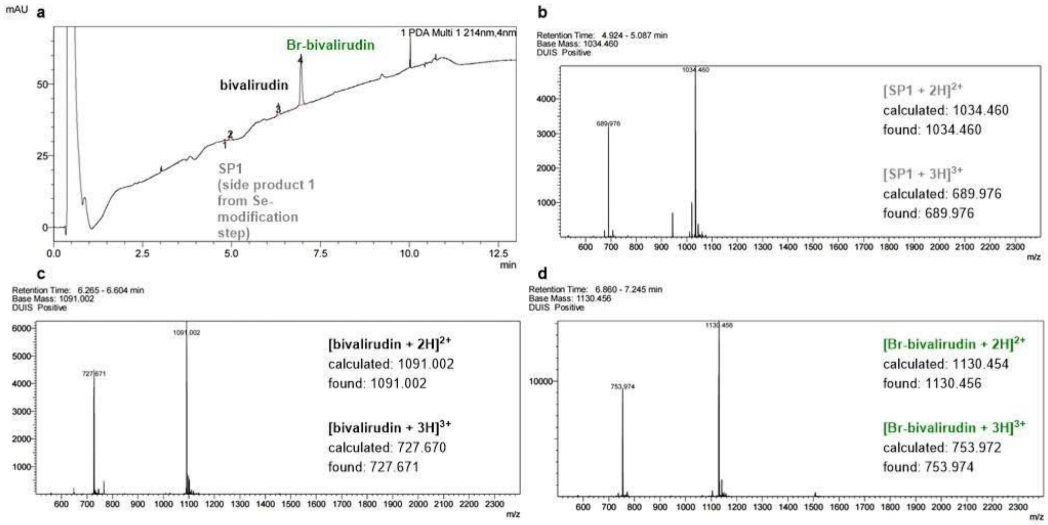

Figure 55. LC/Q-TOF-MS analysis (Method B) of bromo-bivalirudin **23** for characterization. a) LC chromatogram (PDA 214 nm) b) Peak 2 (side product 1 from previous *Se*-modification step, SP1) ion series c) Peak 3 (bivalirudin) ion series d) Peak 4 (product, Br-bivalirudin) ion series.

LC/Q-TOF-MS/MS results of peak 4 (bromo-bivalirudin 23)

b^+	b^{2+}		Sequence		y^+	y^{2+}
148.0757	74.5415	1	F	20	2257.9036	1129.4555<br>**1129.4556 (–0.1)**
245.1285<br>**245.1283 (0.8)**	123.0679	2	P	19	2110.8352	1055.9213
401.2296<br>**401.2298 (–0.5)**	201.1185	3	R	18	2013.7825	1007.3949
498.2824<br>**498.2846 (–4.4)**	249.6449	4	P	17	1857.6814<br>**1857.6816 (–0.1)**	929.3443
555.3038<br>**555.3034 (0.7)**	278.1556	5	G	16	1760.6286	880.8180
612.3253<br>**612.3258 (–0.8)**	306.6663	6	G	15	1703.6071	852.3072
669.3468<br>**669.3481 (–1.9)**	335.1770	7	G	14	1646.5857	823.7965
726.3682<br>**726.3679 (0.4)**	363.6878	8	G	13	1589.5642	795.2858
840.4112<br>**840.4120 (–1.0)**	420.7092	9	N	12	1532.5427	766.7750
897.4326<br>**897.4320 (0.7)**	449.2200	10	G	11	1418.4998	709.7536
1012.4596<br>**1012.4602 (–0.6)**	506.7334<br>**506.7339 (–1.0)**	11	D	10	1361.4783	681.2428
1159.5280<br>**1159.5285 (–0.4)**	580.2677<br>**580.2675 (0.3)**	12	F	9	1246.4514<br>**1246.4531 (–1.4)**	623.7294
1288.5706<br>**1288.5725 (–1.5)**	644.7889<br>**644.7887 (0.3)**	13	E	8	1099.3830<br>**1099.3818 (1.1)**	550.1952
1417.6132<br>**1417.6136 (–0.3)**	709.3102<br>**709.3113 (–1.6)**	14	E	7	970.3404<br>**970.3419 (–1.5)**	485.6739
1530.6972<br>**1530.6970 (0.1)**	765.8523<br>**765.8526 (–0.4)**	15	I	6	841.2978<br>**841.2982 (–0.5)**	421.1526
1627.7500	814.3787<br>**814.3796 (–1.1)**	16	P	5	728.2137<br>**728.2139 (–0.3)**	364.6105
1756.7926<br>**1756.7921 (0.3)**	878.9000<br>**878.9000 (0)**	17	E	4	631.1610<br>**631.1617 (–1.1)**	316.0842
1885.8352<br>**1885.8398 (–2.4)**	943.4213<br>**943.4220 (–0.7)**	18	E	3	502.1184<br>**502.1189 (–1.0)**	251.5629
2126.8090	1063.9082<br>**1063.9090 (–0.8)**	19	**Y + Br**	2	373.0758<br>**373.0760 (–0.5)**	187.0416
2239.8931	1120.4502<br>**1120.4505 (–0.3)**	20	L	1	132.1020<br>**132.1017 (2.3)**	66.5546

Immonium ion

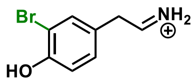

m/z calculated: 213.9861, 215.9841

m/z found: 213.9871, 215.9841

Table 31. LC/Q-TOF-MS/MS analysis of **23**. Sample was directly measured without any operation. Peptide concentration for analytical sample: 0.054 mg/mL. b^{n+}, peptide fragment starting from the N-terminus of the analyte with n positive charges; y^{n+}, peptide fragment starting from the C-terminus of the analyte with n positive charges; the integer, residue numbers in the corresponding fragments, integer in blue or orange indicates the corresponding b or y fragment was found, respectively; the number with 4

decimals in black, theoretical m/z numbers for the corresponding fragment; the bold numbers with 4 decimals in blue or orange, found m/z numbers for the corresponding b or y fragment, respectively; the number in bracket, the deviation of the theoretical m/z number from the found m/z number in ppm.

Preparation of coumarin-bivalirudin 24

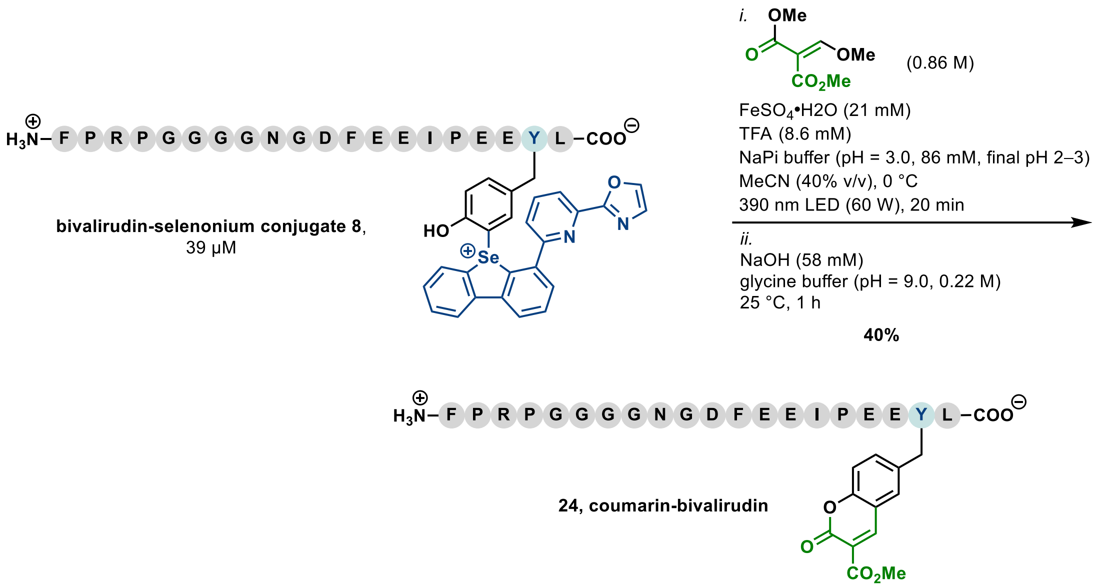

Photoarylation of the Michael acceptor: Under argon atmosphere and at 20–25 °C, 20 µL of a bivalirudin-selenonium salt **8** stock solution (90 µM, 1.8 nmol, 4.6 µg, 1.0 equiv., final concentration c = 39 µM) in NaPi buffer (pH 3.0, c = 0.20 M, 4.0 µmol, final concentration c = 86 mM) was added to an Eppendorf tube (1.5 mL). 4.0 µL of a TFA stock solution (0.10 M, 0.40 µmol, 46 µg, 0.10 equiv. to NaPi buffer species, final concentration c = 8.6 mM) in UHQ-H_2O were added to acidify the mixture, which prevented the precipitation of Fe^{2+} (added in next step) by phosphate buffer. Then, 4.0 µL of freshly-prepared $FeSO_4\cdot 7H_2O$ stock solution (0.25 M, 1.0 µmol, 0.28 mg, 5.6×10^2 equiv., final concentration c = 21 mM) in UHQ-H_2O was added, followed by the addition of 10.7 µL of MeCN (final volume percentage 40%) and 8.0 µL of dimethyl 2-methoxymethylene-malonate stock solution (5.0 M, 40 µmol, 7.0 mg, 2.2×10^4 equiv., final concentration c = 0.86 M) in MeCN. The mixture (final solution pH 2–3, indicated by general pH test paper) was vortexed for 5 sec. and the Eppendorf tube was floated on ice-water bath. The ice-water bath was placed under 1 Kessil PR160-390 nm LED (2.5 cm away from the LED) and irradiated for 20 min (see the "Photoreaction set-up" section). The temperature of the reaction mixture was kept at approximately 0°C through the use of the ice-water bath. Subsequently, 12 µL of UHQ-H_2O and 21 µL of MeCN (final volume percentage 50%) were added to the mixture under argon atmosphere. The mixture was vortexed for 5 sec., transferred into a Thermocycler pre-heated at 25 °C, and incubated at 25 °C for 10 min at 600 rpm. Next, the mixture was washed with ethyl acetate (80 µL × 2) to remove the excessive dimethyl 2-methoxymethylene-malonate and the aqueous layer was gently purged by argon 2–4 mm above the solution via a needle (Φ 0.80 × 40 mm) for 90 sec. to remove residual organic solvent. The solution volume was adjusted to 40 µL by the addition of UHQ-H_2O (ca. 10 µL). Then, 40 µL of MeCN (final volume percentage 50%) was added and the mixture was vortexed for 5 sec., transferred into a Thermocycler pre-heated at 25 °C, and incubated at 25 °C for 10 min at 600 rpm. The resulting white suspension was centrifuged at 20–25 °C for 1 min at 13,300 rpm and the pellets were discarded. The supernatant was used for the coumarin formation immediately.

Coumarin formation under basic conditions: Under ambient atmosphere and at 20–25 °C, 80 µL of glycine-Na buffer (pH 9.0, c = 0.50 M, 40 µmol, 10 equiv. to NaPi buffer species, final concentration c = 0.22 M) and 21 µL of a NaOH stock solution (0.50 M, 11 µmol, 0.42 mg, 2.6 equiv. to NaPi buffer species, final concentration c = 58 mM) in UHQ-H_2O were added to an Eppendorf tube (1.5 mL) and the mixture was vortexed for 5 sec. (**Important:** Do not directly mix the NaOH solution with the supernatant, which can lead to the decomposition of the peptide). Subsequently, all the supernatant from the previous photoarylation step (ca. 80 µL, containing ca. 4 µmol NaPi buffer species) was added and the mixture was vortexed for 5 sec., resulting a color change of the solution to pale yellow. The mixture was transferred into a Thermocycler pre-heated at 25 °C, and incubated at 25 °C for 1 h at 600 rpm. The resulting mixture was stored at -20 °C and directly used for LC-MS analysis for yield determination by LC-MS post-run PDA analysis at 214 nm (40%, average of three experiments) as well as for LC/Q-TOF-MS/MS analysis for product characterization without any further purification.

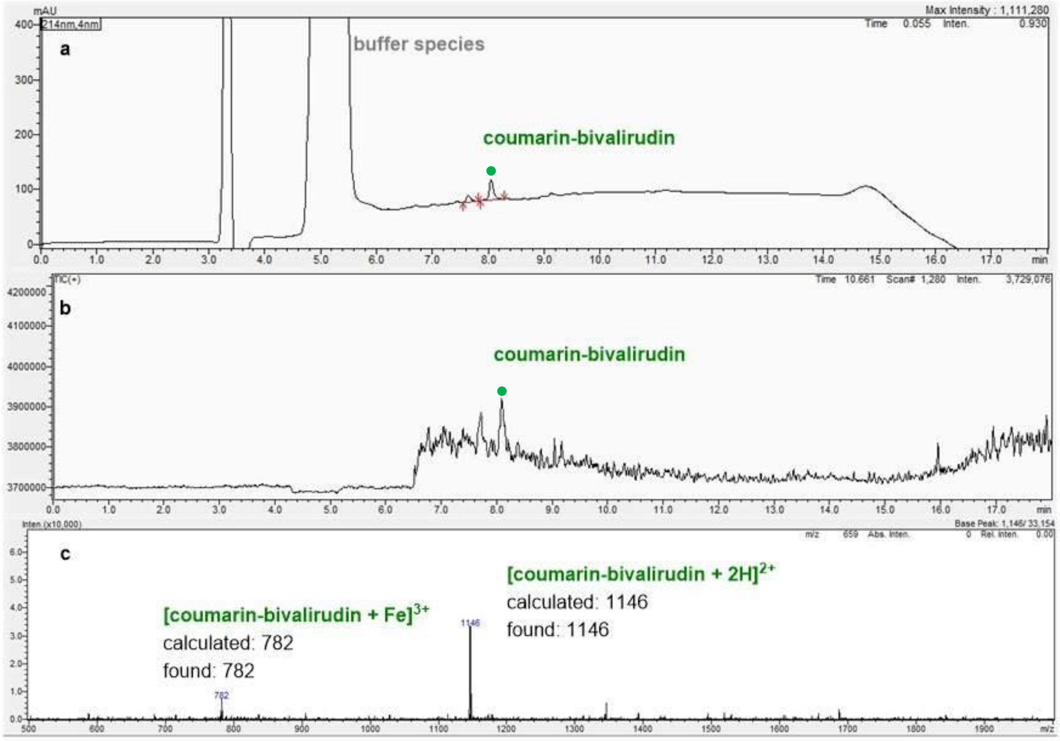

Figure 56. LC-MS analysis (Method A) of coumarin-bivalirudin **24** for yield determination. a) LC chromatogram (PDA 214 nm) and the peak used for integration b) total ion chromatogram c) Product peak ion series.

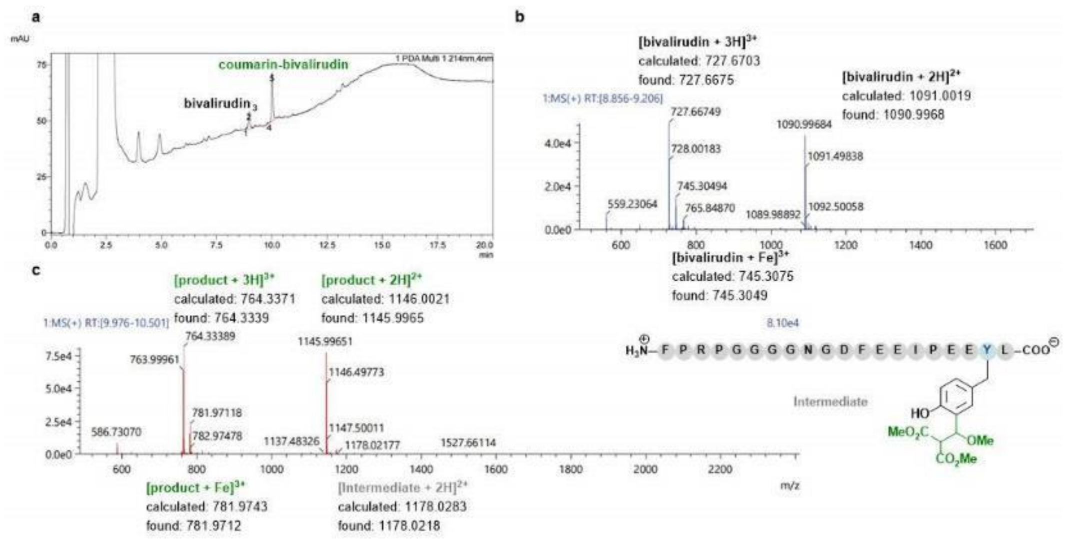

Figure 57. LC/Q-TOF-MS analysis (Method B) of coumarin-bivalirudin **24** for characterization. a) LC chromatogram (PDA 214 nm) b) Peak 2 (bivalirudin) ion series c) Peak 5 (coumarin-bivalirudin and the intermediate of trace amount) ion series.

LC/Q-TOF-MS/MS results of peak 5 (coumarin-bivalirudin 24)

b^{+}	b^{2+}		Sequence		y^{+}	y^{2+}
148.0757	74.5415	1	F	20	2289.9935	1145.5004
245.1285<br>**245.1283 (0.8)**	123.0679	2	P	19	2142.9251	1071.9662
401.2296<br>**401.2276 (5.0)**	201.1185	3	R	18	2045.8724	1023.4398
498.2824<br>**498.2809 (3.0)**	249.6449	4	P	17	1889.7712<br>**1889.7629 (4.4)**	945.3893
555.3038<br>**555.3027 (2.0)**	278.1556	5	G	16	1792.7185	896.8629
612.3253<br>**612.3226 (4.4)**	306.6663	6	G	15	1735.6970	868.3522
669.3468<br>**669.3439 (4.3)**	335.1770	7	G	14	1678.6756	839.8414
726.3682<br>**726.3653 (4.0)**	363.6878<br>**363.6867 (3.0)**	8	G	13	1621.6541	811.3307
840.4112<br>**840.4070 (5.0)**	420.7092<br>**420.7071 (5.0)**	9	N	12	1564.6326	782.8200
897.4326<br>**897.4275 (5.7)**	449.2200<br>**449.2185 (3.3)**	10	G	11	1450.5897	725.7985
1012.4596<br>**1012.4553 (4.2)**	506.7334<br>**506.7311 (4.5)**	11	D	10	1393.5682	697.2878
1159.5280<br>**1159.5224 (4.8)**	580.2677<br>**580.2653 (4.1)**	12	F	9	1278.5413<br>**1278.5385 (2.2)**	639.7743
1288.5706<br>**1288.5645 (4.7)**	644.7889<br>**644.7859 (4.7)**	13	E	8	1131.4729<br>**1131.4730 (–0.1)**	566.2401
1417.6132<br>**1417.6063 (4.9)**	709.3102<br>**709.3074 (3.9)**	14	E	7	1002.4303<br>**1002.4263 (4.0)**	501.7188
1530.6972<br>**1530.6905 (4.4)**	765.8523<br>**765.8493 (3.9)**	15	I	6	873.3877<br>**873.3832 (5.2)**	437.1975
1627.7500	814.3787<br>**814.3740 (5.8)**	16	P	5	760.3036<br>**760.2998 (5.0)**	380.6555

1756.7926	878.9000<br>**878.8963 (4.2)**	**17**	E	**4**	663.2509<br>**663.2486 (3.5)**	332.1291
1885.8352	943.4213<br>**943.4172 (4.3)**	**18**	E	**3**	534.2083<br>**534.2057 (4.9)**	267.6078
2158.8989	1079.9531<br>**1079.9490 (3.8)**	**19**	**Coumarin**	**2**	405.1657<br>**405.1635 (5.4)**	203.0865
2271.9830	1136.4951<br>**1136.4888 (5.5)**	**20**	L	**1**	132.1017<br>**132.1009 (6.1)**	66.5546

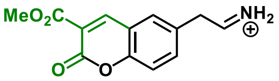

Immonium ion

m/z calculated: 246.0760

m/z found: 246.0745

Table 32. LC/Q-TOF-MS/MS analysis of **24**. Sample was directly measured without any operation. Peptide concentration for analytical sample: 0.026 mg/mL. b^{n+}, peptide fragment starting from the N-terminus of the analyte with n positive charges; y^{n+}, peptide fragment starting from the C-terminus of the analyte with n positive charges; the integer, residue numbers in the corresponding fragments, integer in blue or orange indicates the corresponding b or y fragment was found, respectively; the number with 4 decimals in black, theoretical m/z numbers for the corresponding fragment; the bold numbers with 4 decimals in blue or orange, found m/z numbers for the corresponding b or y fragment, respectively; the number in bracket, the deviation of the theoretical m/z number from the found m/z number in ppm.

Preparation of *p*-F-phenyl-bivalirudin 25

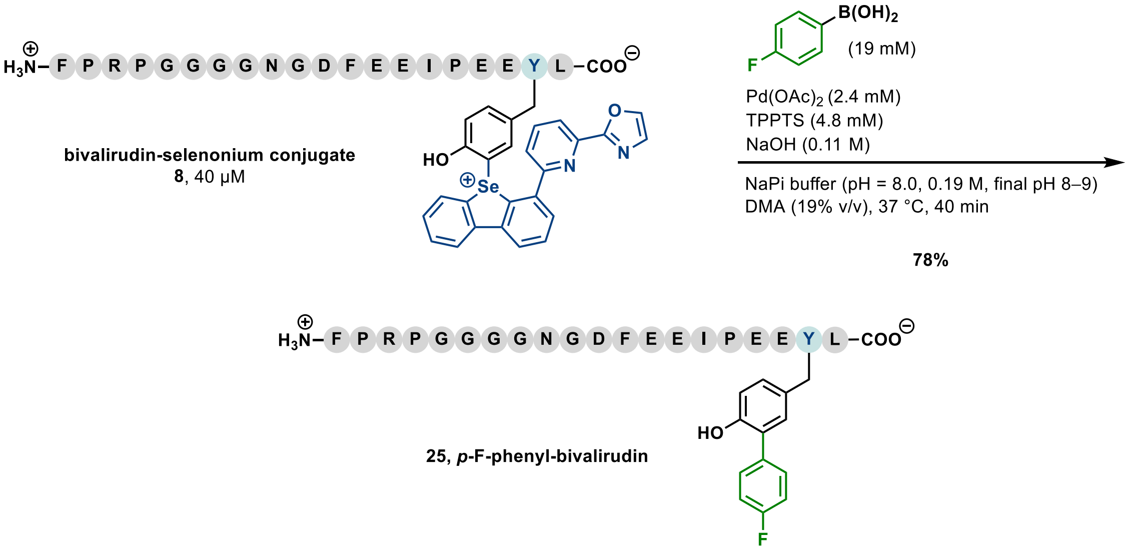

Preparation of the catalyst stock solution

Stock solution of palladium catalyst: Under argon atmosphere and at 20–25 °C, an Eppendorf tube (1.5 mL) was charged with $Pd(OAc)_2$ (0.60 mg, 2.5 µmol, 1.0 equiv., final concentration c = 13 mM) and the ligand 3,3',3''-phosphanetriyltris(benzenesulfonic acid) trisodium salt (TPPTS) (2.8 mg, 5.0 µmol, 2.0 equiv., final concentration c = 25 mM). Then, 100 µL of NaPi buffer (pH 8.0, c = 0.50 M, final concentration c = 0.25 M), 50 µL of UHQ-water, and 50 µL of the cosolvent DMA (final volume percentage 25%) were added. The mixture was vortexed for 20 sec., transferred into a Thermocycler pre-heated at 40 °C, and incubated at 40 °C for 15 min at 1,000 rpm to give a red-brown solution. The solution was stored at –20 °C for further usage.

Dilution buffer: Under argon atmosphere and at 20–25 °C, to an Eppendorf tube (1.5 mL) 100 µL of NaPi

buffer (pH 8.0, c = 0.50 M, final concentration c = 0.25 M), 50 µL of UHQ-water, and 50 µL of the cosolvent DMA (final volume percentage 25%) were added. The solution was vortexed for 5 min and stored at ambient temperature for further usage.

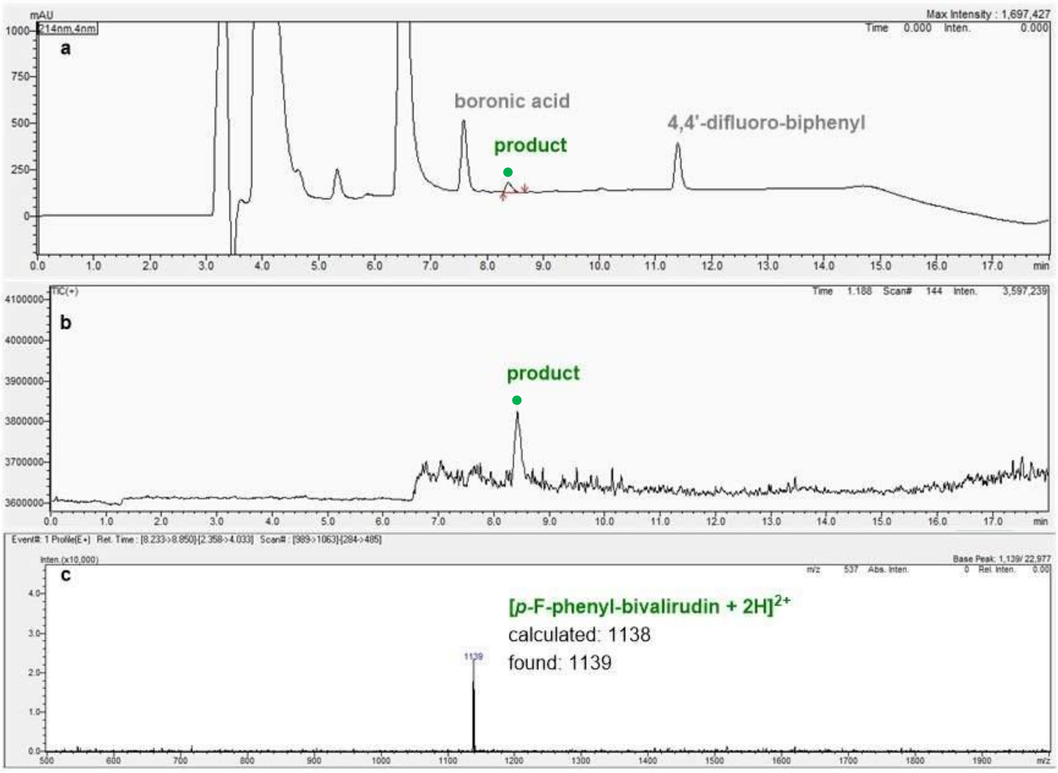

Figure 58. LC-MS analysis (Method A) of *p*-F-phenyl-bivalirudin **25** for yield determination. a) LC chromatogram (PDA 214 nm) and the peak used for integration b) total ion chromatogram c) Product peak ion series.

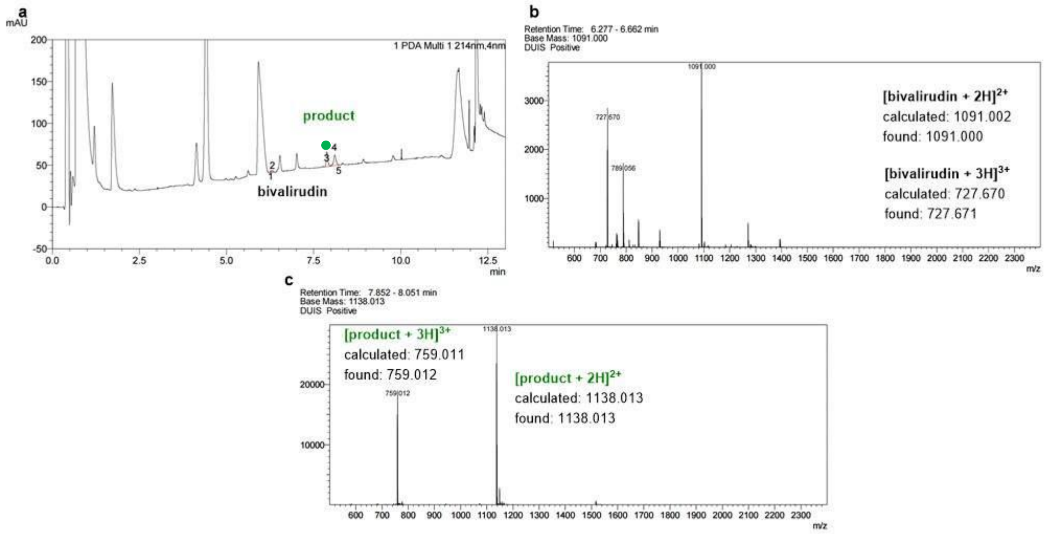

Figure 59. LC/Q-TOF-MS analysis (Method B) of *p*-F-phenyl-bivalirudin **25** for characterization. a) LC chromatogram (PDA 214 nm) b) Peak 1 and 2 (bivalirudin) ion series c) Peak 3 (product, *p*-F-phenyl-bivalirudin) ion series.

LC/Q-TOF-MS/MS results of peak 3 (*p*-F-phenyl-bivalirudin 25)

b^+	b^{2+}		Sequence		y^+	y^{2+}
148.0757	74.5415	**1**	F	**20**	2274.0150	1137.5112

						**1137.5113 (–0.1)**
245.1285<br>**245.1285 (0)**	123.0679	**2**	P	**19**	2126.9466	1063.9770
401.2296<br>**401.2299 (–0.7)**	201.1185	**3**	R	**18**	2029.8938	1015.4506
498.2824<br>**498.2822 (0.4)**	249.6449	**4**	P	**17**	1873.7927<br>**1873.7920 (0.4)**	937.4000
555.3038<br>**555.3041 (–0.5)**	278.1556	**5**	G	**16**	1776.7400	888.8736
612.3253<br>**612.3263 (–1.6)**	306.6663	**6**	G	**15**	1719.7185	860.3629
669.3468<br>**669.3465 (0.4)**	335.1770	**7**	G	**14**	1662.6970	831.8522
726.3682<br>**726.3664 (2.5)**	363.6878	**8**	G	**13**	1605.6756	803.3414
840.4112<br>**840.4111 (0.1)**	420.7092	**9**	N	**12**	1548.6541	774.8307
897.4326<br>**897.4322 (0.4)**	449.2200	**10**	G	**11**	1434.6112<br>**1434.6110 (0.1)**	717.8092
1012.4596<br>**1012.4601 (–0.5)**	506.7334<br>**506.7331 (0.6)**	**11**	D	**10**	1377.5897	689.2985
1159.5280<br>**1159.5279 (0.1)**	580.2677<br>**580.2638 (6.7)**	**12**	F	**9**	1262.5628<br>**1262.5636 (–0.6)**	631.7850
1288.5706<br>**1288.5713 (–0.5)**	644.7889<br>**644.7910 (–3.3)**	**13**	E	**8**	1115.4944<br>**1115.4997 (–4.8)**	558.2508
1417.6132<br>**1417.6127 (0.4)**	709.3102<br>**709.3103 (–0.1)**	**14**	E	**7**	986.4518<br>**986.4513 (0.5)**	493.7295
1530.6972<br>**1530.6969 (0.2)**	765.8523<br>**765.8524 (–0.1)**	**15**	I	**6**	857.4092<br>**857.4097 (–0.6)**	429.2082
1627.7500	814.3787<br>**814.3799 (–1.5)**	**16**	P	**5**	744.3251<br>**744.3252 (–0.1)**	372.6662
1756.7926<br>**1756.7957 (–1.8)**	878.9000<br>**878.8994 (0.7)**	**17**	E	**4**	647.2723<br>**647.2719 (0.6)**	324.1398
1885.8352<br>**1885.8365 (–0.7)**	943.4213<br>**943.4216 (–0.3)**	**18**	E	**3**	518.2297<br>**518.2296 (0.2)**	259.6185
2142.9204	1071.9639<br>**1071.9639 (0)**	**19**	**Y + *p*-F-phenyl**	**2**	389.1872<br>**389.1876 (–1.0)**	195.0972
2256.0044	1128.5059<br>**1128.5053 (0.5)**	**20**	L	**1**	132.1017<br>**132.1019 (–1.5)**	66.5546

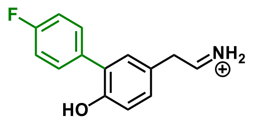

Immonium ion

m/z calculated: 230.0976

m/z found: 230.0976

Table 33. LC/Q-TOF-MS/MS analysis of **25**. Sample was directly measured without any operation. Peptide concentration for analytical sample: 0.052 mg/mL. b^{n+}, peptide fragment starting from the N-terminus of the analyte with n positive charges; y^{n+}, peptide fragment starting from the C-terminus of the analyte with n positive charges; the integer, residue numbers in the corresponding fragments, integer in blue or orange indicates the corresponding b or y fragment was found, respectively; the number with 4 decimals in black, theoretical m/z numbers for the corresponding fragment; the bold numbers with 4 decimals in blue or orange, found m/z numbers for the corresponding b or y fragment, respectively; the number in bracket, the deviation of the theoretical m/z number from the found m/z number in ppm.

Preparation of iodo-insulin 26

insulin-selenonium conjugate 11, 16 µM

mixture: mono-/double-modification = 2.6/1
Y26/Y14 = 1.4/1

NaI (0.10 M)
*i*Pr-I (0.10 M)
→
TFA (50 mM)
MeCN (40% v/v), 0 °C
390 nm LED (60 W), 20 min

26, iodo-insulin

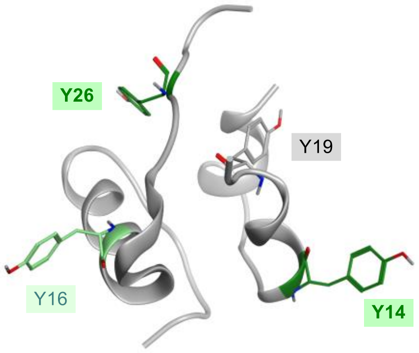

64%
mono-/double-modification = 3.6/1
Y26/Y14/Y16 =3.6/1/1

Under argon atmosphere and at 20–25 °C, 12.5 µL of the insulin-selenonium conjugate **11** solution (**11**: c = 62 µM, 0.78 nmol, 4.9 µg, 1.0 equiv. final concentration c = 16 µM; insulin: 23 µM, 0.29 nmol, 1.7 µg, 0.37 equiv. final concentration c = 6.0 µM) in aqueous solution of TFA (c = 0.10 M), 12.5 µL of a TFA stock solution (c = 0.10 M, final concentration c = 50 mM), 5 µL of a NaI stock solution (c = 1.0 M, 5.0 µmol, 0.75 mg, 6.4 × 10^3 equiv., final concentration c = 0.10 M) in UHQ-H_2O, 15 µL of the cosolvent MeCN (final volume percentage 40%), and 5 µL of an isopropyl iodide stock solution (c = 1.0 M, 5.0 µmol, 0.85 mg, 6.4 × 10^3 equiv., final concentration c = 0.10 M) in MeCN were added to an Eppendorf tube (1.5 mL). The mixture was vortexed for 5 sec., transferred into a Thermocycler pre-heated at 25 °C and incubated at 25 °C for 5 min at 600 rpm. Next, the Eppendorf tube was floated on ice-water bath and the ice-water bath was placed under 1 Kessil PR160-390 nm LED (2.5 cm away from the LED) and irradiated for 20 min (see the "Photoreaction set-up" section). The temperature of the reaction mixture was kept at approximately 0 °C through the use of the ice-water bath. Subsequently, 5.0 µL of a sodium ascorbate stock solution (c = 0.10 M, 0.50 µmol, 99 µg, 6.4 × 10^2 equiv.) in UHQ-H_2O was introduced under argon atmosphere. The mixture was vortexed for 5 sec., transferred into a Thermocycler pre-heated at 25 °C, and incubated at 25 °C for 20 min at 600 rpm. The mixture was washed with ethyl acetate (40 µL × 2) to remove excessive isopropyl iodide. The aqueous layer was diluted with 20 µL of UHQ-H_2O and was gently purged by argon 2–4 mm above the solution via a needle (Φ 0.80 × 40 mm) for 90 sec. to remove residual organic solvent. The solution volume was adjusted to 50 µL by the addition of UHQ-H_2O (ca. 10 µL). The resulting mixture was used for LC-MS analysis for yield determination as well as HRMS and LC/Q-TOF-MS/MS analysis for product characterization and site-selectivity determination. The mixture was stored at –78 °C after flash-frozen in liquid nitrogen.

HRMS ESI-pos (m/z, most abundant mass)

Insulin: calc'd for $C_{257}H_{383}N_{65}O_{77}S_6$ [M], 5806.64; deconvoluted, 5806.63. Deviation: 1.7 ppm.

Mono-I-insulin: calc'd for $C_{257}H_{382}N_{65}O_{77}S_6I$ [M], 5932.56; deconvoluted, 5932.53. Deviation: 5.1 ppm

Di-I-insulin: calc'd for $C_{257}H_{381}N_{65}O_{77}S_6I_2$ [M], 6058.44; deconvoluted, 6058.42. Deviation: 3.3 ppm

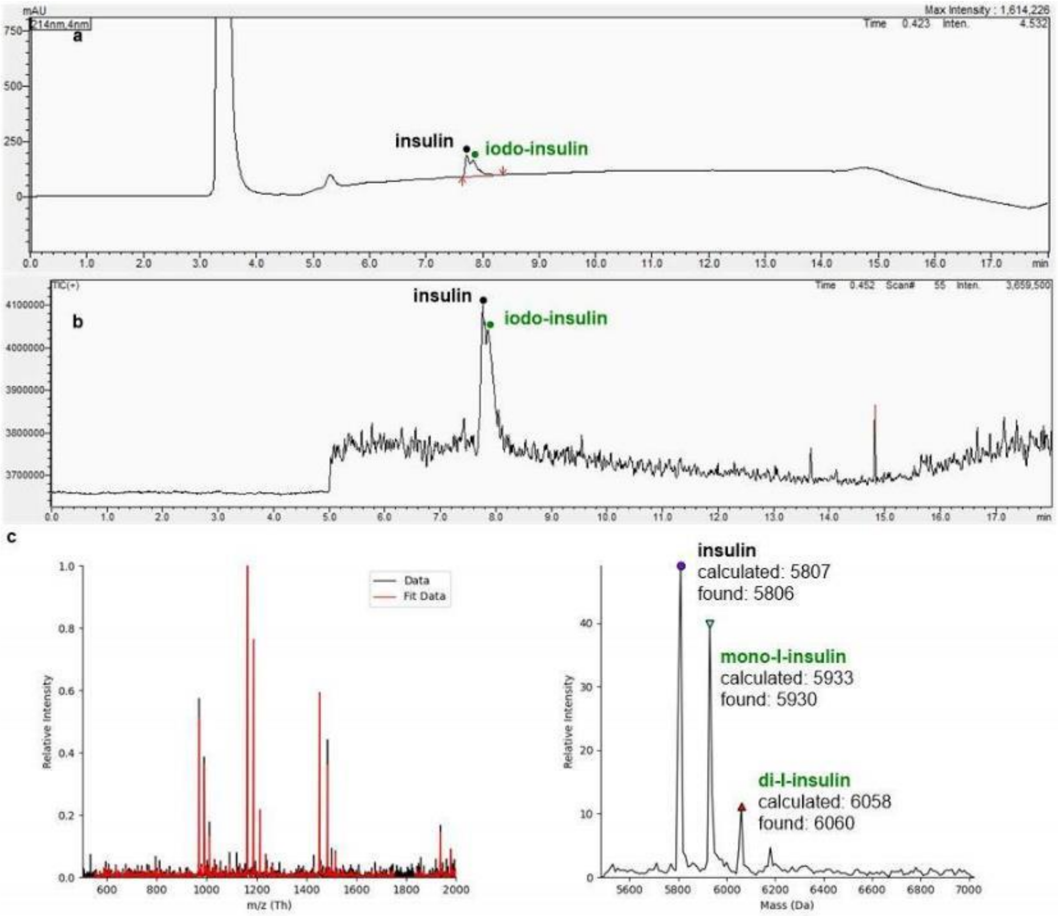

Figure 60. LC-MS analysis (Method A) of iodo-insulin mixture **26**. a) LC chromatogram (PDA 328 nm) b) total ion chromatogram c) Ion series (with Unidec fitting) and the deconvoluted spectrum. Black spots, insulin; green spots, iodo-insulin mixtures (mono- and double-modification).

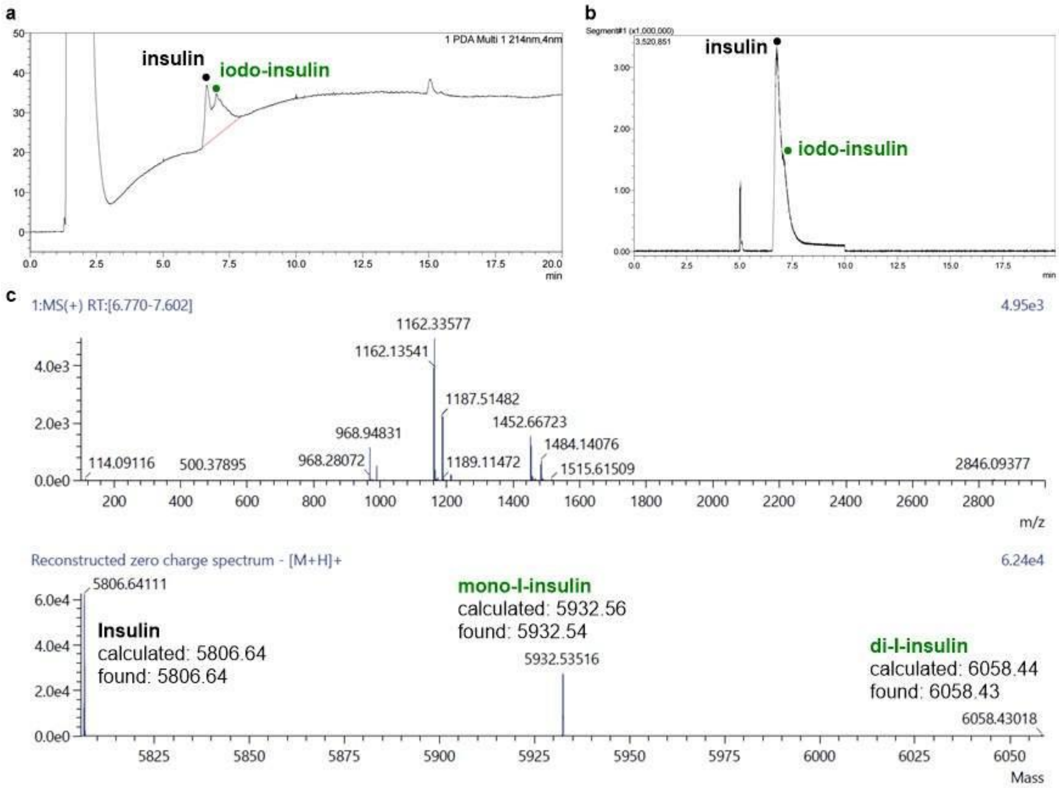

Figure 61. LC/Q-TOF-MS analysis (Method C) of iodo-insulin mixture **26** for characterization. a) LC chromatogram (PDA 214 nm) b) Total ion chromatogram (TIC) c) Ion series and deconvoluted spectrum

of the whole peak in TIC. Black spots, insulin; green spots, iodo-insulin mixtures (mono- and double-modification).

Note: The zero-charge deconvoluted spectrum of the LC/Q-TOF-MS was unsuitable for quantification because of the significant deviation of the coversion based on the zero-charge deconvolution spectrum from that estimated from LC chromatogram (PDA 214 nm). In the LC chromatogram of Fig. 60 and Fig. 61, the integral area of the products was almost identical or even slightly higher than that of unmodified insulin. Assuming that the single-atom modification on aromatic ring has little effects on the peptidyl group absorption of the modified protein at 214 nm, the amount of product can be estimated to be close to the amount of unmodified insulin. The conversion based on the zero-charge deconvoluted spectrum of the LC-MS matches the conversion estimated from the PDA analysis at 214 nm (56:44, iodinated insulins:insulin, Fig. 60) and was thus used for yield determination. However, the conversion based on the spectrum of LC/Q-TOF-MS was much lower (31:69, iodinated insulins:insulin, Fig. 61) compared to the conversion estimated from PDA analysis. A possible reason for the deviation could be the higher sensitivity of the LC/Q-TOF-MS detector to unmodified insulin than iodinated insulins.

Yield determination

Due to the difficulties in the separation of insulin from the products, the yield was determined by both PDA analysis at 214 nm and peak areas obtained from the zero-charge spectra after deconvolution of LC-MS. Based on the equation described in the section of "Quantification for transformations of *Se*-modified bivalirudin and proteins" , the recovery of total protein (83%), the conversion of mono- and di-iodo-insulin (44% and 12%, respectively), and the yield of mono- and di-iodo-insulin from *Se*-insulin **11** (50% and 14%, respectively) were determined. Therefore, the yield of iodinated insulins was 64%, with the ratio of mono-/double-iodination 3.6 (all results are an average of the results of three experiments).

Site-selectivity determination

Sample preparation for LC/Q-TOF-MS/MS analysis Under argon atmosphere and at 20–25 °C, 25 µL of the iodo-insulin **26** product mixture (for total protein: c = 15 µM, 0.38 nmol, 2.2 µg, 1.0 equiv.) in the aqueous solution of TFA (c = 50 mM) containing NaI (cac = 0.10 M) and sodium ascorbate (cac = 10 mM), 3.5 µL of UHQ-H_2O, and 7.5 µL of NH_4HCO_3 buffer (pH 9.0, c = 500 mM, final concentration c = 100 mM), and 5.0 µL of a DTT stock solution (c = 0.20 M, 1.0 µmol, 0.15 mg, 2.6 × 10^3 equiv., final concentration c = 24 mM) in UHQ-H_2O were added to an Eppendorf tube (1.5 mL). The mixture was vortexed for 5 sec. and 1.5 µL of the trypsin (0.2 mg/mL, 0.3 µg) was added to fragment the protein. Then the mixture was transferred into a Thermocycler pre-heated at 37 °C and incubated at 37 °C for 3 h at 600 rpm. The resulting mixture was analyzed by LC/QTOF-MS/MS to determine the site of modification.

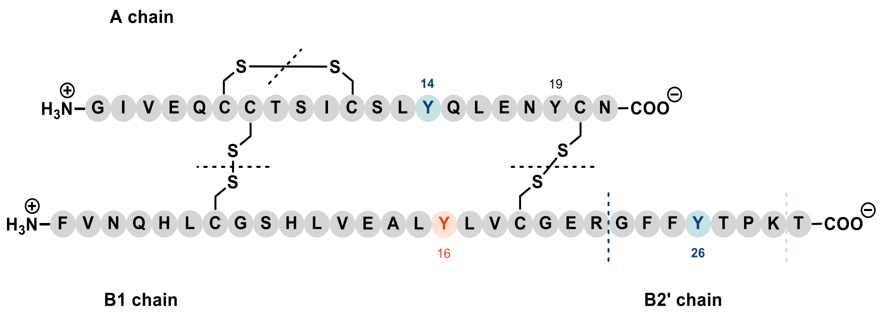

Figure 62. Fragmentation of iodo-insulin **26**. Black dashed lines, disulfide bond broken by DTT reduction;

blue dashed line, peptide bond cleaved by trypsin; pale blue dashed line, the trypsin cleavage site not cleaved due to the short incubation time; blue Y, iodinated tyrosine residues converted from *Se*-modified tyrosine residues; orange Y, iodinated tyrosine residues converted from unmodified tyrosine residues.

The results suggested single-site iodination on A chain, B1 chain, and B2' chain (Fig. 63). The modification sites were located at Y14 of A chain, Y16 of B1 chain, and Y4 of B2' chain (Y26 of B chain) confirmed by MS/MS analysis (Tables 34–37). Considering that the iodinated B1 chain and the iodinated B2' chain each contain one iodo-tyrosine residue and the iodinated A chain contains one iodo-tyrosine residue and one unmodified tyrosine residue, the ratio of modification on different tyrosine residues was determined to be 3.6/1/1 (Y26/Y14/Y16) based on the post-run PDA analysis at 280 nm, following the relationship that the extinction coefficient at 280 nm of tyrosine is half of that of 3-iodo-tyrosine[22].

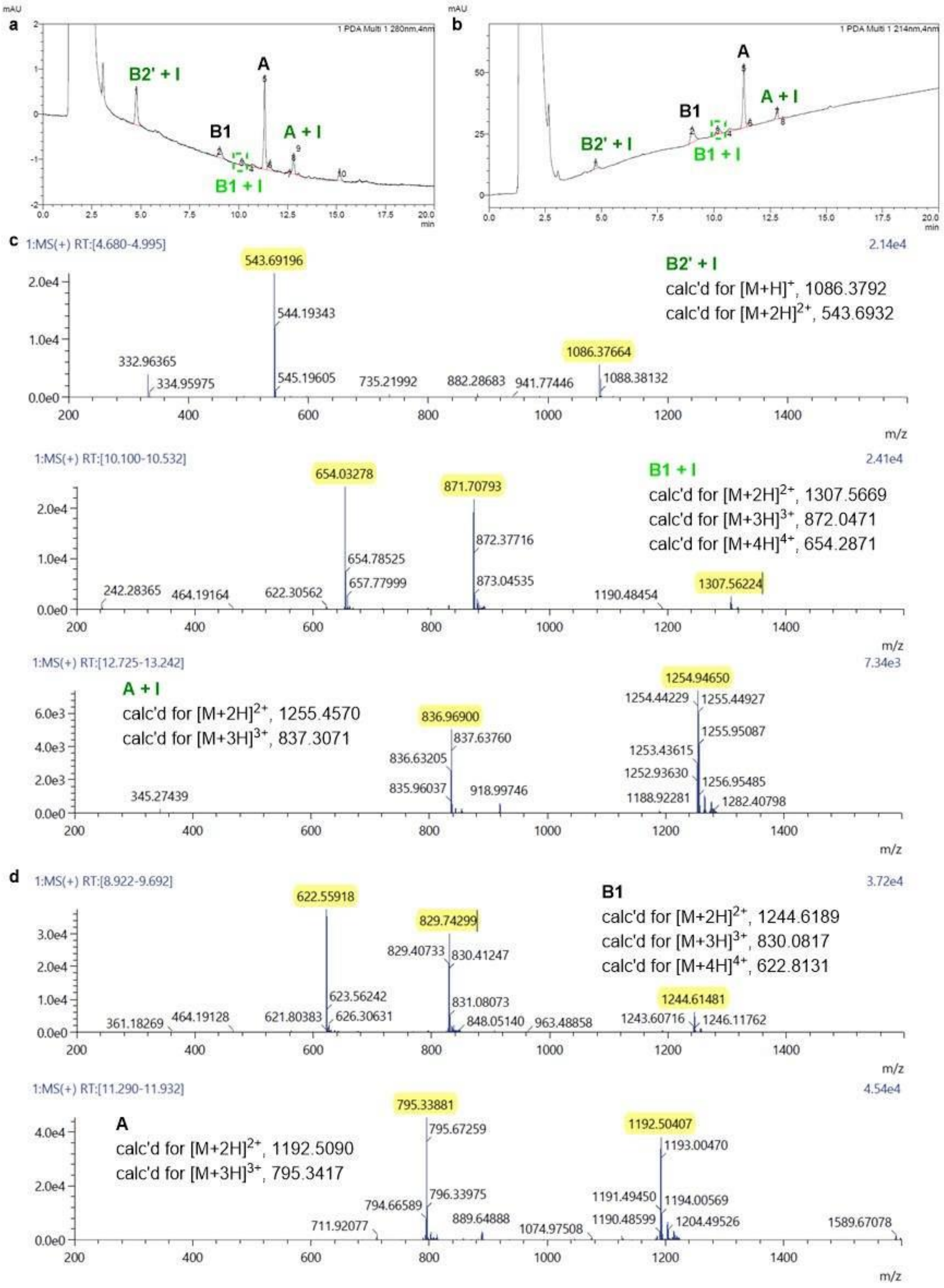

Figure 63. LC-MS analysis (Method D) of digested iodo-insulin **26** product mixture. a) LC chromatogram (PDA 280 nm) b) LC chromatogram (PDA 214 nm) c) ion series of iodinated B2', B1 and A chain d) ion series of unmodified B1 and A chain.

MS/MS result of B2'-I chain:

b^+	b^{2+}		Sequence		y^+	y^{2+}
58.0288	29.5181	**1**	G	**8**	1086.3792	543.6933
205.0972<br>**205.0965 (3.4)**	103.0523	**2**	F	**7**	1029.3578	515.1825
352.1656<br>**352.1646 (2.8)**	176.5865	**3**	F	**6**	882.2894<br>**882.2867 (3.1)**	441.6483
641.1256<br>**641.1245 (1.7)**	321.0665	**4**	**Y + I**	**5**	735.2209<br>**735.2187 (3.0)**	368.1141
742.1733<br>**742.1710 (3.1)**	371.5903	**5**	T	**4**	446.2610<br>**446.2596 (3.1)**	223.6342
839.2260	420.1167	**6**	P	**3**	345.2133<br>**345.2122 (3.2)**	173.1103
967.3210	484.1642	**7**	K	**2**	248.1605<br>**248.1603 (0.8)**	124.5839
1068.3687	534.6880	**8**	T	**1**	120.0656<br>**120.0653 (2.5)**	60.5364

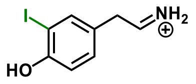

Immonium ion

m/z calculated: 261.9723

m/z found: 261.9716

Table 34. LC/QTOF-MS/MS analysis of B2'-I peptide. Total peptide concentration for analytical sample: 0.052 mg/mL. b^{n+}, peptide fragment starting from the N-terminus of the analyte with n positive charges; y^{n+}, peptide fragment starting from the C-terminus of the analyte with n positive charges; the integer, residue numbers in the corresponding fragments, integer in blue or orange indicates the corresponding b or y fragment was found, respectively; the number with 4 decimals in black, theoretical m/z numbers for the corresponding fragment; the bold numbers with 4 decimals in blue or orange, found m/z numbers for the corresponding b or y fragment, respectively; the number in bracket, the deviation of the theoretical m/z number from the found m/z number in ppm.

MS/MS result of B1-I chain:

b^+	b^{2+}		Sequence		y^+	y^{2+}
148.0757	74.5415	**1**	F	**22**	2613.1238	1307.0656
247.1442<br>**247.1435 (2.8)**	124.0757	**2**	V	**21**	2466.0554	1233.5314
361.1871	181.0972	**3**	N	**20**	2366.9870	1183.9972<br>**1183.9889 (7.0)**
489.2457<br>**489.2435 (4.5)**	245.1265	**4**	Q	**19**	2252.9441	1126.9757
626.3046<br>**626.3025 (3.4)**	313.6559	**5**	H	**18**	2124.8855	1062.9464<br>**1062.9397 (6.3)**
739.3886<br>**739.3857 (3.9)**	370.1980	**6**	L	**17**	1987.8266	994.4170<br>**994.4125 (4.5)**
842.3978<br>**842.3926 (6.2)**	421.7026	**7**	C	**16**	1874.7425	937.8749<br>**937.8692 (6.1)**
899.4193<br>**899.4162 (3.4)**	450.2133	**8**	G	**15**	1771.7333	886.3703

986.4513<br>**986.4485 (2.8)**	493.7293	**9**	S	**14**	1714.7119	857.8596
1123.5102<br>**1123.5071 (2.8)**	562.2588<br>**562.2571 (3.0)**	**10**	H	**13**	1627.6798<br>**1627.6832 (–2.1)**	814.3436<br>**814.3417 (2.3)**
1236.5943<br>**1236.5911 (2.6)**	618.8008<br>**618.7988 (3.2)**	**11**	L	**12**	1490.6209<br>**1490.6181 (1.9)**	745.8141
1335.6627<br>**1335.6602 (1.9)**	668.3350<br>**668.3321 (4.3)**	**12**	V	**11**	1377.5369<br>**1377.5331 (2.8)**	689.2721
1464.7053	732.8563<br>**732.8533 (4.1)**	**13**	E	**10**	1278.4685<br>**1278.4646 (3.1)**	639.7379
1535.7424	768.3749<br>**768.3726 (3.0)**	**14**	A	**9**	1149.4259<br>**1149.4220 (3.4)**	575.21656
1648.8265	824.9169<br>**824.9141 (3.4)**	**15**	L	**8**	1078.3888<br>**1078.3858 (2.8)**	539.6980
1937.7864	969.3969<br>**969.3934 (3.6)**	**16**	**Y + I**	**7**	965.3047<br>**965.3016 (3.2)**	483.1560
2050.8705	1025.9389<br>**1025.9364 (2.4)**	**17**	L	**6**	676.3447<br>**676.3427 (3.0)**	338.6760
2149.9389	1075.4731<br>**1075.4697 (3.2)**	**18**	V	**5**	563.2607<br>**563.2588 (3.4)**	282.1340
2252.9481	1126.9777	**19**	C	**4**	464.1922<br>**464.1915 (1.5)**	232.5998
2309.9696	1155.4884<br>**1155.4877 (0.6)**	**20**	G	**3**	361.1831<br>**361.1835 (–1.1)**	181.0952
2439.0122	1220.0097<br>**1220.0006 (7.5)**	**21**	E	**2**	304.1616<br>**304.1607 (3.0)**	152.5845
2595.1133	1298.0603	**22**	R	**1**	175.1190<br>**175.1186 (2.3)**	88.0632

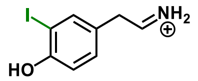

Immonium ion

m/z calculated: 261.9723

m/z found: 261.9716

Table 35. LC/QTOF-MS/MS analysis of B1-I peptide. Total peptide concentration for analytical sample: 0.052 mg/mL. b^{n+}, peptide fragment starting from the N-terminus of the analyte with n positive charges; y^{n+}, peptide fragment starting from the C-terminus of the analyte with n positive charges; the integer, residue numbers in the corresponding fragments, integer in blue or orange indicates the corresponding b or y fragment was found, respectively; the number with 4 decimals in black, theoretical m/z numbers for the corresponding fragment; the bold numbers with 4 decimals in blue or orange, found m/z numbers for the corresponding b or y fragment, respectively; the number in bracket, the deviation of the theoretical m/z number from the found m/z number in ppm.

MS/MS result of A-I chain:

Iodination on **Y14** (matched with MS/MS data):

b^+	b^{2+}		Sequence		y^+	y^{2+}
58.0288	29.5181	**1**	G	**21**	2508.9040	1254.9557<br>**1254.9442 (9.2)**
171.1129<br>**171.1124 (2.9)**	86.0601	**2**	I	**20**	2451.8825	1226.4449
270.1813<br>**270.1805 (3.0)**	135.5943	**3**	V	**19**	2338.7985	1169.9029
399.2239	200.1156	**4**	E	**18**	2239.7300	1120.3687

**399.2224 (3.8)**					**2239.7162 (6.2)**	**1120.3657 (2.7)**
527.2824<br>**527.2815 (1.7)**	264.1449	**5**	Q	**17**	2110.6875<br>**2110.6764 (5.3)**	1055.8474
630.2916<br>**630.2892 (2.9)**	315.6495	**6**	C	**16**	1982.6289<br>**1982.6173 (5.9)**	991.8181
733.3008<br>**733.2995 (1.8)**	367.1541	**7**	C	**15**	1879.6197<br>**1879.6102 (5.1)**	940.3135
834.3485<br>**834.3482 (0.4)**	417.6779	**8**	T	**14**	1776.6105<br>**1776.6021 (4.7)**	888.8089
921.3805<br>**921.3770 (3.8)**	461.1939	**9**	S	**13**	1675.5628<br>**1675.5575 (3.2)**	838.2851
1034.4646<br>**1034.4595 (4.9)**	517.7360	**10**	I	**12**	1588.5308<br>**1588.5299 (0.6)**	794.7691
1137.4738<br>**1137.4659 (6.9)**	569.2405	**11**	C	**11**	1475.4467<br>**1475.4403 (4.3)**	738.2270
1224.5058<br>**1224.5036 (1.8)**	612.7566	**12**	S	**10**	1372.4376<br>**1372.4343 (2.4)**	686.7224
1337.5899<br>**1337.5832 (5.0)**	669.2986	**13**	L	**9**	1285.4055<br>**1285.4024 (2.4)**	643.2064
1626.5498<br>**1626.5431 (4.1)**	813.7786	**14**	**Y + I**	**8**	1172.3215<br>**1172.3182 (2.8)**	586.6644
1754.6084<br>**1754.6011 (4.2)**	877.8079	**15**	Q	**7**	883.3615<br>**883.3592 (2.6)**	442.1844
1867.6925<br>**1867.6822 (5.5)**	934.3499<br>**934.3519 (–2.1)**	**16**	L	**6**	755.3029<br>**755.3011 (2.4)**	378.1551
1996.7351<br>**1996.7236 (5.8)**	998.8712	**17**	E	**5**	642.2189<br>**642.2178 (1.7)**	321.6131
2110.7780<br>**2110.7661 (5.6)**	1055.8927<br>**1055.8873 (5.1)**	**18**	N	**4**	513.1763<br>**513.1760 (0.6)**	257.0918
2273.8413<br>**2273.8525 (–4.9)**	1137.4243<br>**1137.4231 (1.1)**	**19**	Y	**3**	399.1333<br>**399.1327 (1.5)**	200.0703
2376.8505	1188.9289<br>**1188.9222 (5.6)**	**20**	C	**2**	236.0700<br>**236.0694 (2.5)**	118.5387
2490.8934	1245.9504	**21**	N	**1**	133.0608<br>**133.0606 (1.5)**	67.0341

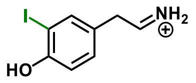

Immonium ion

m/z calculated: 261.9723

m/z found: 261.9719

Table 36. LC/QTOF-MS/MS analysis of A-I peptide. Table presents the MS signals calculated based on Y14 iodination. Total peptide concentration for analytical sample: 0.052 mg/mL. b^{n+}, peptide fragment starting from the N-terminus of the analyte with n positive charges; y^{n+}, peptide fragment starting from the C-terminus of the analyte with n positive charges; the integer, residue numbers in the corresponding fragments, integer in blue or orange indicates the corresponding b or y fragment was found, respectively; the number with 4 decimals in black, theoretical m/z numbers for the corresponding fragment; the bold numbers with 4 decimals in blue or orange, found m/z numbers for the corresponding b or y fragment, respectively; the number in bracket, the deviation of the theoretical m/z number from the found m/z number in ppm.

Iodination on **Y19** (not supported by experimental data):

b^+	b^{2+}		Sequence	y^+	y^{2+}
1500.6532	750.8303	**14**	Y		

1628.7118	814.8595	**15**	Q	**7**	1009.2581	505.1327
1741.7958	871.4016	**16**	L	**6**	881.1996	441.1034
1870.8384	935.9229	**17**	E	**5**	768.1155	384.5614
1984.8813	992.9443	**18**	N	**4**	639.0729	320.0401
			**Y + I**	**3**	525.0300	263.0186

Table 37. LC/QTOF-MS/MS analysis of A-I peptide. Table presents the different MS/MS signals of Y19 modification compared to those of Y14 modification. b^{n+}, peptide fragment starting from the N-terminus of the analyte with n positive charges; y^{n+}, peptide fragment starting from the C-terminus of the analyte with n positive charges; the integer, residue numbers in the corresponding fragments, integer in blue or orange indicates the corresponding b or y fragment was found, respectively; the number with 4 decimals in black, theoretical m/z numbers for the corresponding fragment; the bold numbers with 4 decimals in blue or orange, found m/z numbers for the corresponding b or y fragment, respectively; the number in bracket, the deviation of the theoretical m/z number from the found m/z number in ppm.

Control experiment

insulin, 22 µM → **insulin** + **26, iodo-insulin**

Above arrow: tyrosine-selenonium salt **15** (31 µM); NaI (0.10 M); *i*Pr-I (0.10 M)
Below arrow: TFA (50 mM); MeCN (40% v/v), 0 °C, 390 nm LED (60 W), 20 min

86% recovery **0%**

To investigate whether the iodo-tyrosine residues in iodo-insulins can originate from the side reaction between unmodified tyrosine residues and the iodine formed during the photoreaction (see Supplementary Information 69), a control experiment using unmodified insulin and excessive amount of tyrosine-selenonium salt **15** was conducted. Under ambient atmosphere and at 20–25 °C, 12.5 µL of the insulin stock solution (c = 88 µM, 1.1 nmol, 6.4 µg, 1.0 equiv. final concentration c = 22 µM) in aqueous solution of TFA (c = 0.10 M), 2.5 µL of a stock solution of **15** (c = 0.62 mM, 1.6 nmol, 1.1 µg, 1.4 equiv., final concentration c = 31 µM) in aqueous solution of TFA (c = 0.10 M), 10 µL of a TFA stock solution (c = 0.10 M, final concentration c = 50 mM), 5 µL of a NaI stock solution (c = 1.0 M, 5.0 µmol, 0.75 mg, final concentration c = 0.10 M) in UHQ-H_2O, 15 µL of the cosolvent MeCN (final volume percentage 40%), and 5 µL of an isopropyl iodide stock solution (c = 1.0 M, 5.0 µmol, 0.85 mg, final concentration c = 0.10 M) in MeCN were added to an Eppendorf tube (1.5 mL). The mixture was degassed by gently purging argon 2–4 mm above the solution via a needle (Φ 0.80 × 40 mm) for 10 sec.. Then the cap was closed and the mixture was vortexed for 5 sec., transferred into a Thermocycler pre-heated at 25 °C and incubated at 25 °C for 5 min at 600 rpm. Next, the Eppendorf tube was floated on ice-water bath and the ice-water bath was placed under 1 Kessil PR160-390 nm LED (2.5 cm away from the LED) and irradiated for 20 min (see the “Photoreaction set-up” section). The temperature of the reaction mixture was kept at approximately 0 °C through the use of the ice-water bath. Subsequently, 5.0 µL of a sodium ascorbate stock solution (c = 0.10 M, 0.50 µmol, 99 µg, 6.4 × 10^2 equiv.) in UHQ-H_2O was introduced. The mixture was vortexed for 5 sec., transferred into a Thermocycler pre-heated at 25 °C, and incubated at 25 °C for 20 min at 600 rpm. The mixture was washed with ethyl acetate (40 µL × 2) to remove excessive isopropyl iodide. The aqueous layer was diluted by 20 µL of UHQ-H_2O and was gently purged by argon 2–4 mm above the solution via a needle (Φ 0.80 × 40 mm) for 90 sec. to remove residual organic solvent. The solution volume was adjusted to 50 µL by the addition of UHQ-H_2O (ca. 10 µL). The resulting mixture was directly analyzed by LC-MS analysis and the recovery of ubiquitin was determined based on the post-run PDA analysis at 214 nm (86%, in triplicate). Iodo-insulin was not observed either in the PDA analysis at 214 nm or in the TIC spectrum.

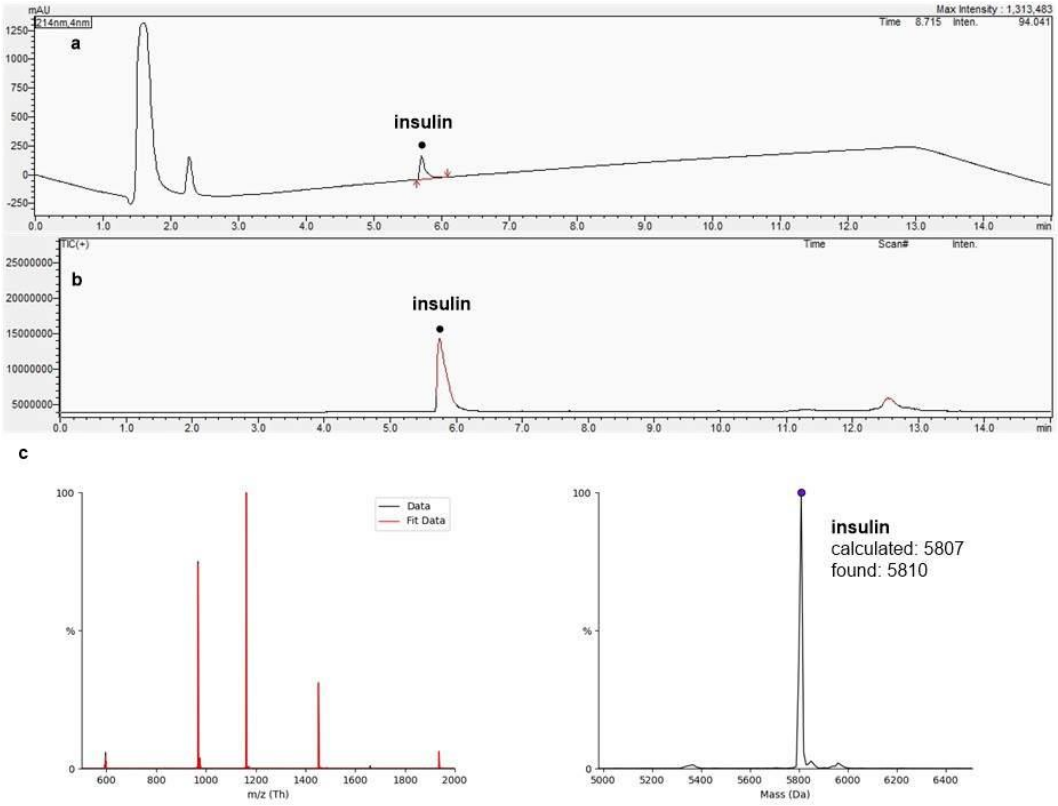

Figure 64. LC-MS analysis (Method A) of the control experiment. a) LC chromatogram (PDA 214 nm) and the peak of insulin. b) Total ion chromatogram c) Ion series (with Unidec fitting) and the deconvoluted spectrum.

Preparation of bromo-ubiquitin 27

Preparation of bromo-ubiquitin 27 on a small scale

ubiquitin-selenonium conjugate 12, 5.6 µM → **27, bromo-ubiquitin**

Above the arrow: $Cu(MeCN)_4PF_6$ (50 mM), $CuBr_2$ (0.10 M), KBr (0.40 M)

Below the arrow: NaPi buffer (pH 3.0, 10 mM), MeCN (40% v/v), 0°C, 390 nm LED (60W), 15 min

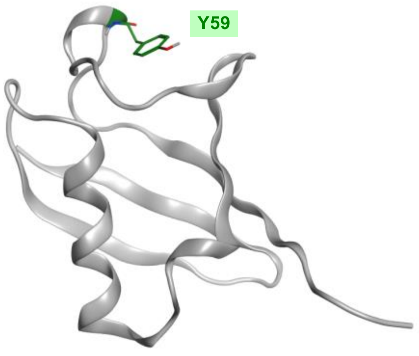

71%, Y59

Preparation of stock solutions

Stock solution A: Under ambient atmosphere, a scintillation vial (4 mL) equipped with a Teflon-coated magnetic stirring bar was charged with $CuBr_2$ (56 mg, 0.25 mmol, 1.0 equiv.), KBr (120 mg, 1.0 mmol, 4.0 equiv.), and the solvent UHQ-H_2O (1.0 mL, $CuBr_2$: c = 0.25 M; KBr: c = 1.0 M). The mixture was stirred at 25 °C until all solids dissolved. Then the vial was degassed by gently purging argon through the solution

via a needle (Φ 0.80 × 120 mm) for 5 min and stored under nitrogen atmosphere for further usage.

Stock solution B: Under ambient atmosphere, a scintillation vial (4 mL) equipped with a Teflon-coated magnetic stirring bar was charged with $CuBr_2$ (110 mg, 0.50 mmol, 1.0 equiv.), KBr (240 mg, 2.0 mmol, 4.0 equiv.), and the solvent UHQ-H_2O (1.0 mL, $CuBr_2$: c = 0.50 M; KBr: c = 2.0 M). The mixture was stirred at 25 °C until all solids dissolved. The solution was stored under ambient atmosphere for further usage.

Photoreaction set-up

Under argon atmosphere and at 20–25 °C, 20 µL of the stock solution A ($CuBr_2$: c = 0.25 M, 5.0 µmol, 1.1 mg, 1.8×10^4 equiv., final concentration c = 0.10 M; KBr: c = 1.0 M, 20 µmol, 2.4 mg, 7.1×10^4 equiv., final concentration c = 0.40 M) in UHQ-H_2O, 10 µL of the stock solution of $Cu(MeCN)_4PF_6$ (c = 0.25 M, 2.5 µmol, 0.93 mg, 8.9×10^3 equiv., final concentration c = 50 mM) in MeCN, and 10 µL of MeCN (final volume percentage 40%) were added to an Eppendorf tube (1.5 mL). The mixture was vortexed for 5 sec. followed by the addition of 10 µL of a ubiquitin-selenonium conjugate **12** reaction mixture (**12**: c = 28 µM, 0.28 nmol, 2.5 µg, 1.0 equiv. final concentration c = 5.6 µM; ubiquitin: 14 µM, 0.14 nmol, 1.2 µg, 0.50 equiv., final concentration c = 2.8 µM; both ubiquitin with methionine oxidized) in NaPi buffer (pH 3.0, c = 50 mM, final concentration c = 10 mM) containing $MgCl_2$ (c = 50 mM, final concentration c = 10 mM) and urea (c = 2.4 M, final concentration c = 0.48 M). The mixture was vortexed for 5 sec., transferred into a Thermocycler pre-heated at 25 °C, and incubated at 25 °C for 5 min at 600 rpm. Next, the Eppendorf tube was floated on ice-water bath and the ice-water bath was placed under 1 Kessil PR160-390 nm LED (2.5 cm away from the LED) and irradiated for 15 min (see the "Photoreaction set-up" section). The temperature of the reaction mixture was kept at approximately 0°C through the use of the ice-water bath. Subsequently, 30 µL of an EDTA-Na_2 stock soluition (pH 8.5, 0.3 M, 9.0 µmol, 3.4 mg, 1.2 equiv. to copper ions) in UHQ-H_2O was added to complex copper ion. The mixture was vortexed for 5 sec., transferred into a Thermocycler pre-heated at 25 °C, and incubated at 25 °C for 5 min at 600 rpm. Then, the mixture was washed with ethyl acetate (40 µL × 2) and the aqueous layer was gently purged by argon 2–4 mm above the solution via a needle (Φ 0.80 × 40 mm) for 2 min to remove residual organic solvent. The solution volume was adjusted to 50 µL by the addition of UHQ-H_2O (ca. 3–7 µL). The resulting solution was directly used for LC-MS analysis for yield determination based on the post-run PDA analysis at 214 nm and the zero-charge spectra after deconvolution of TIC (71%, total protein recovery 77%, conversion 62%, all in triplicate) as well as for LC/QTOF-MS analysis for product characterization without any further purification.

Preparation of bromo-ubiquitin 27 on a large scale (renatured) for LC-FTMS/MS analysis and CD spectrometry

In a glovebox and at 20–25 °C, 60 µL of the stock solution of $Cu(MeCN)_4PF_6$ (c = 0.125 M, 7.5 µmol, 2.8 mg, 4.4×10^3 equiv., final concentration c = 50 mM) in MeCN (final volume percentage 40%) was added to an Eppendorf tube (1.5 mL) under argon atmosphere. Then, the tube was transferred outside, followed by the addition of 30 µL of the stock solution B ($CuBr_2$: c = 0.50 M, 15 µmol, 3.3 mg, 8.8×10^3 equiv., final concentration c = 0.10 M; KBr: c = 2.0 M, 60 µmol, 7.2 mg, 3.5×10^4 equiv., final concentration c = 0.40 M) in UHQ-H_2O. The mixture was vortexed for 5 sec. followed by the addition of 60 µL of a ubiquitin-selenonium conjugate **12** reaction mixture (**12**: c = 28 µM, 1.7 nmol, 15 µg, 1.0 equiv. final concentration

c = 11 µM; ubiquitin: 13 µM, 0.78 nmol, 6.7 µg, 0.46 equiv., final concentration c = 5.2 µM; both ubiquitin with methionine oxidized) in NaPi buffer (pH 3.0, c = 50 mM, final concentration c = 20 mM) containing $MgCl_2$ (c = 50 mM, final concentration c = 20 mM) and urea (c = 2.4 M, final concentration c = 0.96 M). The mixture was degassed by gently purging argon 2–4 mm above the solution via a needle (Φ 0.80 × 40 mm) for 10 sec.. Then, the cap was closed and the mixture was vortexed for 5 sec., transferred into a Thermocycler pre-heated at 25 °C, and incubated at 25 °C for 5 min at 600 rpm. Next, the Eppendorf tube was floated on ice-water bath and the ice-water bath was placed under 1 Kessil PR160-390 nm LED (2.5 cm away from the LED) and irradiated for 15 min (see the "Photoreaction set-up" section). The temperature of the reaction mixture was kept at approximately 0°C through the use of the ice-water bath. Subsequently, 90 µL of an EDTA-Na_2 stock soluition (pH 8.5, 0.30 M, 27 µmol, 10 mg, 1.2 equiv. to copper ions) in UHQ-H_2O was added to complex copper ion. The mixture was vortexed for 5 sec., transferred into a Thermocycler pre-heated at 25 °C, and incubated at 25 °C for 20 min at 600 rpm. Next, the mixture was added to 200 µL of NaPi buffer (pH 7.4, c = 50 mM) in an Eppendorf tube (1.5 mL) dropwise to induce the refolding of the protein at 20–25 °C. The resulting solution was transferred to Amicon® Ultra Centrifugal Filters (3 kDa cutoff, 0.5 mL) and centrifuged at 14,000 ×g and at 4 °C for 15 min to remove most of Cu(EDTA) and MeCN. Subsequently, to the filter 300 µL of NaPi buffer (pH 7.4, c = 50 mM) was added and the mixture was centrifuged at 14,000 ×g and at 4 °C for 15 min again, and this step was repeated for 3 times to change the buffer in the mixture. The volume of the resulting mixture was adjusted to 90 µL by adding ca. 5–10 µL of NaPi buffer (pH 7.4, c = 50 mM). The renatured mixture was analyzed by LC-MS and the yield was determined based on the post-run PDA analysis at 214 nm and the zero-charge spectra after deconvolution of TIC (48%, total protein recovery 52%, conversion 62%). The mixture was used for the sample preparation of LC-FTMS/MS analysis and CD spectrometry without any further purification.

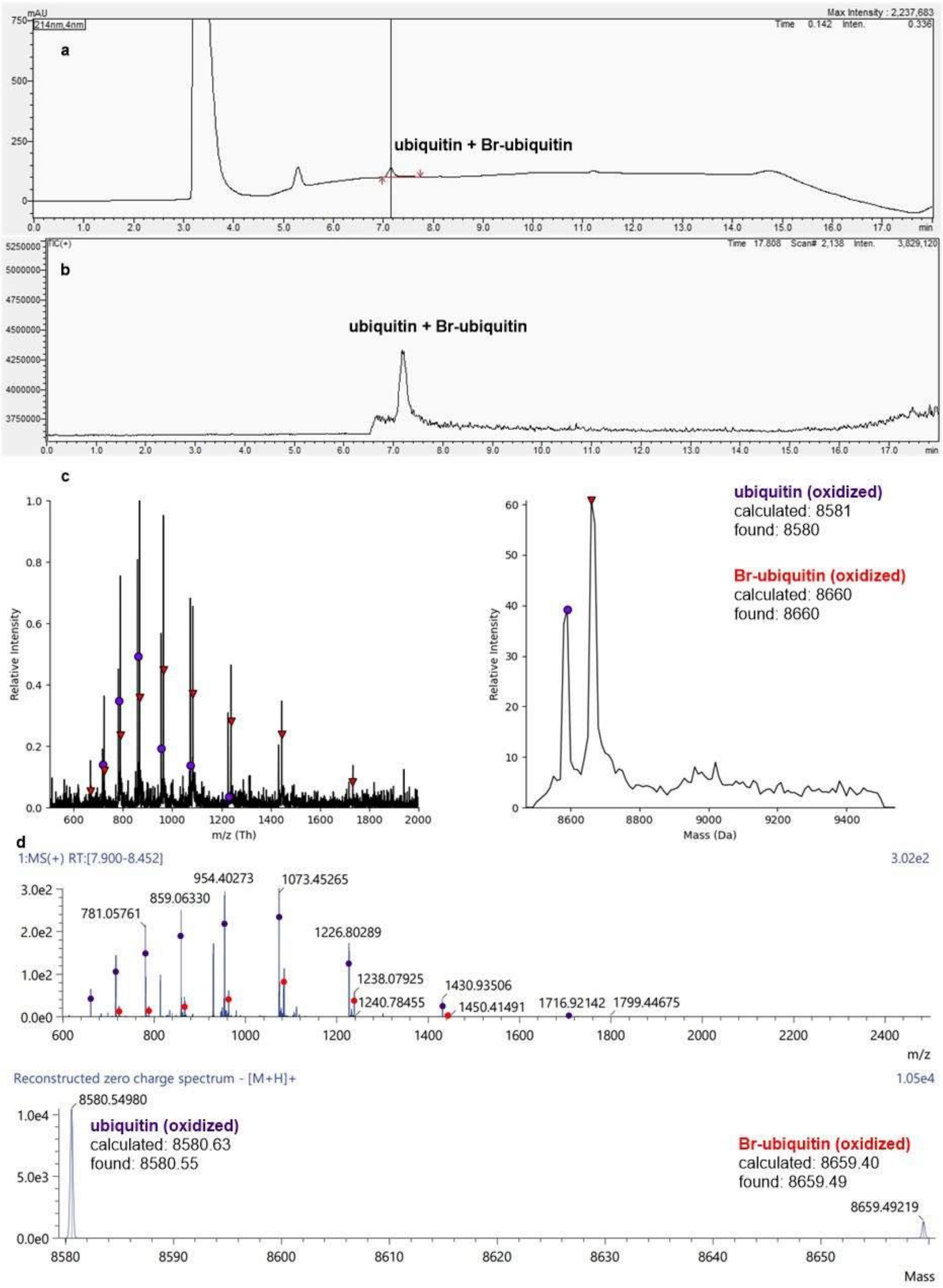

Figure 65. LC-MS analysis (Method A) and LC/Q-TOF MS analysis (Method C) of bromo-ubiquitin **27** prepared on a small scale. a) LC chromatogram (PDA 214 nm) and the peak of ubiquitin mixture b) total ion chromatogram c) Ubiquitin mixture peak ion series and deconvoluted spectrum d) Ubiquitin mixture peak ion series obtained by LC/Q-TOF MS and deconvoluted spectrum. Red spots, bromo-ubiquitin; purple spots, ubiquitin. Ubiquitin and bromo-ubiquitin contain oxidized methionine residues. LC injection volume was 5 µL. (Note: The zero-charge deconvoluted spectrum of LC/Q-TOF-MS measurement is unreliable for quantification, for details see the note of Fig. 61.)

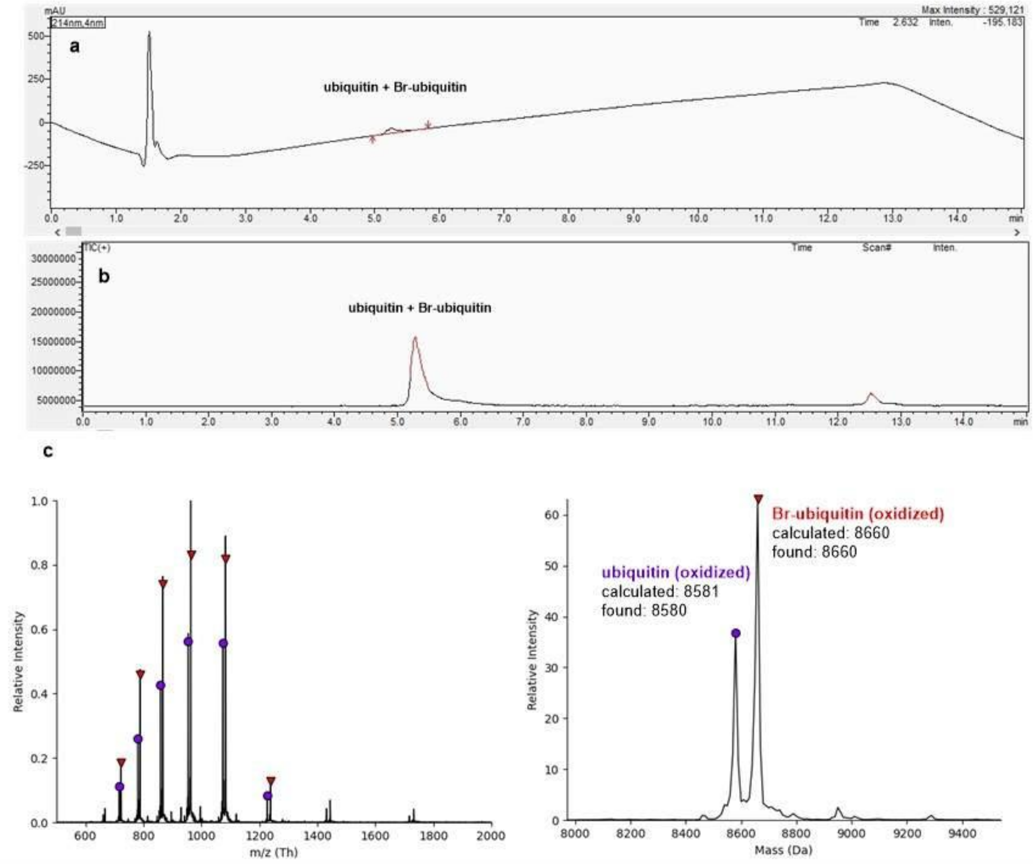

Figure 66. LC-MS analysis (Method A) of bromo-ubiquitin **27** in prepared on a large scale. a) LC chromatogram (PDA 214 nm) and the peak of ubiquitin mixture b) total ion chromatogram c) Ubiquitin mixture peak ion series and deconvoluted spectrum. Red spots,bromo-ubiquitin; purple spots, ubiquitin. Ubiquitin and bromo-ubiquitin contain oxidized methionine residues. LC injection volume was 3 µL.

Sample preparation for LC-FTMS/MS analysis

Under ambient atmosphere and at 20–25 °C, 40 µL of the ubiquitin and bromo-ubiquitin **27** reaction mixture (c = 9.0 µM, 0.35 nmol, 3 µg; for total protein, c = 14 µM, 0.56 nmol, 4.8 µg) in NaPi buffer (pH = 7.4, c = 50 mM) and 60 µL of NH_4HCO_3 buffer (pH 8.0, c = 0.10 M) were added to an Eppendorf tube (1.5 mL). The mixture was vortexed for 5 sec. and 2.0 µL of trypsin (0.2 mg/mL, 0.4 µg) to fragment the protein. The mixture was transferred into a Thermocycler pre-heated at 37 °C, and incubated at 37 °C for 20 h at 600 rpm. The resulting mixture was analyzed by LC-FTMS/MS to determine the site of modification.

MS/MS results:

b^+		Sequence		y^+
88.0394	**1**	S	**16**	1993.9342
203.0663<br>**203.0659 (2.0)**	**2**	D	**15**	1906.9021
444.0401<br>**444.0352 (11.0)**	**3**	**Y + Br**	**14**	1791.8752
558.0831<br>**558.0819 (2.2)**	**4**	N	**13**	1550.9013
671.1671	**5**	I	**12**	1436.8584
799.2257	**6**	Q	**11**	1323.7744<br>**1323.7809 (–4.9)**

927.3207	**7**	K	**10**	1195.7158<br>**1195.7109 (4.1)**
1056.3633	**8**	E	**9**	1067.6208<br>**1067.6211 (–0.3)**
1143.3953	**9**	S	**8**	938.5782<br>**938.5770 (1.3)**
1244.4430	**10**	T	**7**	851.5462<br>**851.5438 (2.8)**
1357.5270	**11**	L	**6**	750.4985<br>**750.4974 (1.5)**
1494.5859	**12**	H	**5**	637.4145
1607.6700	**13**	L	**4**	500.3555
1706.7384	**14**	V	**3**	387.2715
1819.8225	**15**	L	**2**	288.2031
1975.9236	**16**	R	**1**	175.1190

Table 38. MS/MS analysis of peptide internal fragment SDYNIQKESTLHLVLR (from TLSDYNIQKESTLHLVLR, see Supplementary Information 335–336 for details) of bromo-ubiquitin **27** after digestion. Total peptide concentration for analytical sample: 0.047 mg/mL. b^{n+}, peptide fragment starting from the N-terminus of the analyte with n positive charges; y^{n+}, peptide fragment starting from the C-terminus of the analyte with n positive charges; the integer, residue numbers in the corresponding fragments, integer in blue or orange indicates the corresponding b or y fragment was found, respectively; the number with 4 decimals in black, theoretical m/z numbers for the corresponding fragment; the bold numbers with 4 decimals in blue or orange, found m/z numbers for the corresponding b or y fragment, respectively; the number in bracket, the deviation of the theoretical m/z number from the found m/z number in ppm.

CD spectroscopy study

Samples preparation

Bromo-ubiquitin: Under ambient atmosphere and at 20–25 °C, 400 µL (a combination of the renatured product mixture from five times of the large-scale preparation of bromo-ubiquitin) of the ubiquitin and bromo-ubiquitin **27** mixture (c = 9.0 µM, 3.6 nmol, 30 µg; for total protein, c = 14 µM, 5.6 nmol, 48 µg) in NaPi buffer (pH = 7.4, c = 50 mM) was transferred to Amicon® Ultra Centrifugal Filters (3 kDa cutoff, 0.5 mL) and concentrated by centrifugation at 14,000 × g and at 4 °C for 15 min. Subsequently, 300 µL of KPi buffer (pH 7.0, c = 10 mM) was added and the mixture was centrifuged at 14,000 × g and at 4 °C for 15 min, and this step was repeated for three times to change the NaPi buffer in the bromo-ubiquitin mixture. The volume of the resulting mixture was adjusted to 400 µL by ca. 320 µL of KPi buffer (pH 7.0, c = 10 mM). The mixture was analyzed by LC-MS and the concentration of protein was determined based on the post-run PDA analysis at 214 nm and the zero-charge spectra after deconvolution of TIC (for bromo ubiquitin, c = 9.0 µM; for total protein c = 14 µM, 0.12 mg/mL) (Fig. 67).

Unmodified ubiquitin: Under ambient atmosphere and at 20–25 °C, 26 µL of a stock solution of ubiquitin (c = 0.26 mM) in KPi buffer (pH 7.0, c = 10 mM) and 474 µL of KPi buffer (pH 7.0, c = 10 mM) were added to an Eppendorf tube (1.5 mL). The resulting solution was analyzed by LC-MS for the determination of the concentration of the ubiquitin based on the post-run PDA analysis at 214 nm (13 µM, 0.11 mg/mL).

CD spectra measurement and analysis

Circular dichroism (CD) spectroscopy was performed on ubiquitin and bromo-ubiquitin using a J-1100 CD spectrometer (JASCO). Protein solutions in KPi buffer (pH 7.0, 10 mM) and were placed into a 1.0 mm

Light Path QS High Precision Cell quartz cuvette (Hellma Analytics). Measurements were done at 20 °C (in sample holder equipped with thermostat). The scans were made from 350 to 180 nm at a scan speed of 100 nm/min and 0.5 nm data pitch. The slit width was 1.0 nm. The High Tension Voltage never exceeded 700 V during measurements within the analyzed wavelength range. The buffer spectrum was subtracted from the final spectra. The spectra were recorded in five replicates, and the resulting average spectra were plotted together for comparison between the secondary structure of unmodified ubiquitin and that of bromo-ubiquitin (Fig. 68). The spectra of the reaction mixture (bromo-ubiquitin **27** and ubiquitin) and ubiquitin stock solution overlap in the 190–270 nm range, which serves as an evidence that the secondary structure of **27** was fully restored after renaturation.

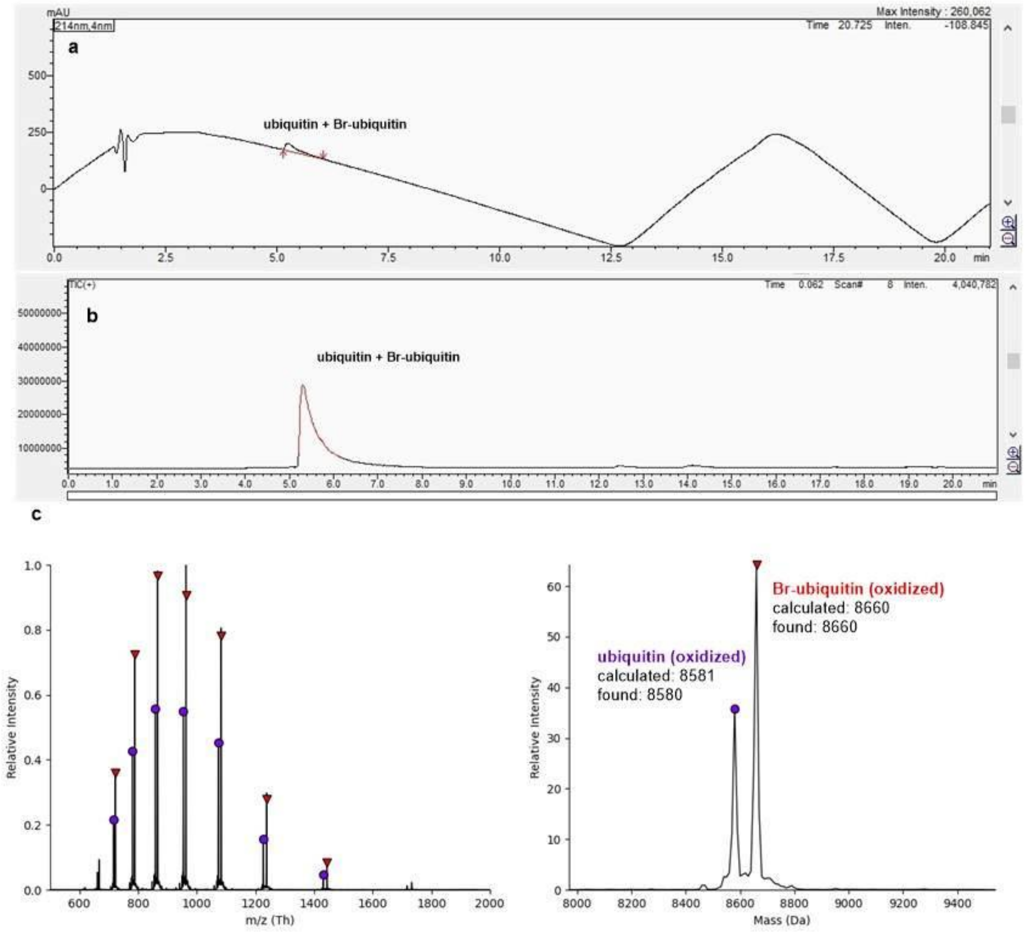

Figure 67. LC-MS analysis (Method A) of bromo-ubiquitin **27** for CD spectrum measuremant. a) LC chromatogram (PDA 214 nm) and the peak of ubiquitin mixture b) Total ion chromatogram. c) Ubiquitin mixture peak ion series and deconvoluted spectrum. Red spots, bromo-ubiquitin; purple spots, ubiquitin. Ubiquitin and bromo-ubiquitin contain oxidized methionine residues. LC injection volume was 5 µL.

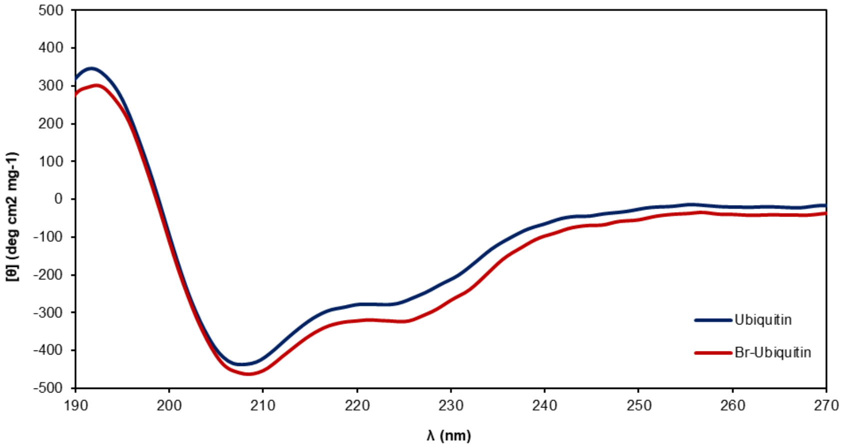

Figure 68. CD spectra of ubiquitin and bromo-ubiquitin **27** after renaturation.

Control experiment

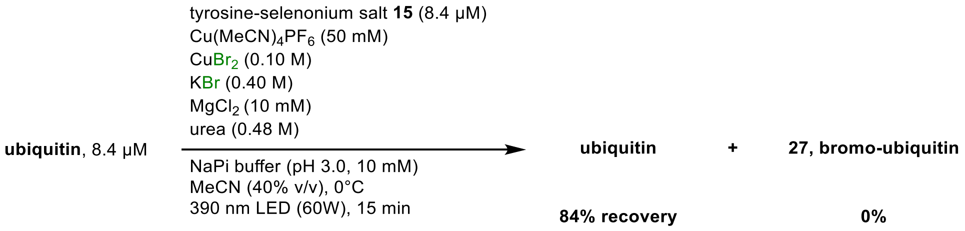

To investigate whether the bromo-tyrosine residues in bromo-ubiquitins can originate from the side reaction of unmodified tyrosine residues during the photoreaction, a control experiment using unmodified ubiquitin and tyrosine-selenonium salt **15** was conducted. In a glovebox and at 20–25 °C, 20 µL of the stock solution of $Cu(MeCN)_4PF_6$ (c = 0.125 M, 2.5 µmol, 0.93 mg, final concentration c = 50 mM) in MeCN (final volume percentage 40%) was added to an Eppendorf tube (1.5 mL) under argon atmosphere. Then, the tube was transferred outside, followed by the addition of 10 µL of the stock solution B ($CuBr_2$: c = 0.50 M, 5.0 µmol, 1.1 mg, final concentration c = 0.10 M; KBr: c = 2.0 M, 20 µmol, 2.4 mg, final concentration c = 0.40 M) in UHQ-H_2O. The mixture was vortexed for 5 sec.. Then, 1.8 µL of UHQ-H_2O, 1.2 µL of the stock solution of $MgCl_2$ (c = 0.40 M, 0.48 µmol, 46 µg, final concentration c = 10 mM) in UHQ-H_2O, 5.0 µL of the stock solution of urea (c = 4.8 M, 24 µmol, 1.4 mg, final concentration c = 0.48 M) in UHQ-H_2O were added to create the exactly same conditions used in the preparation of bromo-ubiquitin **27**. Next, 10 µL of an ubiquitin stock solution (c = 42 µM, 0.42 nmol, 3.6 µg, 1.0 equiv. final concentration c = 8.4 µM) in NaPi buffer (pH 3.0, c = 50 mM, final concentration c = 10 mM) and 2.0 µL of a stock solution of **15** (c = 0.21 mM, 0.42 nmol, 0.30 µg, 1.0 equiv., final concentration c = 8.4 µM) in UHQ-H_2O were added. The mixture was degassed by gently purging argon 2–4 mm above the solution via a needle (Φ 0.80 × 40 mm) for 10 sec.. Then the cap was closed and the mixture was vortexed for 5 sec., transferred into a Thermocycler pre-heated at 25 °C, and incubated at 25 °C for 5 min at 600 rpm. Next, the Eppendorf tube was floated on ice-water bath and the ice-water bath was placed under 1 Kessil PR160-390 nm LED (2.5 cm away from the LED) and irradiated for 15 min. The temperature of the reaction mixture was kept at approximately 0°C through the use of the ice-water bath. Subsequently, 30 µL of an EDTA-Na_2 stock soluition (pH 8.5, 0.30 M, 9.0 µmol, 3.4 mg, 1.2 equiv. to copper ions) in UHQ-

H_2O was added to complex copper ion. The mixture was vortexed for 5 sec., transferred into a Thermocycler pre-heated at 25 °C, and incubated at 25 °C for 20 min at 600 rpm. The resulting mixture was directly analyzed by LC-MS analysis and the recovery of ubiquitin was determined based on the post-run PDA analysis at 214 nm (84%, in triplicate). Bromo-ubiquitin was not observed in either PDA analysis at 214 nm or TIC spectrum.

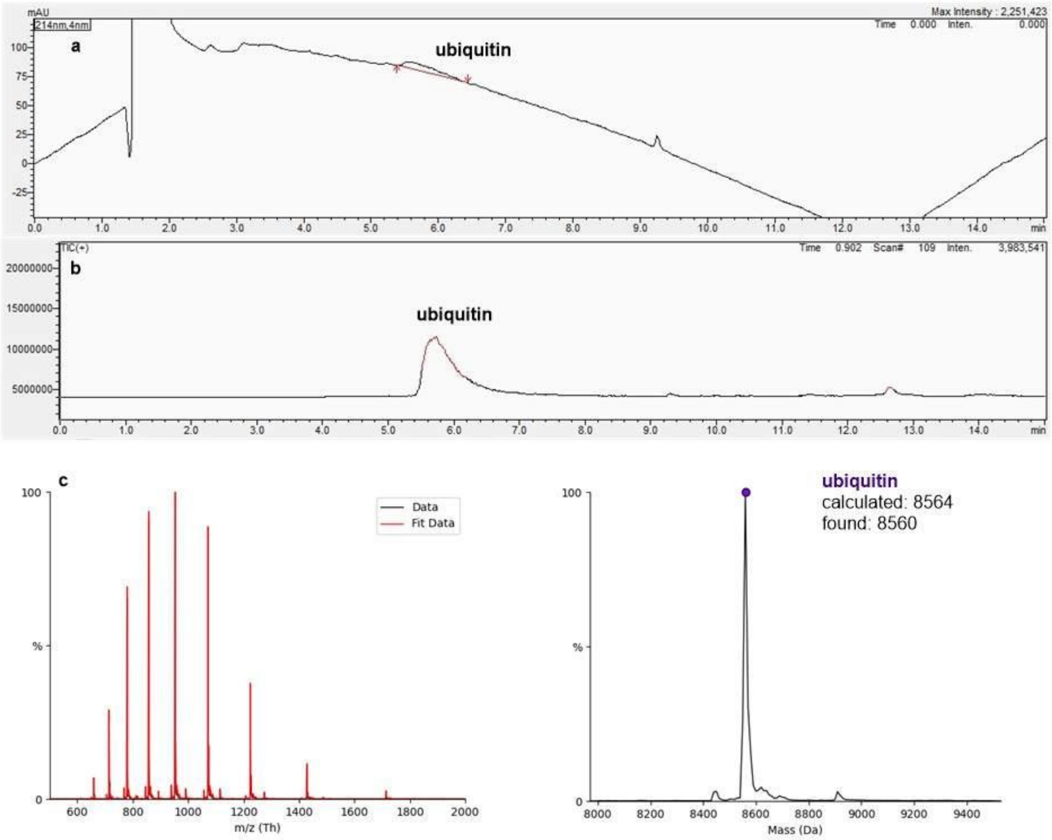

Figure 69. LC-MS analysis (Method A) of the control experiment. a) LC chromatogram (PDA 214 nm) and the peak of ubiquitin mixture. b) Total ion chromatogram. c) Ubiquitin mixture peak ion series and deconvoluted spectrum. Purple spots, ubiquitin. LC injection volume was 5 µL.

UV-vis spectrometry study of NAc-Tyr-NH_2 derived DBSePy-oxa-selenonium salt 15

Blank: Under ambient atmosphere, a scintillation vial (4 mL) equipped with a Teflon-coated magnetic stirring bar was charged with 200 µL of NaPi buffer (pH 6.0, c = 500 mM, final concentration c = 100 mM), 600 µL of UHQ-H_2O, and 200 µL of MeCN (final volume percentage 20%). The mixture was stirred at 25 °C for 5 min and the resulting solution was used as a blank sample. The solution was transferred into a quartz cuvette (1.5 mL) with a screw cap and the absorption spectrum was recorded on a Shimadzu UV-vis Spectrophotometer UV-2600 at 25 °C as a background.

Measure: Under ambient atmosphere, a scintillation vial (4 mL) equipped with a Teflon-coated magnetic stirring bar was charged with 200 µL of NaPi buffer (pH 6.0, c = 500 mM, final concentration c = 100 mM), 440 µL of UHQ-H_2O, and 160 µL of MeCN (final volume percentage 20%). The mixture was stirred at 25 °C for 1 min, followed by the addition of a stock solution of **15** (c = 5.0 mM, 200 µL, 1.0 µmol, final concentration c = 1.0 mM) in MeCN/UHQ-H_2O mixture (1/4, v/v). The mixture was stirred at 25 °C for an additional 5 min and transferred into the same quartz cuvette (1.5 mL). Then, the cuvette was closed by a screw cap and the absorption spectrum was recorded on a Shimadzu UV-vis Spectrophotometer UV-2600 at 25 °C. The measurement was repeated for three times and averaged.

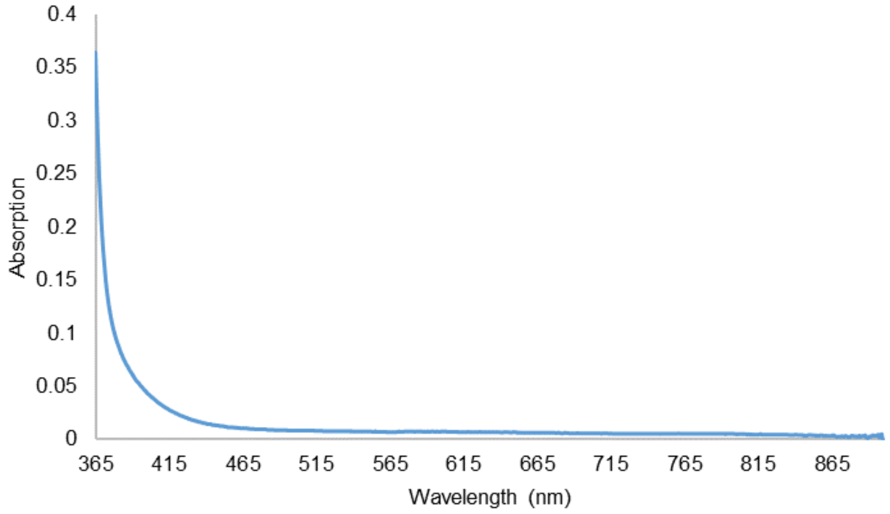

Figure 70. UV-vis absorption spectrum of tyrosinamide selenonium salt **15**.

DFT calculation

Methods

Density Functional Theory (DFT) calculations were performed on the Max-Planck-Institut für Kohlenforschung computer cluster using the ORCA 5.0 program package (Version 5.0 Stable)[23]. Structural optimizations and frequency calculations were performed with the B3LYP functional[24] with D3 dispersion correction[25] and Becke-Johnson damping (BJ)[26] along with RIJCOSX approximation[27], utilizing the def2/J auxiliary basis set[28] and the def2-TZVPP basis set[29] on all atoms. Tight SCF convergence and geometry optimization criterions were chosen. Frequency calculations at the same level had been performed to confirm each stationary point to be either a minimum or a transition structure. Solvent effects of water were taken into account using the conductor-like polarized continuum model (CPCM)[30]. Input files were created using Avogadro 1.2[31] and images were generated using Chemcraft 1.8[32].

Summary of calculation results

Dipole moment of 1 in water

6.64 Debye.

Equilibrium between 5H$^{+}_{Se}$ and 5H$^{+}_{py}$ in aqueous solution

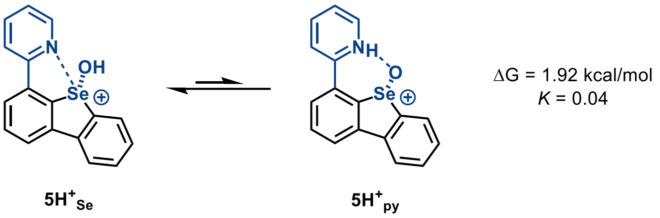

Figure 71. Summary of calculation results.

entry	compound	Free energy G (Ha)
**1**	**5H$^{+}_{Se}$**	–3185.99353803
**2**	**5H$^{+}_{py}$**	–3185.99049544

Table 39. Thermodynamic quantities of **5H$^{+}_{Se}$** and **5H$^{+}_{py}$.**

Dihedral torsions between amide and pyridyl planes in 6H^{+} and 6

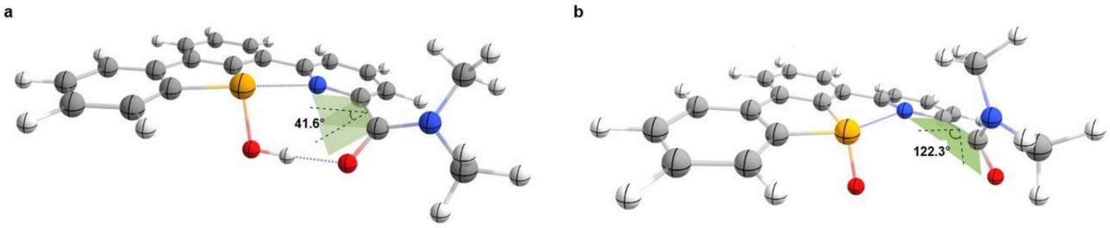

Figure 72. Summary of calculation results.

entry	compound	Free energy G (Ha)
**1**	**6H^{+}**	–3433.22933864
**2**	**6**	–3432.78104336

Table 40. Thermodynamic quantities of compounds used in NMR study.

Bond dissociation energy (BDE) and frontier molecular orbitals of Tyr-selenonium cation

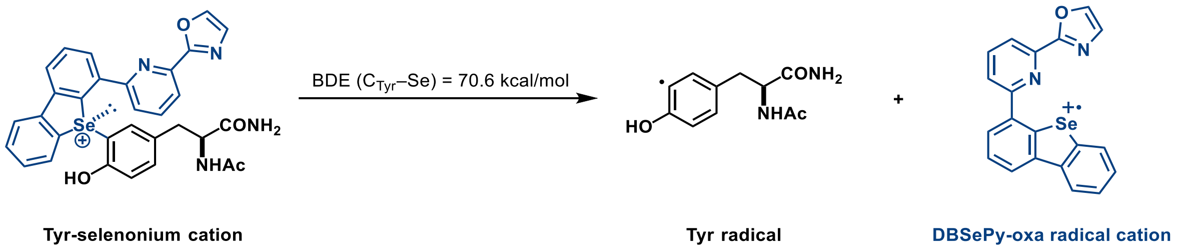

Figure 73. Summary of calculation results.

entry	compound	Enthalpy H (Ha)
**1**	**Tyr-selenonium cation**	–4116.9061718
**2**	**Tyr radical**	–761.84845832
**3**	**DBSePy-oxa radical cation**	–3354.94520652

Table 41. Thermodynamic quantities of Tyr-selenonium salt, Tyr radical, and selenyl radical cation.

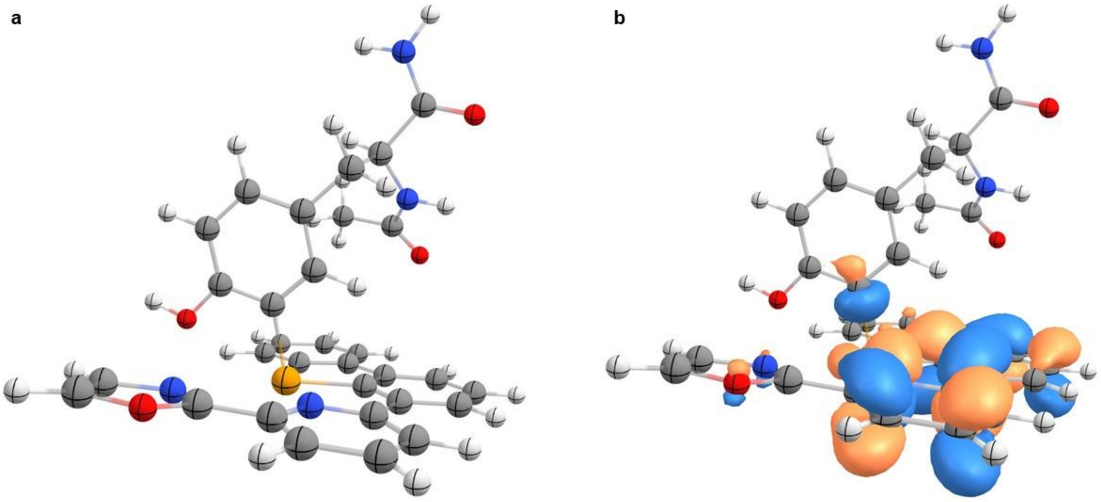

Figure 74. a) Geometry structure of Tyr-selenonium cation. B) Lowest unoccupied molecular orbital (LUMO) of Tyr-selenonium cation.

Cartesian coordinates of the optimized species

1

Se	0.874044	-0.564113	0.566867
C	0.493883	0.255196	-1.172852
C	2.666314	-0.851576	-0.147095
C	-0.716947	0.838057	-1.550837
C	-0.808897	1.328169	-2.861162
H	-1.727808	1.775781	-3.208340
C	0.261565	1.235795	-3.740442
H	0.155609	1.615969	-4.747358
C	1.462121	0.662511	-3.342357
H	2.290481	0.599577	-4.034229

C	1.586567	0.170296	-2.044927
C	2.797065	-0.438667	-1.474780
C	4.021621	-0.617529	-2.116642
H	4.153405	-0.311148	-3.145503
C	5.082522	-1.190979	-1.422365
H	6.033668	-1.324995	-1.919905
C	4.934737	-1.593028	-0.096274
H	5.768991	-2.035005	0.431572
C	3.710813	-1.430699	0.550866
H	3.585980	-1.747415	1.578170
C	-3.894916	1.071525	1.229654
H	-4.673652	1.096085	1.976255
C	-2.762689	0.275662	1.406739
N	-1.779734	0.220433	0.504632
C	-1.860444	0.945457	-0.611917
C	-2.959771	1.775637	-0.860571
H	-3.014055	2.377639	-1.753606
C	-3.981987	1.830799	0.072671
H	-4.839188	2.467549	-0.097617
O	1.101834	0.679623	1.666638
C	-2.599741	-0.539976	2.609555
O	-3.556507	-0.443011	3.572025
C	-1.995828	-1.865825	4.168251
C	-3.157077	-1.295243	4.562216
H	-1.388051	-2.586887	4.686900
H	-3.786434	-1.366028	5.430301
N	-1.652965	-1.373780	2.919231

$5H^{+}_{Se}$

C	-3.492105	-2.276505	-2.458035
C	-2.173269	-2.337179	-2.900239
C	-1.327530	-1.241531	-2.734491
C	-1.851056	-0.107248	-2.143800
C	-3.171964	-0.021529	-1.687428
C	-3.996606	-1.129654	-1.849000
H	-4.137211	-3.135142	-2.584957
H	-1.799093	-3.236377	-3.369318
H	-0.298492	-1.279517	-3.063989
H	-5.021411	-1.105538	-1.505642
C	-2.489270	2.204099	-1.042177
C	-2.615861	3.488693	-0.522184
C	-3.872848	3.830400	-0.008509

C	-4.918631	2.915879	-0.023979
C	-4.760466	1.636995	-0.552466
C	-3.527445	1.267762	-1.078269
H	-4.044121	4.818896	0.390616
H	-5.879274	3.208353	0.376953
H	-5.589217	0.943348	-0.554697
Se	-0.861524	1.499717	-1.758651
O	-0.867117	2.073409	-3.449064
C	-1.471533	4.420917	-0.547330
C	-1.439862	5.612727	0.175420
N	-0.428579	4.038368	-1.300767
C	-0.308309	6.411963	0.101285
H	-2.271996	5.905601	0.796733
C	0.663440	4.798665	-1.359486
C	0.766270	6.003670	-0.678710
H	1.473537	4.432183	-1.977327
H	1.663616	6.599545	-0.760405
H	-0.463898	2.958153	-3.425208
H	-0.264744	7.340230	0.654163

5H$^{+}_{py}$

C	-3.630331	-2.315352	-2.484395
C	-2.311229	-2.454559	-2.909808
C	-1.398354	-1.420981	-2.709215
C	-1.854177	-0.264082	-2.106202
C	-3.173158	-0.097510	-1.671412
C	-4.066474	-1.148457	-1.863509
H	-4.328698	-3.126939	-2.636773
H	-1.990324	-3.367469	-3.392041
H	-0.368065	-1.521909	-3.022190
H	-5.093640	-1.062437	-1.537472
C	-2.379738	2.093792	-1.027142
C	-2.502992	3.408789	-0.575525
C	-3.764072	3.816265	-0.119660
C	-4.841152	2.940504	-0.125917
C	-4.704460	1.644901	-0.606905
C	-3.465300	1.212929	-1.074733
H	-3.906847	4.833876	0.213441
H	-5.803204	3.282212	0.229374
H	-5.558041	0.982019	-0.626694
Se	-0.742422	1.254515	-1.668702
O	-0.445459	2.102523	-3.100530

C	-1.408518	4.396015	-0.589361
C	-1.257265	5.362039	0.398268
N	-0.520913	4.394731	-1.610739
C	-0.225320	6.284508	0.315519
H	-1.936711	5.373168	1.235929
C	0.491730	5.269234	-1.710098
C	0.664235	6.243234	-0.753558
H	-0.109234	7.029235	1.090443
H	1.133327	5.151632	-2.569991
H	1.478650	6.945451	-0.842658
H	-0.578884	3.656792	-2.350712

$6H^+$

Se	0.623064	-0.773426	-0.232669
C	1.226543	1.044736	-0.091800
C	2.478441	-1.282711	-0.123427
C	0.387733	2.156854	-0.051296
C	1.009890	3.410427	-0.031599
H	0.415836	4.310748	0.010750
C	2.395146	3.517613	-0.052633
H	2.847918	4.499339	-0.037770
C	3.208666	2.389547	-0.081149
H	4.284308	2.494323	-0.089785
C	2.622317	1.127725	-0.095871
C	3.320440	-0.165899	-0.107247
C	4.695916	-0.375325	-0.094277
H	5.377974	0.463401	-0.085196
C	5.190212	-1.677113	-0.090411
H	6.259629	-1.837430	-0.075887
C	4.331116	-2.773251	-0.107609
H	4.732702	-3.776972	-0.103918
C	2.950964	-2.581606	-0.135732
H	2.274358	-3.424719	-0.159895
C	-3.757295	1.437888	0.268425
H	-4.800975	1.193454	0.395406
C	-2.795491	0.438237	0.181995
N	-1.500102	0.721730	0.076209
C	-1.078153	1.986285	0.020936
C	-1.986239	3.046866	0.072801
H	-1.652122	4.071316	0.022750
C	-3.334747	2.760758	0.208371

H	-4.055371	3.563970	0.273487
O	0.180247	-1.246720	1.407242
C	-3.107647	-1.025857	0.354918
O	-2.383454	-1.695783	1.119612
N	-4.161309	-1.553559	-0.268466
C	-4.554482	-2.928136	0.029556
H	-5.638517	-2.997473	-0.039611
H	-4.108965	-3.617827	-0.689926
H	-4.232488	-3.195474	1.029810
C	-4.882173	-0.928878	-1.375608
H	-5.884627	-0.634334	-1.063969
H	-4.347369	-0.063965	-1.750055
H	-4.965045	-1.657410	-2.181702
H	-0.821694	-1.349965	1.383209

6

Se	0.392336	-0.858256	-0.264192
C	0.989540	0.996516	-0.048764
C	2.279009	-1.314995	-0.066977
C	0.160603	2.120464	-0.031953
C	0.779092	3.369086	0.117897
H	0.183925	4.269680	0.131339
C	2.158155	3.477059	0.238496
H	2.606224	4.455388	0.346707
C	2.965923	2.347119	0.221315
H	4.038374	2.444398	0.317456
C	2.380153	1.090466	0.081737
C	3.099104	-0.192464	0.068895
C	4.475940	-0.377101	0.186072
H	5.138078	0.471749	0.289340
C	4.998841	-1.666727	0.172315
H	6.067110	-1.808700	0.266302
C	4.164775	-2.774945	0.038266
H	4.585463	-3.771382	0.030056
C	2.787625	-2.601218	-0.090317
H	2.133715	-3.456256	-0.202635
C	-4.013810	1.556570	-0.408232
H	-5.067614	1.336499	-0.495907
C	-3.066011	0.560013	-0.610322
N	-1.761219	0.783527	-0.493631
C	-1.309820	1.997384	-0.166517

C	-2.196063	3.058909	0.036460
H	-1.840516	4.042907	0.299246
C	-3.558286	2.829546	-0.092042
H	-4.259643	3.637977	0.063062
O	-0.267657	-1.336493	1.201181
C	-3.489450	-0.864816	-0.866680
O	-4.266840	-1.397057	-0.066917
N	-2.958420	-1.499205	-1.926454
C	-3.193311	-2.924234	-2.108521
H	-3.960271	-3.097750	-2.866940
H	-2.266021	-3.393752	-2.437141
H	-3.512091	-3.364640	-1.170102
C	-2.243635	-0.842704	-3.013533
H	-2.693955	-1.144019	-3.960474
H	-2.309608	0.235227	-2.927624
H	-1.193081	-1.136424	-3.015205

Tyr-selenonium cation

Se	-2.487388	-1.105397	-0.575729
C	-3.497444	0.321554	0.235990
C	-0.893895	-1.135957	0.527249
C	-3.330217	1.677001	-0.046401
C	-4.465282	-0.166527	1.119690
C	-3.544232	-2.315227	0.526670
C	-0.606895	-0.127830	1.429316
C	-0.058813	-2.238678	0.365608
C	-4.187355	2.564305	0.614806
C	-2.279621	2.142173	-0.977232
C	-5.306234	0.746946	1.751468
C	-4.486959	-1.621020	1.288960
C	-3.426048	-3.692637	0.554837
C	0.539510	-0.205976	2.215998
H	-1.275847	0.711885	1.541821
C	1.103065	-2.304258	1.130162
O	-0.429665	-3.195414	-0.517783
C	-5.157630	2.102568	1.495121
H	-4.096898	3.626988	0.449745
C	-2.110173	3.483325	-1.334212
N	-1.478243	1.191788	-1.458579
H	-6.062253	0.403902	2.442606
C	-5.334675	-2.350529	2.120918

C	-4.275129	-4.403725	1.399462
H	-2.690791	-4.202661	-0.048516
C	0.798528	0.805313	3.294282
C	1.388722	-1.300397	2.042792
H	1.768154	-3.150084	1.009063
H	-5.803520	2.814309	1.990324
C	-1.076153	3.817928	-2.193732
H	-2.765589	4.250351	-0.954339
C	-0.484857	1.509602	-2.293425
C	-5.219999	-3.734203	2.174606
H	-6.073021	-1.844952	2.727021
H	-4.198765	-5.481248	1.449089
H	1.868635	0.875828	3.489901
H	0.444902	1.792886	2.995738
H	2.285608	-1.376126	2.644043
C	-0.240954	2.825117	-2.684993
H	-0.922507	4.848718	-2.481276
H	-5.874613	-4.297385	2.825648
H	0.572899	3.056813	-3.354797
C	0.336807	0.403227	-2.770155
O	1.444364	0.686859	-3.504895
C	1.219795	-1.481537	-3.230175
C	2.000394	-0.528991	-3.791542
H	1.342099	-2.550633	-3.242798
H	2.903962	-0.527856	-4.373138
N	0.160403	-0.868804	-2.583635
H	0.224404	-3.903862	-0.557585
C	0.084645	0.419643	4.618062
H	0.376581	-0.589774	4.896373
C	0.532263	1.398847	5.709235
O	-0.062890	2.459147	5.886090
N	1.621774	1.035323	6.399086
H	2.015049	1.663602	7.082052
H	2.080802	0.154548	6.240172
N	-1.356269	0.494123	4.509489
C	-2.259055	-0.511270	4.440578
H	-1.744375	1.421651	4.617739
O	-3.466376	-0.263941	4.471938
C	-1.747311	-1.926750	4.327158
H	-1.034615	-2.034157	3.512226
H	-1.244067	-2.222398	5.249578

H	-2.593614	-2.585501	4.156891

Tyr radical

C	-0.855701	-1.029753	0.481580
C	-0.588230	-0.048399	1.392946
C	-0.124733	-2.183290	0.298463
C	0.526131	-0.214648	2.233660
H	-1.211828	0.832510	1.482410
C	0.989977	-2.336951	1.131011
O	-0.490879	-3.090352	-0.649368
C	0.823156	0.777881	3.321586
C	1.298690	-1.365284	2.076601
H	1.608790	-3.221594	1.028583
H	1.895447	0.808930	3.518398
H	0.508033	1.779166	3.024527
H	2.160740	-1.512920	2.714842
H	0.127479	-3.831257	-0.648744
C	0.099379	0.421627	4.647637
H	0.406008	-0.572679	4.959891
C	0.510328	1.442280	5.712753
O	-0.088796	2.508739	5.828307
N	1.576403	1.107102	6.453318
H	1.947011	1.763060	7.122780
H	2.042315	0.222461	6.343155
N	-1.340110	0.455124	4.510099
C	-2.193957	-0.581126	4.357800
H	-1.753586	1.376486	4.467252
O	-3.398449	-0.378232	4.182107
C	-1.631580	-1.980613	4.422536
H	-0.933384	-2.156176	3.604782
H	-1.095644	-2.145922	5.357893
H	-2.454057	-2.685747	4.350468

DBSePy-oxa radical cation

Se	0.117845	-0.496290	-0.042733
C	-0.097753	0.562436	-1.582160
C	1.907869	-0.758156	-0.568713
C	-1.288139	1.203157	-1.973515
C	-1.252131	1.935909	-3.168453
H	-2.132275	2.463118	-3.503115
C	-0.092573	2.021560	-3.919363
H	-0.091773	2.602042	-4.831241

C	1.082951	1.376926	-3.514719
H	1.976147	1.458349	-4.118119
C	1.089400	0.642146	-2.342572
C	2.217660	-0.095659	-1.773894
C	3.502131	-0.212406	-2.282637
H	3.776218	0.273598	-3.208653
C	4.451277	-0.969878	-1.589614
H	5.452090	-1.054197	-1.990282
C	4.131429	-1.615564	-0.395022
H	4.881889	-2.194958	0.123971
C	2.850591	-1.513853	0.130020
H	2.587657	-2.006997	1.055837
C	-4.679560	0.844573	0.501043
H	-5.509569	0.729938	1.181150
C	-3.380893	0.509560	0.900464
N	-2.334333	0.645203	0.087394
C	-2.505075	1.117086	-1.149667
C	-3.771547	1.474215	-1.625972
H	-3.910276	1.830890	-2.634375
C	-4.863826	1.331033	-0.782684
H	-5.853729	1.594853	-1.127685
C	-3.105380	0.007242	2.242231
O	-4.160456	-0.214508	3.069452
C	-2.268327	-0.704497	4.069075
C	-3.612185	-0.669830	4.233204
H	-1.510554	-1.005978	4.771272
H	-4.289308	-0.908477	5.032836
N	-1.960382	-0.267073	2.793193

X-Ray Crystallographic Data

Crystal data of $5H^{+}MsO^{-}$ (CCDC 2304770)

The atoms are depicted with 50% probability ellipsoids. The crystallographic data are summarized in the following table.

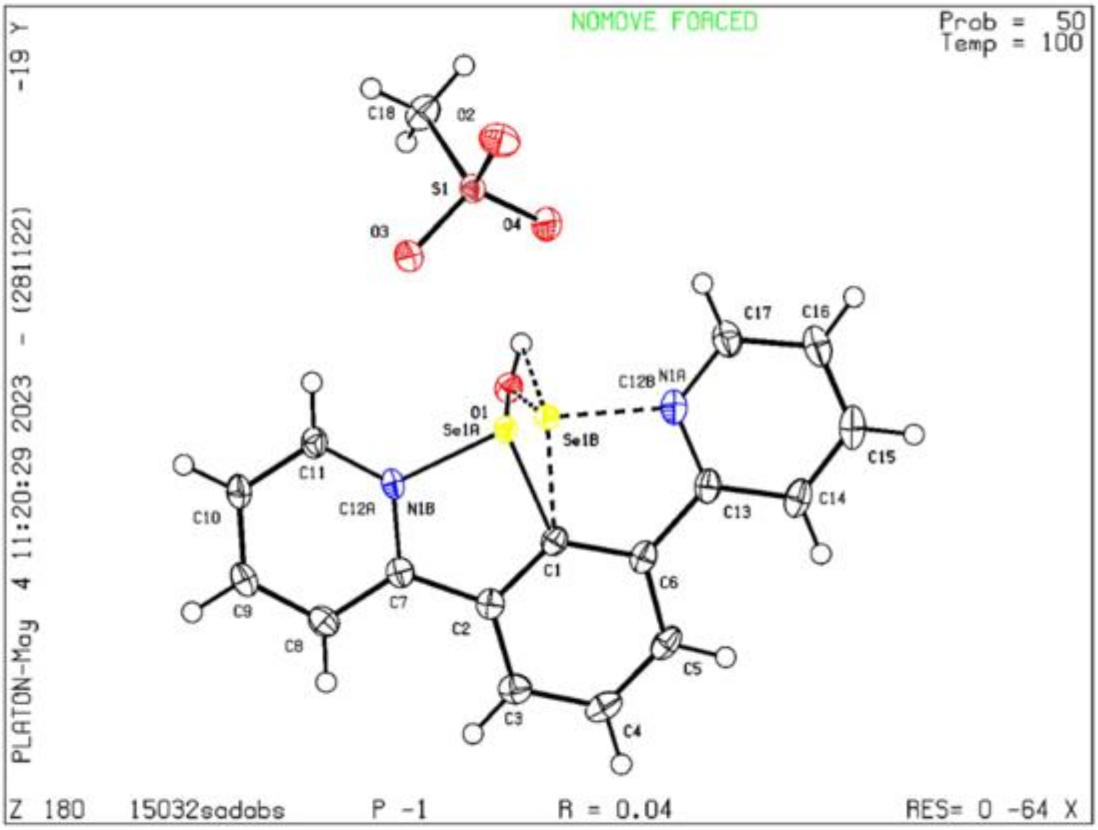

Figure 75. X-ray structure of **$5H^{+}MsO^{-}$**. Blue = nitrogen, yellow= selenium, red = oxygen. The H atom attached to O1 was found and refined, others were refined isotropically. There was a disorder on selenium atom, which showed 93% probability in Se1A and 7% probability in Se1B, due to the existence of the enantiomer of ca. 7% amount. An A-alert of the short distance between Se1A and O2 (2.80 Å) was reported in the corresponding checkcif file, and the short distance was due to the formation of a new chalcogen bond between O2 of MsO^{-} and Se–O bond of **$5H^{+}$** during the crystallization.

Crystal data and structure refinement

Identification code	15032	
Empirical formula	$C_{18} H_{15} N O_4 S Se$	
Color	light yellow	
Formula weight	420.33 g · mol^{-1}	
Temperature	100(2) K	
Wavelength	0.71073 Å	
Crystal system	Triclinic	
Space group	**$P\bar{1}$, (no. 2)**	
Unit cell dimensions	a = 8.7730(6) Å	α = 105.439(4)°.

	b = 10.2182(7) Å	β = 103.247(4)°.
	c = 11.0865(8) Å	γ = 111.543(4)°.
Volume	829.71(10) $Å^3$	
Z	2	
Density (calculated)	1.682 $Mg \cdot m^{-3}$	
Absorption coefficient	2.412 mm^{-1}	
F(000)	424 e	
Crystal size	0.245 x 0.086 x 0.038 mm^3	
θ range for data collection	2.047 to 31.197°.	
Index ranges	$-12 \le h \le 12$, $-14 \le k \le 14$, $-16 \le l \le 16$	
Reflections collected	153013	
Independent reflections	5366 [R_{int} = 0.0930]	
Reflections with I>2σ(I)	4689	
Completeness to θ = 25.242°	100.0 %	
Absorption correction	Semi-empirical from equivalents	
Max. and min. transmission	0.95 and 0.72	
Refinement method	Full-matrix least-squares on F^2	
Data / restraints / parameters	5366 / 0 / 235	
Goodness-of-fit on F^2	1.046	
Final R indices [I>2σ(I)]	R_1 = 0.0398	wR^2 = 0.0963
R indices (all data)	R_1 = 0.0480	wR^2 = 0.1004
Largest diff. peak and hole	1.1 and -0.9 $e \cdot Å^{-3}$	

Bond lengths [Å] and angles [°].

Se(1A)-O(1)	1.7718(17)	Se(1A)-C(1)	1.914(2)
Se(1A)-C(12A)	1.924(2)	Se(1B)-O(1)	1.796(4)
Se(1B)-H(1)	1.90(4)	Se(1B)-C(1)	1.942(4)
Se(1B)-C(12B)	1.961(5)	O(1)-H(1)	0.72(4)
N(1A)-C(13)	1.343(3)	N(1A)-C(17)	1.337(3)
N(1B)-C(7)	1.394(3)	N(1B)-C(11)	1.378(3)
C(1)-C(2)	1.392(3)	C(1)-C(6)	1.397(3)
C(2)-C(3)	1.393(3)	C(2)-C(7)	1.474(3)
C(3)-C(4)	1.400(4)	C(4)-C(5)	1.377(4)
C(5)-C(6)	1.405(3)	C(6)-C(13)	1.478(3)
C(7)-C(8)	1.400(3)	C(7)-C(12A)	1.394(3)
C(8)-C(9)	1.390(4)	C(9)-C(10)	1.389(4)
C(10)-C(11)	1.387(3)	C(11)-C(12A)	1.378(3)
C(12B)-C(13)	1.343(3)	C(12B)-C(17)	1.337(3)
C(13)-C(14)	1.401(3)	C(14)-C(15)	1.384(4)
C(15)-C(16)	1.385(4)	C(16)-C(17)	1.393(4)
S(1)-O(2)	1.4484(19)	S(1)-O(3)	1.4584(19)
S(1)-O(4)	1.4712(19)	S(1)-C(18)	1.763(3)

O(1)-Se(1A)-C(1)	96.19(9)	O(1)-Se(1A)-C(12A)	96.70(9)
C(1)-Se(1A)-C(12A)	87.32(10)	O(1)-Se(1B)-H(1)	22.3(12)
O(1)-Se(1B)-C(1)	94.42(17)	O(1)-Se(1B)-C(12B)	103.4(2)
C(1)-Se(1B)-H(1)	115.6(12)	C(1)-Se(1B)-C(12B)	90.12(18)
C(12B)-Se(1B)-H(1)	95.5(12)	Se(1A)-O(1)-H(1)	98(3)
Se(1B)-O(1)-H(1)	87(3)	C(17)-N(1A)-C(13)	119.4(2)
C(11)-N(1B)-C(7)	123.3(2)	C(2)-C(1)-Se(1A)	111.44(17)
C(2)-C(1)-Se(1B)	131.4(2)	C(2)-C(1)-C(6)	123.8(2)
C(6)-C(1)-Se(1A)	124.69(18)	C(6)-C(1)-Se(1B)	104.8(2)
C(1)-C(2)-C(3)	118.8(2)	C(1)-C(2)-C(7)	115.2(2)
C(3)-C(2)-C(7)	126.0(2)	C(2)-C(3)-C(4)	118.6(2)
C(5)-C(4)-C(3)	121.6(2)	C(4)-C(5)-C(6)	121.3(2)
C(1)-C(6)-C(5)	115.9(2)	C(1)-C(6)-C(13)	120.8(2)
C(5)-C(6)-C(13)	123.3(2)	N(1B)-C(7)-C(2)	114.7(2)
N(1B)-C(7)-C(8)	118.2(2)	C(8)-C(7)-C(2)	127.1(2)
C(12A)-C(7)-C(2)	114.7(2)	C(12A)-C(7)-C(8)	118.2(2)
C(9)-C(8)-C(7)	118.7(2)	C(10)-C(9)-C(8)	121.9(2)
C(11)-C(10)-C(9)	119.9(2)	N(1B)-C(11)-C(10)	118.0(2)
C(12A)-C(11)-C(10)	118.0(2)	C(7)-C(12A)-Se(1A)	111.32(16)
C(11)-C(12A)-Se(1A)	125.31(17)	C(11)-C(12A)-C(7)	123.3(2)

C(13)-C(12B)-Se(1B)	109.24(19)	C(17)-C(12B)-Se(1B)	131.3(2)
C(17)-C(12B)-C(13)	119.4(2)	N(1A)-C(13)-C(6)	115.1(2)
N(1A)-C(13)-C(14)	121.4(2)	C(12B)-C(13)-C(6)	115.1(2)
C(12B)-C(13)-C(14)	121.4(2)	C(14)-C(13)-C(6)	123.6(2)
C(15)-C(14)-C(13)	118.8(2)	C(14)-C(15)-C(16)	119.7(2)
C(15)-C(16)-C(17)	118.2(3)	N(1A)-C(17)-C(16)	122.5(3)
C(12B)-C(17)-C(16)	122.5(3)	O(2)-S(1)-O(3)	112.96(12)
O(2)-S(1)-O(4)	112.76(12)	O(2)-S(1)-C(18)	106.56(13)
O(3)-S(1)-O(4)	111.13(11)	O(3)-S(1)-C(18)	107.58(13)
O(4)-S(1)-C(18)	105.30(12)		

Crystal data of $1H^+HSO_4^-\cdot(MeOH)_2$ (CCDC 2291793)

The atoms are depicted with 50% probability ellipsoids. The crystallographic data are summarized in the following table.

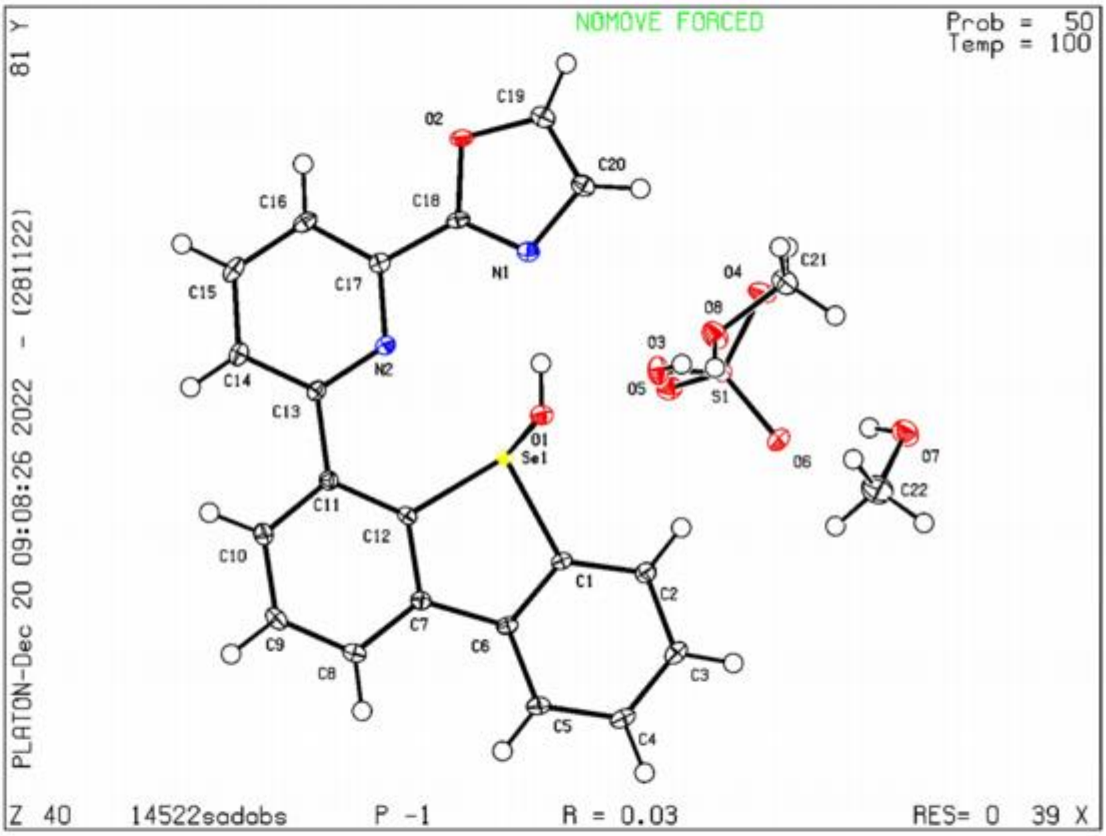

Figure 76. X-ray structure of **$1H^+HSO_4^-\cdot(MeOH)_2$**. Blue = nitrogen, yellow= selenium, red = oxygen. The H atoms attached to O1, O3, and O8 were found and refined, others were refined isotropically.

Crystal data and structure refinement

Identification code	14522	
Empirical formula	C_{22} H_{22} N_2 O_8 S Se	
Color	yellow	
Formula weight	553.43 g·mol^{-1}	
Temperature	100(2) K	
Wavelength	0.71073 Å	
Crystal system	Triclinic	
Space group	***P*-1, (no. 2)**	
Unit cell dimensions	a = 7.0046(3) Å	α = 96.446(2)°.
	b = 12.1537(5) Å	β = 96.762(2)°.
	c = 13.0524(6) Å	γ = 102.882(2)°.
Volume	1064.60(8) Å^3	
Z	2	
Density (calculated)	1.726 Mg·m^{-3}	
Absorption coefficient	1.918 mm^{-1}	
F(000)	564 e	

Crystal size	0.12 x 0.074 x 0.062 mm^3	
θ range for data collection	1.587 to 31.506°.	
Index ranges	$-10 \le h \le 10$, $-17 \le k \le 17$, $-19 \le l \le 19$	
Reflections collected	36604	
Independent reflections	7088 [R_{int} = 0.0337]	
Reflections with I>2σ(I)	6240	
Completeness to θ = 25.242°	100.0 %	
Absorption correction	Gaussian	
Max. and min. transmission	0.92805 and 0.87540	
Refinement method	Full-matrix least-squares on F^2	
Data / restraints / parameters	7088 / 0 / 322	
Goodness-of-fit on F^2	1.047	
Final R indices [I>2σ(I)]	R_1 = 0.0290	wR^2 = 0.0665
R indices (all data)	R_1 = 0.0363	wR^2 = 0.0689
Extinction coefficient	n/a	
Largest diff. peak and hole	0.579 and -0.566 e·$Å^{-3}$	

Bond lengths [Å] and angles [°].

Se(1)-O(1)	1.7487(11)	Se(1)-C(1)	1.9198(15)
Se(1)-C(12)	1.9096(14)	S(1)-O(3)	1.5731(12)
S(1)-O(4)	1.4429(12)	S(1)-O(5)	1.4599(12)
S(1)-O(6)	1.4514(12)	O(1)-H(1)	0.86(3)
O(2)-C(18)	1.3513(18)	O(2)-C(19)	1.3811(19)
O(3)-H(3A)	0.79(3)	O(8)-H(8A)	0.86(3)
O(8)-C(21)	1.426(2)	N(1)-C(18)	1.299(2)
N(1)-C(20)	1.393(2)	N(2)-C(13)	1.3374(19)
N(2)-C(17)	1.343(2)	C(1)-C(2)	1.386(2)
C(1)-C(6)	1.398(2)	C(2)-H(2)	0.9500
C(2)-C(3)	1.392(2)	C(3)-H(3)	0.9500
C(3)-C(4)	1.392(2)	C(4)-H(4)	0.9500
C(4)-C(5)	1.395(2)	C(5)-H(5)	0.9500
C(5)-C(6)	1.391(2)	C(6)-C(7)	1.469(2)
C(7)-C(8)	1.391(2)	C(7)-C(12)	1.397(2)
C(8)-H(8)	0.9500	C(8)-C(9)	1.386(2)
C(9)-H(9)	0.9500	C(9)-C(10)	1.389(2)
C(10)-H(10)	0.9500	C(10)-C(11)	1.397(2)
C(11)-C(12)	1.398(2)	C(11)-C(13)	1.474(2)
C(13)-C(14)	1.399(2)	C(14)-H(14)	0.9500
C(14)-C(15)	1.381(2)	C(15)-H(15)	0.9500
C(15)-C(16)	1.388(2)	C(16)-H(16)	0.9500
C(16)-C(17)	1.388(2)	C(17)-C(18)	1.456(2)
C(19)-H(19)	0.9500	C(19)-C(20)	1.337(2)
C(20)-H(20)	0.9500	C(21)-H(21A)	0.9800
C(21)-H(21B)	0.9800	C(21)-H(21C)	0.9800
O(7)-H(7)	0.8400	O(7)-C(22)	1.417(2)
C(22)-H(22A)	0.9800	C(22)-H(22B)	0.9800
C(22)-H(22C)	0.9800		
O(1)-Se(1)-C(1)	97.27(6)	O(1)-Se(1)-C(12)	105.09(6)
C(12)-Se(1)-C(1)	87.09(6)	O(4)-S(1)-O(3)	107.65(7)
O(4)-S(1)-O(5)	113.46(8)	O(4)-S(1)-O(6)	113.25(7)
O(5)-S(1)-O(3)	102.24(7)	O(6)-S(1)-O(3)	106.87(7)
O(6)-S(1)-O(5)	112.41(7)	Se(1)-O(1)-H(1)	102(2)
C(18)-O(2)-C(19)	104.47(12)	S(1)-O(3)-H(3A)	109(2)
C(21)-O(8)-H(8A)	108.8(18)	C(18)-N(1)-C(20)	104.61(13)
C(13)-N(2)-C(17)	118.83(13)	C(2)-C(1)-Se(1)	125.48(12)
C(2)-C(1)-C(6)	123.09(14)	C(6)-C(1)-Se(1)	111.41(11)

C(1)-C(2)-H(2)	121.2	C(1)-C(2)-C(3)	117.58(14)
C(3)-C(2)-H(2)	121.2	C(2)-C(3)-H(3)	119.8
C(4)-C(3)-C(2)	120.34(14)	C(4)-C(3)-H(3)	119.8
C(3)-C(4)-H(4)	119.3	C(3)-C(4)-C(5)	121.34(15)
C(5)-C(4)-H(4)	119.3	C(4)-C(5)-H(5)	120.5
C(6)-C(5)-C(4)	119.00(14)	C(6)-C(5)-H(5)	120.5
C(1)-C(6)-C(7)	114.78(13)	C(5)-C(6)-C(1)	118.63(14)
C(5)-C(6)-C(7)	126.59(14)	C(8)-C(7)-C(6)	126.16(14)
C(8)-C(7)-C(12)	119.15(14)	C(12)-C(7)-C(6)	114.69(13)
C(7)-C(8)-H(8)	120.7	C(9)-C(8)-C(7)	118.69(15)
C(9)-C(8)-H(8)	120.7	C(8)-C(9)-H(9)	119.1
C(8)-C(9)-C(10)	121.83(14)	C(10)-C(9)-H(9)	119.1
C(9)-C(10)-H(10)	119.7	C(9)-C(10)-C(11)	120.61(14)
C(11)-C(10)-H(10)	119.7	C(10)-C(11)-C(12)	116.87(14)
C(10)-C(11)-C(13)	122.03(14)	C(12)-C(11)-C(13)	121.09(13)
C(7)-C(12)-Se(1)	111.77(11)	C(7)-C(12)-C(11)	122.79(13)
C(11)-C(12)-Se(1)	125.14(11)	N(2)-C(13)-C(11)	115.16(13)
N(2)-C(13)-C(14)	121.66(14)	C(14)-C(13)-C(11)	123.18(13)
C(13)-C(14)-H(14)	120.5	C(15)-C(14)-C(13)	119.02(14)
C(15)-C(14)-H(14)	120.5	C(14)-C(15)-H(15)	120.3
C(14)-C(15)-C(16)	119.50(14)	C(16)-C(15)-H(15)	120.3
C(15)-C(16)-H(16)	121.0	C(15)-C(16)-C(17)	117.99(15)
C(17)-C(16)-H(16)	121.0	N(2)-C(17)-C(16)	122.96(14)
N(2)-C(17)-C(18)	113.88(13)	C(16)-C(17)-C(18)	123.13(14)
O(2)-C(18)-C(17)	119.17(13)	N(1)-C(18)-O(2)	113.81(13)
N(1)-C(18)-C(17)	127.01(14)	O(2)-C(19)-H(19)	126.0
C(20)-C(19)-O(2)	107.92(14)	C(20)-C(19)-H(19)	126.0
N(1)-C(20)-H(20)	125.4	C(19)-C(20)-N(1)	109.19(14)
C(19)-C(20)-H(20)	125.4	O(8)-C(21)-H(21A)	109.5
O(8)-C(21)-H(21B)	109.5	O(8)-C(21)-H(21C)	109.5
H(21A)-C(21)-H(21B)	109.5	H(21A)-C(21)-H(21C)	109.5
H(21B)-C(21)-H(21C)	109.5	C(22)-O(7)-H(7)	109.5
O(7)-C(22)-H(22A)	109.5	O(7)-C(22)-H(22B)	109.5
O(7)-C(22)-H(22C)	109.5	H(22A)-C(22)-H(22B)	109.5
H(22A)-C(22)-H(22C)	109.5	H(22B)-C(22)-H(22C)	109.5

NMR DATA

^{1}H NMR of S1

$CDCl_3$, 500 MHz, 298 K

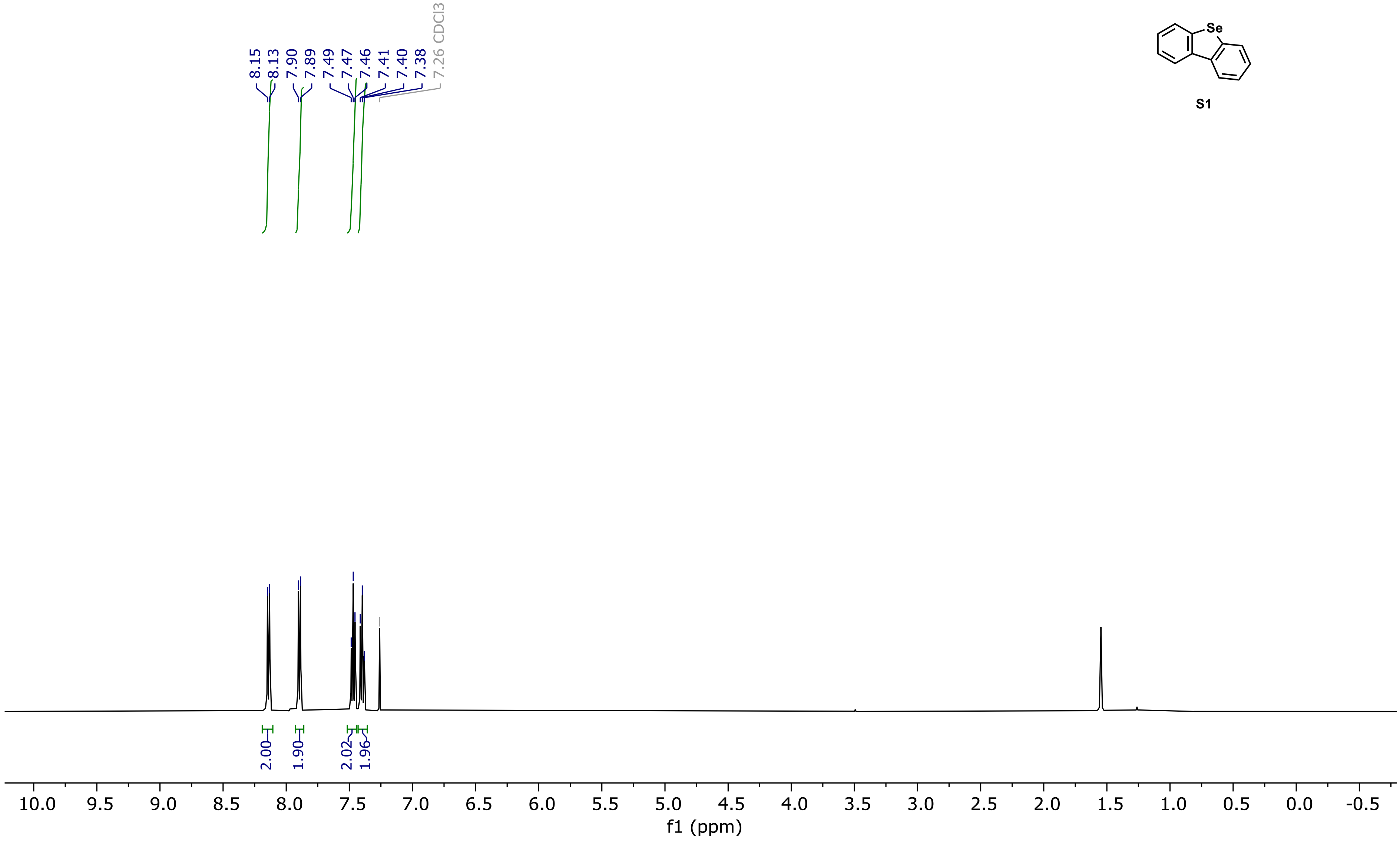

^{13}C NMR of S1

$CDCl_3$, 126 MHz, 298 K

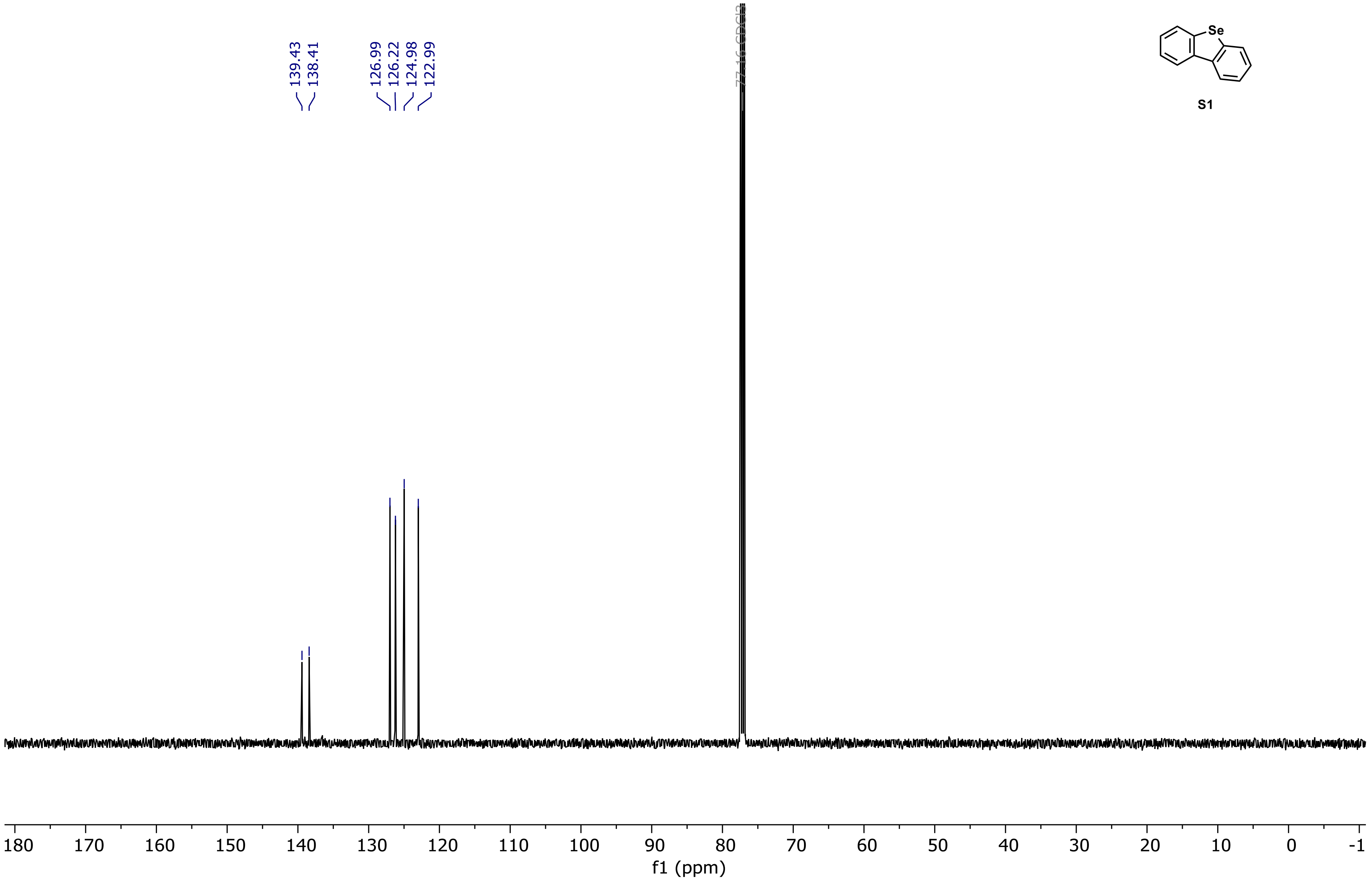

^{77}Se NMR of S1

$CDCl_3$, 95 MHz, 298 K

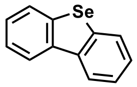

S1

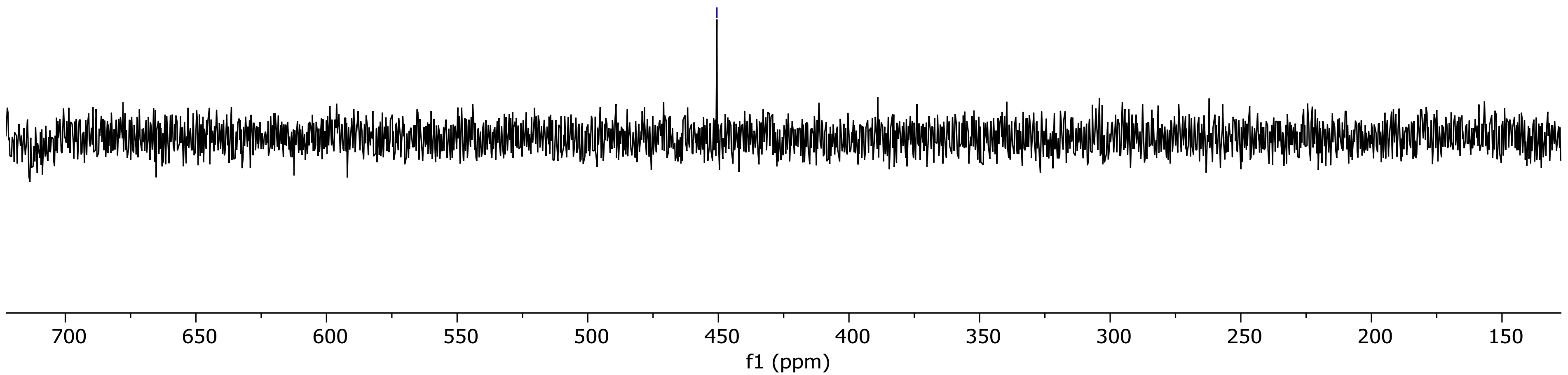

^{1}H NMR of 4

DMSO-d_6, 500 MHz, 298 K

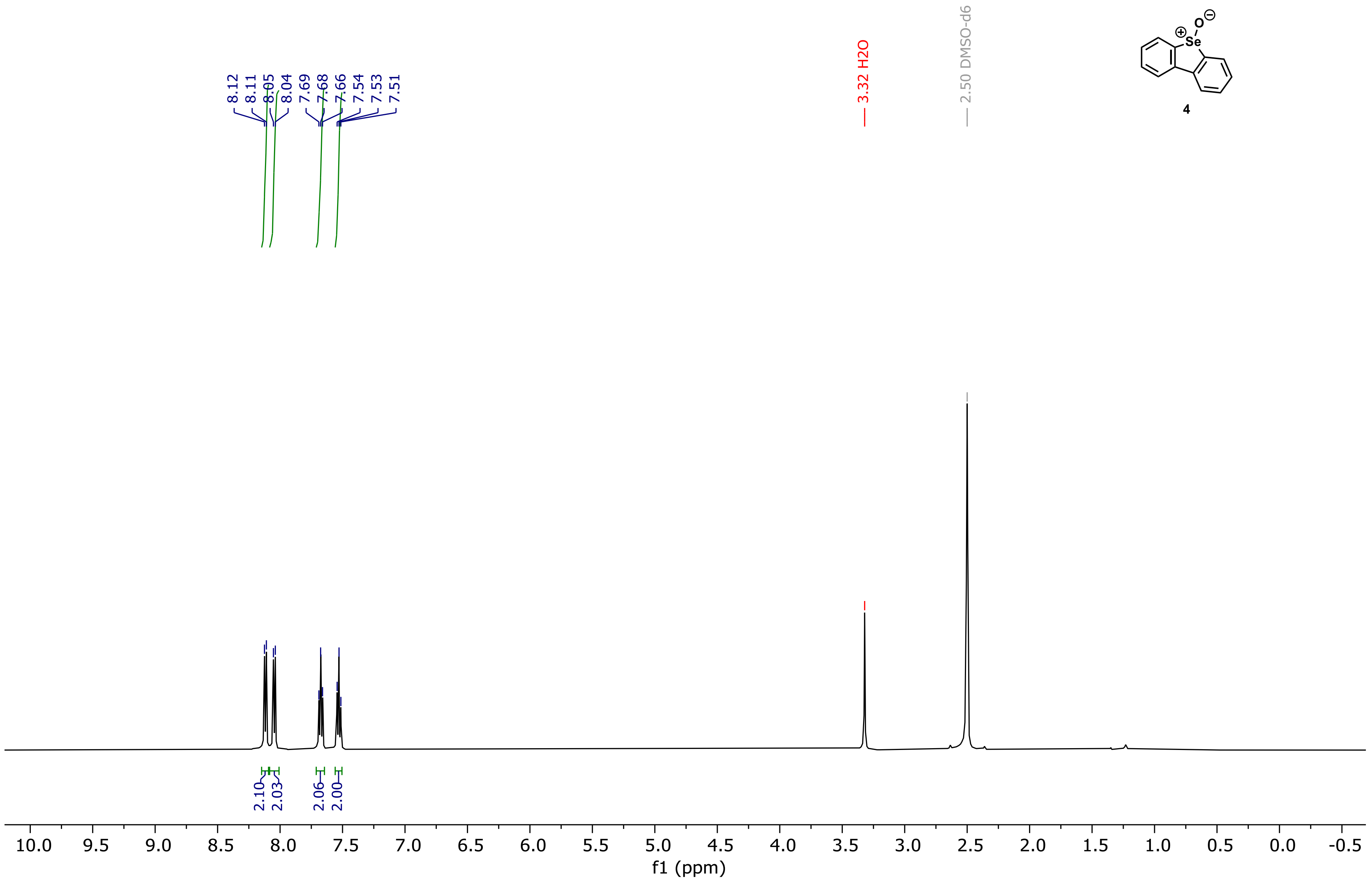

4

^{13}C NMR of 4

DMSO-d_6, 126 MHz, 298 K

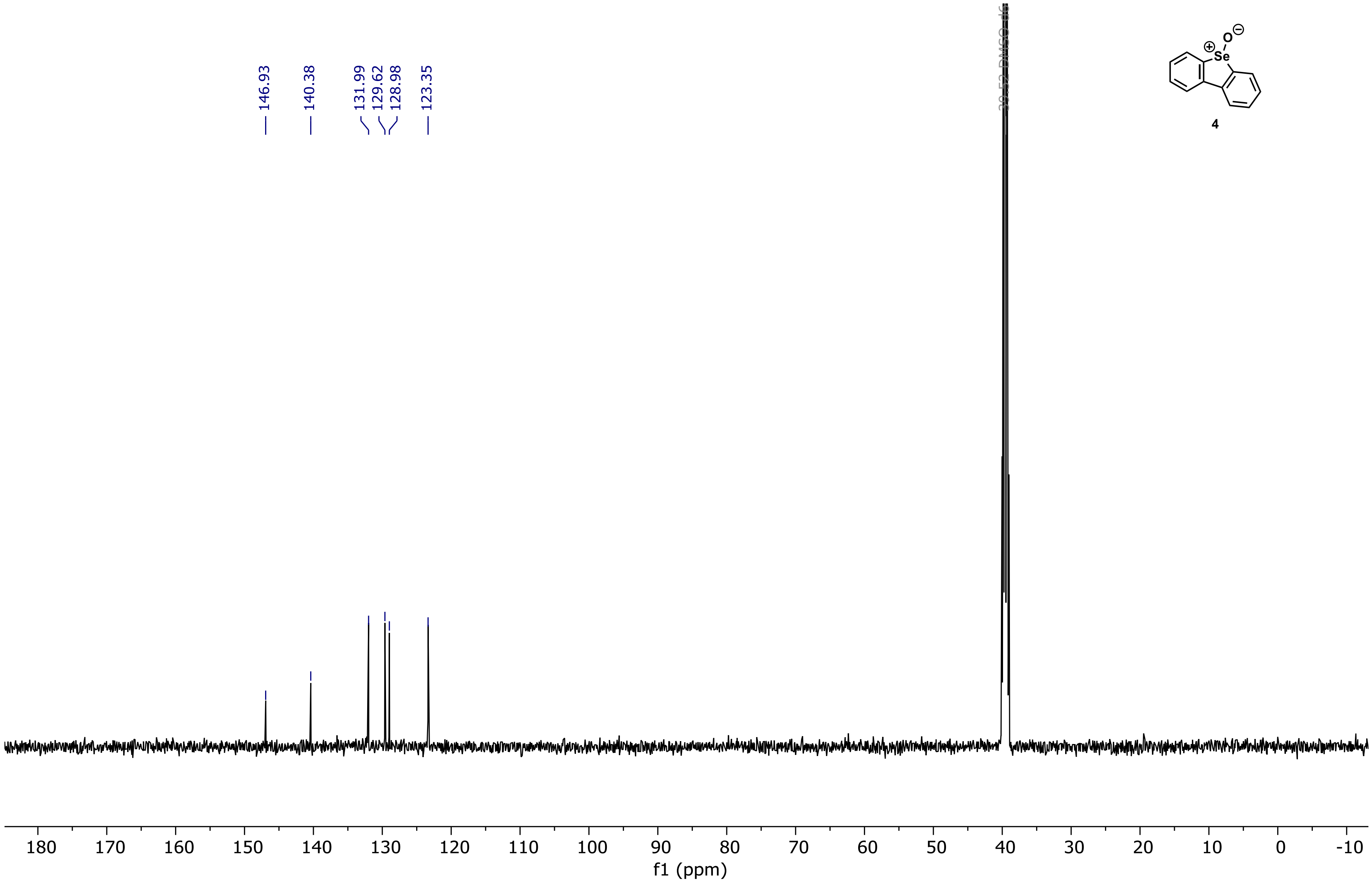

^{77}Se NMR of 4

DMSO-d_6, 115 MHz, 298 K

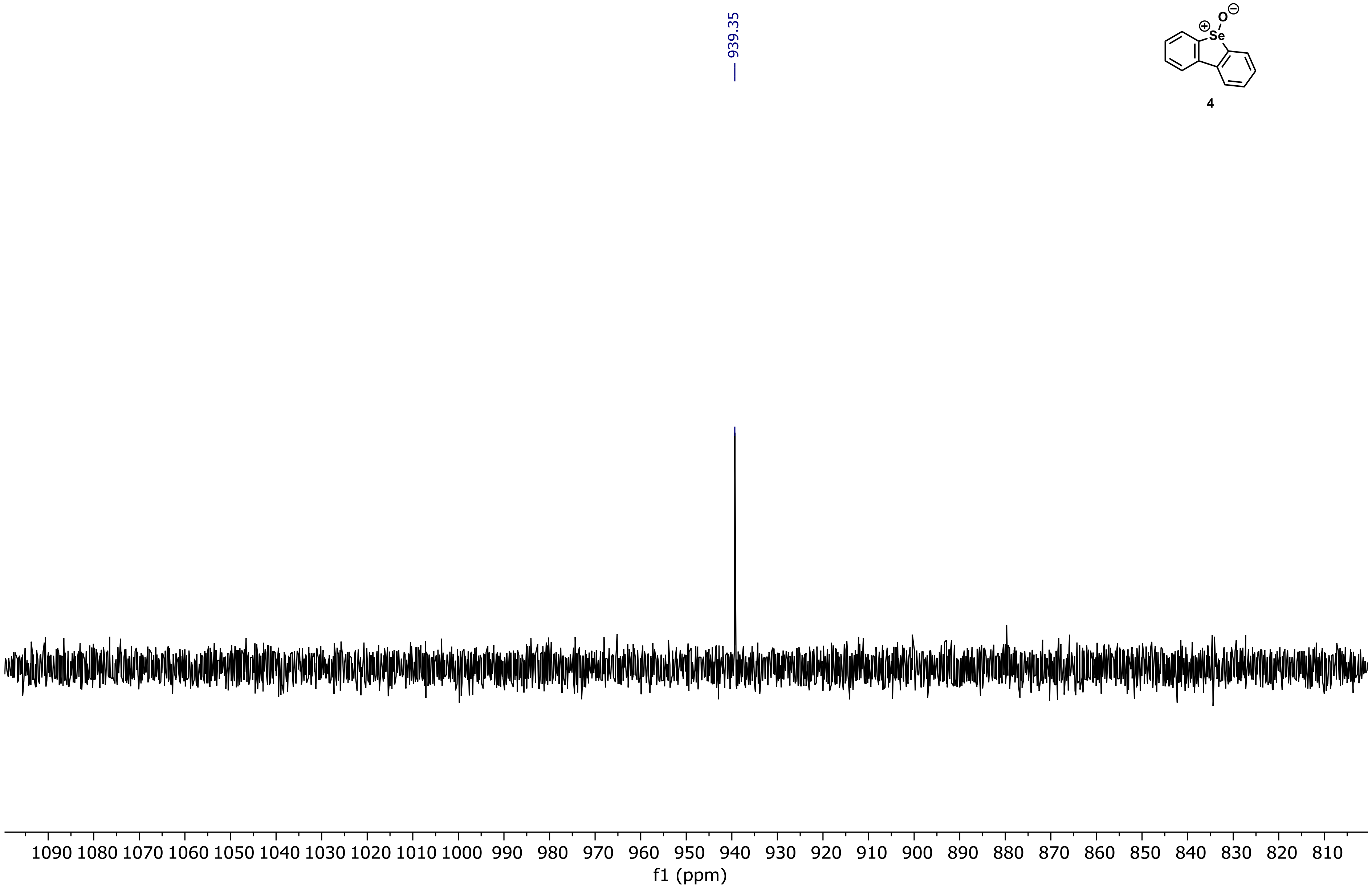

^{1}H NMR of S2

DMSO-d_6, 500 MHz, 298 K

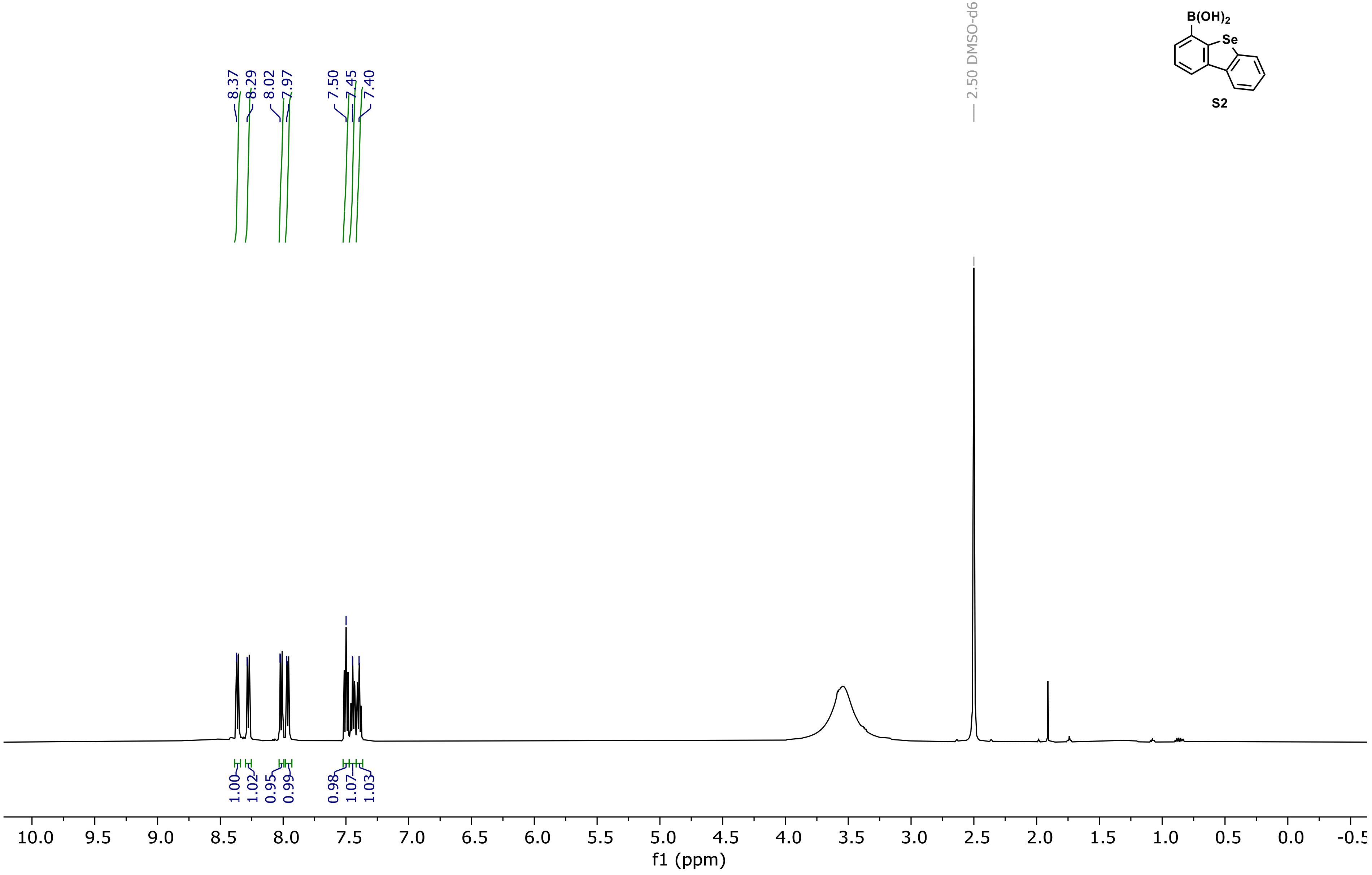

^{13}C NMR of S2

DMSO-d_6, 126 MHz, 298 K

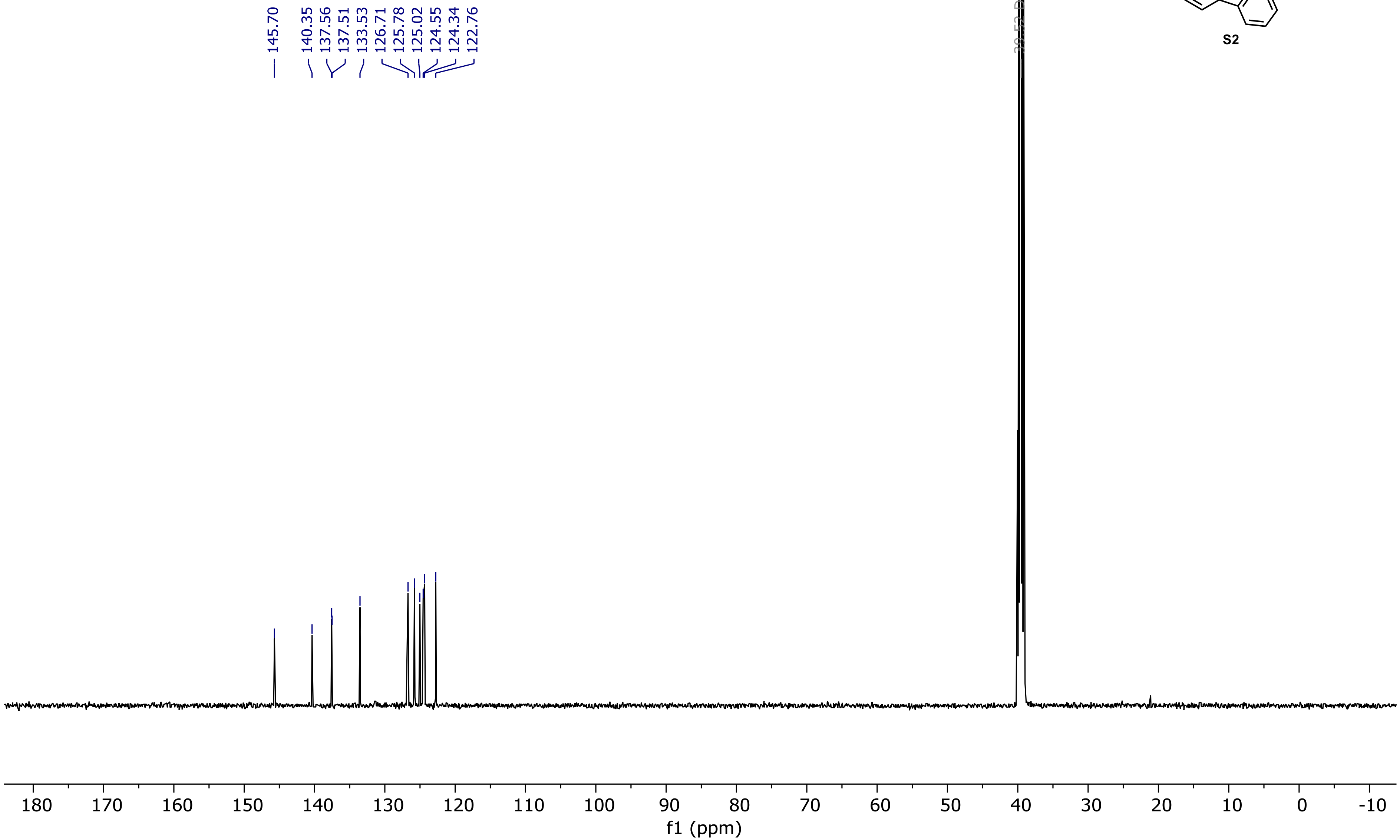

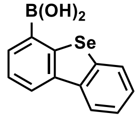

S2

^{77}Se NMR of S2

DMSO-d_6, 95 MHz, 298 K

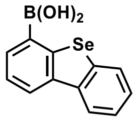

S2

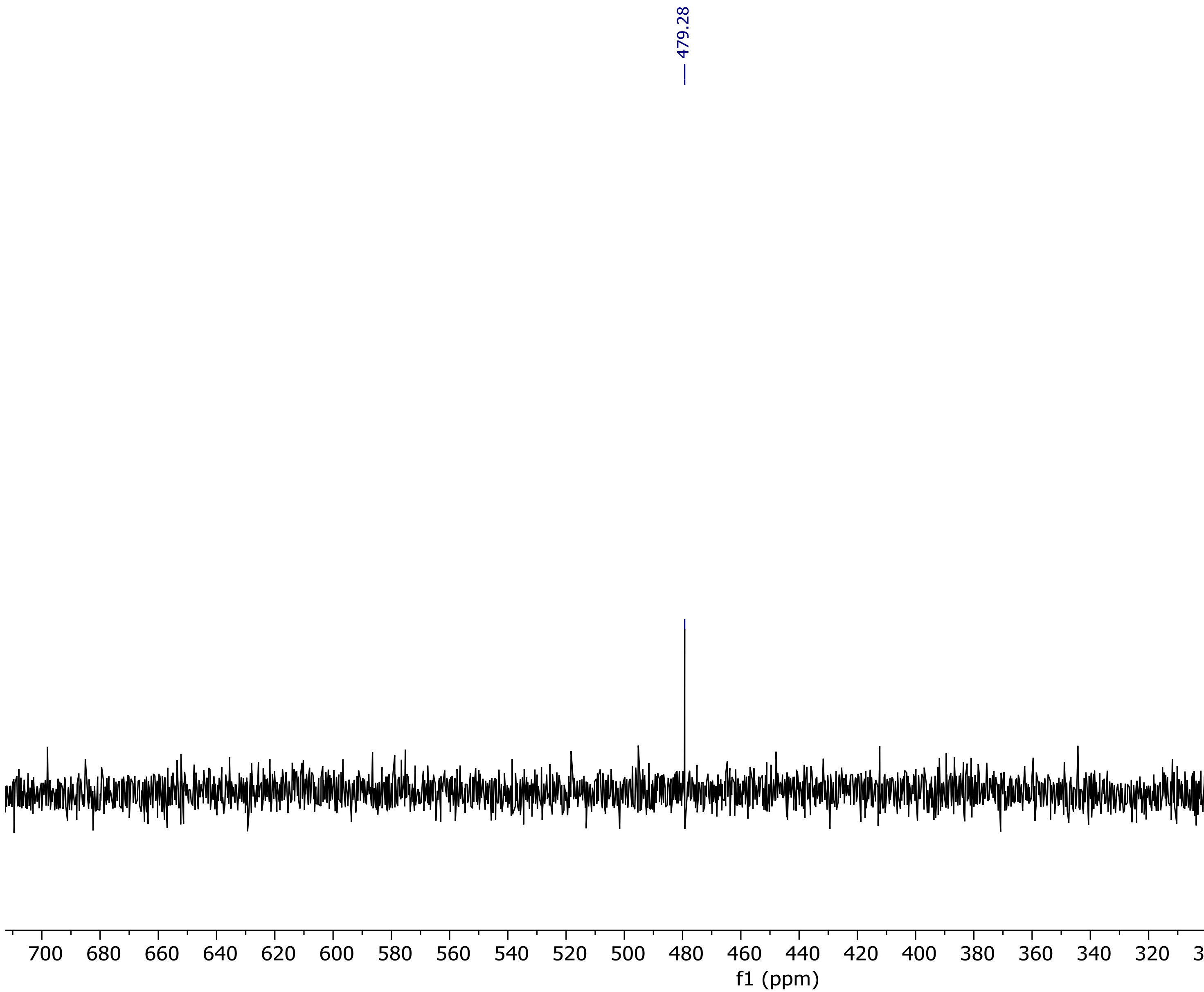

^{1}H NMR of S3

CDCl$_3$, 600 MHz, 298 K

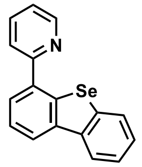

S3

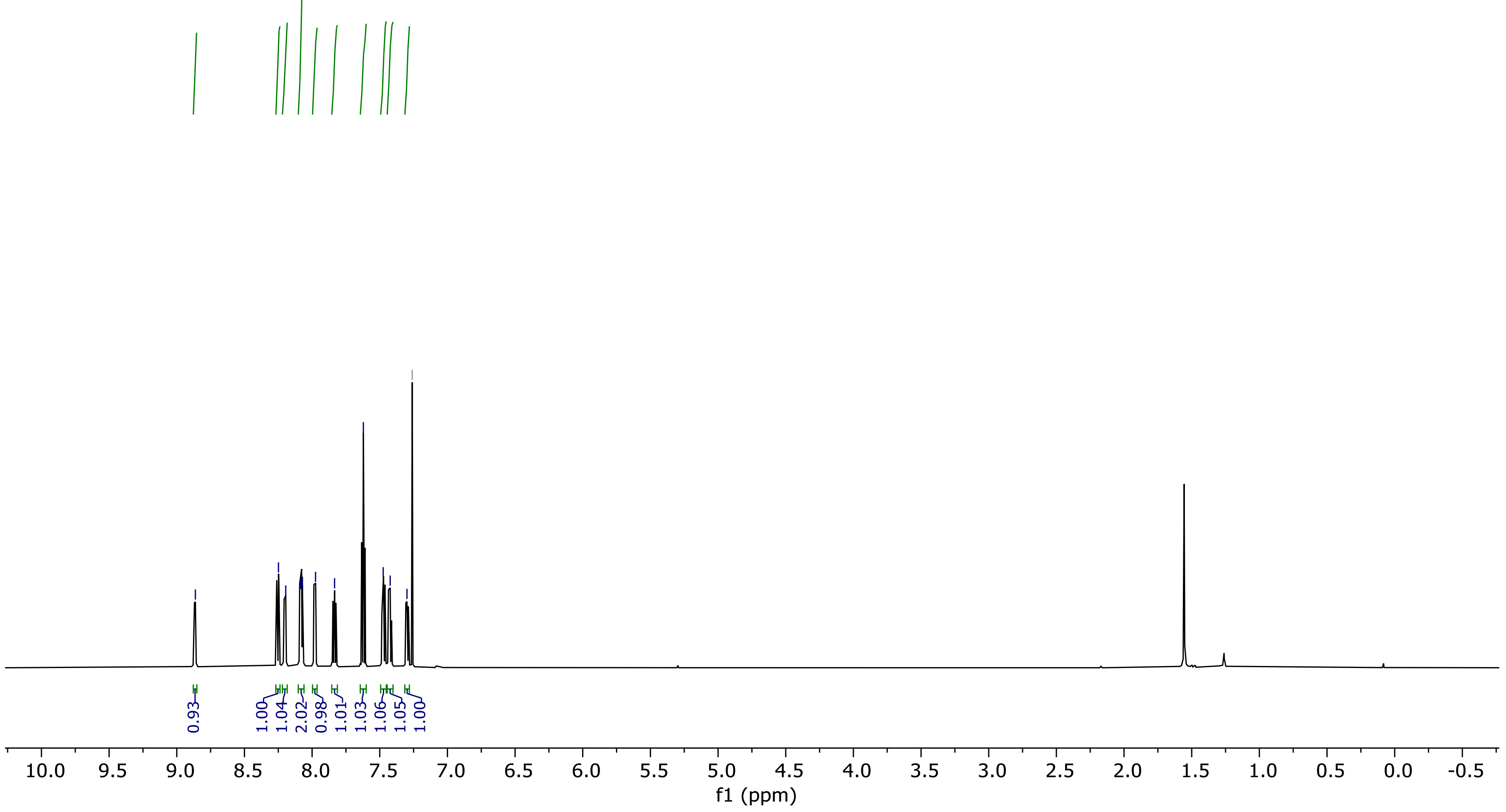

^{13}C NMR of S3

CDCl$_3$, 151 MHz, 298 K

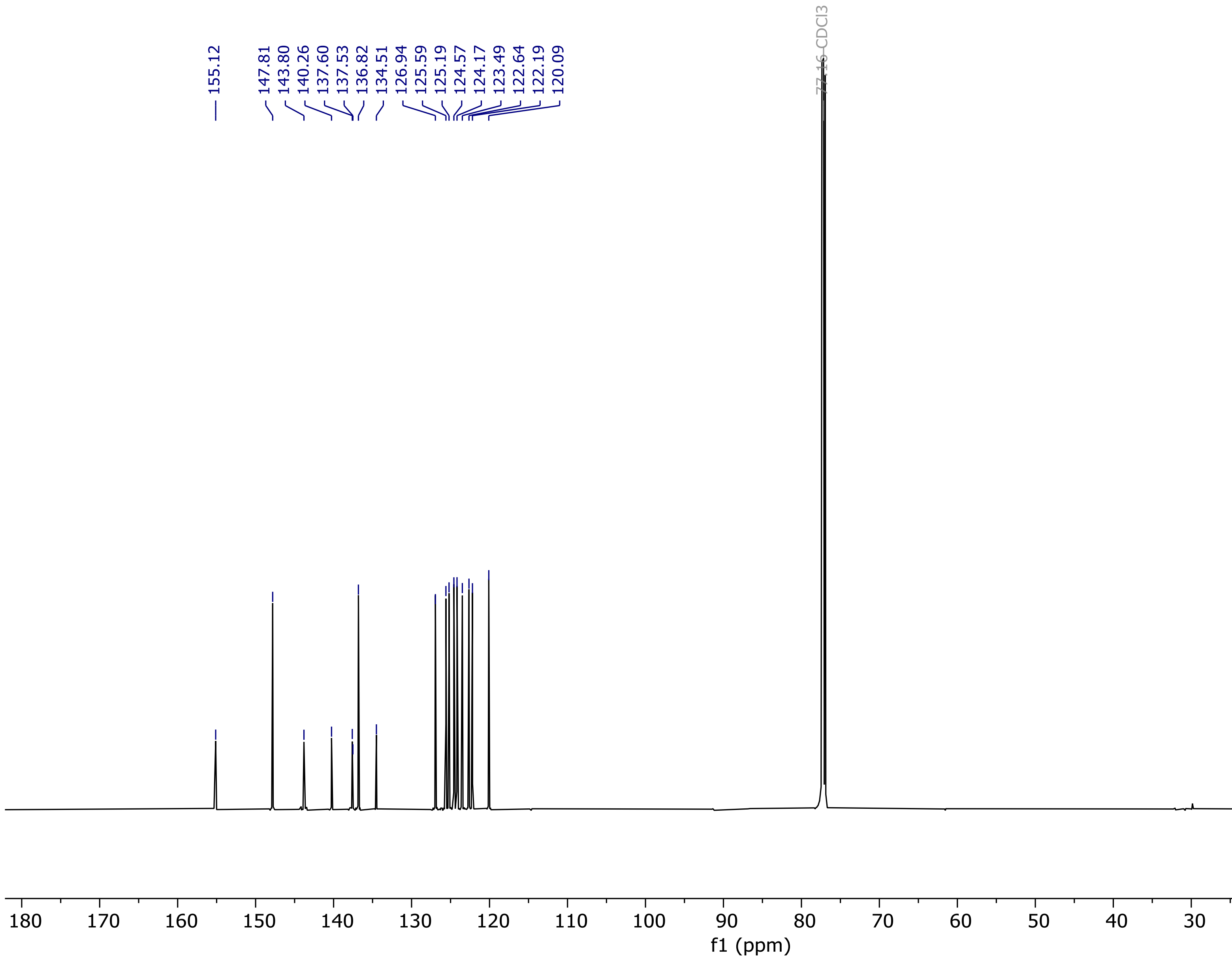

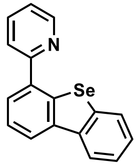

S3

^{77}Se NMR of S3

$CDCl_3$, 115 MHz, 298 K

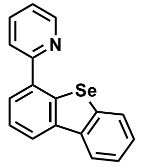

S3

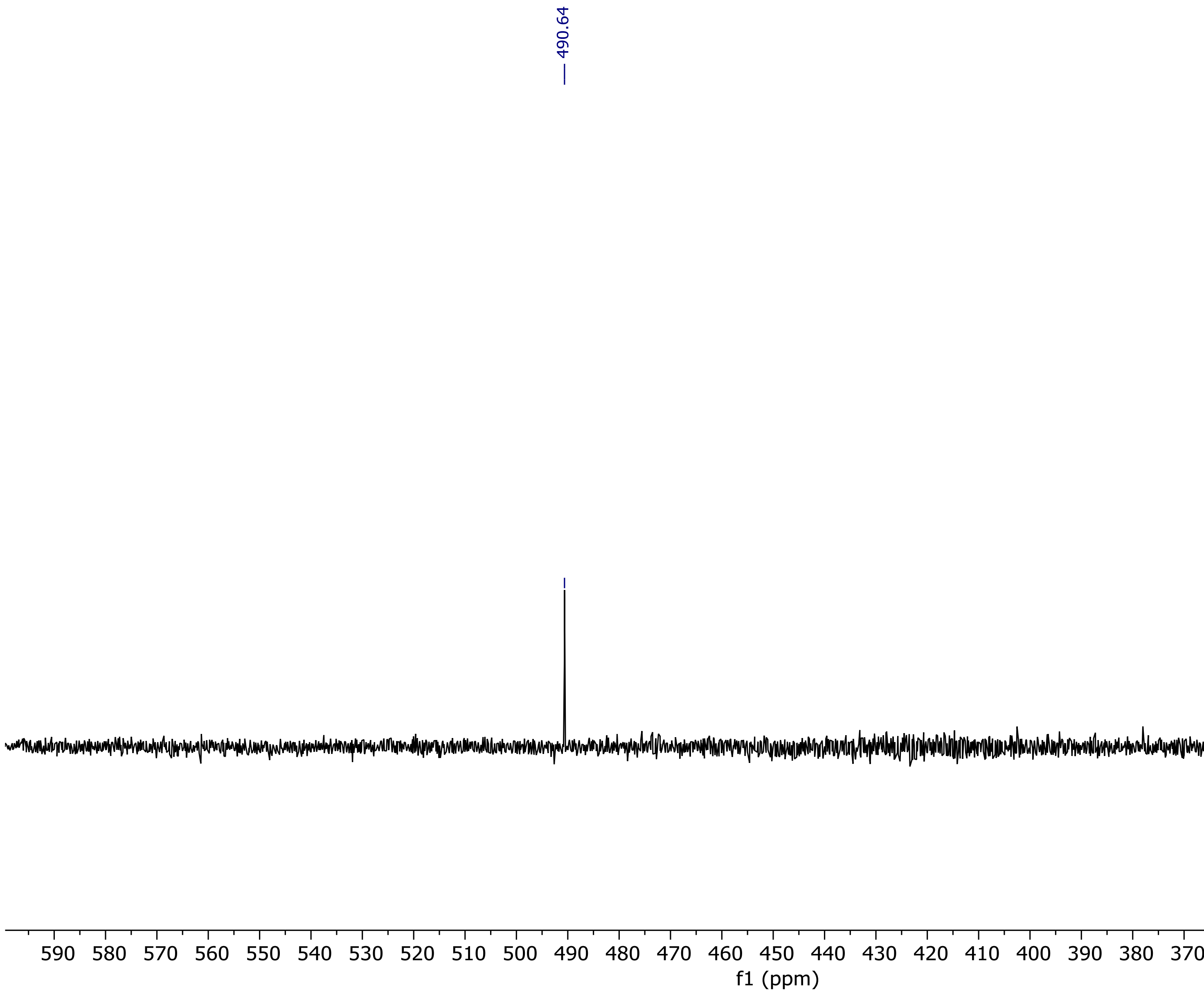

^{1}H NMR of 5

DMSO-d_6, 500 MHz, 298 K

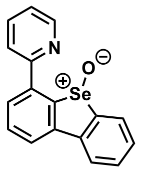

5

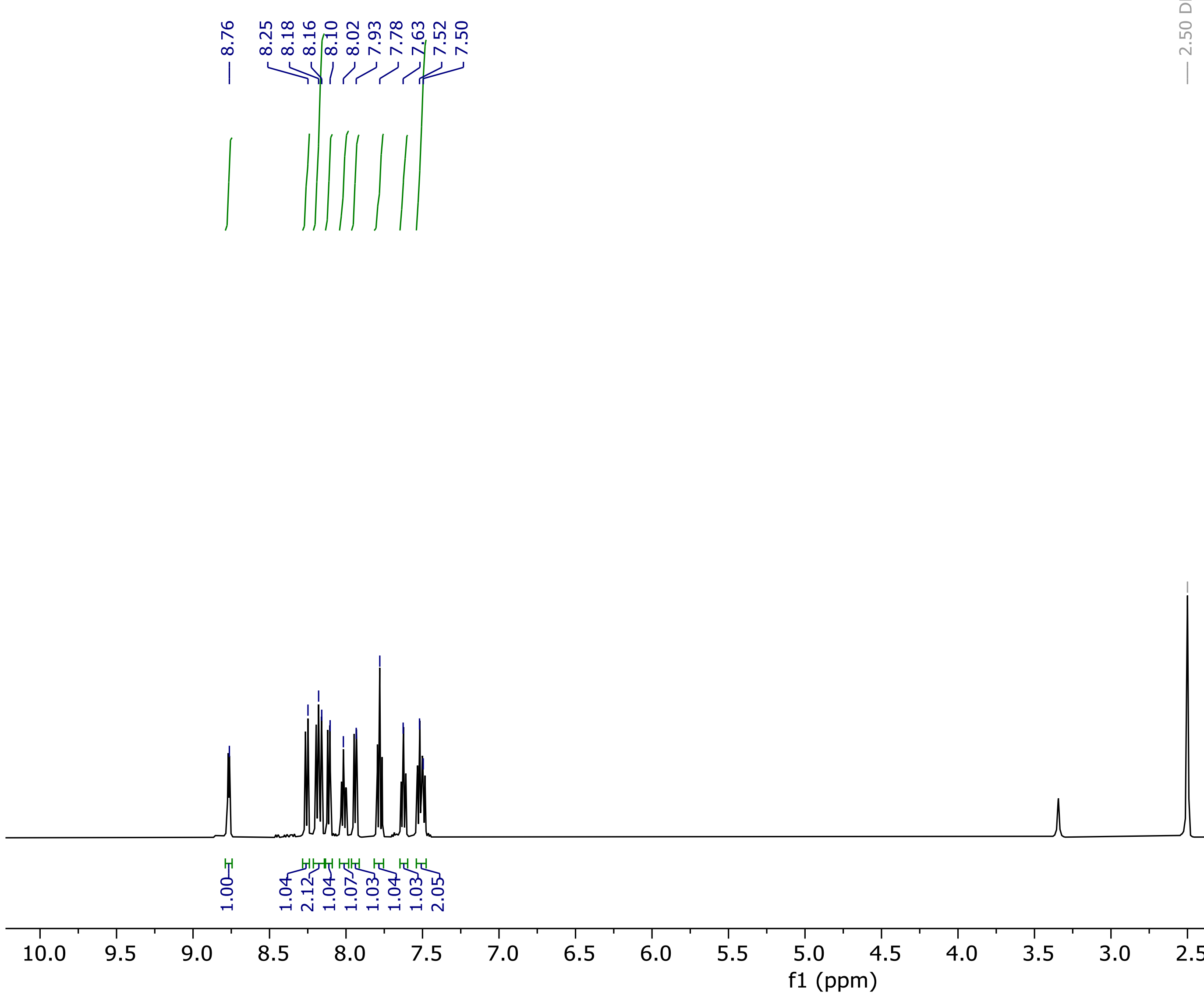

^{13}C NMR of 5

DMSO-d_6, 126 MHz, 298 K

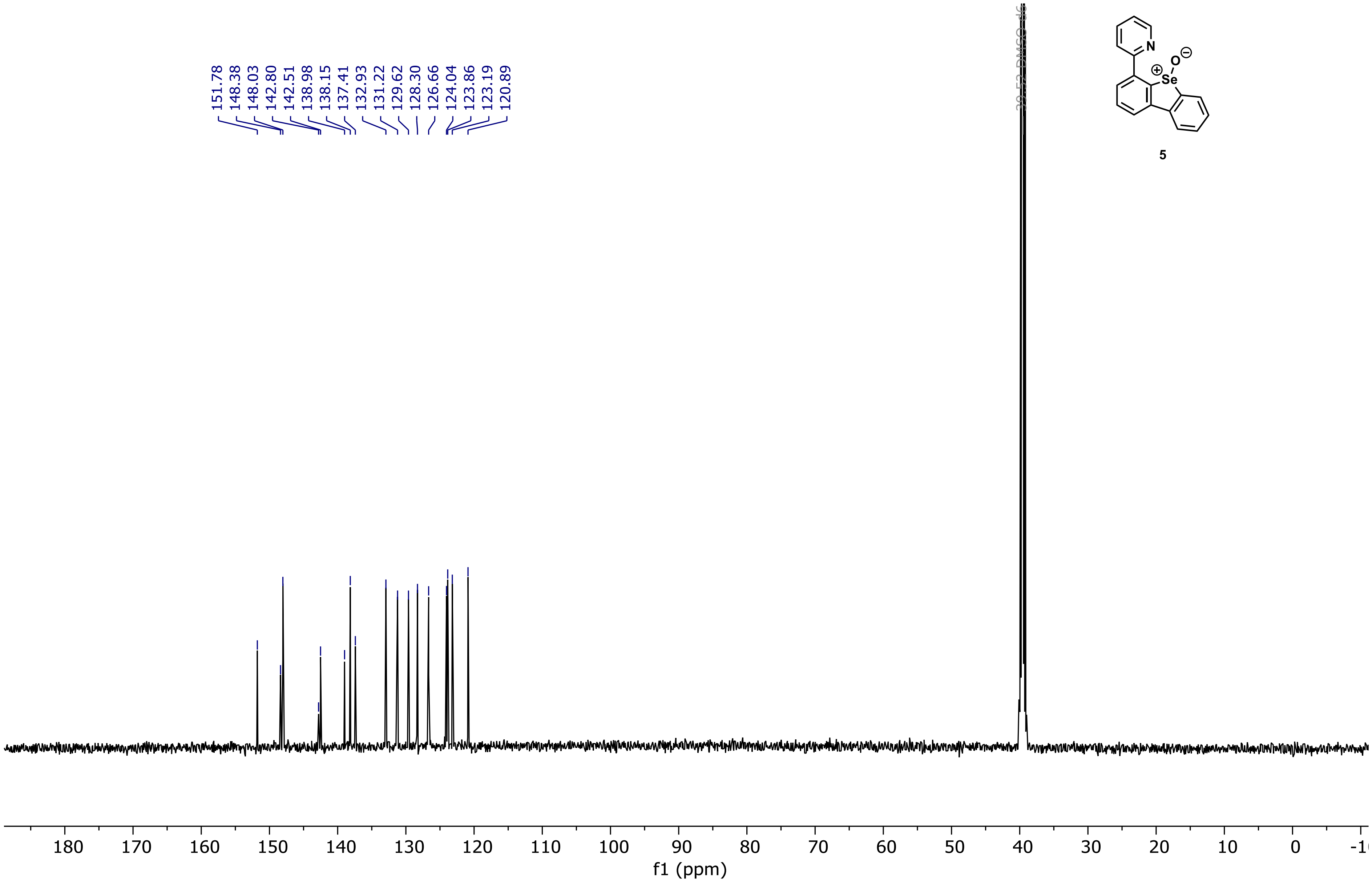

^{77}Se NMR of 5

DMSO-d_6, 115 MHz, 298 K

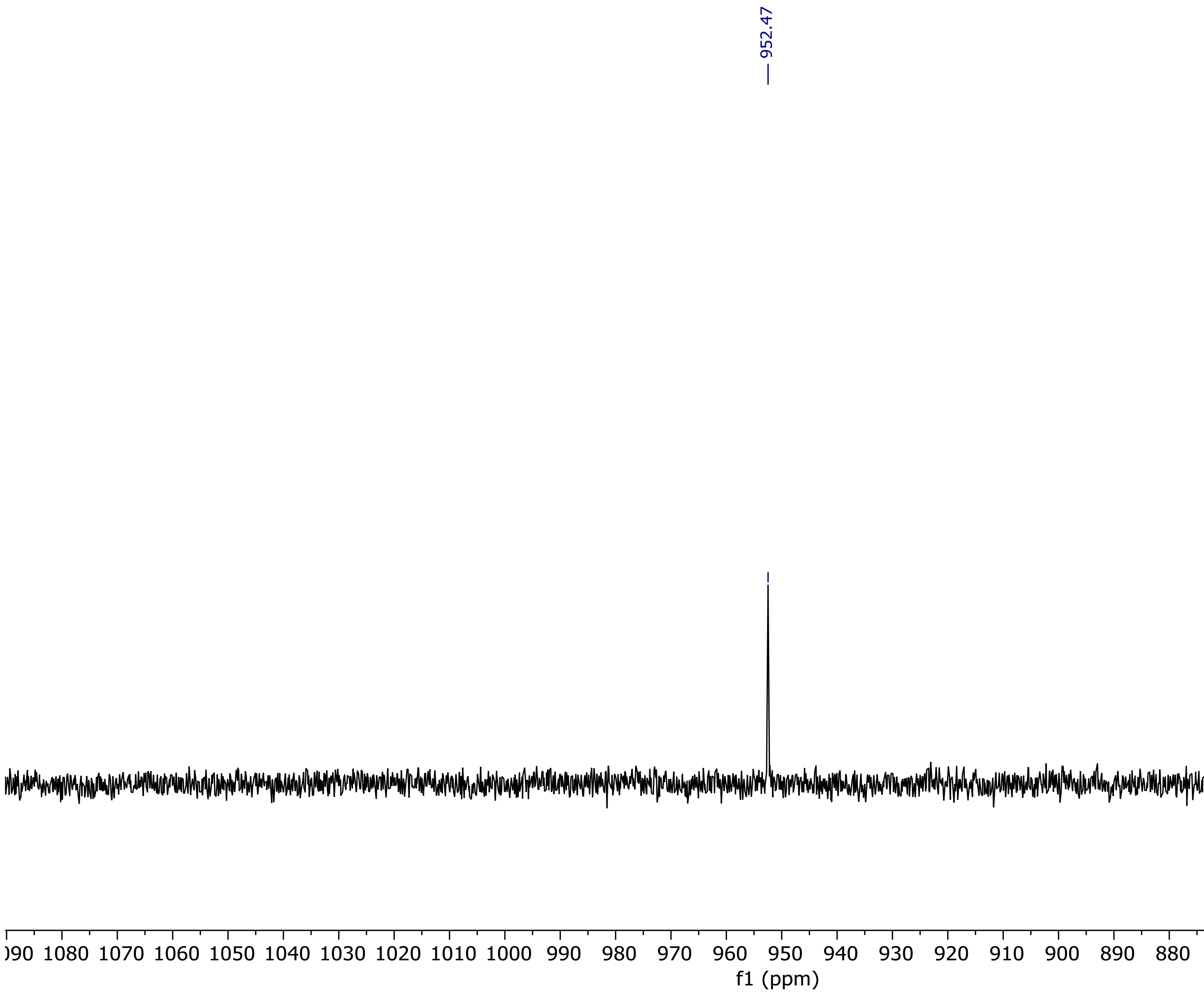

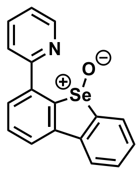

5

^{1}H NMR of S4

$CDCl_3$, 500 MHz, 298 K

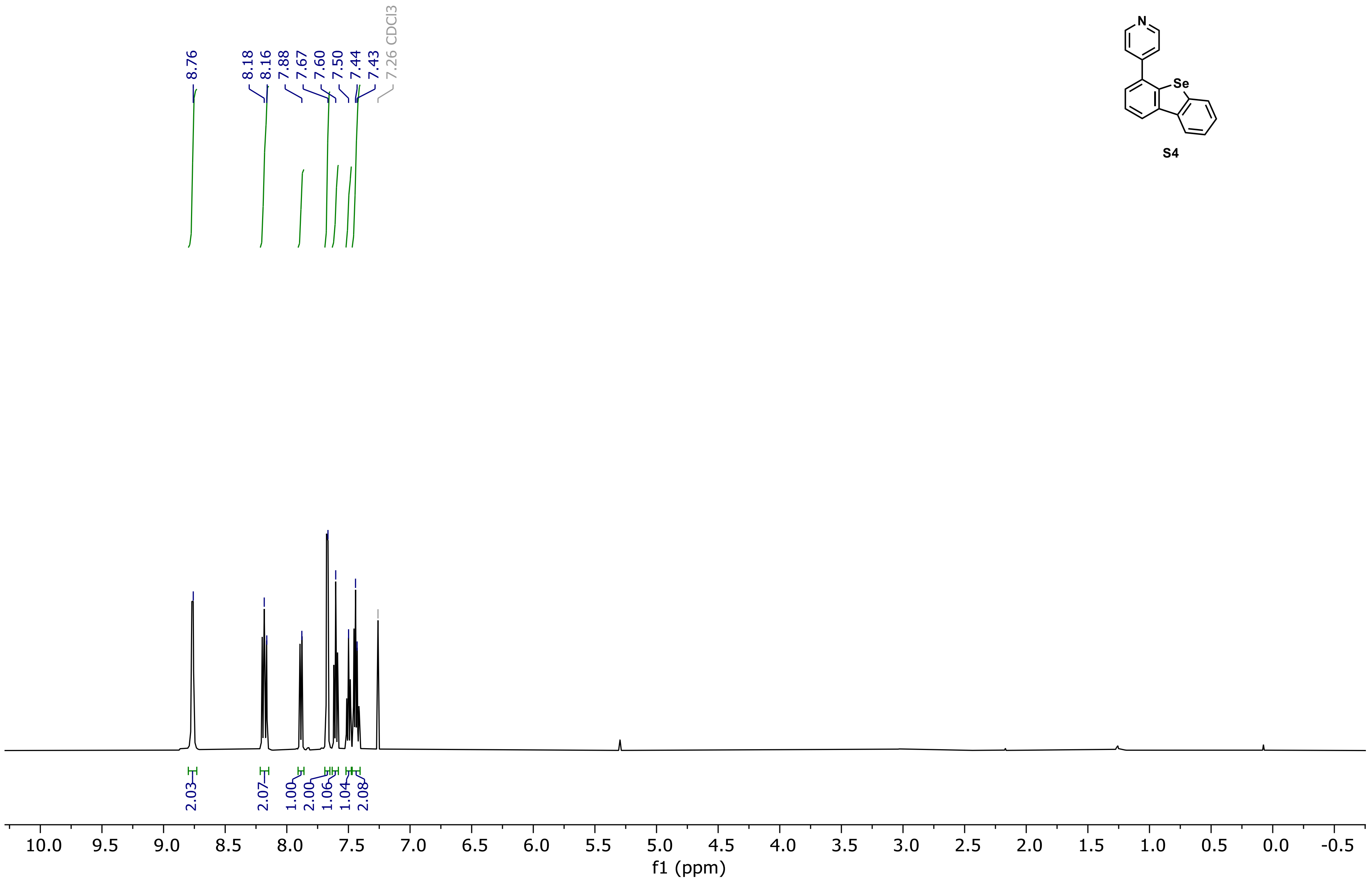

S4

^{13}C NMR of S4

$CDCl_3$, 126 MHz, 298 K

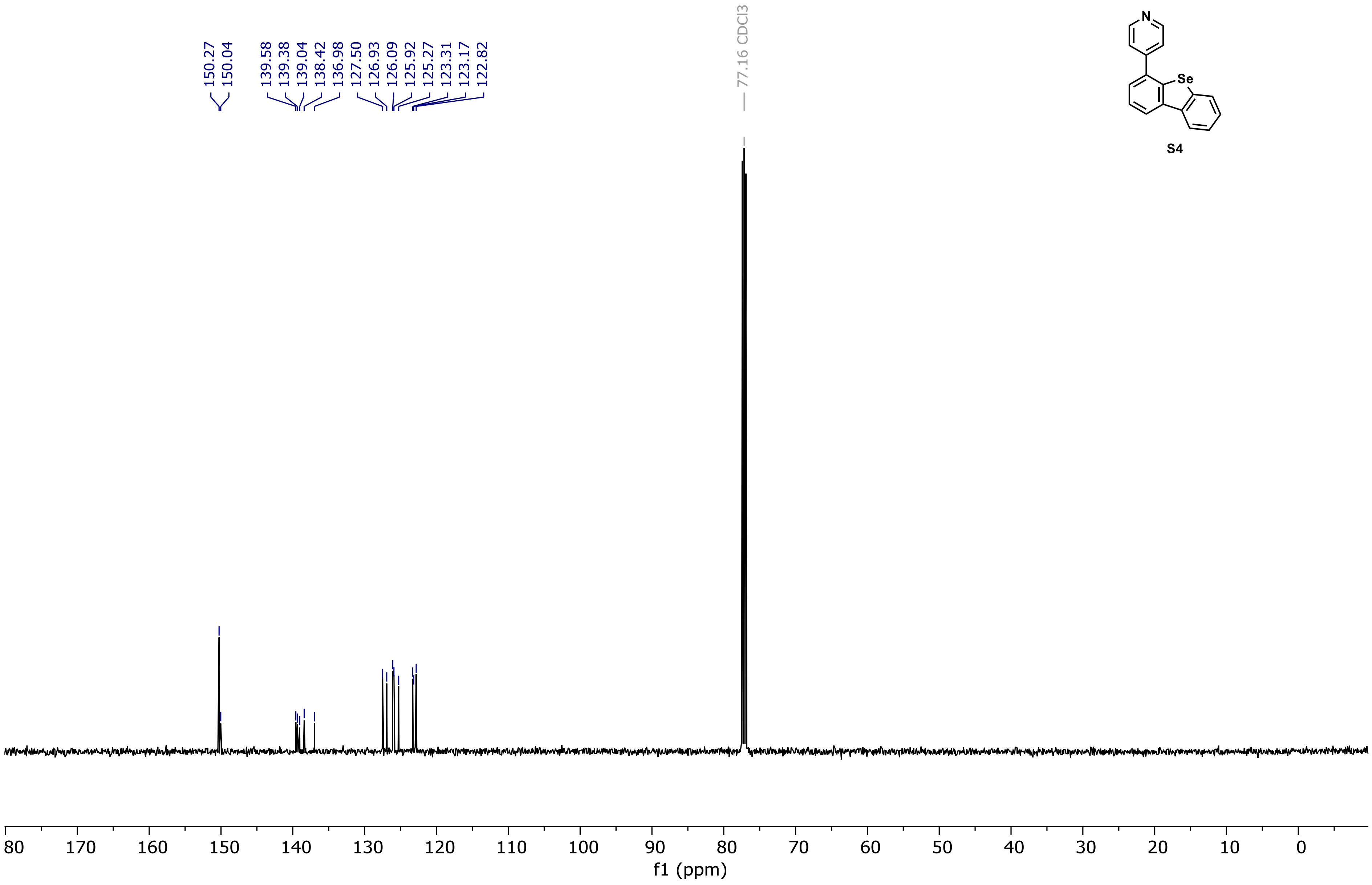

S4

^{77}Se NMR of S4

$CDCl_3$, 115 MHz, 298 K

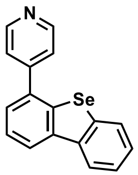

S4

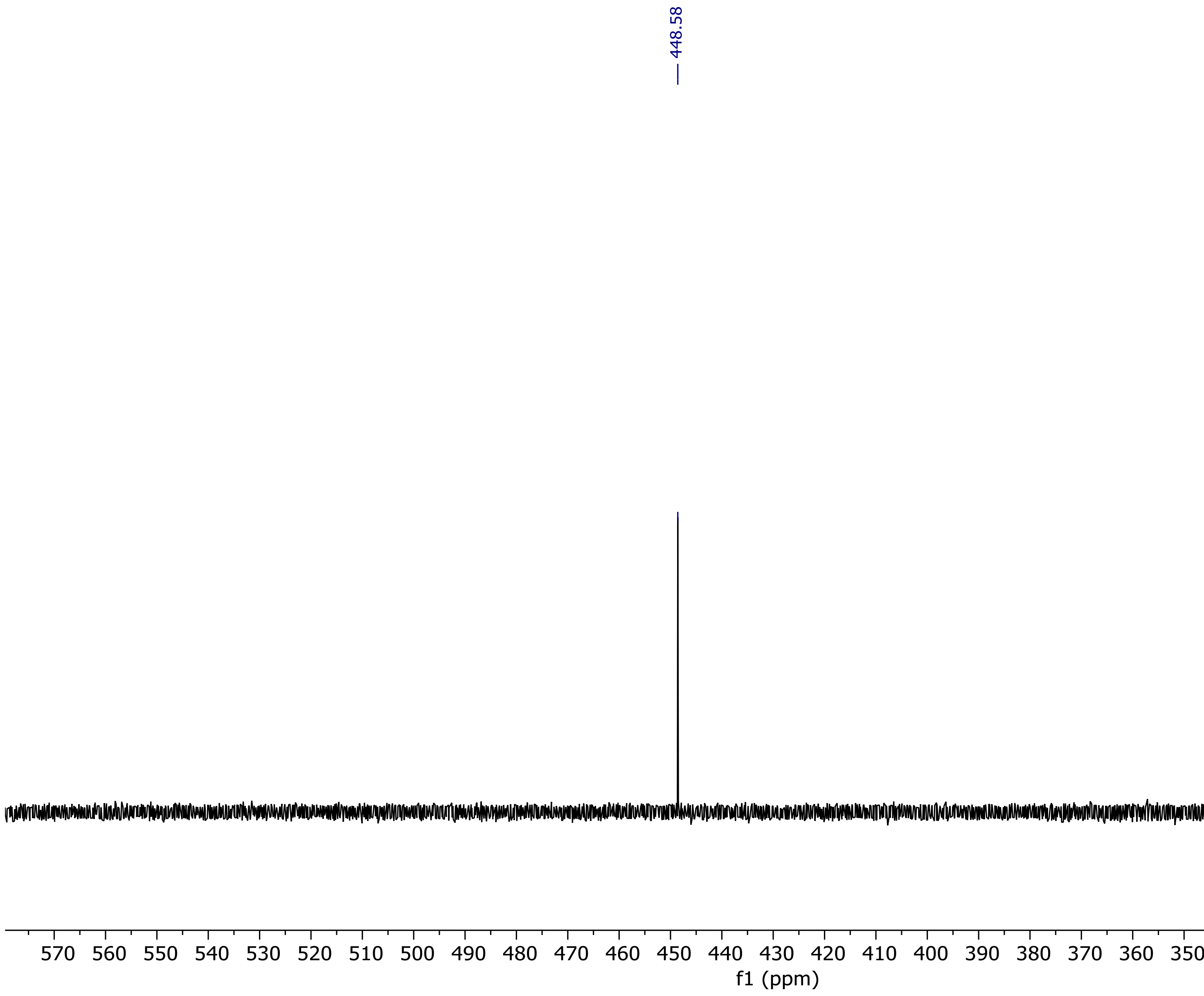

^{1}H NMR of S5

CD3OD, 500 MHz, 298 K

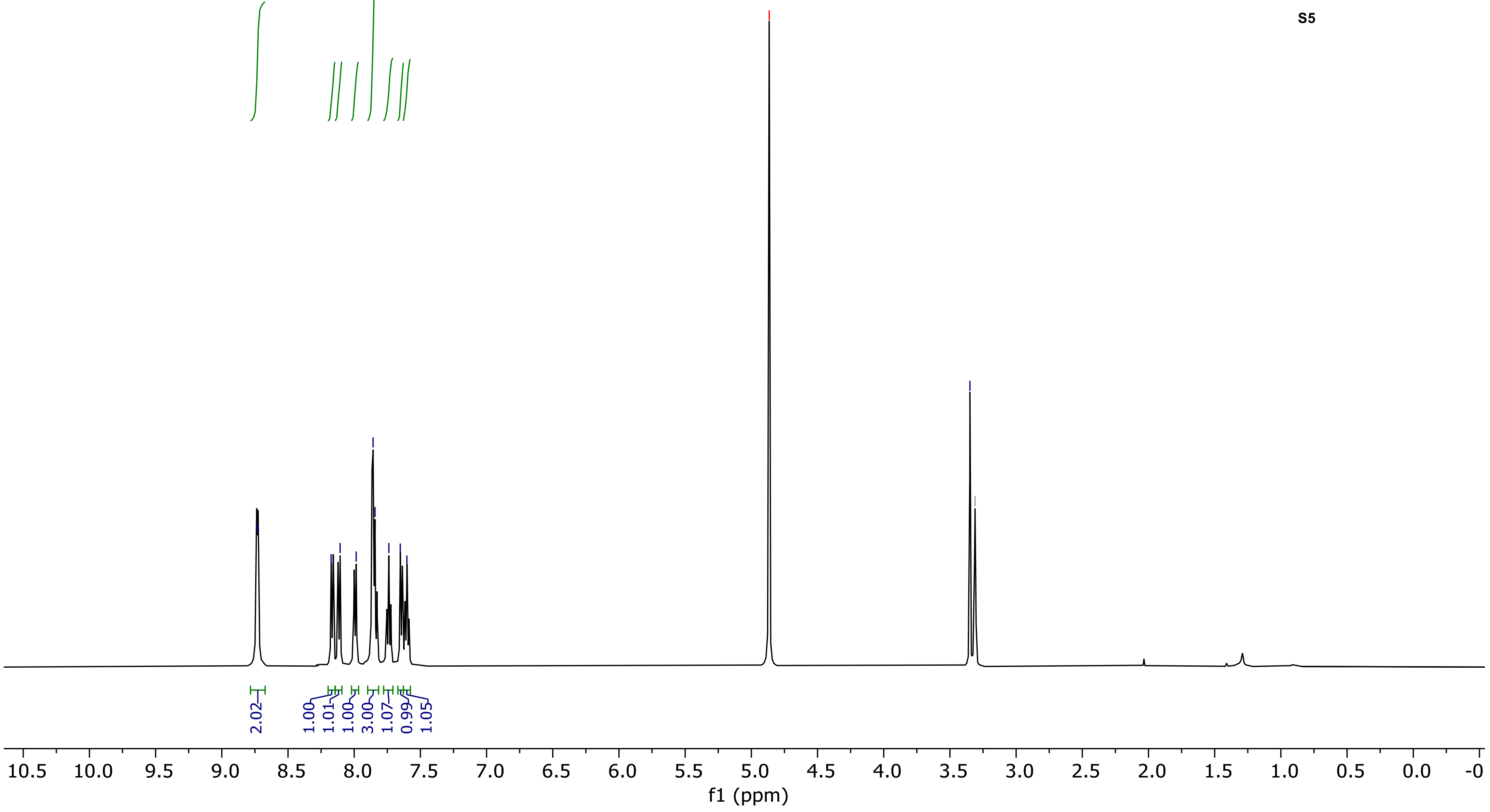

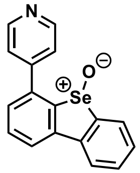

S5

^{13}C NMR of S5

CD_3OD, 126 MHz, 298 K

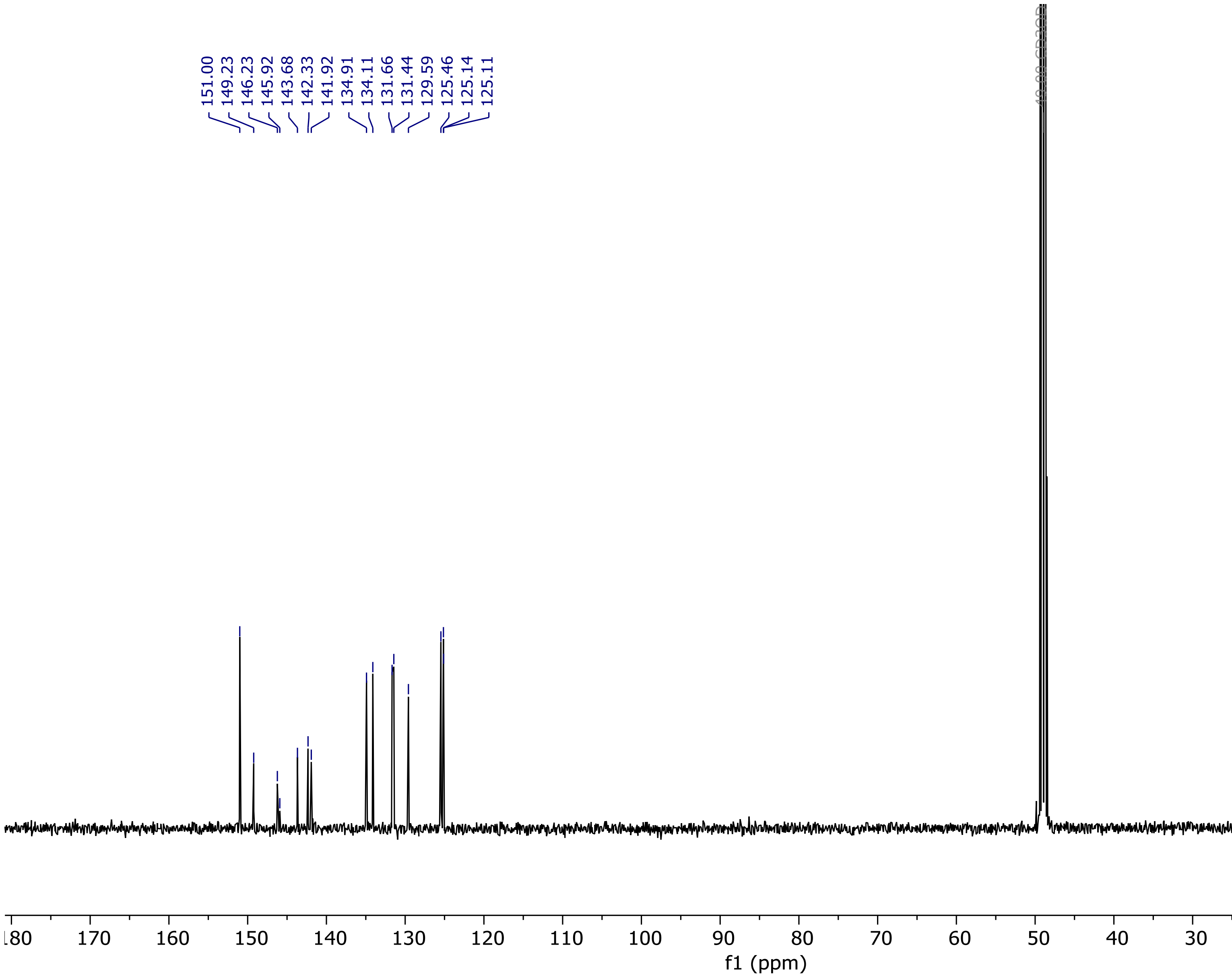

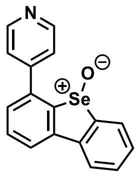

S5

^{77}Se NMR of S5

CD_3OD, 115 MHz, 298 K

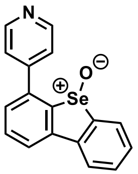

S5

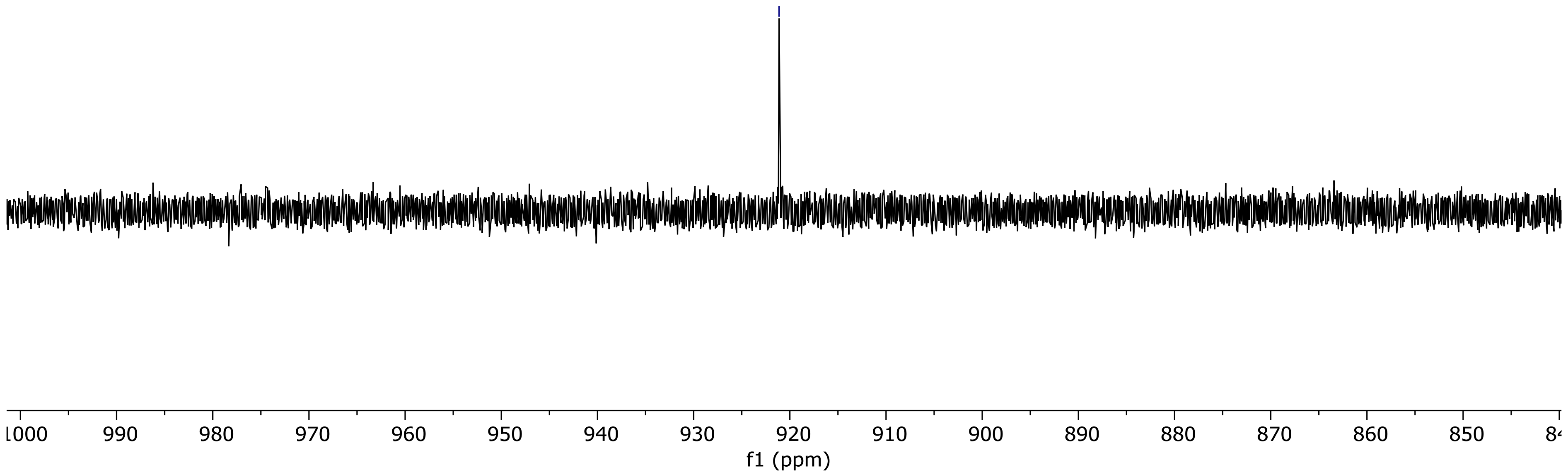

^{1}H NMR of S6

CDCl₃, 500 MHz, 298 K

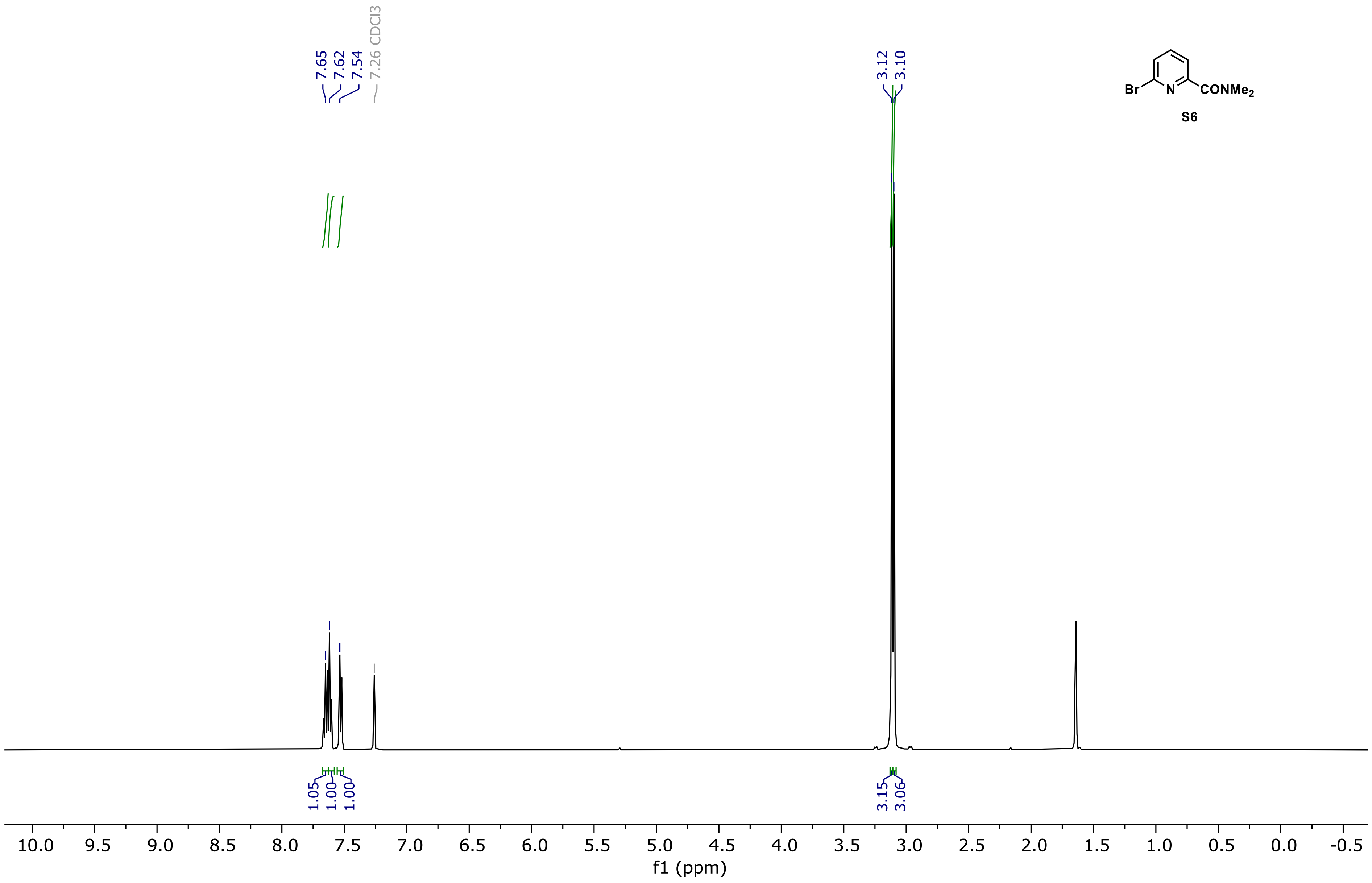

^{13}C NMR of S6

$CDCl_3$, 126 MHz, 298 K

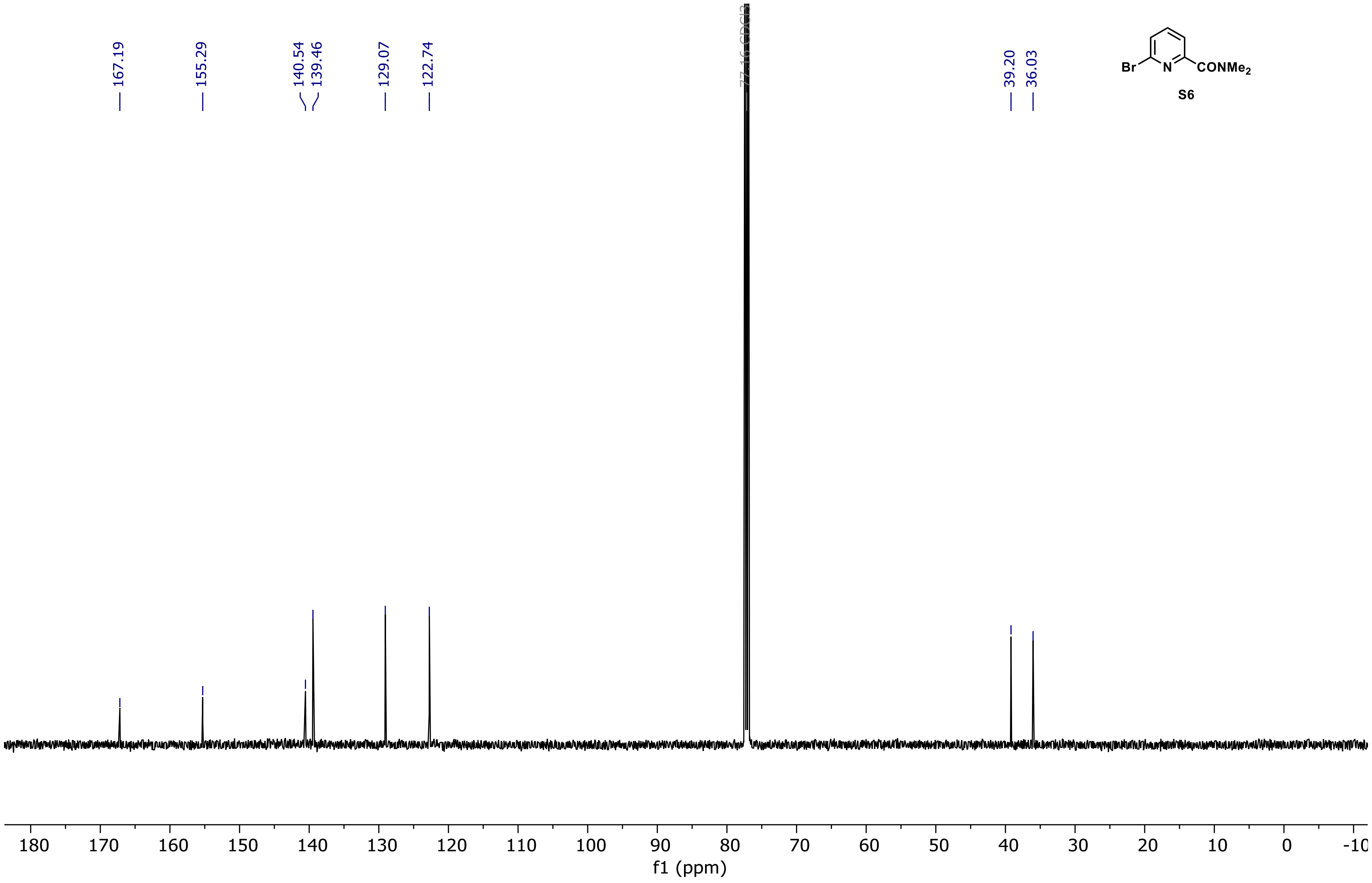

^{1}H NMR of S7

$CDCl_3$, 500 MHz, 298 K

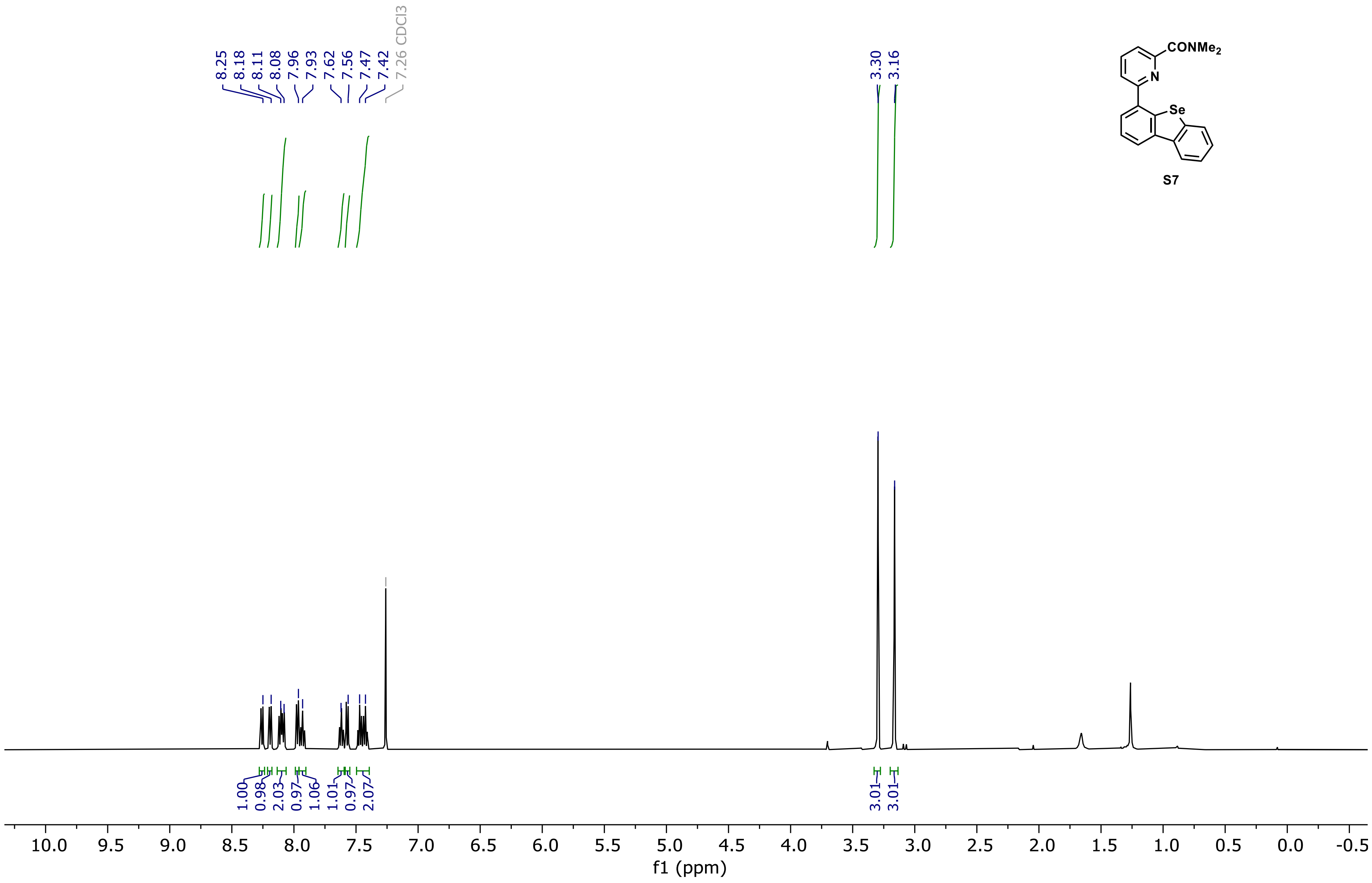

S7

^{13}C NMR of S7

$CDCl_3$, 126 MHz, 298 K

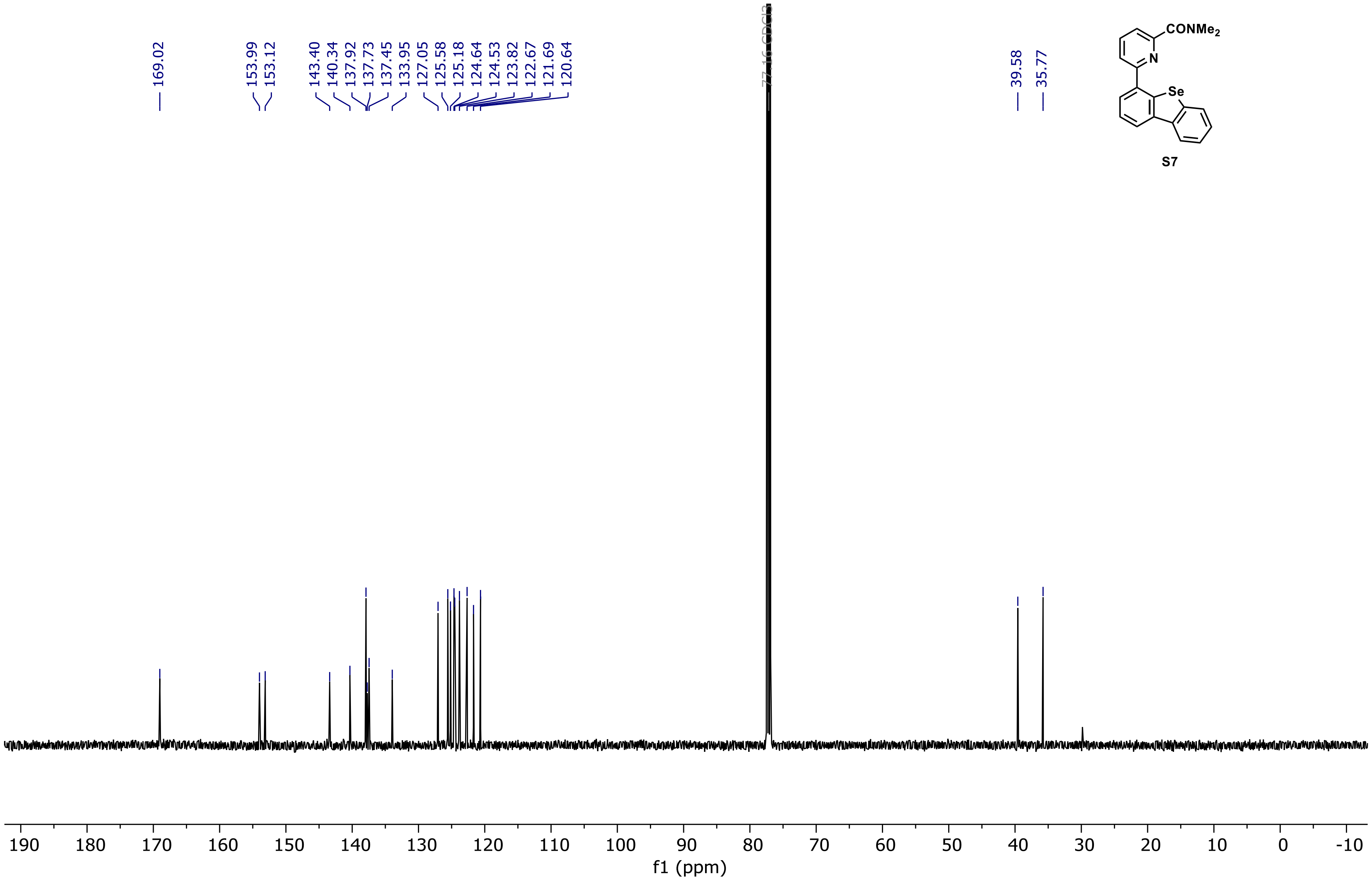

S7

^{77}Se NMR of S7

$CDCl_3$, 115 MHz, 298 K

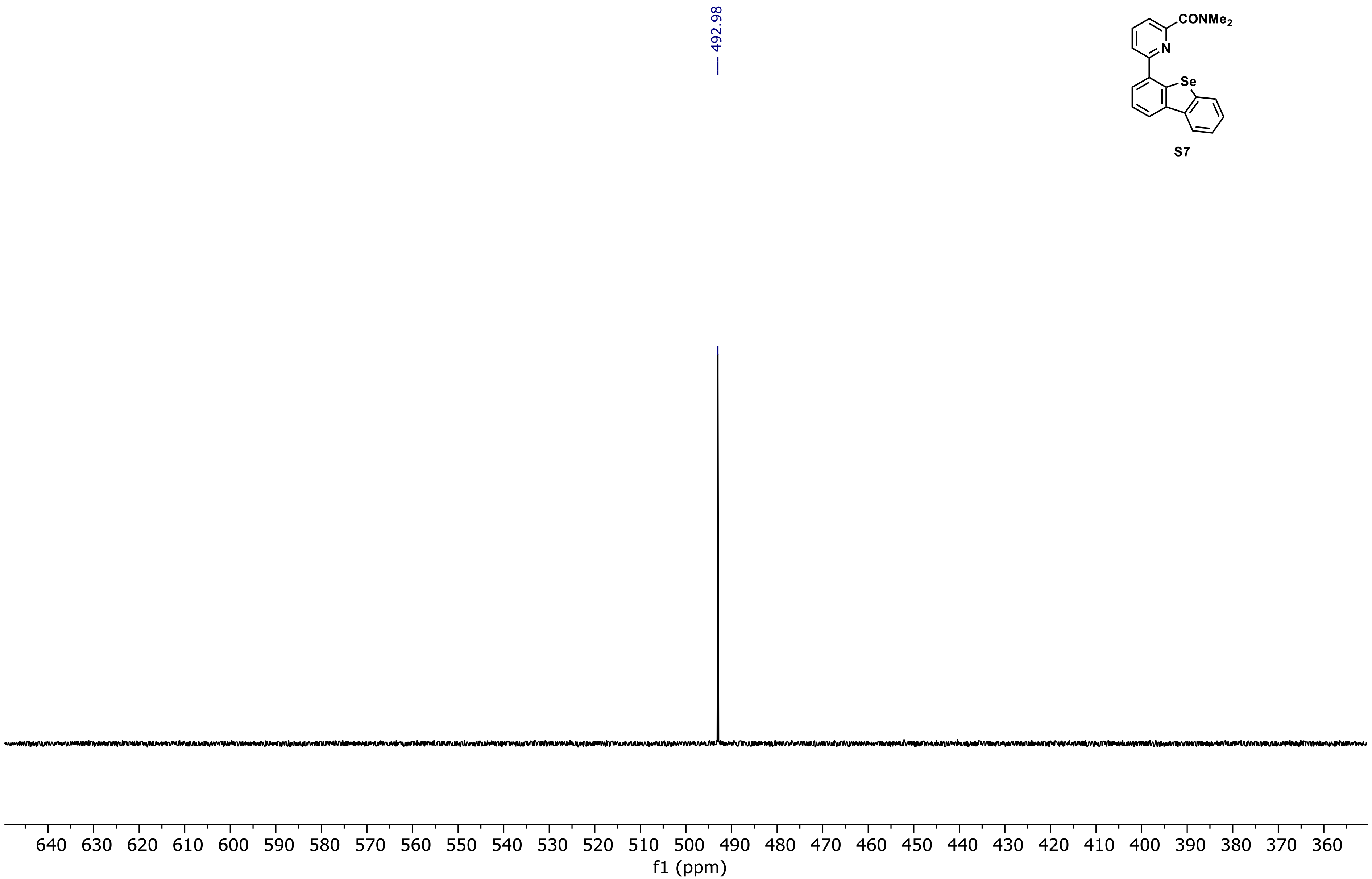

^{1}H NMR of 6

CD3OD, 600 MHz, 298 K

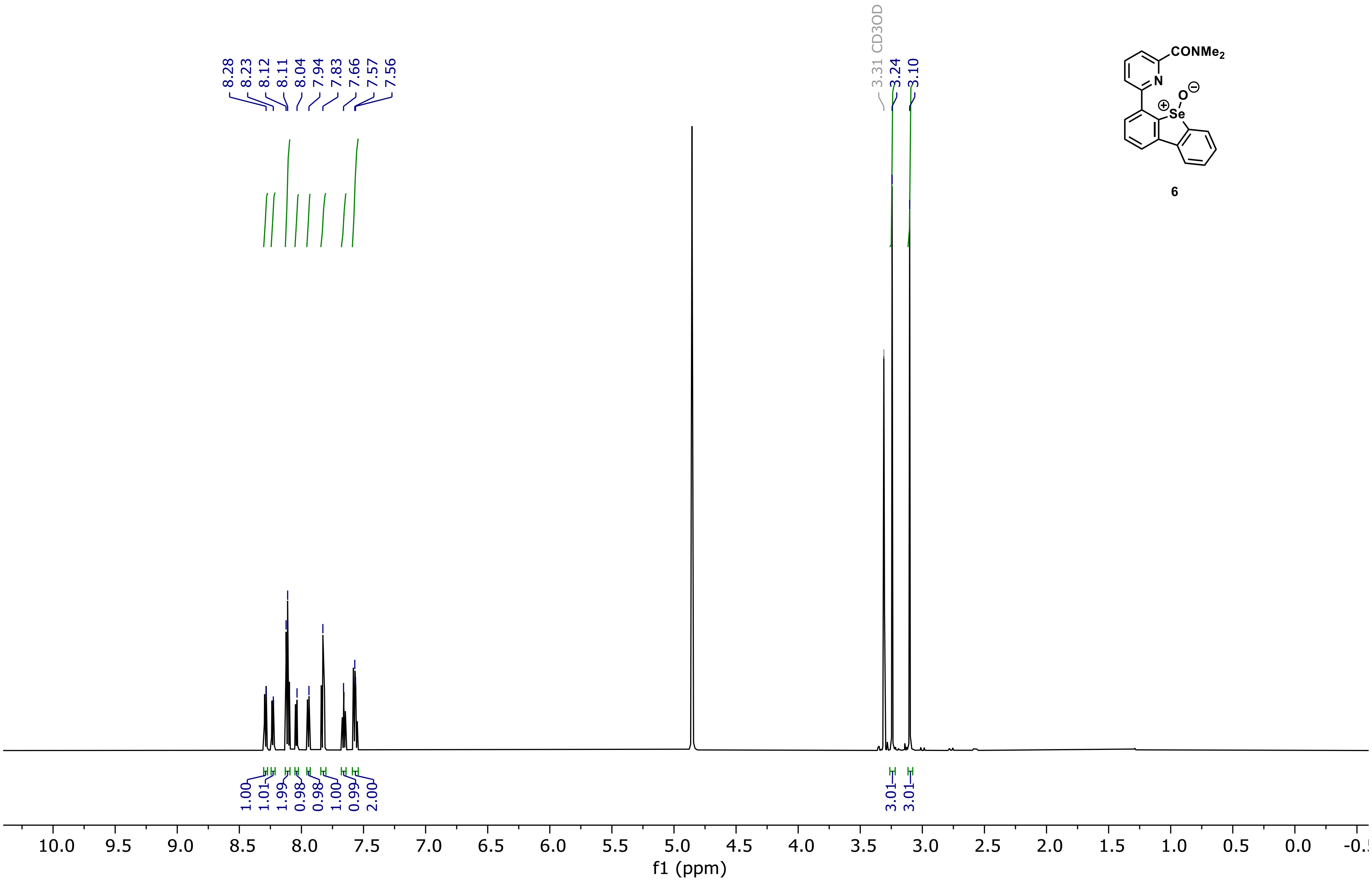

^{13}C NMR of 6

CD$_3$OD, 151 MHz, 298 K

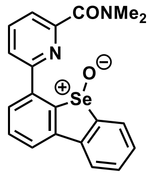

6

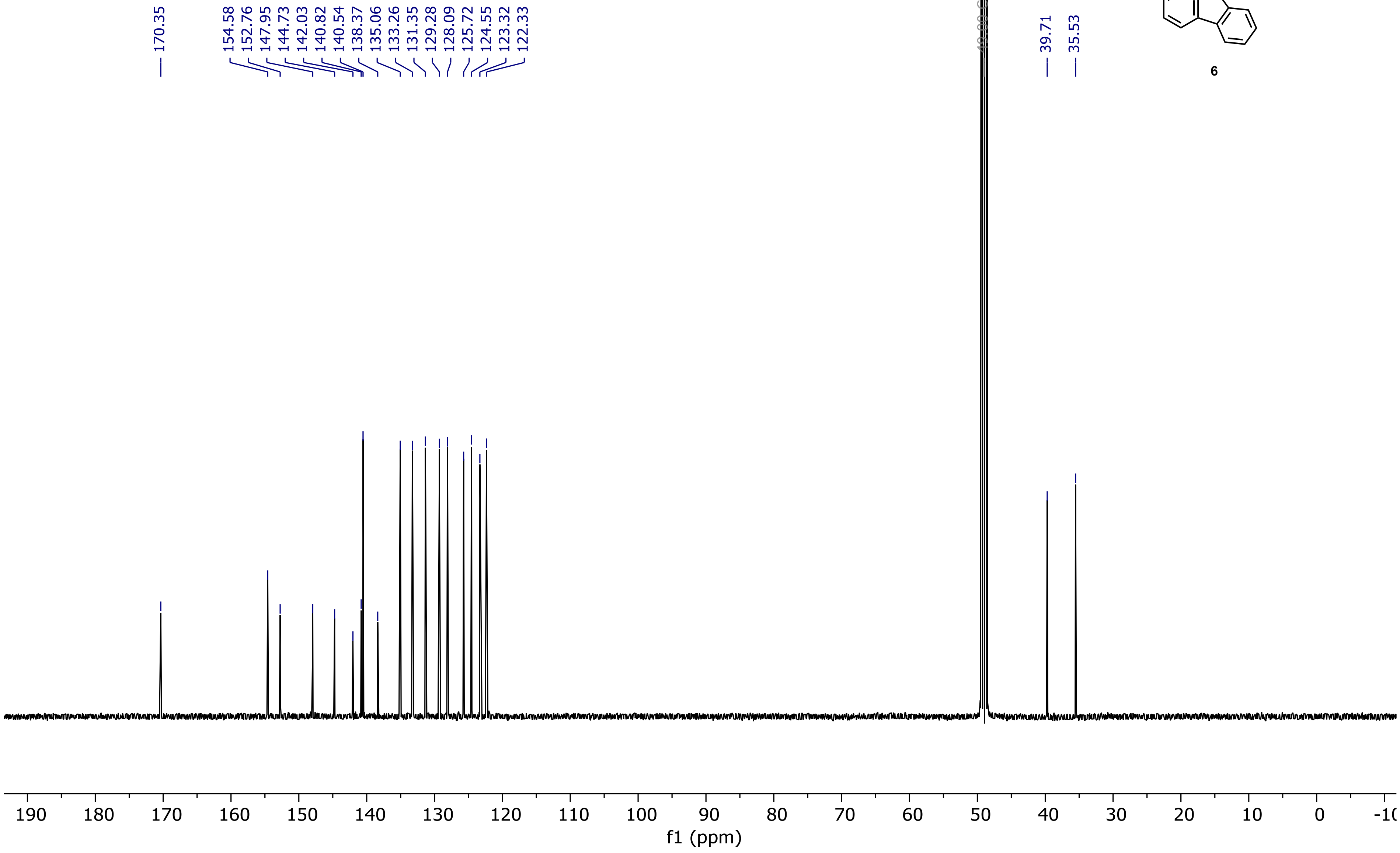

^{77}Se NMR of 6

CD3OD, 115 MHz, 298 K

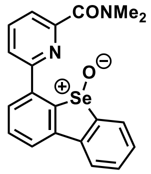

6

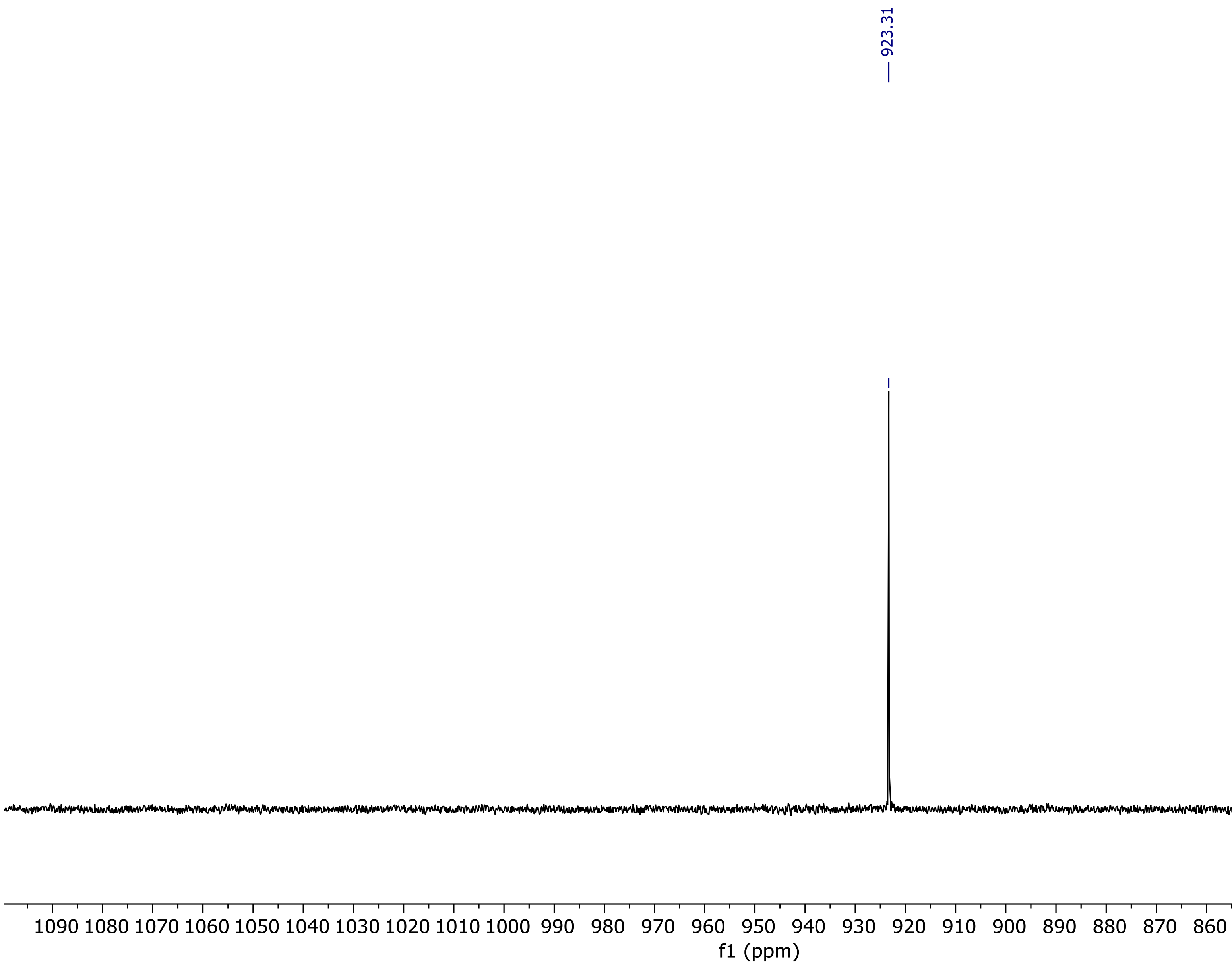

^{1}H NMR of S8

CDCl3, 500 MHz, 298 K

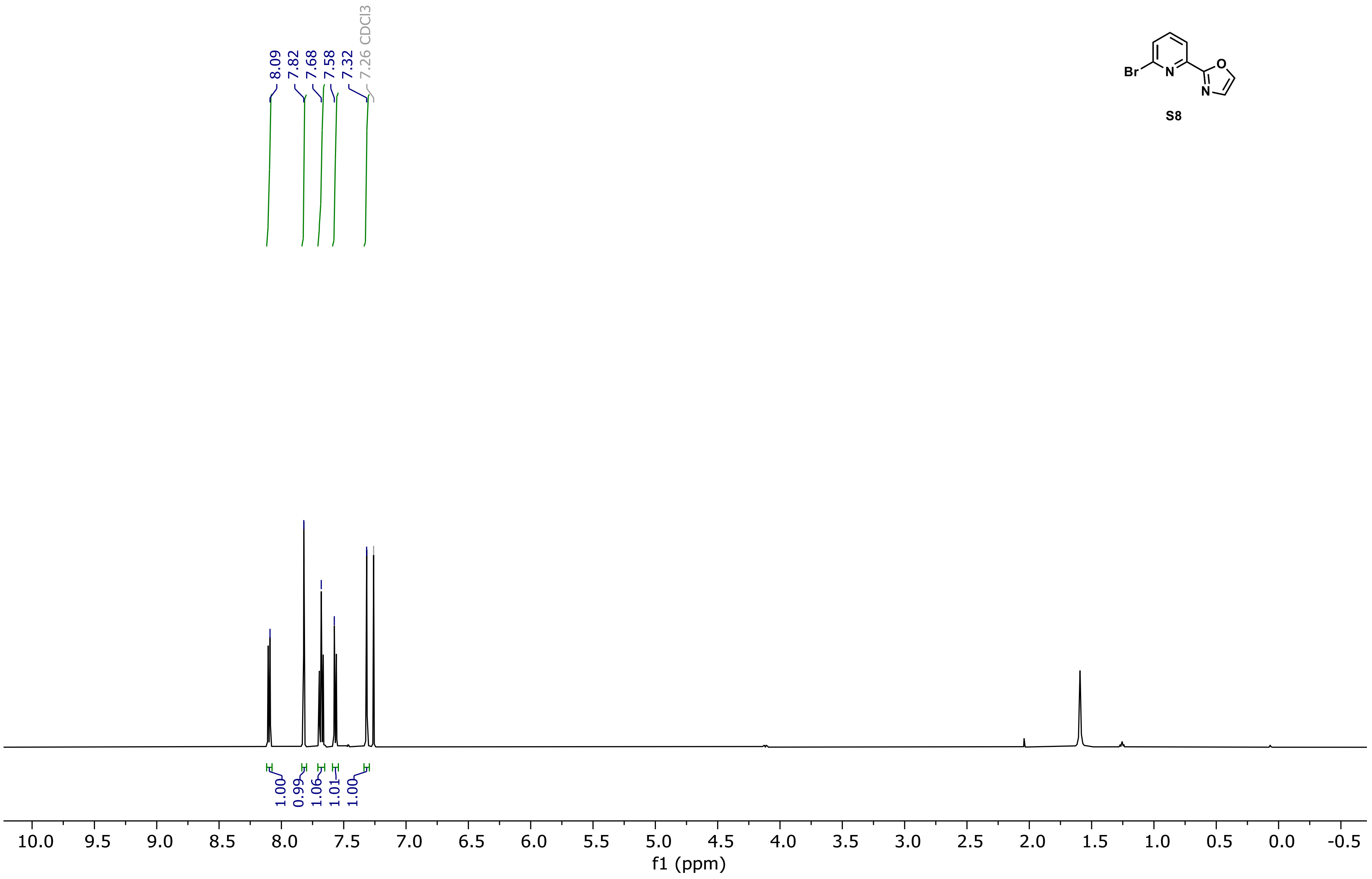

^{13}C NMR of S8

$CDCl_3$, 126 MHz, 298 K

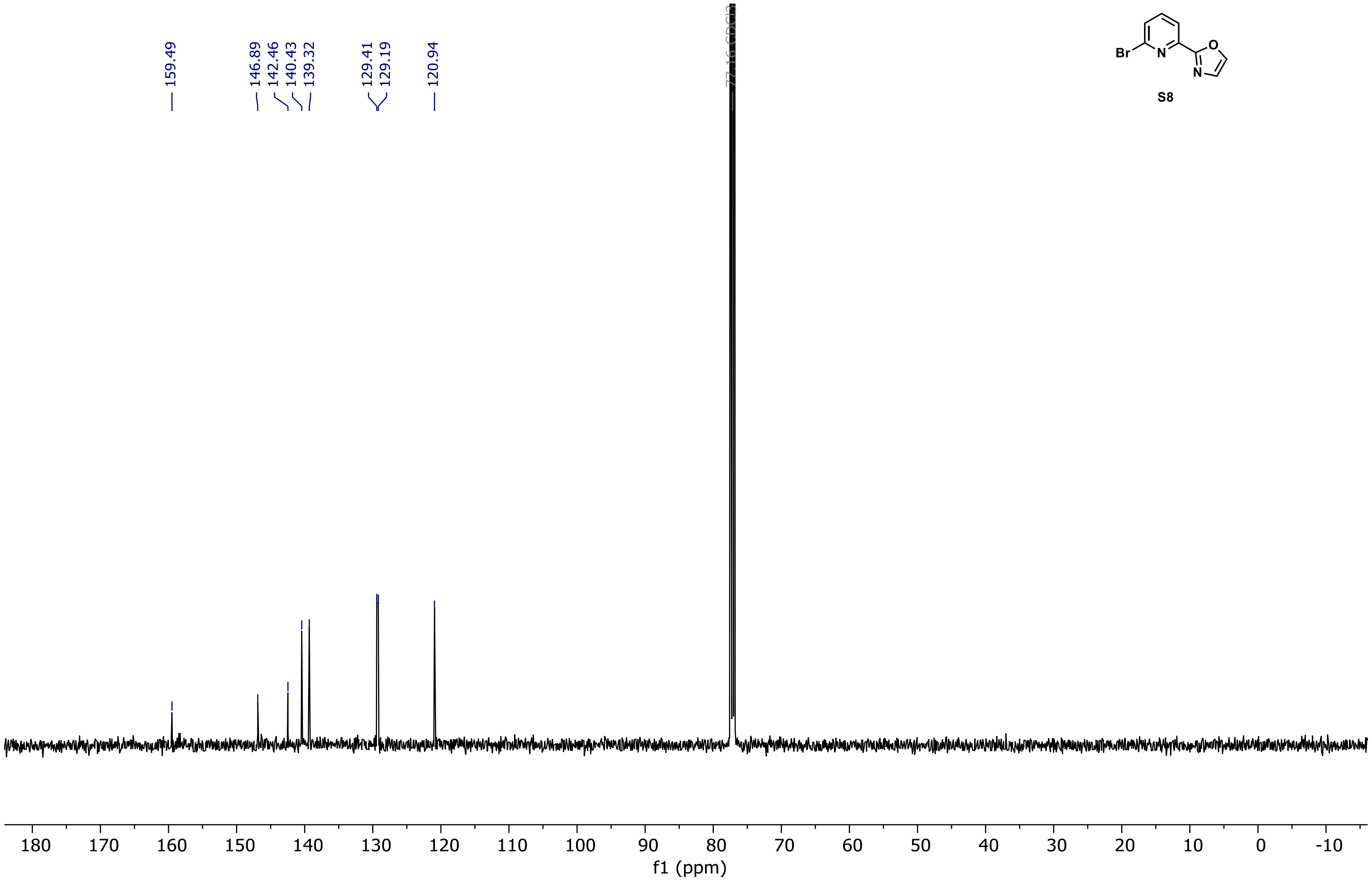

^{1}H NMR of S9

$CDCl_3$, 500 MHz, 298 K

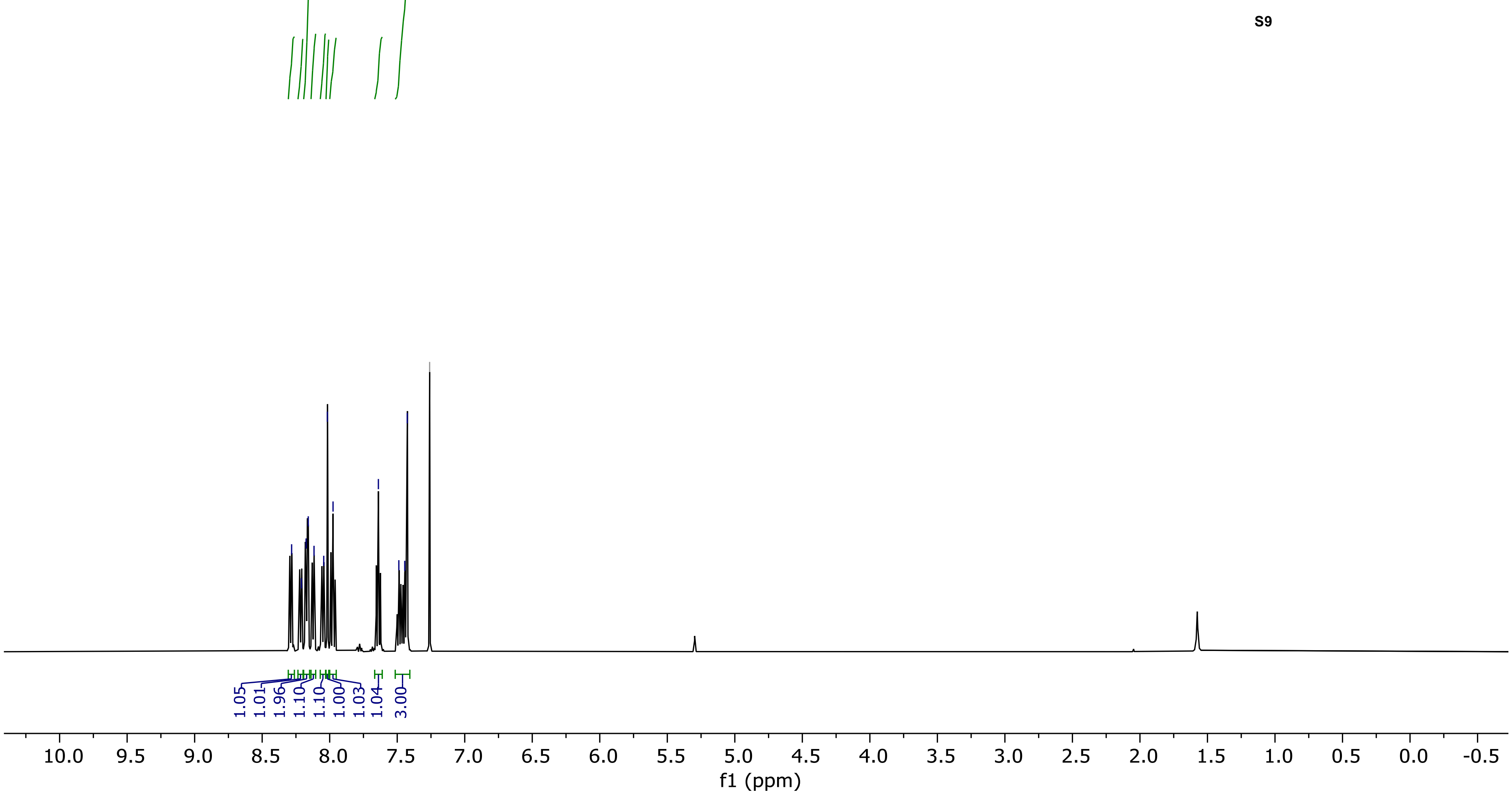

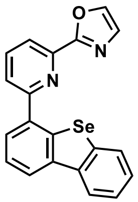

S9

^{13}C NMR of S9

$CDCl_3$, 126 MHz, 298 K

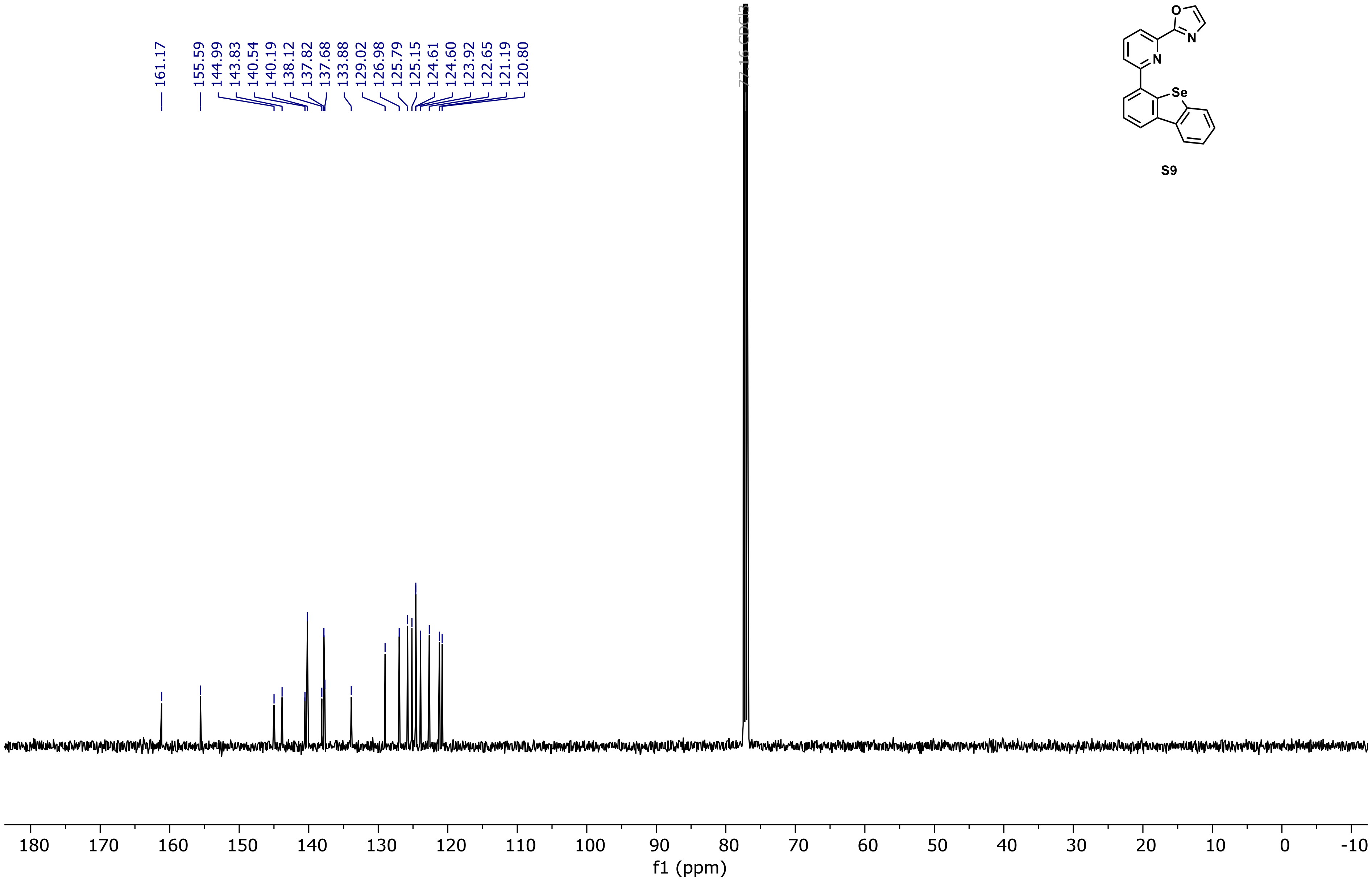

^{77}Se NMR of S9

CDCl$_3$, 115 MHz, 298 K

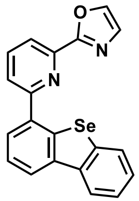

S9

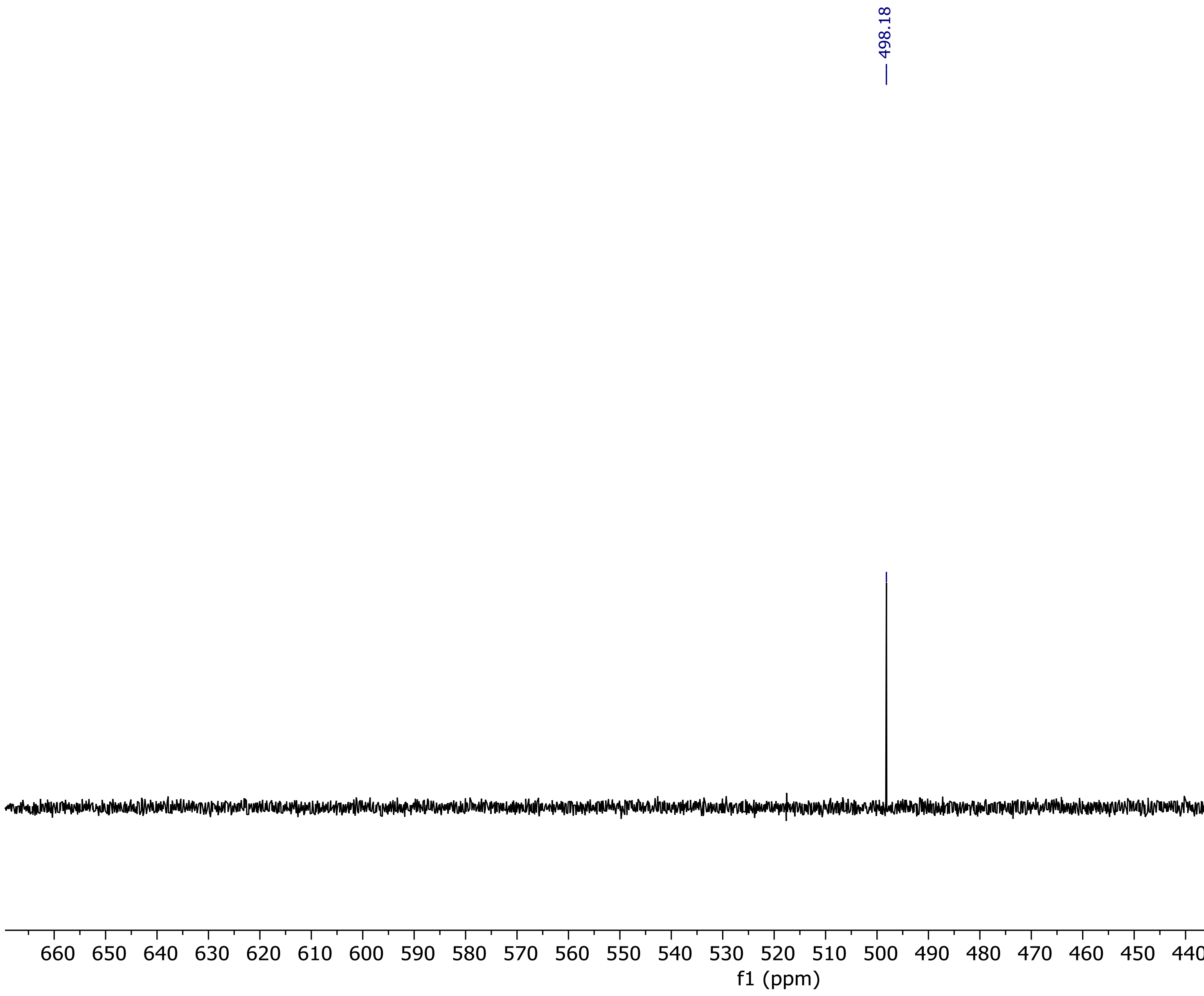

^{1}H NMR of 1

CD3OD, 500 MHz, 298 K

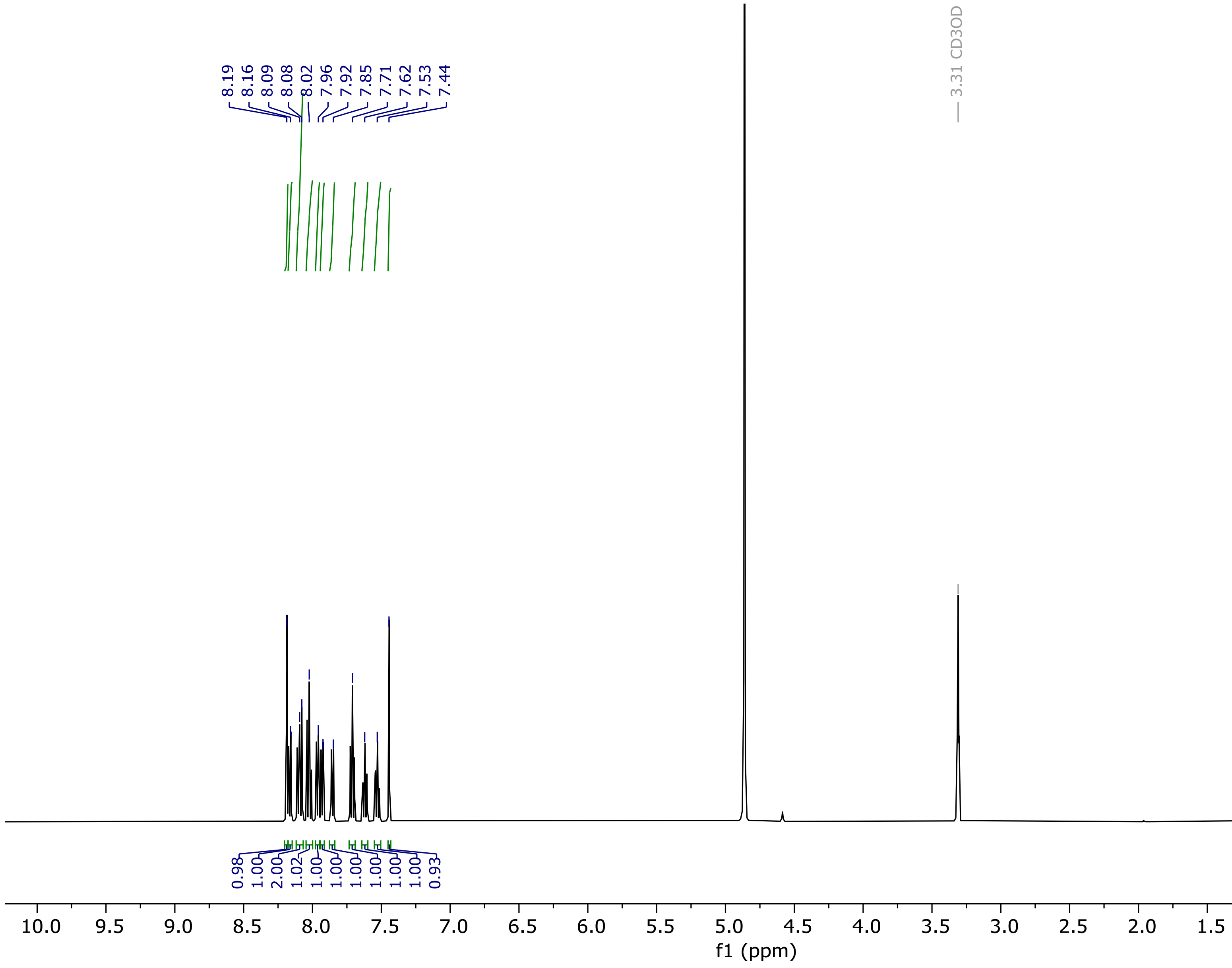

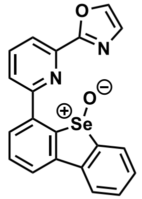

1

^{13}C NMR of 1

CD_3OD, 126 MHz, 298 K

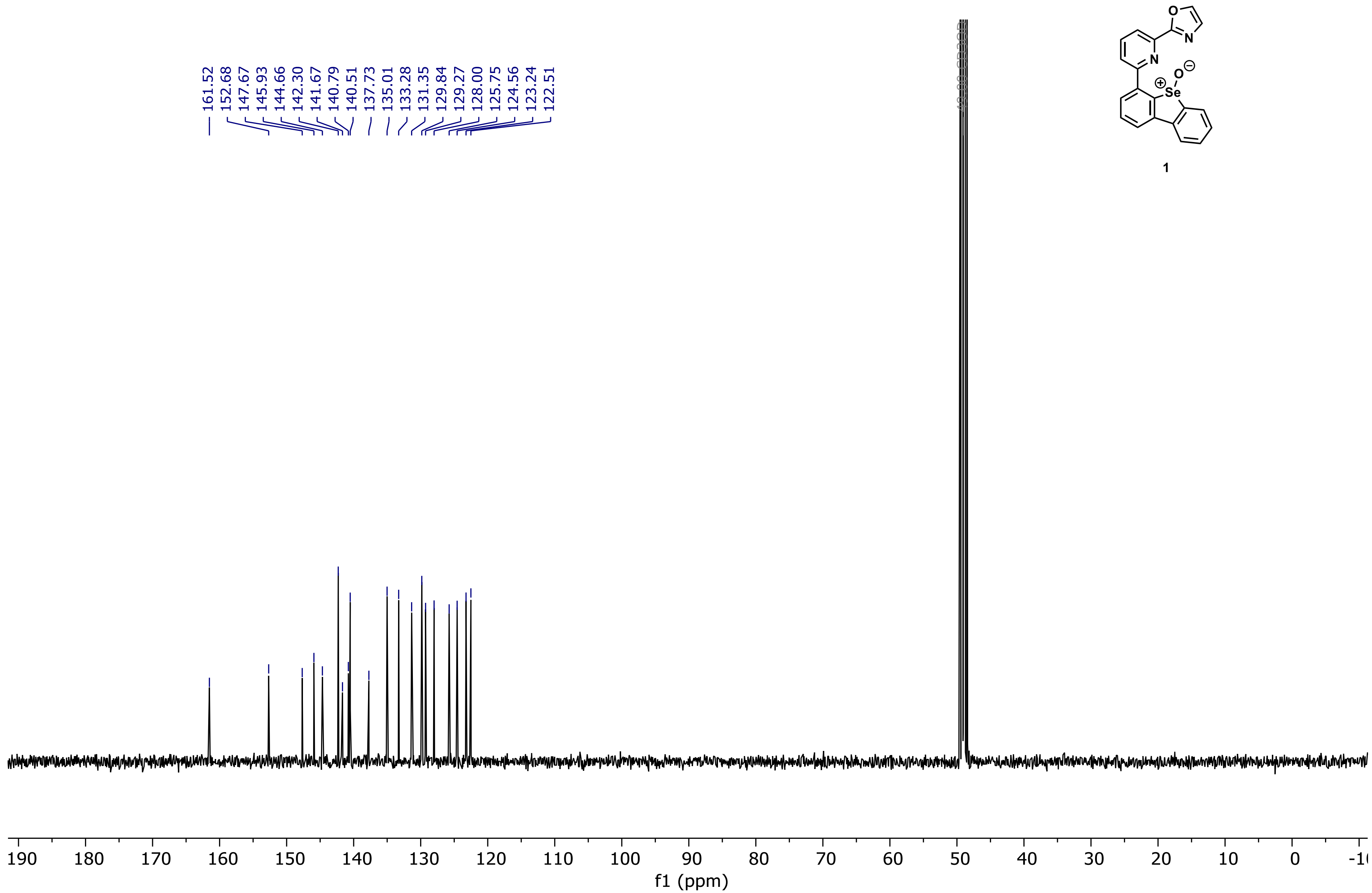

1

^{77}Se NMR of 1

CD$_3$OD, 115 MHz, 298 K

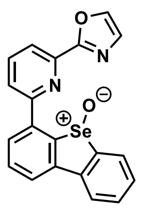

1

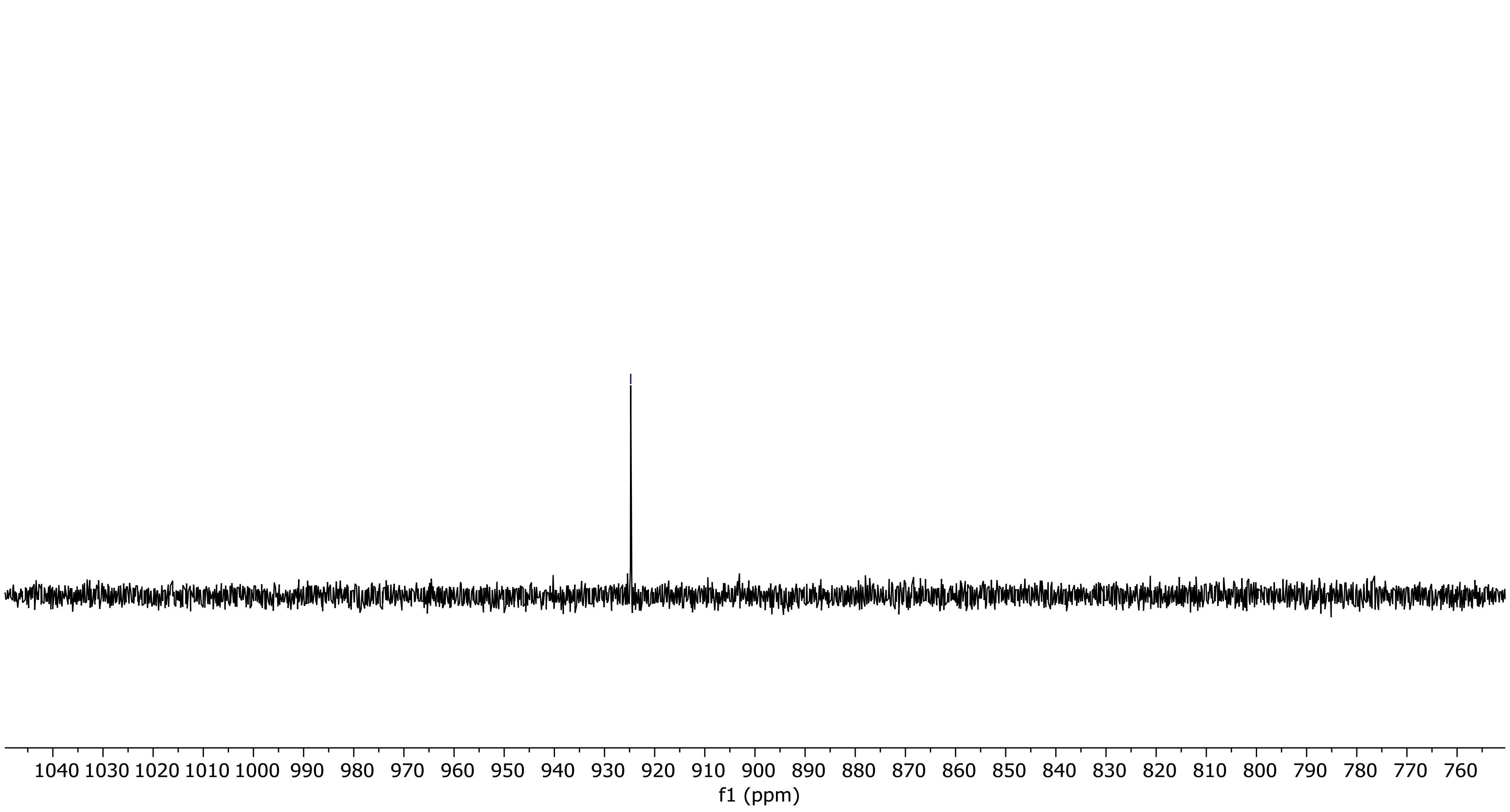

^{1}H NMR of S10

CDCl$_3$, 500 MHz, 298 K

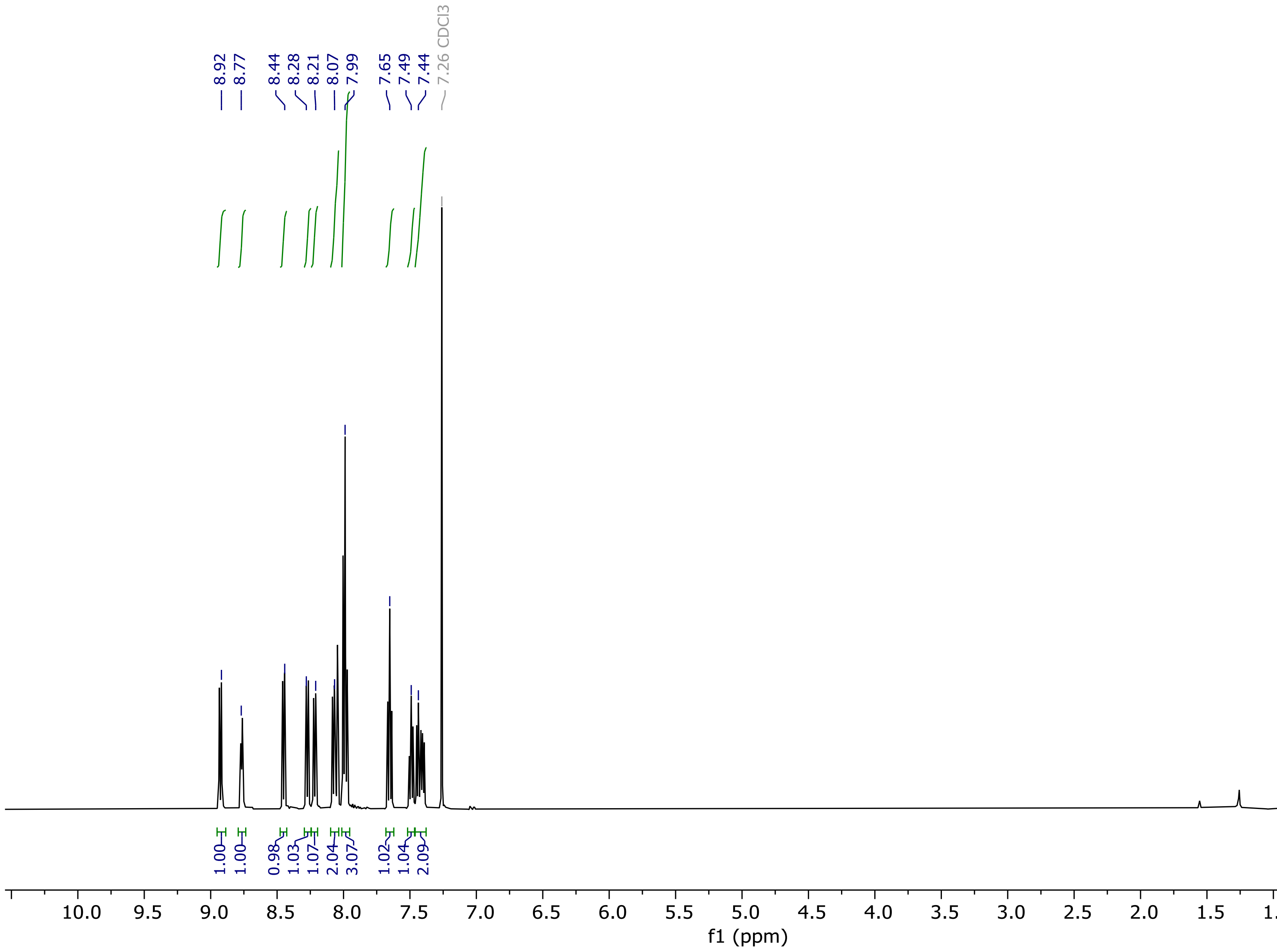

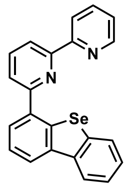

S10

^{13}C NMR of S10

CDCl$_3$, 151 MHz, 298 K

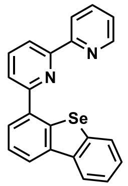

S10

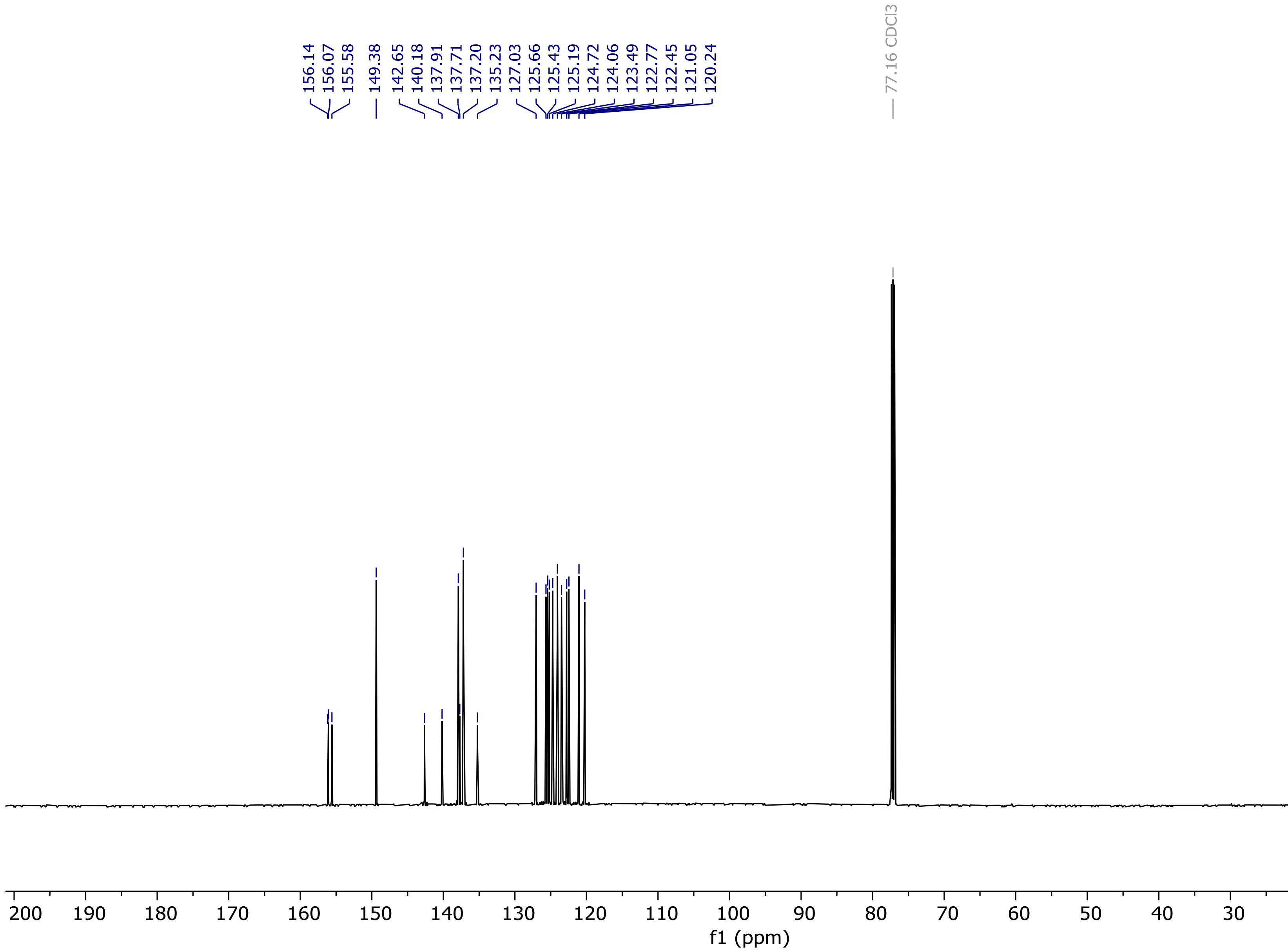

^{77}Se NMR of S10

CDCl$_3$, 115 MHz, 298 K

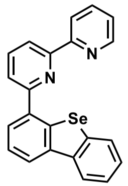

S10

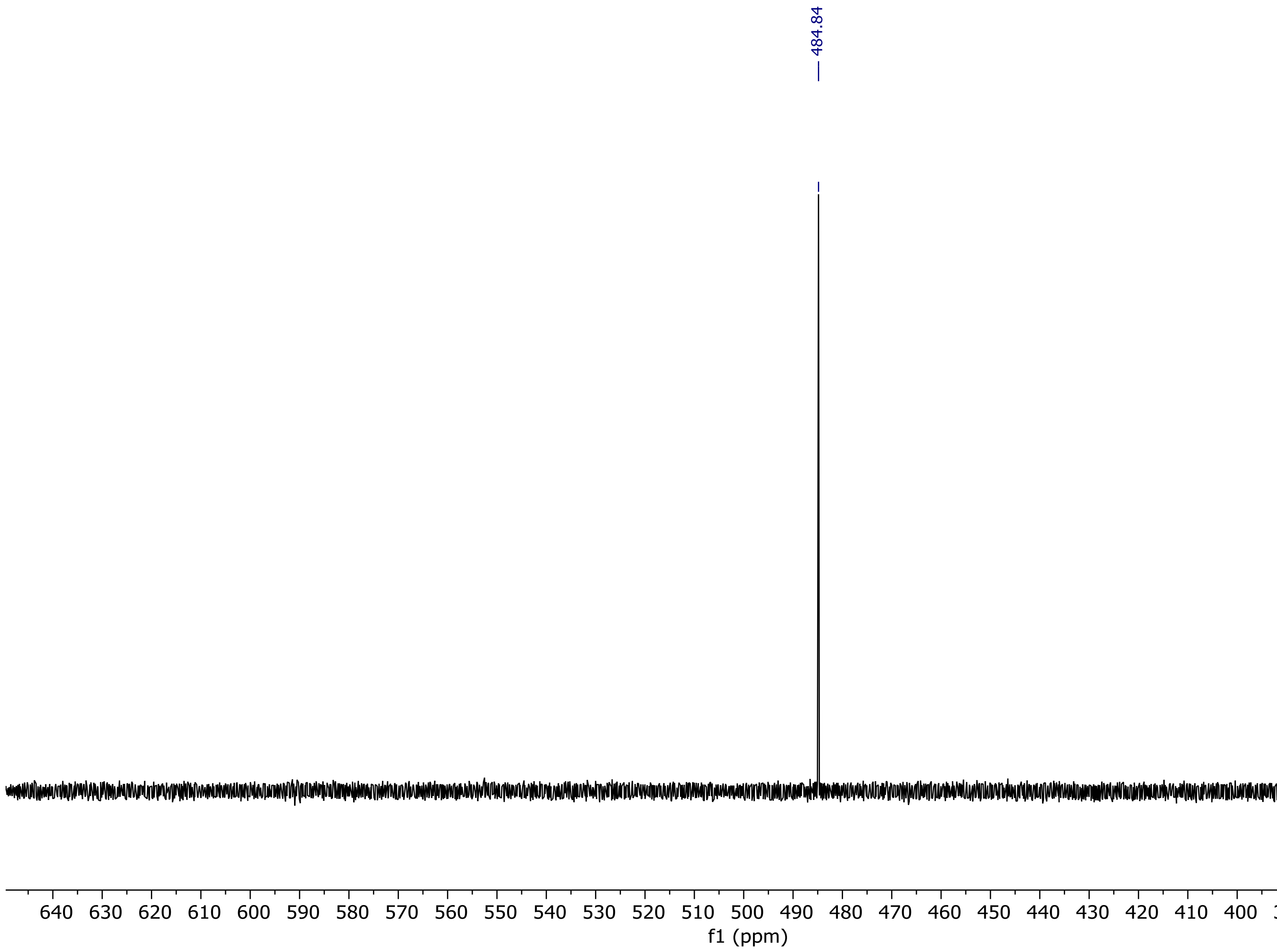

^{1}H NMR of S11

CDCl$_3$, 600 MHz, 298 K

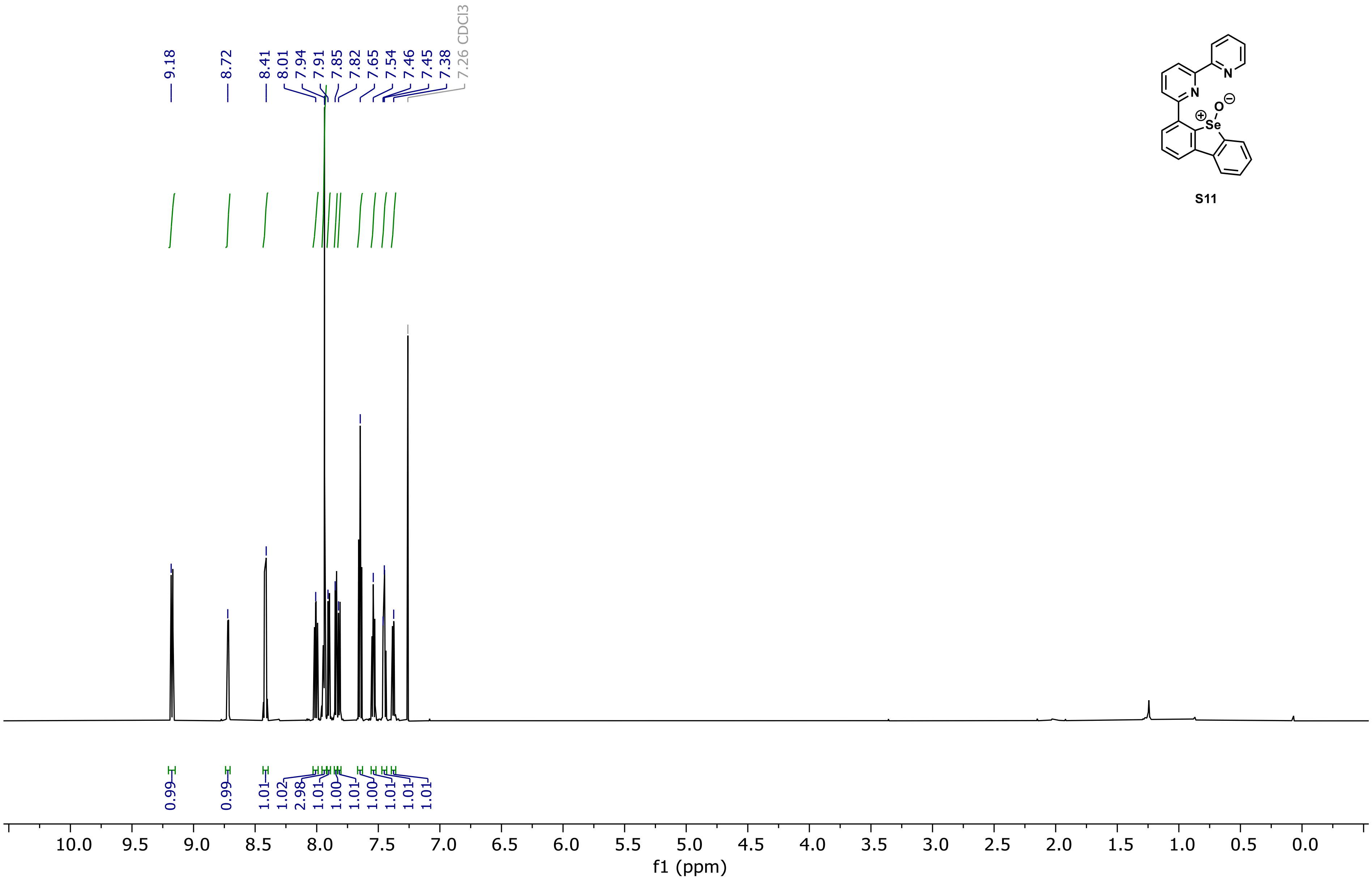

S11

^{13}C NMR of S11

$CDCl_3$, 151 MHz, 298 K

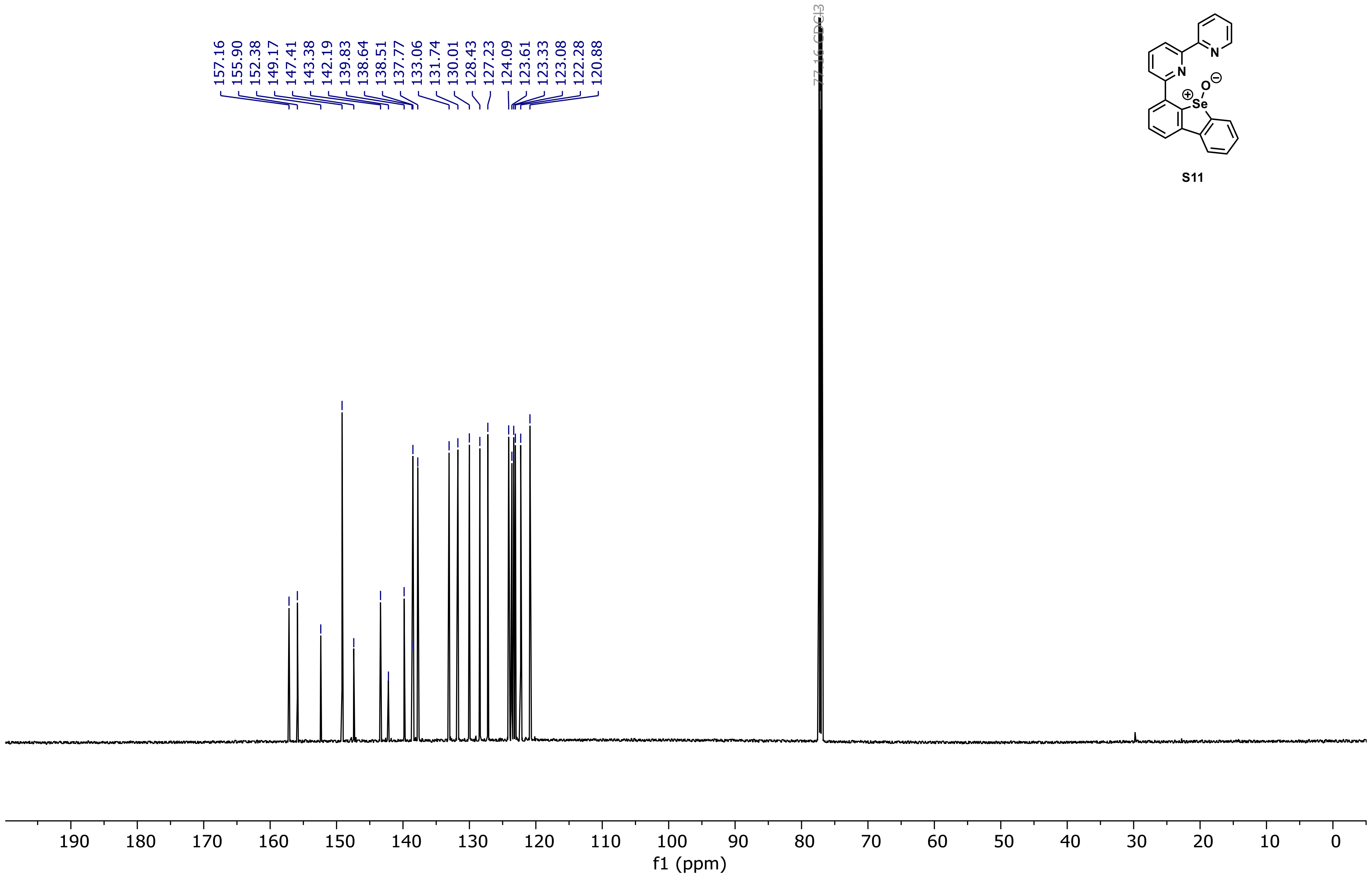

^{77}Se NMR of S11

$CDCl_3$, 115 MHz, 298 K

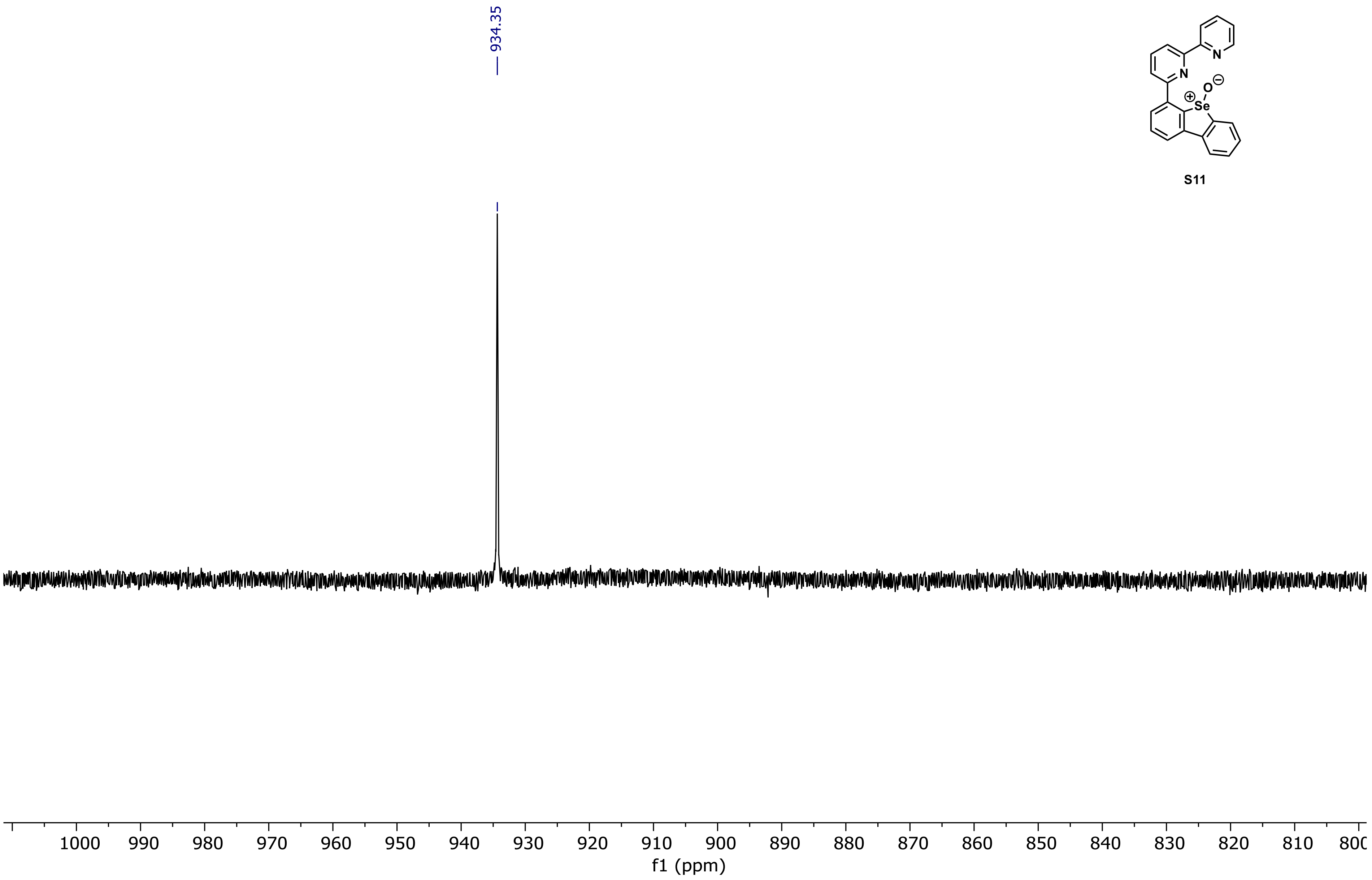

^{1}H NMR of S12

CD3OD, 600 MHz, 298 K

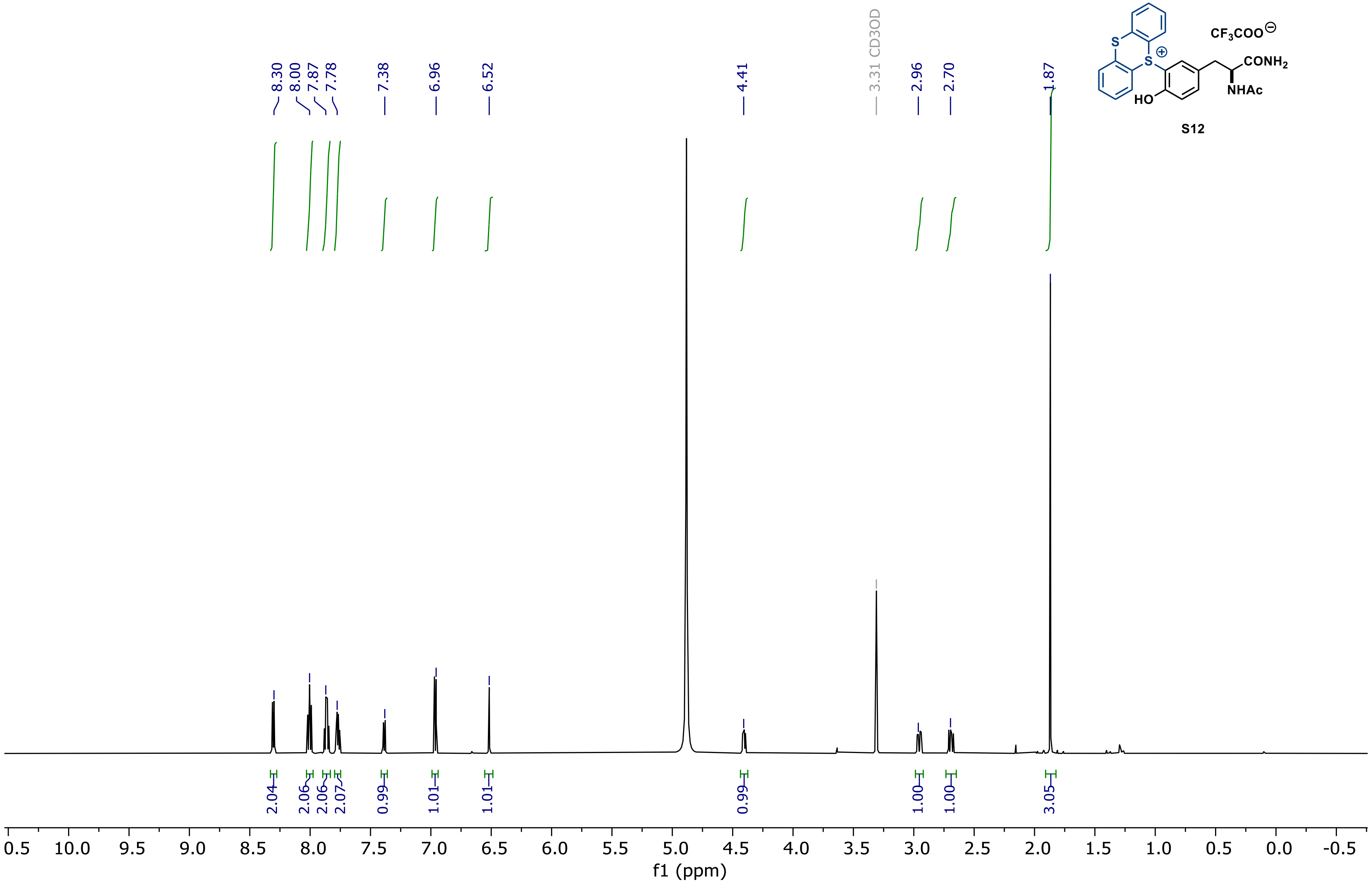

^{13}C NMR of S12

CD3OD, 151 MHz, 298 K

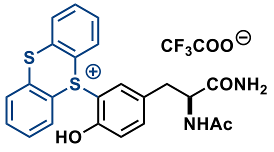

S12

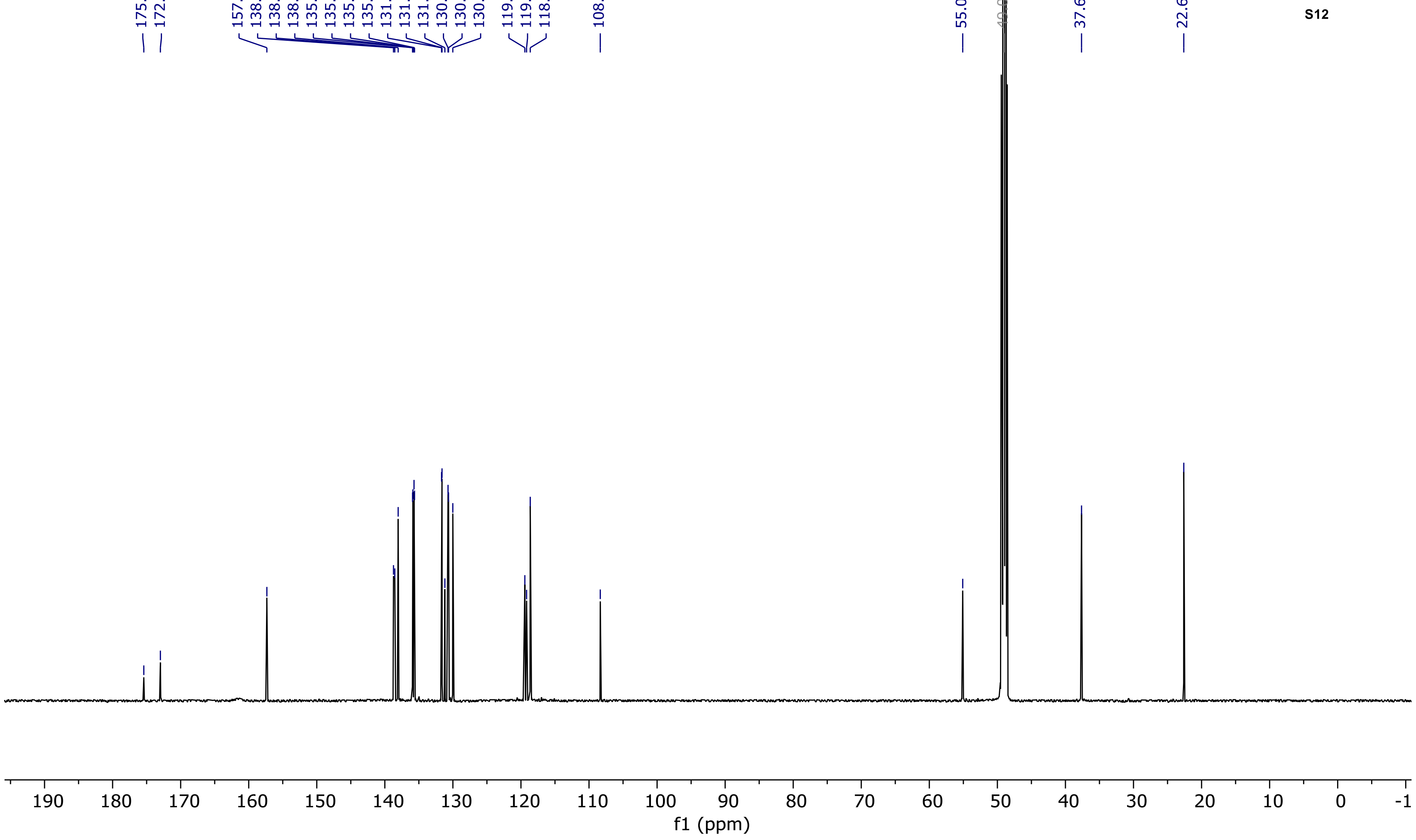

^{19}F NMR of S12

CD_3OD, 565 MHz, 298 K

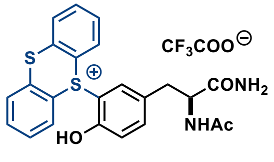

S12

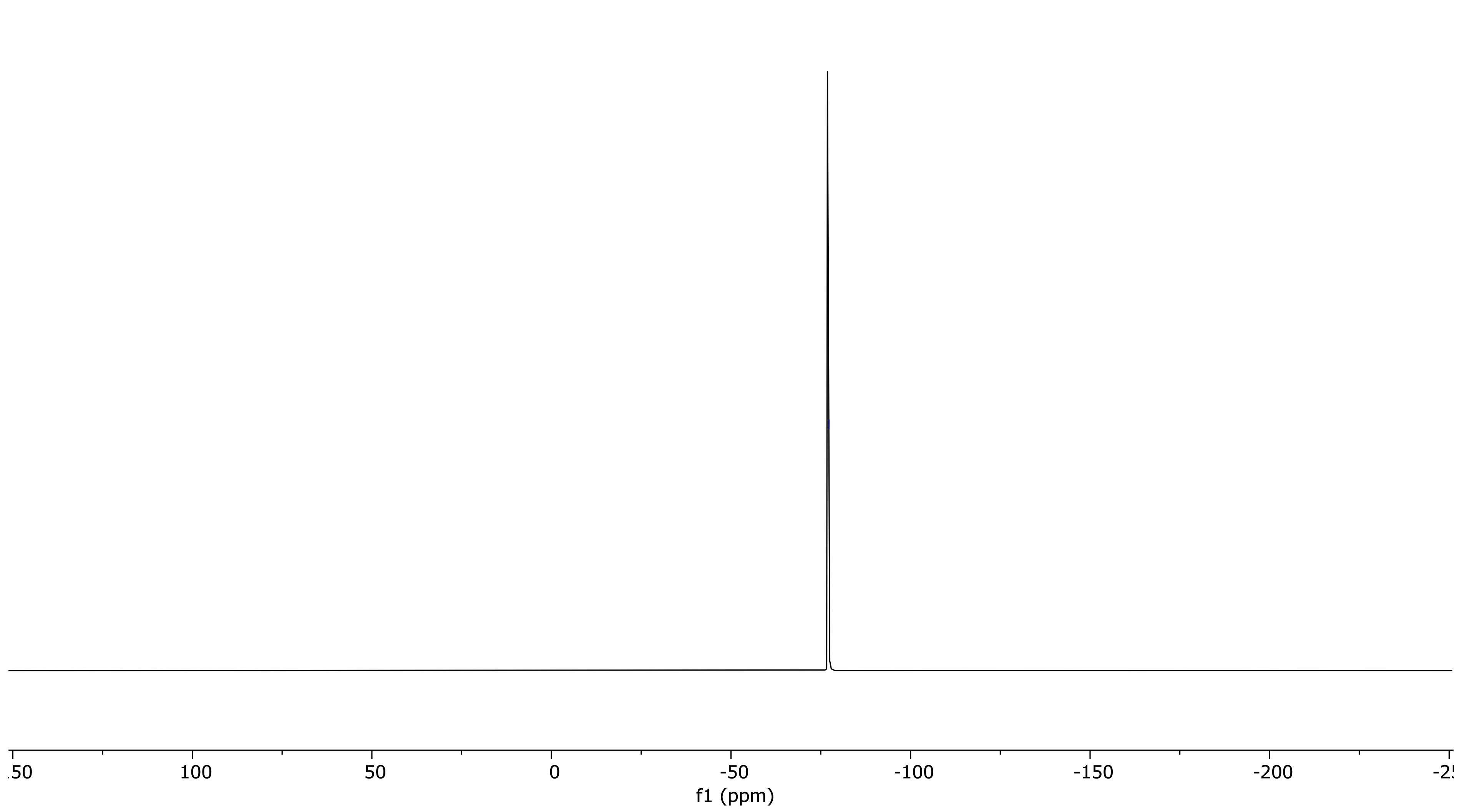

^{1}H NMR of S13

CD3OD, 600 MHz, 298 K

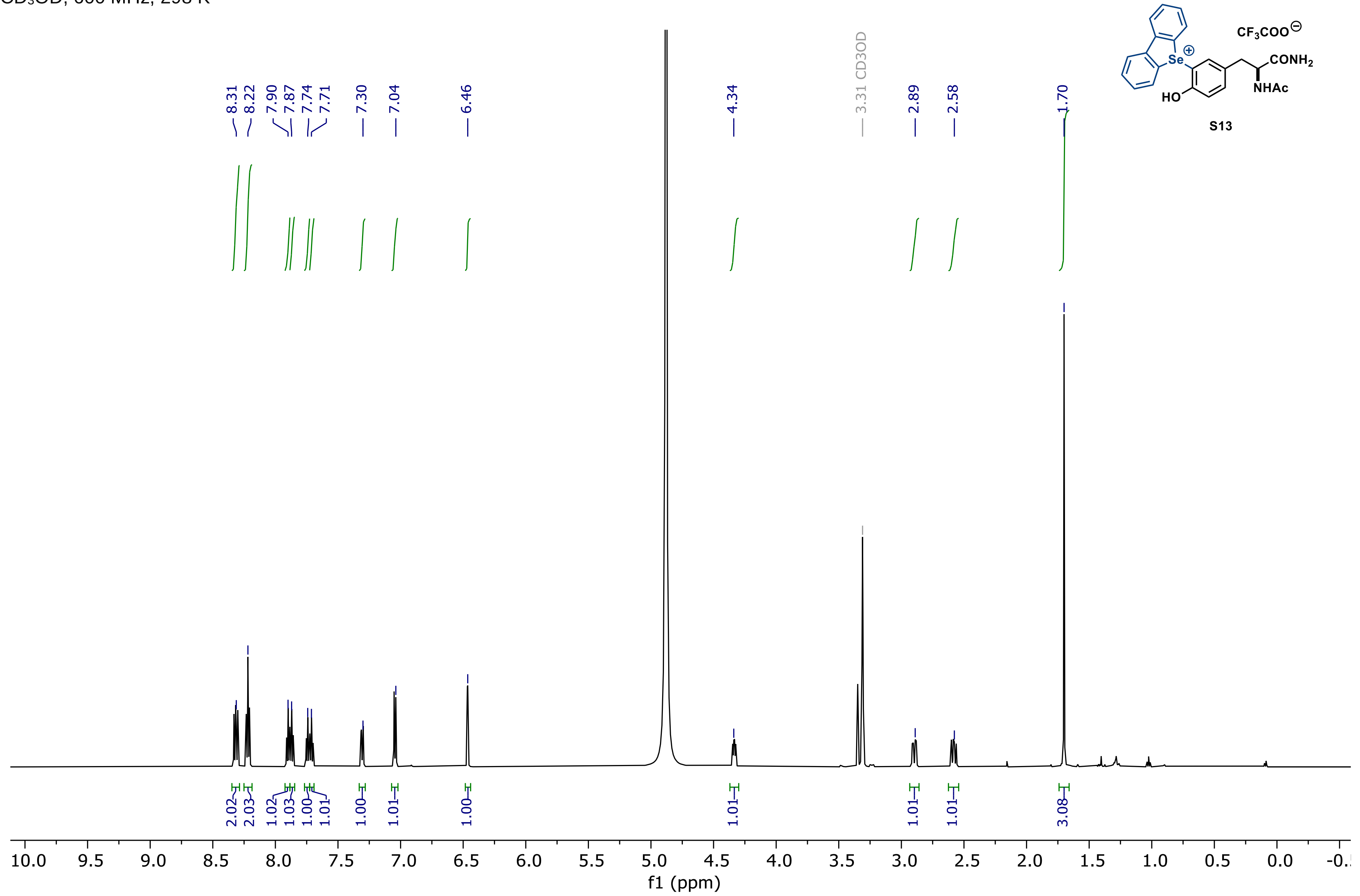

^{13}C NMR of S13

CD3OD, 151 MHz, 298 K

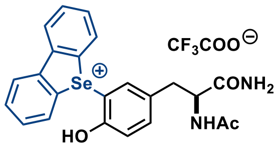

S13

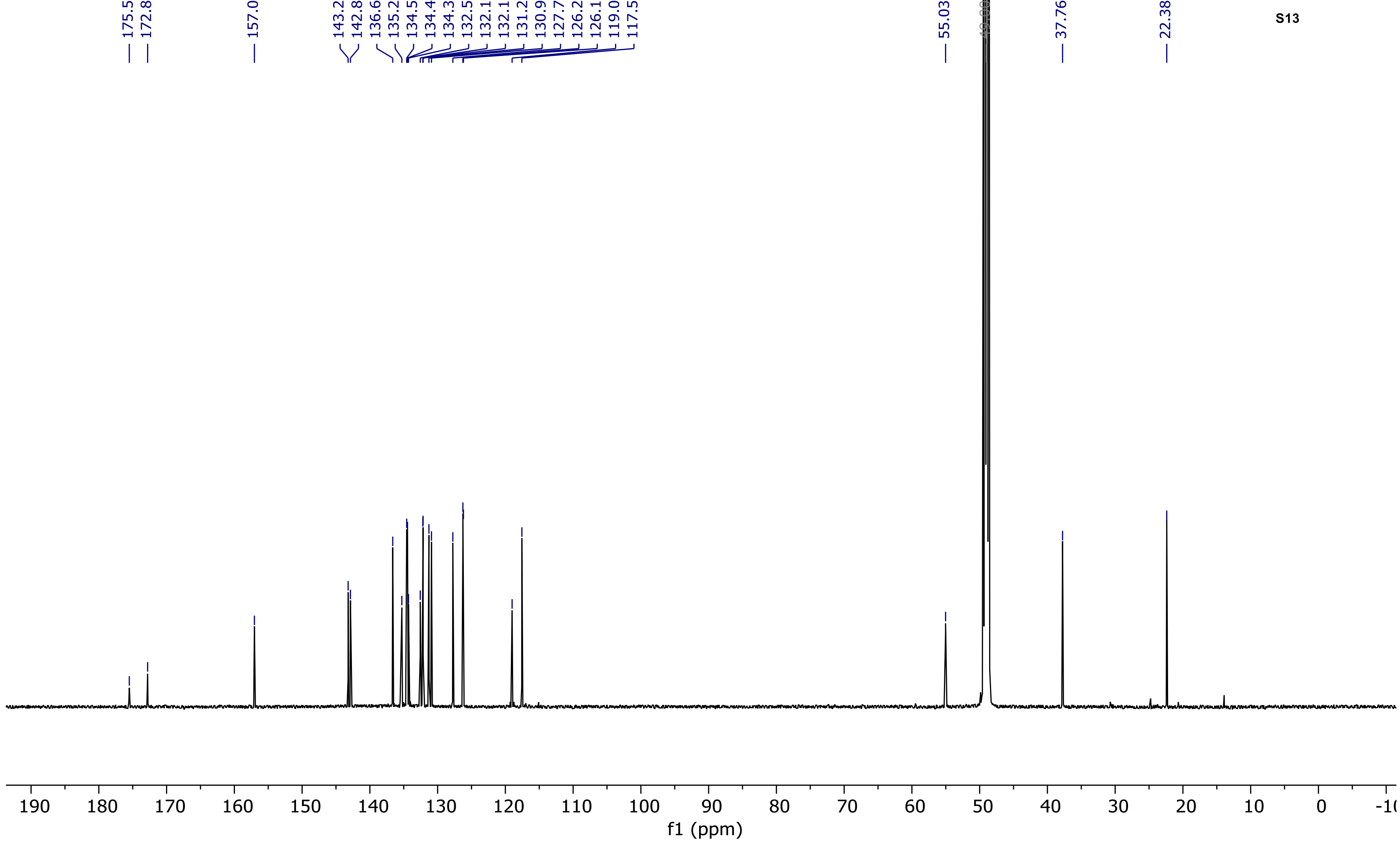

^{19}F NMR of S13

CD_3OD, 565 MHz, 298 K

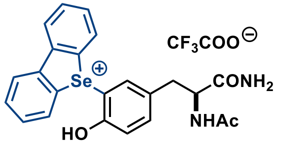

S13

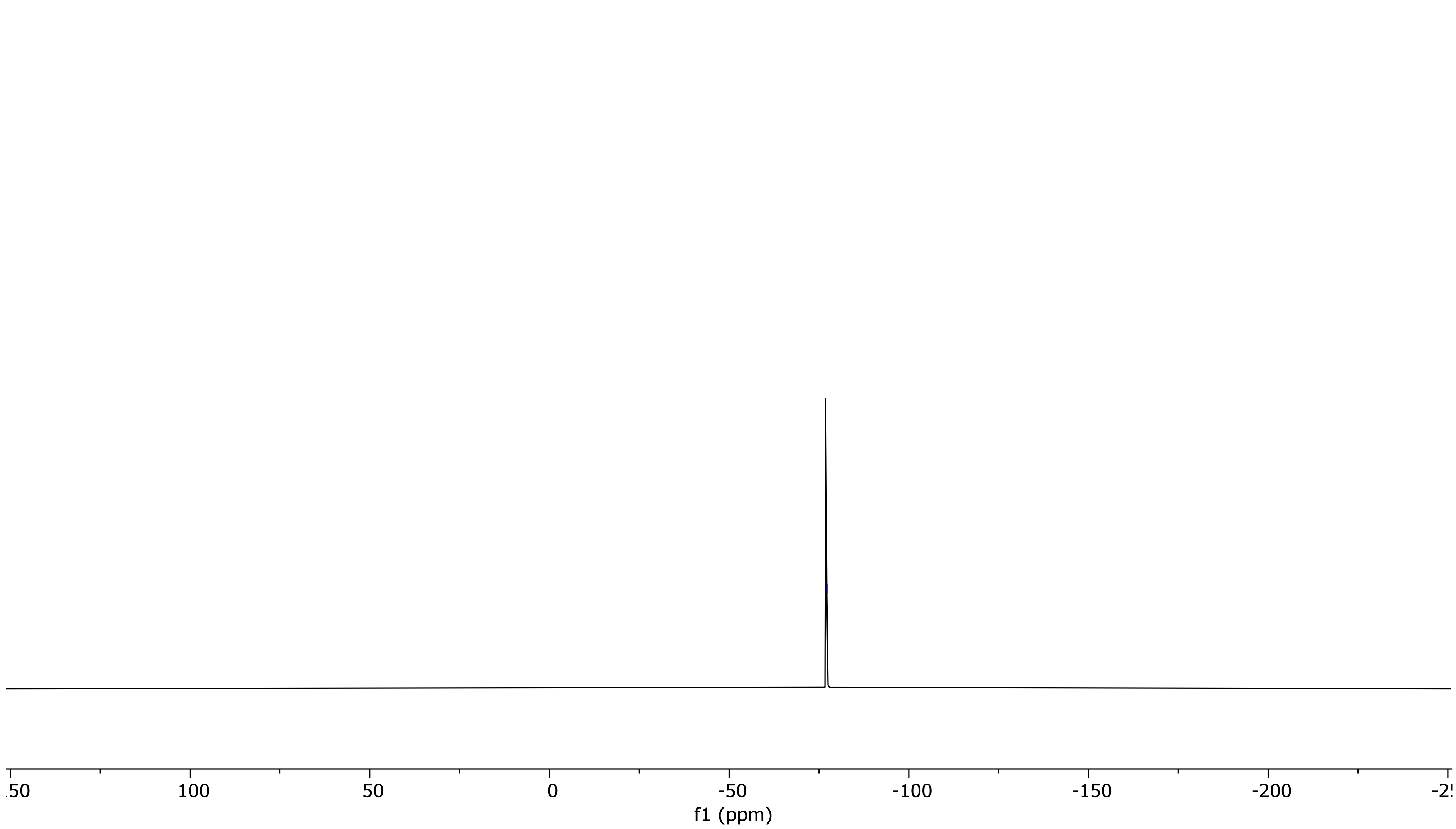

^{77}Se NMR of S13

CD_3OD, 115 MHz, 298 K

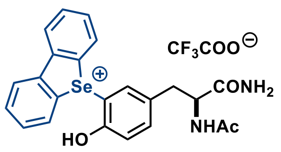

S13

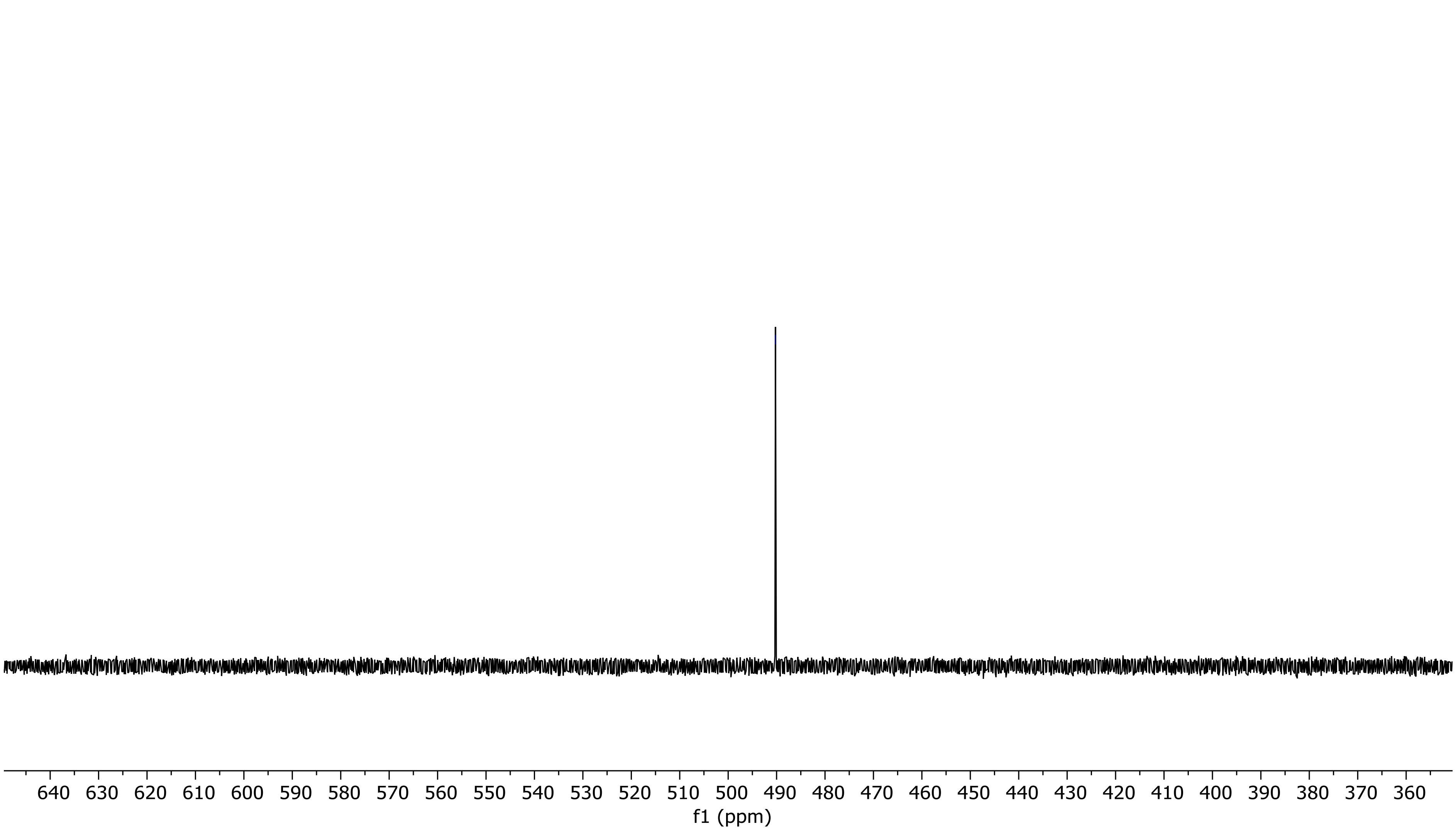

^{1}H NMR of S14

CD3OD, 600 MHz, 298 K

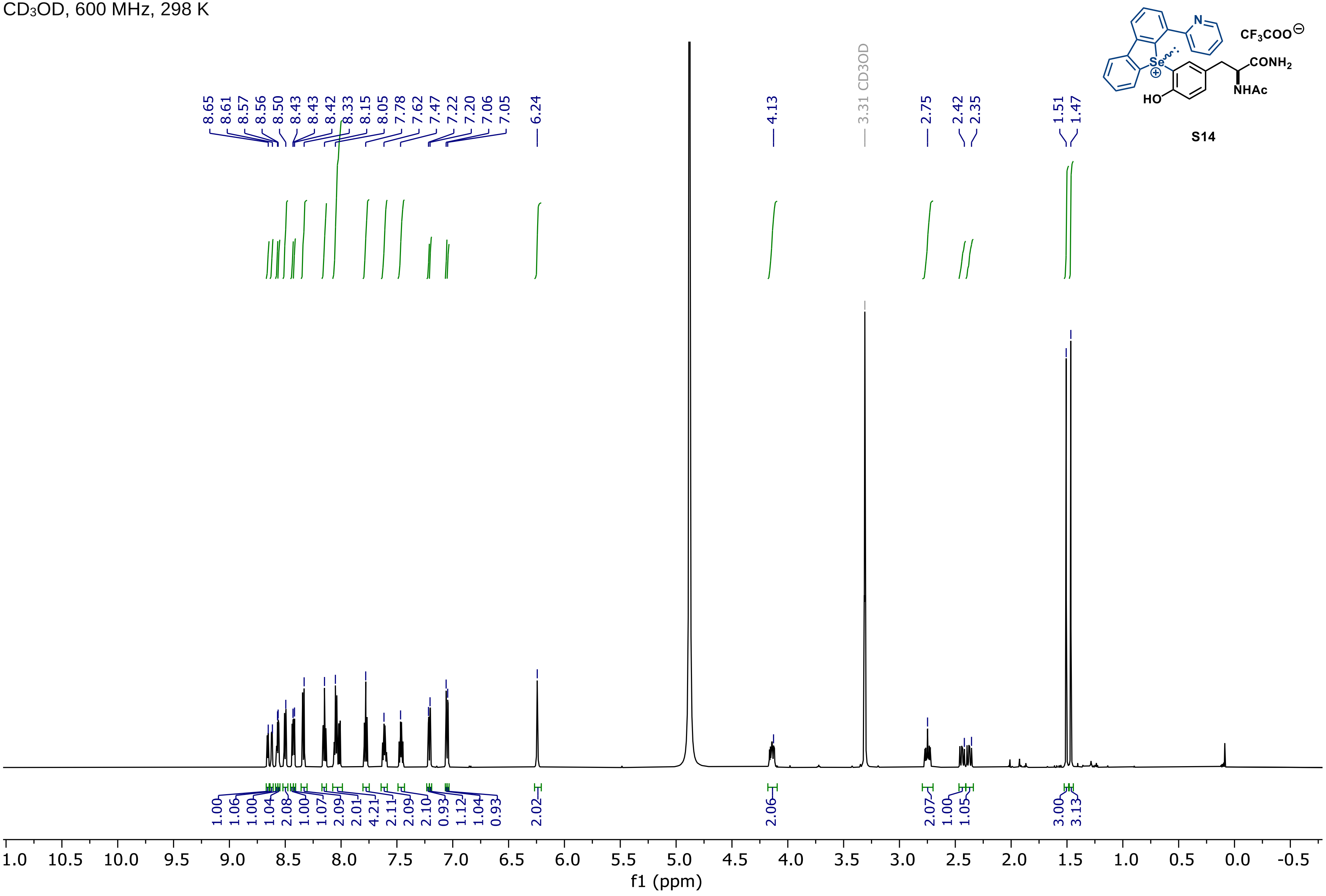

^{13}C NMR of S14

CD3OD, 151 MHz, 298 K

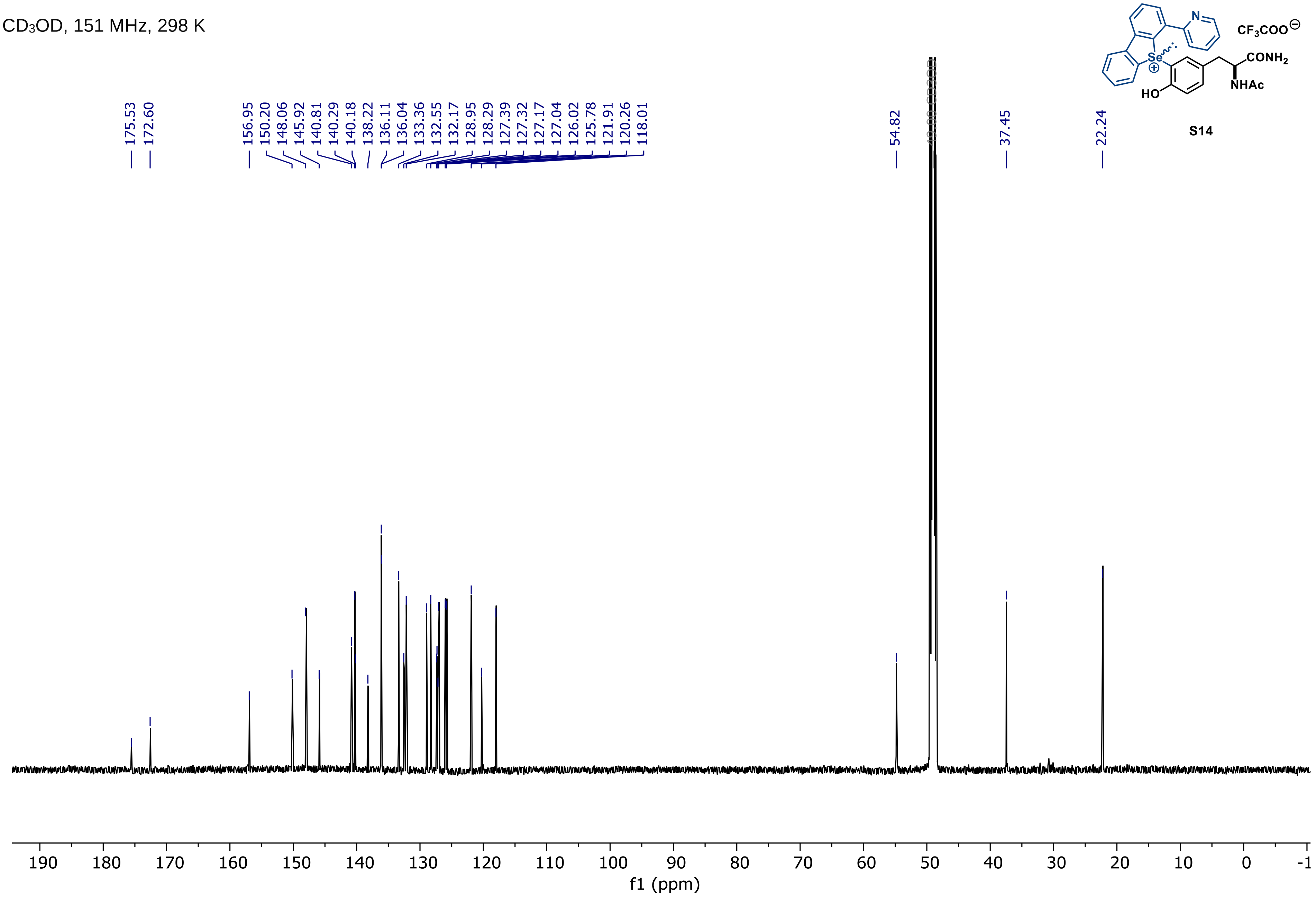

^{19}F NMR of S14

CD_3OD, 565 MHz, 298 K

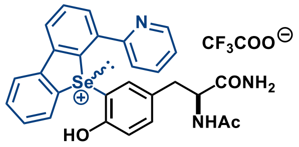

S14

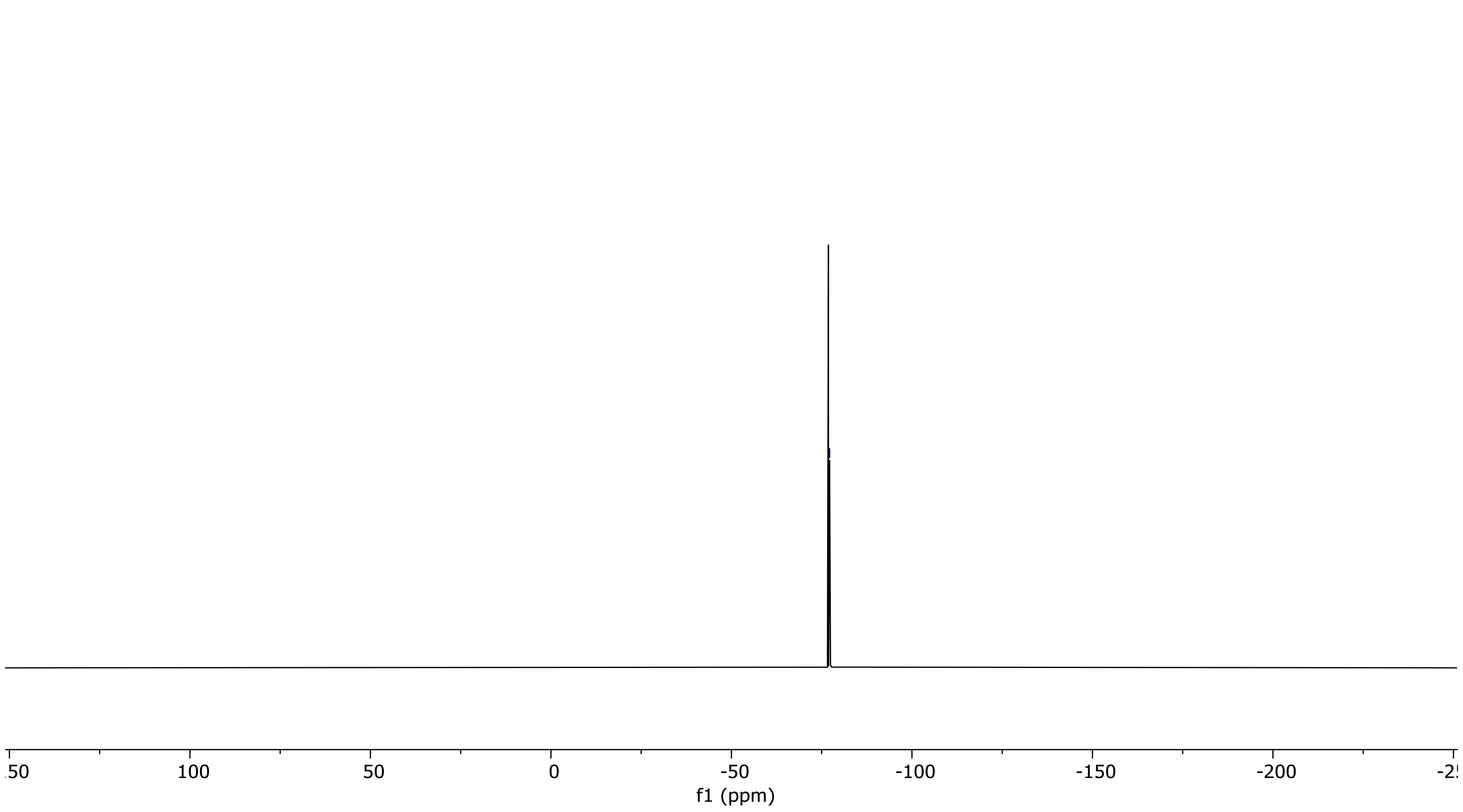

^{77}Se NMR of S14

CD_3OD, 115 MHz, 298 K

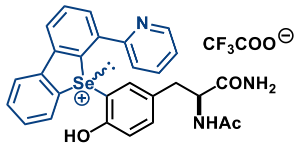

S14

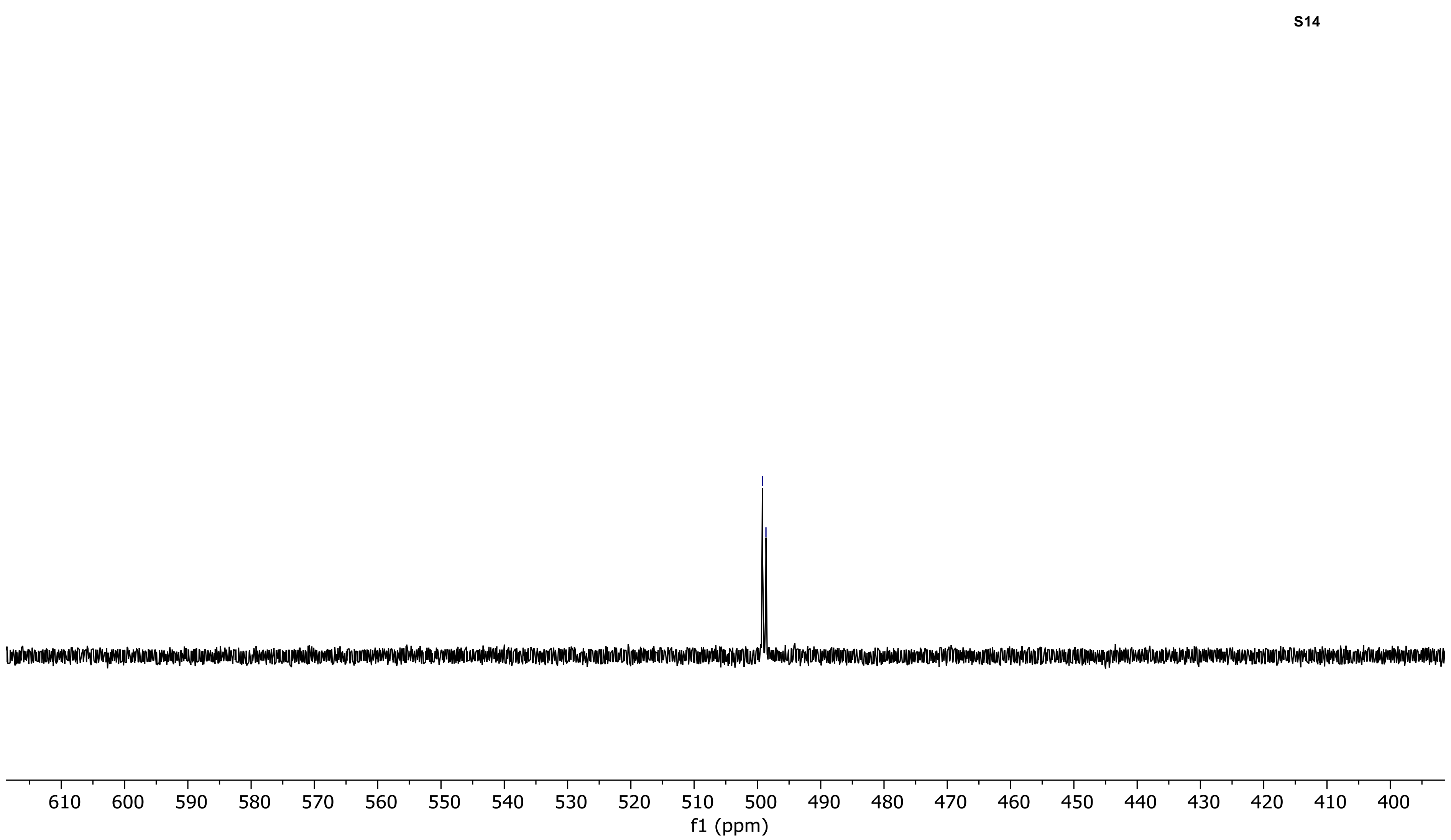

^{1}H NMR of S15

CD_3OD, 600 MHz, 298 K

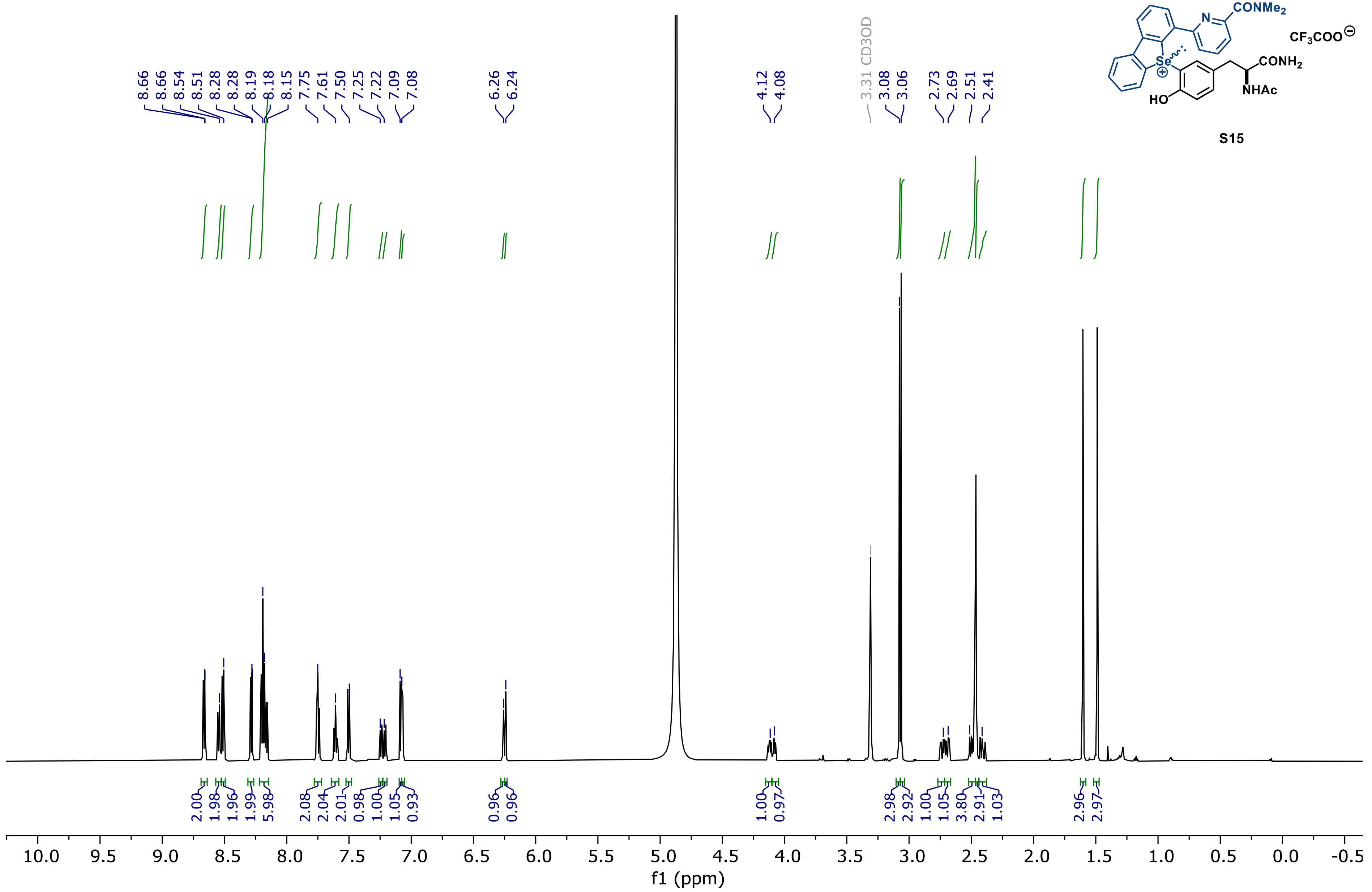

^{13}C NMR of S15

CD₃OD, 151 MHz, 298 K

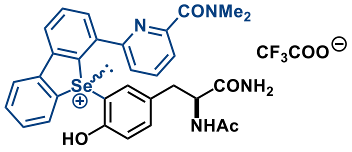

S15

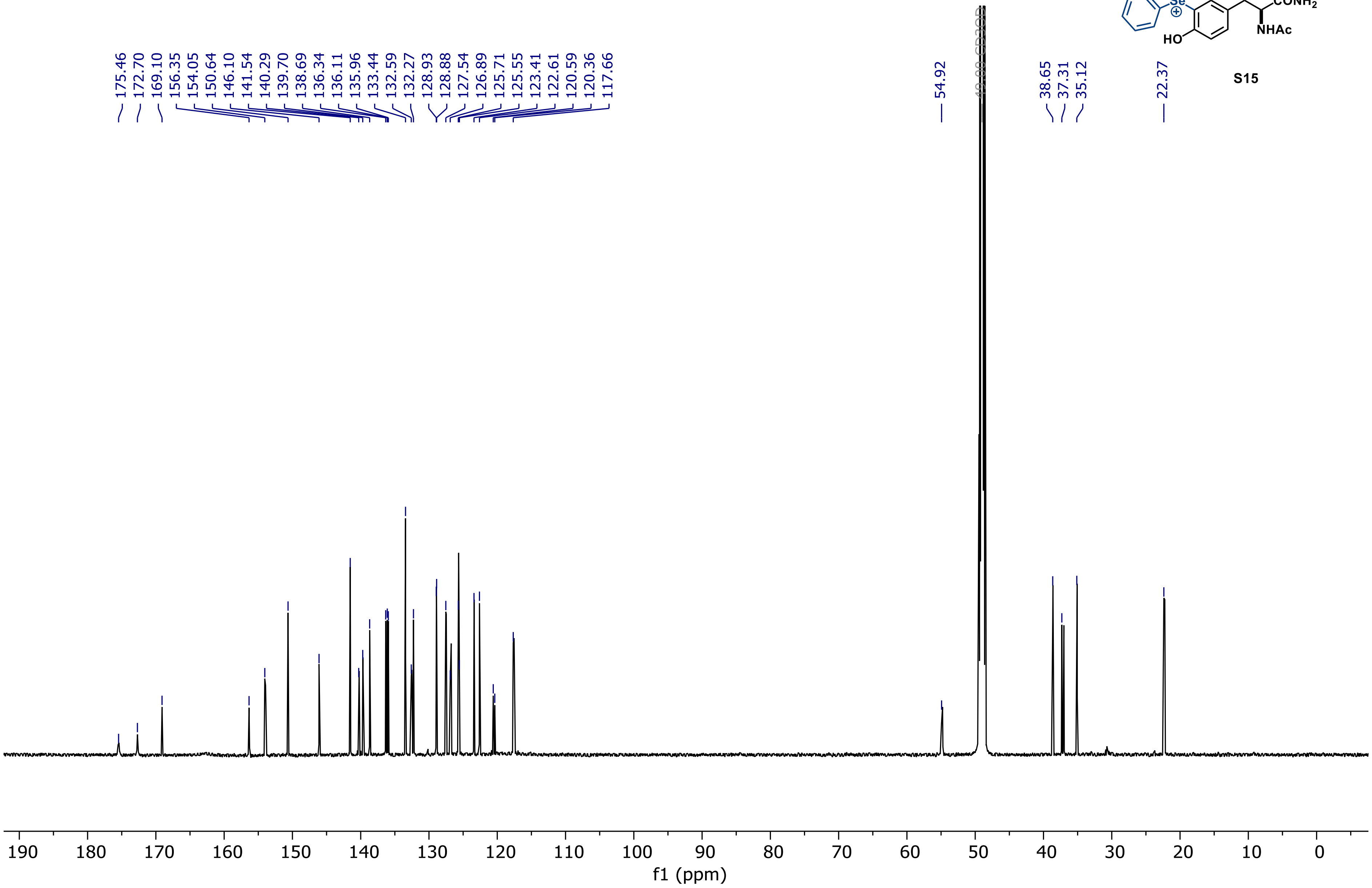

^{19}F NMR of S15

CD3OD, 565 MHz, 298 K

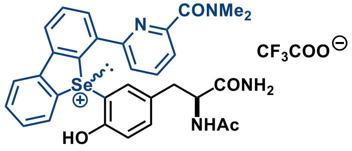

S15

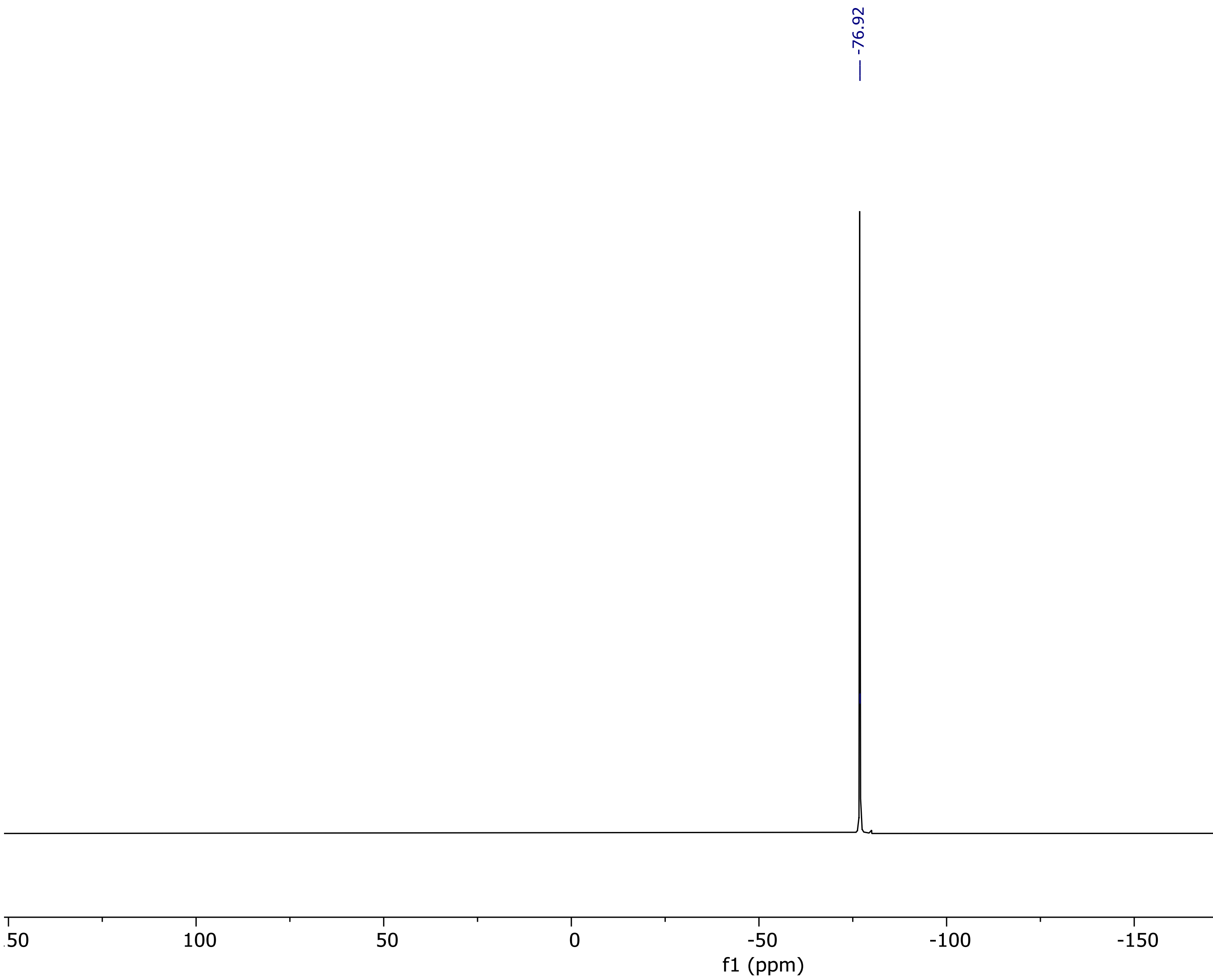

^{77}Se NMR of S15

CD_3OD, 115 MHz, 298 K

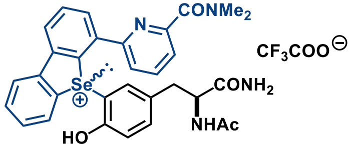

S15

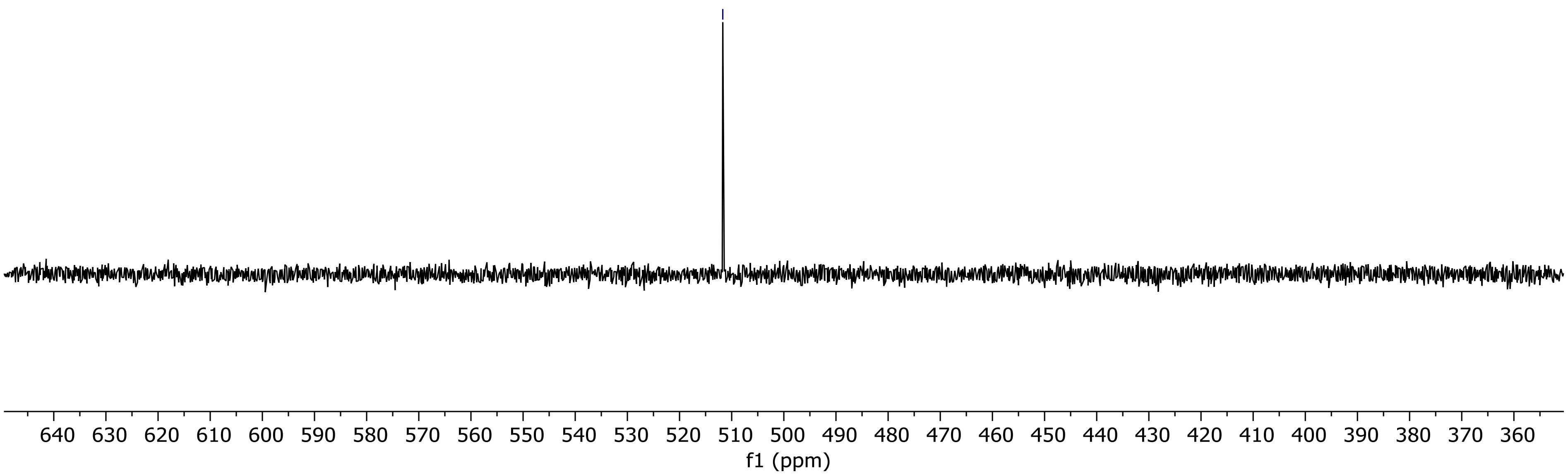

^{1}H NMR of 15

CD3OD, 600 MHz, 298 K

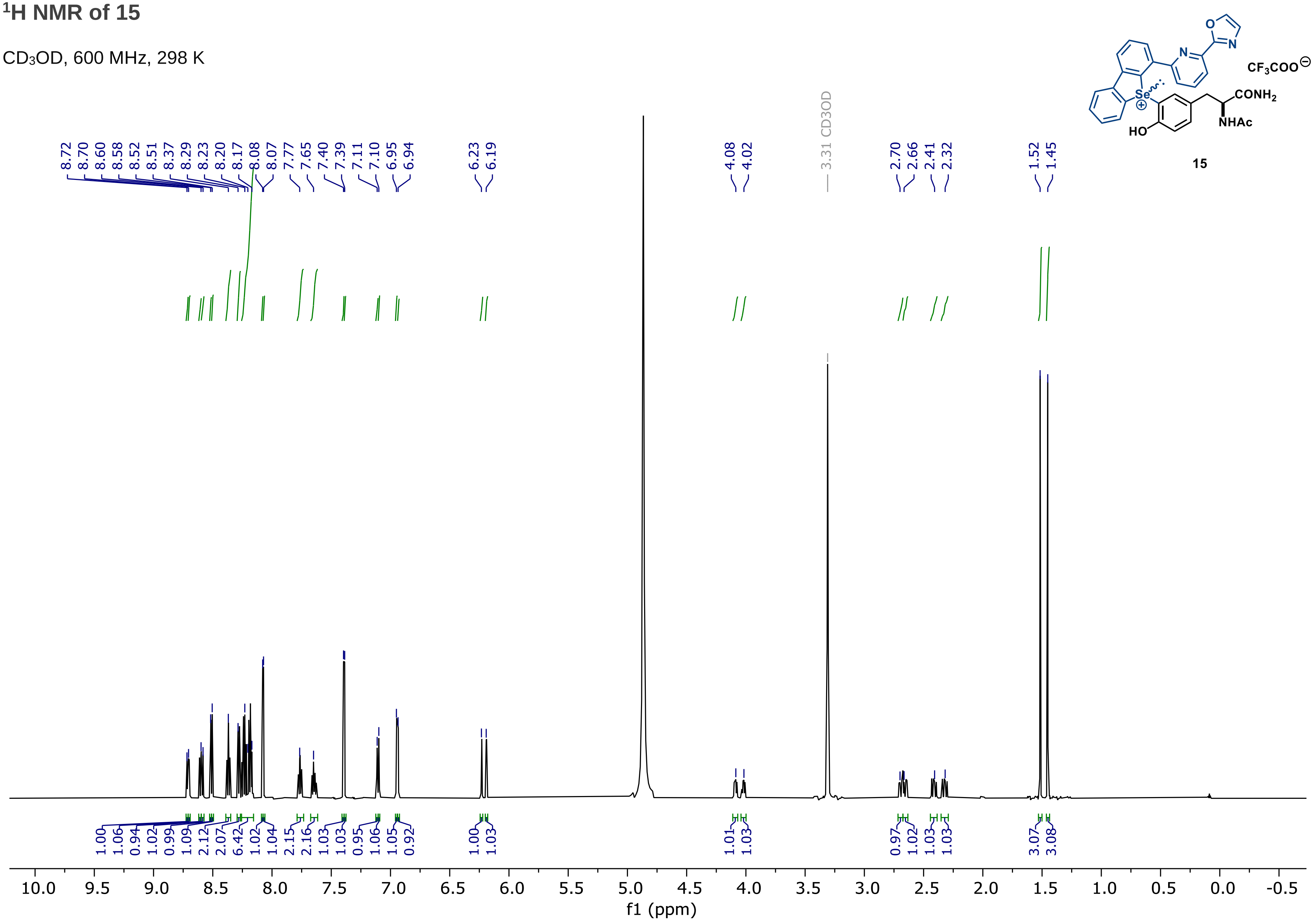

^{13}C NMR of 15

CD_3OD, 151 MHz, 298 K

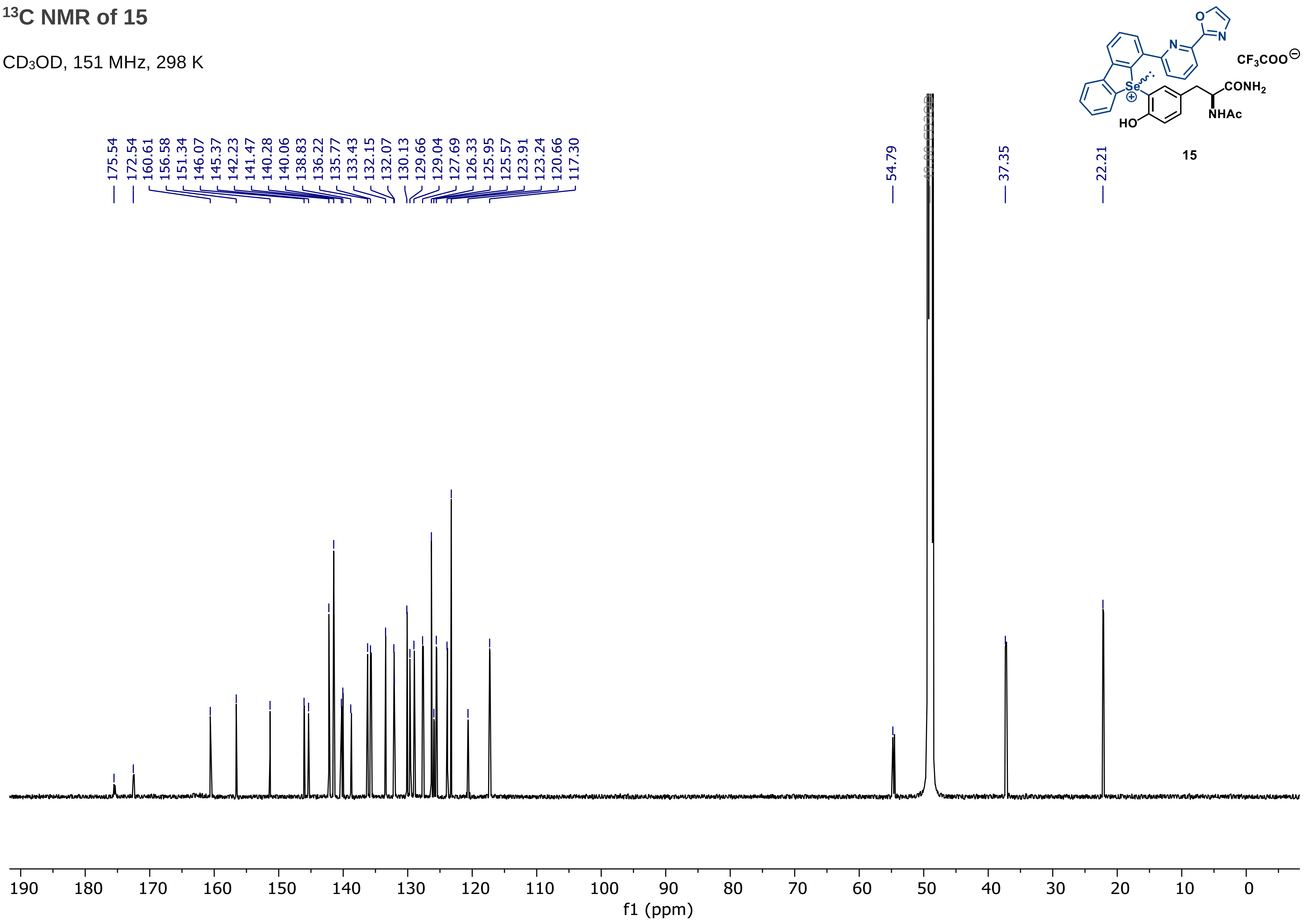

^{19}F NMR of 15

CD$_3$OD, 565 MHz, 298 K

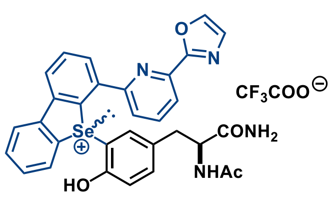

15

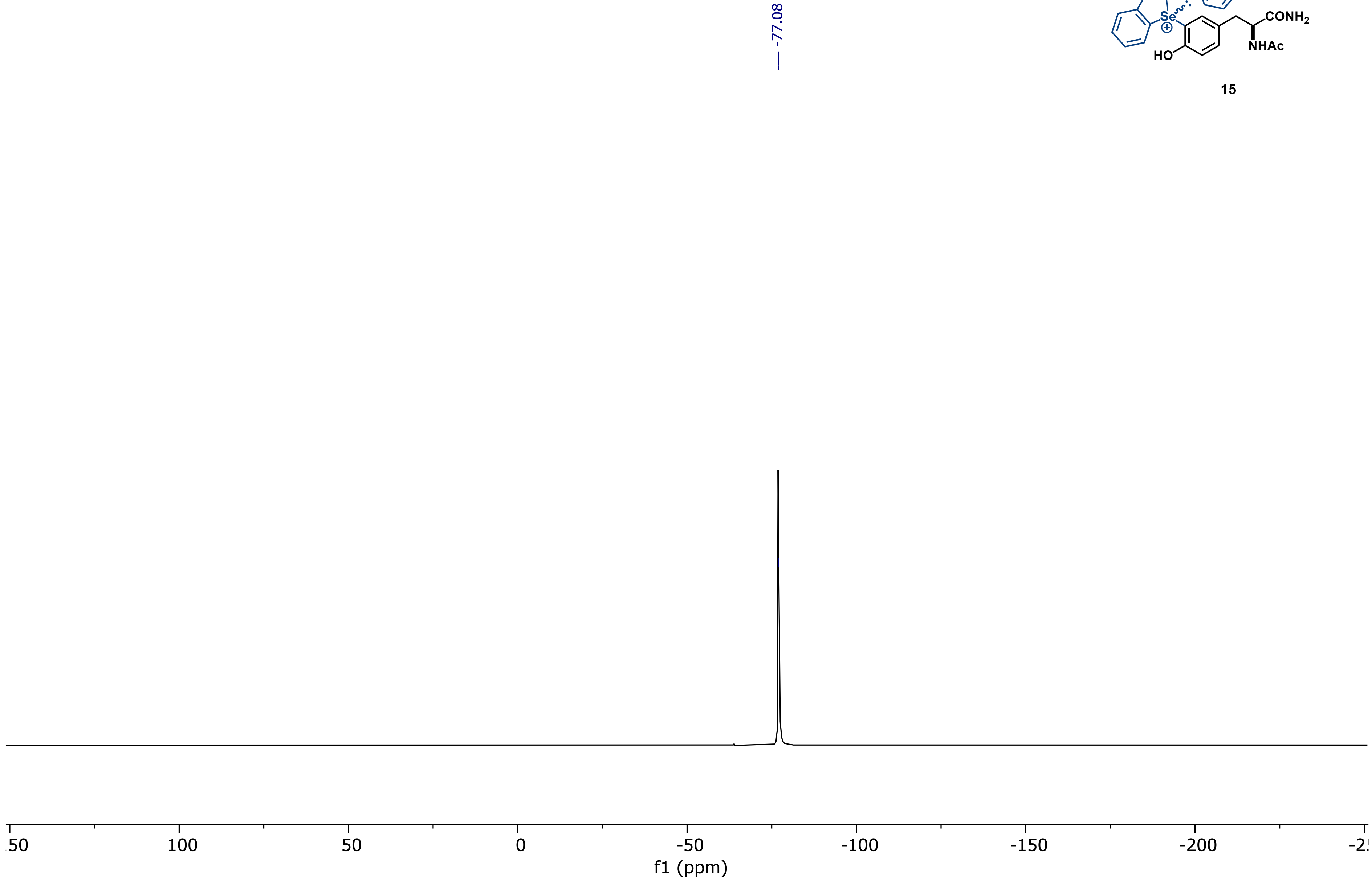

^{77}Se NMR of 15

CD3OD, 115 MHz, 298 K

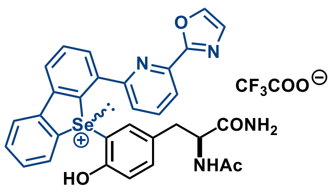

15

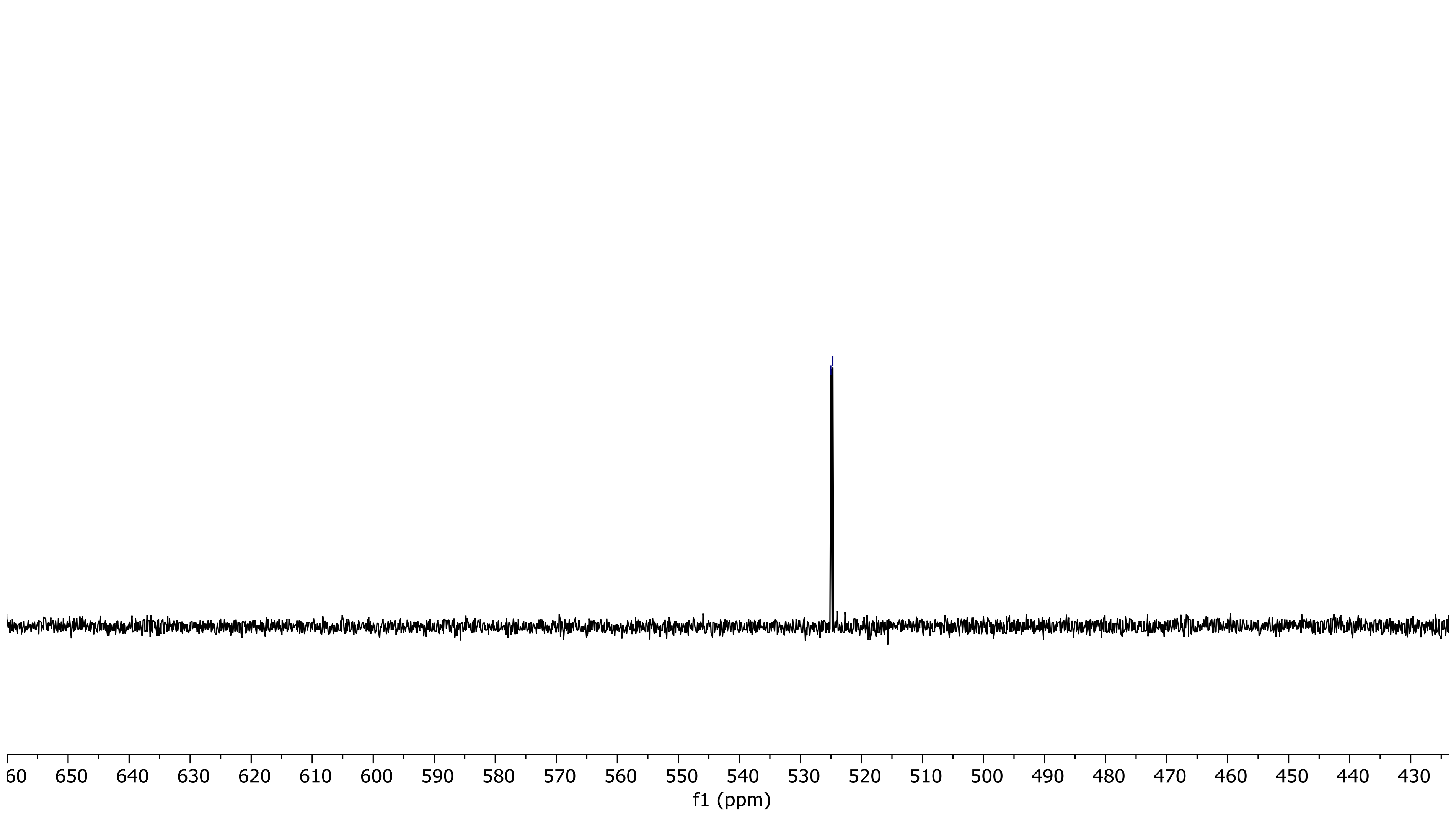

^{1}H NMR of S16

CDCl$_3$, 500 MHz, 298 K

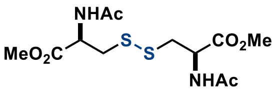

S16

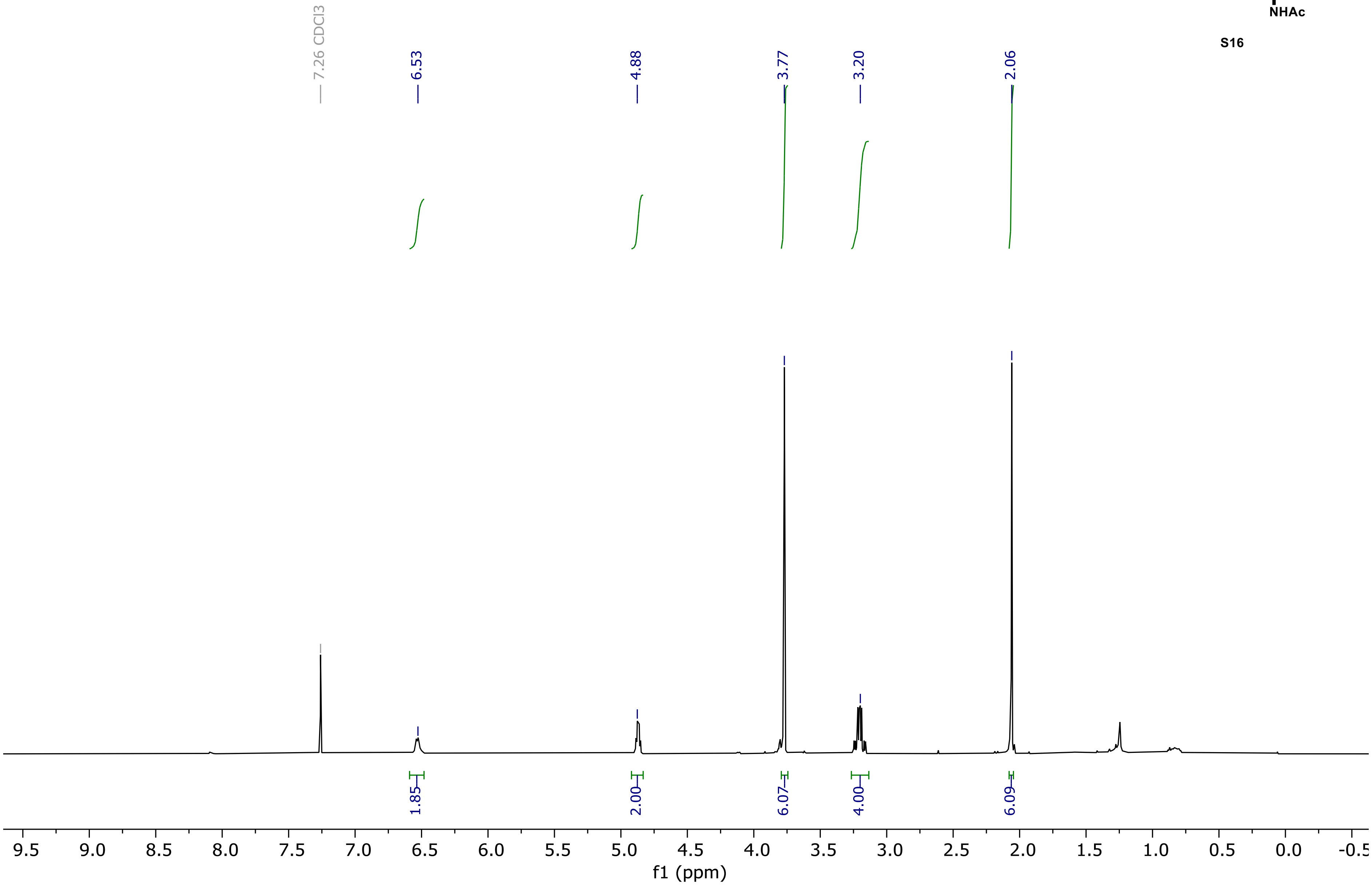

^{13}C NMR of S16

CDCl$_3$, 126 MHz, 298 K

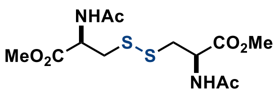

S16

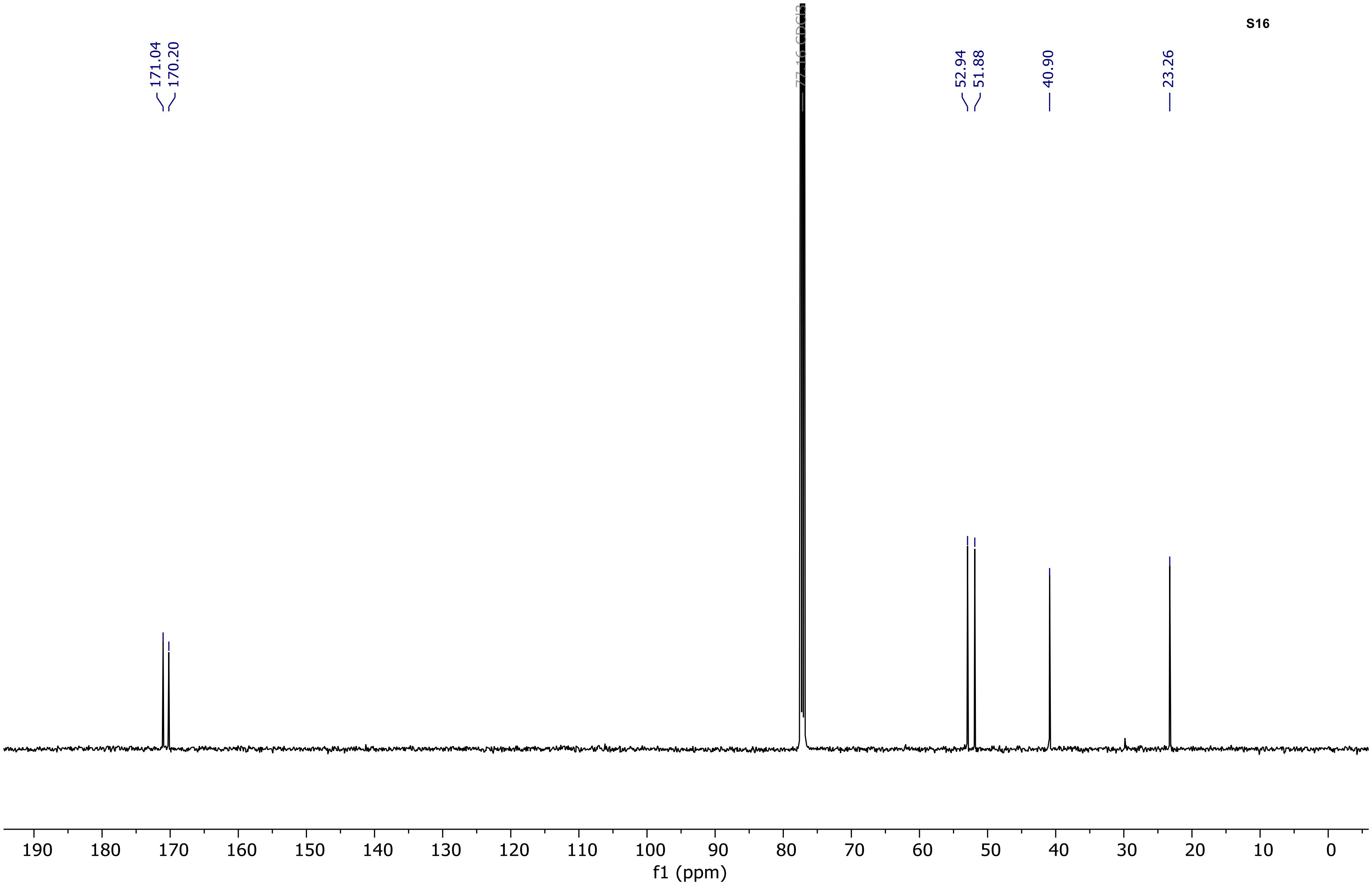

^{1}H NMR of 16

CD3OD, 500 MHz, 298 K

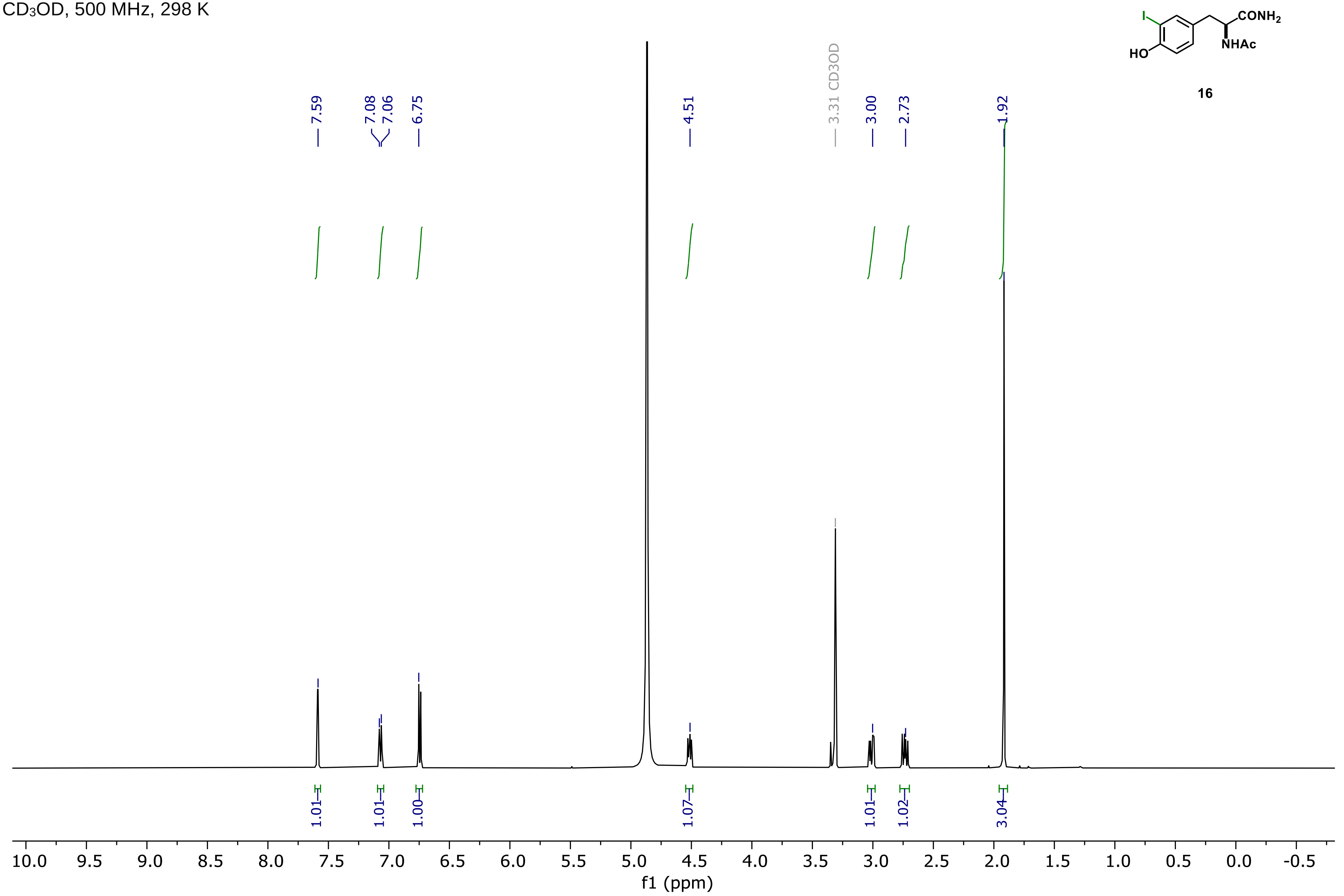

16

^{13}C NMR of 16

CD3OD, 126 MHz, 298 K

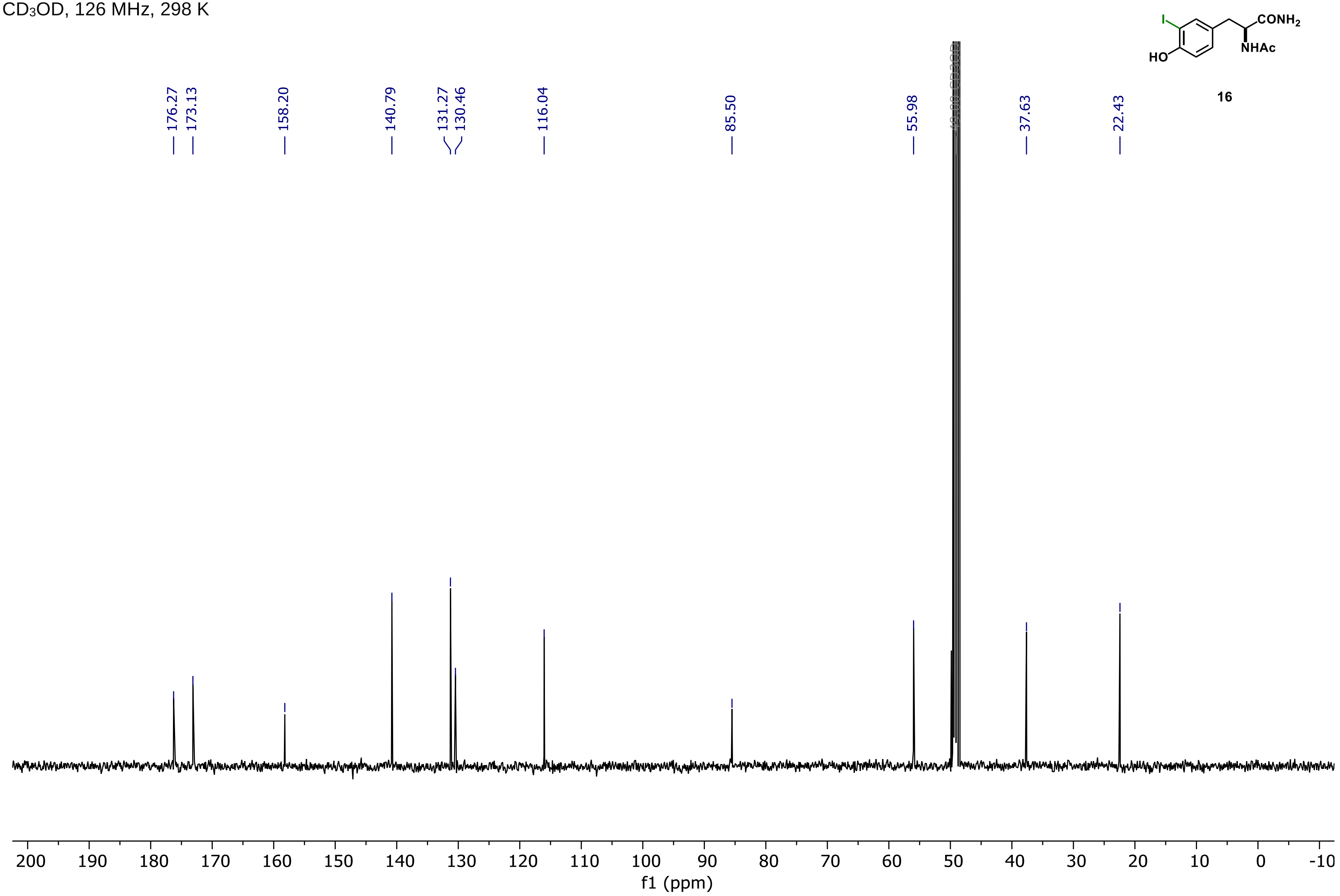

^{1}H NMR of 17

CD3OD, 500 MHz, 298 K

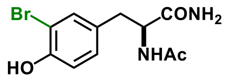

17

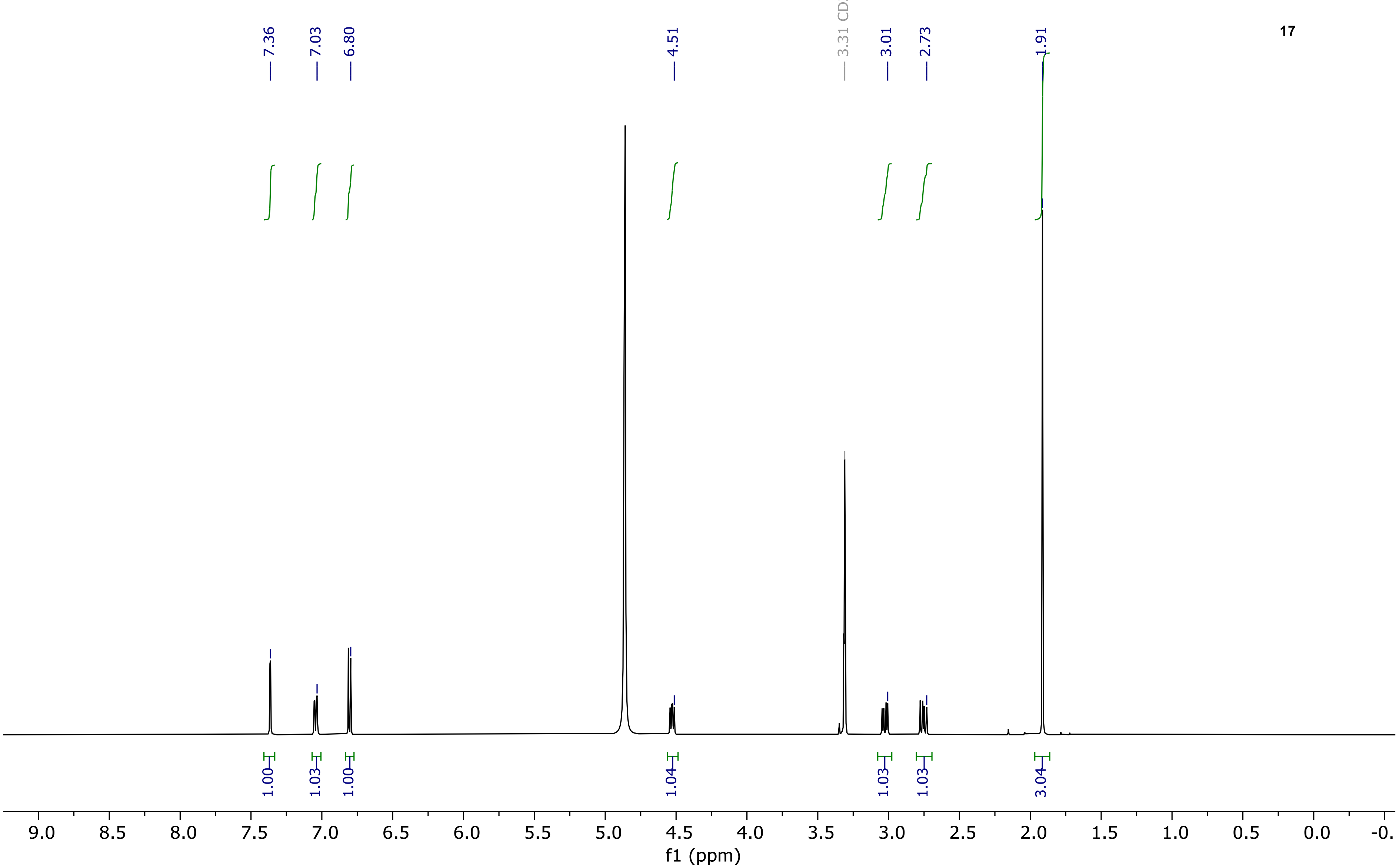

^{13}C NMR of 17

CD_3OD, 126 MHz, 298 K

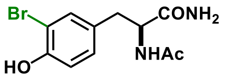

17

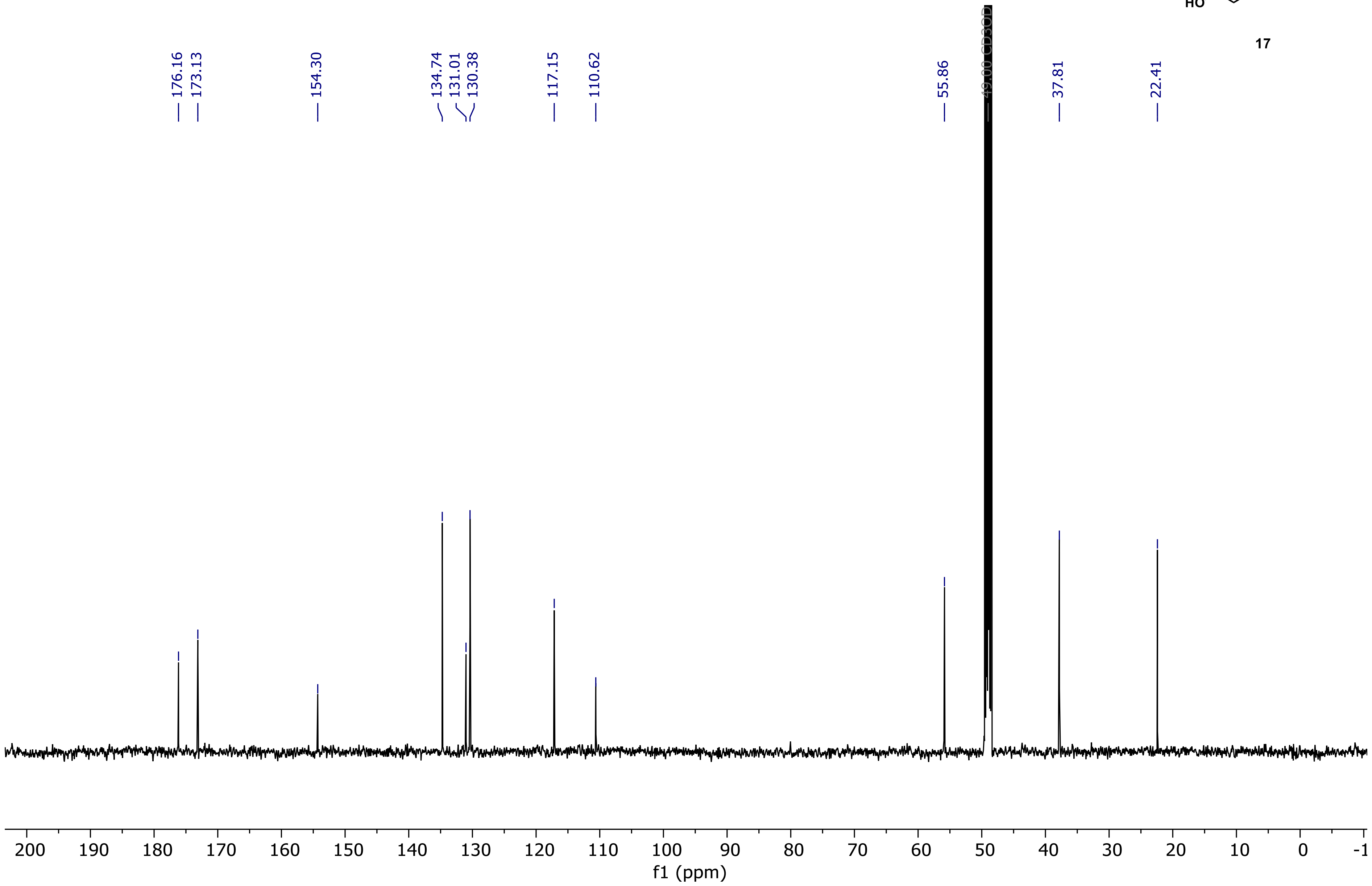

^{1}H NMR of 18

CD$_3$OD, 500 MHz, 298 K

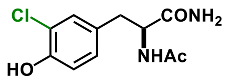

18

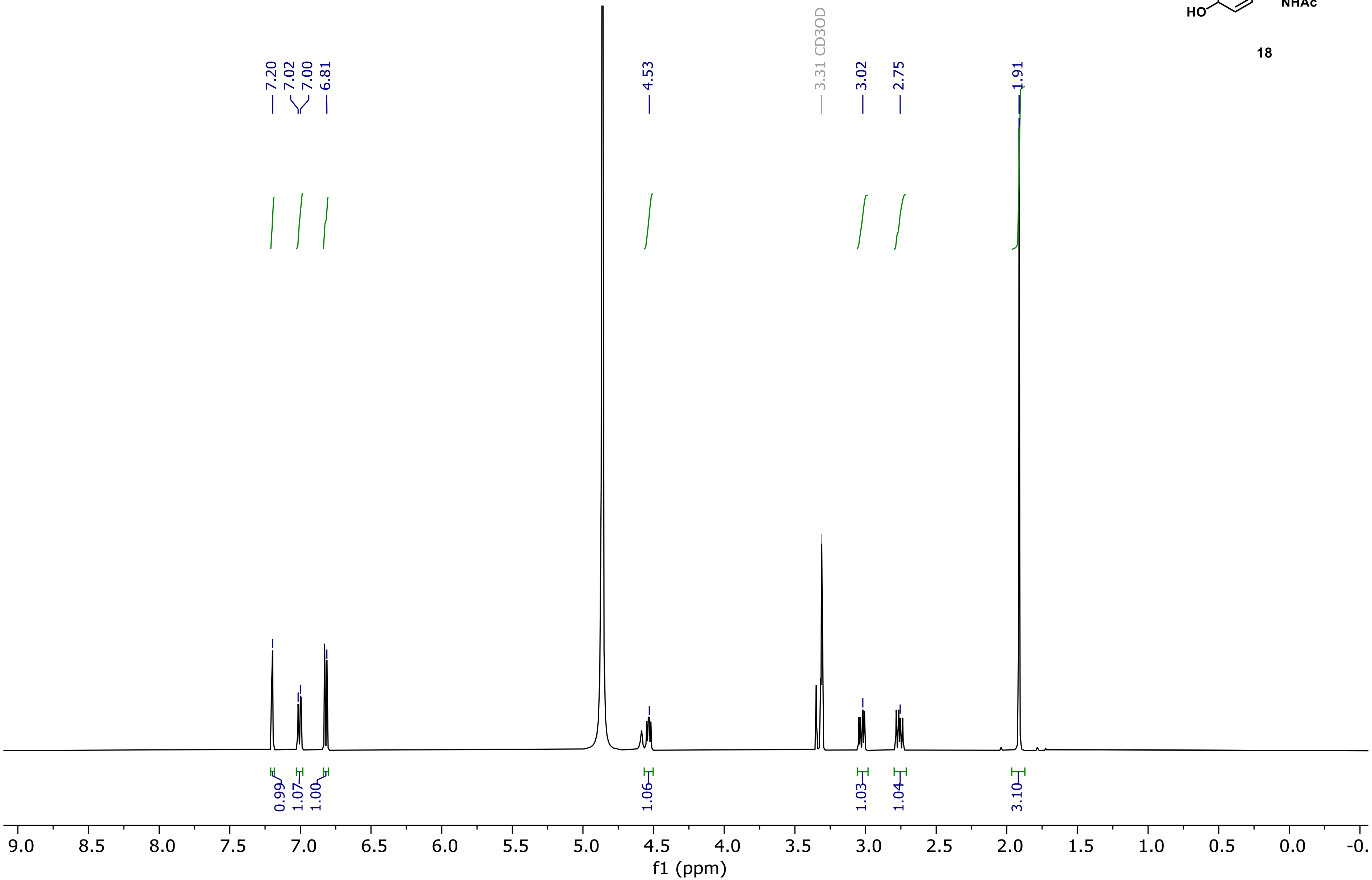

^{13}C NMR of 18

CD_3OD, 126 MHz, 298 K

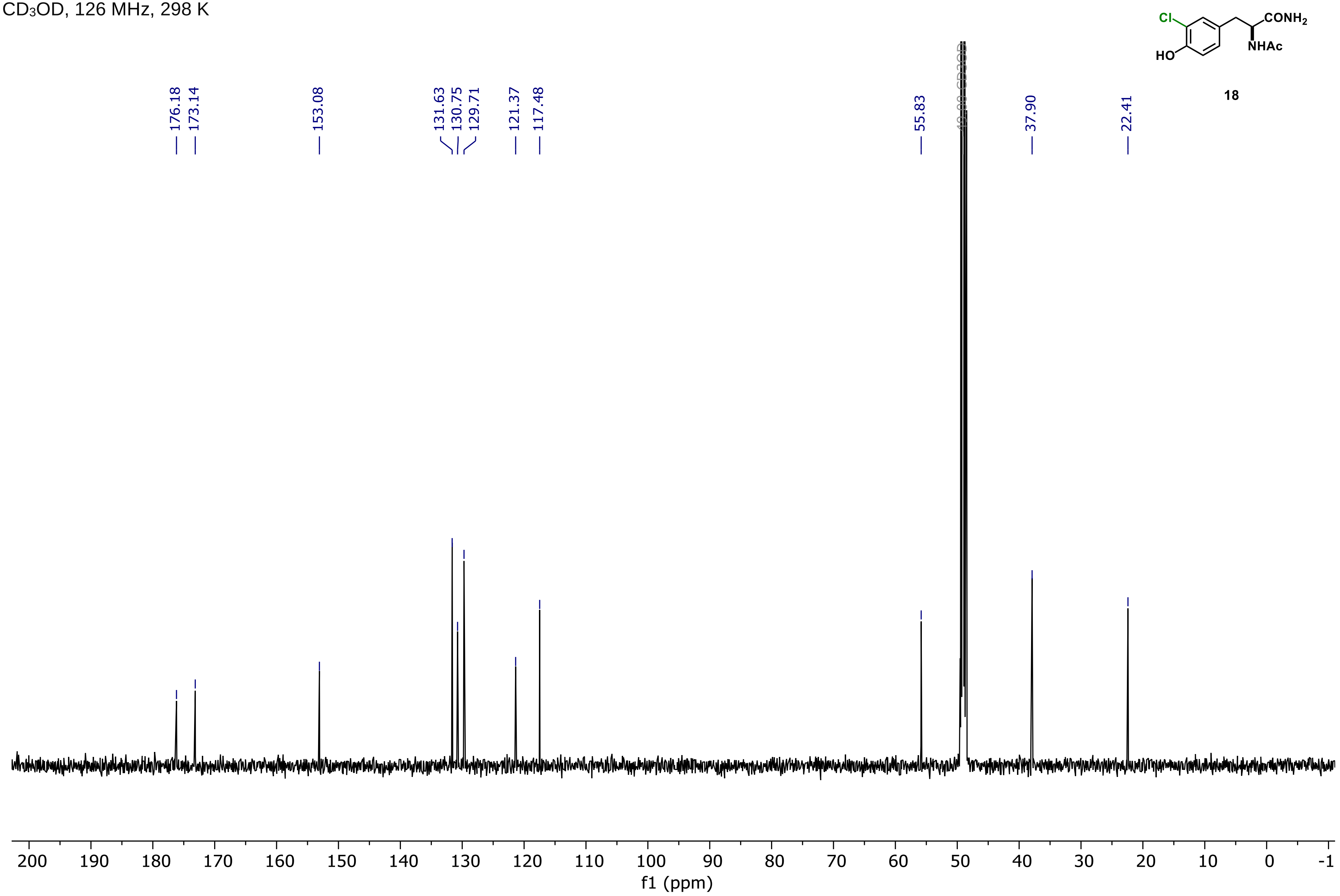

^{1}H NMR of 19

CD3OD, 600 MHz, 298 K

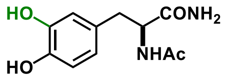

19

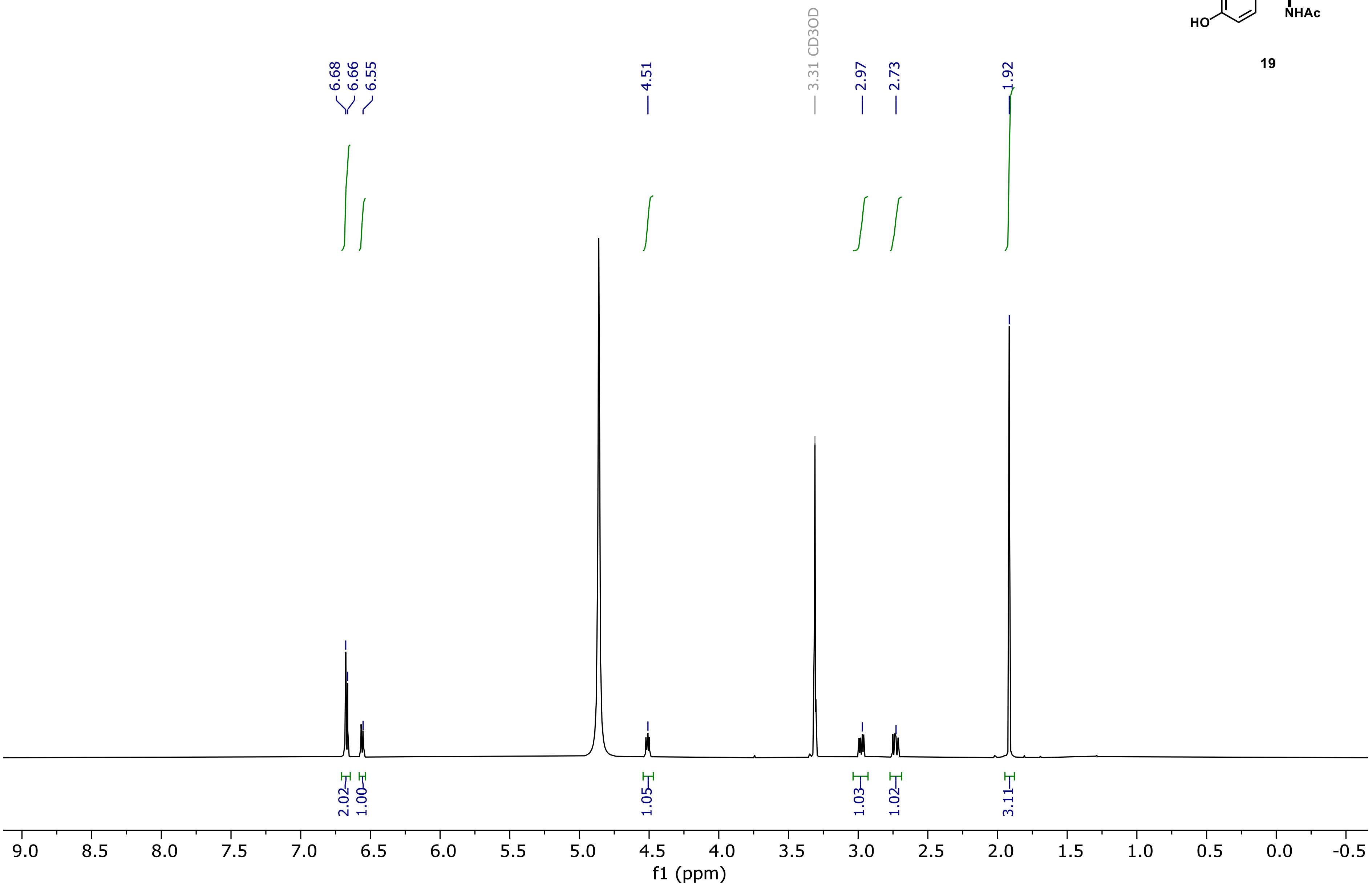

^{13}C NMR of 19

CD_3OD, 151 MHz, 298 K

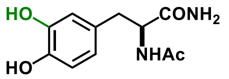

19

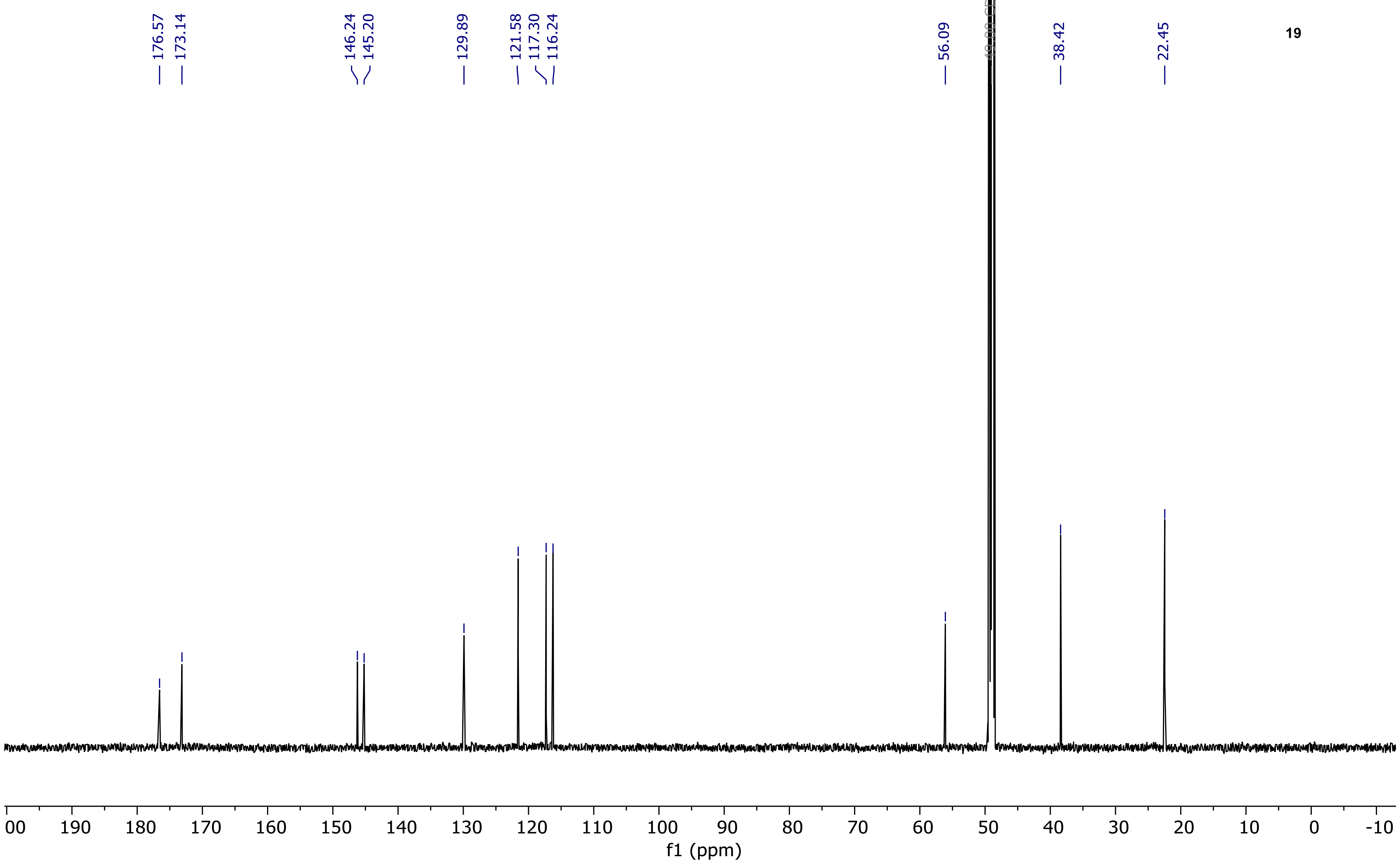

^{1}H NMR of 20

DMSO-d_6, 600 MHz, 298 K

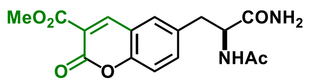

20

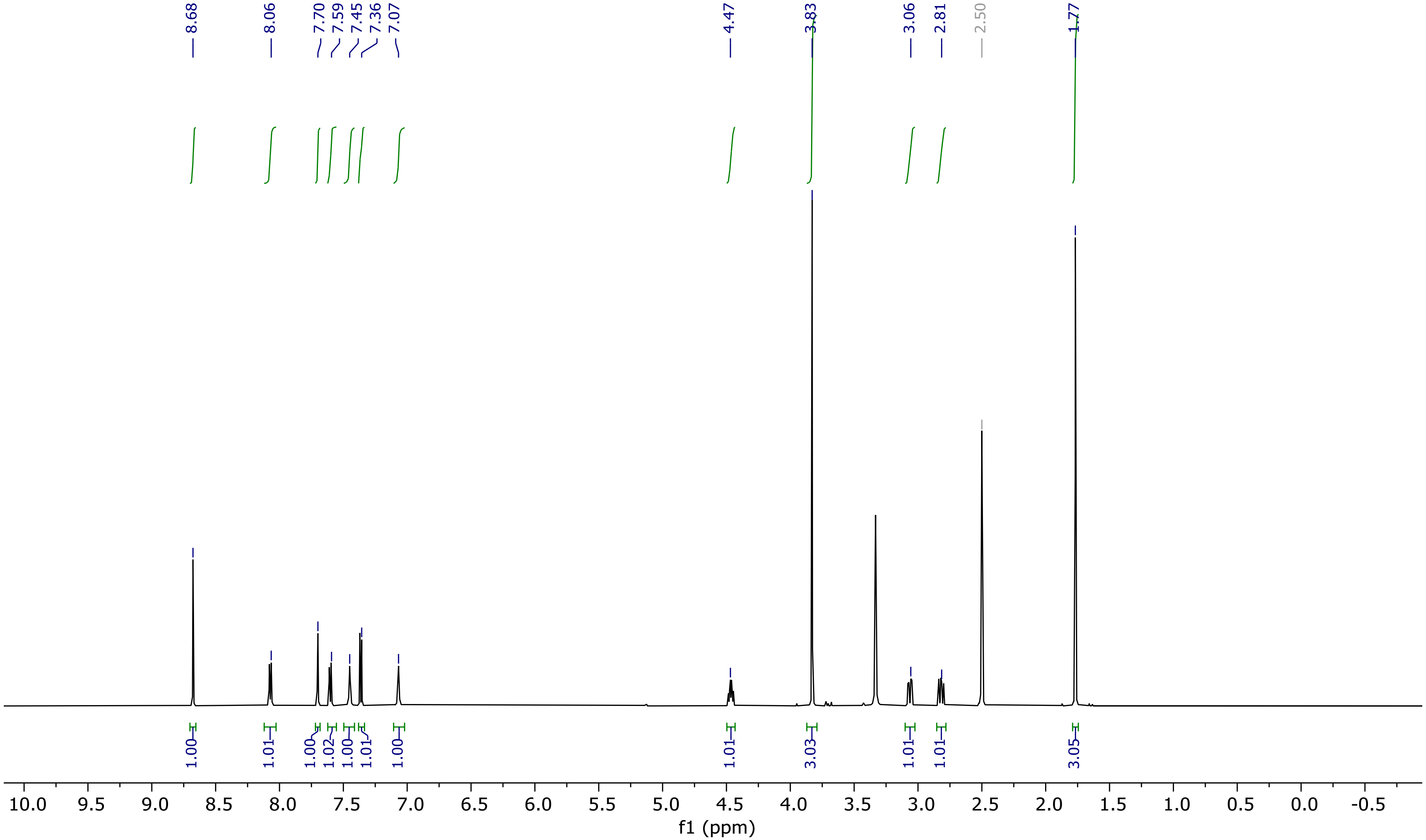

^{13}C NMR of 20

DMSO-d_6, 151 MHz, 298 K

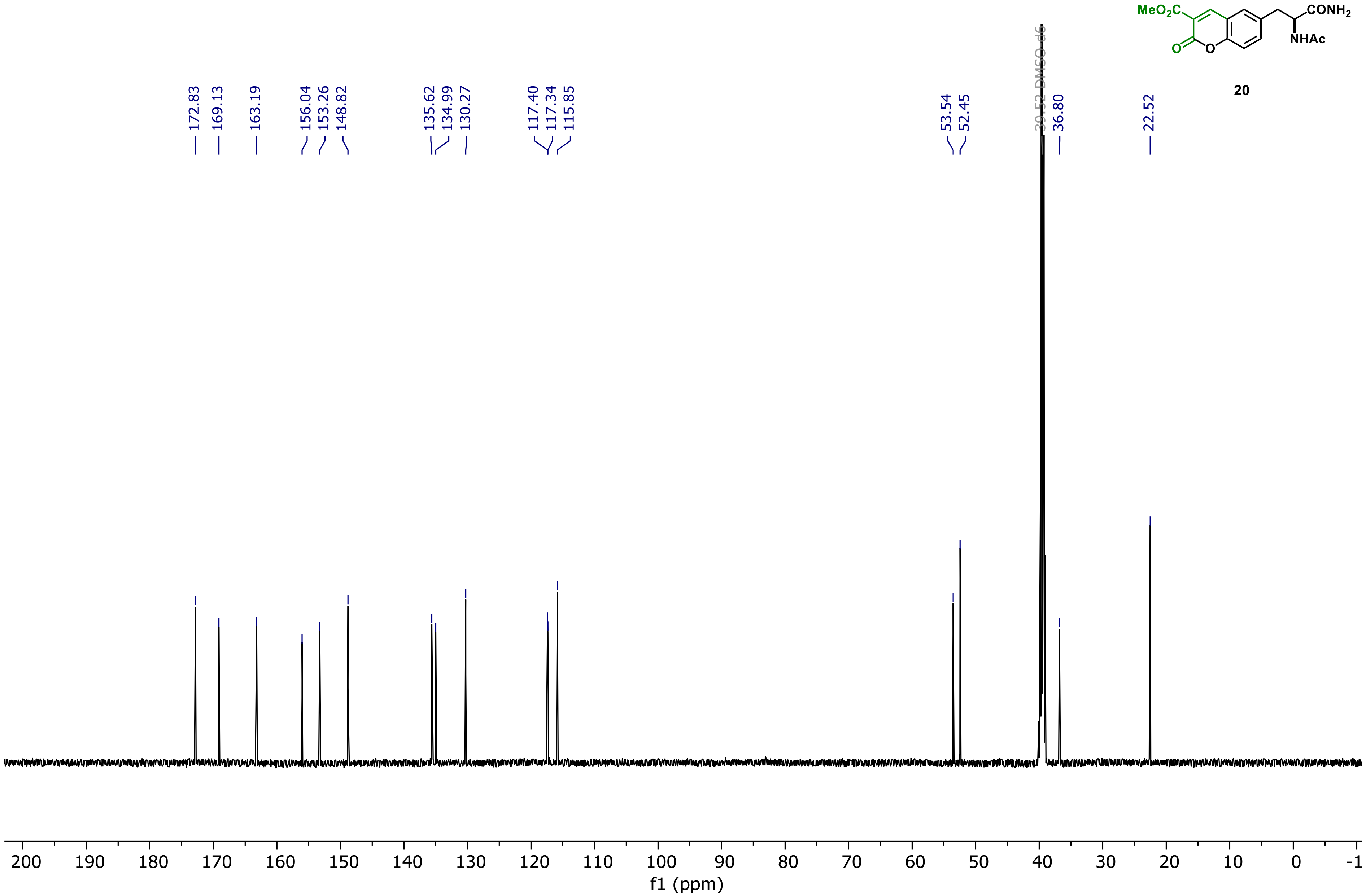

^{1}H NMR of 21

CD_3OD, 500 MHz, 298 K

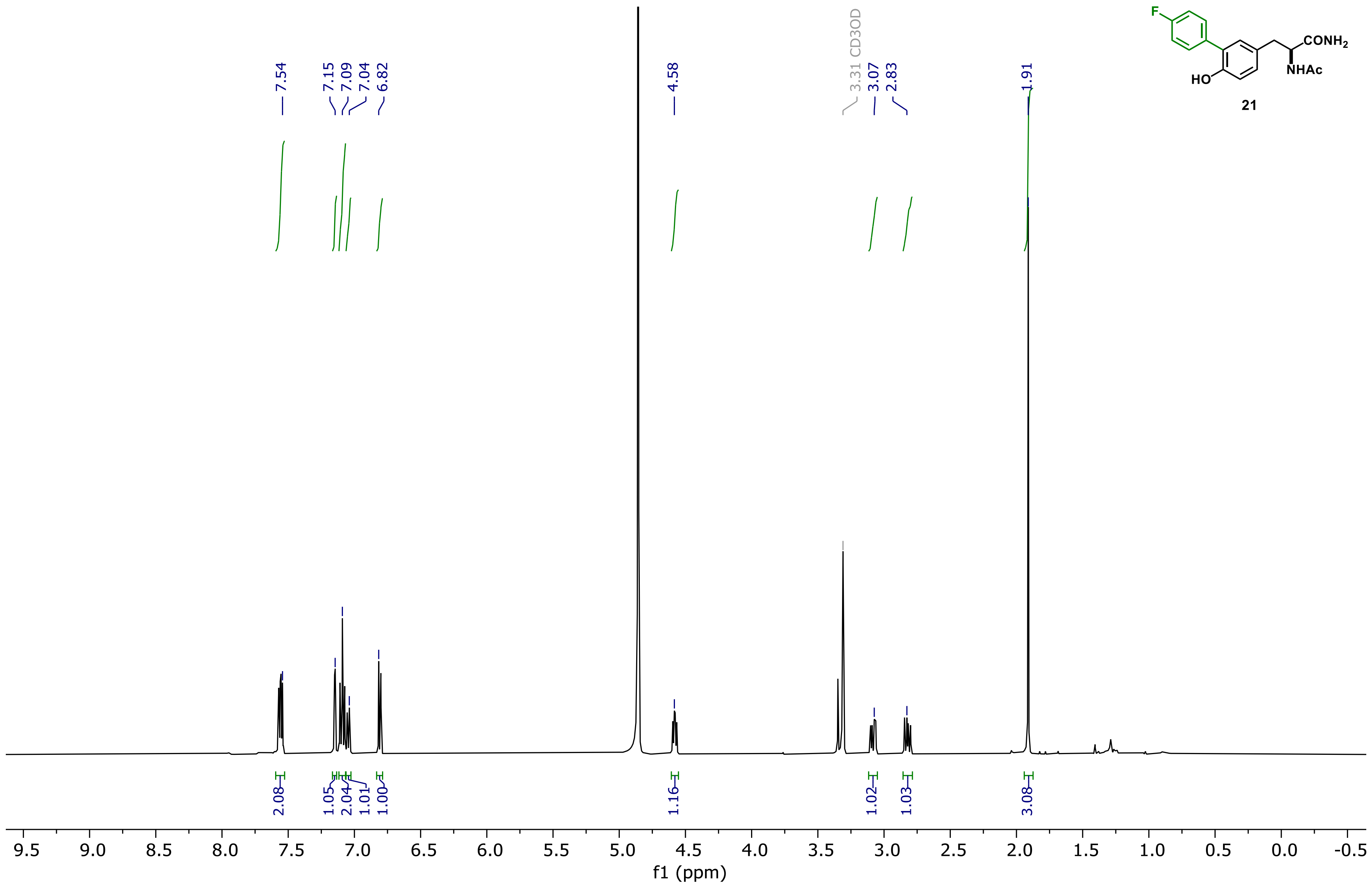

^{13}C NMR of 21

CD_3OD, 126 MHz, 298 K

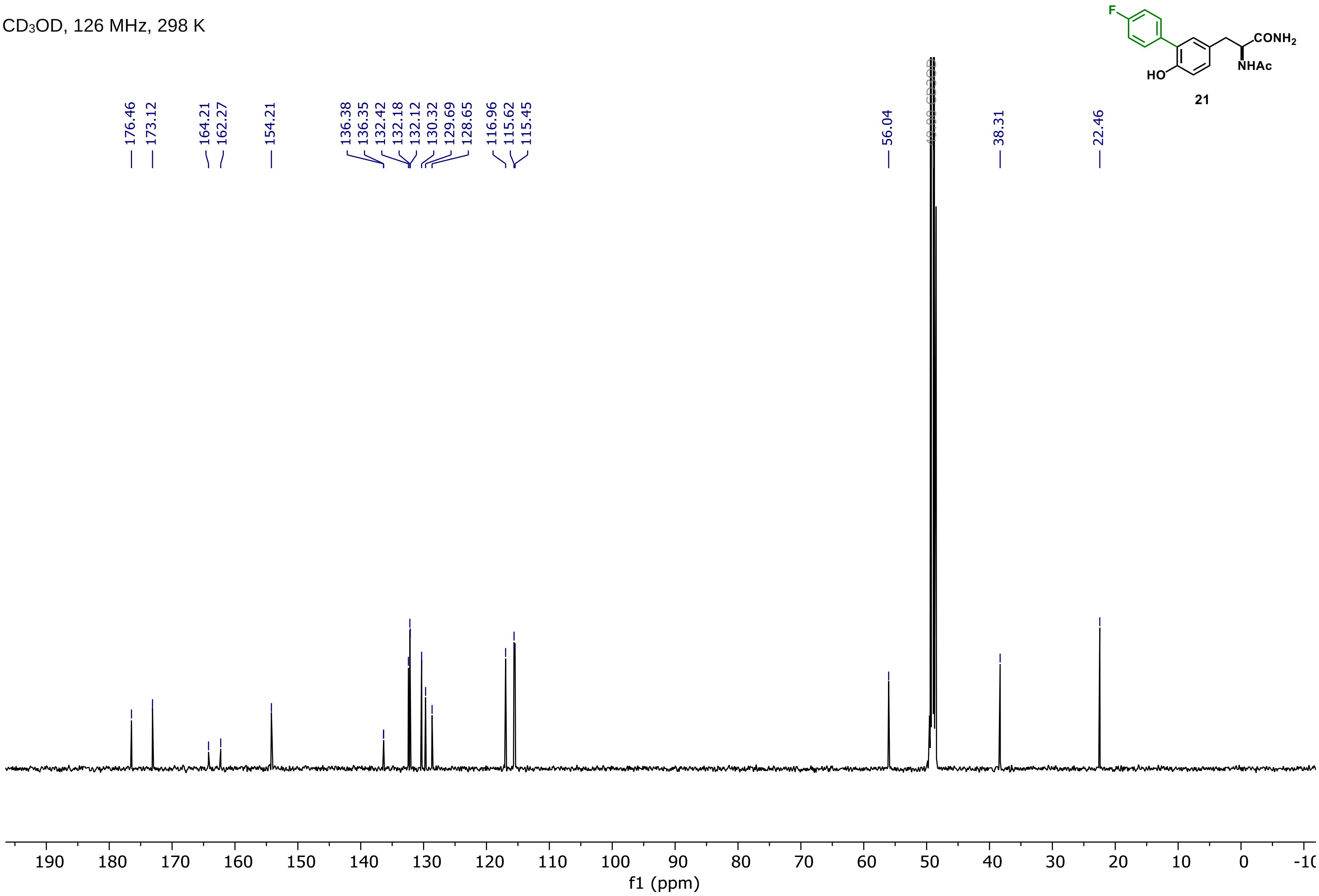

^{19}F NMR of 21

CD_3OD, 126 MHz, 298 K

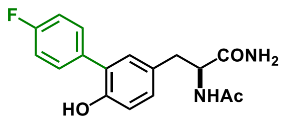

21

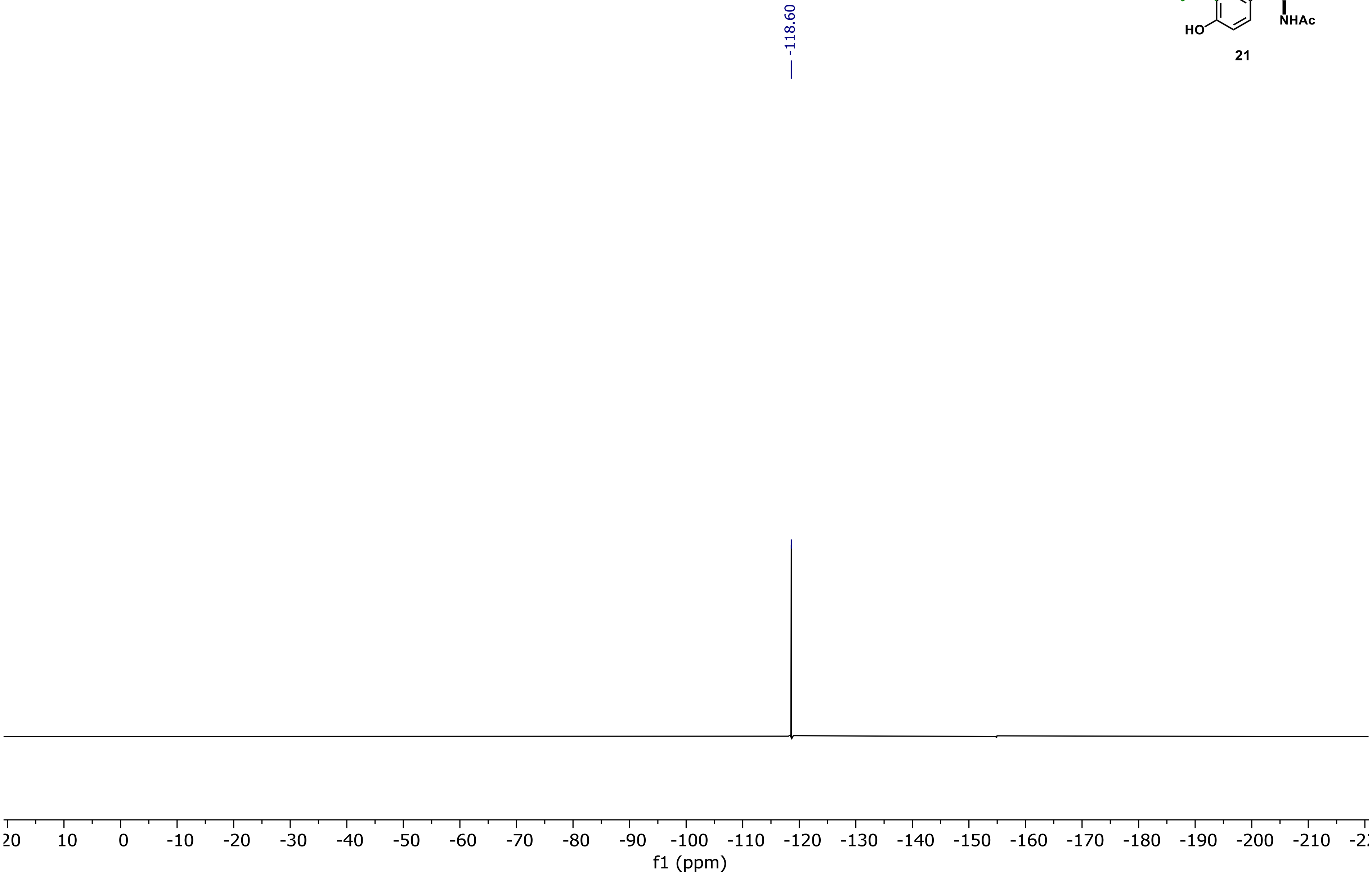

NMR signal assignment of 22

DMF-d_7, 298 K

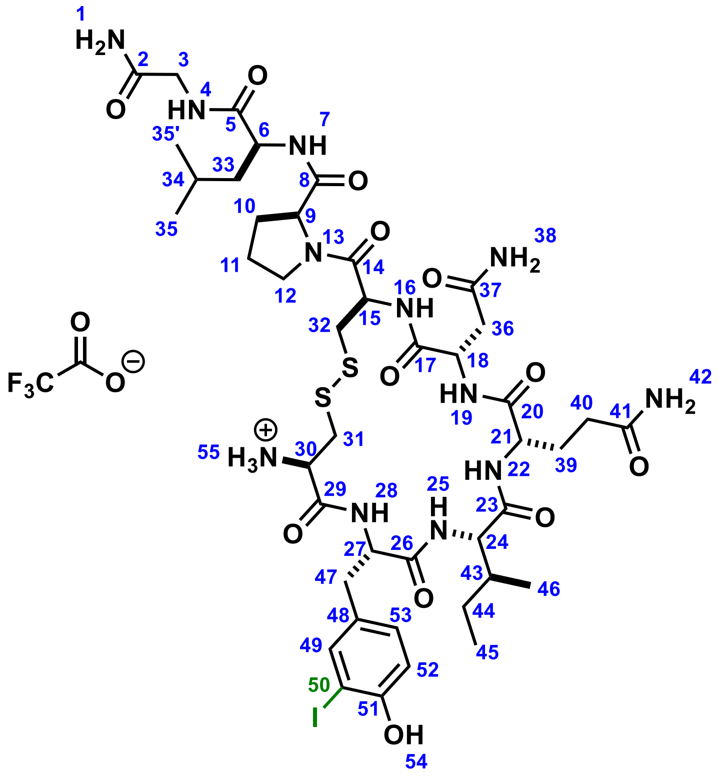

22

Atom	δ (ppm)	Min..Max (ppm)	J	COSY	HSQC	HMBC	NOESY
1 N							
H2	7.11	7.10..7.11				3	
3 C	42.66	42.65..42.67			3a, 3b	1, 4	
Ha	3.87	3.84..3.89		3b, 4	3		4
Hb	3.71	3.68..3.74		3a, 4	3		4
4 N							
H	7.94	7.94..7.95		3a, 3b		3	3a, 3b, 6

6 C	52.25	52.24..52.26			6		
H	4.34	4.34..4.35	7.80(7)	7, 33	6	8, 33, 34	4, 7
7 N							
H	8.2	8.19..8.21	7.80(6)	6		8	6, 9, 10a, 10b, 33
8 C	172.89	172.89..172.90				6, 7, 9	
9 C	61.67	61.66..61.69			9		
H	4.44	4.44..4.45		10a, 10b	9	8, 10, 11	7
10 C	29.38	29.38..29.38			10a, 10b	9, 11, 12	
Ha	2.18	2.18..2.19		9, 10b, 11	10		7
Hb	1.95	1.95..1.96		9, 10a, 11	10		7
11 C	25.1	25.09..25.11			11	9, 12	
H2	1.97	1.96..1.97		10a, 10b, 12	11	10	
12 C	47.58	47.57..47.58			12		
H2	3.71	3.70..3.71		11	12	10, 11	16
13 N							
14 C	169.43	169.42..169.45				15, 32b	
15 C	52.42	52.42..52.42			15	32b	
H	5.05	5.04..5.05		16, 32a, 32b	15	14, 32	16
16 N							
H	8.45	8.45..8.45		15			12, 15, 18
17 C							
18 C	51.15	51.13..51.16			18	36a, 36b	
H	4.73	4.71..4.76		19, 36a, 36b	18	36	16, 19
19 N							
H	7.95	7.95..7.95		18			18, 21
20 C	171.9	171.89..171.91				21, 39	
21 C	55.45	55.45..55.46			21	39, 40	
H	4.1	4.10..4.10		22, 39	21	20, 39, 40	19, 22
22 N							
H	8.63	8.62..8.63		21			21, 39
23 C							
24 C	60.55	60.55..60.55			24	46	
H	4.05	4.04..4.06		25, 43	24		25

25 N							
H	8.62	8.62..8.62		24			24, 27, 43, 46
26 C							
27 C	55.34	55.34..55.34			27		
H	4.8	4.80..4.80		47a, 47b	27		25, 49, 53
32 C	42.03	42.03..42.03			32a, 32b	15	
Ha	3.3	3.30..3.30		15, 32b	32		
Hb	3.18	3.18..3.18		15, 32a	32	14, 15	
33 C	40.01	40.00..40.02			33	6, 34, 35, 35'	
H2	1.67	1.67..1.67		6, 34	33	34	7
34 C	24.94	24.93..24.94			34	6, 33, 35, 35'	
H	1.69	1.69..1.70		33, 35, 35'	34	33, 35, 35'	
35 C	23.07	23.07..23.08			35	34, 35'	
H3	0.9	0.88..0.92		34	35	33, 34, 35'	
35' C	21.12	21.12..21.13			35'	34, 35	
H3	0.86	0.85..0.87		34	35'	33, 34, 35	
36 C	36.9	36.89..36.91			36a, 36b	18, 38	
Ha	2.9	2.89..2.90		18, 36b	36	18	
Hb	2.78	2.77..2.79		18, 36a	36	18	
37 C							
38 N							
H2	7.07	7.07..7.08				36	
39 C	26.67	26.65..26.68			39	21, 40	
H2	2.04	2.04..2.04		21, 40	39	20, 21, 40, 41	22
40 C	31.85	31.84..31.86			40	21, 39, 42	
H2	2.37	2.31..2.43		39	40	21, 39, 41	
41 C	175.4	175.39..175.41				39, 40	
42 N							
H2	6.92	6.92..6.93				40	
43 C	36.54	36.53..36.55			43	44b, 45, 46	
H	1.93	1.93..1.94	6.80(46)	24, 44a, 44b, 46	43		25

^{1}H NMR of 22

DMF-d_7, 600 MHz, 298 K

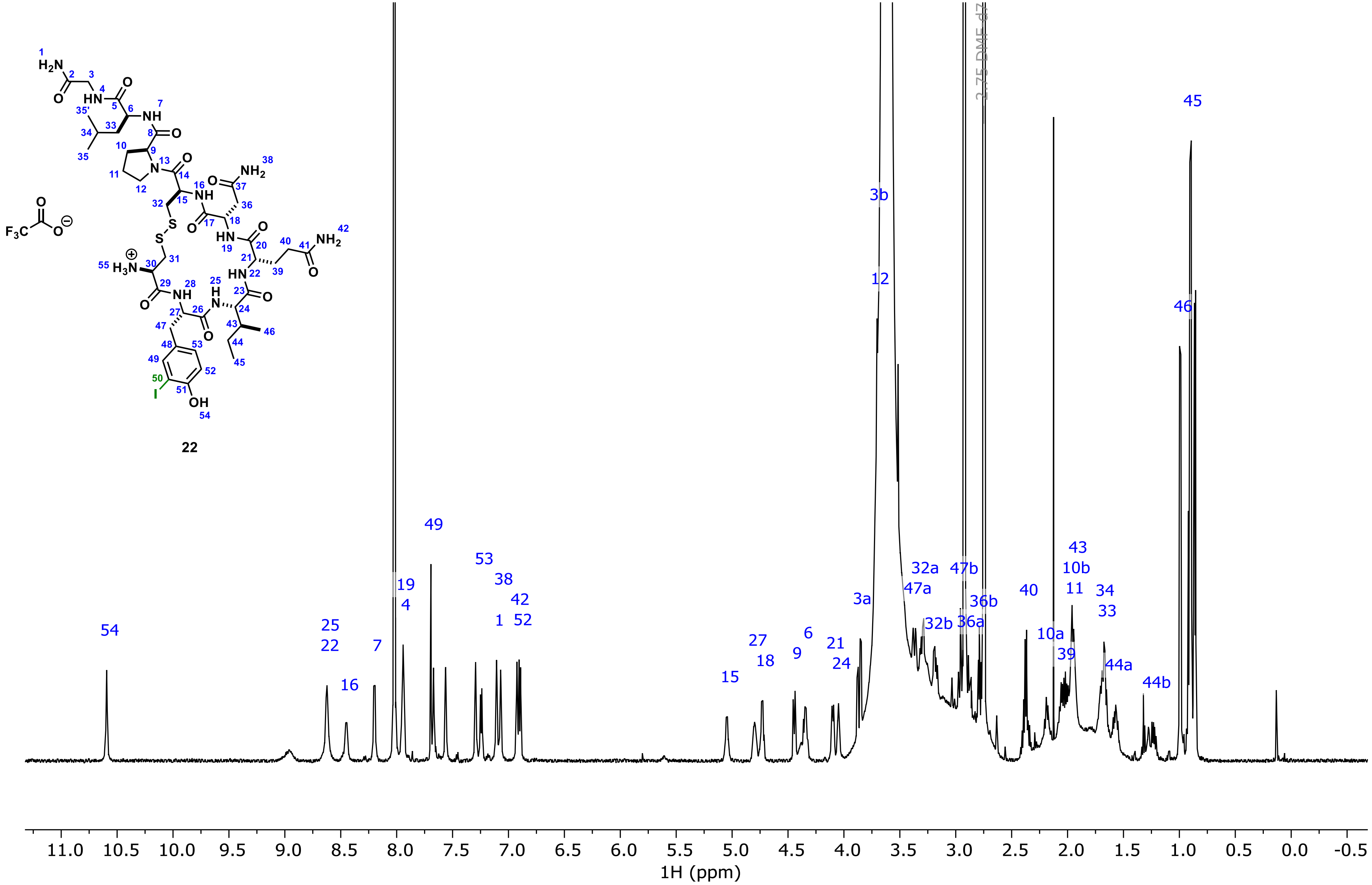

^{13}C NMR of 22

DMF-d_7, 151 MHz, 298 K

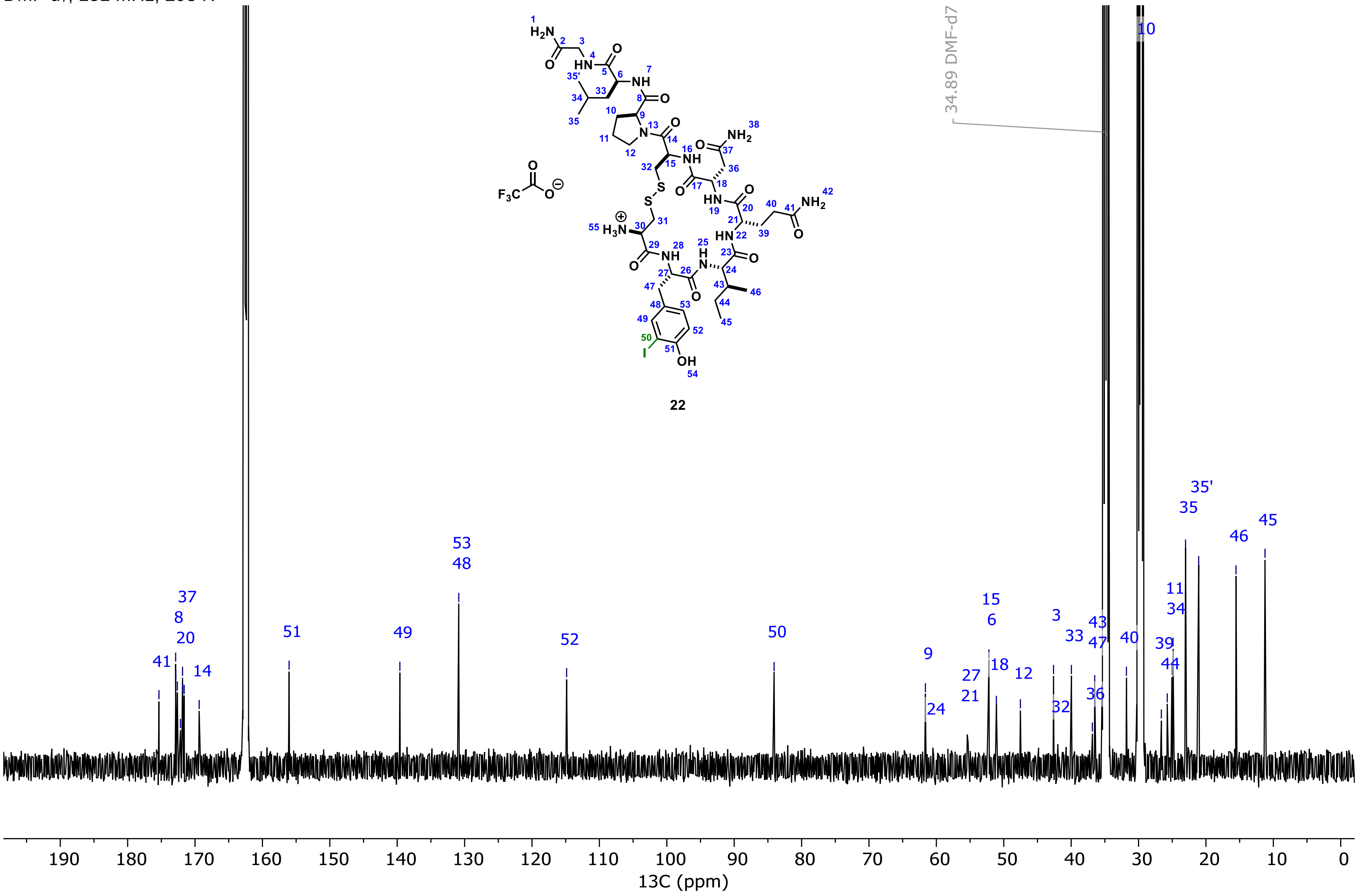

^{19}F NMR of 22

DMF-d_7, 565 MHz, 298 K

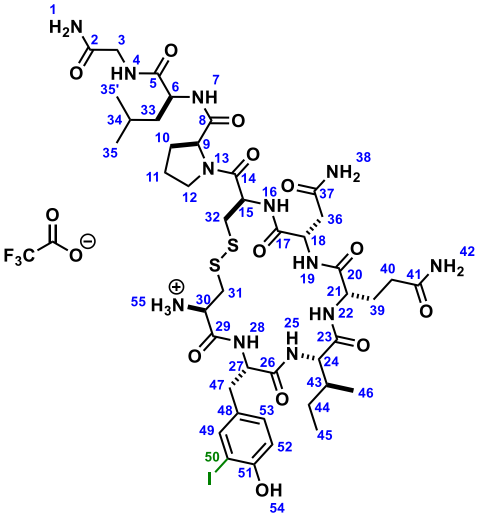

22

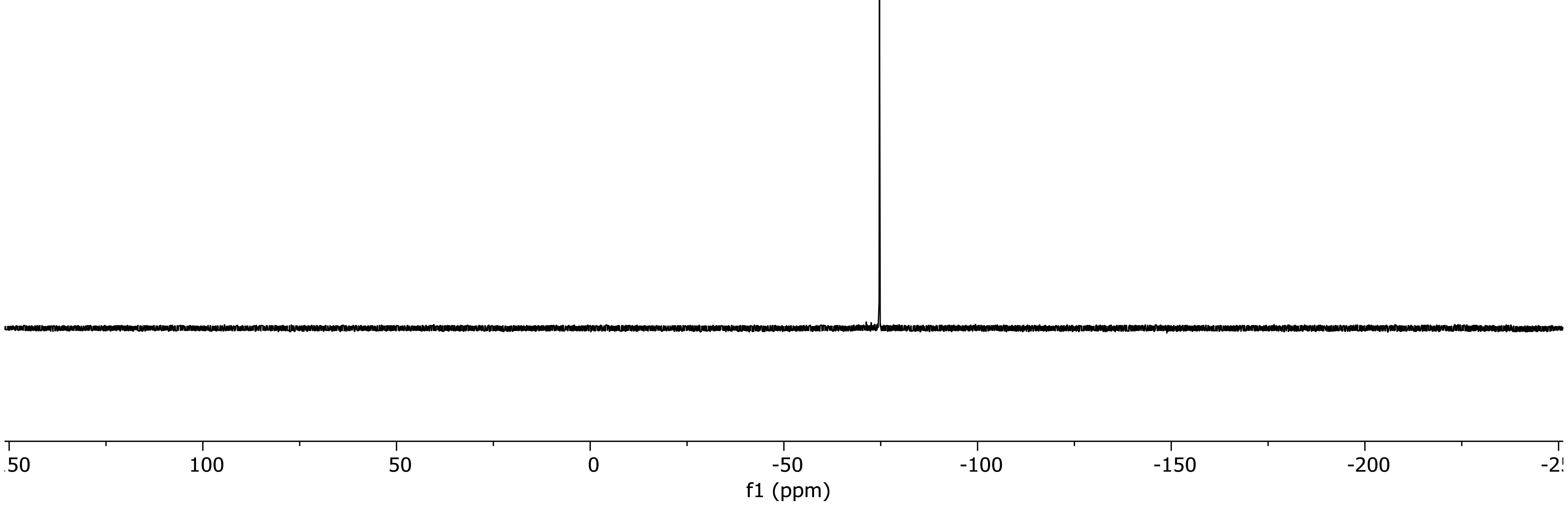

^{1}H-^{13}C HSQC NMR of 22

DMF-d_7, 298 K

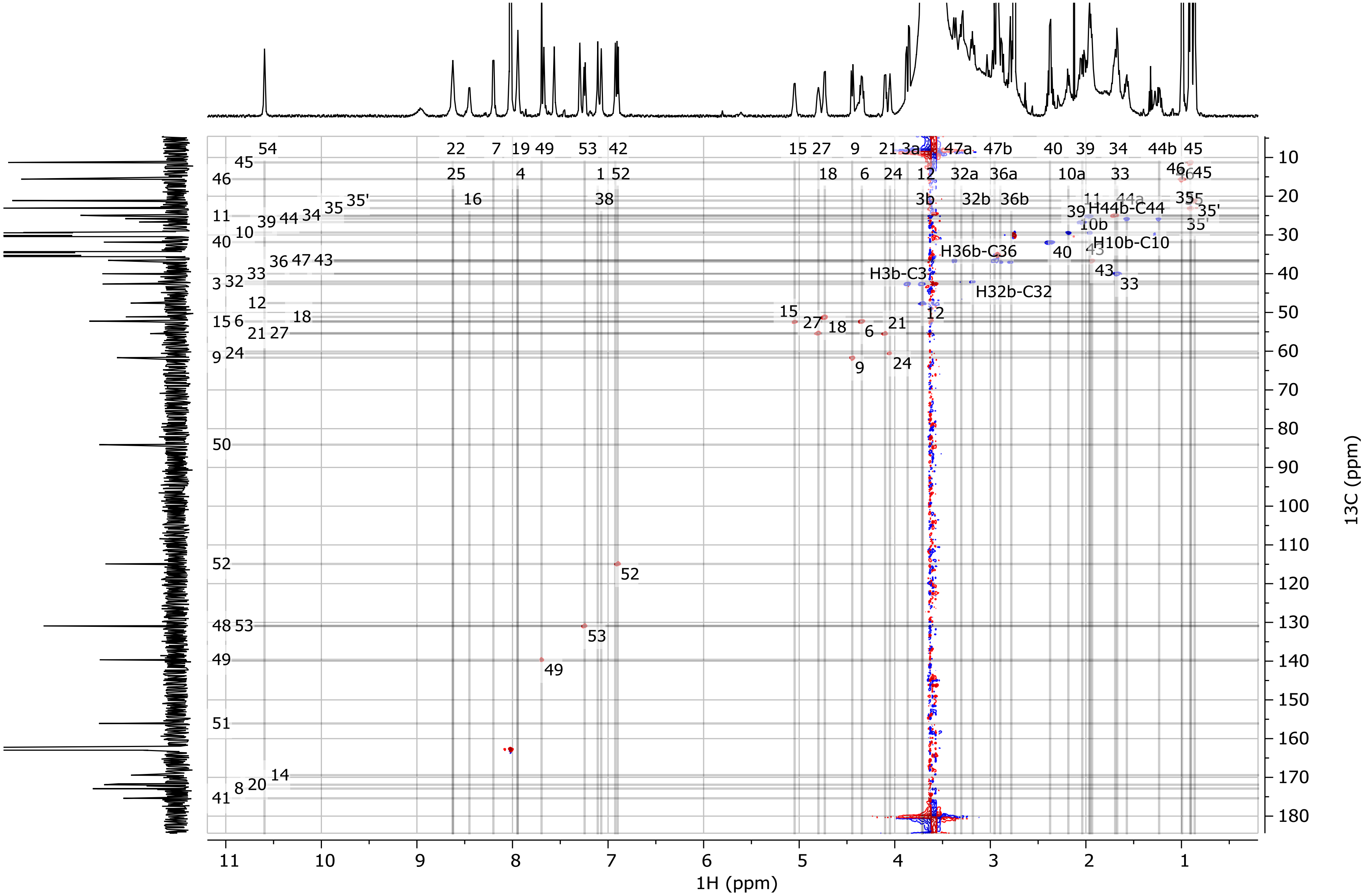

^{1}H-^{13}C HMBC NMR of 22

DMF-d_7, 298 K

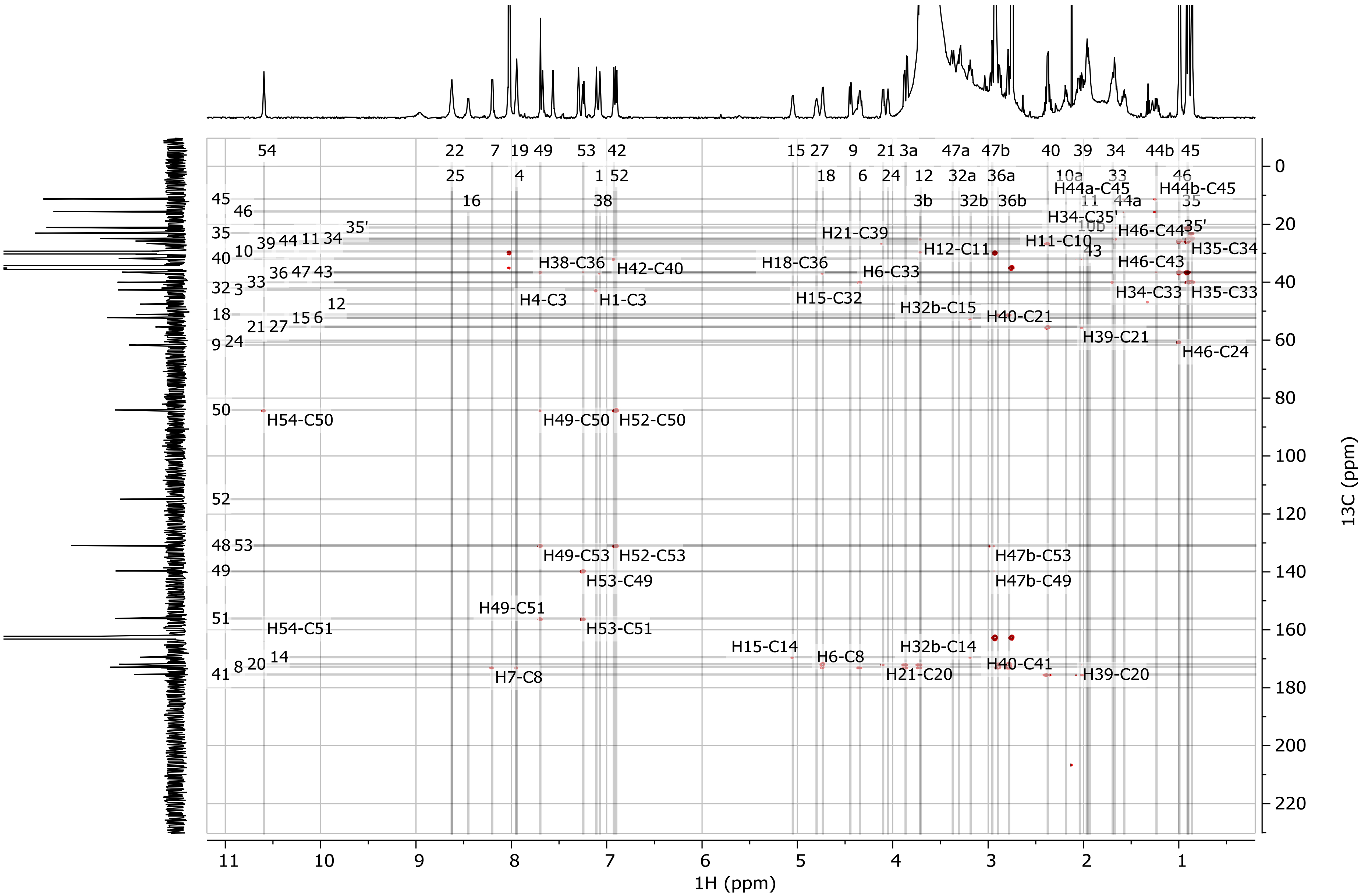

COSY NMR of 22

DMF-d_7, 298 K

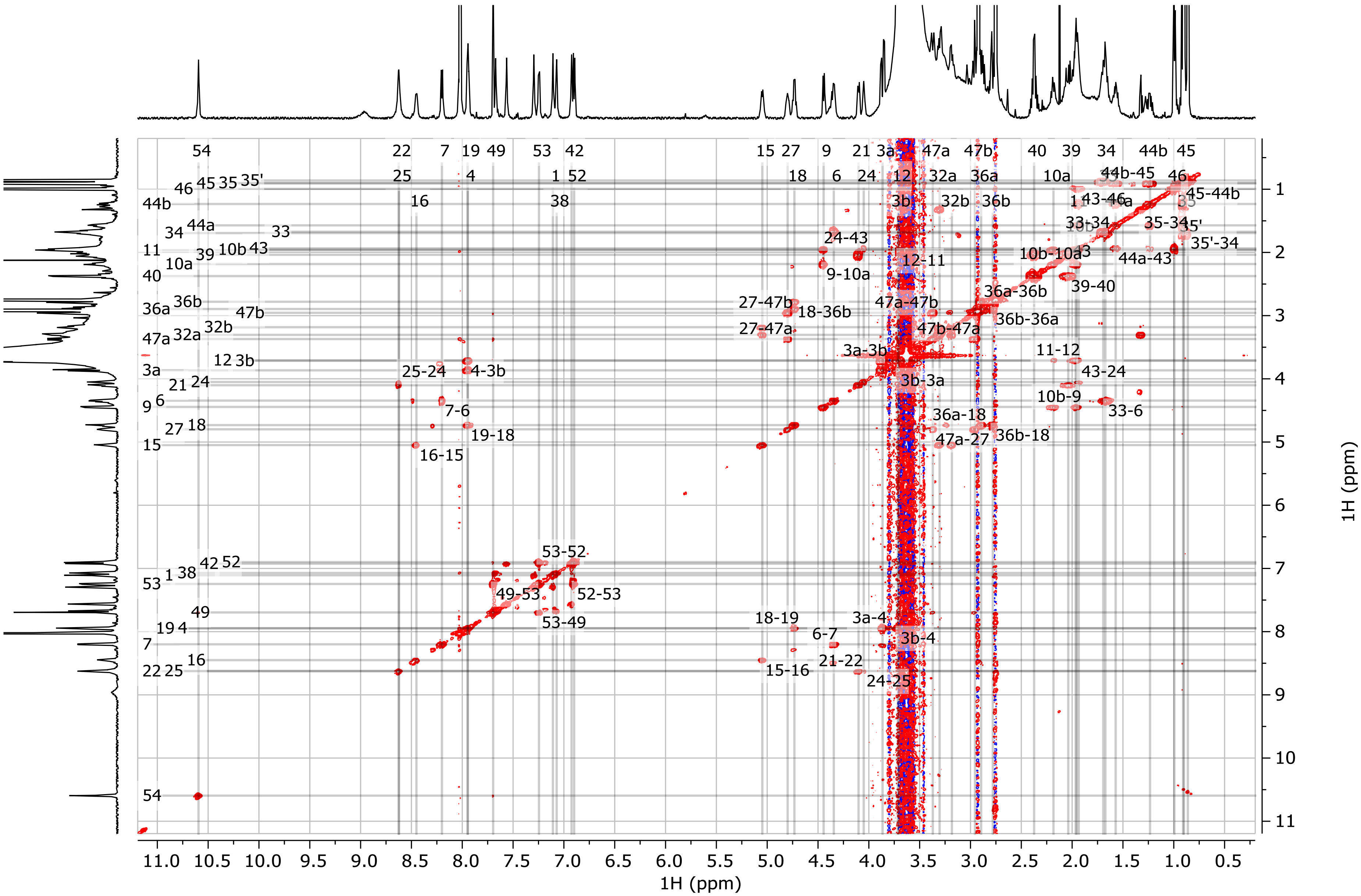

NOESY NMR of 22

DMF-d_7, 298 K

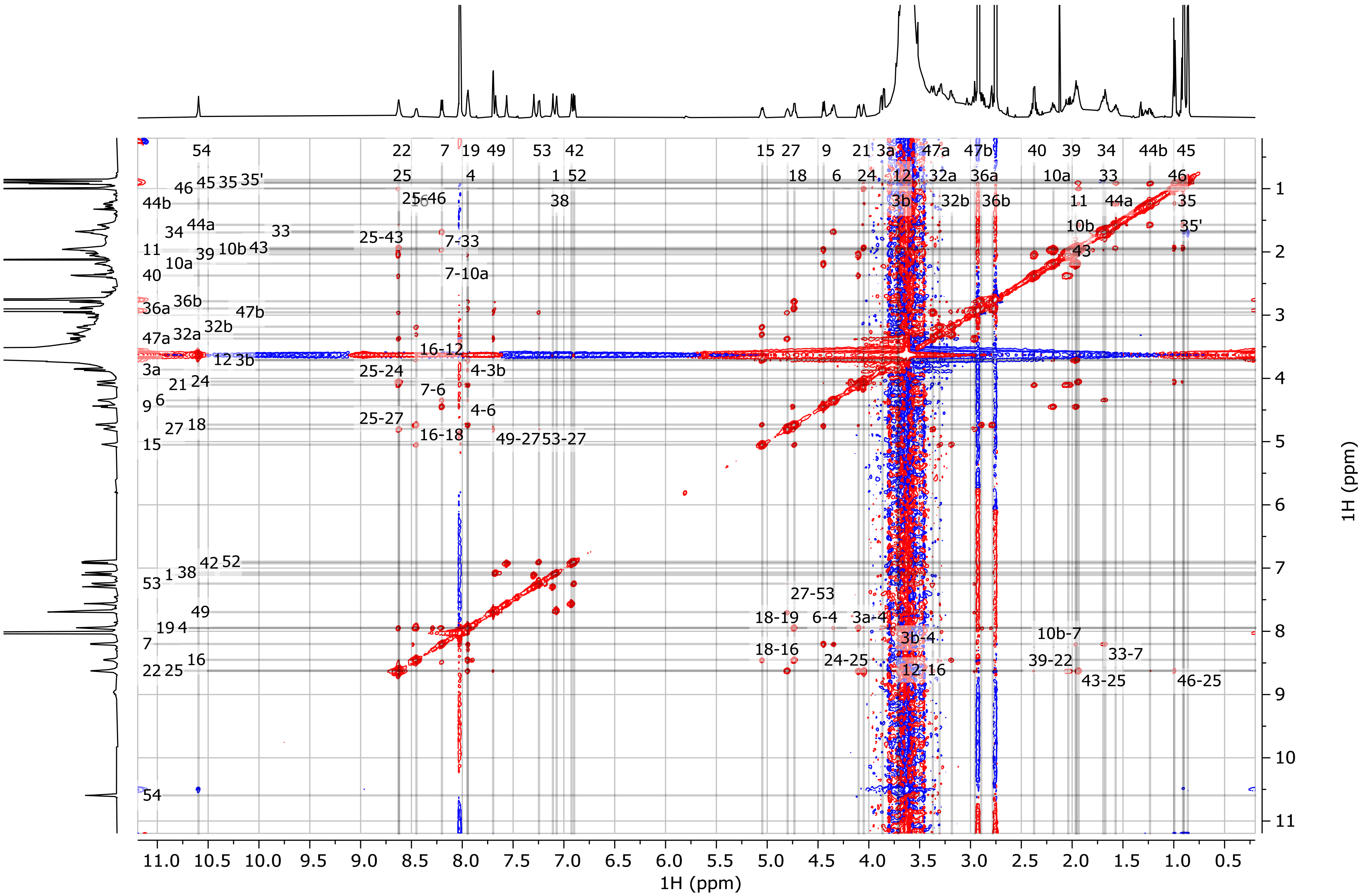

ROESY NMR of 22

DMF-d_7, 298 K

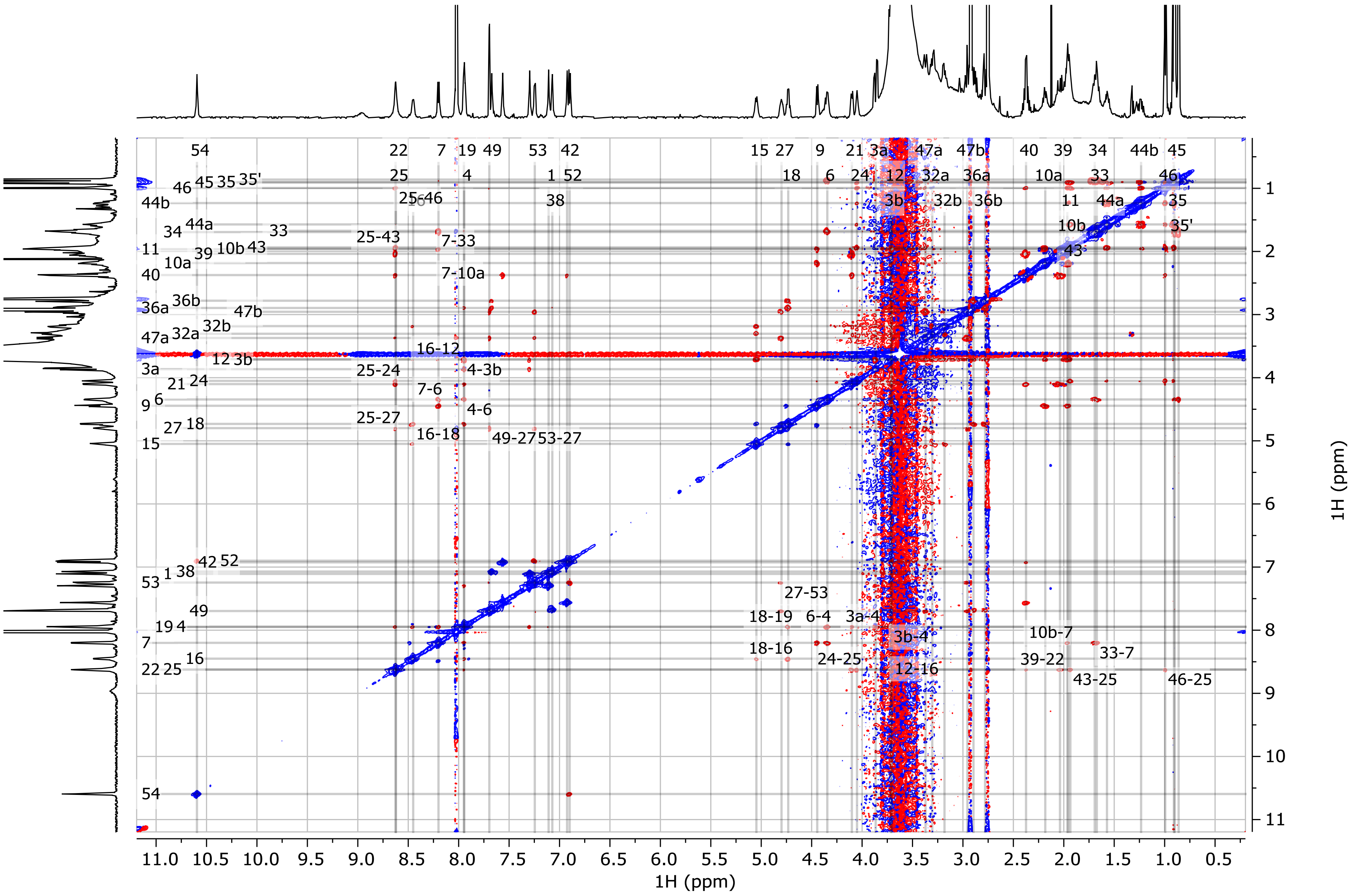

MS AND MS/MS DATA

Oxytocin-selenonium conjugate 7

17.10.2023 08:04 p.7/13 *** Angegebene Mol.-Gewichte u. Massenzahlen basieren auf dem häufigsten Isotop der Elemente *** MassLib

characteristical ions

```
 655.2081 = [C61H74N12O12S2Se1]2+        b8 || y1   -
 598.6657 = [C55H63N11O11S2Se1]2+        b7 || y2  188.1394 = [C8H18N3O2]+
1099.2721 = [C50H55N10O10S2Se1]+         b6 || y3  285.1921 = [C13H25N4O3]+
 550.1391 = [C50H56N10O10S2Se1]2+
 996.2625 = [C47H50N9O9S1Se1]+           b5 || y4  388.2013 = [C16H30N5O4S1]+
 882.2188 = [C43H44N7O7S1Se1]+           b4 || y5  502.2445 = [C20H36N7O6S1]+
 754.1601 = [C38H36N5O5S1Se1]+           b3 || y6  630.3033 = [C25N44N9O8S1]+
 641.0764 = [C32H25N4O4S1Se1]+           b2 || y7  743.3875 = [C31H55N10O9S1]+
                                      -  b1 || y8   -

 651.1511 = [C35H31N4O4Se1]+
 510.0721 = [C28H20N3O2Se1]+
```

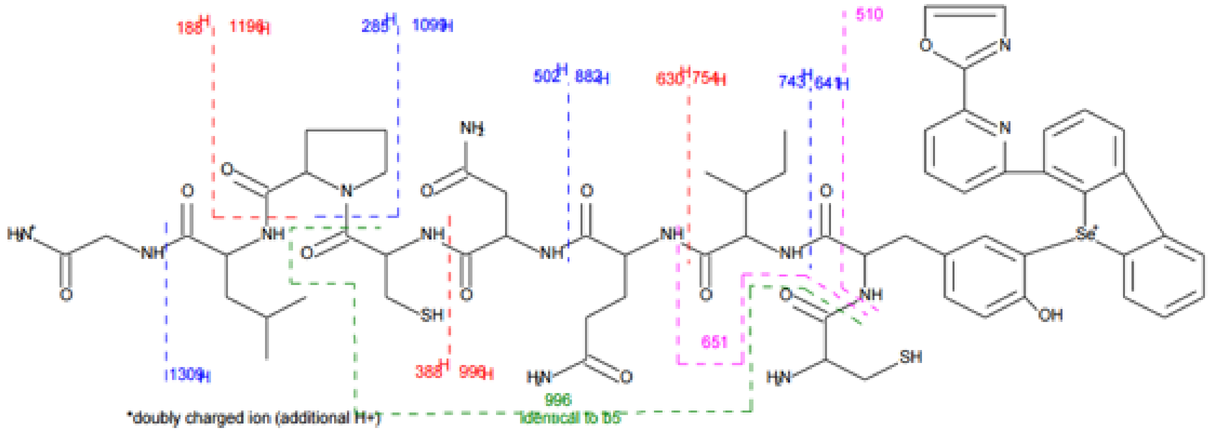

possible origin of observed fragments

17.10.2023 08:04 p.4/13 *** Angegebene Mol.-Gewichte u. Massenzahlen basieren auf dem häufigsten Isotop der Elemente *** **MassLib**

characteristical ions

1005 = [C43H65N12O12S2]+
1007 = [C43H67N12O12S2]+
988 = [C43H62N11O12S2]+
990 = [C43H64N11O12S2]+
971 = [C43H59N10O12S2]+
973 = [C43H61N10O12S2]+
931 = [C41H59N10O11S2]+
933 = [C41H61N10O11S2]+
914 = [C41H56N9O11S2]+
916 = [C41H58N9O11S2]+
899 = [C36H59N12O11S2]+
901 = [C36H61N12O11S2]+
882 = [C36H56N11O11S2]+
884 = [C36H58N12O11S2]+
721 = [C30H41N8O9S2]+
723 = [C30H43N8O9S2]+
712 = [C28H42N9O9S2]+
714 = [C28H44N9O9S2]+
704 = [C30H38N7O9S2]+
706 = [C30H40N7O9S2]+
693 = [C29H41N8O8S2]+
695 = [C29H43N8O8S2]+
676 = [C29H38N7O8S2]+
678 = [C29H40N8O8S2]+
615 = [C23H35N8O8S2]+
617 = [C23H37N8O8S2]+

743 = [C31H55N10O9S1]+
630 = [C25H44N9O8S1]+
502 = [C20H36N7O6S1]+
388 = [C16H30N5O4S1]+

376 = [C20H12N2O1Se1]+
285 = [C13H25N4O3]+
211 = [C11H19N2O2]+

MassLib V9.4 MPI für Kohlenforschung

17.10.2023 08:04 p.9/13 *** Angegebene Mol.-Gewichte u. Massenzahlen basieren auf dem häufigsten Isotop der Elemente *** MassLib

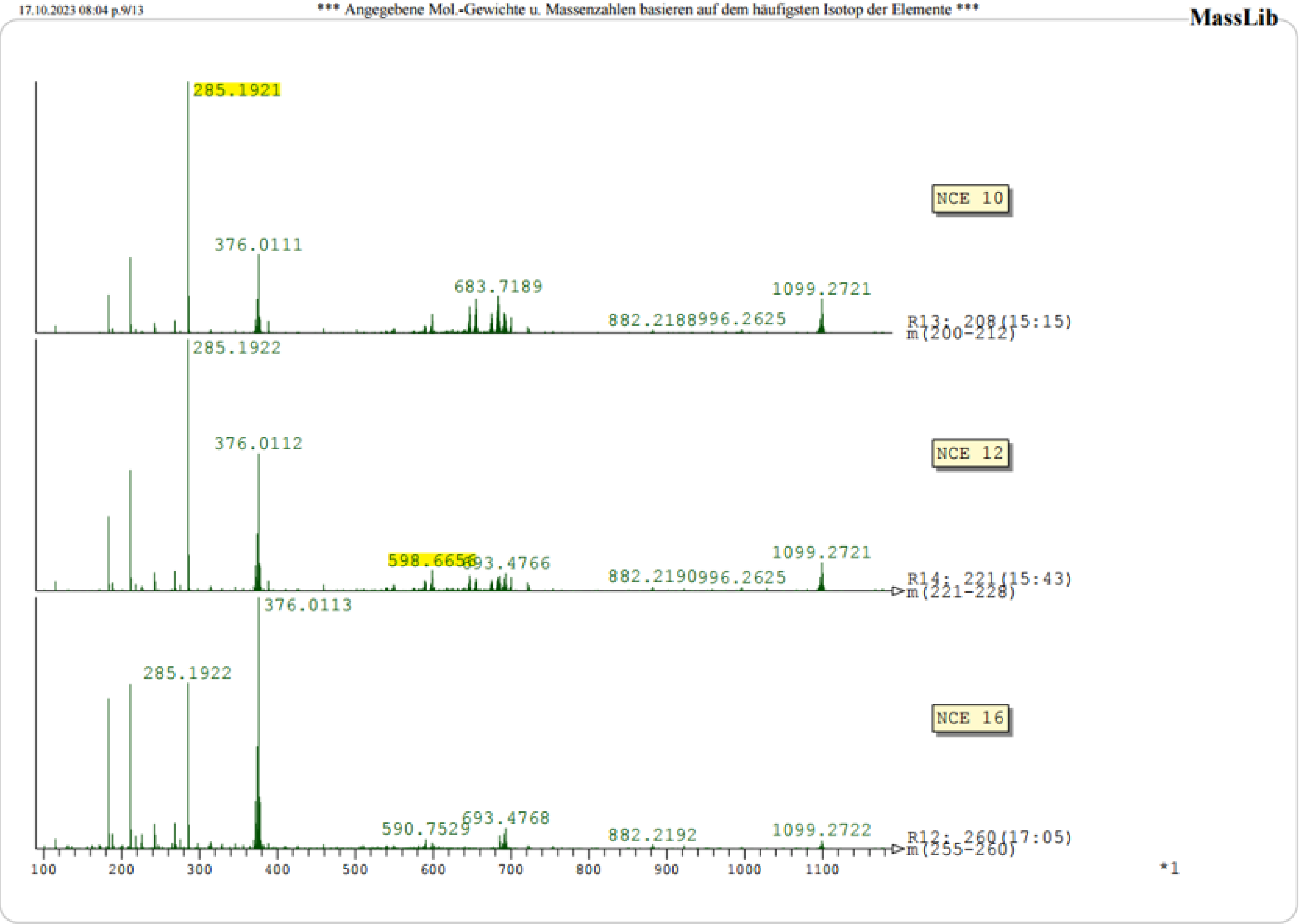

MassLib V9.4 MPI für Kohlenforschung

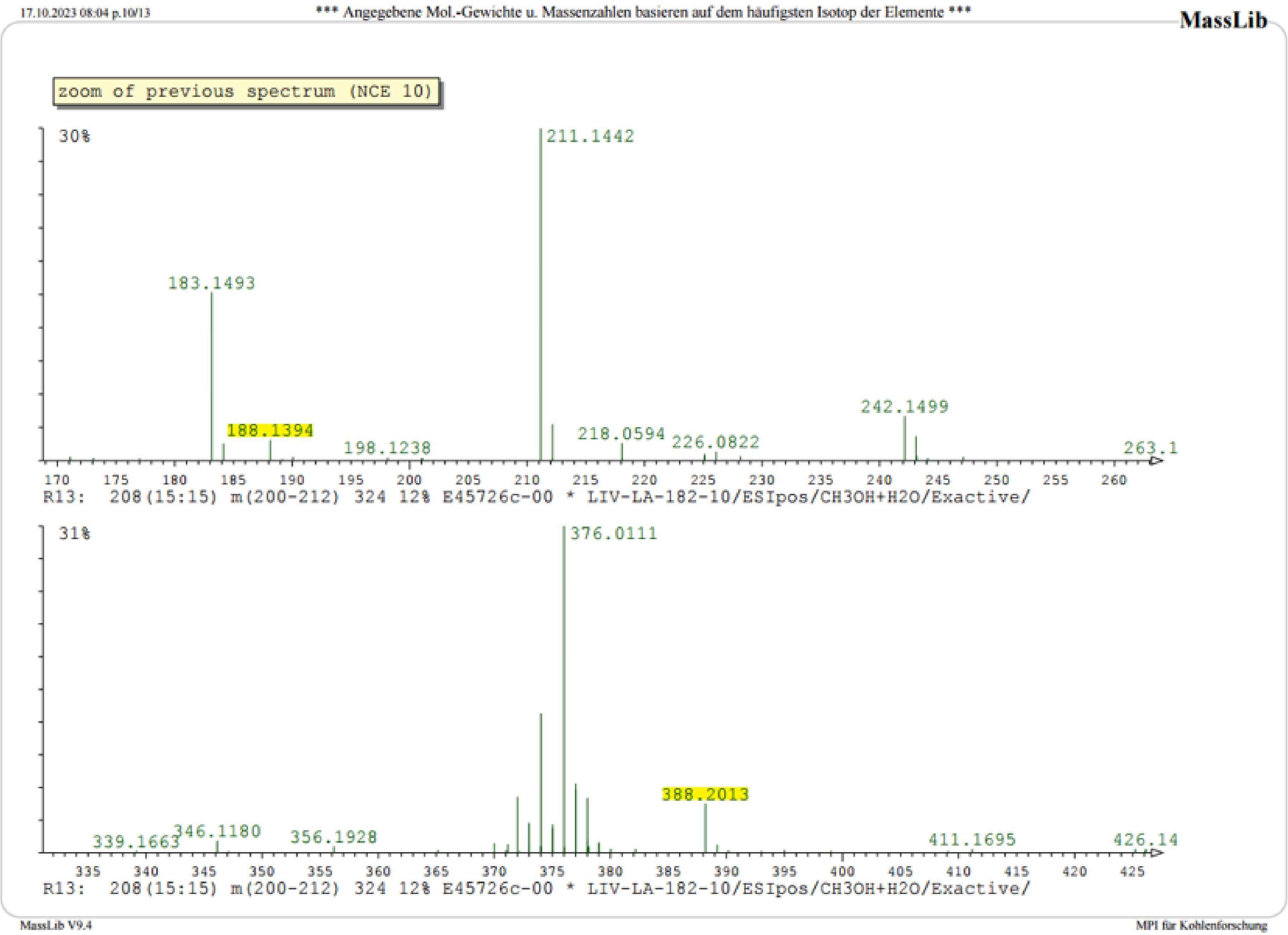
17.10.2023 08:04 p.10/13
*** Angegebene Mol.-Gewichte u. Massenzahlen basieren auf dem häufigsten Isotop der Elemente ***
MassLib
zoom of previous spectrum (NCE 10)
30%
211.1442
183.1493
188.1394
198.1238
218.0594
226.0822
242.1499
263.1
R13: 208(15:15) m(200-212) 324 12% E45726c-00 * LIV-LA-182-10/ESIpos/CH3OH+H2O/Exactive/
31%
376.0111
388.2013
339.1663
346.1180
356.1928
411.1695
426.14
R13: 208(15:15) m(200-212) 324 12% E45726c-00 * LIV-LA-182-10/ESIpos/CH3OH+H2O/Exactive/
MassLib V9.4
MPI für Kohlenforschung

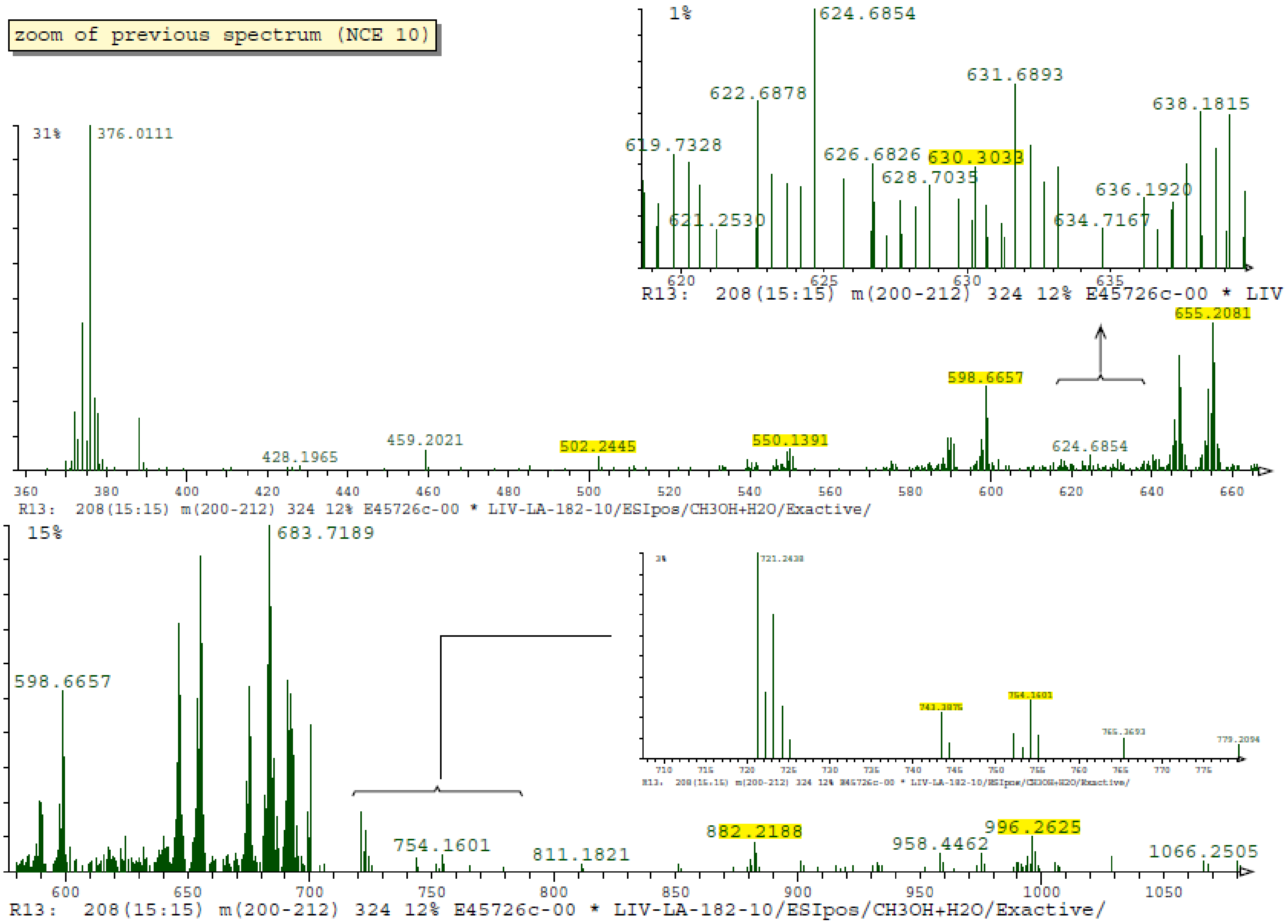
zoom of previous spectrum (NCE 10)
1%
624.6854
631.6893
622.6878
638.1815
619.7328
626.6826
630.3033
628.7035
636.1920
621.2530
634.7167
R13: 208(15:15) m(200-212) 324 12% E45726c-00 * LIV
655.2081
598.6657
31%
376.0111
459.2021
428.1965
502.2445
550.1391
624.6854
R13: 208(15:15) m(200-212) 324 12% E45726c-00 * LIV-LA-182-10/ESIpos/CH3OH+H2O/Exactive/
15%
683.7189
598.6657
754.1601
811.1821
882.2188
958.4462
996.2625
1066.2505
R13: 208(15:15) m(200-212) 324 12% E45726c-00 * LIV-LA-182-10/ESIpos/CH3OH+H2O/Exactive/

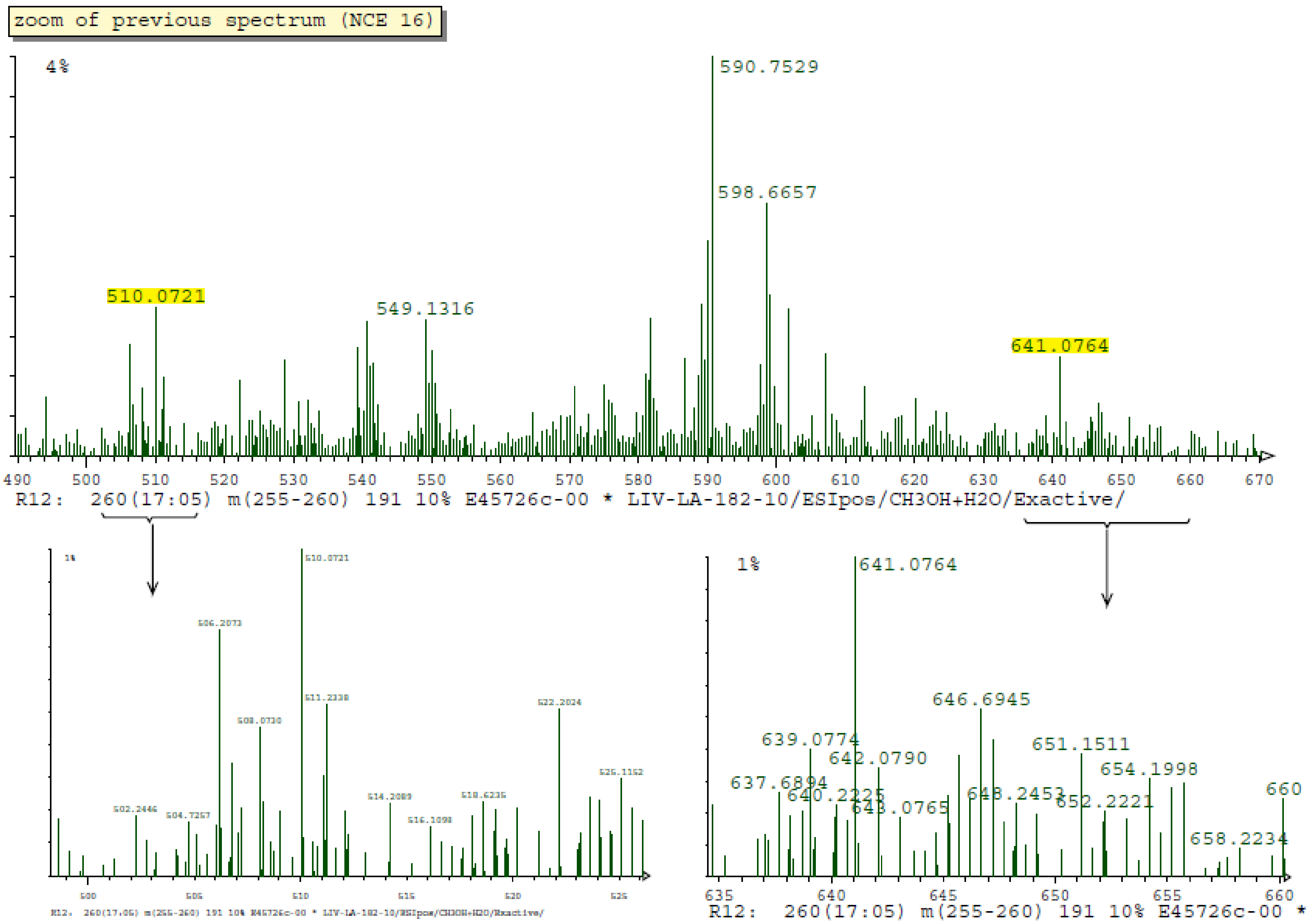
zoom of previous spectrum (NCE 16)
4%
590.7529
598.6657
510.0721
549.1316
641.0764
490 500 510 520 530 540 550 560 570 580 590 600 610 620 630 640 650 660 670
R12: 260(17:05) m(255-260) 191 10% E45726c-00 * LIV-LA-182-10/ESIpos/CH3OH+H2O/Exactive/
1%
641.0764
646.6945
639.0774
651.1511
642.0790
654.1998
637.6894
640.2225
648.2453
660
643.0765
652.2221
658.2234
635 640 645 650 655 660
R12: 260(17:05) m(255-260) 191 10% E45726c-00 *

Bivalirudin-selenonium conjugate 8

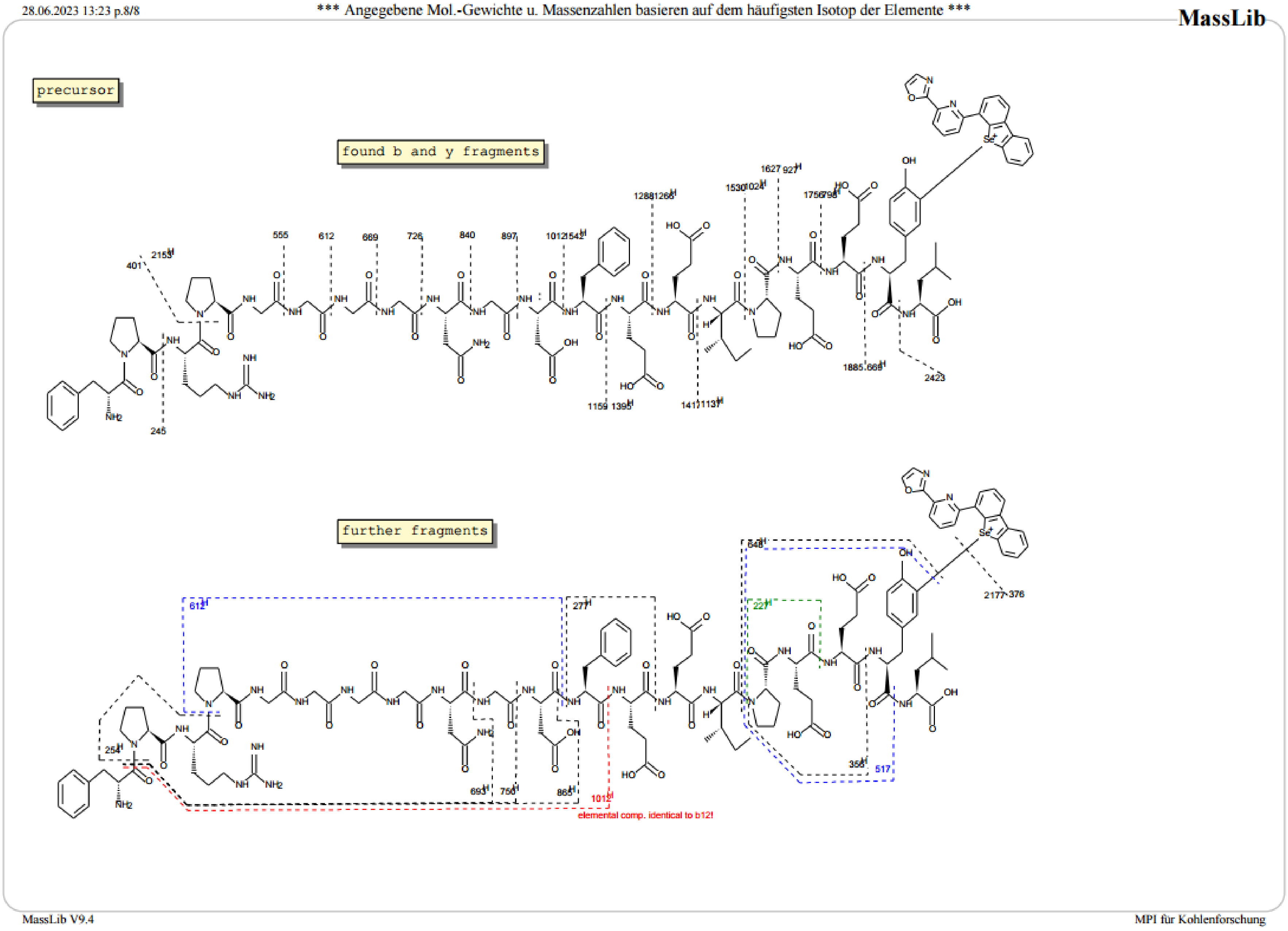

characteristical ions (spectra + zoom on the following pages)

245.1282 = [C14H17N2O2]+	b2	|	-	
401.2293 = [C20H29N6O3]+	b3	|	y17	1077.3870 = [C98H122N20O31Se1]+ (2153 + H+ additional charge carrier)
555.3040 = [C27H39N8O5]+	b5	|	-	
612.3256 = [C29H42N9O6]+ (low intensity)	b6	|	-	
669.3476 = [C31H45N10O7]+	b7	|	-	
726.3688 = [C33H48N11O8]+	b8	|	-	
840.4113 = [C37H54N13O10]+	b9	|	-	
869.4385 = [C38H57N14O10]+<br>897.4337 = [C39H57N14O11]+<br>914.4603 = [C39H60N15O11]+<br>984.4661 = [C42H62N15O13]+<br>995.4346 = [C43H59N14O14]+	b10	|	-	
1012.4613 = [C43H62N15O14]+/506.7336 = [C43H63N15O14]2+	b11	|	y9	771.7709 = [C75H89N11O20Se1]2+ (1542 + H+)
1159.5292 = [C52H71N16O15]+ / 580.2674 = [C52H18N16O15]2+	b12	|	y8	1395.4677 = [C66H79N10O19Se1]+ (low intensity)
1288.5696 = [C57H78N17O18]+ / 644.7891 = [C57H79N17O18]2+	b13	|	y7	1266.4254 = [C61H72N9O16Se1]+
1417.6135 = [C62H85N18O21]+ / 709.3104 = [C62H86N18O21]2+<br>700.3047 = [C62H84N18O20]2+<br>751.8550 = [C67H97N19O21]2+<br>756.8472 = [C68H95N19O21]2+	b14	|	y6	1137.3842 = [C56H65N8O13Se1]+<br>/569.1948 = [C56H66N8O13Se1]2+
1530.6968 = [C68H96N19O22]+ / 765.8521 = [C68H97N19O22]2+	b15	|	y5	1024.3002 = [C50H54N7O12Se1]+<br>/512.6530 = [C50H55N7O12Se1]2+
814.3782 = [C73H104N20O23]2+ (1627 + H+)	b16	|	y4	927.2458 = [C45H47N6O11Se1]+
878.8990 = [C78H111N21O26]2+ (1756 + H+)	b17	|	y3	798.2031 = [C40H40N5O8Se1]+
943.4210 = [C83H118N22O29]2+ (1885 + H+)	b18	|	y2	669.1609 = [C35H33N4O5Se1]+
808.6371 = [C112H138N25O32Se1]3+	b19	|		

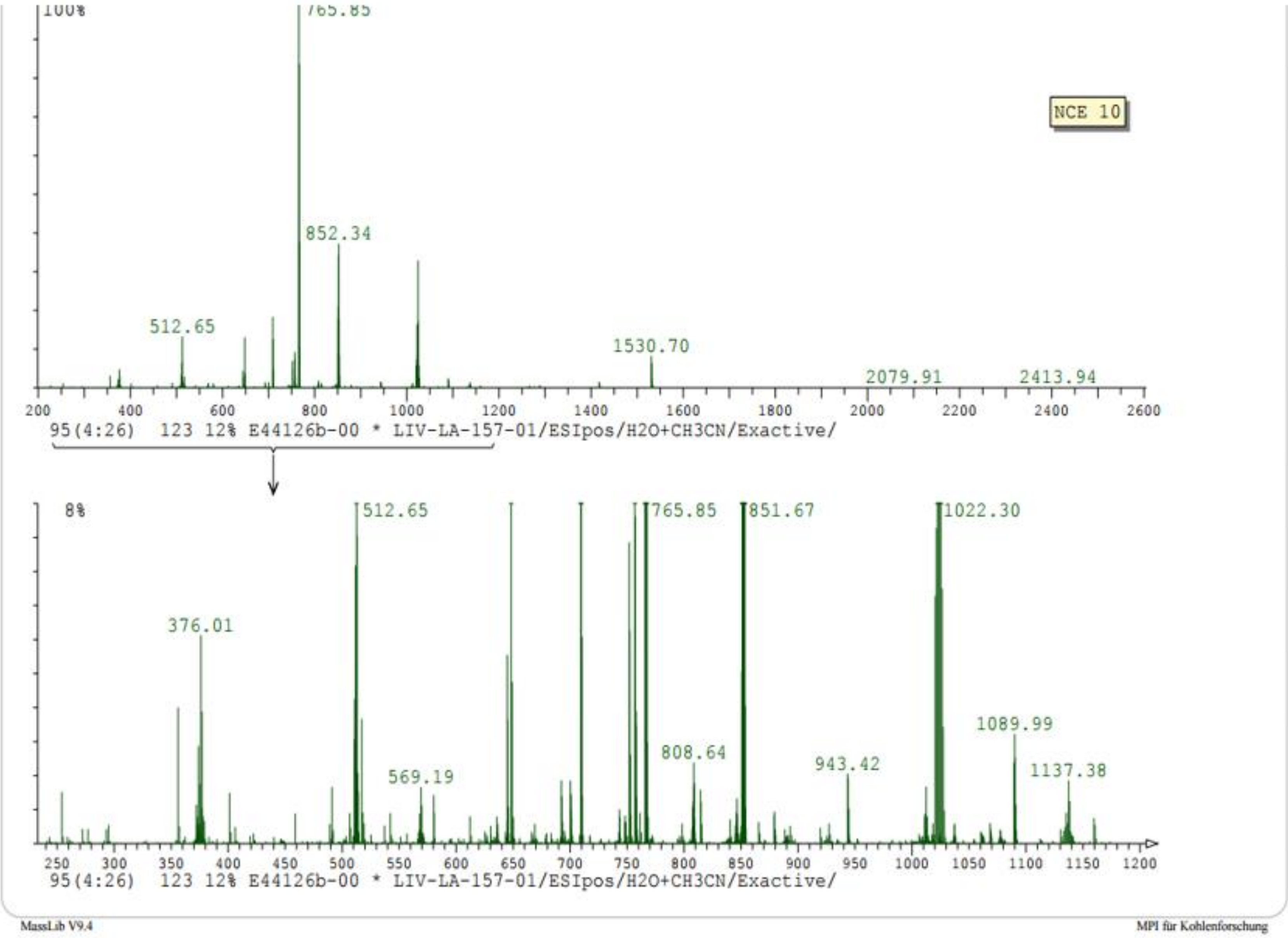
100%
765.85
NCE 10
852.34
512.65
1530.70
2079.91
2413.94
200 400 600 800 1000 1200 1400 1600 1800 2000 2200 2400 2600
95(4:26) 123 12% E44126b-00 * LIV-LA-157-01/ESIpos/H2O+CH3CN/Exactive/
8%
512.65
765.85
851.67
1022.30
376.01
1089.99
808.64
943.42
1137.38
569.19
250 300 350 400 450 500 550 600 650 700 750 800 850 900 950 1000 1050 1100 1150 1200
95(4:26) 123 12% E44126b-00 * LIV-LA-157-01/ESIpos/H2O+CH3CN/Exactive/
MassLib V9.4
MPI für Kohlenforschung

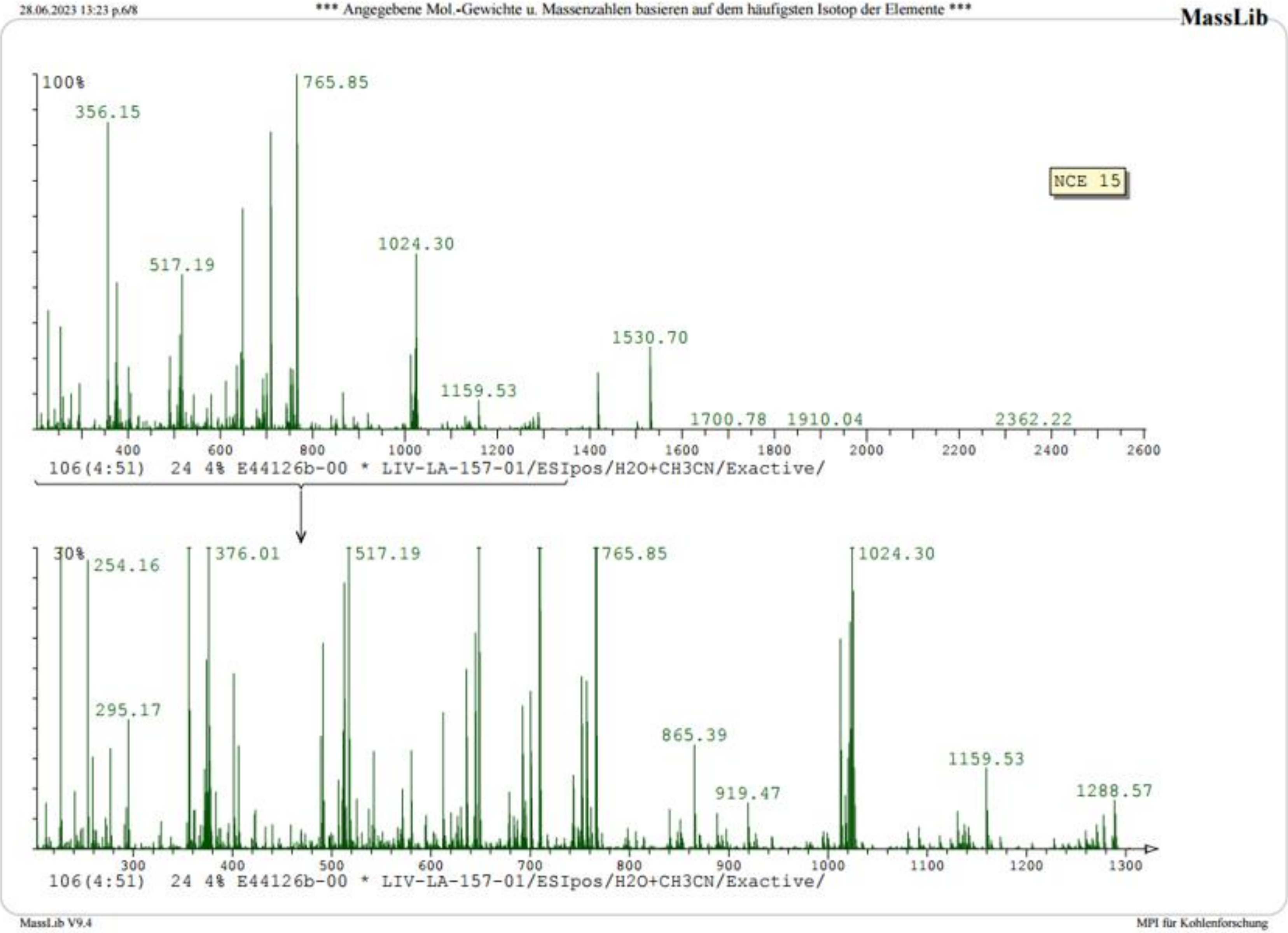
28.06.2023 13:23 p.6/8
*** Angegebene Mol.-Gewichte u. Massenzahlen basieren auf dem häufigsten Isotop der Elemente ***
MassLib
100%
765.85
356.15
NCE 15
1024.30
517.19
1530.70
1159.53
1700.78
1910.04
2362.22
400
600
800
1000
1200
1400
1600
1800
2000
2200
2400
2600
106(4:51) 24 4% E44126b-00 * LIV-LA-157-01/ESIpos/H2O+CH3CN/Exactive/
30%
254.16
376.01
517.19
765.85
1024.30
295.17
865.39
1159.53
919.47
1288.57
300
400
500
600
700
800
900
1000
1100
1200
1300
106(4:51) 24 4% E44126b-00 * LIV-LA-157-01/ESIpos/H2O+CH3CN/Exactive/
MassLib V9.4
MPI für Kohlenforschung

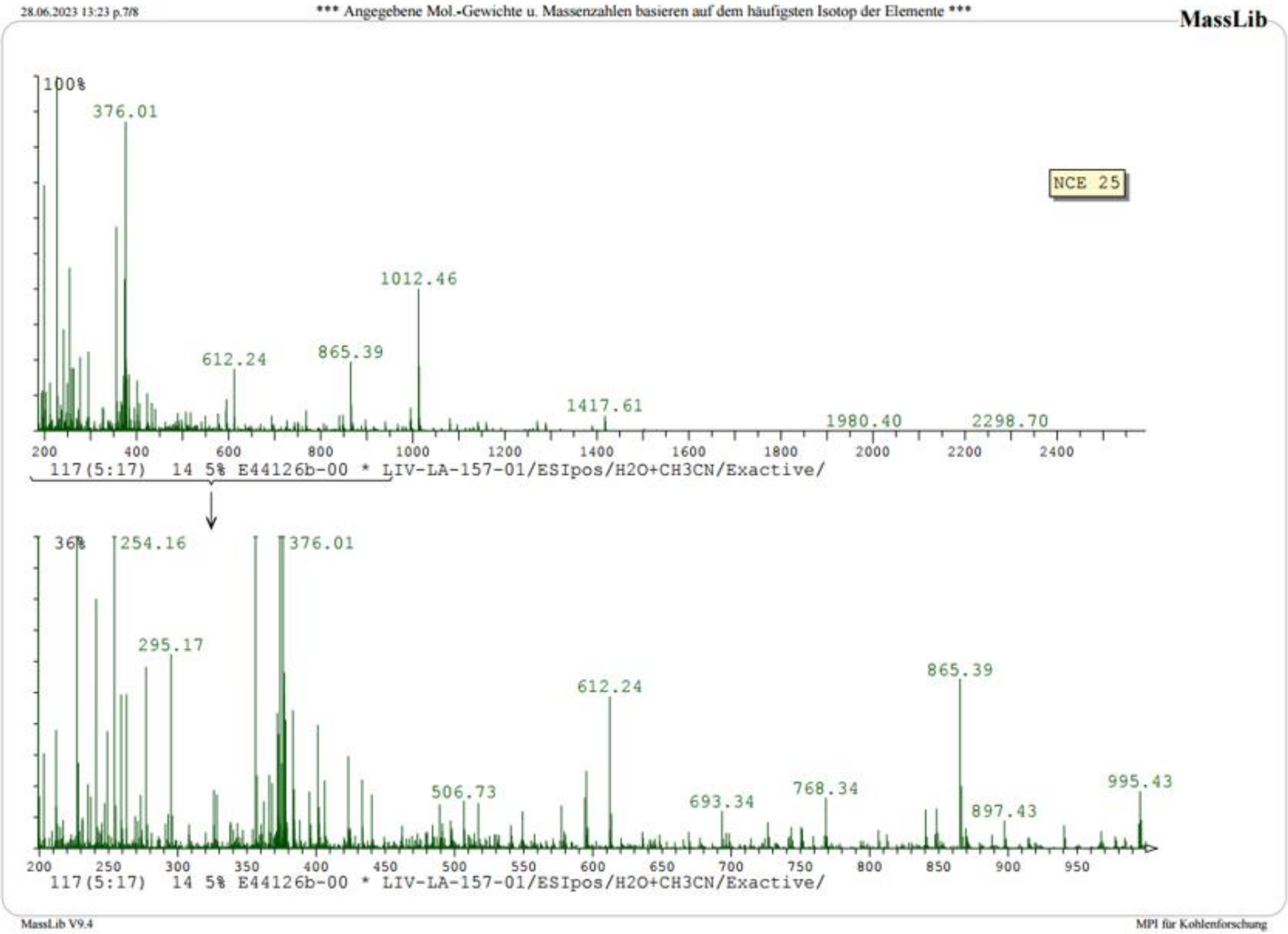
28.06.2023 13:23 p.7/8
*** Angegebene Mol.-Gewichte u. Massenzahlen basieren auf dem häufigsten Isotop der Elemente ***
MassLib
100%
376.01
NCE 25
1012.46
612.24
865.39
1417.61
1980.40
2298.70
117(5:17) 14 5% E44126b-00 * LIV-LA-157-01/ESIpos/H2O+CH3CN/Exactive/
36%
254.16
376.01
295.17
612.24
865.39
506.73
693.34
768.34
897.43
995.43
117(5:17) 14 5% E44126b-00 * LIV-LA-157-01/ESIpos/H2O+CH3CN/Exactive/
MassLib V9.4
MPI für Kohlenforschung

Angiotensin I-selenonium conjugate 9

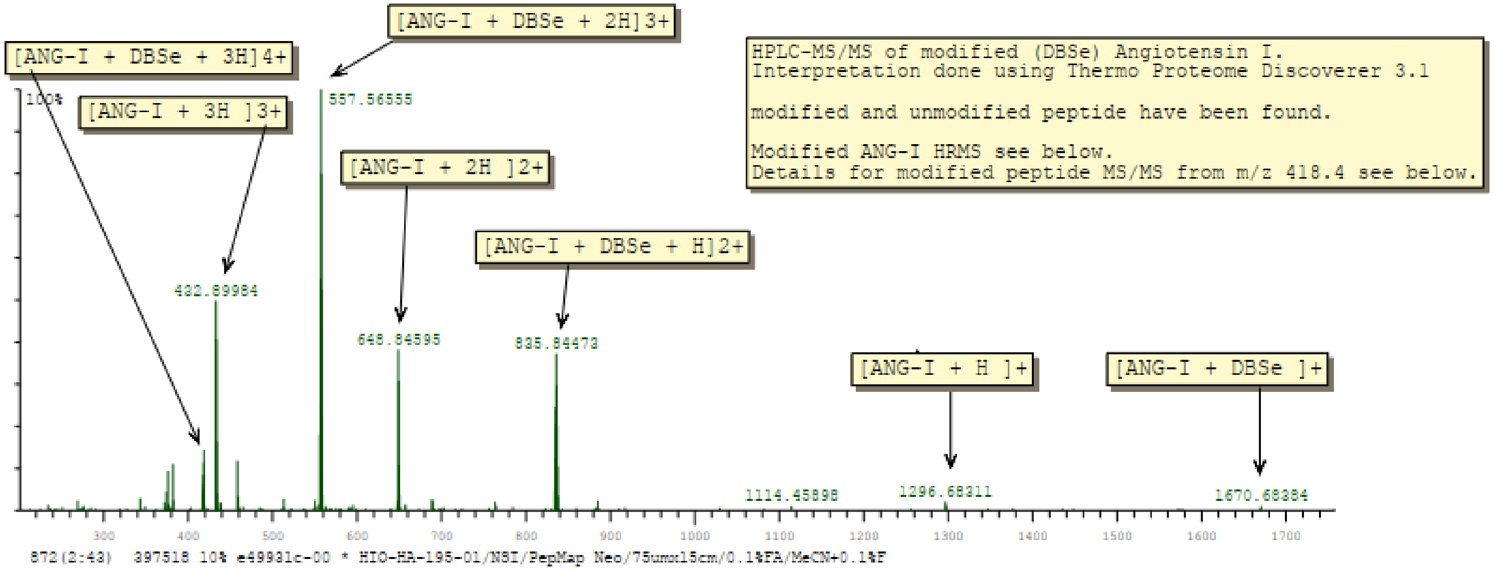

HRMS foor intact modified peptide.
Please note that theor. m/z below is the value for a fully resolved monoisotopic signal (signal with only the most abundant isotopes in the ion).
Due to small contributions of lesser isotopologue signals that cannot be resolved at finite instrument resolution the "achievable" theoretical value is shifted.
For the given mass resolution this value will be 557.56545,
reulting in an error of -0.18 ppm.

Mass to be matched (m/z): 557.56555 charge: 3

Mass tolerance: ±0.002
restriction of atom numbers:

C	H	N	O	Se
80-90	95-110	15-24	10-20	1-1

Number of calculated formulas: 8

Formula	Diff. (ppm)	theor. m/z	
C84 H104 N16 O16 Se1	-0.09	557.56550	
C83 H98 N23 O11 Se1	-0.09	557.56550	
C85 H100 N20 O12 Se1	0.70	557.56594	
C82 H102 N19 O15 Se1	-0.90	557.56505	<--
C87 H102 N17 O13 Se1	1.51	557.56639	
C81 H106 N15 O19 Se1	-1.69	557.56461	
C80 H100 N22 O14 Se1	-1.70	557.56460	
C90 H100 N18 O10 Se1	3.12	557.56729	

Characteristic ions (triply charged):
557.57 = [M + C20H11N2O1Se1 + 2H]3+ (M = Angiotensin I)

MS/MS ions found for m/z 418.4 ([M + DBSe + 3H]4+)
Given is always the most abundant ion in the pattern.

ion	theory	exp.	error [ppm]
(a3)+	343.20883	343.20901	-0.53
(a5)2+	497.17966	497.18127	-3.23
(a6)2+	565.70912	565.70905	+0.12
(a8)2+	687.76971	687.77032	-0.89
(a8)3+	458.84890	458.84933	-0.94
(a9)3+	504.53520	504.53622	-2.02
(b2)+	272.13533	272.13577	-1.61
(b5)2+	511.17712	511.17862	-2.93
(b6)2+	579.70658	579.70721	-1.09
(b8)2+	701.76717	701.76794	-1.10
(immonium 6/8)+	110.07127	110.07146	-1.69
(x5)2+	338.66372	338.66412	-1.17
(x6)2+	395.20576	395.20593	-0.44
(y1)+	132.10191	132.10213	-1.70
(y2)+	269.16082	269.16101	-0.72
(y3)+	416.22923	416.22937	-0.33
(y4)+	513.28199	513.28186	+0.26
(y4)2+	257.14464	257.14471	-0.29
(y5)2+	325.67409	325.67432	-0.70
(z5)2+	317.66473	317.66498	-0.79
(z6)2+	374.20676	374.20688	-0.32

ion numbering

a|b|c|imm.	Seq.	x|y|z
1	D	10
2	R	9
3	V	8
4	Y-DBSe	7
5	I	6
6	H	5
7	P	4
8	F	3
9	H	2
10	L	1

The modification site will be > 3
as by the presence of
- the (a3)+ ion

The modification site will be <= 5
as by the presence of
- the (a5)2+ ion (includes mod.)
- the (b2)2+ ion (includes mod.)

The modification site will be < 5
as by the presence of
- the (x6)2+ ion
- the (z6)2+ ion

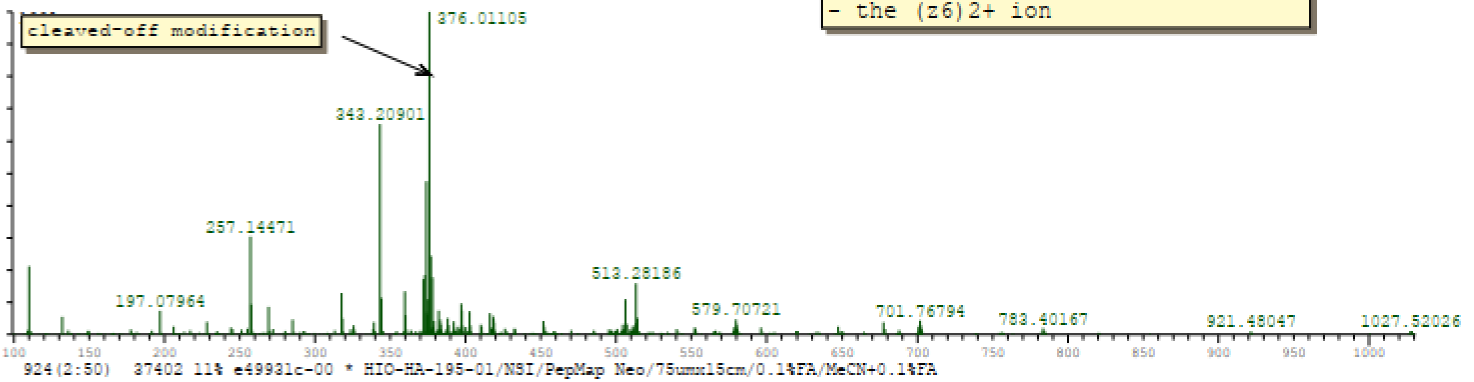

Pramlintide-selenonium conjugate 10

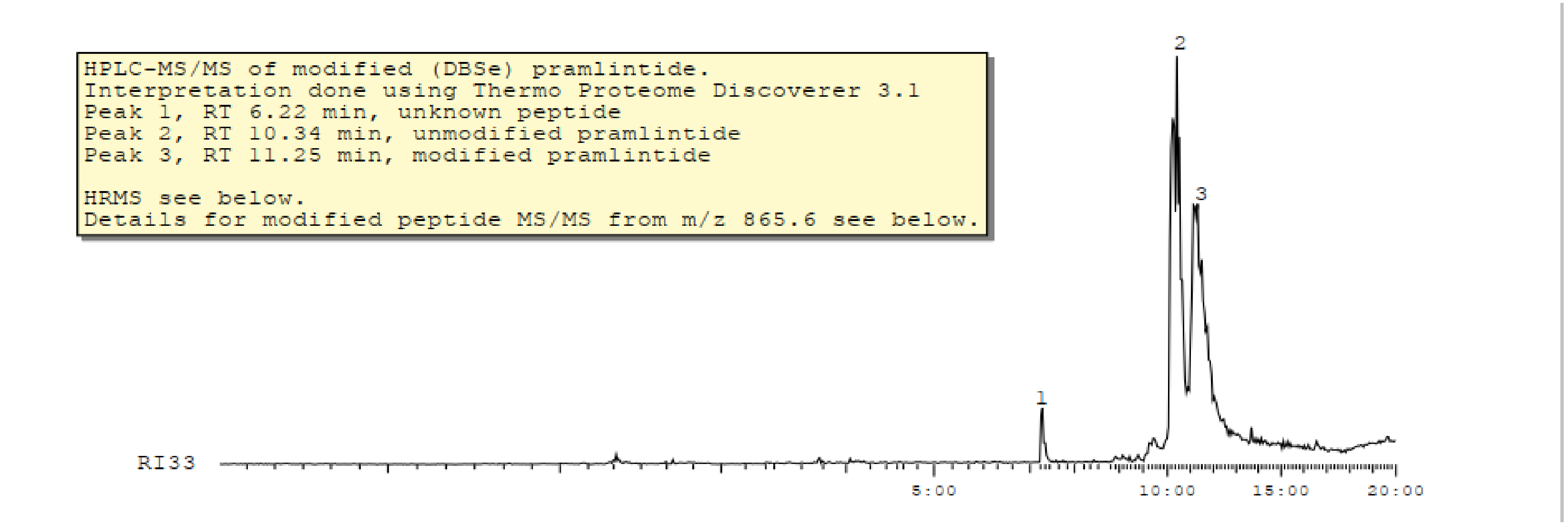

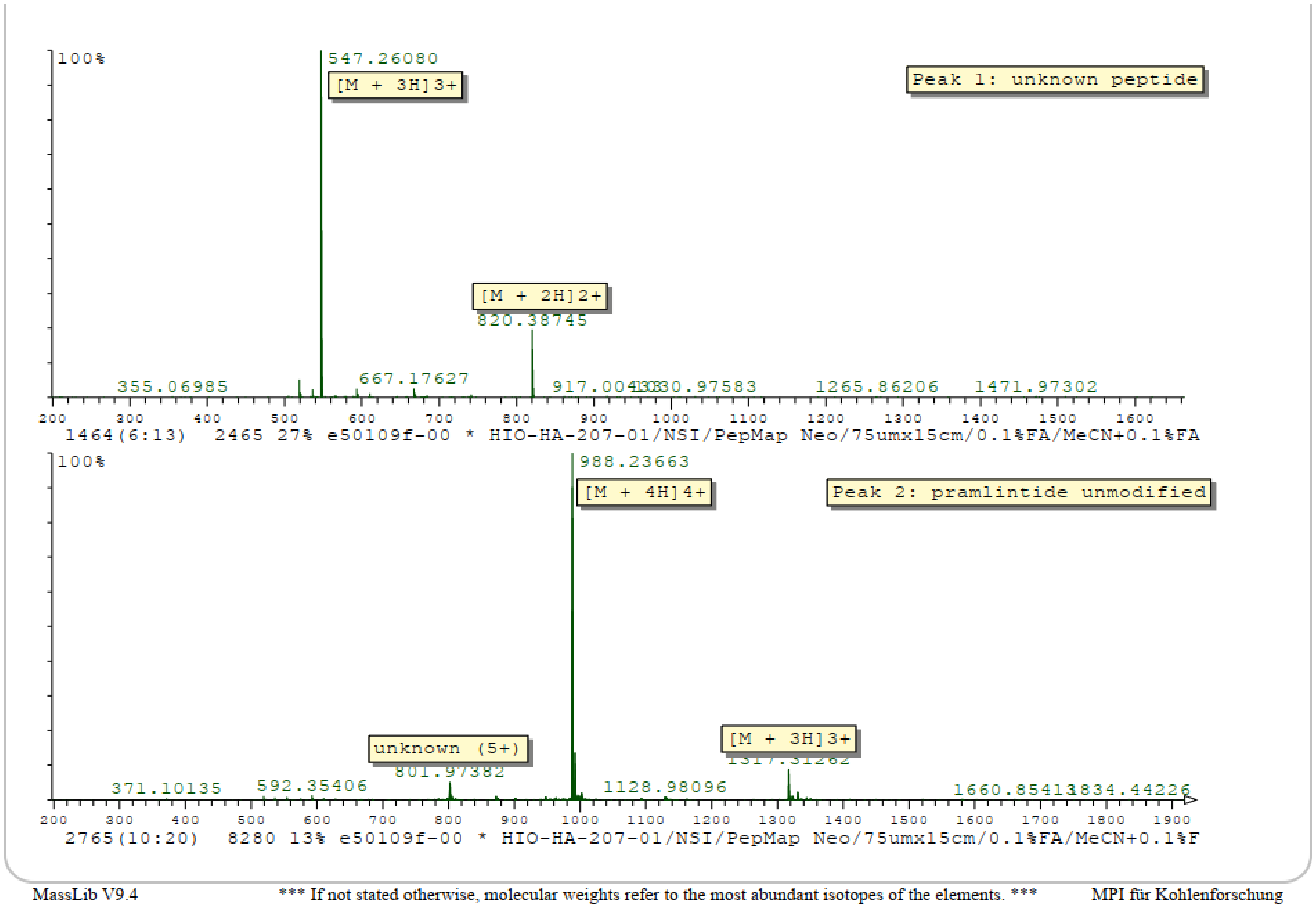
100%
547.26080
[M + 3H]3+
Peak 1: unknown peptide
[M + 2H]2+
820.38745
355.06985
667.17627
1265.86206
1471.97302
1464(6:13) 2465 27% e50109f-00 * HIO-HA-207-01/NSI/PepMap Neo/75umx15cm/0.1%FA/MeCN+0.1%FA
100%
988.23663
[M + 4H]4+
Peak 2: pramlintide unmodified
unknown (5+)
801.97382
[M + 3H]3+
371.10135
592.35406
1128.98096
2765(10:20) 8280 13% e50109f-00 * HIO-HA-207-01/NSI/PepMap Neo/75umx15cm/0.1%FA/MeCN+0.1%F
MassLib V9.4
*** If not stated otherwise, molecular weights refer to the most abundant isotopes of the elements. ***
MPI für Kohlenforschung

5.07.2024 13:15 p.275 *** If not stated otherwise, molecular weights refer to the most abundant isotopes of the elements. *** MassLib

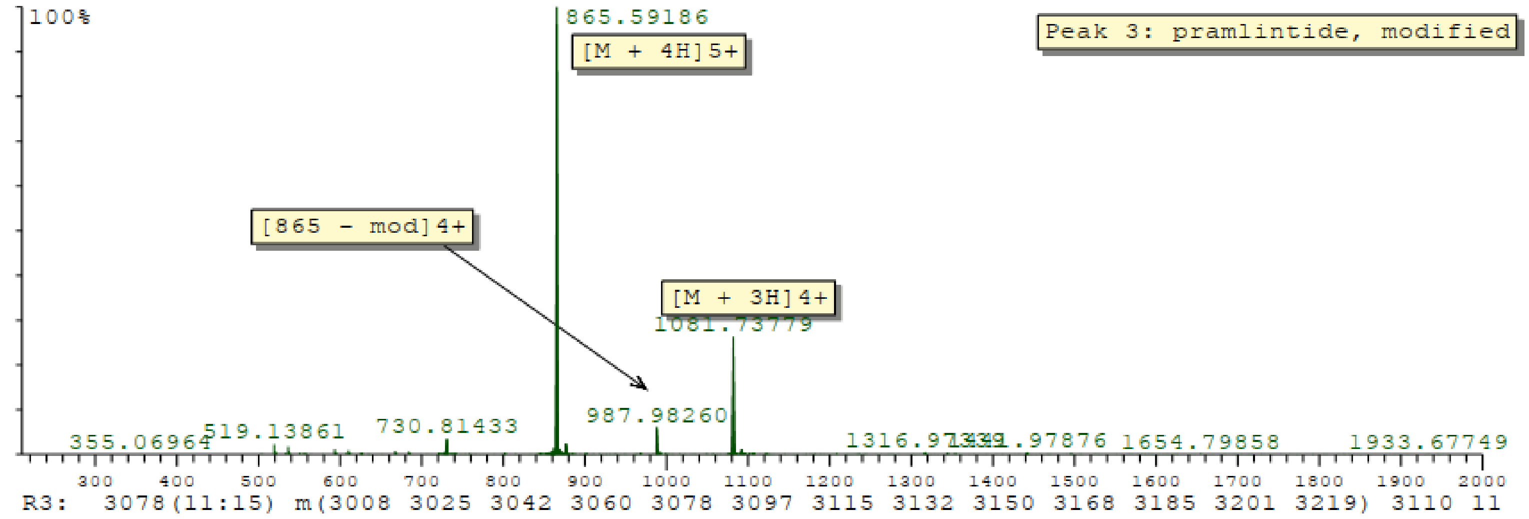

Mass to be matched (m/z): 865.19193 charge: 5

Mass tolerance: ±0.005
restriction of atom numbers:

C	H	N	O	S	Se
190-193	275-285	50-55	50-60	2-2	1-1

Number of calculated formulas: 8

Formula	Diff. (ppm)	theor. m/z	
C190 H282 N55 O53 S2 Se1	1.03	865.19282	
C193 H284 N50 O55 S2 Se1	-1.25	865.19085	
C192 H284 N52 O54 S2 Se1	1.34	865.19309	
C191 H282 N53 O54 S2 Se1	-1.56	865.19058	<-
C193 H278 N55 O51 S2 Se1	-3.85	865.18860	
C191 H284 N54 O53 S2 Se1	3.94	865.19534	
C192 H282 N51 O55 S2 Se1	-4.16	865.18833	
C190 H280 N54 O54 S2 Se1	-4.47	865.18806	

HRMS for intact modified peptide. Please note that theor. m/z in the table is the value for a fully resolved monoisotopic signal. Due to small contributions of lesser isotopologue signals that cannot be resolved at finite instrument resolution the "achievable" theoretical value is shifted. For the given mass resolution this value will be 865.19167, reulting in an error of -0.3 ppm.

Characteristic ions (fivefold charged):
865.19 = [M + C20H11N2O1Se1 + 4H]5+ (M = pramlintide)

MS/MS of [M + 4H]5+ ion cluster arounf m/z 865.6
Fragmetnation method: CID (30.0% collision energy)

ion	theory	exp.	Error [ppm]	
(b17)2+	917.45856	917.45764	+1.00	
(b18)2+	985.98801	985.98853	-0.53	
(b24)2+	1289.10791	1289.10608	+1.42	
(b26)2+	1394.17632	1394.17407	+1.61	
(b22)3+	791.71107	791.70929	+2.25	
(b23)3+	840.73387	840.73267	+1.43	
(b26)3+	929.78664	929.78607	+0.61	
(b27)3+	967.48133	967.48077	+0.58	
(b28)3+	999.83225	999.83148	+0.77	
(b30)3+	1065.86573	1065.86316	+2.41	
(b31)3+	1103.88004	1103.87891	+1.02	
(b24)4+	645.05759	645.05719	+0.62	
(b25)4+	669.32078	669.31897	+2.71	
(b26)4+	697.59180	697.59222	-0.60	
(b27)4+	725.86281	725.86206	+1.04	
(b28)4+	750.12600	750.12634	-0.45	
(b32)4+	852.92895	852.92865	+0.35	
(b36)4+	942.71498	942.71442	+0.59	
(y5)+	914.23708	914.23639	+0.76	
(y3)+	770.18359	770.18347	+0.16	
(y2)+	656.14066	656.14038	+0.43	(only traces)
(y10)2+	711.75445	711.75464	-0.26	

Desired modification site Y37 can be confirmed by presence of (y3)+ ion with modificaiton present (modification on N35, T36 or Y37) and (b36)4+ ion without modification (no modification from pos 1-36).

a|b|c|I	Seq.	x|y|z
1	K	37
2	C-|	36
3	N |	35
4	T |	34
5	A |	33
6	T |	32
7	C-|	31
8	A	30
9	T	29
10	Q	28
11	R	27
12	L	26
13	A	25
14	N	24
15	F	23
16	L	22
17	V	21
18	H	20
19	S	19
20	S	18
21	N	17
22	N	16
23	F	15
24	G	14
25	P	13
26	I	12
27	L	11
28	P	10
29	P	9
30	T	8
31	N	7
32	V	6
33	G	5
34	S	4
35	N	3
36	T	2
37	Y-Amidated-DBSe	1

Insulin-selenonium conjugate 11

HRMS

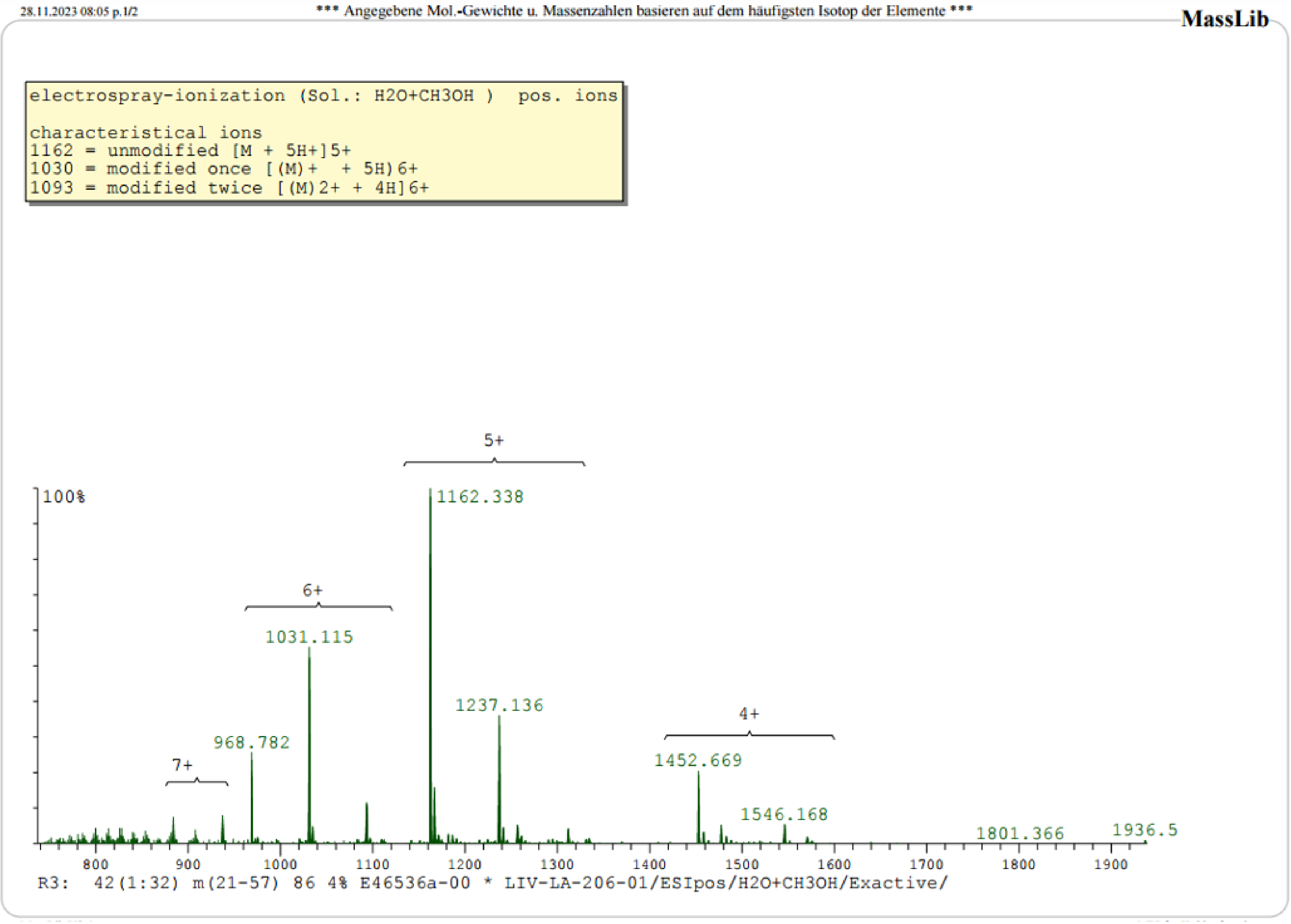

28.11.2023 08:05 p.2/2 *** Angegebene Mol.-Gewichte u. Massenzahlen basieren auf dem häufigsten Isotop der Elemente *** **MassLib**

No.	Monoisotopic Mass	Sum Intensity	Number of Charge States	Average Charge	Delta Mass	Rel. Abundance	Fractional Abundance
1	5803,6468	1150569,4	5	4,95	0	100	48,44

Charge State	Calculated Monoisotopic m/z	Monoisotopic Mass for This Charge	Mostabund m/z	Charge Normalized Intensity	Fit %
3	1935,5561	5803,6435	1936,5580	9770,05	95,6
4	1451,9189	5803,6381	1452,6689	196646,94	97,3
5	1161,7366	5803,6446	1162,3380	782735,22	96,7
6	968,2817	5803,6434	968,7825	160804,56	97,6
7	830,0996	5803,6415	830,5283	612,64	88,5

No.	Monoisotopic Mass	Sum Intensity	Number of Charge States	Average Charge	Delta Mass	Rel. Abundance	Fractional Abundance
2	5825,6273	169164,4	4	4,87	21,9805	14,70	7,12
3	6177,6470	744965,7	4	5,54	374,0002	64,75	31,36

Charge State	Calculated Monoisotopic m/z	Monoisotopic Mass for This Charge	Mostabund m/z	Charge Normalized Intensity	Fit %
4	1545,4185	6177,6360	1546,1682	51854,72	97,7
5	1236,5363	6177,6364	1237,1363	282933,92	97,3
6	1030,6148	6177,6410	1031,1155	369046,08	97,2
7	883,5280	6177,6399	883,9570	41130,98	97,4

No.	Monoisotopic Mass	Sum Intensity	Number of Charge States	Average Charge	Delta Mass	Rel. Abundance	Fractional Abundance
4	6199,6281	84992,67	5	5,48	395,98	7,39	3,58
5	6546,6466	39866,75	4	6,12	743,00	3,46	1,68
6	6550,6458	165596,45	5	6,07	747,00	14,39	6,97

Charge State	Calculated Monoisotopic m/z	Monoisotopic Mass for This Charge	Mostabund m/z	Charge Normalized Intensity	Fit %
4	1638,6671	6550,6352	1639,6684	3417,81	96,4
5	1311,1351	6550,6325	1311,9357	34225,04	96,1
6	1092,7805	6550,6389	1093,4487	78943,64	96,0
7	936,8129	6550,6364	937,3853	45717,35	96,8
8	819,8372	6550,6349	820,3379	3292,62	96,3

No.	Monoisotopic Mass	Sum Intensity	Number of Charge States	Average Charge	Delta Mass	Rel. Abundance	Fractional Abundance
7	6572,6282	20119,96	4	6,03	768,9814	1,75	0,85

MassLib V9.4 MPI für Kohlenforschung

LC/Q-TOF-MS and MS/MS for 11 after trypsin digestion and reduction

MS-TIC

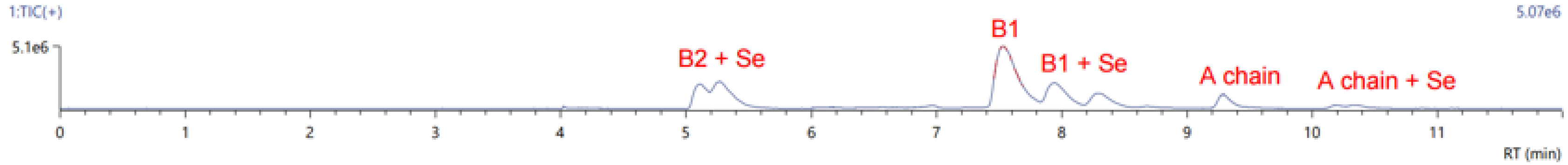

LC/Q-TOF-MS/MS (A chain + Se, both peaks)

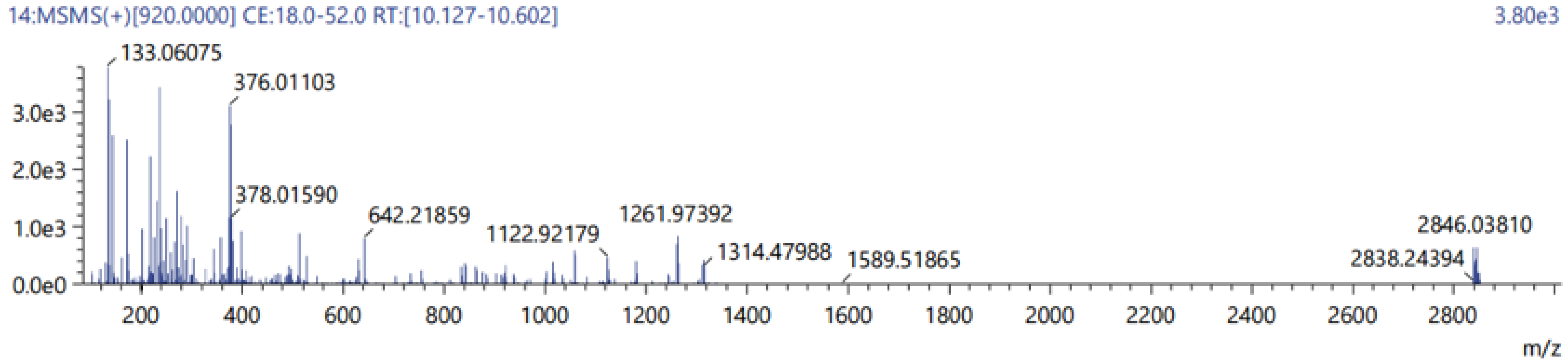

LC/Q-TOF-MS/MS (A chain + Se, former peak)

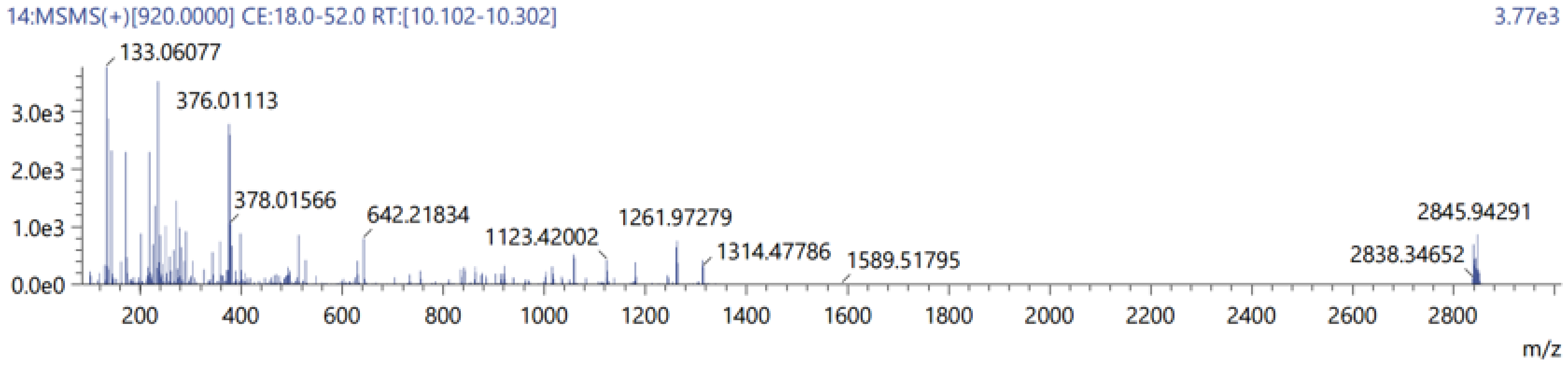

LC/Q-TOF-MS/MS (A chain + Se, latter peak)

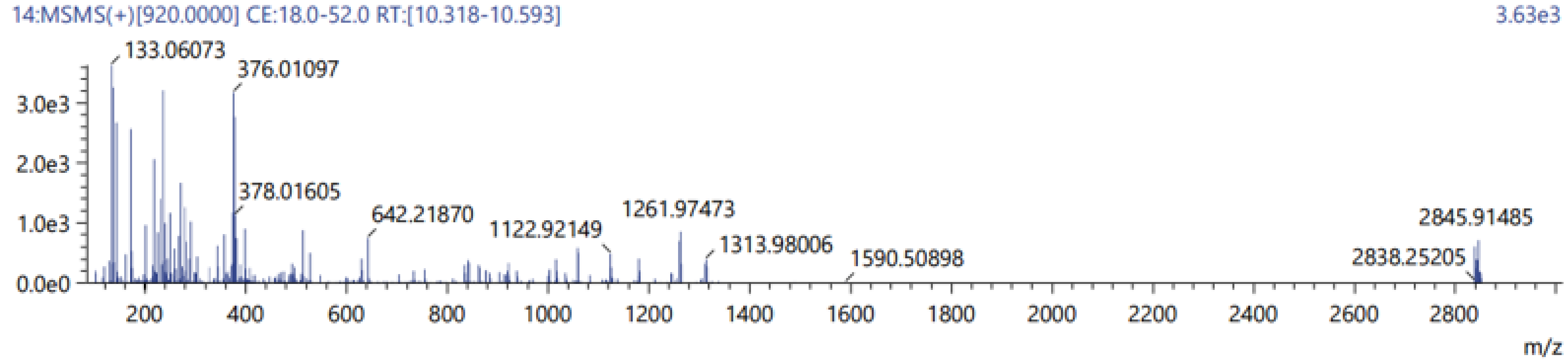

LC/Q-TOF-MS/MS (B2 chain + Se, both peaks)

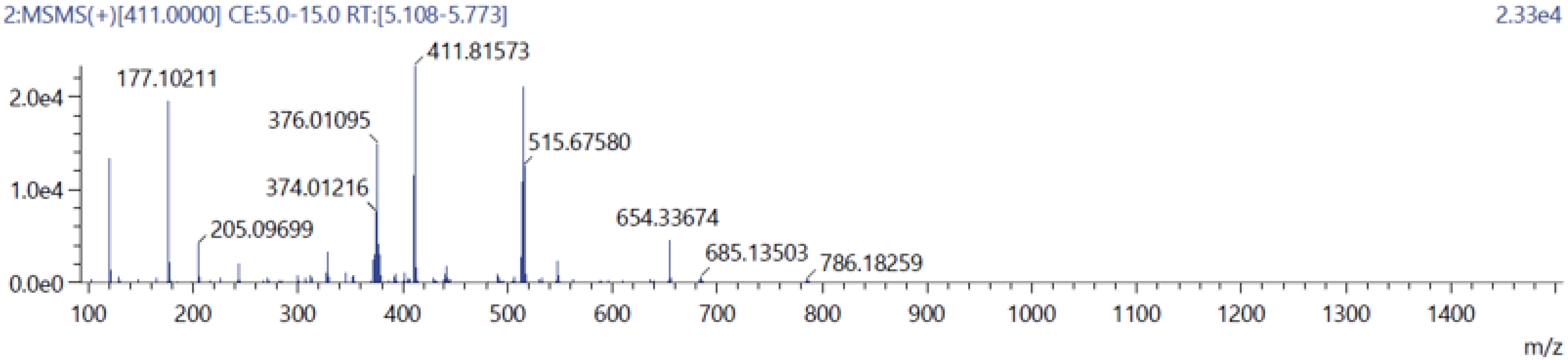

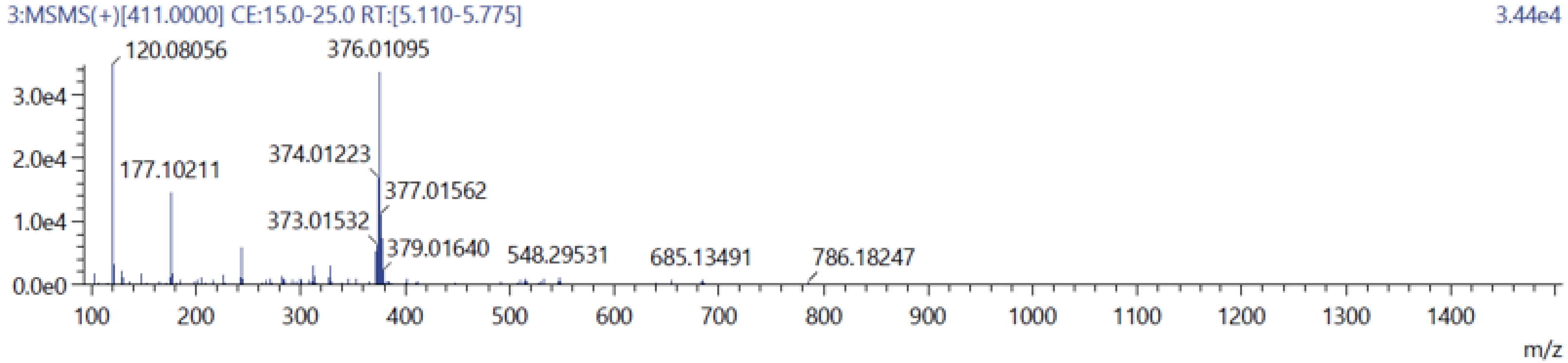

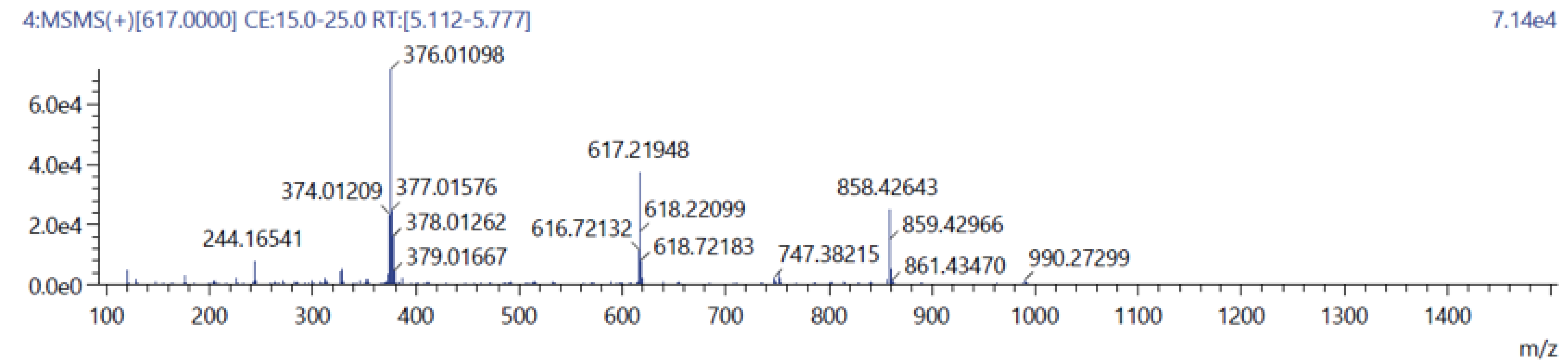
4:MSMS(+)[617.0000] CE:15.0-25.0 RT:[5.112-5.777]
7.14e4
376.01098
617.21948
374.01209
377.01576
858.42643
618.22099
378.01262
616.72132
859.42966
244.16541
618.72183
747.38215
379.01667
861.43470
990.27299
m/z

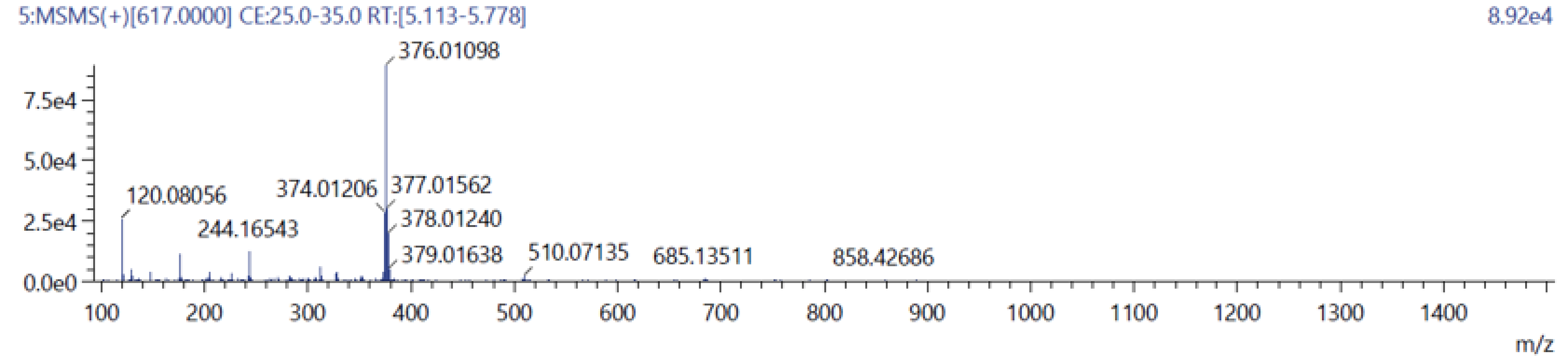
5:MSMS(+)[617.0000] CE:25.0-35.0 RT:[5.113-5.778]
8.92e4
376.01098
120.08056
374.01206
377.01562
244.16543
378.01240
379.01638
510.07135
685.13511
858.42686
m/z

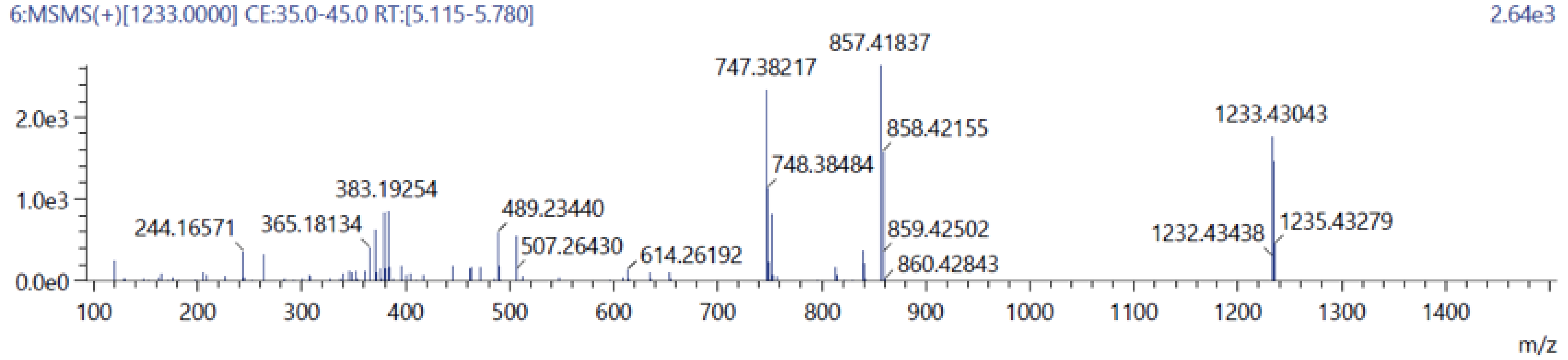
6:MSMS(+)[1233.0000] CE:35.0-45.0 RT:[5.115-5.780]
2.64e3
857.41837
747.38217
1233.43043
858.42155
748.38484
383.19254
489.23440
244.16571
365.18134
859.42502
1232.43438
1235.43279
507.26430
614.26192
860.42843
m/z

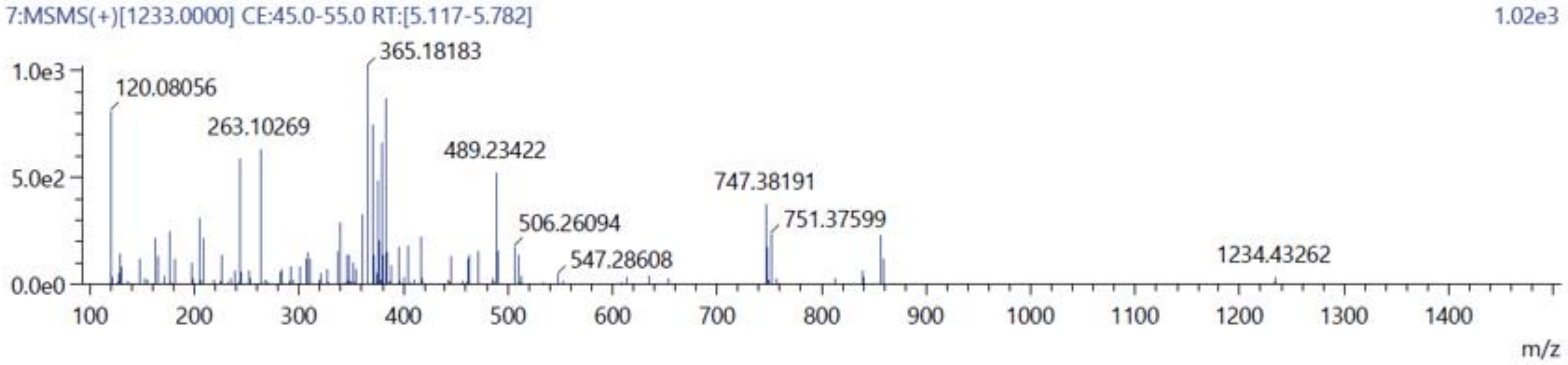
7:MSMS(+)[1233.0000] CE:45.0-55.0 RT:[5.117-5.782]
1.02e3
365.18183
120.08056
263.10269
489.23422
747.38191
751.37599
506.26094
547.28608
1234.43262
1.0e3
5.0e2
0.0e0
100
200
300
400
500
600
700
800
900
1000
1100
1200
1300
1400
m/z

Ubiquitin-selenonium conjugate 12

LC-FTMS of 12 after trypsin digestion

HPLC-MS/MS of modified bovine Ubiquitin, tryptic digest.
Interpretation done using Thermo Proteome Discoverer 3.1
A selenium containing modification has been found on
peptide [R].TLSDYNIQK.[E].
The modification is most likely located on tyrosine.
The modification location on Aspartic acid as found by
the software agorithm is very unlikely to be real.
Details see below

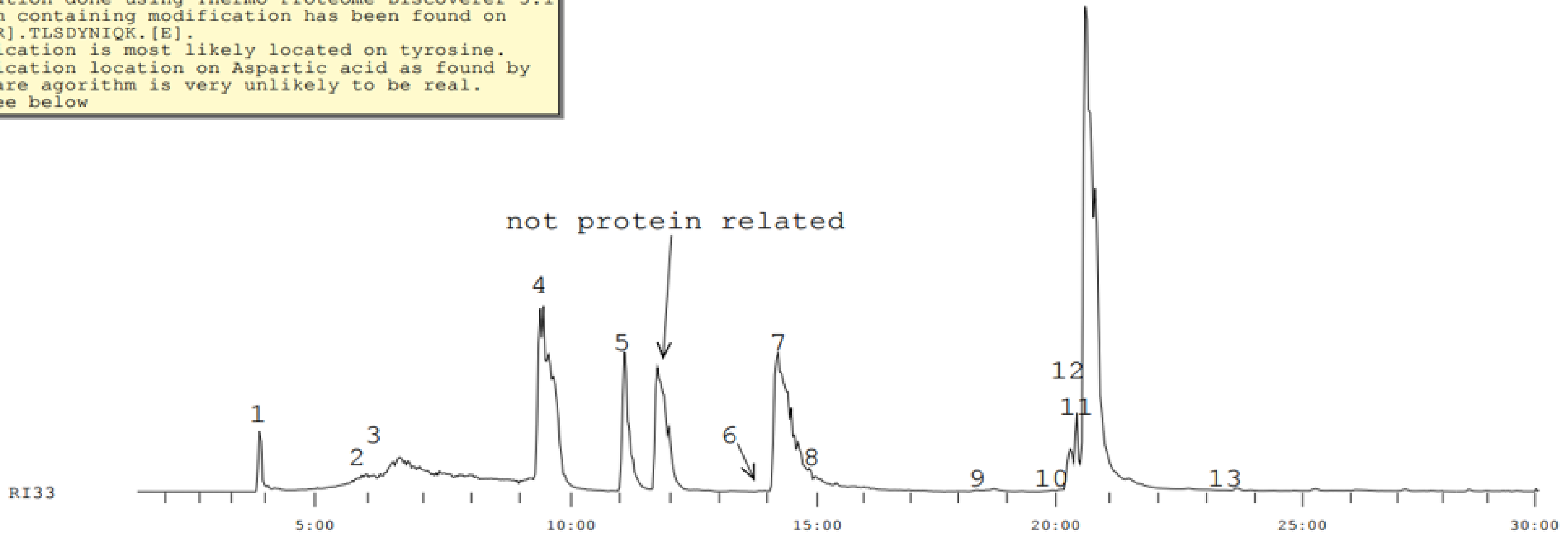

peptides identified

#	RT/min	Annotated Sequence	Mod.	Theor. m/z	Confidence (XCorr)
1	3.96	[K].QLEDGR.[T]		717.35259	1.50
2	5.74	[K].IQDKEGIPPDQQR.[L]		1523.78125	4.24
3	6.04	[K].EGIPPDQQR.[L]		1039.51670	2.20
4	9.50	[-].MQIFVK.[T]	1×Ox [M1]	781.42767	1.89
5	11.16	[R].TLSDYNIQK.[E]		1081.55242	1.98
6	14.03	[-].MQIFVK.[T]		765.43276	1.42
7	14.31	[K].ESTLHLVLR.[L]		1067.62077	2.66
8	15.84	[-].MQIFVK.[T]	1×Ac [N-Term]	807.44332	1.48
9	18.18	[K].TITLEVEPSDTIENVKAK.[I]		1987.05938	1.72
10	20.24	[K].TITLEVEPSDTIENVK.[A]		1787.92730	4.21
11	20.47	[R].TLSDYNIQK.[E]	1×DBSe [Y5]	1455.54825	1.63
12	20.47	[R].TLSDYNIQK.[E]	1×DBSe [D4]	1455.54825	1.59 <-- not probable
13	23.22	[R].TLSDYNIQKESTLHLVLR.[L]		2130.15534	2.40

MS/MS for the peptide 11

MS/MS ions found for #11 (mass error in ppm)

#1	b+	Immonium	Seq.	y+	#2
1			T		9
2	215.13895 (+0.32)		L		8
3	302.17065 (+1.31)		S	1241.41553 (+0.78)	7
4	417.19760 (+0.94)		D	1154.38867 (-3.63)	6
5		510.07150 (+0.04)	Y-DBSe		5
6	1068.30408 (-3.75)		N	502.29813 (+0.48)	4
7			I	388.25558 (-0.35)	3
8			Q	275.17120 (+0.66)	2
9			K		1

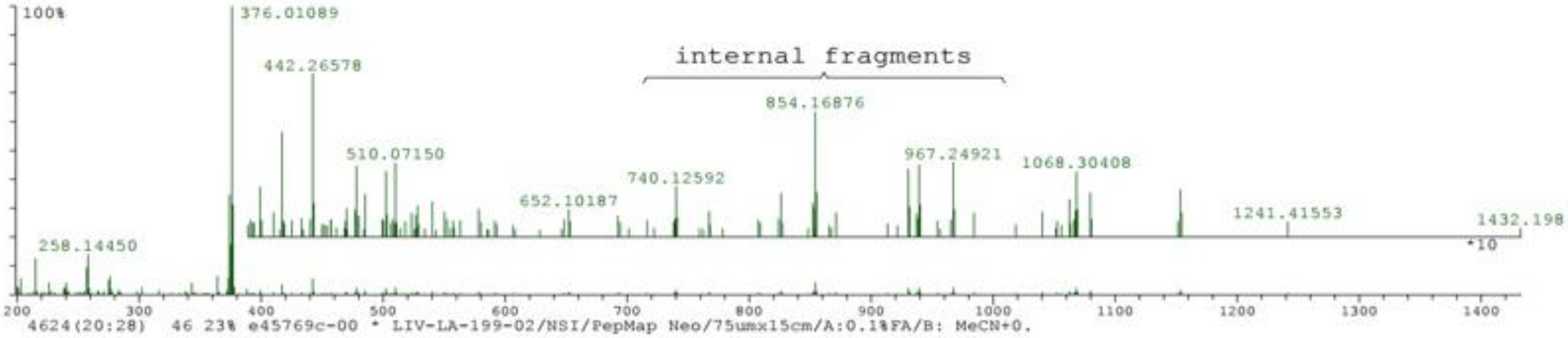

Note: MS signal 376 corresponds to the radical cation of selenide **S9**, which is related to the fragmentation of the selenonium group.

Ribonuclease A-selenonium conjugate 13

HRMS

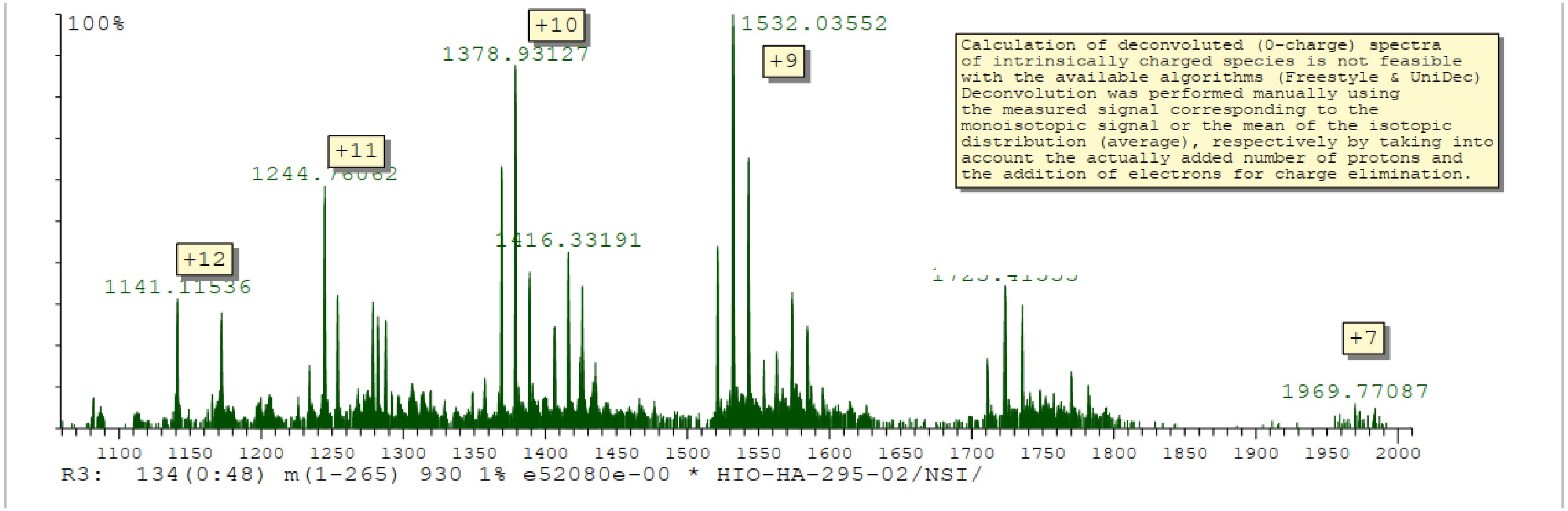

MS analysis of intact protein by direct injection MS.
unmodified:<br>Deconvoluted mass (0-charge):<br>Monoisotopic: 13673.25830 (Thermo Freestyle)<br>Average: 13682.30078 (Unidec)<br><br>Theoretical mass (Thermo Freestyle):<br>Monoisotopic: 13673.25937<br>Average: 13682.28196 (R=100)
modified with 1 x C20H11N2OSe<br>Deconvoluted mass (0-charge) calculated based on charge 9-12:<br>Monoisotopic: 14048.28694<br>Average: 14056.29055<br><br>Theoretical mass (Thermo Freestyle):<br>Monoisotopic: 14048.26303<br>Average: 14056.43044 (R=100)

LC-FTMS of 13 after trypsin digestion

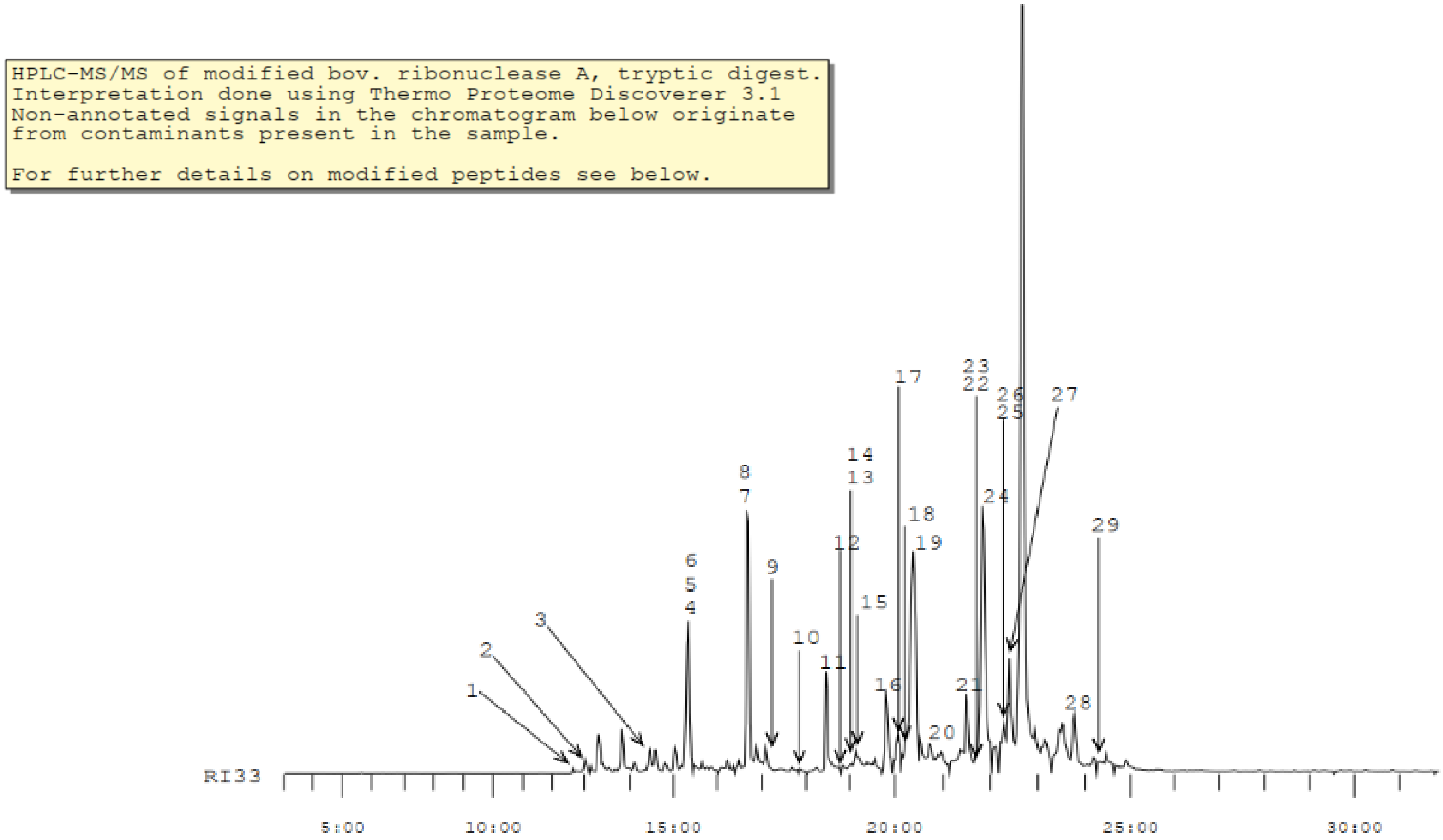

10.10.2024 11:57 p.2/26 *** If not stated otherwise, molecular weights refer to the most abundant isotopes of the elements. *** **MassLib**

#	RT/min	Sequence	Modifications	m/z	Abundance	XCorr	
1	12.69	[R].NLTKDR.[C]	-	373.71144	9.40E+07	1.75	
2	14.02	[R].QHMDSSTSAASSSNYCNQMMK.[S]	3×Oxidation [M3; M19; M20]	804.97680	1.70E+07	7.15	
			1×Carbamidomethyl [C16]				
3	14.58	[R].ETGSSKYPNCAYK.[T]	1×Carbamidomethyl [C10]	752.84049	1.50E+09	4.77	
4	15.32	[K].YPNCAYK.[T]	1×Carbamidomethyl [C4]	458.20476	1.37E+10	2.38	
5	15.33	[R].QHMDSSTSAASSSNYCNQMMK.[S]	1×Carbamidomethyl [C16]	799.64282	1.53E+08	9.42	
			2×Oxidation [M19; M]				
6	15.33	[R].QHMDSSTSAASSSNYCNQMMK.[S]	1×Oxidation [M]	794.31287	9.07E+08	9.26	
			1×Carbamidomethyl [C16]				
7	16.67	[R].QHMDSSTSAASSSNYCNQMMK.[S]	1×Gln->pyro-Glu [N-Term]	1182.45307	2.63E+08	5.76	
			1×Carbamidomethyl [C16]				
			1×Oxidation [M]				
8	16.67	[R].QHMDSSTSAASSSNYCNQMMK.[S]	1×Carbamidomethyl [C16]	788.98145	1.52E+10	9.15	
9	17.13	[R].QHMDSSTSAASSSNYCNQMMK.[S]	2×Oxidation [M19; M20]	780.63771	1.52E+07	4.41	
10	17.82	[K].NGQTNCYQSYSTMSITDCRETGSSKYPNCAYK.[T]	1×Carbamidomethyl [C6]	735.51256	1.91E+07	3.92	
			1×Oxidation [M13]				
11	18.45	[R].QHMDSSTSAASSSNYCNQMMK.[S]	1×Gln->pyro-Glu [N-Term]	783.30604	5.00E+09	8.78	
			1×Carbamidomethyl [C16]				
12	18.55	[K].NGQTNCYQSYSTMSITDCR.[E]	2×Carbamidomethyl [C6; C18]	767.97750	1.08E+08	6.56	
			1×Oxidation [M13]				
13	18.99	[K].NVACKNGQTNCYQSYSTMSITDCR.[E]	1×Acetyl [N-Term]	953.73120	1.12E+08	3.13	
			2×Carbamidomethyl [C11; C23]				
			1×Oxidation [M18]				
14	19.03	[R].ETGSSKYPNCAYK.[T]	1×DBSe [Y7]	470.42286	2.76E+08	2.93	<--
			1×Carbamidomethyl [C10]				
15	19.17	[R].QHMDSSTSAASSSNYCNQMMK.[S]	1×DBSe [Y15]	693.48543	4.06E+08	5.15	<--
			1×Carbamidomethyl [C16]				
			2×Oxidation [M19; M20]				
16	19.82	[K].NGQTNCYQSYSTMSITDCR.[E]	2×Carbamidomethyl [C6; C18]	762.64535	3.22E+09	7.36	
17	20.07	[R].QHMDSSTSAASSSNYCNQMMK.[S]	1×Oxidation [M3]	689.48623	3.31E+08	1.44	<--
			1×DBSe [Y15]				
			1×Carbamidomethyl [C16]				
18	20.23	[R].QHMDSSTSAASSSNYCNQMMK.[S]	1×DBSe [Y15]	685.48775	4.03E+08	4.23	<--
			1×Carbamidomethyl [C16]				
19	20.37	[R].CKPVNTFVHESLADVQAVCSQK.[N]	2×Carbamidomethyl [C1; C19]	839.74630	1.72E+10	8.04	
20	20.69	[K].YPNCAYK.[T]	1×DBSe [Y]	430.47147	8.15E+08	2.16	<--
			1×Carbamidomethyl [C4]				
21	21.51	[K].NGQTNCYQSYSTMSITDCR.[E]	1×Acetyl [N-Term]	1143.95542	4.30E+09	2.98	
			1×Oxidation [M13]				
			1×Carbamidomethyl [C18]				
22	21.65	[K].NGQTNCYQSYSTMSITDCR.[E]	2×Carbamidomethyl [C6; C18]	892.64256	1.06E+08	5.26	<--
			1×DBSe [Y]				
			1×Oxidation [M13]				

identified peptides (continued)

#	RT/min	Sequence	Modifications	m/z	Abundance	XCorr	
23	21.74	[R].QHMDSSTSAASSSNYCNQMMK.[S]	1×Gln->pyro-Glu [N-Term]	907.97043	4.15E+08	5.67	<--
			1×DBSe [Y15]				
			1×Carbamidomethyl [C16]				
24	21.83	[K].HIIVACEGNPYVPVHFDASV.[-]	1×Carbamidomethyl [C6]	742.03337	2.39E+10	7.11	
25	22.24	[R].CKPVNTFVHESLADVQAVCSQK.[N]	1×Carbamidomethyl [C1]	820.73820	6.21E+07	6.29	
26	22.28	[K].NGQTNCYQSYSTMSITDCR.[E]	2×Carbamidomethyl [C6; C18]	887.31133	1.25E+09	4.57	<--
			1×DBSe [Y]				
27	22.68	[K].HIIVACEGNPYVPVHFDASV.[-]	1×Carbamidomethyl [C6]	650.27617	9.18E+09	6.39	<--
			1×DBSe [Y11]				
28	23.76	[K].NGQTNCYQSYSTMSITDCR.[E]	1×Acetyl [N-Term]	887.63927	2.65E+09	1.64	<--
			1×DBSe [Y]				
			1×Oxidation [M13]				
			1×Carbamidomethyl [C18]				
29	24.29	[K].NGQTNCYQSYSTMSITDCR.[E]	2×Carbamidomethyl [C6; C18]	759.23135	3.53E+08	3.53	<--
			2×DBSe [Y7; Y10]				

For #22 site of modification is non-conclusive, spectra indicate a higher probability on Y10.
For #26 site of modification is non-conclusive, spectra indicate a higher probability on Y7.
For #28 site of modification is non-conclusive, spectra indicate a higher probability on Y7.

identification of #29 is only tentative, as HRMS of parent ion scan does not match well.
MS/MS is not very conclusive, but the compound definitely contains the Se-modification, probably twice.

MS/MS for the peptide 14

10.10.2024 11:57 p.3/26 *** If not stated otherwise, molecular weights refer to the most abundant isotopes of the elements. ***

MassLib

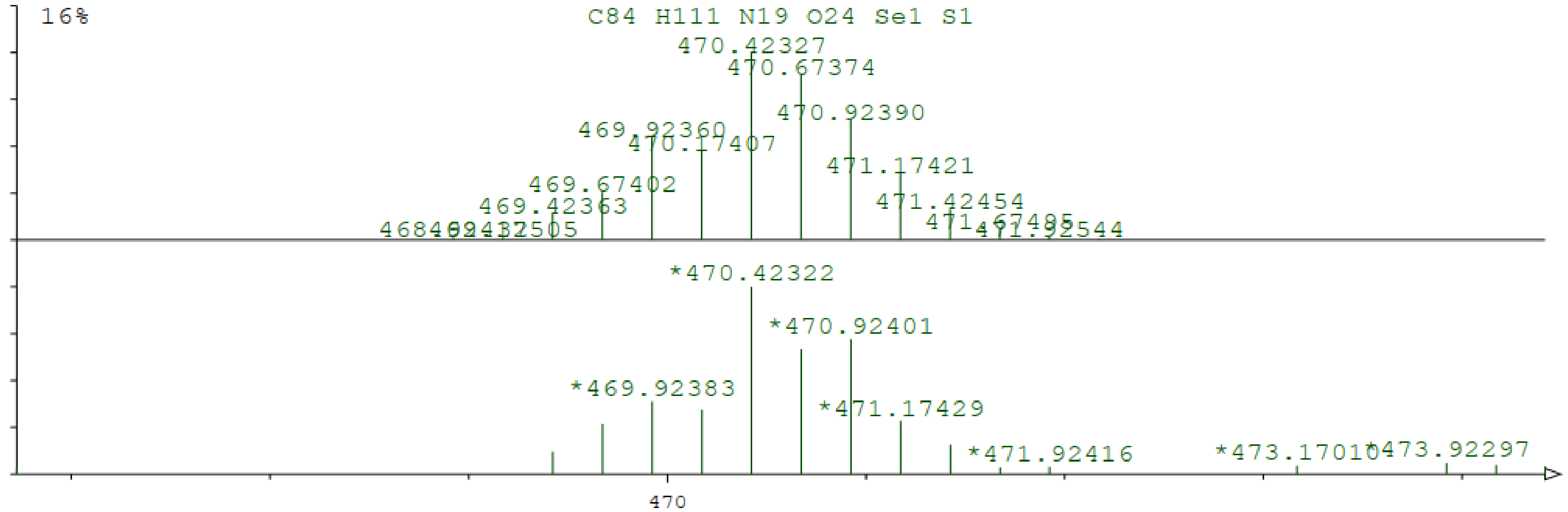

6382(19:11) 5404 8% e51789d-00 * HIO-HA-273-01/NSI/PepMap Neo/75umx50cm/0.1%FA/MeCN+0.1%FA

Mass to be matched (m/z): 470.423220 Charge: 4

Mass Tolerance: ±0.001000

Restriction of atom numbers:

C	H	N	O	Se	S
75-95	99-120	15-25	20-30	1-1	1-1

Number of calculated Formulas: 8

Formula	Diff. (ppm)	theor. m/z	
C86 H113 N16 O25 Se1 S1	-0.12	470.423166	
C85 H107 N23 O20 Se1 S1	-0.12	470.423164	
C87 H109 N20 O21 Se1 S1	0.59	470.423499	
C84 H111 N19 O24 Se1 S1	-0.83	470.422830	<--
C89 H111 N17 O22 Se1 S1	1.31	470.423836	
C83 H115 N15 O28 Se1 S1	-1.54	470.422496	
C82 H109 N22 O23 Se1 S1	-1.54	470.422494	
C75 H113 N22 O28 Se1 S1	1.58	470.423962	

HRMS for intact modified peptide #14.
Please note that theor. m/z in the table is the value for a fully resolved monoisotopic signal.
Due to small contributions of lesser isotopologue signals that cannot be resolved at finite instrument resolution the "achievable" theoretical value is shifted.
For the given mass resolution this value will be 470.42325, resulting in an error of +0.06 ppm.

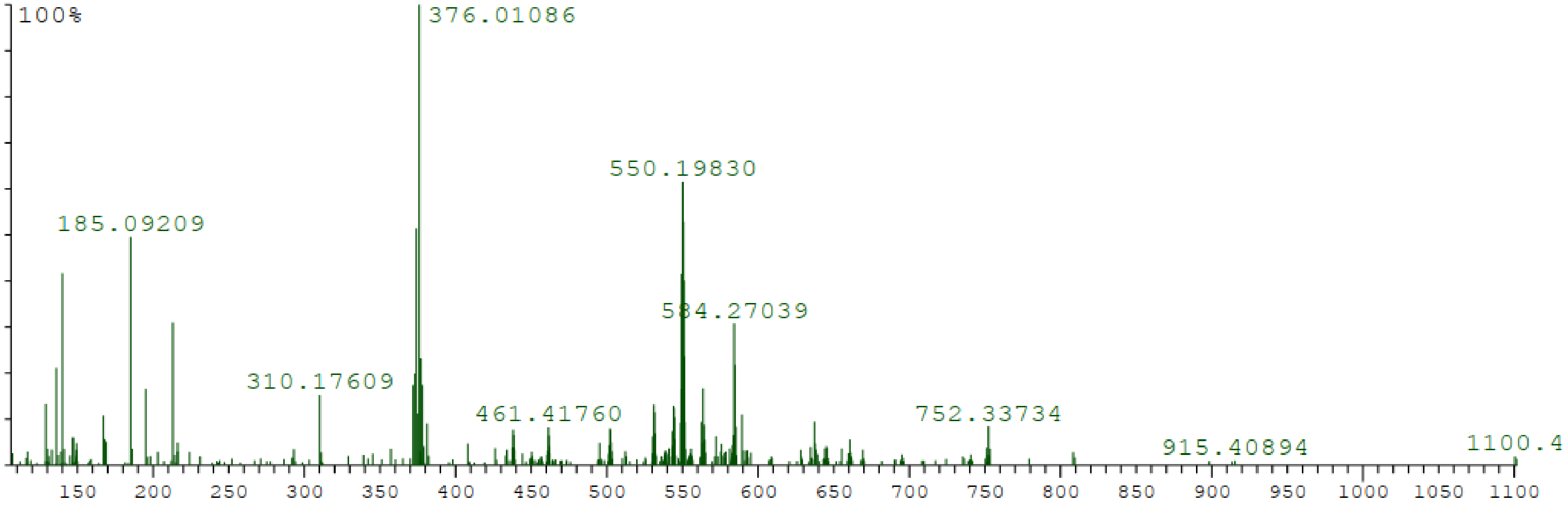

6396(19:13) 87 6% e51789d-00 * HIO-HA-273-01/NSI/PepMap Neo/75umx50cm/0.1%FA/MeCN+0.1%FA/0

ion	theory	exp.	Error [ppm]
(b2)+	231.09755	231.09781	-1.13
(b7)2+	564.17223	564.17279	-0.99
(b7)3+	376.45058	376.45026	+0.85
(I10)+	133.04301	133.04309	-0.60
(I12)+	136.07569	136.07579	-0.73
(y6)+	752.33959	752.33734	+2.99
(y5)+	655.28682	655.28619	+0.96
(y4)+	541.24389	541.24310	+1.47
(y3)+	381.21325	381.21310	+0.38
(y2)+	310.17613	310.17609	+0.14
(y1)+	147.11280	147.11290	-0.65
(y9)2+	752.76651	752.76379	+3.61
(y7)2+	645.20301	645.20392	-1.41
(y6)2+	376.67343	376.67361	-0.47
(y4)2+	271.12559	271.12616	-2.12
(y12)3+	583.88049	583.88080	-0.54
(y11)3+	550.19793	550.19830	-0.68
(y10)3+	531.19077	531.19122	-0.84
(y9)3+	502.18010	502.18002	+0.15
(y8)3+	473.16942	473.16931	+0.23
(y12)4+	438.16218	438.16251	-0.74

MS/MS of peptide #14 ([M + 3H]4+, m/z 470.42)
Fragmetnation: HCD(20.0%)

a|b|c|I	Seq.	x|y|z
1	E	13
2	T	12
3	G	11
4	S	10
5	S	9
6	K	8
7	Y-DBSe	7
8	P	6
9	N	5
10	C-Carbamidomethyl	4
11	A	3
12	Y	2
13	K	1

MS/MS for the peptide 15

10.10.2024 11:57 p.4/26 *** If not stated otherwise, molecular weights refer to the most abundant isotopes of the elements. *** **MassLib**

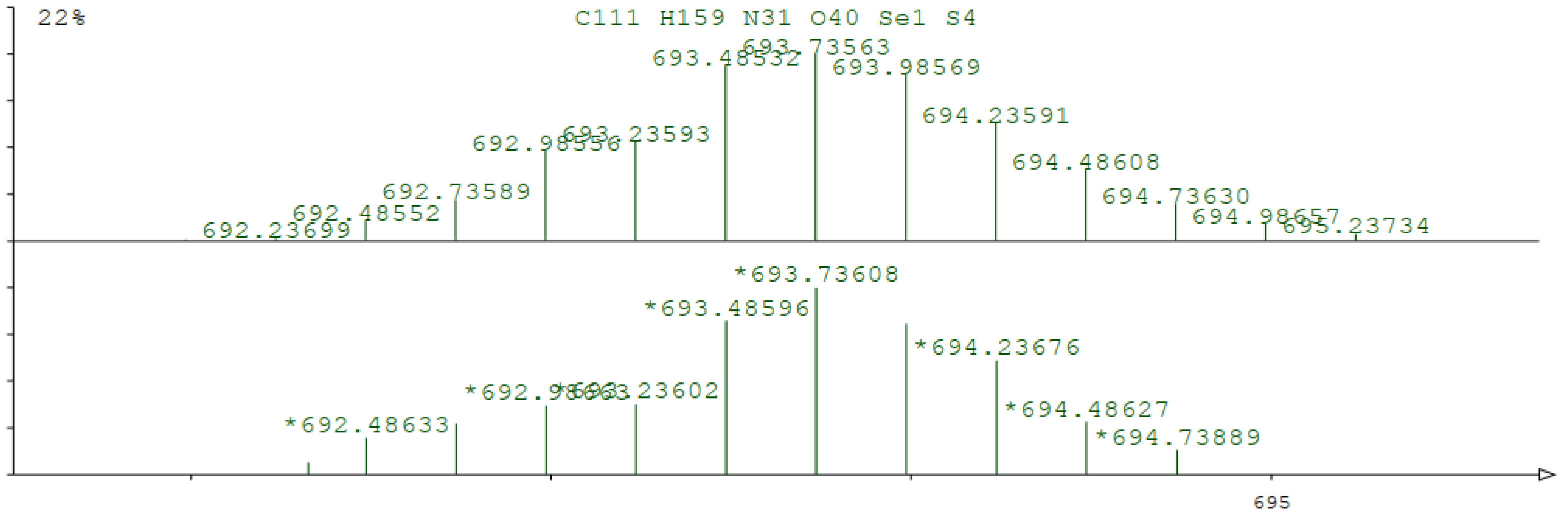

6288(18:58) 1815 6% e51789d-00 * HIO-HA-273-01/NSI/PepMap Neo/75umx50cm/0.1%FA/MeCN+0.1%FA

Mass to be matched (m/z): 693.485960 Charge: 4

Mass Tolerance: ±0.001500
Restriction of atom numbers:

C	H	N	O	Se	S
99-120	99-170	25-35	35-45	1-1	4-4

Number of calculated Formulas: 13

Formula	Diff.(ppm)	theor. m/z
C118 H161 N26 O39 Se1 S4	0.07	693.486007
C102 H161 N34 O44 Se1 S4	-0.23	693.485798
C104 H163 N31 O45 Se1 S4	0.25	693.486134
C116 H159 N29 O38 Se1 S4	-0.42	693.485671
C119 H157 N30 O35 Se1 S4	0.55	693.486341
C105 H159 N35 O41 Se1 S4	0.73	693.486468
C115 H163 N25 O42 Se1 S4	-0.90	693.485337
C114 H157 N32 O37 Se1 S4	-0.90	693.485335
C107 H161 N32 O42 Se1 S4	1.22	693.486804
C113 H161 N28 O41 Se1 S4	-1.38	693.485002
C112 H155 N35 O36 Se1 S4	-1.39	693.484999
C109 H163 N29 O43 Se1 S4	1.70	693.487140
C111 H159 N31 O40 Se1 S4	-1.87	693.484665 <--

HRMS for intact modified peptide #15.
Please note that theor. m/z in the table is the value for a fully resolved monoisotopic signal.
Due to small contributions of lesser isotopologue signals that cannot be resolved at finite instrument resolution the "achievable" theoretical value is shifted.
For the given mass resolution this value will be 693.45830, resulting in an error of -0.95 ppm.

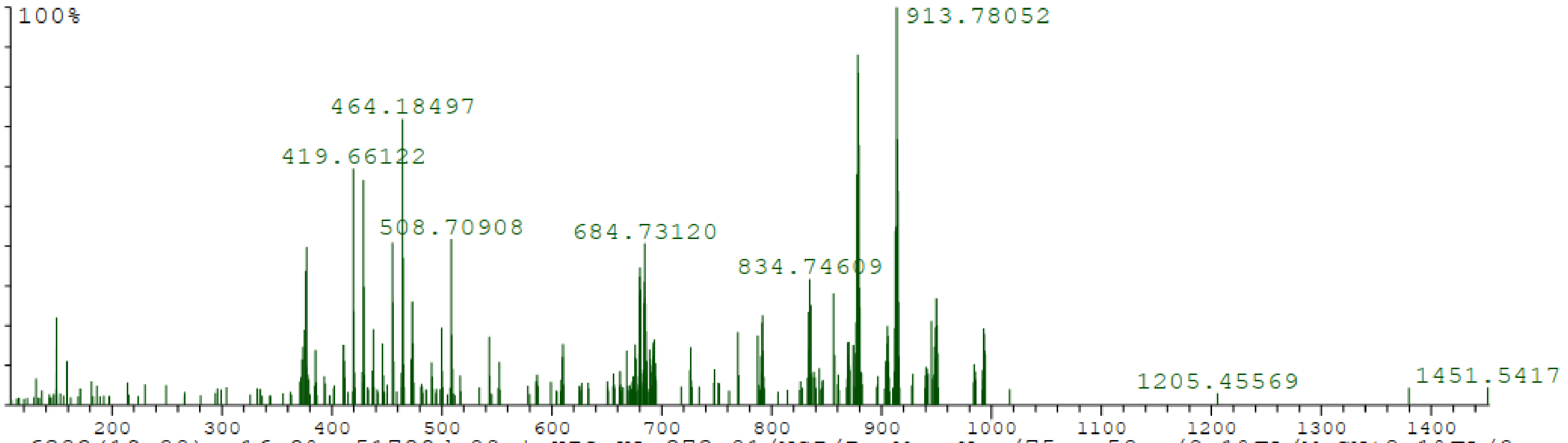

6302(19:00) 16 2% e51789d-00 * HIO-HA-273-01/NSI/PepMap Neo/75umx50cm/0.1%FA/MeCN+0.1%FA/0

MS/MS of peptide #15 ([M + 3H]4+, m/z 693.49)
Fragmetnation: HCD(20.0%)

ion	theory	exp.	Error [ppm]
(b5)+	599.22422	599.22559	-2.28
(b6)+	686.25625	686.25586	+0.57
(b7)+	787.30393	787.30450	-0.73
(b8)+	874.33596	874.33667	-0.82
(b9)+	945.37307	945.37329	-0.23
(b10)+	1016.41018	1016.40649	+3.63
(b6)2+	343.63176	343.63287	-3.22
(b9)2+	473.19017	473.18948	+1.47
(b10)2+	508.70873	508.70908	-0.69
(b11)2+	552.22474	552.22516	-0.75
(y6)+	843.31576	843.31641	-0.77
(y3)+	441.18360	441.18353	+0.17
(y2)+	294.14820	294.14816	+0.15
(y1)+	147.11280	147.11301	-1.40
(y14)2+	992.81373	992.81549	-1.77
(y13)2+	949.29772	949.29724	+0.50
(y12)2+	913.77916	913.78052	-1.49
(y11)2+	878.26060	878.26263	-2.31
(y10)2+	834.74459	834.74609	-1.80
(y9)2+	791.22857	791.22974	-1.47
(y8)2+	747.71256	747.71246	+0.13
(y14)3+	662.21158	662.21387	-3.46
(y13)3+	633.20090	633.20514	-6.69
(y12)3+	609.52186	609.52106	+1.32
(y11)3+	585.84283	585.84552	-4.60

a|b|c|I	Seq.	x|y|z
1	Q	21
2	H	20
3	M	19
4	D	18
5	S	17
6	S	16
7	T	15
8	S	14
9	A	13
10	A	12
11	S	11
12	S	10
13	S	9
14	N	8
15	Y-DBSe	7
16	C-Carbamidomethyl	6
17	N	5
18	Q	4
19	M-Oxidation	3
20	M-Oxidation	2
21	K	1

MS/MS for the peptide 17

10.10.2024 11:57 p.3/26 *** If not stated otherwise, molecular weights refer to the most abundant isotopes of the elements. *** **MassLib**

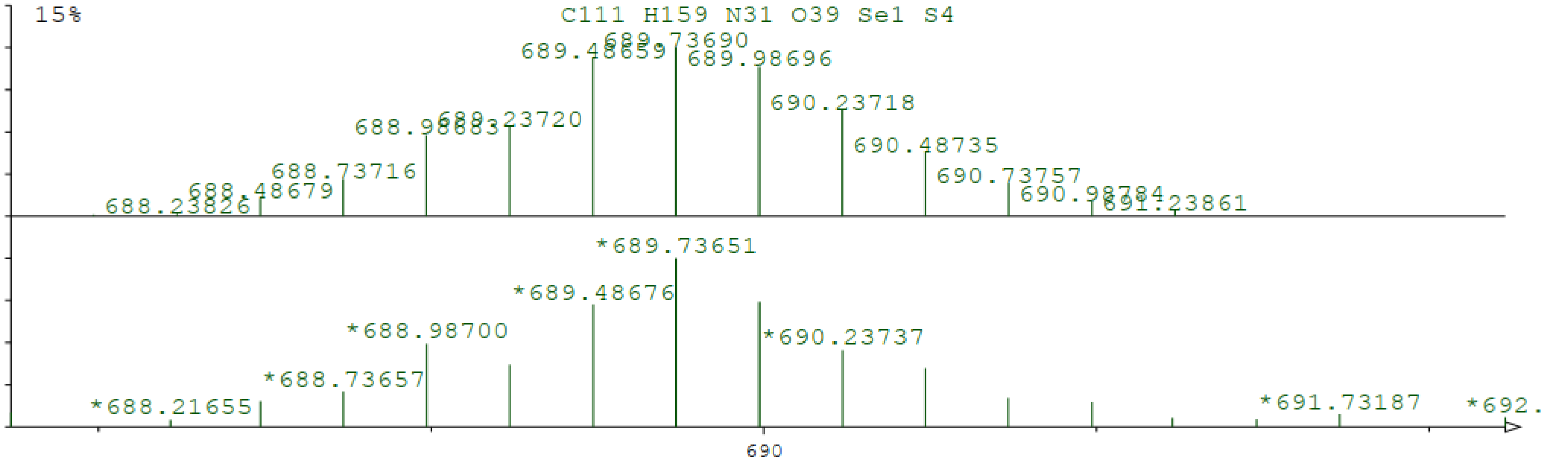

6844(20:14) 7311 5% e51789d-00 * HIO-HA-273-01/NSI/PepMap Neo/75umx50cm/0.1%FA/MeCN+0.1%FA

Mass to be matched (m/z): 689.486760 Charge: 4

Mass Tolerance: ±0.001500
Restriction of atom numbers:

C	H	N	O	Se	S
99-120	99-170	25-35	35-45	1-1	4-4

Number of calculated Formulas: 14

Formula	Diff.(ppm)	theor. m/z	
C115 H163 N25 O41 Se1 S4	-0.22	689.486608	
C114 H157 N32 O36 Se1 S4	-0.22	689.486606	
C116 H159 N29 O37 Se1 S4	0.26	689.486942	
C102 H161 N34 O43 Se1 S4	0.45	689.487069	
C113 H161 N28 O40 Se1 S4	-0.71	689.486273	
C112 H155 N35 O35 Se1 S4	-0.71	689.486270	
C118 H161 N26 O38 Se1 S4	0.75	689.487278	
C104 H163 N31 O44 Se1 S4	0.94	689.487406	
C111 H159 N31 O39 Se1 S4	-1.19	689.485937	<--
C105 H159 N35 O40 Se1 S4	1.42	689.487739	
C106 H165 N28 O45 Se1 S4	1.42	689.487741	
C110 H163 N27 O43 Se1 S4	-1.68	689.485603	
C109 H157 N34 O38 Se1 S4	-1.68	689.485601	
C107 H161 N32 O41 Se1 S4	1.91	689.488075	

HRMS for intact modified peptide #17.
Please note that theor. m/z in the table is the value for a fully resolved monoisotopic signal.
Due to small contributions of lesser isotopologue signals that cannot be resolved at finite instrument resolution the "achievable" theoretical value is shifted.
For the given mass resolution this value will be 689.48657, resulting in an error of -0.28 ppm.

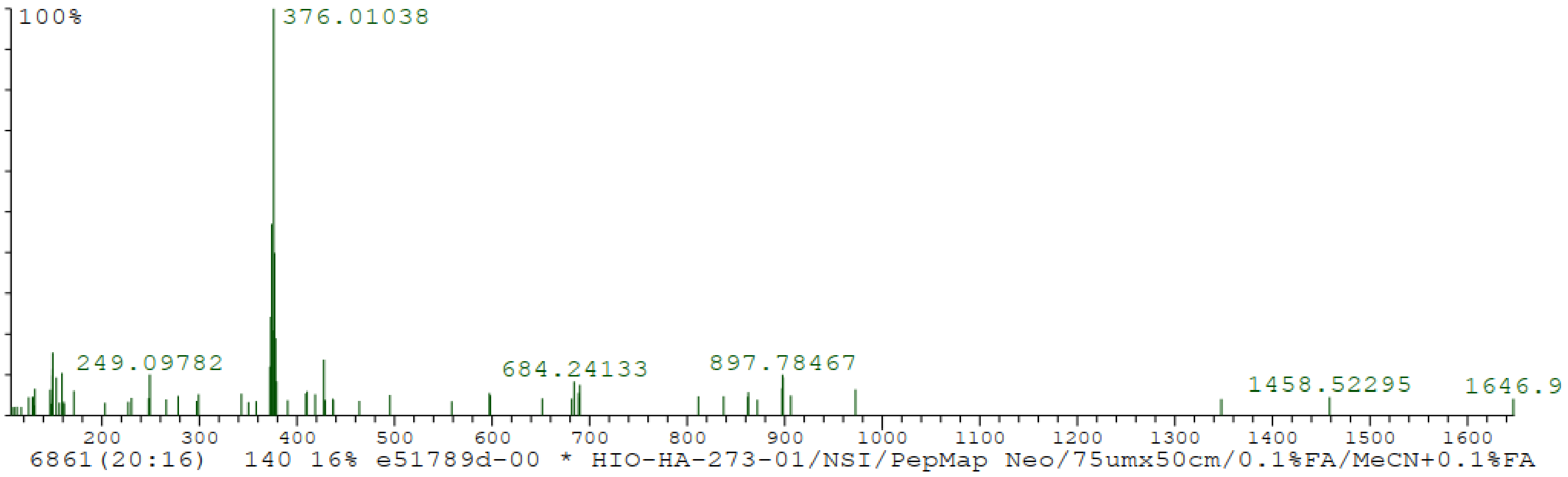

MS/MS of peptide #17 ([M + 3H]4+, m/z 689.49)
Fragmetnation: HCD(30.0%)

ion	theory	exp.	Error [ppm]
(b2)+	266.12477	266.12546	-2.61
(y6)+	811.32593	811.32532	+0.75
(y5)+	651.29528	651.29382	+2.24
(y3)+	409.19377	409.19324	+1.30
(y2)+	278.15329	278.15396	-2.41
(y1)+	147.11280	147.11259	+1.46
(y12)2+	897.78424	897.78467	-0.47

a|b|c|I	Seq.	x|y|z
1	Q	21
2	H	20
3	M-Oxidation	19
4	D	18
5	S	17
6	S	16
7	T	15
8	S	14
9	A	13
10	A	12
11	S	11
12	S	10
13	S	9
14	N	8
15	Y-DBSe	7
16	C-Carbamidomethyl	6
17	N	5
18	Q	4
19	M	3
20	M	2
21	K	1

MS/MS for the peptide 18

10.10.2024 11:57 p.6/26 *** If not stated otherwise, molecular weights refer to the most abundant isotopes of the elements. ***

MassLib

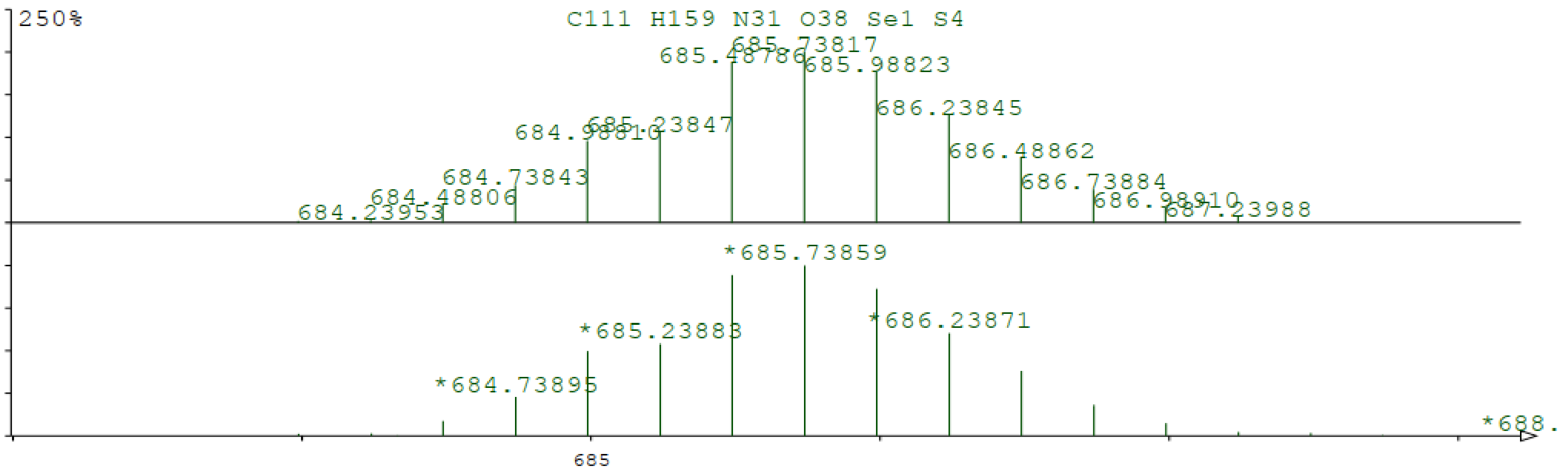

6844(20:14) 7311 5% e51789d-00 * HIO-HA-273-01/NSI/PepMap Neo/75umx50cm/0.1%FA/MeCN+0.1%FA

Mass to be matched (m/z): 685.488280 Charge: 4

Mass Tolerance: ±0.001500
Restriction of atom numbers:

C	H	N	O	Se	S
99-120	99-170	25-35	35-45	1-1	4-4

Number of calculated Formulas: 14

Formula	Diff.(ppm)	theor. m/z	
C102 H161 N34 O42 Se1 S4	0.09	685.488341	
C116 H159 N29 O36 Se1 S4	-0.10	685.488214	
C118 H161 N26 O37 Se1 S4	0.39	685.488549	
C104 H163 N31 O43 Se1 S4	0.58	685.488677	
C115 H163 N25 O40 Se1 S4	-0.58	685.487880	
C114 H157 N32 O35 Se1 S4	-0.59	685.487878	
C105 H159 N35 O39 Se1 S4	1.07	685.489011	
C106 H165 N28 O44 Se1 S4	1.07	685.489012	
C113 H161 N28 O39 Se1 S4	-1.07	685.487544	
C107 H161 N32 O40 Se1 S4	1.56	685.489346	
C108 H167 N25 O45 Se1 S4	1.56	685.489349	
C111 H159 N31 O38 Se1 S4	-1.56	685.487208	<--
C109 H163 N29 O41 Se1 S4	2.05	685.489682	
C110 H163 N27 O42 Se1 S4	-2.05	685.486874	

HRMS for intact modified peptide #18.
Please note that theor. m/z in the table is the value for a fully resolved monoisotopic signal.
Due to small contributions of lesser isotopologue signals that cannot be resolved at finite instrument resolution the "achievable" theoretical value is shifted.
For the given mass resolution this value will be 685.48799, resulting in an error of -0.42 ppm.

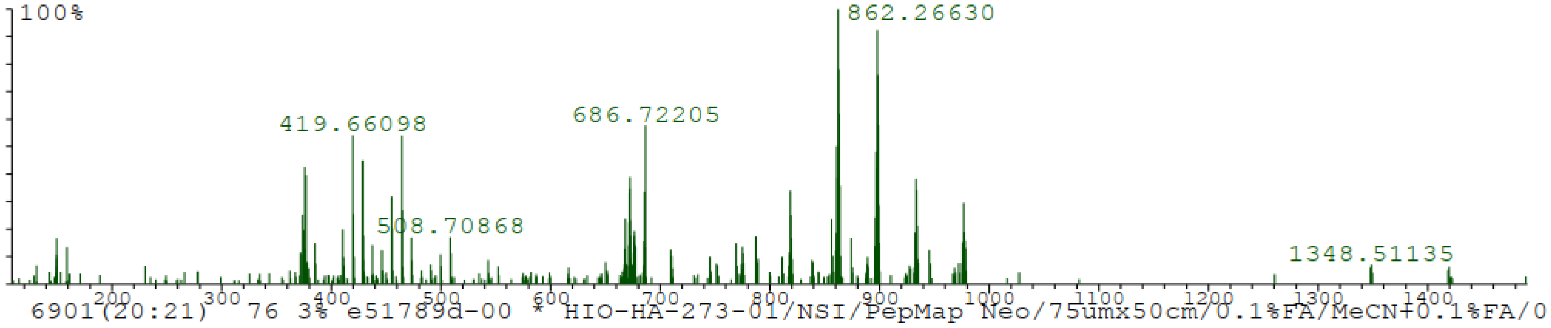

MS/MS of peptide #18 ([M + 3H]4+, m/z 685.49)
Fragmetnation: HCD(20.0%)

ion	theory	exp.	Error [ppm]
(b2)+	266.12477	266.12494	-0.65
(b4)+	512.19219	512.19275	-1.09
(b7)+	787.30393	787.30304	+1.13
(b8)+	874.33596	874.33490	+1.21
(b9)+	945.37307	945.37231	+0.80
(b10)+	1016.41018	1016.40338	+6.69
(b6)2+	343.63176	343.63223	-1.36
(b8)2+	437.67162	437.67044	+2.69
(b9)2+	473.19017	473.18970	+1.00
(b10)2+	508.70873	508.70868	+0.10
(b11)2+	552.22474	552.22430	+0.81
(b15)3+	643.54097	643.54010	+1.35
(I2)+	110.07127	110.07171	-3.96
(y6)+	811.32593	811.32635	-0.52
(y5)+	651.29528	651.29224	+4.66
(y4)+	537.25235	537.25220	+0.28
(y3)+	409.19377	409.19208	+4.14
(y2)+	278.15329	278.15341	-0.44
(y1)+	147.11280	147.11267	+0.91
(y15)2+	1027.34265	1027.34033	+2.26
(y14)2+	976.81882	976.81854	+0.28
(y13)2+	933.30280	933.30273	+0.08
(y12)2+	897.78424	897.78430	-0.06
(y11)2+	862.26569	862.26630	-0.71

ion	theory	exp.	Error [ppm]
(y10)2+	818.74967	818.75024	-0.69
(y9)2+	775.23366	775.23346	+0.26
(y8)2+	731.71764	731.71667	+1.33
(y14)3+	651.54830	651.54919	-1.36
(y12)3+	598.85859	598.85828	+0.51
(y11)3+	575.17955	575.17981	-0.45
(y10)3+	546.16887	546.16949	-1.13

a|b|c|I	Seq.	x|y|z
1	Q	21
2	H	20
3	M	19
4	D	18
5	S	17
6	S	16
7	T	15
8	S	14
9	A	13
10	A	12
11	S	11
12	S	10
13	S	9
14	N	8
15	Y-DBSe	7
16	C-Carb.	6
17	N	5
18	Q	4
19	M	3
20	M	2
21	K	1

MS/MS for the peptide 20

10.10.2024 11:57 p.7/26 *** If not stated otherwise, molecular weights refer to the most abundant isotopes of the elements. *** **MassLib**

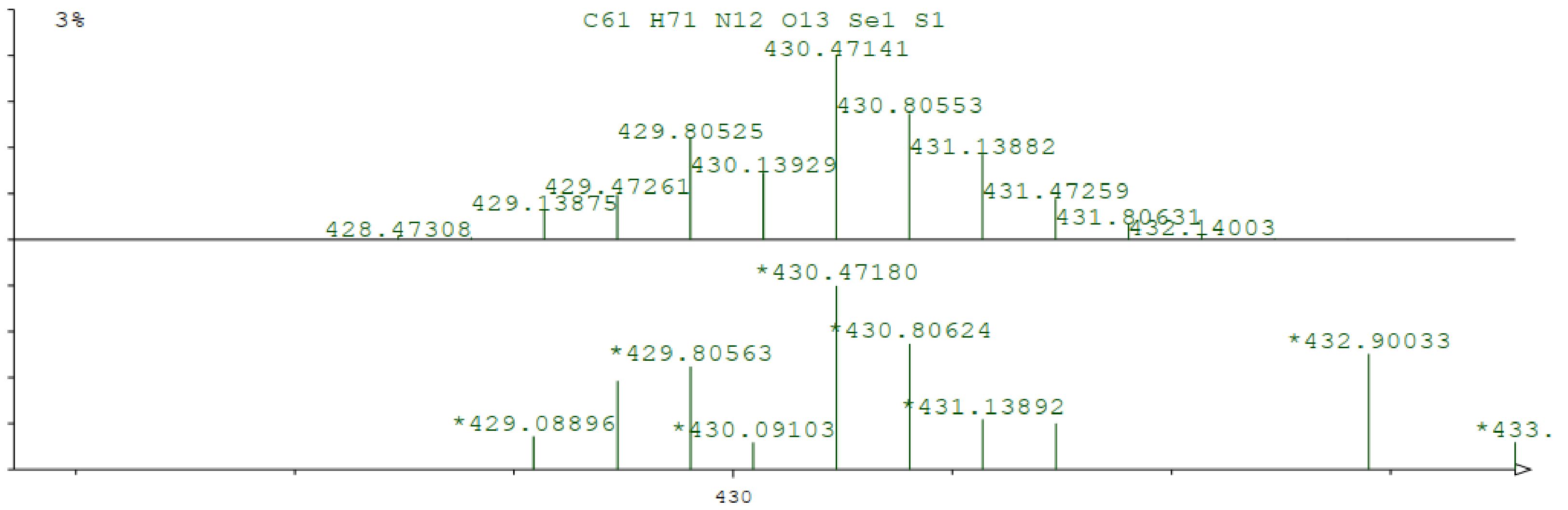

6985(20:31) 9745 7% e51789d-00 * HIO-HA-273-01/NSI/PepMap Neo/75umx50cm/0.1%FA/MeCN+0.1%FA

Mass to be matched (m/z): 430.471800 Charge: 3

Mass Tolerance: ±0.001500
Restriction of atom numbers:

C	H	N	O	Se	S
50-70	60-80	7-17	7-17	1-1	1-1

Number of calculated Formulas: 10

Formula	Diff. (ppm)	theor. m/z	
C64 H69 N13 O10 Se1 S1	0.44	430.471989	
C63 H73 N9 O14 Se1 S1	-0.59	430.471544	
C62 H67 N16 O9 Se1 S1	-0.60	430.471541	
C66 H71 N10 O11 Se1 S1	1.48	430.472437	
C61 H71 N12 O13 Se1 S1	-1.63	430.471096	<--
C52 H73 N15 O17 Se1 S1	1.87	430.472606	
C67 H67 N14 O7 Se1 S1	2.51	430.472882	
C68 H73 N7 O12 Se1 S1	2.52	430.472885	
C60 H75 N8 O17 Se1 S1	-2.67	430.470651	
C59 H69 N15 O12 Se1 S1	-2.68	430.470648	

HRMS for intact modified peptide #20.
Please note that theor. m/z in the table is the value for a fully resolved monoisotopic signal.
Due to small contributions of lesser isotopologue signals that cannot be resolved at finite instrument resolution the "achievable" theoretical value is shifted.
For the given mass resolution this value will be 430.47132, resulting in an error of -1.12 ppm.

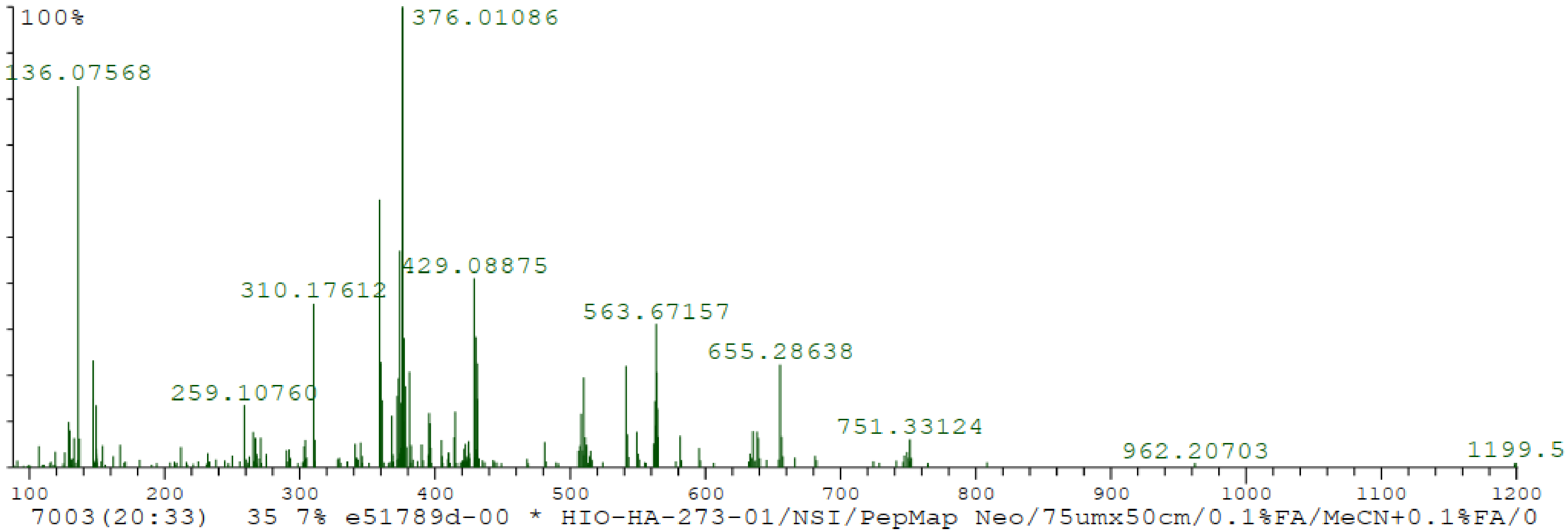

7003(20:33) 35 7% e51789d-00 * HIO-HA-273-01/NSI/PepMap Neo/75umx50cm/0.1%FA/MeCN+0.1%FA/0

ion	theory	exp.	Error [ppm]
(b2)+	635.11920	635.11902	+0.28
(b3)+	749.16213	749.16150	+0.84
(I1)+	510.07152	510.07132	+0.39
(I4)+	133.04301	133.04303	-0.15
(I6)+	136.07569	136.07543	+1.94
(y6)+	752.33959	752.33307	+8.66
(y5)+	655.28682	655.28638	+0.67
(y4)+	541.24389	541.24371	+0.34
(y3)+	381.21325	381.21326	-0.04
(y2)+	310.17613	310.17612	+0.04
(y1)+	147.11280	147.11312	-2.17
(y6)2+	376.67343	376.67331	+0.32
(y5)2+	328.14705	328.14758	-1.62
(y4)2+	271.12559	271.12537	+0.80

MS/MS of peptide #20 ([M + 2H]3+, m/z 430.47)
Fragmetnation: HCD(20.0%)

a|b|c|I	Seq.	x|y|z
1	Y-DBSe	7
2	P	6
3	N	5
4	C-Carbamidomethyl	4
5	A	3
6	Y	2
7	K	1

MS/MS for the peptide 22

10.10.2024 11:57 p.8/26 *** If not stated otherwise, molecular weights refer to the most abundant isotopes of the elements. *** **MassLib**

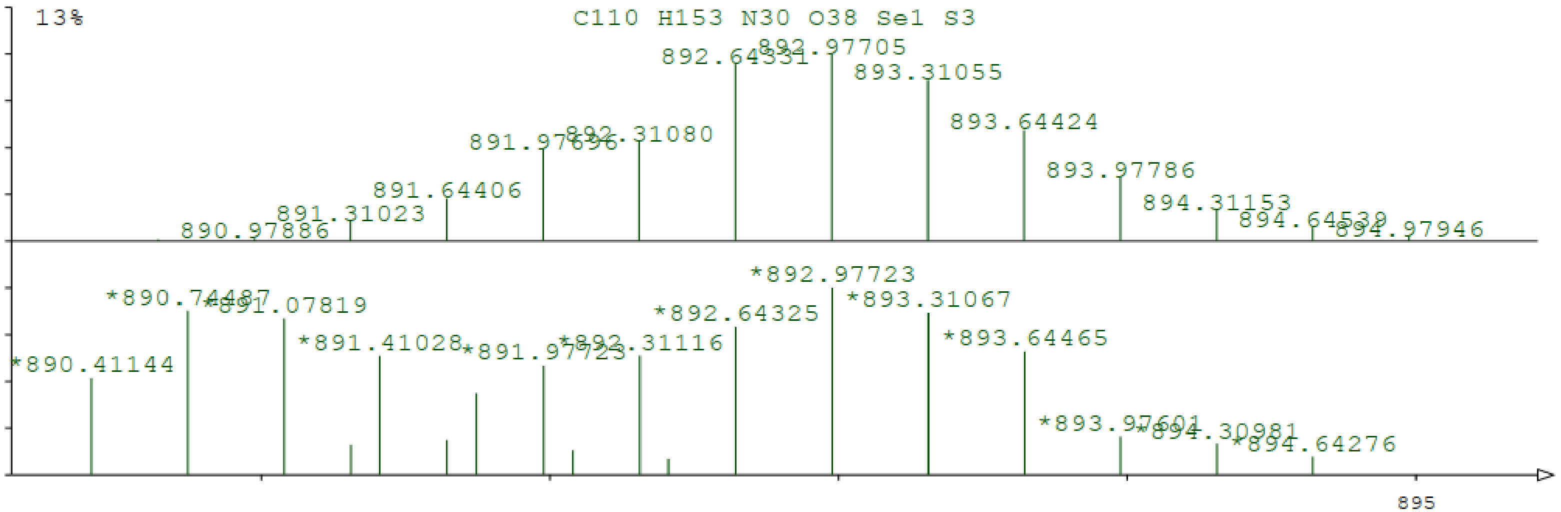

7469(21:35) 4590 6% e51789d-00 * HIO-HA-273-01/NSI/PepMap Neo/75umx50cm/0.1%FA/MeCN+0.1%FA

Mass to be matched (m/z): 892.643250 Charge: 3

Mass Tolerance: ±0.001500
Restriction of atom numbers:

C	H	N	O	Se	S
99-120	99-160	25-35	32-42	1-1	3-3

Number of calculated Formulas: 11

Formula	Diff.(ppm)	theor. m/z	
C113 H151 N31 O35 Se1 S3	0.08	892.643322	
C112 H155 N27 O39 Se1 S3	-0.42	892.642877	
C111 H149 N34 O34 Se1 S3	-0.42	892.642873	
C115 H153 N28 O36 Se1 S3	0.58	892.643769	
C101 H155 N33 O42 Se1 S3	0.77	892.643939	
C110 H153 N30 O38 Se1 S3	-0.92	892.642428	<--
C116 H149 N32 O32 Se1 S3	1.08	892.644214	
C117 H155 N25 O37 Se1 S3	1.08	892.644218	
C109 H157 N26 O42 Se1 S3	-1.42	892.641984	
C108 H151 N33 O37 Se1 S3	-1.42	892.641981	
C118 H151 N29 O33 Se1 S3	1.58	892.644662	

HRMS for intact modified peptide #22.
Please note that theor. m/z in the table is the value for a fully resolved monoisotopic signal.
Due to small contributions of lesser isotopologue signals that cannot be resolved at finite instrument resolution the "achievable" theoretical value is shifted.
For the given mass resolution this value will be 892.64318, resulting in an error of -0.08 ppm.

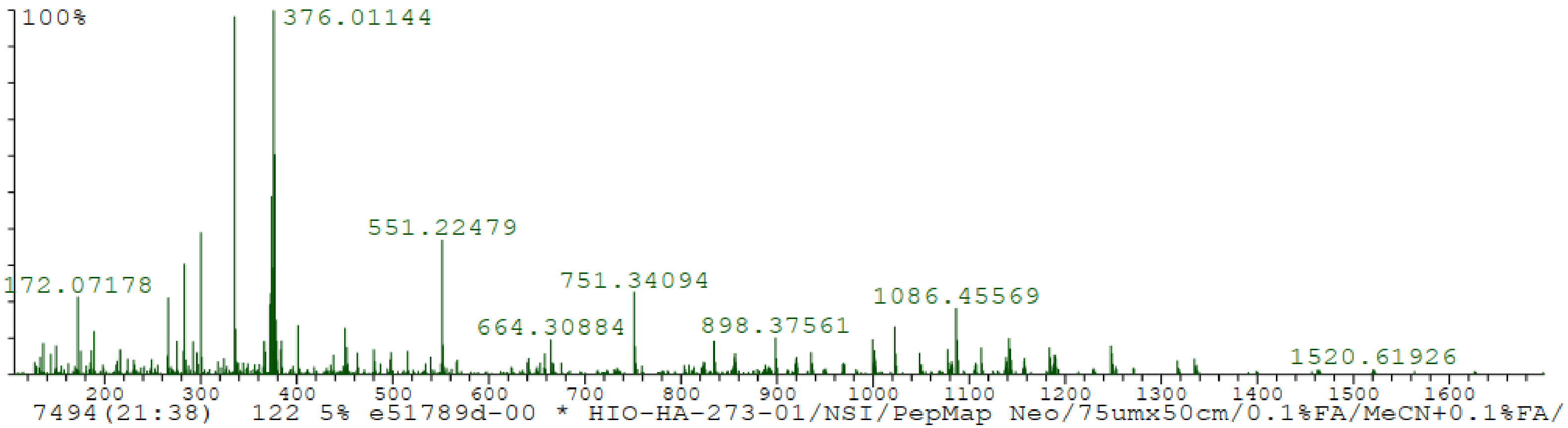

MS/MS of peptide #22 ([M + 2H]3+, m/z 892.64)
Fragmetnation: HCD(30.0%)

ion	theory	exp.	Error [ppm]
(b1)+	115.05020	115.05029	-0.75
(b2)+	172.07167	172.07178	-0.65
(b3)+	300.13025	300.13055	-1.02
(b4)+	401.17792	401.17822	-0.74
(b5)+	515.22085	515.22156	-1.38
(b6)+	675.25150	675.25232	-1.22
(I6)+	133.04301	133.04318	-1.27
(I7)+	136.07569	136.07585	-1.16
(I10)+	510.07152	510.07141	+0.22
(I18)+	133.04301	133.04318	-1.27
(y9)+	1086.45542	1086.45569	-0.25
(y8)+	999.42339	999.42322	+0.17
(y7)+	898.37571	898.37561	+0.12
(y6)+	751.34031	751.34094	-0.83
(y5)+	664.30829	664.30884	-0.83
(y4)+	551.22422	551.22479	-1.03
(y3)+	450.17654	450.17703	-1.08
(y2)+	335.14960	335.14981	-0.62
(y1)+	175.11895	175.11911	-0.90

ion	theory	exp.	Error [ppm]
(y16)2+	1188.89852	1188.90051	-1.67
(y15)2+	1138.37468	1138.37878	-3.60
(y14)2+	1081.35322	1081.35645	-2.99
(y13)2+	1001.33790	1001.34161	-3.71
(y12)2+	919.80623	919.80762	-1.51
(y11)2+	855.77694	855.77869	-2.04
(y10)2+	812.26093	812.26648	-6.84
(y7)2+	449.69150	449.69208	-1.30
(y2)2+	168.07844	168.07738	+6.30
(y14)3+	721.23791	721.24304	-7.12

a|b|c|I	Seq.	x|y|z
1	N	19
2	G	18
3	Q	17
4	T	16
5	N	15
6	C-Carb.	14
7	Y	13
8	Q	12
9	S	11
10	Y-DBSe	10
11	S	9
12	T	8
13	M-Ox.	7
14	S	6
15	I	5
16	T	4
17	D	3
18	C-Carb.	2
19	R	1

MS/MS for the peptide 23

10.10.2024 11:57 p.9/26 *** If not stated otherwise, molecular weights refer to the most abundant isotopes of the elements. ***

MassLib

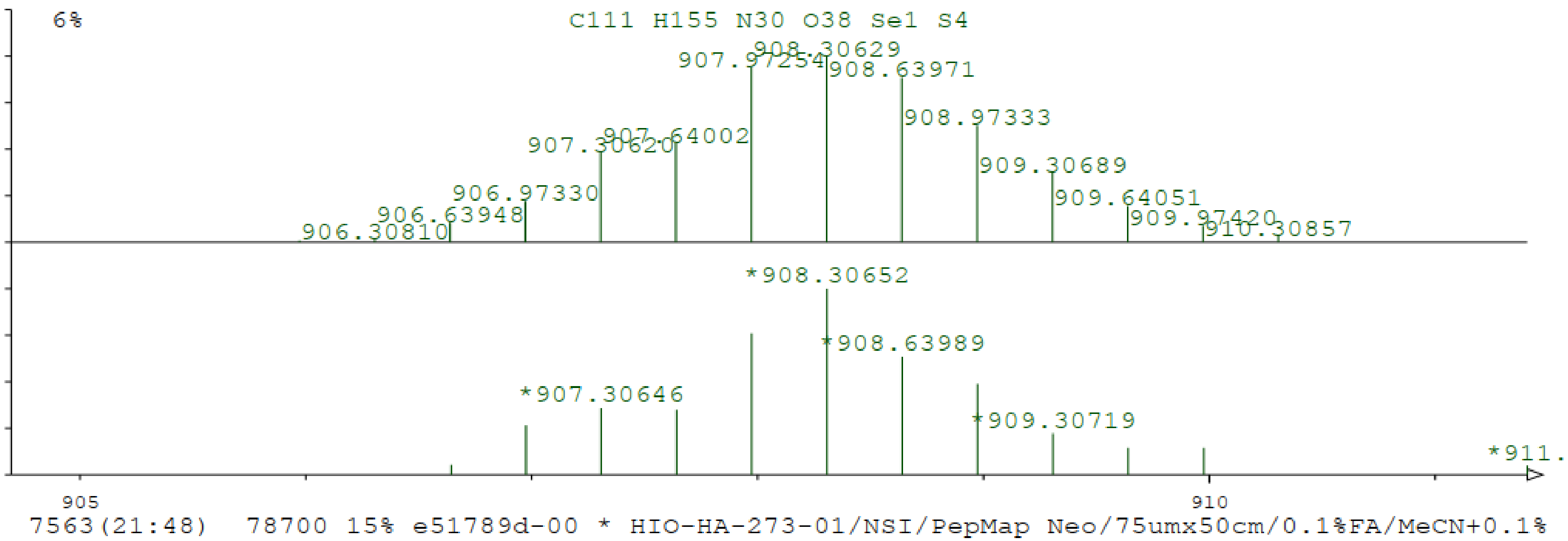

Mass to be matched (m/z): 907.972780 Charge: 3

Mass Tolerance: ±0.001500
Restriction of atom numbers:

C	H	N	O	Se	S
99-120	99-160	25-35	32-42	1-1	4-4

Number of calculated Formulas: 10

Formula	Diff.(ppm)	theor. m/z	
C114 H153 N31 O35 Se1 S4	-0.24	907.972562	
C116 H155 N28 O36 Se1 S4	0.25	907.973010	
C102 H157 N33 O42 Se1 S4	0.44	907.973180	
C113 H157 N27 O39 Se1 S4	-0.73	907.972117	
C112 H151 N34 O34 Se1 S4	-0.73	907.972114	
C117 H151 N32 O32 Se1 S4	0.74	907.973455	
C118 H157 N25 O37 Se1 S4	0.75	907.973458	
C111 H155 N30 O38 Se1 S4	-1.22	907.971669	<--
C119 H153 N29 O33 Se1 S4	1.24	907.973903	
C105 H155 N34 O39 Se1 S4	1.42	907.974073	

HRMS for intact modified peptide #23.
Please note that theor. m/z in the table is the value for a fully resolved monoisotopic signal.
Due to small contributions of lesser isotopologue signals that cannot be resolved at finite instrument resolution the "achievable" theoretical value is shifted.
For the given mass resolution this value will be 907.97277, resulting in an error of -0.01 ppm.

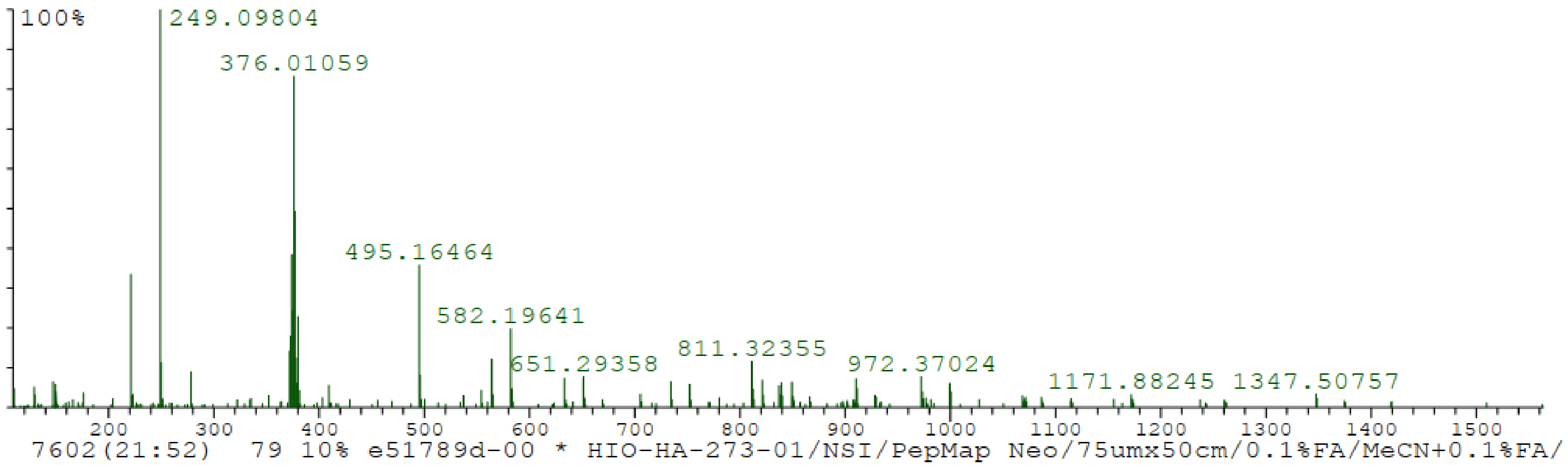

MS/MS of peptide #23 ([M + 2H]3+, m/z 907.97)
Fragmetnation: HCD(30.0%)

ion	theory	exp.	Error [ppm]
(b2)+	249.09822	249.09804	+0.71
(b3)+	380.13870	380.13821	+1.29
(b4)+	495.16564	495.16464	+2.03
(b5)+	582.19767	582.19641	+2.17
(b6)+	669.22970	669.22717	+3.78
(b7)+	770.27738	770.27643	+1.23
(b8)+	857.30941	857.30859	+0.95
(b9)+	928.34652	928.34351	+3.24
(b10)+	999.38364	999.38013	+3.51
(b11)+	1086.41566	1086.40833	+6.75
(b13)+	1260.47972	1260.47424	+4.35
(b14)+	1374.52265	1374.52429	-1.19
(b10)2+	500.19546	500.19495	+1.01
(I2)+	110.07127	110.07138	-0.96
(I16)+	133.04301	133.04326	-1.88
(y6)+	811.32593	811.32355	+2.93
(y5)+	651.29528	651.29358	+2.61
(y4)+	537.25235	537.25134	+1.88
(y3)+	409.19377	409.19284	+2.28
(y2)+	278.15329	278.15292	+1.33
(y1)+	147.11280	147.11282	-0.11

ion	theory	exp.	Error [ppm]
((y19)2+	1237.40840	1237.40564	+2.23
(y18)2+	1171.88815	1171.88245	+4.87
(y17)2+	1114.37468	1114.36877	+5.31
(y16)2+	1070.85867	1070.85645	+2.07
(y15)2+	1027.34265	1027.34363	-0.95
(y14)2+	976.81882	976.81641	+2.46
(y13)2+	933.30280	933.30255	+0.27
(y12)2+	897.78424	897.78259	+1.84
(y11)2+	862.26569	862.26605	-0.42

a|b|c|I	Seq.	x|y|z
1	Q-Gln	21
2	H	20
3	M	19
4	D	18
5	S	17
6	S	16
7	T	15
8	S	14
9	A	13
10	A	12
11	S	11
12	S	10
13	S	9
14	N	8
15	Y-DBSe	7
16	C-Carb.	6
17	N	5
18	Q	4
19	M	3
20	M	2
21	K	1

MS/MS for the peptide 26

10.10.2024 11:57 p.10/26 ***If not stated otherwise, molecular weights refer to the most abundant isotopes of the elements. *** MassLib

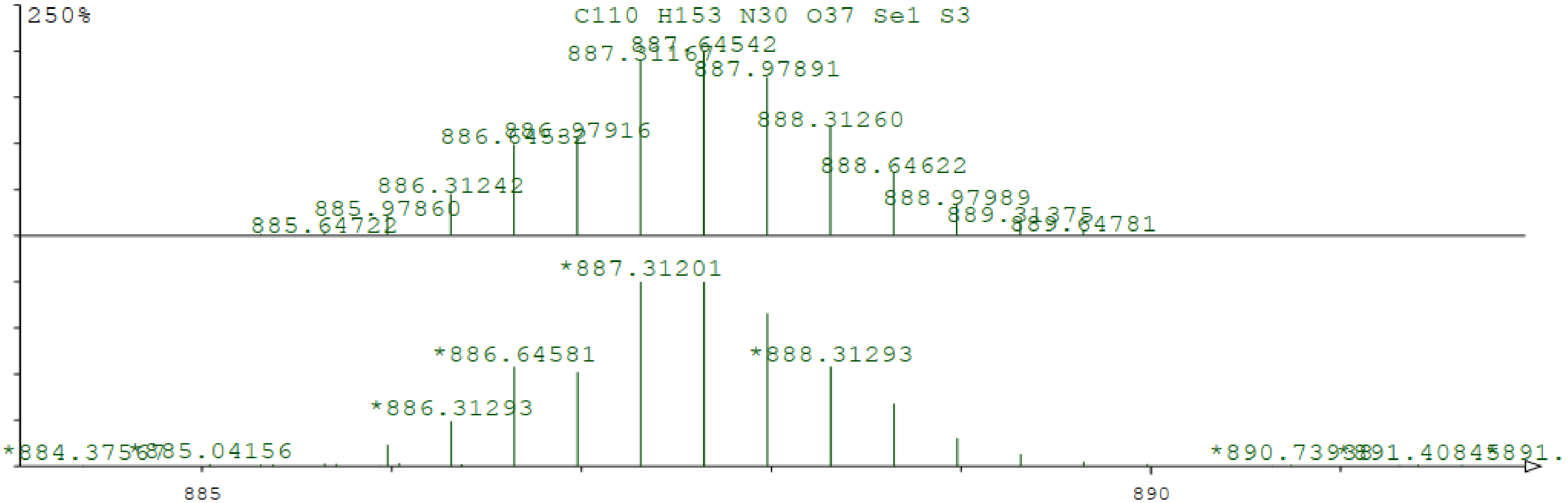

7812(22:20) 8865 6% e51789d-00 * HIO-HA-273-01/NSI/PepMap Neo/75umx50cm/0.1%FA/MeCN+0.1%FA

Mass to be matched (m/z): 887.312010 Charge: 3

Mass Tolerance: ±0.001500
Restriction of atom numbers:

C	H	N	O	Se	S
99-120	99-160	25-35	32-42	1-1	3-3

Number of calculated Formulas: 11

Formula	Diff.(ppm)	theor. m/z	
C115 H153 N28 O35 Se1 S3	0.14	887.312131	
C101 H155 N33 O41 Se1 S3	0.33	887.312300	
C113 H151 N31 O34 Se1 S3	-0.37	887.311683	
C117 H155 N25 O36 Se1 S3	0.64	887.312579	
C103 H157 N30 O42 Se1 S3	0.83	887.312749	
C112 H155 N27 O38 Se1 S3	-0.87	887.311238	
C111 H149 N34 O33 Se1 S3	-0.87	887.311235	
C118 H151 N29 O32 Se1 S3	1.14	887.313024	
C104 H153 N34 O38 Se1 S3	1.33	887.313194	
C110 H153 N30 O37 Se1 S3	-1.37	887.310790	<--
C120 H153 N26 O33 Se1 S3	1.65	887.313472	

HRMS for intact modified peptide #26.
Please note that theor. m/z in the table is the value for a fully resolved monoisotopic signal.
Due to small contributions of lesser isotopologue signals that cannot be resolved at finite instrument resolution the "achievable" theoretical value is shifted.
For the given mass resolution this value will be 887.31190, resulting in an error of -0.12 ppm.

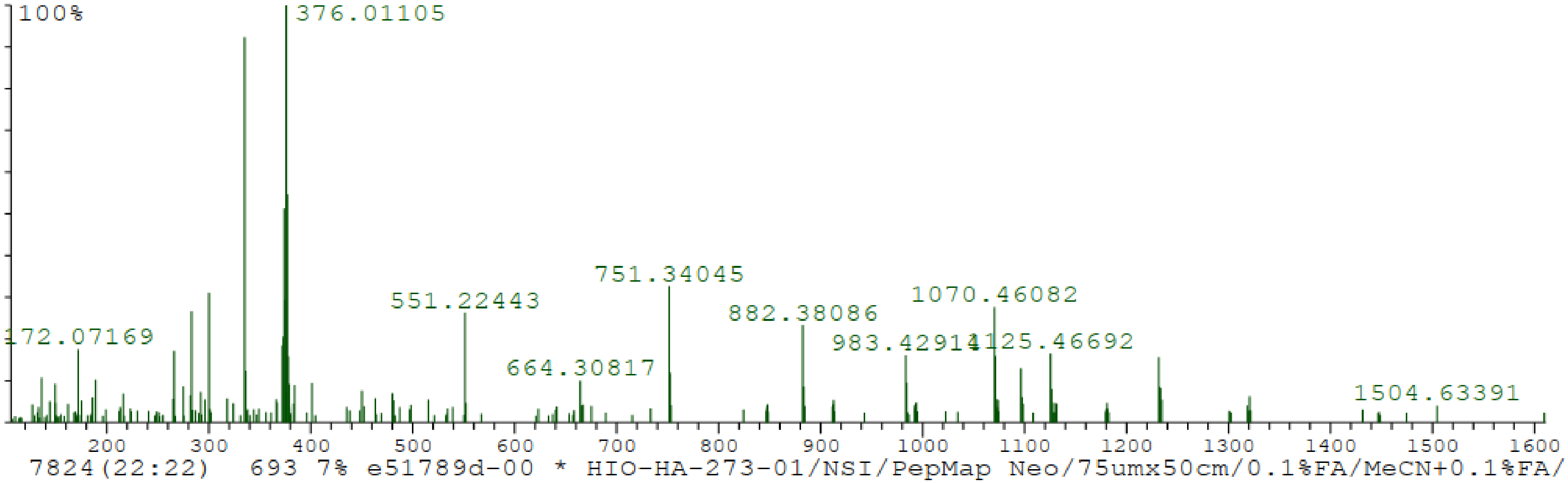

ion	theory	exp.	Error [ppm]
(b2)+	172.07167	172.07169	-0.13
(b3)+	300.13025	300.13031	-0.22
(b4)+	401.17792	401.17734	+1.45
(b5)+	515.22085	515.22119	-0.66
(b6)+	675.25150	675.25226	-1.13
(I6)+	133.04301	133.04265	+2.71
(I10)+	136.07569	136.07565	+0.30
(I18)+	133.04301	133.04265	+2.71
(y12)+	1448.61444	1448.61267	+1.22
(y11)+	1320.55586	1320.54883	+5.33
(y10)+	1233.52383	1233.51575	+6.55
(y9)+	1070.46051	1070.46082	-0.29
(y8)+	983.42848	983.42914	-0.67
(y7)+	882.38080	882.38086	-0.07
(y6)+	751.34031	751.34045	-0.18
(y5)+	664.30829	664.30817	+0.17
(y4)+	551.22422	551.22443	-0.38
(y3)+	450.17654	450.17636	+0.41
(y2)+	335.14960	335.14960	+0.00
(y1)+	175.11895	175.11871	+1.38
(y16)2+	1180.90106	1180.90210	-0.88
(y15)2+	1130.37723	1130.37964	-2.14
(y14)2+	1073.35576	1073.35254	+3.00
(y13)2+	993.34044	993.34088	-0.45

MS/MS of peptide #26 ([M + 2H]3+, m/z 887.31)
Fragmetnation: HCD(30.0%)

a|b|c|I	Seq.	x|y|z
1	N	19
2	G	18
3	Q	17
4	T	16
5	N	15
6	C-Carbamidomethyl	14
7	Y-DBSe	13
8	Q	12
9	S	11
10	Y	10
11	S	9
12	T	8
13	M	7
14	S	6
15	I	5
16	T	4
17	D	3
18	C-Carbamidomethyl	2
19	R	1

MS/MS for the peptide 27

10.10.2024 11:57 p.11/26 ***If not stated otherwise, molecular weights refer to the most abundant isotopes of the elements. *** **MassLib**

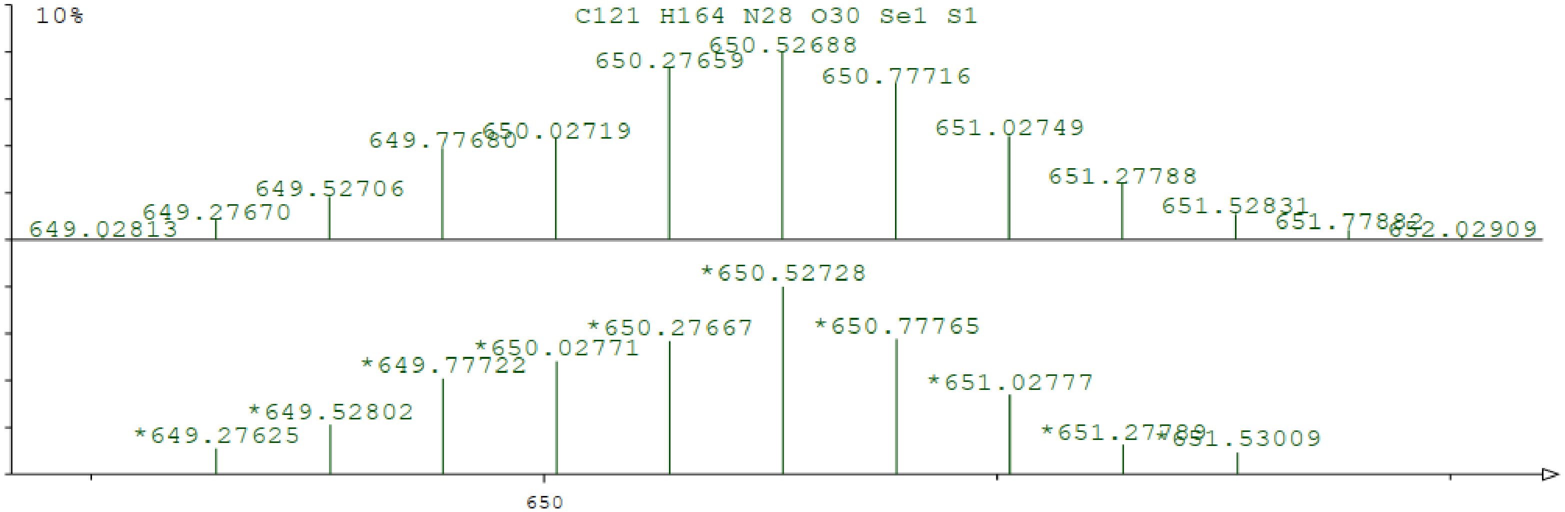

7920(22:34) 48931 13% e51789d-00 * HIO-HA-273-01/NSI/PepMap Neo/75umx50cm/0.1%FA/MeCN+0.1%

```
Mass to be matched (m/z): 650.276670  Charge: 4

Mass Tolerance: ±0.001500
Restriction of atom numbers:
C       H       N       O       Se      S
99-130  99-170  25-35   25-35   1-1     1-1
Number of calculated Formulas:  14
```

Formula	Diff.(ppm)	theor. m/z	
C110 H164 N34 O33 Se1 S1	-0.11	650.276596	
C126 H164 N26 O28 Se1 S1	0.21	650.276805	
C124 H162 N29 O27 Se1 S1	-0.31	650.276469	
C112 H166 N31 O34 Se1 S1	0.40	650.276932	
C123 H166 N25 O31 Se1 S1	-0.82	650.276135	
C122 H160 N32 O26 Se1 S1	-0.83	650.276133	
C113 H162 N35 O30 Se1 S1	0.92	650.277266	
C114 H168 N28 O35 Se1 S1	0.92	650.277268	
C129 H162 N27 O25 Se1 S1	1.24	650.277475	
C121 H164 N28 O30 Se1 S1	-1.34	650.275799	<--
C120 H158 N35 O25 Se1 S1	-1.34	650.275797	
C115 H164 N32 O31 Se1 S1	1.43	650.277602	
C119 H162 N31 O29 Se1 S1	-1.86	650.275464	
C117 H166 N29 O32 Se1 S1	1.95	650.277938	

HRMS for intact modified peptide #27.
Please note that theor. m/z in the table is the value for a fully resolved monoisotopic signal.
Due to small contributions of lesser isotopologue signals that cannot be resolved at finite instrument resolution the "achievable" theoretical value is shifted.
For the given mass resolution this value will be 650.27662, resulting in an error of -0.08 ppm.

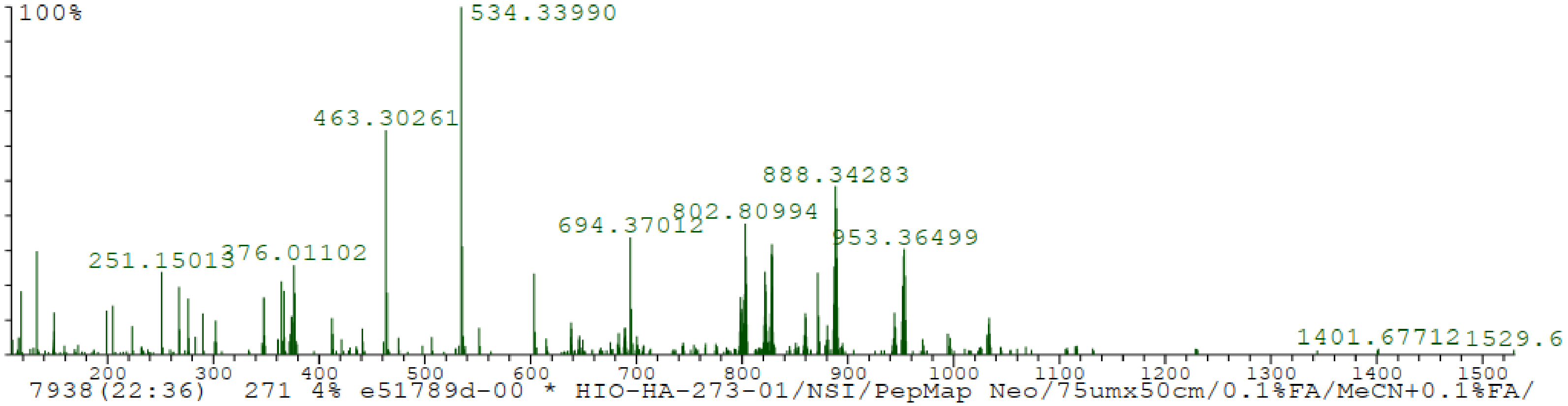

MS/MS of peptide #27 ([M + 3H]4+, m/z 650.28)
Fragmetnation: HCD(20.0%)

ion	theory	exp.	Error [ppm]
(b2)+	251.15025	251.14925	+3.99
(b3)+	364.23432	364.23419	+0.35
(b4)+	463.30273	463.30261	+0.26
(b5)+	534.33984	534.33990	-0.10
(b6)+	694.37049	694.37012	+0.54
(b7)+	823.41309	823.41382	-0.89
(b8)+	880.43455	880.43237	+2.47
(b9)+	994.47748	994.47607	+1.41
(b4)2+	232.15500	232.15459	+1.78
(b5)2+	267.67356	267.67346	+0.37
(b6)2+	347.68888	347.68887	+0.04
(b7)2+	412.21018	412.21021	-0.07
(b8)2+	440.72091	440.72101	-0.22
(b9)2+	497.74238	497.74420	-3.66
(b17)3+	774.98260	774.98407	-1.89
(b18)3+	798.66164	798.66333	-2.11
(b19)3+	827.67232	827.67413	-2.19
(I1)+	110.07127	110.07103	+2.22
(I6)+	133.04301	133.04300	+0.08
(I15)+	110.07127	110.07103	+2.22

ion	theory	exp.	Error [ppm]
(y9)+	970.49926	970.49860	+0.68
(y8)+	871.43084	871.43066	+0.21
(y6)+	675.30967	675.30878	+1.31
(y5)+	538.25075	538.25244	-3.13
(y3)+	276.15540	276.15707	-6.06
(y2)+	205.11828	205.11819	+0.46
(y1)+	118.08626	118.08627	-0.13
(y16)2+	1068.39660	1068.39648	+0.11
(y15)2+	1032.87804	1032.87891	-0.84
(y14)2+	952.86272	952.86420	-1.55
(y13)2+	888.34142	888.34283	-1.58
(y12)2+	859.83069	859.83234	-1.92
(y11)2+	802.80923	802.80994	-0.89
(y8)2+	436.21906	436.21982	-1.74
(y16)3+	712.60016	712.59509	+7.11
(y15)3+	688.92112	688.92157	-0.65

a|b|c|I	Seq.	x|y|z
1	H	20
2	I	19
3	I	18
4	V	17
5	A	16
6	C-Carb.	15
7	E	14
8	G	13
9	N	12
10	P	11
11	Y-DBSe	10
12	V	9
13	P	8
14	V	7
15	H	6
16	F	5
17	D	4
18	A	3
19	S	2
20	V	1

MS/MS for the peptide 28

10.10.2024 11:57 p.12/26 *** If not stated otherwise, molecular weights refer to the most abundant isotopes of the elements. ***

MassLib

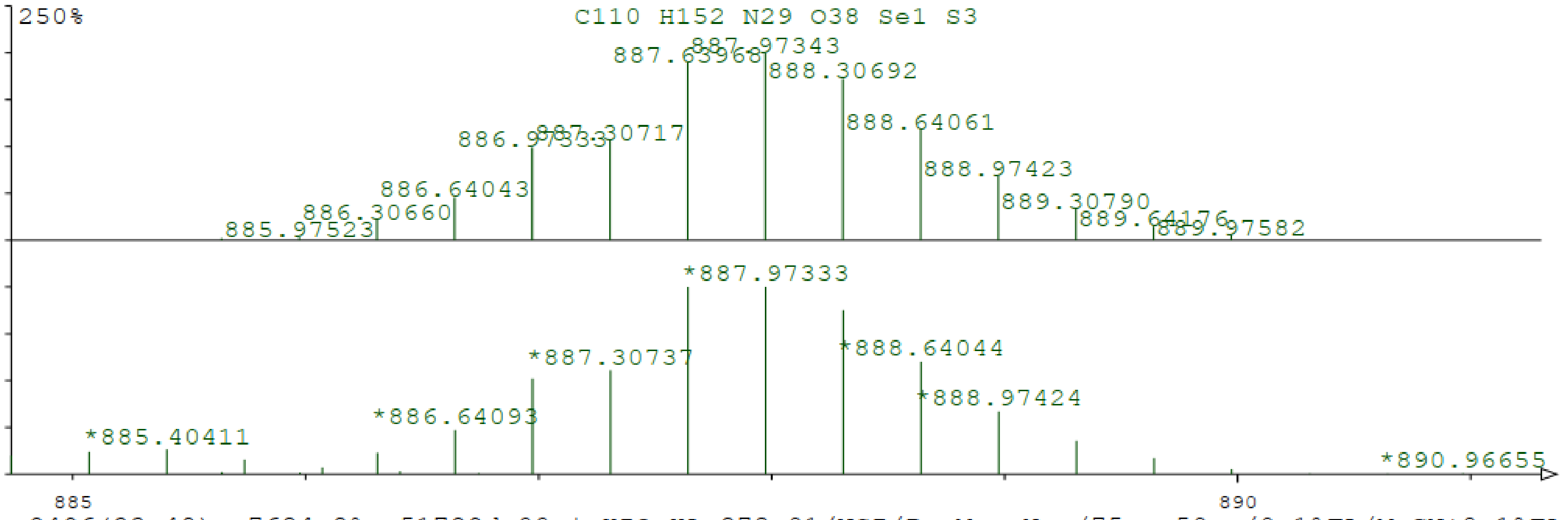

8496(23:49) 7624 8% e51789d-00 * HIO-HA-273-01/NSI/PepMap Neo/75umx50cm/0.1%FA/MeCN+0.1%FA

Mass to be matched (m/z): 887.639950 Charge: 3

Mass Tolerance: ±0.001500
Restriction of atom numbers:

C	H	N	O	Se	S
99-120	99-160	25-35	35-45	1-1	3-3

Number of calculated Formulas: 8

Formula	Diff.(ppm)	theor. m/z	
C115 H152 N27 O36 Se1 S3	0.21	887.640136	
C113 H150 N30 O35 Se1 S3	-0.29	887.639688	
C101 H154 N32 O42 Se1 S3	0.40	887.640306	
C112 H154 N26 O39 Se1 S3	-0.80	887.639243	
C103 H156 N29 O43 Se1 S3	0.91	887.640754	
C110 H152 N29 O38 Se1 S3	-1.30	887.638796	<--
C104 H152 N33 O39 Se1 S3	1.41	887.641199	
C105 H158 N26 O44 Se1 S3	1.41	887.641202	

HRMS for intact modified peptide #28.
Please note that theor. m/z in the table is the value for a fully resolved monoisotopic signal.
Due to small contributions of lesser isotopologue signals that cannot be resolved at finite instrument resolution the "achievable" theoretical value is shifted.
For the given mass resolution this value will be 887.63942, resulting in an error of -0.60 ppm.

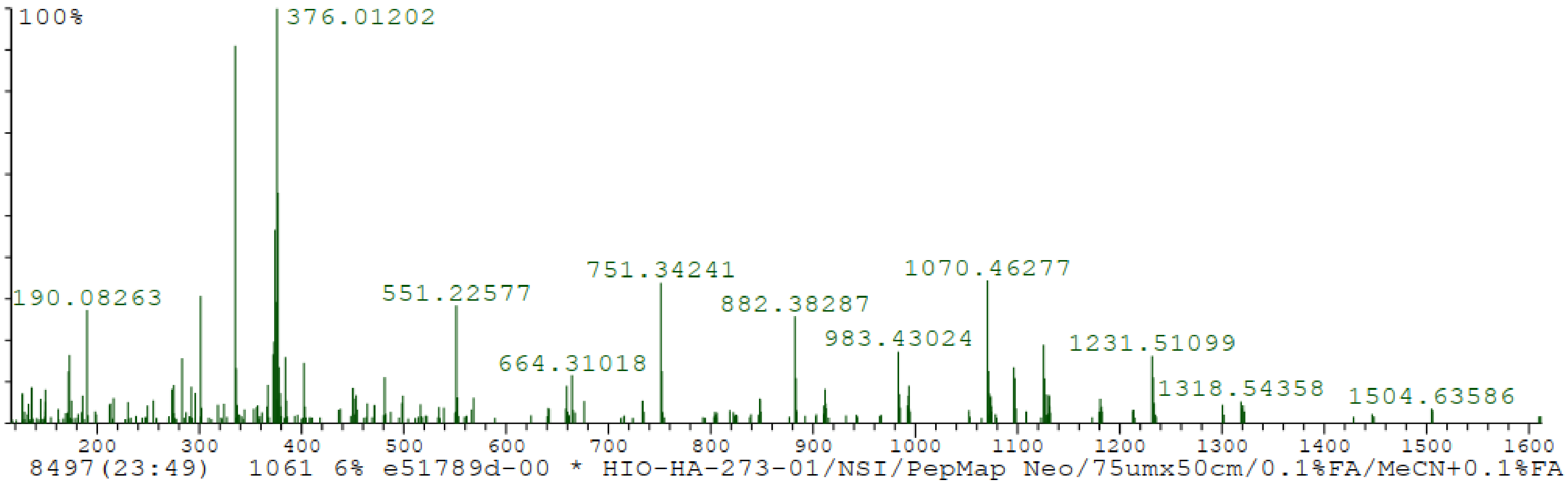

MS/MS of peptide #28 ([M + 2H]3+, m/z 887.64)
Fragmetnation: HCD(30.0%)

ion	theory	exp.	Error [ppm]
(I1)+	129.06585	129.06621	-2.76
(I10)+	136.07569	136.07607	-2.79
(I18)+	133.04301	133.04335	-2.55
(y6)+	751.34031	751.34241	-2.79
(y5)+	664.30829	664.31018	-2.85
(y4)+	551.22422	551.22577	-2.81
(y3)+	450.17654	450.17755	-2.24
(y2)+	335.14960	335.15048	-2.62
(y1)+	175.11895	175.11828	+3.83
(y12-H2O)+	1446.59879	1446.59680	+1.38
(y11-H2O)+	1318.54021	1318.54358	+2.55
(y10-H2O)+	1231.50818	1231.51099	+2.28

a|b|c|I	Seq.	x|y|z
1	N-Acetyl	19
2	G	18
3	Q	17
4	T	16
5	N	15
6	C	14
7	Y-DBSe	13
8	Q	12
9	S	11
10	Y	10
11	S	9
12	T	8
13	M-Oxidation	7
14	S	6
15	I	5
16	T	4
17	D	3
18	C-Carbamidomethyl	2
19	R	1

MS/MS for the peptide 29

10.10.2024 11:57 p.13/26 *** If not stated otherwise, molecular weights refer to the most abundant isotopes of the elements. *** MassLib

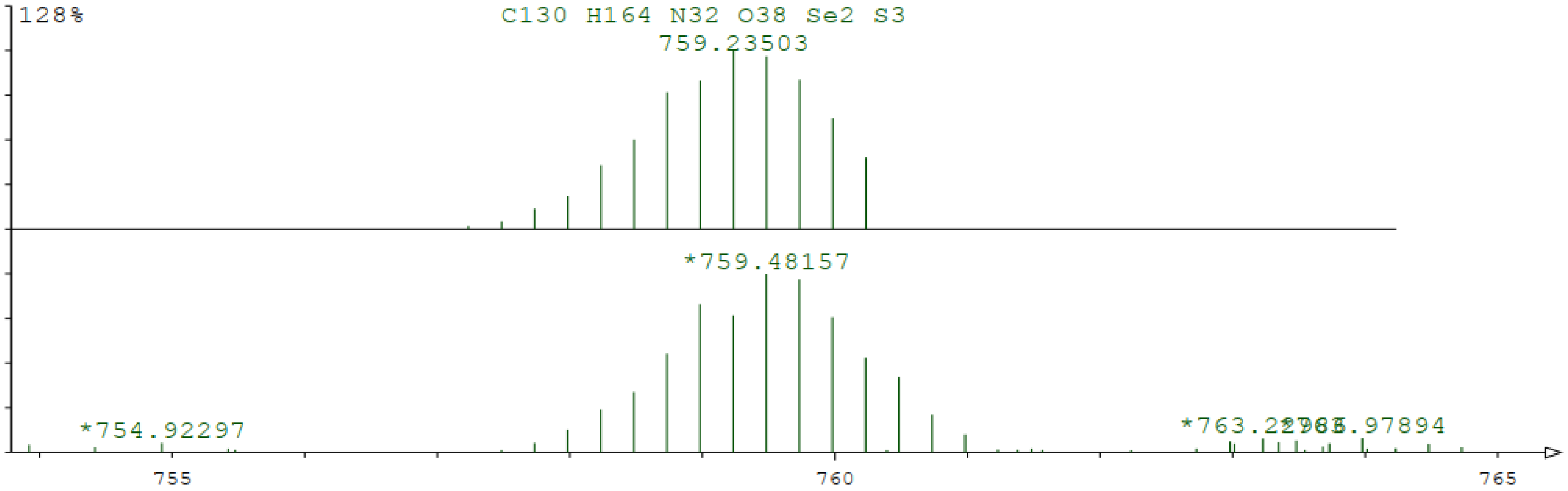

8751(24:24) 1778 4% e51789d-00 * HIO-HA-273-01/NSI/PepMap Neo/75umx50cm/0.1%FA/MeCN+0.1%FA

Mass to be matched (m/z): 759.231930 Charge: 4

Mass Tolerance: ±0.002000

Restriction of atom numbers:

C	H	N	O	Se	S
99-135	99-170	25-35	35-45	2-2	3-3

Number of calculated Formulas: 14

Formula	Diff.(ppm)	theor. m/z
C134 H162 N31 O36 Se2 S3	-0.27	759.231729
C133 H166 N27 O40 Se2 S3	-0.70	759.231395
C132 H160 N34 O35 Se2 S3	-0.71	759.231393
C131 H164 N30 O39 Se2 S3	-1.15	759.231059
C125 H164 N34 O40 Se2 S3	1.23	759.232862
C126 H170 N27 O45 Se2 S3	1.23	759.232864
C130 H168 N26 O43 Se2 S3	-1.59	759.230726
C129 H162 N33 O38 Se2 S3	-1.59	759.230723
C127 H166 N31 O41 Se2 S3	1.67	759.233197
C128 H166 N29 O42 Se2 S3	-2.03	759.230389
C128 H162 N35 O37 Se2 S3	2.11	759.233531
C129 H168 N28 O42 Se2 S3	2.11	759.233534
C126 H164 N32 O41 Se2 S3	-2.47	759.230054
C130 H164 N32 O38 Se2 S3	2.55	759.233867

HRMS for intact modified peptide #29.
Please note that theor. m/z in the table is the value for a fully resolved monoisotopic signal.
Due to small contributions of lesser isotopologue signals that cannot be resolved at finite instrument resolution the "achievable" theoretical value is shifted.
For the given mass resolution this value will be 759.23505, resulting in an error of +4.11 ppm.

As HRMS and isotope pattern do not match well, identification is tentative at most.
Still, the compound does contain a Se-modification, probably twice.

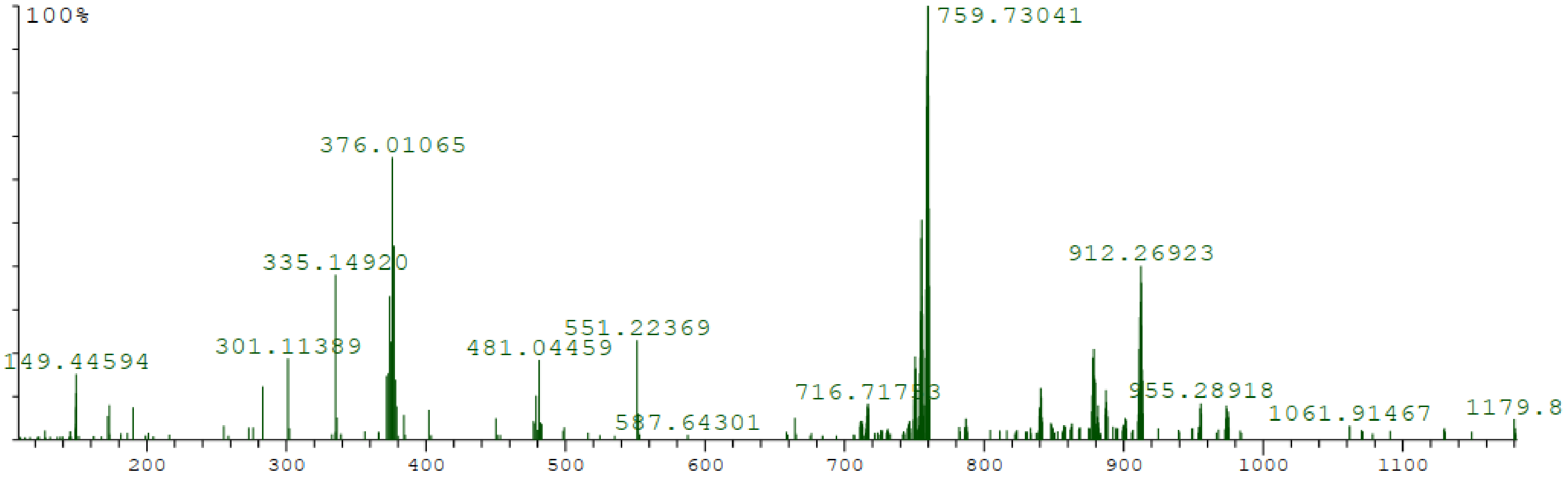

8760(24:25) 209 4% e51789d-00 * HIO-HA-273-01/NSI/PepMap Neo/75umx50cm/0.1%FA/MeCN+0.1%FA/

MS/MS of peptide #29 ([M + 3H]4+, m/z 759.23)
Fragmetnation: HCD(20.0%)

ion	theory	exp.	Error [ppm]
(b2)+	172.07167	172.07130	+2.14
(b3)+	300.13025	300.13016	+0.28
(b17)3+	900.59529	900.59045	+5.38
(y9)+	1070.46051	1070.46436	-3.60
(y8)+	983.42848	983.42773	+0.76
(y7)+	882.38080	882.38153	-0.83
(y6)+	751.34031	751.34271	-3.19
(y5)+	664.30829	664.30743	+1.29
(y4)+	551.22422	551.22424	-0.03
(y3)+	450.17654	450.17599	+1.23
(y2)+	335.14960	335.14932	+0.84
(y10)2+	804.26347	804.26392	-0.56
(y5)2+	332.65778	332.65781	-0.09
(y4)2+	276.11575	276.11581	-0.22
(y17)3+	954.95460	954.95587	-1.33
(y16)3+	912.26841	912.27100	-2.84
(y15)3+	878.58585	878.58728	-1.62
(y14)3+	840.57154	840.57446	-3.47
(y17)4+	716.46777	716.46991	-2.98

a|b|c|I	Seq.	x|y|z
1	N	19
2	G	18
3	Q	17
4	T	16
5	N	15
6	C-Carbamidomethyl	14
7	Y-DBSe	13
8	Q	12
9	S	11
10	Y-DBSe	10
11	S	9
12	T	8
13	M	7
14	S	6
15	I	5
16	T	4
17	D	3
18	C-Carbamidomethyl	2
19	R	1

Human lysozyme-selenonium conjugate 14

HRMS

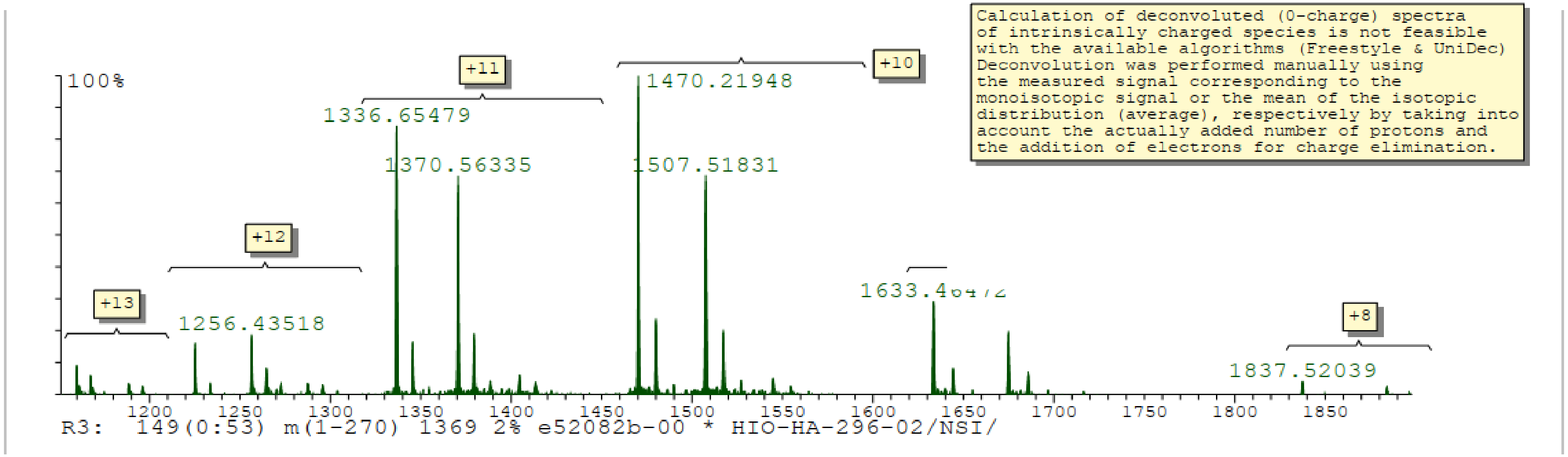

MS analysis of intact protein by direct injection MS.

unmodified:
Deconvoluted mass (0-charge):
Monoisotopic: 14683.10480 (Thermo Freestyle)
Average: 14692.15039 (Unidec)

Theoretical mass (Thermo Freestyle):
Monoisotopic: 14683.08945
Average: 14692.50666 (R=100)

modified with 1 x C20H11N2OSe
Deconvoluted mass (0-charge) calculated based on charge 8-12:
Monoisotopic: 15058.09028
Average: 15065.94353

Theoretical mass (Thermo Freestyle):
Monoisotopic: 15058.09311
Average: 15066.78043 (R=100)

modified with 2 x C20H11N2OSe
Deconvoluted mass (0-charge) calculated based on charge 9-12:
Monoisotopic: 15433.17550
Average: 15441.12988

Theoretical mass (Thermo Freestyle):
Monoisotopic: 15433.09677
Average: 15441.04894 (R=100)

LC-FTMS of 14 after trypsin digestion

```
HPLC-MS/MS of modified human Lysozyme, tryptic digest.
Interpretation done using Thermo Proteome Discoverer 3.1

For further details on modified peptides see below.
```

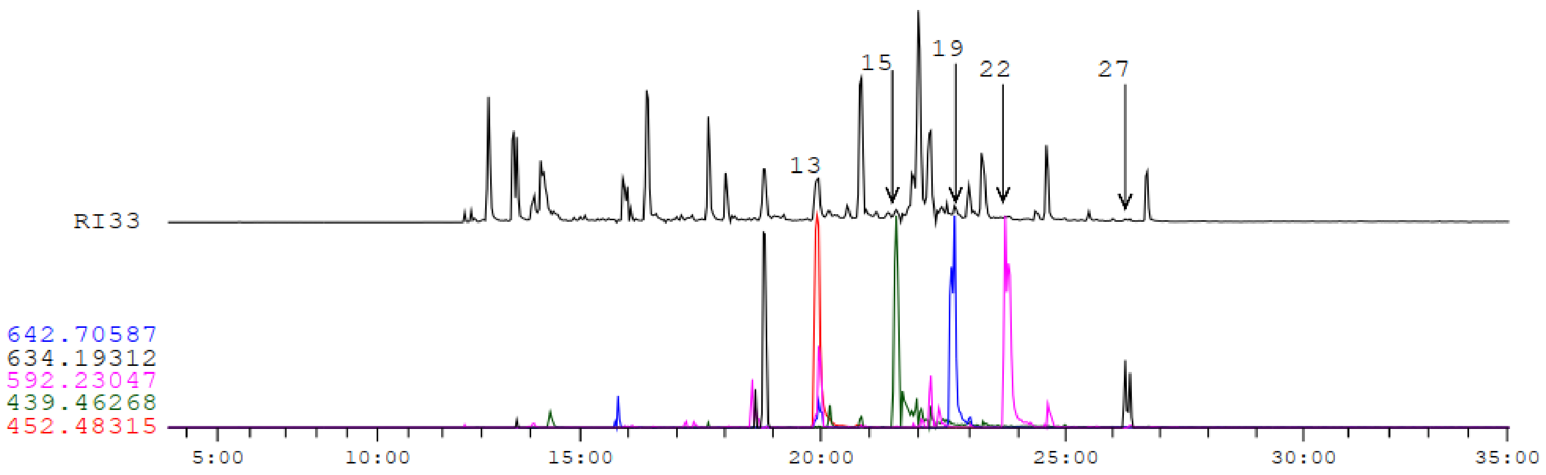

peptides identified from Lysozyme (human)

#	RT/min	Sequence	Modifications	m/z	Abundance	XCorr	
1	13.58	[K].RVVRDPQGIR.[A]	-	399.23908	9.55E+07	4.12	
2	13.65	[R].ATNYNAGDR.[S]	-	491.22312	2.07E+10	3.47	
3	13.73	[R].CELAR.[T]	1×Carbamidomethyl [C1]	324.66034	1.09E+10	2.12	
4	14.07	[R].VVRDPQGIR.[A]	-	347.20532	3.85E+09	3.53	
5	14.08	[K].RLGMDGYR.[G]	1×Oxidation [M4]	328.49570	7.87E+08	2.68	
6	14.24	[R].DPQGIR.[A]	-	343.18502	2.20E+10	2.23	
7	15.08	[R].LGMDGYR.[G]	1×Oxidation [M3]	414.18973	1.61E+09	2.05	
8	15.93	[K].RLGMDGYR.[G]	-				
9	15.99	[R].YWCNDGK.[T]	1×Carbamidomethyl [C3]	471.69279	4.38E+09	1.97	
10	16.38	[K].WESGYNTR.[A]	-	506.72778	2.70E+10	2.40	
11	17.65	[R].LGMDGYR.[G]	-	406.19223	2.48E+10	2.15	
12	18.02	[R].QYVQGCGV.[-]	1×Carbamidomethyl [C6]	455.70811	1.24E+09	3.70	
13	19.93	[R].ATNYNAGDR.[S]	1×DBSe [Y4]	452.48314	8.86E+09	3.73	<--
14	20.83	[R].AWVAWR.[N]	-	394.71339	2.84E+10	2.16	
15	21.54	[R].YWCNDGK.[T]	1×DBSe [Y1]<br>1×Carbamidomethyl [C3]	439.46267	1.57E+09	2.56	<--
16	21.88	[K].TPGAVNACHLSCSALLQDNIADAVACAK.[R]	3×Carbamidomethyl [C8; C12; C26]	976.46418	4.01E+09	9.74	
17	22.01	[R].STDYGIFQINSR.[Y]	-	700.84343	3.48E+10	5.00	
18	22.43	[K].TPGAVNACHLSCSALLQDNIADAVACAK.[R]	2×Carbamidomethyl [C8; C12]	957.45662	5.33E+08	8.63	
19	22.66	[R].QYVQGCGV.[-]	1×DBSe [Y2]<br>1×Carbamidomethyl [C6]	642.70588	5.34E+08	3.03	<--
20	22.69	[R].GISLANWMCLAK.[W]	1×Oxidation [M8]<br>1×Carbamidomethyl [C9]	690.34417	7.96E+08	4.91	
21	22.98	[R].GISLANWMCLAK.[W]	1×Oxidation [M8]	661.83350	1.06E+07	2.85	
22	23.73	[R].STDYGIFQINSR.[Y]	1×DBSe [Y4]	592.23049	6.36E+08	5.09	<--
23	24.31	[R].GISLANWMCLAKWESGYNTR.[A]	1×Oxidation [M8]<br>1×Carbamidomethyl [C9]	791.70894	8.05E+07	4.36	
24	24.38	[R].QYVQGCGV.[-]	1×Gln->pyro-Glu [N-Term]<br>1×Carbamidomethyl [C6]	447.19463	2.86E+08	2.93	
25	24.60	[R].GISLANWMCLAK.[W]	1×Carbamidomethyl [C9]	682.34661	1.11E+10	4.81	
26	25.01	[R].GISLANWMCLAK.[W]	-	653.83893	2.59E+07	2.96	
27	26.26	[R].QYVQGCGV.[-]	1×Gln->pyro-Glu [N-Term]<br>1×DBSe [Y2]<br>1×Carbamidomethyl [C6]	634.19312	1.77E+08	3.05	<--

MS/MS for the peptide 13

7.10.2024 12:09 p.2/12 *** If not stated otherwise, molecular weights refer to the most abundant isotopes of the elements. *** MassLib

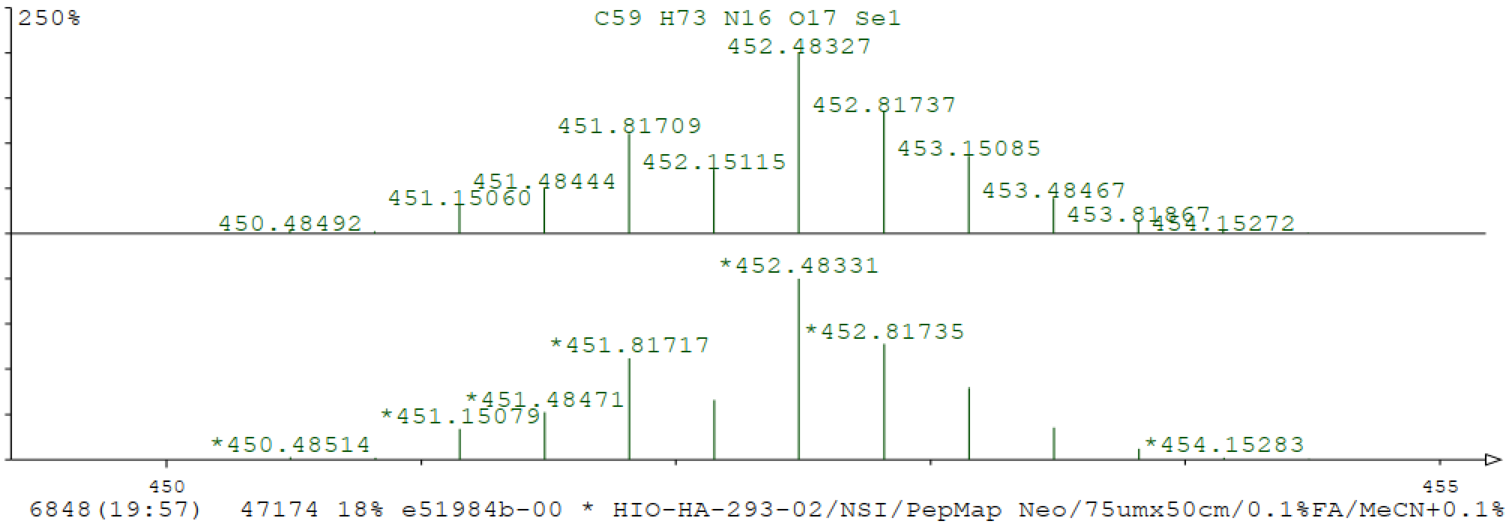

Mass to be matched (m/z): 452.483310 Charge: 3

Mass Tolerance: ±0.001000
Restriction of atom numbers:

C	H	N	O	Se	S
55-65	60-80	10-20	13-23	1-1	max 2

Number of calculated Formulas: 12

Formula	Diff.(ppm)	theor. m/z	
C60 H69 N20 O13 Se1	0.17	452.483385	
C61 H75 N13 O18 Se1	0.17	452.483388	
C62 H79 N13 O13 Se1 S2	0.81	452.483678	
C59 H73 N16 O17 Se1	-0.82	452.482940	<--
C62 H71 N17 O14 Se1	1.16	452.483833	
C63 H77 N10 O19 Se1	1.16	452.483836	
C56 H77 N16 O17 Se1 S1	1.67	452.484064	
C65 H75 N13 O13 Se1 S1	-1.67	452.482554	
C58 H77 N12 O21 Se1	-1.80	452.482495	
C57 H71 N19 O16 Se1	-1.81	452.482492	
C64 H73 N14 O15 Se1	2.15	452.484281	
C57 H79 N15 O15 Se1 S2	-2.15	452.482337	

HRMS for intact modified peptide #13.
Please note that theor. m/z in the table is the value for a fully resolved monoisotopic signal.
Due to small contributions of lesser isotopologue signals that cannot be resolved at finite instrument resolution the "achievable" theoretical value is shifted.
For the given mass resolution this value will be 452.48325, resulting in an error of -0.13 ppm.

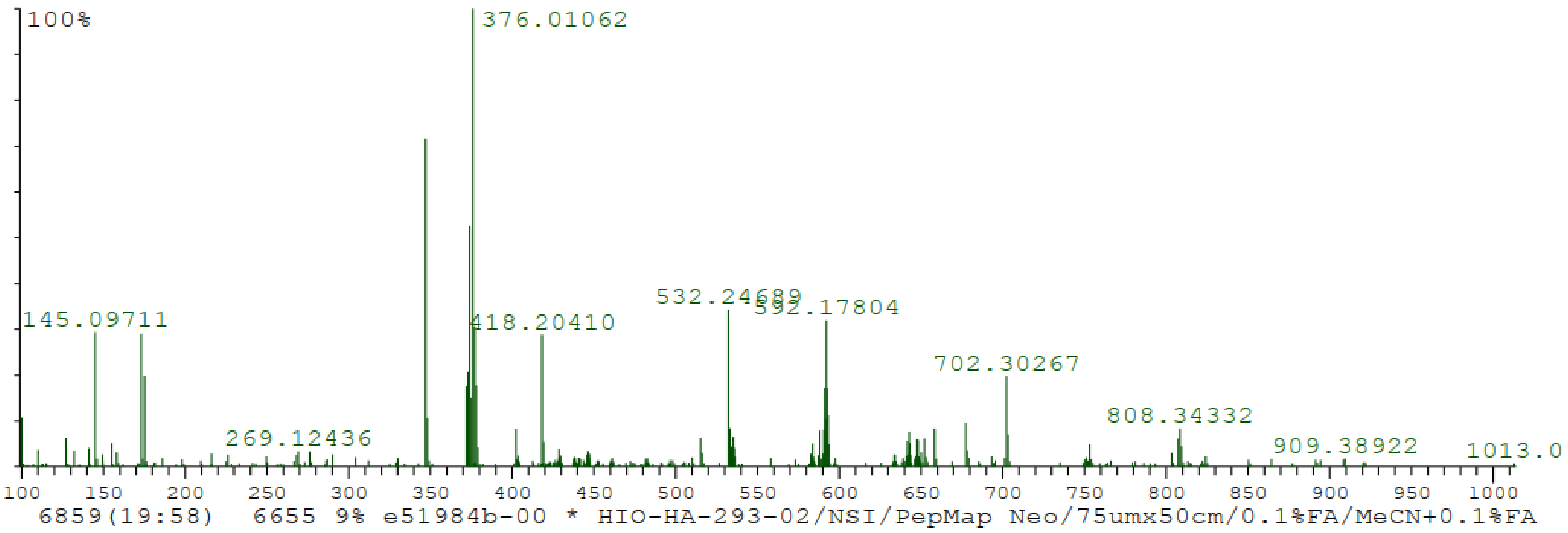

ion	theory	exp.	Error [ppm]
(b2)+	173.09207	173.09195	+0.69
(b3)+	287.13500	287.13449	+1.76
(b4)+	824.19415	824.19177	+2.89
(b4)2+	412.60072	412.60083	-0.28
(b5)2+	469.62218	469.62265	-1.00
(b6)2+	505.14074	505.13849	+4.45
(b7)2+	533.65147	533.65649	-9.41
(I4)+	510.07152	510.07028	+2.43
(y5)+	532.24740	532.24689	+0.96
(y4)+	418.20447	418.20410	+0.89
(y3)+	347.16736	347.16699	+1.06
(y2)+	290.14590	290.14566	+0.81
(y1)+	175.11895	175.11884	+0.64
(y8)2+	642.70222	642.70197	+0.39
(y7)2+	592.17838	592.17804	+0.58
(y6)2+	535.15692	535.15692	+0.00
(y5)2+	266.62734	266.62708	+0.97
(y4)2+	209.60587	209.60545	+2.03
(y8)3+	428.80391	428.80368	+0.53

a|b|c|I	Seq.	x|y|z
1	A	9
2	T	8
3	N	7
4	Y-DBSe	6
5	N	5
6	A	4
7	G	3
8	D	2
9	R	1

MS/MS of peptide #13 ([M + 2H]3+, m/z 452.48

MS/MS for the peptide 15

7.10.2024 12:09 p.3/12 *** If not stated otherwise, molecular weights refer to the most abundant isotopes of the elements. *** MassLib

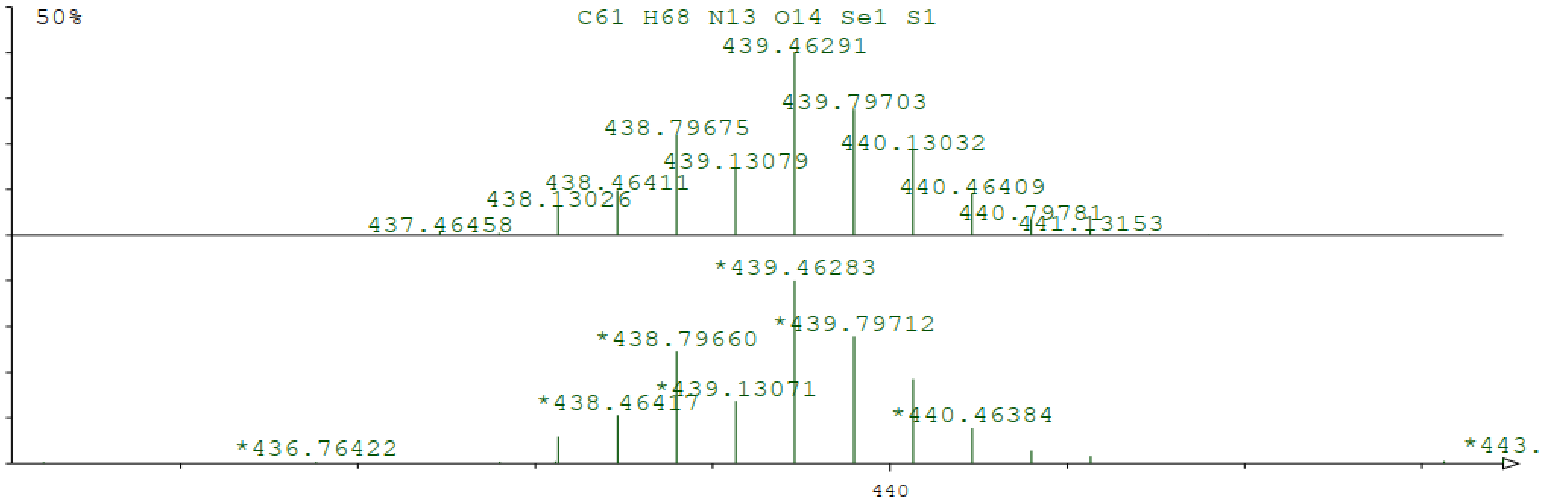

7601(21:40) 8755 9% e51984b-00 * HIO-HA-293-02/NSI/PepMap Neo/75umx50cm/0.1%FA/MeCN+0.1%FA

Mass to be matched (m/z): 439.462830 Charge: 3

Mass Tolerance: ±0.001000
Restriction of atom numbers:

C	H	N	O	Se	S
55-65	60-80	10-20	10-20	1-1	max 2

Number of calculated Formulas: 12

Formula	Diff.(ppm)	theor. m/z	
C55 H74 N12 O17 Se1 S2	0.00	439.462832	
C55 H66 N16 O18 Se1	0.36	439.462987	
C62 H64 N17 O10 Se1 S1	0.49	439.463046	
C63 H70 N10 O15 Se1 S1	0.50	439.463049	
C61 H68 N13 O14 Se1 S1	-0.52	439.462601	<--
C56 H70 N16 O13 Se1 S2	1.02	439.463277	
C56 H62 N20 O14 Se1	1.37	439.463432	
C57 H68 N13 O19 Se1	1.38	439.463435	
C64 H66 N14 O11 Se1 S1	1.51	439.463494	
C59 H66 N16 O13 Se1 S1	-1.54	439.462153	
C58 H72 N13 O14 Se1 S2	2.04	439.463725	
C65 H60 N17 O10 Se1	-2.07	439.461922	

HRMS for intact modified peptide #15.
Please note that theor. m/z in the table is the value for a fully resolved monoisotopic signal.
Due to small contributions of lesser isotopologue signals that cannot be resolved at finite instrument resolution the "achievable" theoretical value is shifted.
For the given mass resolution this value will be 439.46288, resulting in an error of +0.11 ppm.

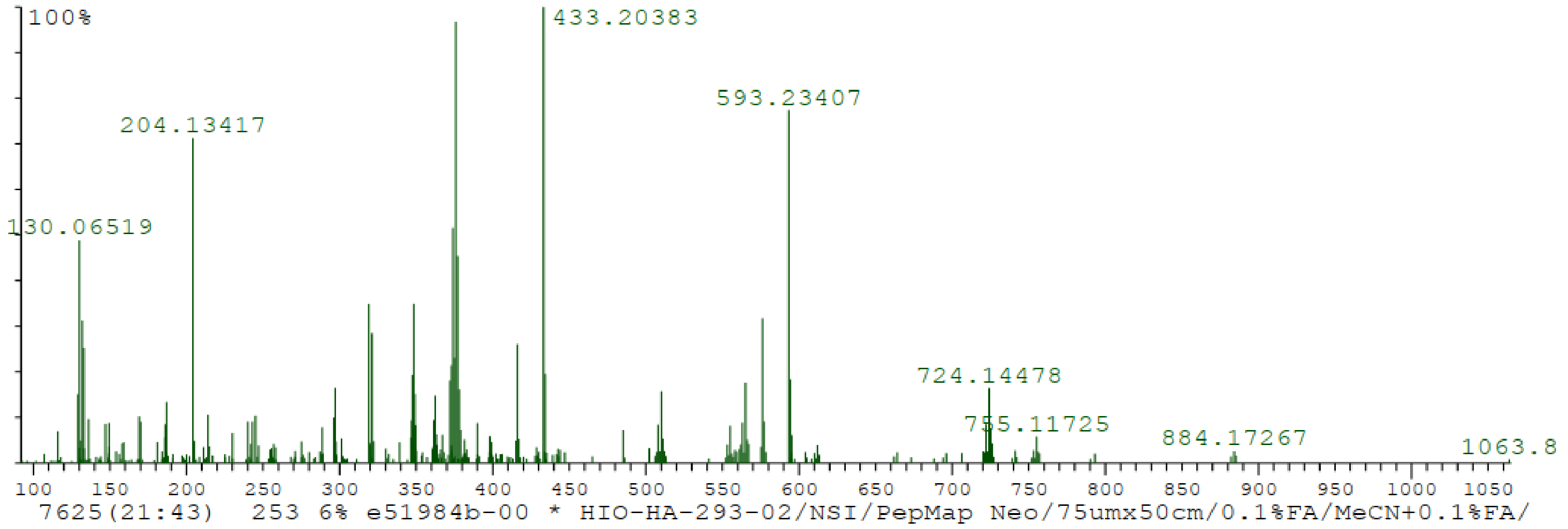

ion	theory	exp.	Error [ppm]
(b2)+	724.14575	724.14478	+1.34
(b3)+	884.17640	884.17267	+4.21
(b2)2+	362.57651	362.57648	+0.09
(I1)+	510.07152	510.07126	+0.51
(I2)+	159.09167	159.09174	-0.41
(I3)+	133.04301	133.04311	-0.75
(y5)+	593.23479	593.23407	+1.21
(y4)+	433.20414	433.20383	+0.71
(y3)+	319.16121	319.16107	+0.44
(y2)+	204.13427	204.13417	+0.48
(y1)+	147.11280	147.11287	-0.45
(y6)2+	390.16069	390.16049	+0.51
(y5)2+	297.12103	297.12103	+0.01
(y4)2+	217.10571	217.10587	-0.75

MS/MS of peptide #15 ([M + 2H]3+, m/z 439.46)
Fragmetnation: HCD(20.0%)

a|b|c|I	Seq.	x|y|z
1	Y-DBSe	7
2	W	6
3	C-Carbamidomethyl	5
4	N	4
5	D	3
6	G	2
7	K	1

MS/MS for the peptide 19

7.10.2024 12:09 p.4/12 *** If not stated otherwise, molecular weights refer to the most abundant isotopes of the elements. *** MassLib

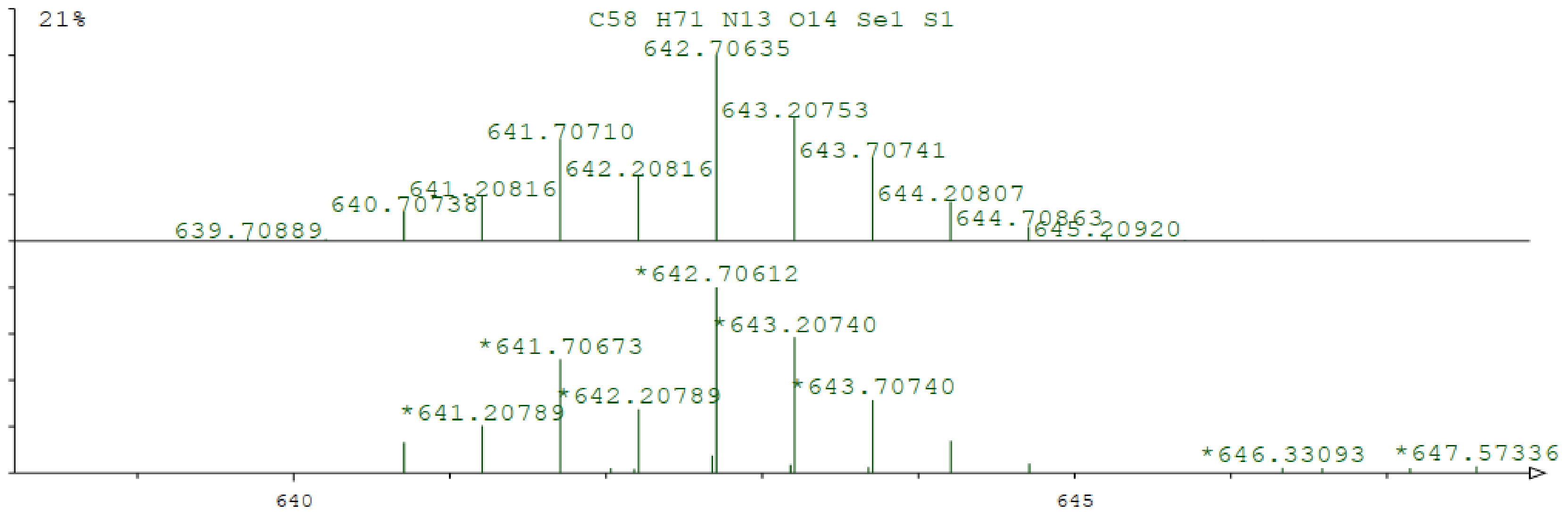

8124(22:47) 5710 6% e51984b-00 * HIO-HA-293-02/NSI/PepMap Neo/75umx50cm/0.1%FA/MeCN+0.1%FA

Mass to be matched (m/z): 642.706120 Charge: 2

Mass Tolerance: ±0.001000

Restriction of atom numbers:

C	H	N	O	Se	S
55-65	60-80	10-20	10-20	1-1	max 2

Number of calculated Formulas: 6

Formula	Diff.(ppm)	theor. m/z	
C58 H71 N13 O14 Se1 S1	-0.32	642.705914	<--
C59 H67 N17 O10 Se1 S1	0.72	642.706581	
C60 H73 N10 O15 Se1 S1	0.72	642.706585	
C64 H65 N14 O11 Se1	-0.86	642.705567	
C64 H73 N10 O10 Se1 S2	-1.22	642.705334	
C56 H69 N16 O13 Se1 S1	-1.37	642.705242	

HRMS for intact modified peptide #19.
Please note that theor. m/z in the table is the value for a fully resolved monoisotopic signal.
Due to small contributions of lesser isotopologue signals that cannot be resolved at finite instrument resolution the "achievable" theoretical value is shifted.
For the given mass resolution this value will be 642.70632, resulting in an error of +0.31 ppm.

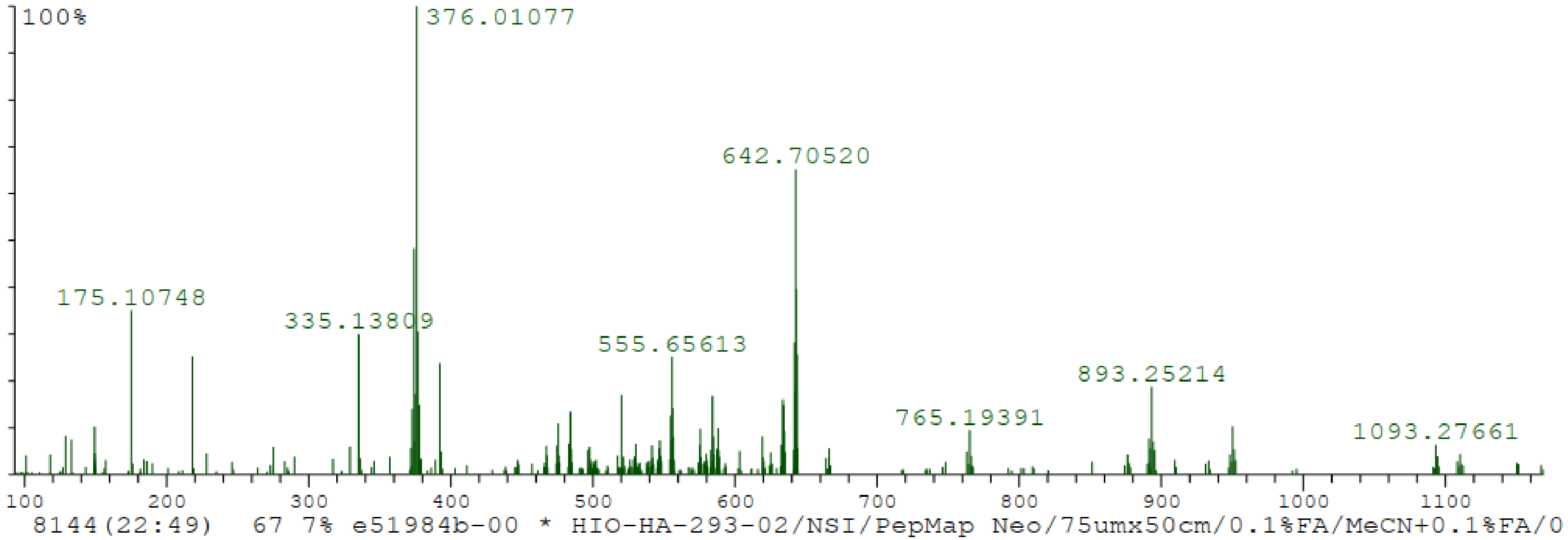

MS/MS of peptide #19 ([M + H]2+, m/z 642.71

ion	theory	exp.	Error [ppm]
(b2)+	666.12501	666.12592	-1.36
(b3)+	765.19343	765.19391	-0.63
(b4)+	893.25200	893.25214	-0.15
(b5)+	950.27347	950.27509	-1.71
(b6)+	1110.30412	1110.30591	-1.62
(b7)+	1167.32558	1167.32776	-1.87
(b4)2+	447.12964	447.12979	-0.33
(b5)2+	475.64037	475.64056	-0.40
(b6)2+	555.65570	555.65613	-0.78
(b7)2+	584.16643	584.16626	+0.29
(I1)+	101.07094	101.07050	+4.37
(I4)+	101.07094	101.07050	+4.37
(I6)+	133.04301	133.04295	+0.45
(y6)+	619.28682	619.28662	+0.33
(y5)+	520.21841	520.21826	+0.29
(y4)+	392.15983	392.15952	+0.79
(y3)+	335.13837	335.13809	+0.83
(y2)+	175.10772	175.10748	+1.36

a|b|c|I	Seq.	x|y|z
1	Q	8
2	Y-DBSe	7
3	V	6
4	Q	5
5	G	4
6	C-Carbamidomethyl	3
7	G	2
8	V	1

MS/MS for the peptide 22

7.10.2024 12:09 p.5/12 *** If not stated otherwise, molecular weights refer to the most abundant isotopes of the elements. ***

MassLib

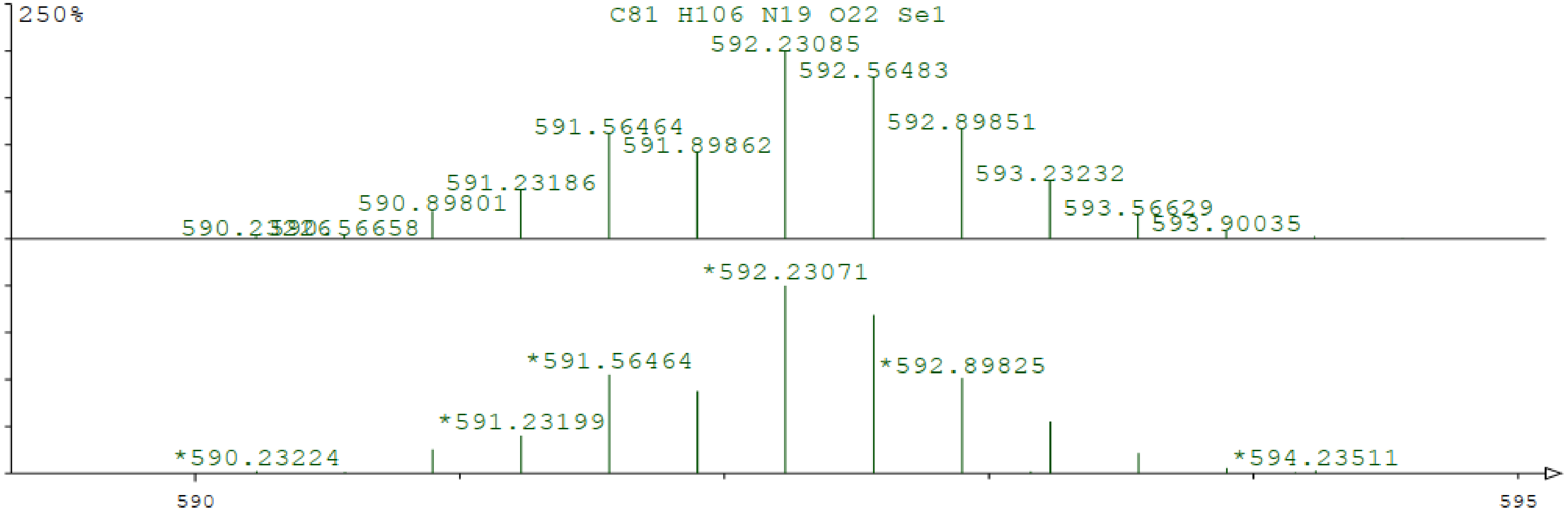

8593(23:50) 2437 6% e51984b-00 * HIO-HA-293-02/NSI/PepMap Neo/75umx50cm/0.1%FA/MeCN+0.1%FA

Mass to be matched (m/z): 592.230710 Charge: 3

Mass Tolerance: ±0.001000

Restriction of atom numbers:

C	H	N	O	Se
70-90	95-115	15-25	15-25	1-1

Number of calculated Formulas: 8

Formula	Diff.(ppm)	theor. m/z	
C82 H102 N23 O18 Se1	0.03	592.230726	
C83 H108 N16 O23 Se1	0.03	592.230729	
C81 H106 N19 O22 Se1	-0.73	592.230280	<--
C84 H104 N20 O19 Se1	0.78	592.231173	
C70 H106 N25 O25 Se1	1.07	592.231343	
C79 H104 N22 O21 Se1	-1.48	592.229833	
C85 H100 N24 O15 Se1	1.53	592.231618	
C86 H106 N17 O20 Se1	1.54	592.231621	

HRMS for intact modified peptide #22.
Please note that theor. m/z in the table is the value for a fully resolved monoisotopic signal.
Due to small contributions of lesser isotopologue signals that cannot be resolved at finite instrument resolution the "achievable" theoretical value is shifted.
For the given mass resolution this value will be 592.23083, resulting in an error of +0.20 ppm.

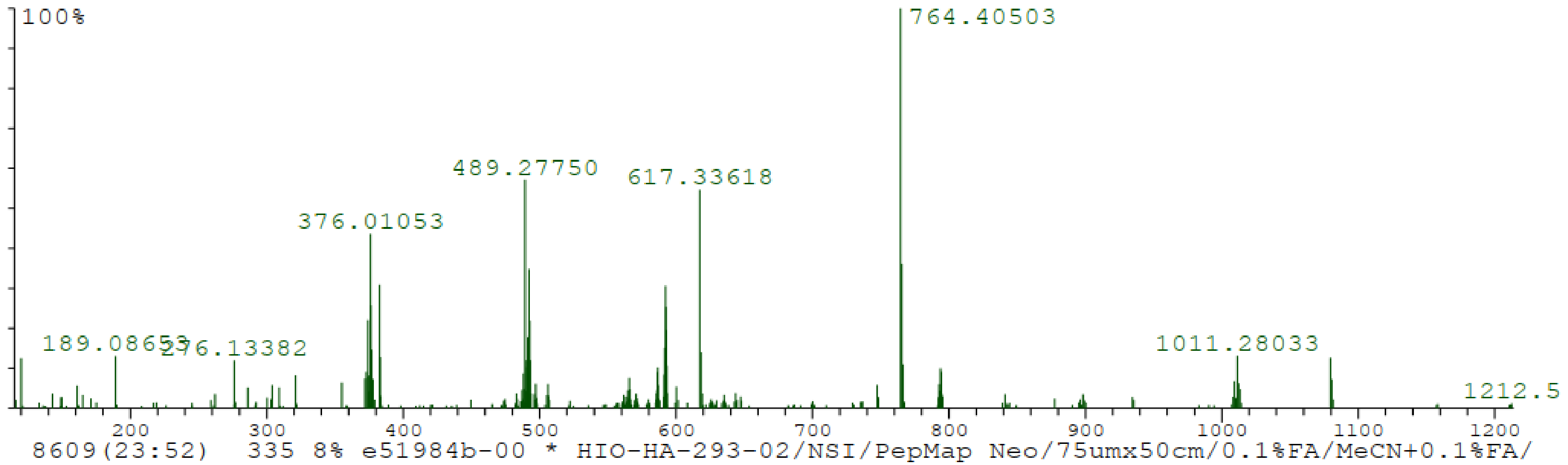

ion	theory	exp.	Error [ppm]
(b2)+	189.08698	189.08605	+4.91
(b3)+	304.11393	304.11340	+1.73
(b4)+	841.17308	841.17413	-1.24
(b5)+	898.19455	898.19604	-1.66
(b6)+	1011.27861	1011.28033	-1.70
(b7)+	1158.34703	1158.34875	-1.49
(b4)2+	421.09018	421.08829	+4.49
(b5)2+	449.60091	449.60184	-2.06
(b6)2+	506.14294	506.14337	-0.84
(b7)2+	579.67715	579.67932	-3.74
(b8)2+	643.70644	643.70667	-0.36
(I7)+	120.08078	120.08047	+2.55
(y8)+	934.51049	934.51263	-2.29
(y7)+	877.48903	877.48785	+1.34
(y6)+	764.40496	764.40503	-0.09
(y5)+	617.33655	617.33618	+0.60
(y4)+	489.27797	489.27750	+0.96
(y3)+	376.19391	376.19345	+1.22
(y2)+	262.15098	262.15057	+1.57
(y1)+	175.11895	175.11902	-0.39
(y11)2+	844.32577	844.32837	-3.08
(y10)2+	793.80193	793.80267	-0.93
(y7)2+	439.24815	439.24753	+1.42
(y6)2+	382.70612	382.70566	+1.20
(y5)2+	309.1719		

a|b|c|I	Seq.	x|y|z
1	S	12
2	T	11
3	D	10
4	Y-DBSe	9
5	G	8
6	I	7
7	F	6
8	Q	5
9	I	4
10	N	3
11	S	2
12	R	1

MS/MS of peptide #22 ([M + 2H]3+, m/z 592.23)
Fragmetnation: HCD(20.0%)

MS/MS for the peptide 27

7.10.2024 12:09 p.6/12 *** If not stated otherwise, molecular weights refer to the most abundant isotopes of the elements. *** MassLib

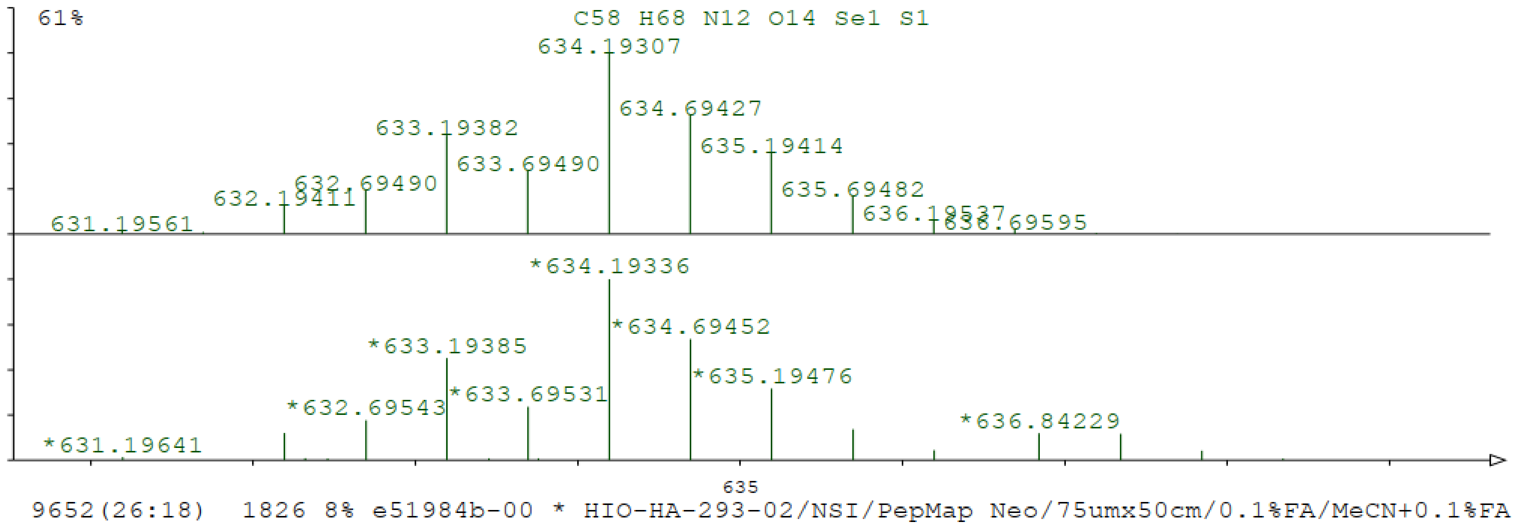

Mass to be matched (m/z): 634.193360 Charge: 2

Mass Tolerance: ±0.001000
Restriction of atom numbers:

C	H	N	O	Se	S
50-70	60-80	7-17	10-20	1-1	max 2

Number of calculated Formulas: 12

Formula	Diff.(ppm)	theor. m/z
C60 H70 N9 O15 Se1 S1	-0.08	634.193311
C59 H64 N16 O10 Se1 S1	-0.08	634.193307
C52 H66 N15 O18 Se1	-0.22	634.193218
C68 H66 N7 O13 Se1	0.44	634.193636
C53 H70 N15 O13 Se1 S2	0.46	634.193654
C54 H76 N8 O18 Se1 S2	0.47	634.193658
C52 H74 N11 O17 Se1 S2	-0.59	634.192986
C66 H64 N10 O12 Se1	-0.62	634.192964
C54 H68 N12 O19 Se1	0.84	634.193890
C61 H66 N13 O11 Se1 S1	0.98	634.193978
C58 H68 N12 O14 Se1 S1	-1.14	634.192639 <--
C55 H72 N12 O14 Se1 S2	1.52	634.194326

HRMS for intact modified peptide #27.
Please note that theor. m/z in the table is the value for a fully resolved monoisotopic signal.
Due to small contributions of lesser isotopologue signals that cannot be resolved at finite instrument resolution the "achievable" theoretical value is shifted.
For the given mass resolution this value will be 634.19301, resulting in an error of -0.55 ppm.

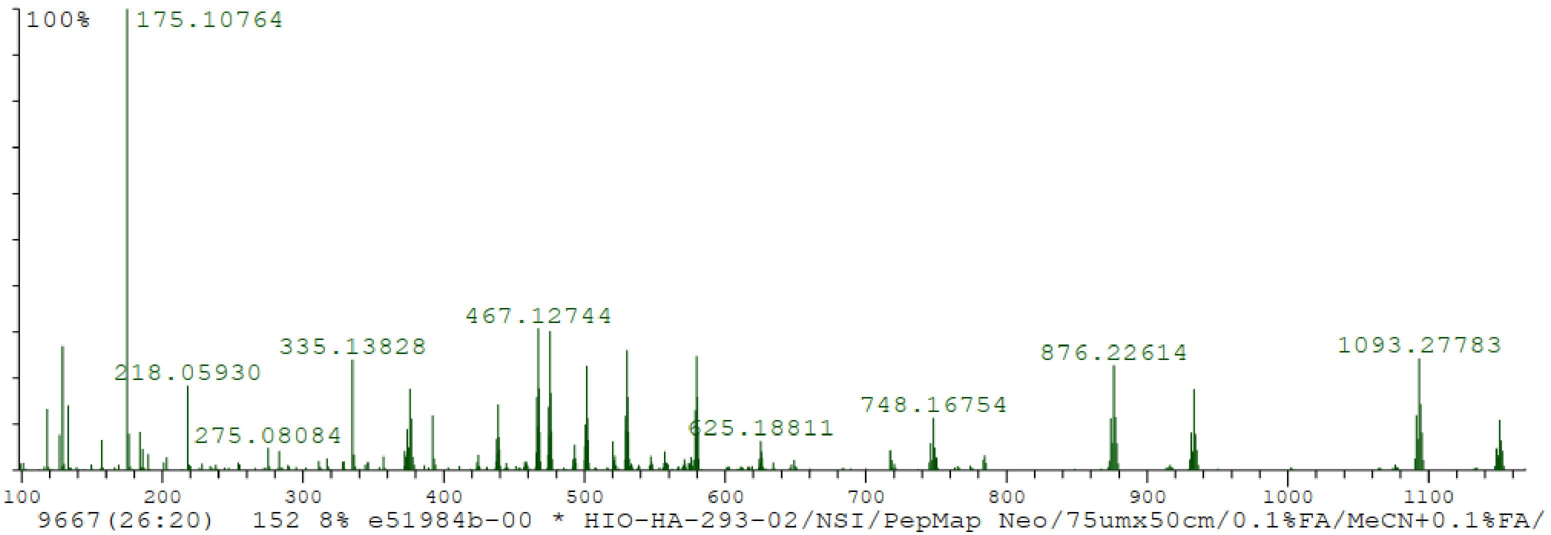

MS/MS of peptide #27 ([M + H]2+, m/z 634.19)

ion	theory	exp.	Error [ppm]
(b2)+	649.09846	649.09924	-1.20
(b3)+	748.16688	748.16754	-0.89
(b4)+	876.22545	876.22614	-0.78
(b5)+	933.24692	933.24738	-0.49
(b6)+	1093.27757	1093.27783	-0.24
(b7)+	1150.29903	1150.29871	+0.28
(b4)2+	438.61637	438.61688	-1.17
(b5)2+	467.12710	467.12744	-0.73
(b6)2+	547.14242	547.14246	-0.07
(b7)2+	575.65315	575.65338	-0.39
(I4)+	101.07094	101.07076	+1.80
(I6)+	133.04301	133.04305	-0.30
(y6)+	619.28682	619.28687	-0.08
(y5)+	520.21841	520.21844	-0.06
(y4)+	392.15983	392.15982	+0.03
(y3)+	335.13837	335.13828	+0.26
(y2)+	175.10772	175.10764	+0.45
(y1)+	118.08626	118.08629	-0.30

a|b|c|I	Seq.	x|y|z
1	Q-Gln->pyro-Glu	8
2	Y-DBSe	7
3	V	6
4	Q	5
5	G	4
6	C-Carbamidomethyl	3
7	G	2
8	V	1

Bromo-bivalirudin 23

LC/Q-TOF-MS/MS

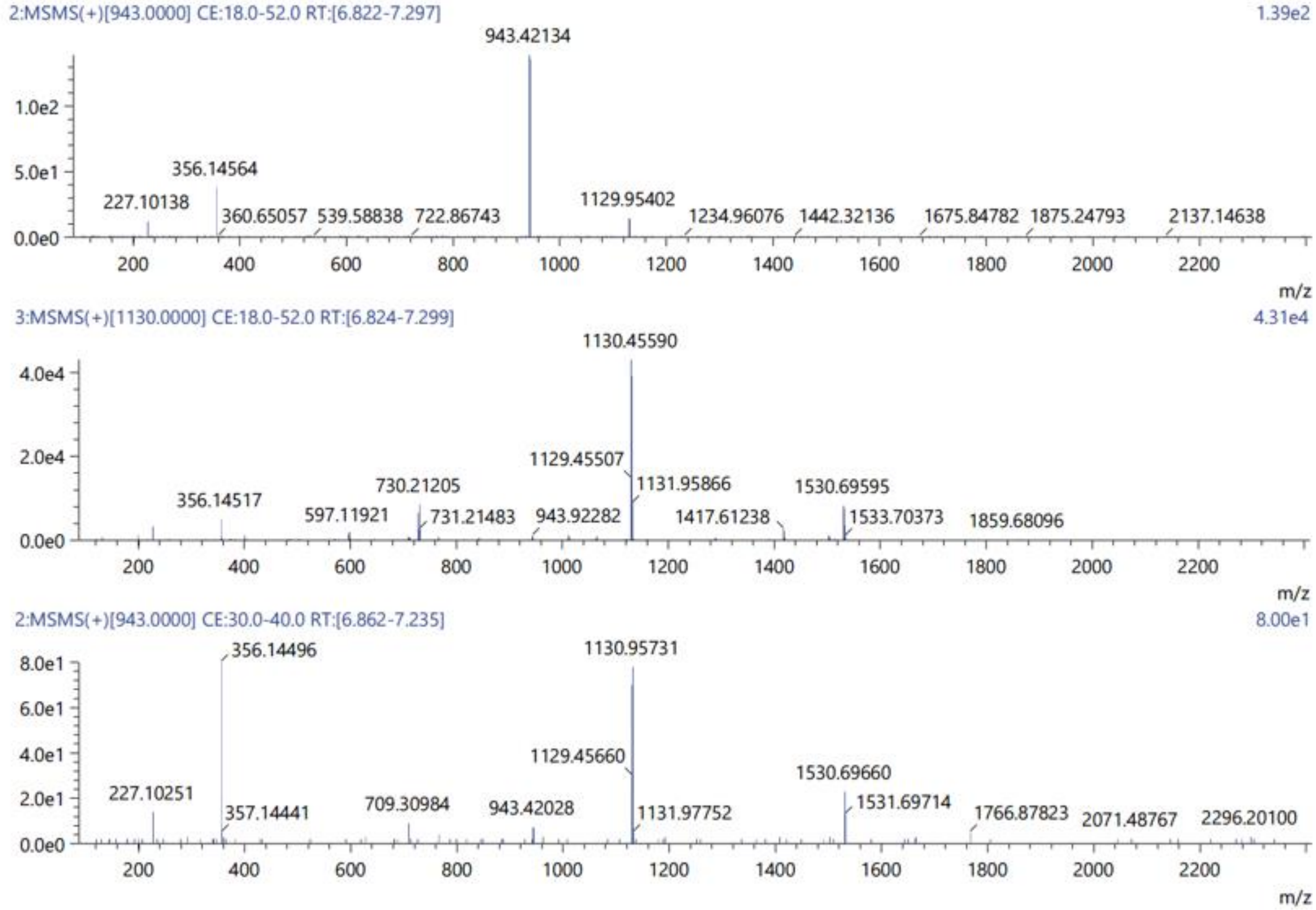

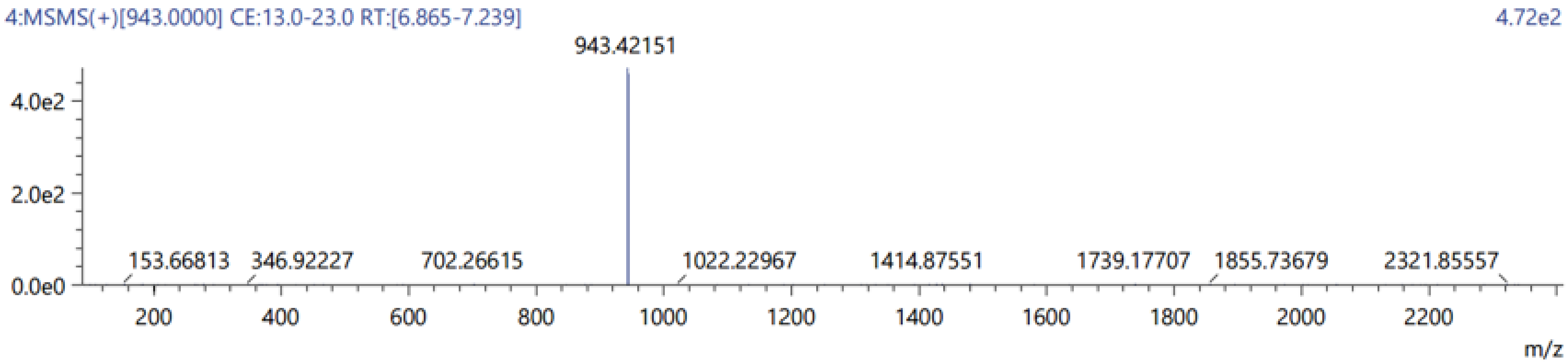
4:MSMS(+)[943.0000] CE:13.0-23.0 RT:[6.865-7.239]
4.72e2
943.42151
4.0e2
2.0e2
0.0e0
153.66813
346.92227
702.26615
1022.22967
1414.87551
1739.17707
1855.73679
2321.85557
200
400
600
800
1000
1200
1400
1600
1800
2000
2200
m/z

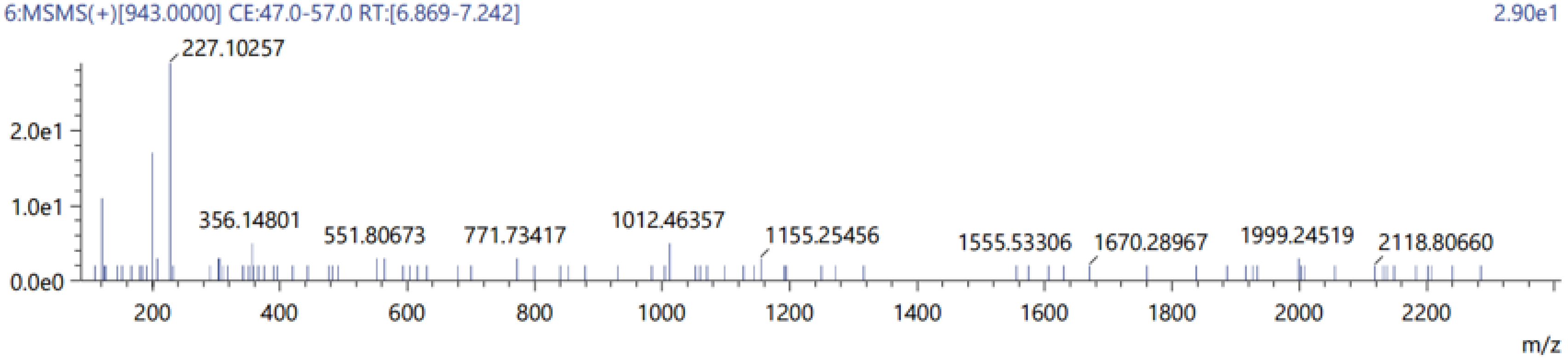
6:MSMS(+)[943.0000] CE:47.0-57.0 RT:[6.869-7.242]
2.90e1
227.10257
2.0e1
1.0e1
0.0e0
356.14801
551.80673
771.73417
1012.46357
1155.25456
1555.53306
1670.28967
1999.24519
2118.80660
200
400
600
800
1000
1200
1400
1600
1800
2000
2200
m/z

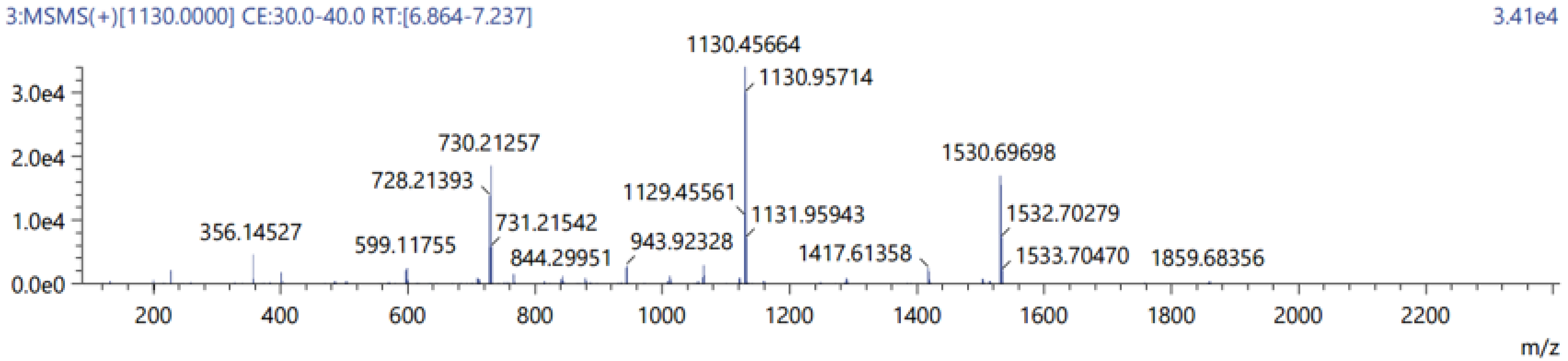
3:MSMS(+)[1130.0000] CE:30.0-40.0 RT:[6.864-7.237]
3.41e4
1130.45664
1130.95714
3.0e4
2.0e4
1.0e4
0.0e0
730.21257
728.21393
731.21542
356.14527
599.11755
844.29951
1129.45561
943.92328
1131.95943
1417.61358
1530.69698
1532.70279
1533.70470
1859.68356
200
400
600
800
1000
1200
1400
1600
1800
2000
2200
m/z

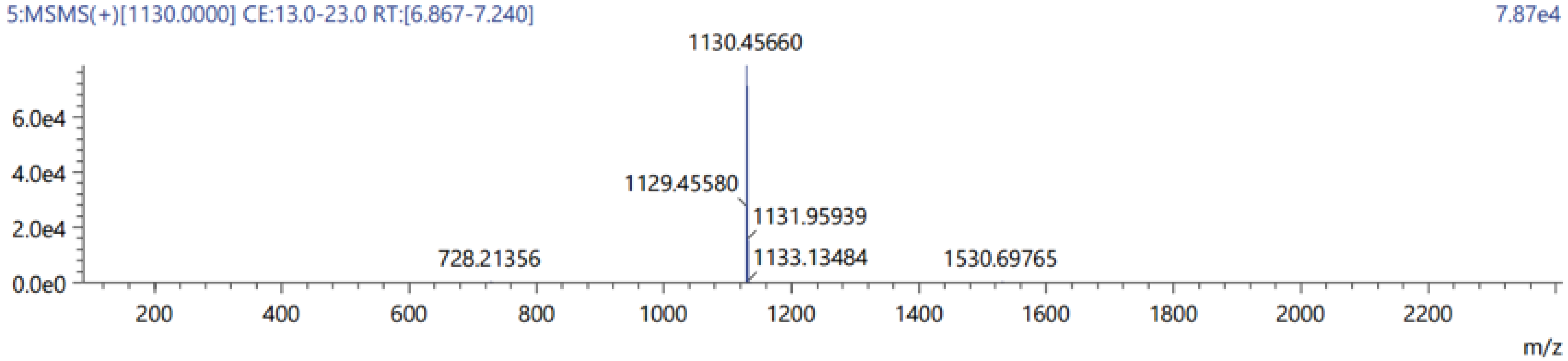
5:MSMS(+)[1130.0000] CE:13.0-23.0 RT:[6.867-7.240]
7.87e4
1130.45660
1129.45580
1131.95939
1133.13484
728.21356
1530.69765
6.0e4
4.0e4
2.0e4
0.0e0
200
400
600
800
1000
1200
1400
1600
1800
2000
2200
m/z

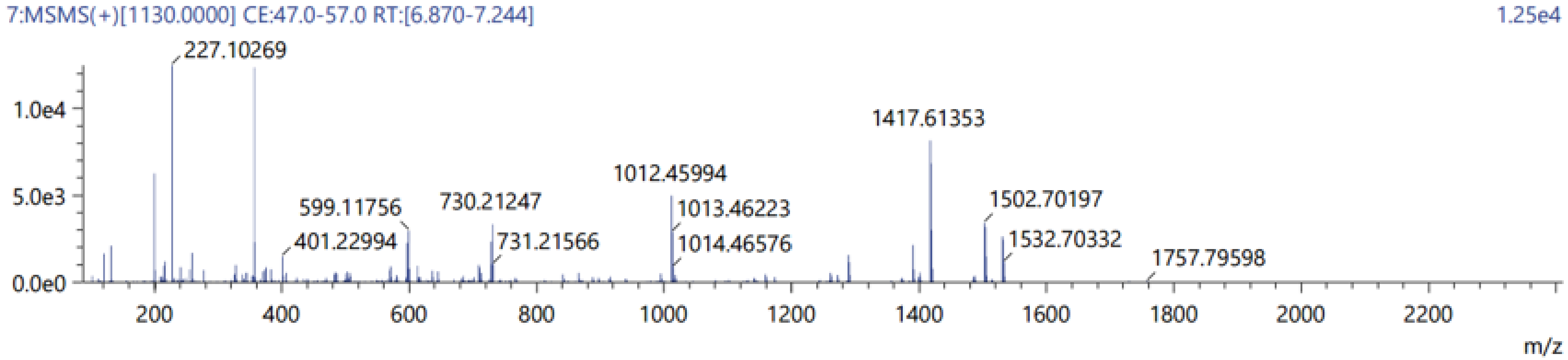
7:MSMS(+)[1130.0000] CE:47.0-57.0 RT:[6.870-7.244]
1.25e4
227.10269
1417.61353
1012.45994
599.11756
730.21247
1013.46223
1502.70197
401.22994
731.21566
1014.46576
1532.70332
1757.79598
1.0e4
5.0e3
0.0e0
200
400
600
800
1000
1200
1400
1600
1800
2000
2200
m/z

Coumarin-bivalirudin 24

LC/Q-TOF-MS/MS

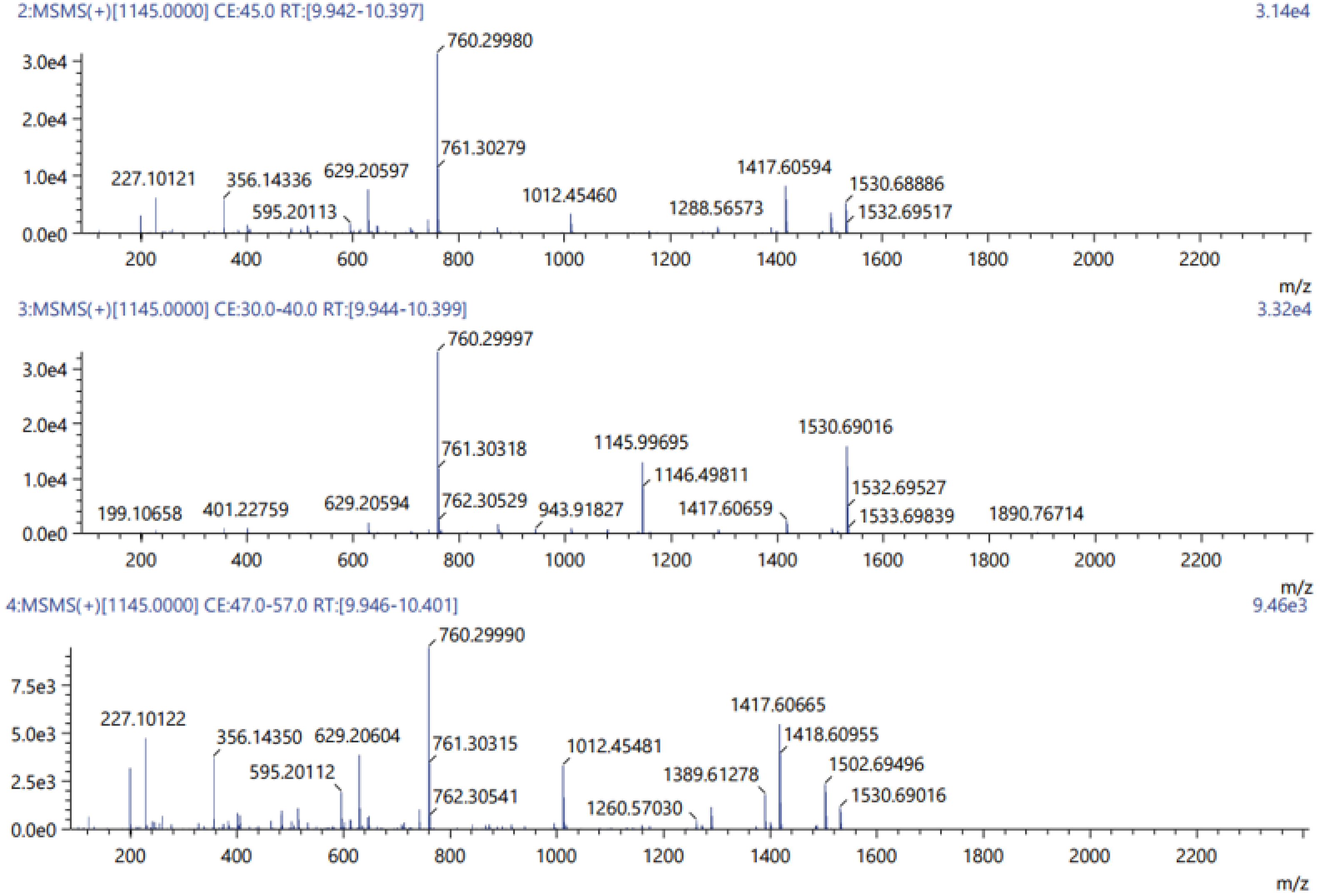

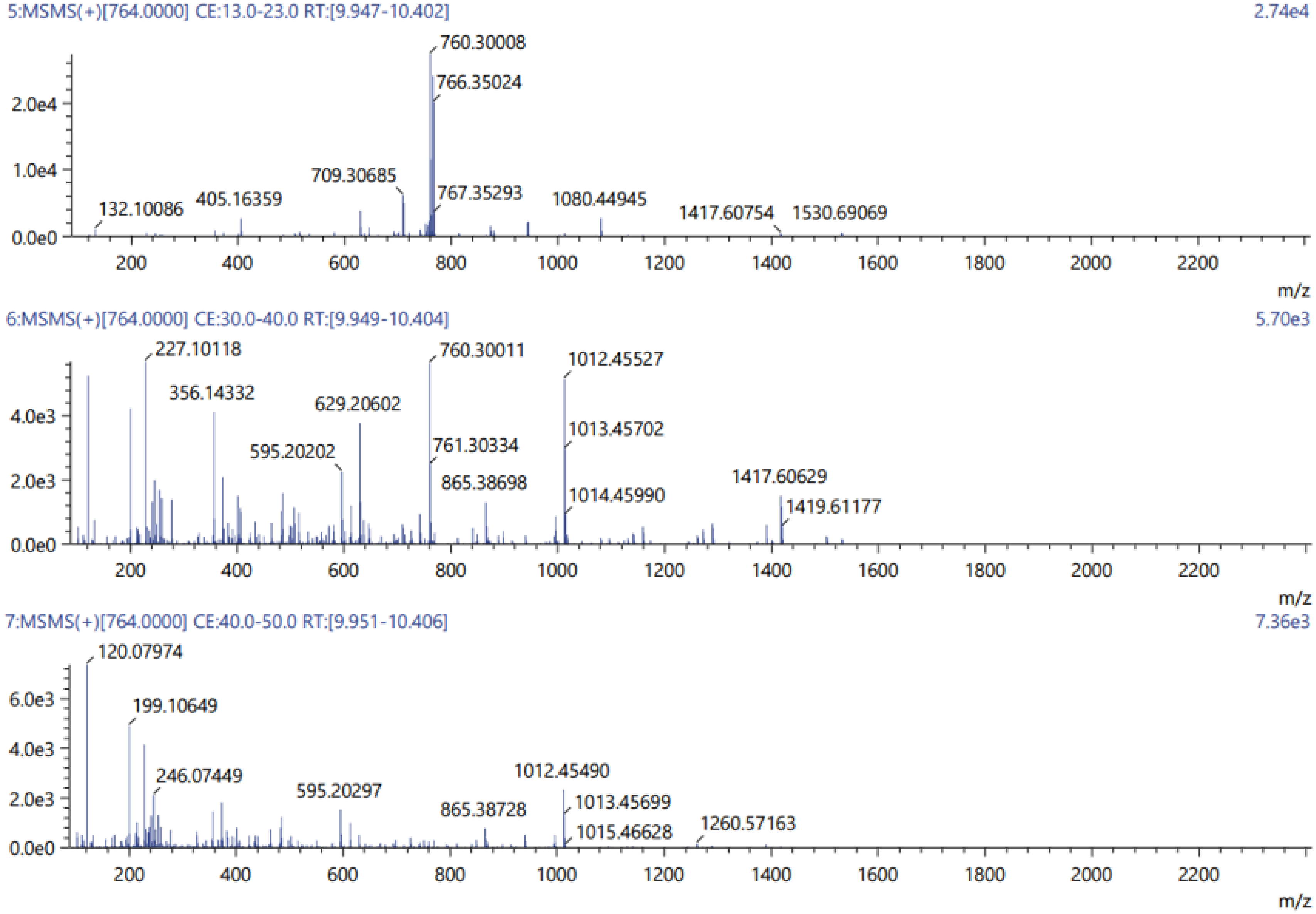
5:MSMS(+)[764.0000] CE:13.0-23.0 RT:[9.947-10.402]
2.74e4
760.30008
766.35024
709.30685
767.35293
1080.44945
132.10086
405.16359
1417.60754
1530.69069
2.0e4
1.0e4
0.0e0
200
400
600
800
1000
1200
1400
1600
1800
2000
2200
m/z
6:MSMS(+)[764.0000] CE:30.0-40.0 RT:[9.949-10.404]
5.70e3
227.10118
356.14332
629.20602
595.20202
760.30011
761.30334
865.38698
1012.45527
1013.45702
1014.45990
1417.60629
1419.61177
4.0e3
2.0e3
0.0e0
m/z
7:MSMS(+)[764.0000] CE:40.0-50.0 RT:[9.951-10.406]
7.36e3
120.07974
199.10649
246.07449
595.20297
865.38728
1012.45490
1013.45699
1015.46628
1260.57163
6.0e3
4.0e3
2.0e3
0.0e0
m/z

p-F-phenyl-bivalirudin 25

LC/Q-TOF-MS/MS

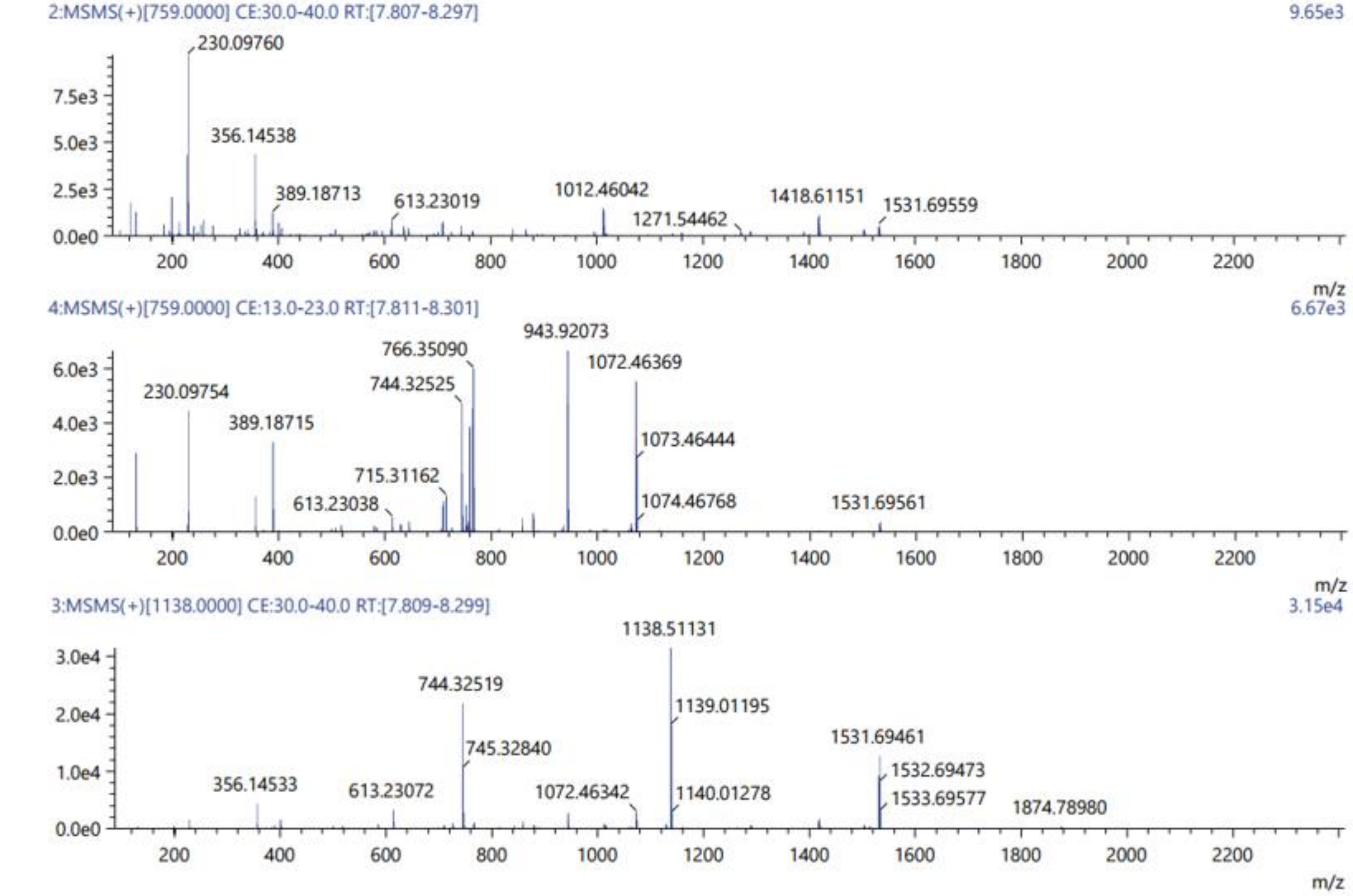

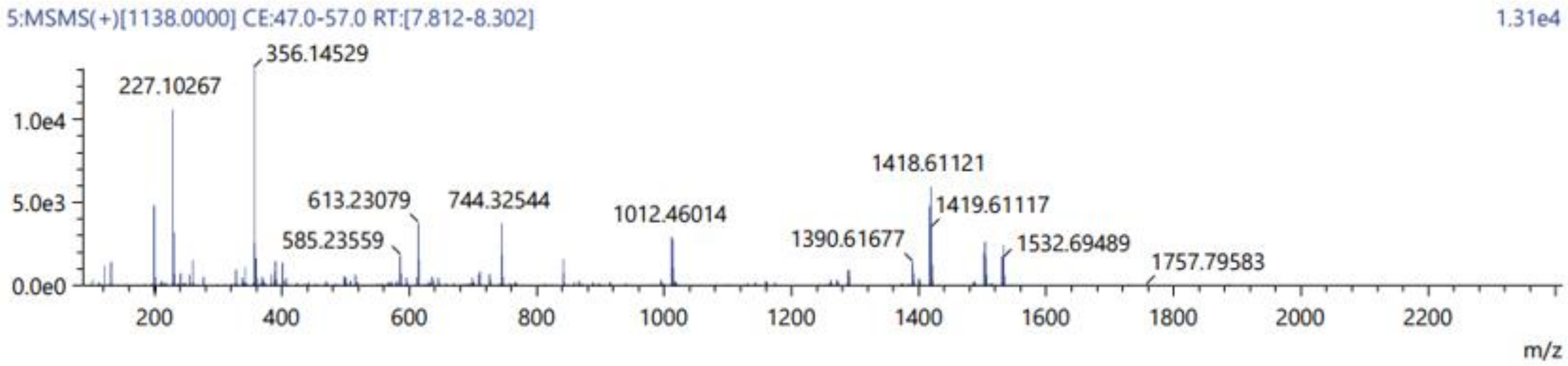
5:MSMS(+)[1138.0000] CE:47.0-57.0 RT:[7.812-8.302]
1.31e4
356.14529
227.10267
1.0e4
5.0e3
0.0e0
613.23079
744.32544
585.23559
1012.46014
1418.61121
1419.61117
1390.61677
1532.69489
1757.79583
200
400
600
800
1000
1200
1400
1600
1800
2000
2200
m/z

Iodo-insulin 26

HRMS

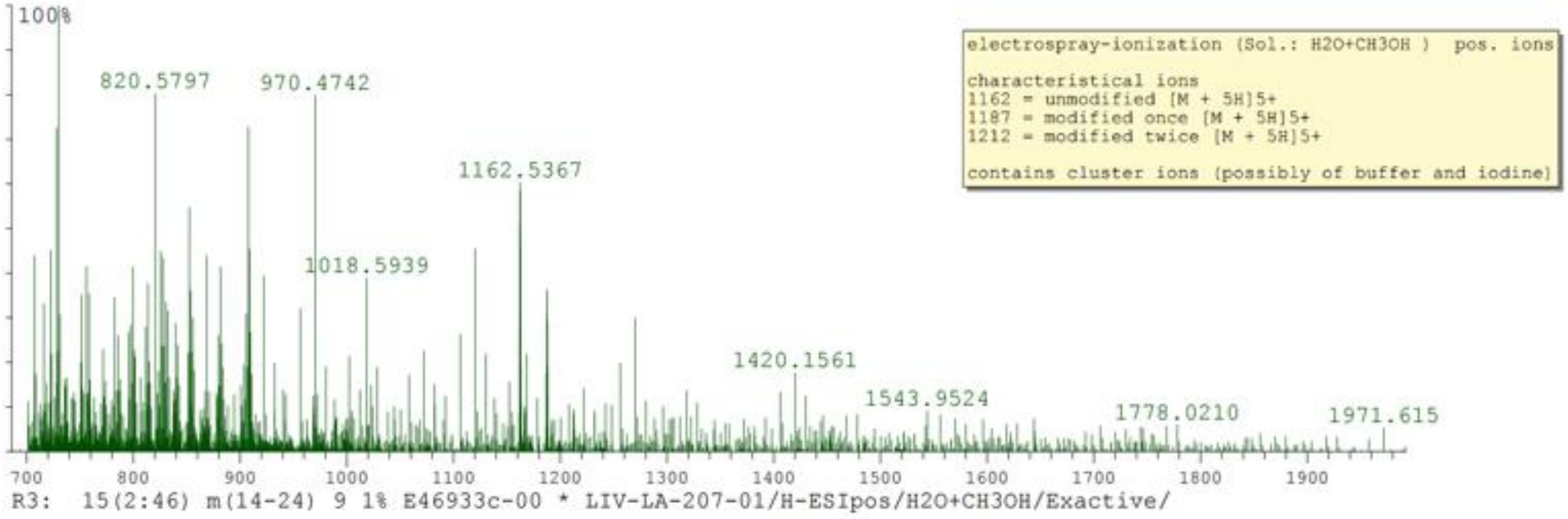

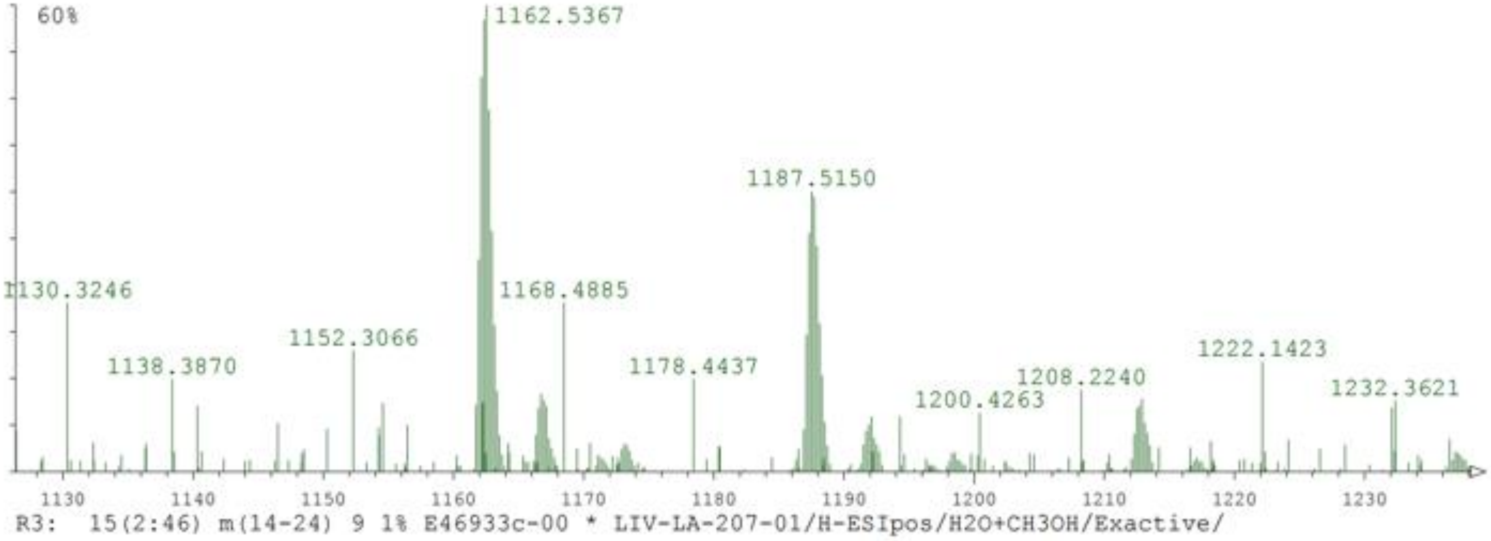

No.	Monoisotopic Mass	Sum Intensity	Number of Charge States	Average Charge	Delta Mass	Rel. Abundance	Fractional Abundance
1	5803,6384	64244,6	3	5,09	0	100	45,62

Charge State	Calculated Monoisotopic m/z	Monoisotopic Mass for This Charge	Mostabund m/z	Charge Normalized Intensity	Fit %
4	1451,9168	5803,6309	1452,6671	2833,34	96,3
5	1161,7349	5803,6372	1162,3362	52816,77	97,5
6	968,2803	5803,6341	968,7810	8594,53	97,9

No.	Monoisotopic Mass	Sum Intensity	Number of Charge States	Average Charge	Delta Mass	Rel. Abundance	Fractional Abundance
2	5825,6246	8976,6	2	5,06	21,9862	13,97	6,37
3	5857,5616	5079,6	2	5,45	53,9232	7,91	3,61
4	5929,5329	40109,0	3	5,07	125,8945	62,43	28,48

Charge State	Calculated Monoisotopic m/z	Monoisotopic Mass for This Charge	Mostabund m/z	Charge Normalized Intensity	Fit %
4	1483,3909	5929,5323	1484,1409	2193,74	87,9
5	1186,9142	5929,5336	1187,5156	32965,60	98,6
6	989,2631	5929,5312	989,7638	4949,70	93,7

No.	Monoisotopic Mass	Sum Intensity	Number of Charge States	Average Charge	Delta Mass	Rel. Abundance	Fractional Abundance
5	5951,5221	5690,0	2	5,04	147,8837	8,86	4,04
6	5983,4507	2727,7	2	5,52	179,8123	4,25	1,94
7	6055,4297	9354,5	2	5,13	251,7913	14,56	6,64

Charge State	Calculated Monoisotopic m/z	Monoisotopic Mass for This Charge	Mostabund m/z	Charge Normalized Intensity	Fit %
5	1212,0934	6055,4304	1212,6948	8184,35	97,5
6	1010,2457	6055,4298	1010,7462	1170,15	95,6

No.	Monoisotopic Mass	Sum Intensity	Number of Charge States	Average Charge	Delta Mass	Rel. Abundance	Fractional Abundance
8	6177,6352	3403,7	2	5,39	373,9968	5,30	2,42
9	6303,5277	1252,3	2	5,30	499,8893	1,95	0,89

LC/Q-TOF-MS and MS/MS for 26 after trypsin digestion and reduction

MS-TIC

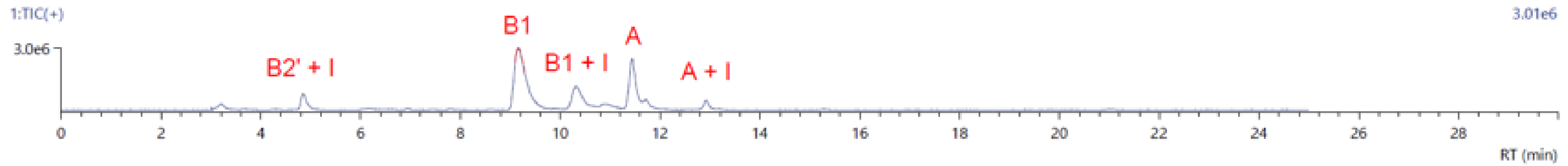

LC/Q-TOF-MS/MS (B2' chain + I)

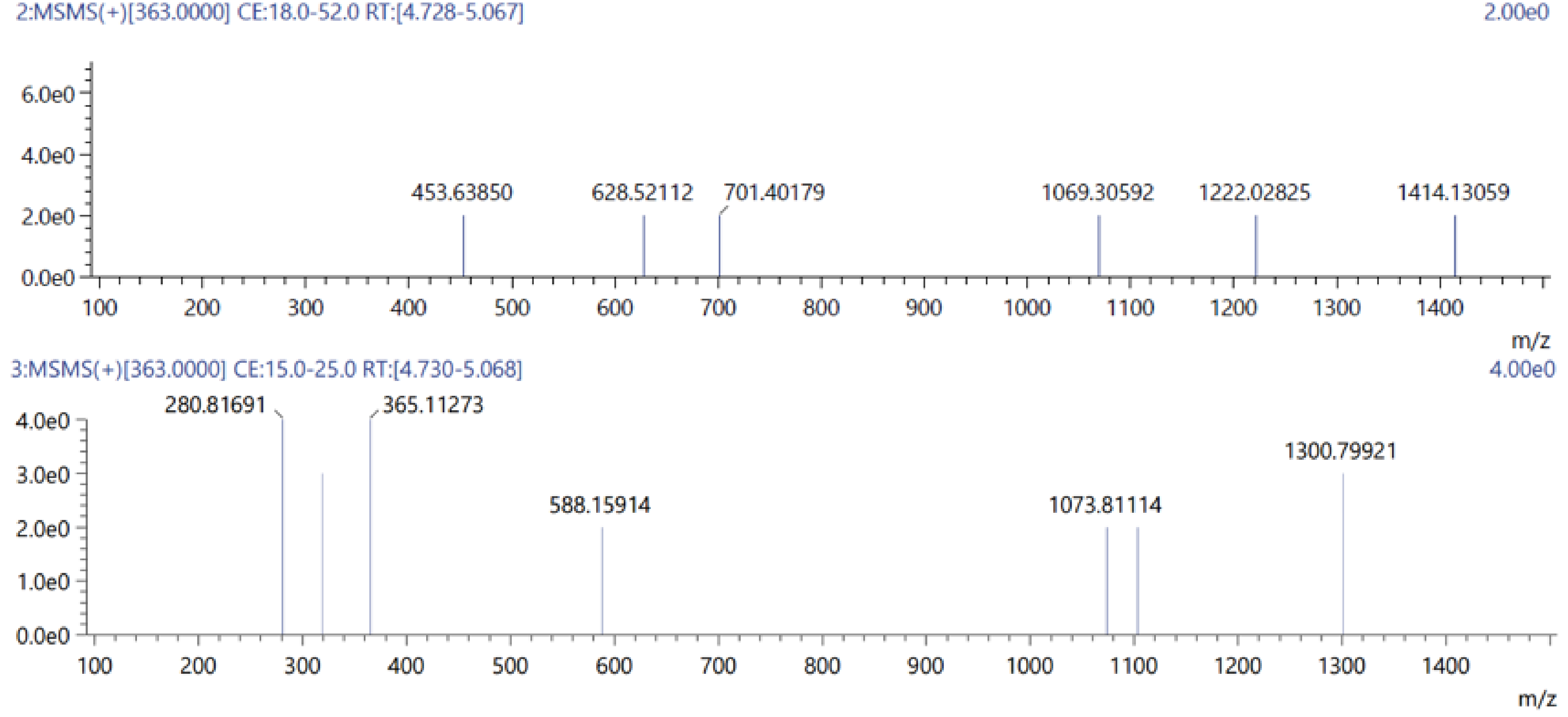

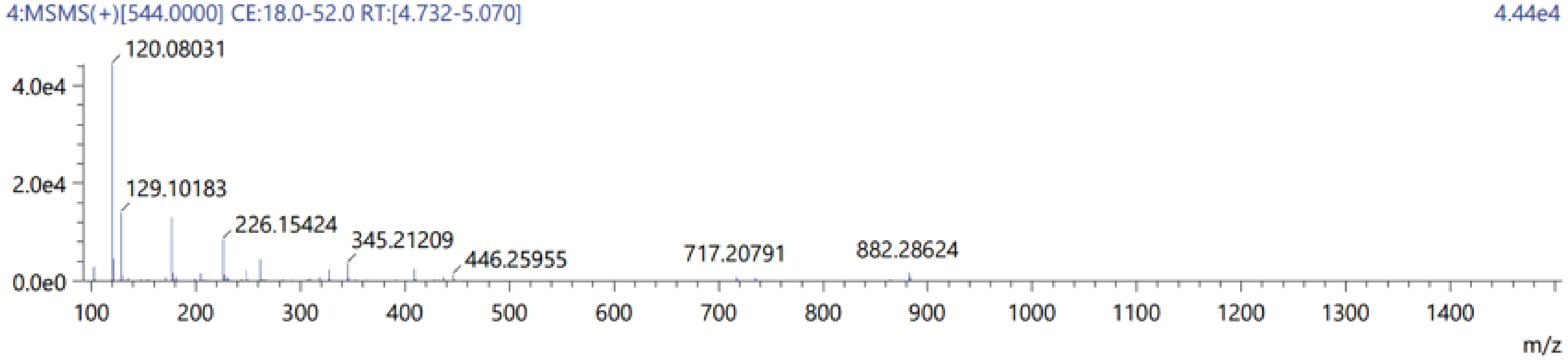
4:MSMS(+)[544.0000] CE:18.0-52.0 RT:[4.732-5.070]
4.44e4
120.08031
129.10183
226.15424
345.21209
446.25955
717.20791
882.28624
4.0e4
2.0e4
0.0e0
100 200 300 400 500 600 700 800 900 1000 1100 1200 1300 1400
m/z

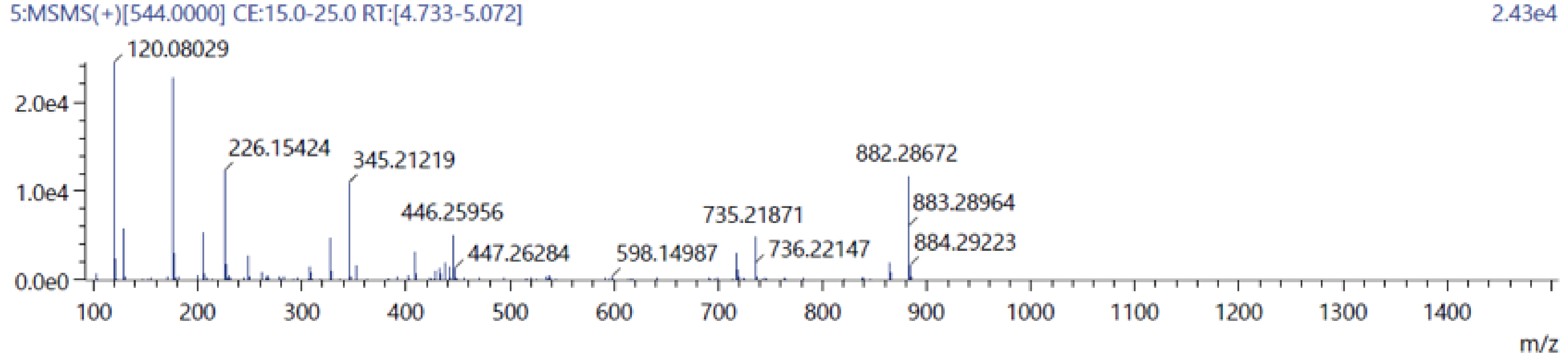
5:MSMS(+)[544.0000] CE:15.0-25.0 RT:[4.733-5.072]
2.43e4
120.08029
226.15424
345.21219
446.25956
447.26284
598.14987
735.21871
736.22147
882.28672
883.28964
884.29223
2.0e4
1.0e4
0.0e0
100 200 300 400 500 600 700 800 900 1000 1100 1200 1300 1400
m/z

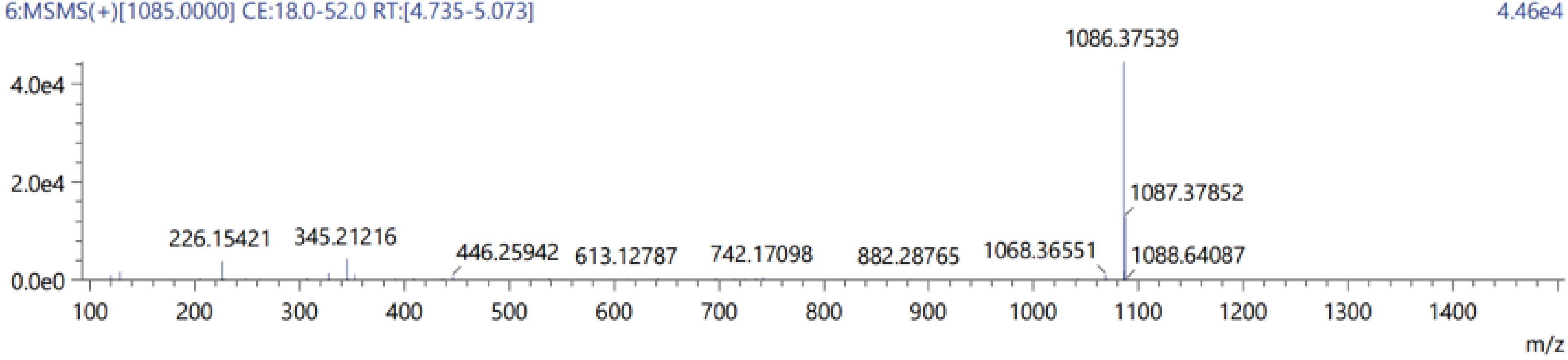
6:MSMS(+)[1085.0000] CE:18.0-52.0 RT:[4.735-5.073]
4.46e4
1086.37539
1087.37852
226.15421
345.21216
446.25942
613.12787
742.17098
882.28765
1068.36551
1088.64087
4.0e4
2.0e4
0.0e0
100 200 300 400 500 600 700 800 900 1000 1100 1200 1300 1400
m/z

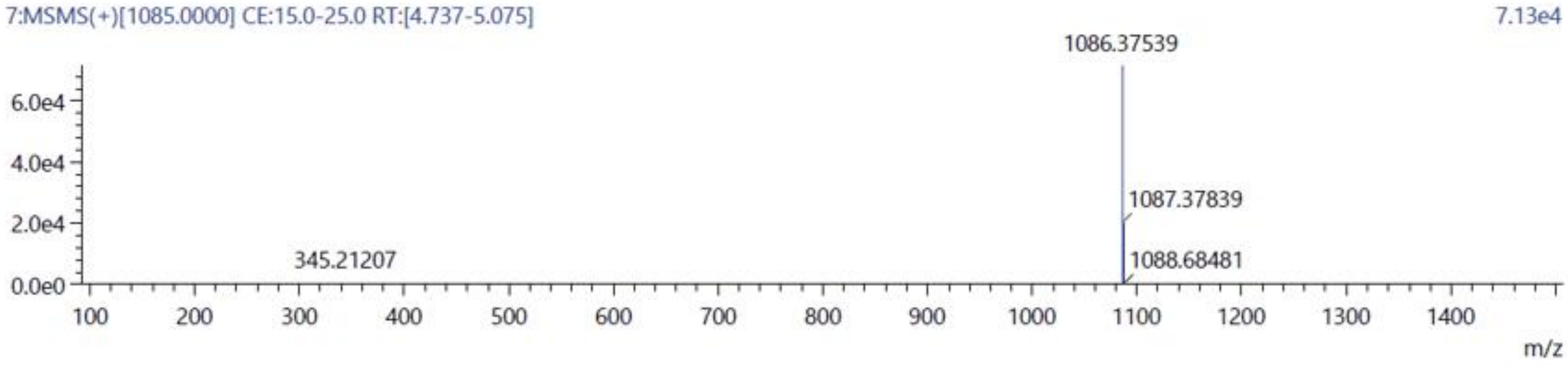

LC/Q-TOF-MS/MS (B1 chain + I)

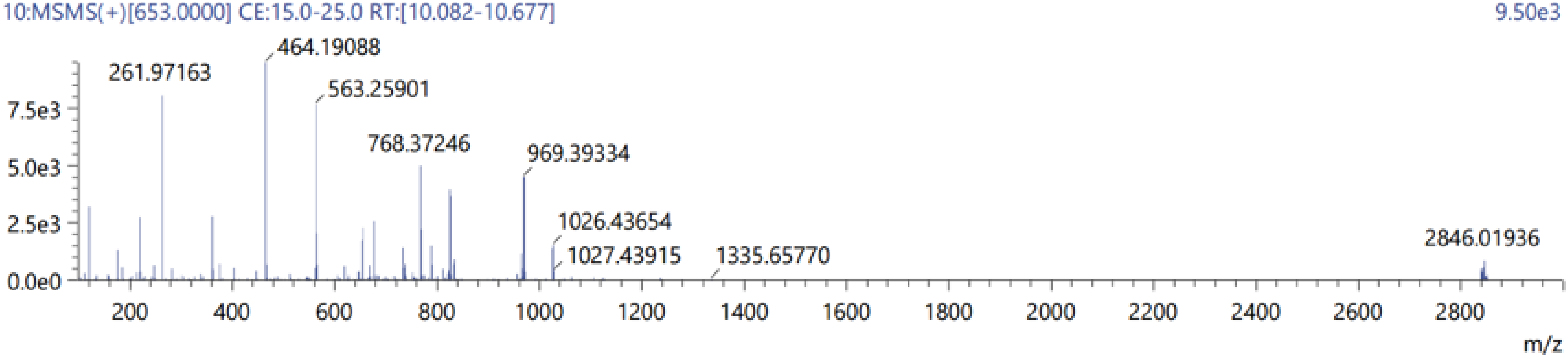

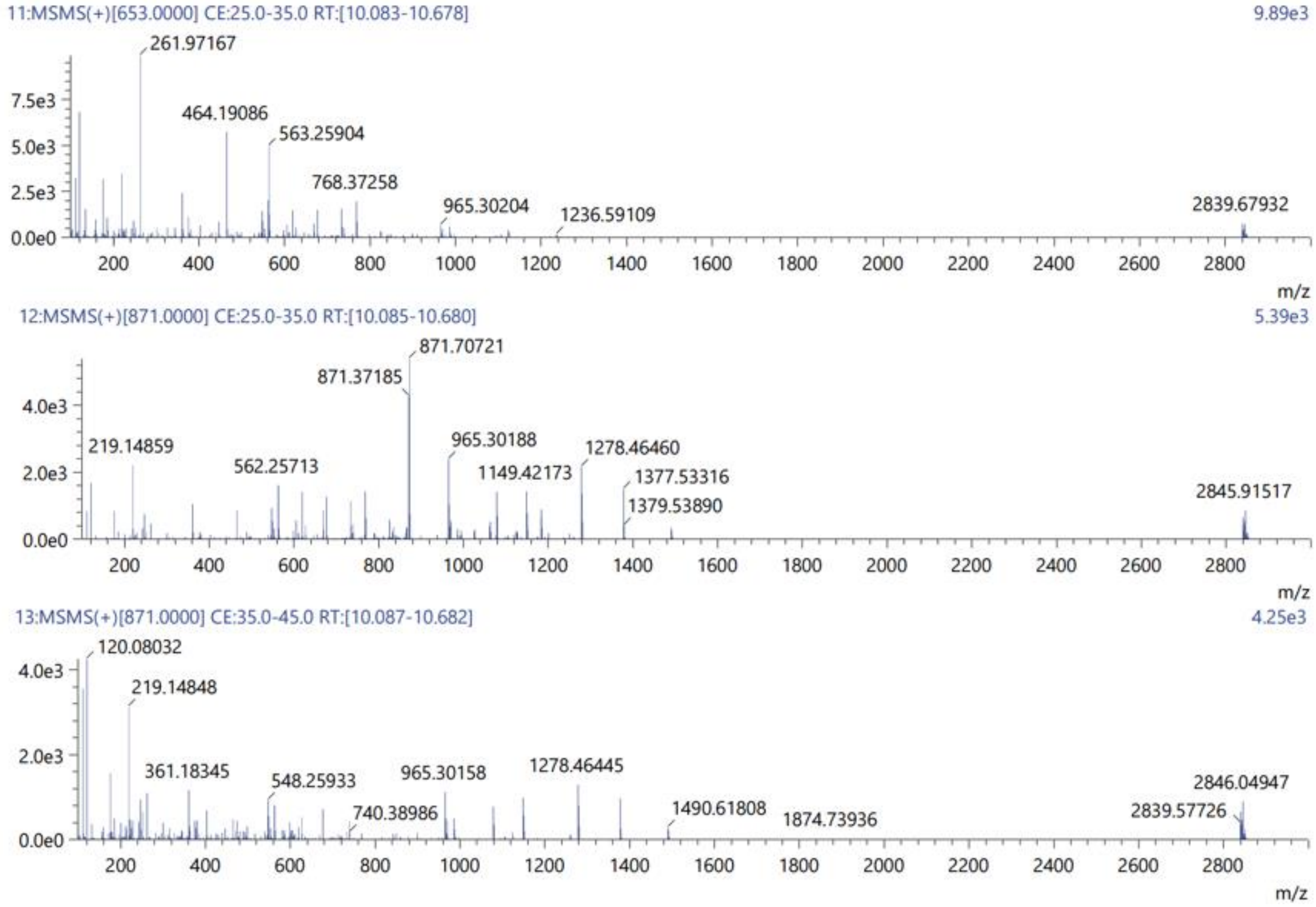
11:MSMS(+)[653.0000] CE:25.0-35.0 RT:[10.083-10.678]
9.89e3
261.97167
464.19086
563.25904
768.37258
965.30204
1236.59109
2839.67932
7.5e3
5.0e3
2.5e3
0.0e0
200 400 600 800 1000 1200 1400 1600 1800 2000 2200 2400 2600 2800
m/z
12:MSMS(+)[871.0000] CE:25.0-35.0 RT:[10.085-10.680]
5.39e3
871.70721
871.37185
219.14859
562.25713
965.30188
1149.42173
1278.46460
1377.53316
1379.53890
2845.91517
4.0e3
2.0e3
0.0e0
200 400 600 800 1000 1200 1400 1600 1800 2000 2200 2400 2600 2800
m/z
13:MSMS(+)[871.0000] CE:35.0-45.0 RT:[10.087-10.682]
4.25e3
120.08032
219.14848
361.18345
548.25933
740.38986
965.30158
1278.46445
1490.61808
1874.73936
2846.04947
2839.57726
4.0e3
2.0e3
0.0e0
200 400 600 800 1000 1200 1400 1600 1800 2000 2200 2400 2600 2800
m/z

LC/Q-TOF-MS/MS (A chain + I)

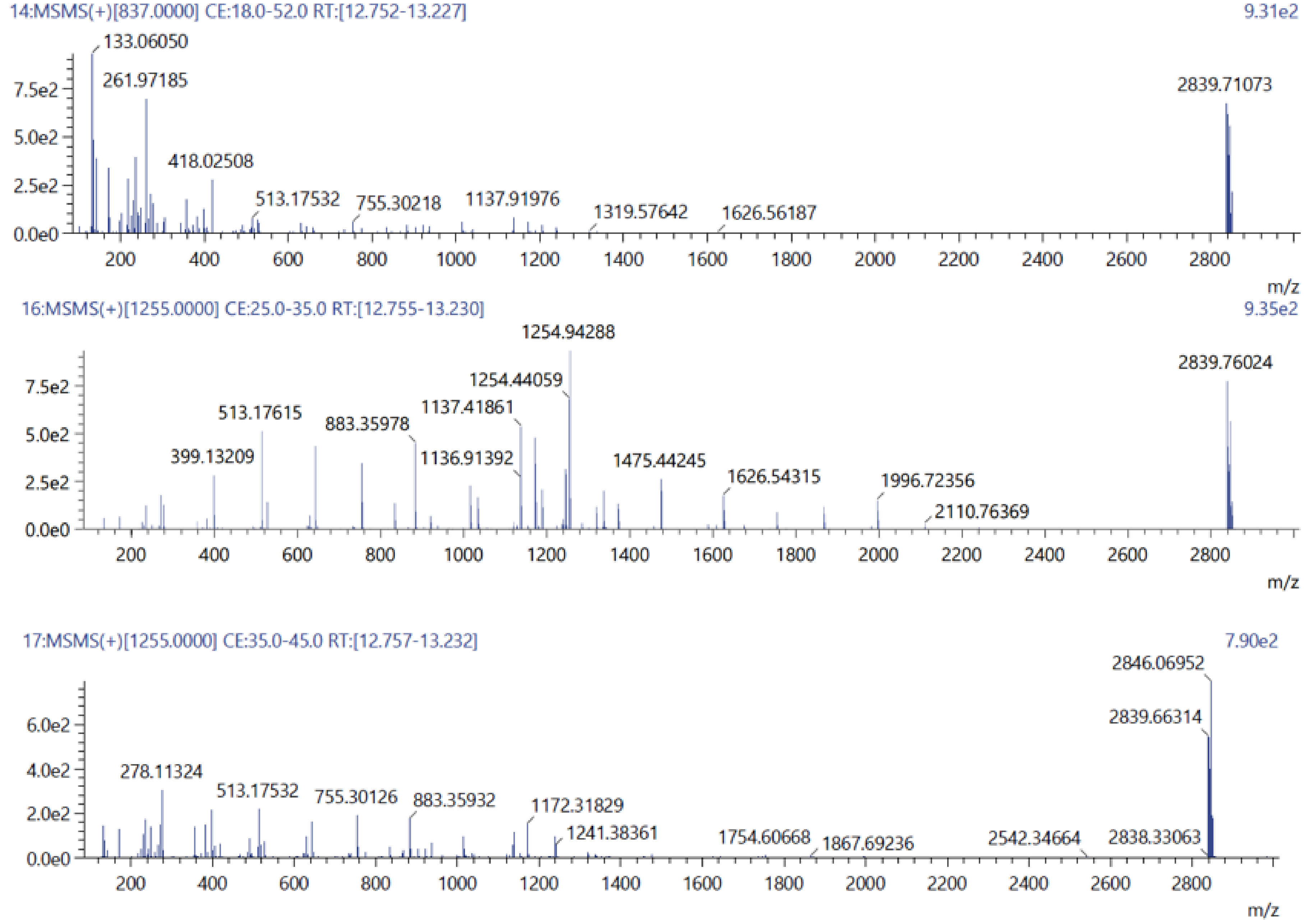

Bromo-ubiquitin 27

LC-FTMS of 27 after trypsin digestion

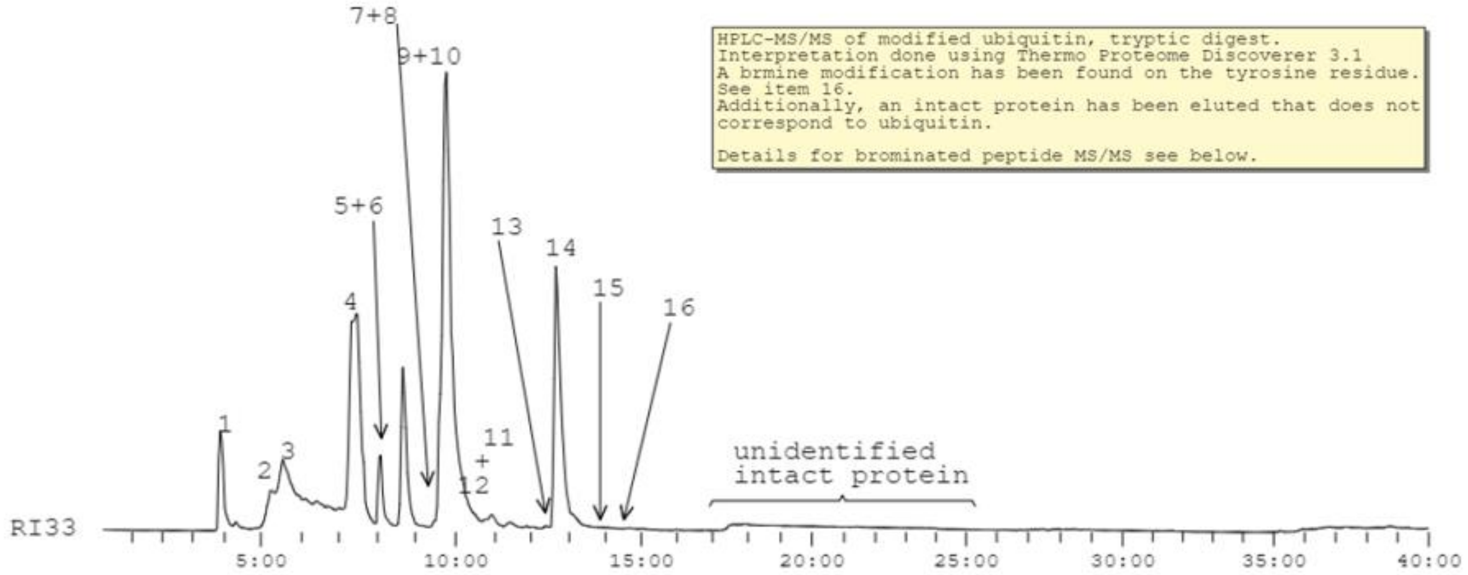

peptides identified (87% sequence coverage)

#	RT/min	Sequence	Mod.	theor. m/z		error/ppm	Conf.
1	3.97	QLEDGR		359.17947	(2+)	-1.29	1.78
2	5.83	IQDKEGIPPDQQR		762.39410	(2+)	-0.21	4.31
3	7.58	EGIPPDQQR		520.26166	(2+)	-0.63	2.27
4	7.67	MQIFVK	1×Ox [M1]	391.21753	(2+)	0.14	1.91
5	7.88	AKIQDKEGIPPDQQR		574.97638	(3+)	0.73	2.77
6	8.16	TLSDYNIQK		541.27954	(2+)	-0.56	2.66
7	9.15	QLEDGRTLSDYNIQK		593.96741	(3+)	0.28	2.24
8	9.39	MQIFVKTLTGK	1×Ox [M1]	427.91290	(3+)	0.50	3.17
9	9.74	MQIFVK		383.21979	(2+)	-0.60	1.80
10	10.08	ESTLHLVLR		534.31409	(2+)	0.12	2.88
11	10.91	MQIFVK	1×Ac [N-Term]	404.22525	(2+)	-0.12	1.40
12	11.48	TITLEVEPSDTIENVKAK		663.02496	(3+)	0.48	4.33
13	12.54	TLTGKTITLEVEPSDTIENVK		763.41309	(3+)	0.68	2.35
14	12.72	TITLEVEPSDTIENVK		894.46729	(2+)	0.00	4.86
15	13.98	TLSDYNIQKESTLHLVLR		710.72253	(3+)	-1.08	2.82
16	14.64	TLSDYNIQKESTLHLVLR	1×Br [Y5]	736.69379	(3+)	0.43	3.26

MS/MS for the peptide 16

MS/MS ions found for #2 (mass error in ppm)

#1	b+	Seq.	y+	y2+	#2
1		T			18
2	215.13844 (+2.69)	L			17
3		S			16
4		D			15
5		Y-Bromo			14
6		N			13
7		I			12
8		Q	1323.78088 (-4.96)		11
9		K	1195.71094 (+4.01)		10
10		E	1067.62109 (-0.30)		9
11		S	938.57697 (+1.29)		8
12		T	851.54376 (+2.80)		7
13		L	750.49744 (+1.37)		6
14					

Internal Fragment	ya+	yb+
ya6-7	200.13940 (-0.23)	
yb3-4		203.06587 (1.86)
yb3-5		444.03516 (11.1)
yb3-6		558.08191 (1.98)
yb5-6		356.02289 (3.24)
yb12-13		215.13844 (2.69)
yb13-14		251.14986 (1.56)
yb14-15		251.14986 (1.56)

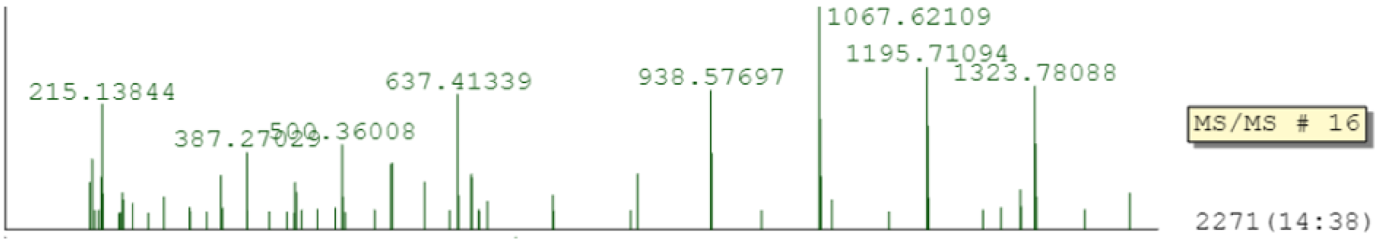

REFERENCES

1. Berger, F., Plutschack, M. B., Riegger, J. *et al.* Site-selective and versatile aromatic C−H functionalization by thianthrenation. *Nature* **567**, 223–228 (2019).
2. Xu, P. et al. Site‐Selective Late‐Stage Aromatic [18F] Fluorination via Aryl Sulfonium Salts. *Angew. Chem. Int. Ed.* **59***,* 1956–1960 (2020).
3. Liljeblad, A., Aksela, R. & Kanerva, L. T. Use of enantio-, chemo-and regioselectivity of acylase I. Resolution of polycarboxylic acid esters. *Tetrahedron: Asymmetry* **12**, 2059–2066 (2001).
4. Fulmer, G. R. et al. NMR Chemical Shifts of Trace Impurities: Common Laboratory Solvents, Organics, and Gases in Deuterated Solvents Relevant to the Organometallic Chemist. *Organometallics* **29**, 2176–2179 (2010).
5. Harris, R. K. et al. Further conventions for NMR shielding and chemical shifts (IUPAC Recommendations 2008). *Pure Appl. Chem.* **80**, 59–84 (2008)
6. Marty, M. T. *et al.* Bayesian Deconvolution of Mass and Ion Mobility Spectra: From Binary Interactions to Polydisperse Ensembles. *Analytical Chemistry* **87**, 4370–4376 (2015).
7. Juliá, F., Shao, Q., Duan, M. *et al.* High site selectivity in electrophilic aromatic substitutions: mechanism of C–H thianthrenation. *J. Am. Chem. Soc.* **143**, 16041–16054 (2021).
8. Hadži, D., Klofutar, C. & Oblak, S. Hydrogen bonding in some adducts of oxygen bases with acids. Part IV. Basicity in hydrogen bonding and in ionisation. *J. Chem. Soc. A*, 905–908 (1968).
9. Origin, Version 2019b. OriginLab Corporation, Northampton, MA, USA.
10. Umezawa, T., Sugihara, Y., Ishii, A. & Nakayama, J. Synthesis and properties of monocyclic selenophene 1-oxides. *J. Am. Chem. Soc*. **120**, 12351–12352 (1998).
11. Nakanishi, W., Ikeda, Y. & Iwamura, H. The structure of 2-carboxyphenyl methyl selenoxide, its sodium salt and related compounds in solution, studied by 1H, 13C and 77Se NMR. *Org. Magn. Reson.* **20**, 117–122 (1982).
12. He, X., Wang, X., Tse, Y. L., Ke, Z. & Yeung, Y. Y. Applications of selenonium cations as lewis acids in organocatalytic reactions. *Angew. Chem. Int. Ed.* **57**, 12869–12873 (2018).
13. Martin, G. J., Martin, M. L. & Gouesnard, J.-P. in *15N-NMR spectroscopy* 127–128 (Springer, 1981).
14. Iwaoka, M. & Tomoda, S. Nature of the Intramolecular Se···N Nonbonded Interaction of 2-Selenobenzylamine Derivatives. An Experimental Evaluation by 1H, 77Se, and 15N NMR Spectroscopy. *J. Am. Chem. Soc*. **118**, 8077–8084 (1996).
15. Hull, W. E., Kricheldorf, H. R. & Fehrle, M. 15N-NMR spectroscopy. IV. Comparison of poly (L-lysine) and isopoly (L-lysine). *Biopolymers* **17**, 2427–2443 (1978).
16. Eaton, G., Symons, M. C. & Rastogi, P. P. Spectroscopic studies of the solvation of amides with N–H groups. part 1. –the carbonyl group. *J. Chem. Soc., Faraday Trans. 1* **85**, 3257–3271 (1989).
17. Dhami, K. S. & Stothers, J. B. Carbon-13 carbonyl shielding and steric inhibition of resonance a new and better method for estimating angles of twist in conjugated systems. *Tetrahedron Lett.* **5**, 631–639 (1964).
18. Semenov, V. A., Samultsev, D. O. & Krivdin, L. B. Theoretical and experimental study of ^{15}N NMR protonation shifts. *Magn. Reson. Chem.* **53**, 433–441 (2015).
19. Busto-Moner, L., Feng, C. J., Antoszewski, A., Tokmakoff, A. & Dinner, A. R. Structural ensemble of the insulin monomer. *Biochemistry* **60**, 3125–3136 (2021).
20. Hua, Q. X., Jia, W. & Weiss, M. A. Conformational dynamics of insulin. *Front. Endocrinol.*,**2**, 48 (2011).
21. Hughes, C. S. et al. Single-pot, solid-phase-enhanced sample preparation for proteomics experiments. *Nat. Protoc.* **14**, 68-85, doi: 10.1038/s41596-018-0082-x (2019).
22. Herriott, R. M. Identification of mono-iodotyrosine from iodinated pepsin. *J. Gen. Physiol.* **31**, 19 (1947).
23. Neese, F. The ORCA program system. WIREs *Comput. Mol. Sci.* **2**, 73–78 (2012).
24. Lee, C., Yang, W. & Parr, R. G. Development of the Colle-Salvetti correlation-energy formula into a functional of the electron density. *Phys. Rev. B* **37**, 785–789 (1988).
25. Grimme, S., Antony, J., Ehrlich, S. & Krieg, H. A consistent and accurate ab initio parametrization of density functional dispersion correction (DFT-D) for the 94 elements H–Pu. *J. Chem. Phys.* **132**, 154104 (2010).
26. Grimme, S., Ehrlich, S. & Goerigk, L. Effect of the damping function in dispersion corrected density functional theory. *J. Comput. Chem.* **32**, 1456–1465 (2011).
27. Neese, F., Wennmohs, F., Hansen, A. & Becker, U. Efficient, caimate and parallel Hartree–Fock and hybrid DFT calculations. A ‘chain-of-spheres’ algorithm for the Hartree–Fock exchange. *Chem. Phy.* **356**, 98–109 (2009).
28. Weigend, F. Accurate Coulomb-fitting basis sets for H to Rn. *Phys. Chem. Chem. Phys.* **8**, 1057–1065 (2006).

29. Weigend, F. & Ahlrichs, R. Balanced basis sets of split valence, triple zeta valence and quadruple zeta valence quality for H to Rn: Design and assessment of accuracy, *Phys. Chem. Chem. Phys.* **7**, 3297–3305 (2005).
30. Barone, V. & Cossi, M. Quantum calculation of molecular energies and energy gradients in solution by a conductor solvent model, *J. Phys. Chem. A* **102**, 1995–2001 (1998).
31. Hanwell, M. D. et al. Avogadro: an advanced semantic chemical editor, visualization, and analysis platform. *J. Cheminform.* **4**, 17 (2012).
32. Zhurko, G. A., & Zhurko, D. A. Chemcraft-graphical program for visualization of quantum chemistry computations. https://chemcraftprog.com.
